# Association of total sleep duration variability with risk of new stroke in the middle-aged and elderly Chinese population

**DOI:** 10.1186/s12883-024-03727-8

**Published:** 2024-06-25

**Authors:** Jiangping Ma, Nuo Ma, Lu Zhang, Linghao Xu, Xueyuan Liu, Guilin Meng

**Affiliations:** 1 Department of Neurology, Shanghai Tenth People’s Hospital, Tongji University School of Medicine, Shanghai, China; 2 Department of Neurology, Tongren Hospital, Shanghai Jiao Tong University School of Medicine, Shanghai, China; 3 Department of Cardiology, Shanghai East Hospital, Tongji University School of Medicine, Shanghai, China

**Keywords:** Sleep duration, Variability, Standard deviation, Stroke, Sex

## Abstract

**Objective:**

To investigate the association between total sleep duration variability and stroke in the middle-aged and elderly population in China.

**Methods:**

Data were collected from the 2011, 2013, 2015, and 2018 surveys of the China Health and Retirement Longitudinal Study (CHARLS). A total of 3485 participants, who had not experienced a stroke until 2015 and completed the follow-up in 2018, were enrolled to analyze the relationship between total sleep duration variability and new stroke. Total sleep duration was calculated by summing self-reported nocturnal sleep duration and daytime napping. The variability was determined by calculating the standard deviation (SD) of total sleep duration across the first three waves. A binary logistic regression model was utilized to analyze this association.

**Results:**

Of the 3485 participants, 183 (5.25%) sustained a stroke event. A dose-response relationship was observed, indicating an increased stroke risk of 0.2 per unit (hours) increase in total sleep duration variability [OR (95% CI): 1.20 (1.01–1.42)]. Upon stratification by sex groups, this increased risk was significant only in men [OR (95% CI): 1.44 (1.12–1.83)].

**Conclusion:**

Increased total sleep duration variability was associated with an increased risk of stroke in the middle-aged and elderly, independent of factors such as age, nocturnal sleep duration, napping habits, region of residence, hypertension, diabetes mellitus, dyslipidemia, BMI, smoking, drinking habits, and marital status. However, a more notable correlation was observed in males.

**Supplementary Information:**

The online version contains supplementary material available at 10.1186/s12883-024-03727-8.

## Introduction

Early prevention of stroke—a clinical condition characterized by neurological deficits arising from disrupted cerebral blood flow [1],—is crucial, given its status as the second leading cause of death and the third leading cause of disability in adults globally [2]. Stroke causes are diverse, encompassing hypertension, diabetes, obesity, advanced age, smoking, and dyslipidemia. However, these traditional risk factors do not account for all stroke occurrences [3–7]. Recent research increasingly highlights the impact of sleep disturbances on stroke development [8–11].

Sleep, a vital physiological activity, is crucial for maintaining physical and mental health. Numerous studies have explored the correlation between sleep duration and stroke risk, suggesting non-linear [12], J-shaped [13], or U-shaped relationships [14]. Collectively, our study’s findings suggest that both excessive and insufficient sleep increase stroke risk [12–15].

In addition to sleep duration, sleep variability is an important indicator of sleep health [16]. Sleep variability refers to the regularity of sleep/wake patterns [17]. Excessive sleep variability can disrupt internal circadian rhythms, adversely affecting metabolism [18]. Research indicates that sleep variability is linked to elevated risks of obesity, dyslipidemia, hyperglycemia, hypertension, and metabolic syndrome [19].

However, the association between sleep duration variability and stroke remains underexplored [20]. This study aims to examine the link between total sleep duration variability and new stroke incidents, including sex-specific differences, in a middle-aged and elderly Chinese cohort (China Health and Retirement Longitudinal Study[CHARLS]).

## Methods

### Study population

CHARLS, a prospective, nationally representative, and population-based study, focuses on the economic, social, and health status of adults aged 45 and above. Commenced in 2011 with 17,596 randomly selected participants, the study has examined data on general health status, lifestyle habits, and demographic characteristics at two-year intervals. To date, four waves of data are available. The protocol received approval from the Ethical Review Committee of Peking University (approval numbers: IRB00001052-11015 for the main household survey and IRB00001052-11014 for biomarker collection). All participants signed the informed consent and repository consent that permitted their data to be shared after a detailed presentation of the risks and benefits related to study participation.

**Fig. 1:**
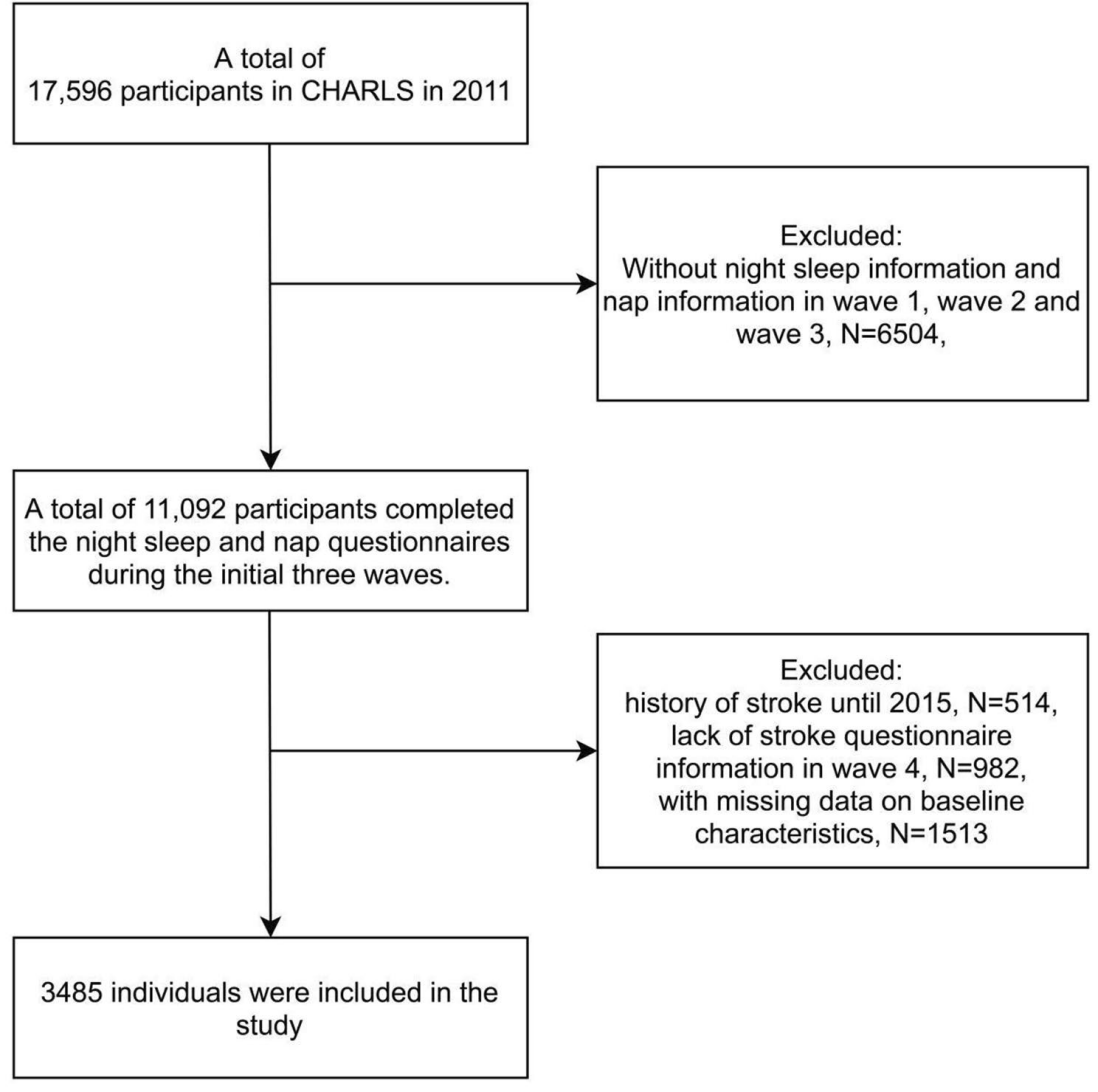
Flowchart of the study population

For this study, we excluded individuals with incomplete responses in the first three rounds of the sleep questionnaire (Supplementary material). Additionally, we excluded subjects who reported a stroke episode in the 2011, 2013, and 2015 surveys, and those with missing information in the 2018 stroke questionnaire (Supplementary material). We also excluded individuals missing complete information from the 2011 baseline. Ultimately, we analyzed a sample of 3,485 participants to investigate the association between total sleep duration variability and new stroke incidence. Further details about the enrollment process are provided in Fig. 1.

### Assessment of total sleep duration variability

The total sleep duration over 24 h for each participant was calculated by adding the nocturnal sleep duration and daytime napping together [21]. Nocturnal sleep duration was obtained through the question: “In the previous month, how many hours of actual sleep did you get each night?” Meanwhile, daytime napping was assessed with: “In the previous month, how long did you nap for after lunch?“. For each participant, the standard deviation of total sleep duration was calculated using the total sleep time data from 2011, 2013, and 2015, representing the variability in total sleep duration.

### Assessment of stroke

Stroke was the primary outcome of this study. Stroke incidence was assessed through self-reporting with the question: “Has a physician previously informed you that you have had a stroke?” An affirmative response was deemed indicative of a stroke occurrence. The primary outcome of interest was the occurrence of the first stroke event following the 2015 survey.

### Assessment of covariates

The selection of covariates was guided by previous studies [11]. The selected socio-demographic characteristics included age, gender (male/female), educational attainment (primary and below/junior to secondary/undergraduate and above), marital status (married/unmarried), and residency (rural/urban). The categories “Married with spouse present” and “Married but not living with spouse” were classified as married, while “Separated”, “Divorced”, “Widowed”, and “Never married” were classified as unmarried. Lifestyle behaviors included smoking status (never/ever), alcohol consumption status (never/ever), and physical activity level (light/moderate/vigorous). Smoking status was categorized as “Never smoked” and “Ever smoked”, and alcohol consumption as “Never drunk” and “Ever drunk”. Clinical indicators included self-reported diagnoses of hypertension, diabetes mellitus, dyslipidemia, and body mass index (BMI). Nocturnal sleep duration and daytime napping at baseline were also considered to control for covariates.

### Statistical analyses

Continuous variables were presented as the mean (SD) and compared using t-tests, while categorical variables were presented as counts and proportions and compared using Chi-square tests. Multivariable logistic regression analysis was used to examine the longitudinal correlation between total sleep duration variability and the incidence of new strokes. The multicollinearity of covariates (age, gender, educational attainment, marital status, residence, physical activity, smoking status, drinking status, hypertension, diabetes, BMI, dyslipidemia, nocturnal sleep duration, and daytime napping) was checked using the Variance Inflation Factor (VIF). A potential non-linear association between total sleep duration variability and stroke onset was examined using restricted cubic spline regression. In addition, subgroup and interaction analyses were performed. Statistical analyses were performed with R software, version 4.2.2 (http://www.R-project.org/). A two-sided *P*-value of < 0.05 was considered statistically significant.

## Results

### Baseline characteristics of study participants

During a 3-year follow-up period, 183 (5.25%) of 3485 participants without prior stroke history in 2015 developed a stroke. Statistically significant differences in age, marital status, and history of dyslipidemia, hypertension, and diabetes mellitus were observed, as depicted in Table 1. Compared with participants without a stroke, those with a stroke were more likely to be older (60.1 vs. 57.1), unmarried (14.8% vs. 9.0%), have diabetes (12.0% vs. 4.8%), have hypertension (44.3% vs. 21.1%), or have dyslipidemia (16.9% vs. 8.5%).

**Table 1:** Baseline characteristics of participants by stroke occurrence

Characteristics{ (*n*,%) or [mean (SD)]}	Stroke(*n* = 3485)		*P*-overall
	No	Yes	
**Participants(n)**	3302	183	
**Age, y[mean (SD)]**	57.1 (8.79)	60.1 (7.82)	<0.001
**Gender(n,%)**			0.514
Male	1461 (44.2%)	86 (47.0%)	
Female	1841 (55.8%)	97 (53.0%)	
**Residence(n,%)**			0.096
Rural	1110 (33.6%)	73 (39.9%)	
Urban	2192 (66.4%)	110 (60.1%)	
**Marital Status(n,%)**			0.012
Separated/Divorced/Widowed/Never Married	296 (9.0%)	27 (14.8%)	
Married	3006 (91.0%)	156 (85.2%)	
**Education(n,%)**			1.000
Primary and below	2261 (68.5%)	126 (68.9%)	
Junior to secondary	1015 (30.7%)	56 (30.6%)	
Undergraduate and above	26 (0.79%)	1 (0.55%)	
**Smoking Status(n,%)**			0.345
Never	2126 (64.4%)	111 (60.7%)	
Ever	1176 (35.6%)	72 (39.3%)	
**Drinking Status(n,%)**			0.885
Never	2066 (62.6%)	113 (61.7%)	
Ever	1236 (37.4%)	70 (38.3%)	
**Exercise(n,%)**			0.100
Light	1232 (37.3%)	82 (44.8%)	
Moderate	2069 (62.7%)	101 (55.2%)	
Vigorous	1 (0.03%)	0 (0.00%)	
**Diabetes(n,%)**			<0.001
No	3144 (95.2%)	161 (88.0%)	
Yes	158 (4.8%)	22 (12.0%)	
**Hypertension(n,%)**			<0.001
No	2604 (78.9%)	102 (55.7%)	
Yes	698 (21.1%)	81 (44.3%)	
**Dyslipidemia(n,%)**			<0.001
No	3021 (91.5%)	152 (83.1%)	
Yes	281 (8.5%)	31 (16.9%)	
**BMI[mean (SD)]**	24.3 (31.1)	24.2 (3.60)	0.869
**Napping duration[mean (SD)]**	31.0 (41.2)	32.8 (42.7)	0.564
**Nocturnal sleep duration[mean (SD)]**	6.45 (1.81)	6.39 (1.85)	0.691

Compared to women, men were more likely to be older, married, highly educated, and have a history of smoking or drinking, with longer daytime napping and nocturnal sleep durations, as depicted in Table 2. However, they were less likely to have diabetes, hypertension, or dyslipidemia.

**Table 2:** Baseline characteristics of the study population by gender

Characteristics [(*n*,%) or mean (SD)]	Gender(*n* = 3485)		*P*-Value
	Male	Female	
**Participants (n)**	1547	1938	
**Age, Years [Mean (SD)]**	58.5 (8.52)	56.3 (8.83)	<0.001
**Residence (n,%)**			0.231
Rural	508 (32.8%)	675 (34.8%)	
Urban	1039 (67.2%)	1263 (65.2%)	
**Marital Status (n,%)**			0.001
Separated/Divorced/Widowed/Never Married	114 (7.37%)	209 (10.8%)	
Married	1433 (92.6%)	1729 (89.2%)	
**Education Level (n,%)**			<0.001
Primary and Below	890 (57.5%)	1497 (77.2%)	
Junior to Secondary	637 (41.2%)	434 (22.4%)	
Undergraduate and Above	20 (1.29%)	7 (0.36%)	
**Smoking Status (n,%)**			0.000
Never Smoked	417 (27.0%)	1820 (93.9%)	
Ever Smoked	1130 (73.0%)	118 (6.09%)	
**Alcohol Consumption (n,%)**			<0.001
Never Drank	512 (33.1%)	1667 (86.0%)	
Ever Drank	1035 (66.9%)	271 (14.0%)	
**Exercise Frequency (n,%)**			0.922
Light	586 (37.9%)	728 (37.6%)	
Moderate	961 (62.1%)	1209 (62.4%)	
Vigorous	0 (0.00%)	1 (0.05%)	
**Diabetes Status (n,%)**			0.039
No	1481 (95.7%)	1824 (94.1%)	
Yes	66 (4.27%)	114 (5.88%)	
**Hypertension Status (n,%)**			0.047
No	1226 (79.3%)	1480 (76.4%)	
Yes	321 (20.7%)	458 (23.6%)	
**Dyslipidemia Status (n,%)**			0.012
No	1430 (92.4%)	1743 (89.9%)	
Yes	117 (7.56%)	195 (10.1%)	
**BMI [Mean (SD)]**	23.3 (12.5)	25.1 (39.1)	0.060
**Napping Duration [Mean (SD)]**	36.8 (42.9)	26.5 (39.3)	<0.001
**Nocturnal Sleep Duration [Mean (SD)]**	6.54 (1.76)	6.37 (1.86)	0.006
**New Stroke Incidence (n, %)**			0.514
No	1461 (94.4%)	1841 (95.0%)	
Yes	86 (5.56%)	97 (5.01%)	

### Total sleep duration variability & new stroke

**Fig. 2:**
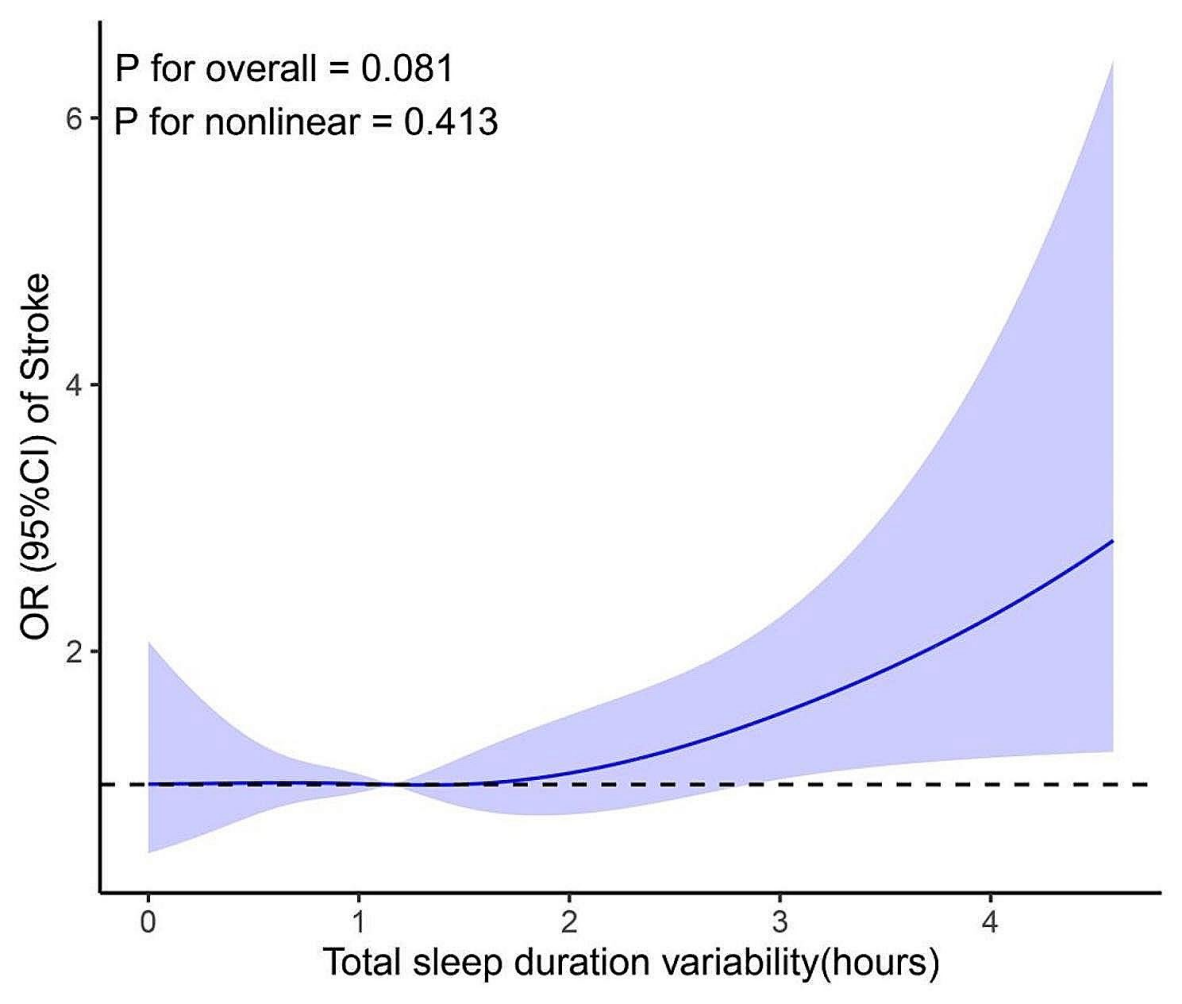
Restricted cubic spline diagram illustrating the association between total sleep duration variability and incidence of new stroke, adjusted for gender, age, education, marital status, residence, smoking and drinking status, BMI, physical activity level, and history of hypertension, dyslipidemia, diabetes, nocturnal sleep duration, and napping duration at baseline. (Tsleep_sd: Total Sleep Duration Variability)

Crude and adjusted logistic regression models were performed, yielding comparable results. After controlling for covariates (as listed in Table 3), a higher variability in total sleep duration was associated with an increased stroke risk. In the restricted cubic spline regression models, a linear correlation between total sleep duration variability and the risk of incident stroke was demonstrated in Fig. 2 (*p* = 0.413). Specifically, subjects with greater variability in total sleep duration exhibited a higher risk of stroke.

Females showed no significant association between total sleep duration variability and stroke risk (Table 4). However, in males, a significant association was noted, with increased total sleep duration variability linked to a higher risk of new stroke (OR = 1.43, 95% CI: 1.12–1.83).

**Fig. 3:**
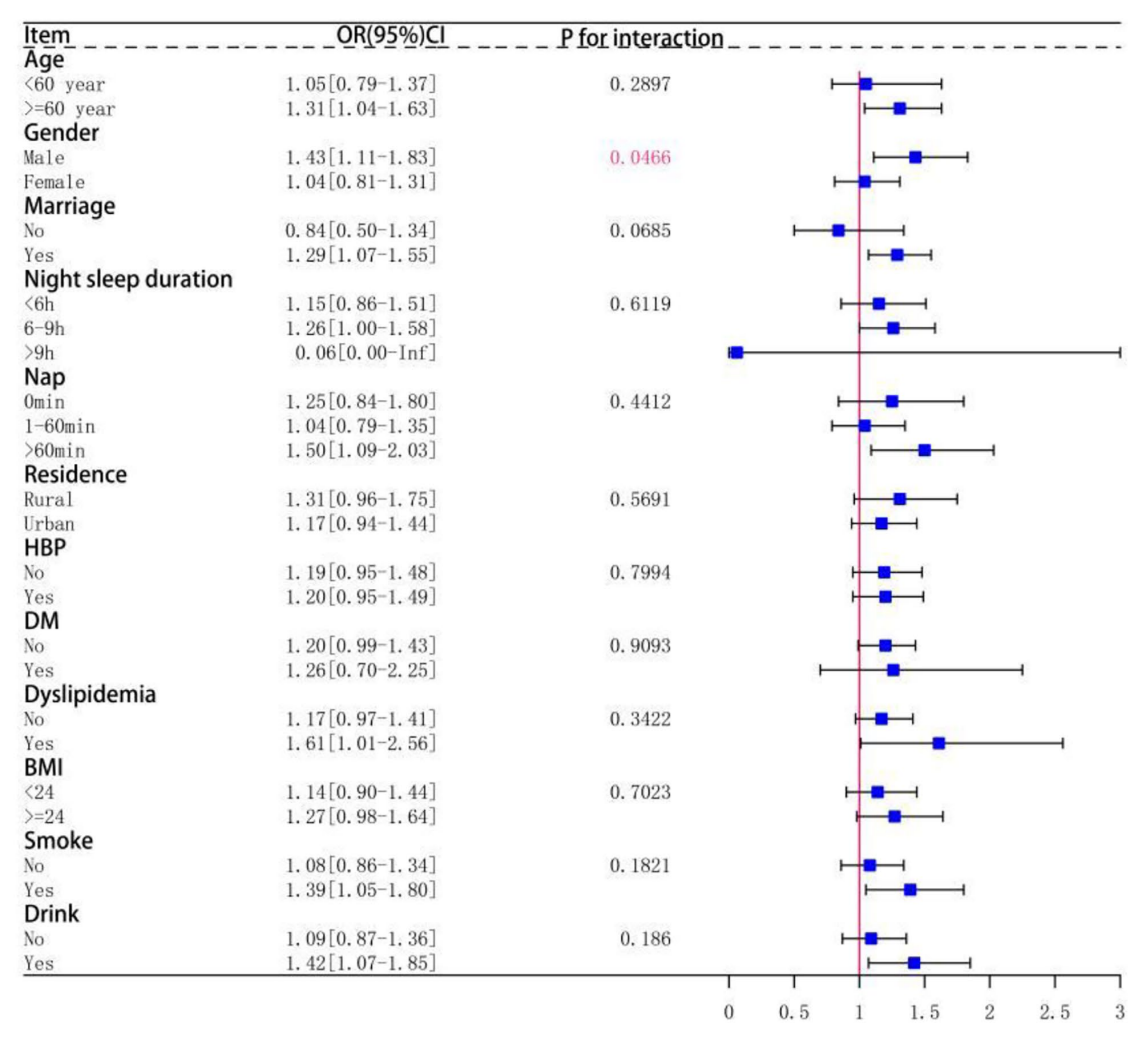
Subgroup analysis of total sleep duration variability and stroke, including interaction analysis of covariates and total sleep duration variability in both sexes

**Fig. 4:**
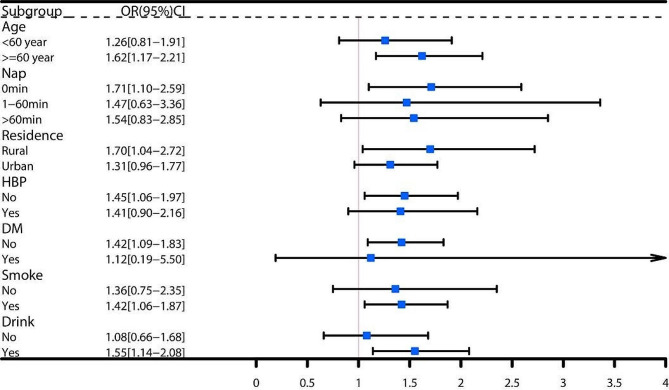
Subgroup analysis of total sleep duration variability and stroke in males

**Fig. 5:**
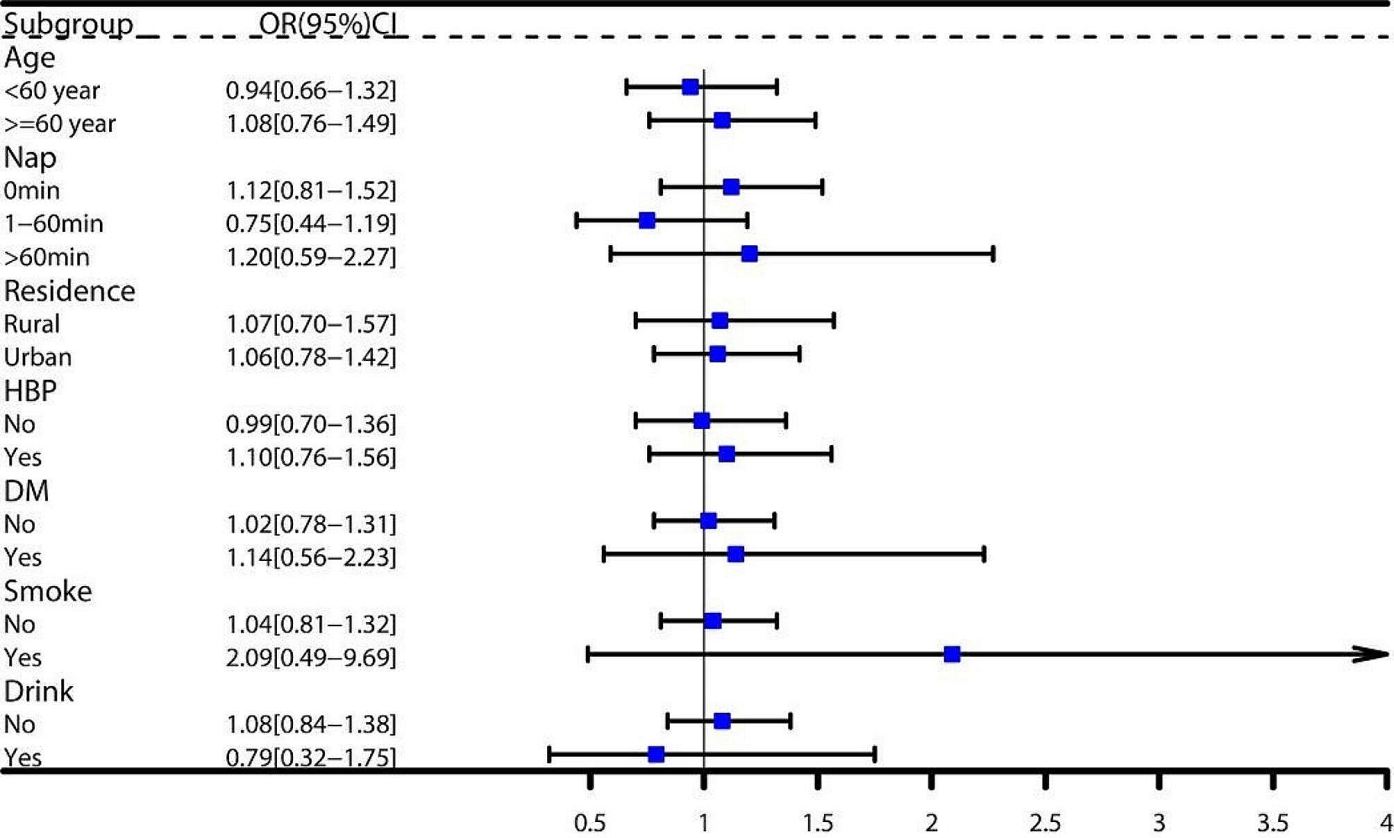
Subgroup analysis of total sleep duration variability and stroke in females

Subgroup analyses indicated a stronger association between increased total sleep duration variability and stroke incidence in individuals who were older (age ≥ 60 years), male, were married, slept for 6–9 h per day, napped for more than 60 min per day, had dyslipidemia, and had a history of smoking or alcohol consumption (see Fig. 3). This association was particularly pronounced in males who were older (age ≥ 60 years), did not engage in daytime napping, had no history of hypertension or diabetes, and had a history of smoking or alcohol consumption (refer to Fig. 4). Nevertheless, no similar association was found in women (see Fig. 5).

**Fig. 6:**
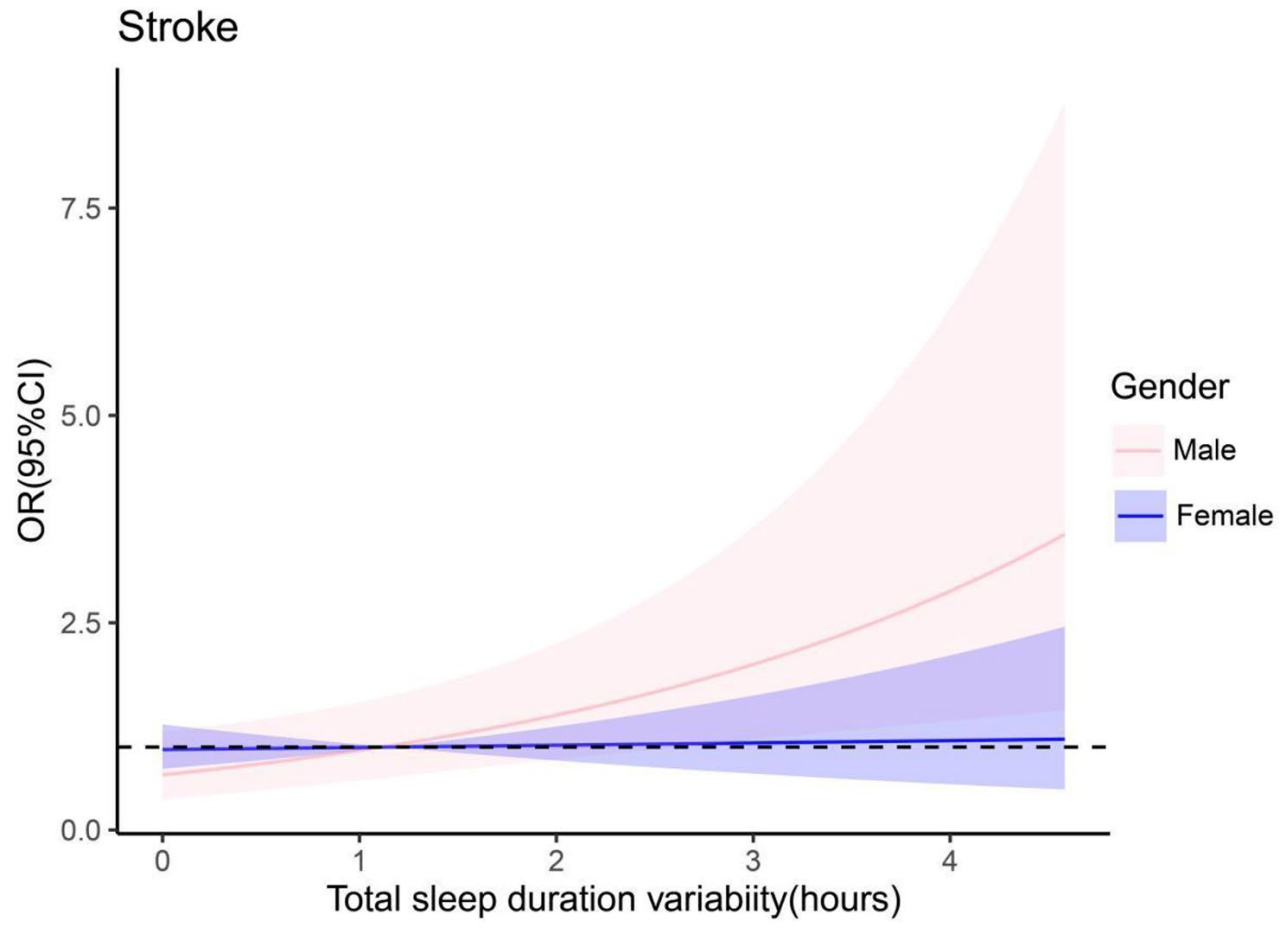
Interaction of total sleep duration variability with gender (Tsleep_sd: Total Sleep Duration Variability)

Furthermore, an interaction was observed between total sleep duration variability and gender. Men demonstrated higher susceptibility to stroke with increased total sleep duration variability compared to women (as shown in Fig. 6).

**Table 3:** Logistic regression analysis for the association between total sleep duration variability and stroke

		Crude	Model1	Model2	Model3
	Case	OR(95%CI)	OR(95%CI)	OR(95%CI)	OR(95%CI)
Tsleep_sd	183(5.25%)	**1.18(1.00-1.39)**	1.18(0.99–1.39)	1.20(1.01–1.42)	**1.20(1.01–1.42)**

Tsleep_sd: Total Sleep Duration Variability;

Model 1, Adjusted for Gender, Age, Education, Marital Status, Residence;

Model 2, Adjusted for Gender, Age, Education, Marital Status, Residence, Smoking Status, Drinking Status, BMI, Physical Activity Level and History of Hypertension, Dyslipidemia, and Diabetes;

Model 3, Adjusted for Factors in Model 2 and Nocturnal Sleep Duration and Napping Duration at Baseline

**Table 4:** Adjusted odds ratio in association between total sleep duration variability & new stroke by subgroup

Variables	case	Crude	Model1	Model2	Model3
		OR(95%CI)	OR(95%CI)	OR(95%CI)	OR(95%CI)
Sex					
Male	86(5.56%)	**1.46(1.14–1.85)**	**1.42(1.10–1.79)**	**1.44(1.12–1.83)**	**1.44(1.12–1.83)**
Female	97(5.01%)	1.01(0.80–1.27)	0.98(0.77–1.22)	1.00(0.79–1.26)	1.03(0.81–1.30)
Age					
< 60 year	89(4.12%)	1.06(0.81–1.36	1.07(0.81–1.38)	1.06(0.80–1.38)	1.05(0.79–1.37)
>=60 year	94(7.08%)	1.23(0.99–1.52)	1.28(1.02–1.58)	1.30(1.03–1.61)	1.31(1.04–1.63)
Diabetes					
Yes	22(12.22%)	1.13(0.67–1.81)	1.17(0.69–1.94)	1.11(0.66–1.80)	1.26(0.70–2.25)
No	161(4.87%)	1.19(0.99–1.41)	1.18(0.98–1.40)	1.20(1.00-1.43)	1.20(0.99–1.43)
Hypertension					
Yes	81(10.40%)	1.14(0.87–1.47)	1.19(0.91–1.55)	1.23(0.93–1.61)	1.24(0.93–1.62)
No	102(3.77%)	1.23(0.99–1.52)	1.20(0.96–1.49)	1.20(0.96–1.49)	1.20(0.95–1.49)
Dyslipidemia					
Yes	31(9.94%)	1.45(0.93–2.18)	1.38(0.88–2.09)	1.44(0.93–2.20)	1.61(1.01–2.56)
No	152(4.79%)	1.16(0.97–1.38)	1.17(0.97–1.40)	1.18(0.97–1.41)	1.17(0.97–1.41)
Night sleep duration					
< 6 h	63(5.21%)	1.15(0.87–1.49)	1.16(0.87–1.51)	1.14(0.86–1.49)	1.15(0.86–1.51)
6–9 h	103(5.26%)	1.23(0.99–1.51)	1.22(0.98–1.51)	1.28(1.02–1.59)	1.26(1.00-1.58)
> 9 h	17(5.38%)	0.70(0.14–1.93)	0.82(0.15–2.90)	0.70(0.14–1.93)	0.06(0.00-Inf)
Nap					
0 min	40(4.58%)	1.31(0.91–1.85)	1.27(0.86–1.82)	1.26(0.85–1.82)	1.25(0.84–1.80)
1–60 min	99(5.18%)	0.99(0.76–1.25)	0.97(0.74–1.24)	1.03(0.78–1.33)	1.04(0.79–1.35)
> 60 min	44(6.28%)	1.40(1.04–1.85)	1.46(1.08–1.95)	1.47(1.07–1.99)	1.50(1.09–2.03)
Residence					
Country	73(6.17%)	1.25(0.94–1.64)	1.26(0.93–1.67)	1.33(0.98–1.77)	1.31(0.96–1.75)
City	110(4.78%)	1.18(0.96–1.44)	1.13(0.91–1.39)	1.17(0.94–1.44)	1.17(0.94–1.44)
BMI					
< 24	96(4.64%)	1.18(0.93–1.47)	1.16(0.91–1.45)	1.16(0.91–1.45)	1.14(0.90–1.44)
≥ 24	87(6.14%)	1.22(0.95–1.54)	1.24(0.97–1.58)	1.26(0.97–1.61)	1.27(0.98–1.64)
Smoke					
Yes	72(5.77%)	1.39(1.07–1.78)	1.37(1.05–1.77)	1.39(1.05–1.80)	1.39(1.05–1.80)
No	111(4.96%)	1.07(0.86–1.32)	1.07(0.85–1.33)	1.08(0.86–1.34)	1.08(0.86–1.34)
Drink					
Yes	70(5.36%)	1.39(1.07–1.78)	1.36(1.04–1.76)	1.40(1.06–1.83)	1.42(1.07–1.85)
No	113(5.19%)	1.09(0.88–1.34)	1.36(1.04–1.75)	1.09(0.87–1.35)	1.09(0.87–1.36)
Marriage					
Yes	156(4.93%)	1.25(1.04–1.48)	1.25(1.04–1.49)	1.29(1.07–1.54)	1.29(1.07–1.55)
No	27(8.26%)	0.80(0.49–1.24)	0.86(0.52–1.35)	0.83(0.50–1.31)	0.84(0.50–1.34)

### Sensitivity analysis

Similar results were obtained from sensitivity analyses, which excluded individuals who had used sedative-hypnotics during the initial three waves, as shown in Table 5. The association between sleep duration variability (assessed by the coefficient of variation of total sleep duration or the standard deviation of nocturnal sleep duration) and the risk of stroke in men persisted, as shown in Table 6.

**Table 5:** Sensitivity analyses 1 of the association between total sleep duration variability and stroke (after excluding individuals who had used sedative-hypnotics during the initial three waves)

Subgroup	case	Adjusted
		OR(95%CI)
Total	181(5.23%)	**1.20(1.01–1.42)**
Male	86(5.58%)	**1.43(1.12–1.83)**
Female	95(4.94%)	1.04(0.82–1.32)

**Table 6:** Sensitivity analyses 2 of the association between total sleep duration variability and stroke

Subgroup	case	tsleep_cv	sleep_sd
		OR(95%CI)	OR(95%CI)
Male	86(5.56%)	**4.65(1.15–16.92)**	**1.35(1.04–1.74)**
Female	97(5.01%)	1.38(0.39–4.32)	1.07(0.83–1.36)

Tsleep_cv: Total Sleep Duration Variability Assessed by the Coefficient of Variation of Total Sleep Duration;

Sleep_sd: Nocturnal Sleep Duration Variability Assessed by the Standard Deviation of Nocturnal Sleep Duration

## Discussion

In this prospective cohort study, we found that among middle-aged and elderly individuals, increasing variability in total sleep duration over 4 years was significantly associated with an increased risk of new stroke. Additionally, a linear, positive correlation between total sleep duration variability and stroke risk was observed. This correlation was especially relevant in individuals over 60 years of age, males, those with a BMI > 28, married individuals, and those with a history of smoking or alcohol use.

More research has focused on the correlation between sleep duration at a single point in time and stroke risk, but research on the impact of prolonged changes in sleep duration on stroke risk is limited [15, 22, 23]. Findings from the Kailuan study, a prospective cohort study in a Chinese adult population, suggested that both stable low and increasing low sleep duration trajectories might be associated with a higher risk of subsequent first cardiovascular events, including atrial fibrillation, myocardial infarction, and stroke [15]. In the Tongji Dongfeng cohort study, individuals who consistently slept for ≥ 9 h per night or increased their sleep duration from 7 to 9 h to ≥ 9 h had a higher risk of stroke compared to those who consistently slept for 7–9 h per night. A previous study of a British population revealed that individuals who consistently slept for extended periods (> 8 h) or had a significant increase in sleep duration (from less than 6 h to > 8 h) were at least twice as likely to experience stroke compared to those who consistently slept for 6–8 h [23]. Previous research focused on the qualitative categorization of long-term variations in sleep duration, assessing the relationship between variously classified long-term sleep patterns and stroke risk. However, these studies did not establish a dose-response relationship between quantitatively measured changes in sleep duration and stroke risk. Therefore, our study employed the calculation of intra-individual standard deviation (SD) from three measurements of total sleep time over four years to provide a quantitative assessment of the long-term changes in total sleep duration for each participant.

To the best of our knowledge, this study is the first to investigate the association between sleep duration variability and stroke incidence. Previous research has primarily focused on the relationship between sleep variability and cardiovascular disease, along with its associated risk factors [24]. Findings indicate a correlation between total sleep duration variability and BMII [24], obesity/overweight [25], glycated hemoglobin26, hypertension [17, 26], cardiovascular disease [27], and metabolic syndrome [28]. Regarding our findings that increased total sleep duration variability might elevate stroke incidence, we hypothesize the following contributing mechanisms. First, inconsistent sleep patterns increase the likelihood of risk factors associated with cardiovascular disease [18], such as dyslipidemia, high blood pressure, and elevated blood glucose levels, potentially leading to stroke onset [29, 30]. Second, irregular sleep patterns may affect melatonin production. Melatonin plays a crucial role as an antioxidant and neuroprotectant within the body [31]. Experimental studies have demonstrated melatonin’s role in preventing the onset and progression of stroke [32, 33]. Third, sleep rhythm disorders may be linked to specific genetic abnormalities. Several circadian genes, such as clock, per2, and bmal1, regulate a broad spectrum of cardiovascular rhythms and processes, including blood pressure, endothelial function, and thrombosis [34–37]. There is a significant correlation between circadian genes and enzymes that regulate reactive oxygen species (ROS) [38]. Experimental studies indicated that the deficiency of the Per2 gene heightened susceptibility to ROS [39], and the downregulation of the Bmal1 gene disrupted redox defenses, leading to increased oxidative damage [40]. Fourth, irregular sleep disrupts the autonomic nervous system’s rhythm, thus affecting cardiovascular function [41, 42]. The autonomic nervous system is responsible for regulating the body’s stress response to a wide range of stressors, including acute and chronic risk factors for stroke, mitigating the effects of these stressors and restoring homeostasis [43, 44]. However, impaired function of the autonomic nervous system can disrupt the stress response and contribute to the onset of stroke [43]. Finally, irregular sleep patterns represent a mismatch between daily lifestyle activities, such as eating and exercising, and the body’s natural circadian rhythm. This can result in metabolic changes that are linked to obesity, ultimately increasing the occurrence of stroke [45–47].

In this study, a significant association between total sleep time variability and stroke was not observed in women. Previous studies have shown that men have a higher lifetime risk of ischemic stroke than women within most age strata [48]. Most of the women enrolled in the study were in either the perimenopausal or postmenopausal stage, characterized by low levels of estrogen. Studies indicate that estrogen hinders stroke progression through its anti-atherosclerotic effects on the vascular system and its modulation of adipogenesis. Additionally, estrogen reduces stroke damage by causing coronary artery vasodilation and providing neuroprotection to brain and glial cells during ischemia [49]. Thus, the lack of estrogen might play a significant role in the occurrence of stroke in women, and the relative importance of total sleep duration variability might be diminished.

This study is significant due to its longitudinal evaluation of changes in sleep duration over a 4-year period, its analysis of the quantitative and qualitative links between total sleep duration variability and stroke incidence, and its status as a prospective cohort study in a middle-aged and elderly population in China, offering more rational and credible findings from a broader societal sample. Furthermore, the study accounted for possible confounding factors and carried out thorough subgroup analyses, interaction analyses, and sensitivity analyses. However, it is important to acknowledge that this study has certain limitations. Firstly, participants’ self-reported information was used to evaluate sleep rather than an objective measure, possibly introducing error. a more objective and.

accurate measurement of sleep duration should be polysomnography (PSG) [50]. Secondly, participants’ self-reported their stroke diagnosis, based on the doctor’s judgment, which could be prone to bias. Thirdly, the study database did not evaluate details on other aspects of sleep, such as snoring, sleep apnea, and sleep quality. we could not obtain relevant data to assess sleep quality in CHARLS, which can be supplemented by follow-up studies. Finally, it should be noted that the findings of this study are relevant only to the Chinese population aged 45 or above and cannot be generalized to other populations.

## Conclusion

In conclusion, this study reveals a dose-response relationship between the variability of total sleep duration and future stroke risk in the middle-aged and elderly Chinese population. This relationship is independent of age, nocturnal sleep duration, napping habits, area of residence, and a history of hypertension, diabetes mellitus, dyslipidemia, Body Mass Index (BMI), smoking status, alcohol consumption, and marital status. Stabilizing and minimizing fluctuations in sleep duration could play a role in preventing future stroke events. Particularly, it is crucial for men to focus on maintaining stable sleep patterns.

### Electronic supplementary material

Below is the link to the electronic supplementary material.

Supplementary Material 1

Supplementary Material 2

Supplementary Material 3

Supplementary Material 4

## Data Availability

The datasets analysed during the current study are available in the [CHARLS] repository, [https://charls.pku.edu.cn/]
