# Supplementary material for: Association of total sleep duration variability with risk of new stroke in the middle-aged and elderly Chinese population
Source: BMC Neurol. 2024 Jun 25;24:217. doi: 10.1186/s12883-024-03727-8 (PMC11197293; doi:10.1186/s12883-024-03727-8)
Supplement: Supplementary file 1 — Supplementary Material 1 [file 12883_2024_3727_MOESM1_ESM.pdf]

CHINA HEALTH AND RETIREMENT LONGITUDINAL STUDY – BASELINE QUESTIONNAIRE

August 2011

CHINA CENTER FOR ECONOMIC RESEARCH
PEKING UNIVERSITY

CONTENTS

COVERSCREEN 2
A HOUSEHOLD ROSTER 7
B 12
C FAMILY 21
C1 PARENT CHILDREARING AND SIBLING INFORMATION 21
CA PARENT INFORMATION 21
CB CHILDREARING INFORMATION 25
CC SIBLINGS 34
C2 TIME TRANSFER AND TRANSFERS 36
CD TIME TRANSFER 36
CE TRANSFERS 37
CF TIME SPENT PROVIDING CARE 45
D HEALTH STATUS AND FUNCTIONING 48
DA HEALTH STATUS 48
DB FUNCTIONAL LIMITATIONS AND HELPERS 62
DC COGNITION & DEPPRESSION 68
DE SELF-REPORTED HEALTH &VIGNETTES 72
E HEALTH CARE AND INSURANCE 78
PART I MEDICAL INSURANCE 78
PART II HEALTH CARE COSTS AND UTILIZATION 80
F WORK, RETIREMENT AND PENSION 91
FA JOB STATUS 91
FB WORK HISTORY 92
FC CURRENT PRIMARY JOB/OCCUPATION 93
FK UNEMPLOYMENT AND JOB SEARCH ACTIVITIES 107
FL MOST RECENT JOB 107
FM RETIREMENT 110
FN PENSION INSURANCE 116
G &H INCOME, EXPENDITRUES AND ASSETS 127
G1 VIGNETTS ON INCOME 127
G2 HOUSEHOLD INCOME AND EXPENDITURES 127
HA HOUSEHOLD ASSETS 135
HB INDIVIDUAL ASSETS 144
I. HOUSING CHARACTERISTICS 151
HOUSEHOLD CONTACTS 154
J. INTERVIEWER OBSERVATION 156
APPENDIX 158

COVERSCREEN

[IWER: Find a respondent (he/she does not have to be 45+ years of age, but could not be nanny, short workers or visitors) from the sample house.]
[（45）.]

CV000. Did the household receive *a letter to the resident?*
（1）Yes
（2）No

CV001. How many households live in this dwelling? _____0..25Households

[PROCEDURE: Ask each household the following questions.]
[:]

CV002_1. Is this the main home for this household? Is there another house located elsewhere in which the family lives most of the time?
（1）Theres another house they live most of the time.
（2）Its the main home. skip to CV002 CV002

CV002_2. Is that house located in this neighbourhood? /
（1）Yes
（2）No **go to next household**

[PROCEDUREIf each household answered no in CV002_2QUIT.]
[CV002_2.]
We appreciate your time. However, there is no eligible household for us to interview this time.
!

[PROCEDUREIf more than one household lives hereask each one CV002 and CV003.]
[CV002 CV003 .]

CV002. How many persons live in this household? ___________ 0..25Persons

CV003. How many persons aged 39 + years that belong to your household? (Those who was born before July 1, 1971 based on the solar calendar or May 9, 1971 based on the lunar calendar,)39（（）197171（）197159)
________0..25Persons

[PROCEDURE: List all households with age-eligible members then randomly choose one, continue CV001_1-CV030. If there is no eligible household, end the cover screen.]
[CV001_1- CV030]

[PROCEDUREIf only one household lives hereask CV001_1 to CV030]
[CV001_1 CV030.]

[IWER: Find a respondent (Call it RB) (he/she does not have to be 45+ years old, but could not be nanny, short workers and visitors etc) from the chosen eligible household, and ask CV001_1 to CV030.]
[（45）CV001_1 CV030]

COVERSCREEN

We appreciate your participation. The China Health and Retirement Longitudinal Study (CHARLS) is interested in learning about important aspects of peoples lives, such as their health, financial, and family situations, and in providing a high-quality public use dataset on aging for academic and policy research. The survey sample is composed of persons aged 45 and older from about 10,000 households throughout the country.
10,00045！

CV001_1. How many dwelling units does your household live in this village/community? /?____________
0..25Houses
[IWER: Not ask the house property, but the number of houses in which they are living.]

CV001_2. How many people lived with this household for more than six months in the past year(including those attending school/work away from home but returning to this home almost every week, but not including non-family members who live here for school or work reasons but generally return to ones own home every week.)? （Not yourself but including all other people living with; nannies should be included. Do not include persons who recently left because they got married to a member outside the household and moved to another household, or persons who recently divorced a member and then moved out.） 66_________ 0..25Persons
[IWER: This type excludes one who lived with this household for more than six months but has gone out of the country, and he/she lived in the resident living district. 6]

[PROCEDURE: If CV001_2 >0, ask CV001_2_1]
[CV001_2 >0, CV001_2_1]

CV001_2_1. What are their names? ___________

CV001_3. In the past year, how many people lived in this household for less than 6 months, but recently joined this household because they were new born or were adopted, or got married to another household member, or they got divorced from someone outside the household and moved back to this household? （Not Including yourself）6? __________0..25Persons

[PROCEDURE: If CV001_3 >0, ask CV001_3_1]
[CV001_3 >0, CV001_3_1]

CV001_3_1. What are their names? ____________

CV001_5. In the past year, how many people lived in the household for less than 6 months because they lived with more than one household during the year, but still spent more time with this household than any other household, for example old persons or someone needs to be taken after by others? （NOT including the persons above）_______0..25Persons

[PROCEDURE: If CV001_5 >0, ask CV001_5_1]
[CV001_5 >0, CV001_5_1]

CV001_5_1. What are their names? __________

CV001_4. In the past year, how many people who went to school/joined army/went to work away from home lived elsewhere in a dormitory or workplace (or live-in nanny lives in others house) but not living in the resident living district? （NOT including the persons above）（） _______ 0..25Persons

[PROCEDURE: If CV001_4 >0, ask CV001_4_1]
[CV001_4 >0, CV001_4_1]

CV001_4_1. What are their names? ___________

CV001_4_2. Other than the people you have already described, how many persons lived in your house hold for more than 6 months in the past year, but have since left this household and are no longer m embers of the household (changzhu renkou)? 6______0..25Persons

[PROCEDURE: If CV001_4_2>0, ask CV001_4_3]
[CV001_4_2 >0, CV001_4_3]

CV001_4_3. What are their names? __________

[PROCUDURE: get the total number of all the Household members according to charls mentioned by RB (CVTotal), and list them.]
[: RB (CVTotal).]

CV020. You have described [**CVAll**] persons who are part of your household. They are as follows [LIST OF ALL PERSONS name IN HH]. [**CVAll**][]
Are there any other household members in your household?
[IWER: probe and judge by yourself according to the definition of CHARLS household member. CHARLS）
[IWER: Confirm that all household members have been listed. If Not all members are listed, please (1) Yes to add new one.
[(1)]

(1) Yes
(2) No

[PROCUDURE: IF CV020 = 1 asked the following question for additional person]
[: CV020 = 1]

CV001_6_1. What is his/her name? /___________

CV010. What is [CV001_6_1]s type? [CV001_6_1]
(1) Lived with this household for more than six months in the past year. 6
(2) New household members. 6
(3) Rotating parents or other household members.
(4) Lived elsewhere in a dormitory or workplace but not in residential area.
(5) Lived in the household for more than 6 months in the past year but have left the household and are no longer members of the household (changzhu renkou). 6

CV025_0. Does the household have any live-in nanny?
(1)Yes, on the list
(2)Yes, not on the list skip to CV030
(3)No skip to CV030

If CV025_0 =1, ask:
CV025. Please check (Mult choice).（）
1-25: the list of household member

CV030. In the following list, who was born before July 1, 1971 based on the solar calendar or May 9, 1971based on the lunar calendar?(Those whore aged 39 and older) （）197171（）197159（39）
1-25: the list of household member exclude the nanny
30: None

Option 1: No eligible household member
CONTINUE TO EXIT
We appreciate your time. There is no age-eligible person for us to interview this time. Thank you for your time!
！

Option 2: Have eligible household member(s)
[Randomly select one age-eligible household member if there is more than one.]
[]

[PROCEDURE: Member in Fourth Category is eligible to be main respondent, but Fifth Category i s not.]
[.]

CV005. [IWER: Record Gender of Main Respondent. .]
(1) Male
(2) Female

MRbirth. When was [MR] born? []
_______1900..2011 Year(MRbirth_year) ______0..12Month(MRbirth_mon) ______0..31Day(MRbirth_day)
[IWER: Mark the year using four digits. :]
MRbirth_type. Is your answer based on the solar or the lunar calendar? （）（）
(1) Solar calendar （）
(2) Lunar calendar （）

[Show Card 1]
CV033. What is [preload MR]s marital status? []
[IWER: common-law marriage is considered as married. .]
(1）Married with spouse present
(2）Married but not living with spouse temporarily for reasons such as work
（3）Separated （）
（4）Divorced
（5）Widowed
(6) Never married

[PROCEDURE: if married, CV033 =1/2, then ask CV040]
[CV033 =1/2CV040]

CV040. Who is [MR]s spouse? []
1-25:the list of household member not including himself
30: not in the listname

CV045. [IWER: confirm the gender of Main Respodents spouse MR.]
(1) Male
(2) Female

Determine the Financial Respondent
CV031. I will ask some questions about household financial status. Who would be most knowledgeable about this? Please tell me who will answer the financial module.
[**Please list all the household members, and pick one member from them.]**

Determine the Family Respondent

[PROCEDURE: If [Married with spouse present or living with a partner as if they were married] ask CV032]
[CV032]

CV032. I will ask some questions about family status. Who would be most knowledgeable about this, [MR] or [MRs spouse]? [][]
(1) [MR] []
(2) [MRs spouse] []

[PROCEDURE: If the Main Respondet is 39 to 44, end this interview and begin contact sheet onl y]
[3944.]
We appreciate your time. You are not our sample now, but you will become our sample in 6 years. Can you tell us your contact information so that we can keep in touch with you?
[IWERPIs start contact sheet.]
[]6[][]/

[PROCEDURE: If the Main Respondet is 45 or older, continue the interview]
[45[(MR_age_year<1966 and MR_age_month<7 and MR_ager=1) or (MR_age_year<1966 and MR_ag e_month<6 and MR_ager=2)].]

[IWER: Coverscreen is completed. Please click <next> to back SMS to continue interview.]
[<>]

A HOUSEHOLD ROSTER

[IWER: Please conduct this part of the interview (A Household roster) when the family respondent is at home. Dont allow a proxy to complete the entire section. H]

A001. [[IWER: Take down the type of this neighbourhood.]
(1) Rural Village
(2) Urban Community

[INTRO: Relatives can have important effects on your life. Wed like to ask you some question s about other members of your household:]

[IWER: The names of other household members, not including MR and spouse, are preloaded from the coverscreen information. Ask each household member the following questions:]

*[1]**A002.** Gender of this household member []
（1）Male
（2）Female

***A003.** When was [name] born? []
_______(A003_1)1900..2011 (HBirthyear)Year ______(A003_2)0..12 (HBirthmonth)Month

[Show Card 1]
***A004.** What is [name]s marital status? []
[Notice Common-law marriage is considered as married.]
(1) Married with spouse present
(2) Married but not living with spouse temporarily for reasons such as work
(3) Separated （）
(4) Divorced
(5) Widowed
(6) Never married

[PROCEDURE: If A004 =3,4,5,6 and >=45years old, please ask A005]
[A004 =3,4,5,645A005]
[Intro: It is common that many people who are not currently married often live with partners. Please bear me a question on this. The answer to this question will be kept strictly confidential and will be used for researchpurposes only.]

A005. Is [name] unmarried but Living with a partner? []
(1) Yes
(2) No

[2]A006.** What is the relationship of [name] to you? []
(1) Mother Go to A009 A009
(2) Father Go to A009 A009
(3) Mother-in-law / Go to A009 A009
(4) Father-in-law / Go to A009 A009
(5) Sibling Go to A009 A009
(6) Brother-in-law, sister-in-law / Go to A009 A009
(7) Child Go to A007 A007
(8) Spouse of child / Go to A009 A009
(9) Grandchild Go to A009 A009
(10) Other relative（specify） （） _______(A006_1) Go to A009 A009

1 *****, Please refer to LOOP questionnaire HMem_Roster.dta. *LOOP
2 ******, Please refer to LOOP questionnaire HHMem1.dta. **LOOPBA

[PROCEDURE: If CV033=1/2, please ask A007] [CV033 =1/2, A007]

****A007.** What is the relationship between [name] and you? []
（1） The biological child of your and your (current) spouse. （）
（2） The biological child of you, but not of your (current) spouse.
（3） The biological child of your (current) spouse, but not of yours. （）
（4） The adopted or foster child of you or your spouse. （）

[PROCEDURE: If CV033=3/4/5/6please ask A008] [CV033 =3/4/5/6A008]

****A008.** What is the relationship between [name] and you? []
（1） Your biological child.
（2） Your adopted or foster child or step child.

****A009.** What is the current hukou status of [name]? []
(1) Agriculture Hukou Skip to A012 .A012
(2) Non-Agriculture Hukou Skip to A012 .A012
(3) Unified Residency Hukou
[IWER: for the place where agricultural hukou is abandoned.]
(4) Do not have Hukou Skip to A013 .A013
F1

****A010.** What is [name]s Hukou status before he/she has the unified residency hukou? []
(1) Agriculture Hukou
(2) Non-Agriculture Hukou
(3) Do not have Hukou
F1

****A011.** When did [name] have the unified residence Hukou? []
________ 2000..2011year
F1

****A012.** What is the location of [name]s current hukou? []
(1) This household
(2) This village/neighborhood /
(3) Another village/neighborhood in this county/city //
(4) Another county/city in this province/___________(A012_1)city__________ (A012_2) county
(5)Another province _______(A012_3)province //, _____ _______(A012_4)city_____ ______(A012_5) county[IWER: Choose from the list of provincessee appendix 2]
(6) Abroad

[PROCEDURE: If A006=7, ask A021. A006=7()A021]

****A021.** Where was the birth place of CHILD's NAME? []
(1) This village/neighborhood /
(2) Another village/neighborhood in this county/city //
(3) Another county/city in this province/___________(A021_1)city__________ (A021_2) county
(4)Another province _______(A021_3)province //, _____ _______(A021_4)city_____ ______(A021_5) county[IWER: Choose from the list of provincessee appendix 2]
(5) Abroad

****A022.** Is [name]s present hukou status and location the same as his/her first Hukou? [

(1) Yes →Skip to precedure before A013 A013
(2) No

****A023.** How did [name]s Hukou status or location change?
[]
(1)Both Hukou status and location have changed
(2)Only Hukou status has changed
(3)Only Hukou location has changed →Skip to A025 A025

****A024.** What was [name]s first HuKou status? []
(1) Agricultural Hukou
(2) Non-agricultual Hukou

[If A023=2, skip A025. A023=2A025]

****A025.** What was the location of [name]s first hukou? []
(1) This village/neighborhood /
(2) Another village/neighborhood in this county/city //
(3) Another county/city in this province/___________(A025_1)city__________ (A025_2) county
(4)Another province _______(A025_3)province //, _____ _______(A025_4)city_____ ______(A025_5) county[IWER: Choose from the list of provincessee appendix 2]
(5) Abroad

[PROCEDURE: If the person is less than 6 years old, skip to the next person 6]

A013. Is [name] still in school now? []
(3) Yes
(4) No →Skip to A0015 A015
[IWER: Fulltime student, not on-the-job student.]

A014. What level of schooling and grade is [name] currently enrolled in? []
(1) Primary school grade 1 1
(2) Primary school grade 2 2
(3) Primary school grade 3 3
(4) Primary school grade 4 4
(5) Primary school grade 5 5
(6) Primary school grade 6 6
(7) Middle school grade 1 1
(8) Middle school grade 2 2
(9) Middle school grade 3 3
(10) Middle school grade 4 4
(11) High school, grade 1 1
(12) High school, grade 2 2
(13) High school, grade 3 3
(14) Vocational/technical high school year 1 1
(15) Vocational/technical high school year 2 2
(16) Vocational/technical high school year 3 3
(17) College year 1 /1
(18) College year 2 /2
(19) College year 3 /3
(20) College year 4 /4
(21) College year 5 /5
(22) College year 6 /66
(23) Masters degree
(24) Doctoral degree/ Ph.D. degree

[PROCEDURE:Skip to procedures befor A016A016]

[PROCEDURE: If the person is less than 12 years old, skip to the next person 12]

A015. What is the highest level of education completed? []

(1) No formal education (illiterate) （）
(2) Did not finish primary school but capable of reading or writing
(3) Sishu/home school
(4) Graduate from elementary school
(5) Graduate from middle school
(6) Graduate from high school
(7) Graduate from vocational school （）
(8) Graduate from Two/Three Year College / Associate degree
(9) Graduate from Four Year College / Bachelors degree
(10) Graduate from Post-graduate, Masters degree
(11) Graduate from Post-graduate, Doctoral degree/Ph.D.

[PROCEDURE: If the person is less than 16 years old, skip to the next person 16]

A016. Did [name] spend one or more months away from the household in the past year? []

(1) Yes
(2) No Skip A017, A018, A019 A017, A018, A019

A017. How many months in the past year did [name] live away from home?
[]____ 0..12months

[Softcheck if 0 or missing is reported in A017 and yes is checked in A016 . A017 0A016 =1]

A018. Where is the main place that [name] lived during his/her time away? []

(1) This county/city/
(2) Another county/city in this province/___（A018_1）city____（A018_2） county
(3)Another province ________(A018_3)province //___________(A018_4)city___________(A018_5) county
[IWER: Choose from the list of provincessee appendix 2.]
(4) Abroad

A019. What type of location did [name] live in? []?

(1) City
(2) County
(3) Town
(4) Village

[PROCEDURE: Go/Proceed to the next person]

A020. How often did the respondent receive assistance in answering section A-HOUSEHOLD ROSTER? A
[[IWER: If it is answered by a proxy, please record the respondents reaction.]

(1) Never
(2) A few times
(3) Most or all of the time

NOTE: ONLY THOSE WHO WERE BORN BEFORE JULY 1, 1966, AND THEIR SPOUSES WILL BE INTERVIEWED. 196671

[Show Card 2]

BA001. What is your Chinese Zodiac sign? ________________
[IWER: Choose from the list of Chinese Zodiac signssee Appendix 1]

BA002. When were you born?
_______ 1900..2011 (BA002_1)year ______0..12 (BA002_2) month______0..31 (BA002_3)day
[IWERThe year must be a number in the range [1900 – 2011]. Mark the year using four digits. Take down the month as its actual number. For example, write January as 1 not 01, December as 12. If do not remember month and day, fill 0. [1900－2011]41101,12120]

[CAPI: Check date of birth by Zodiac.]
[If the person does not know the date of birth, BA002 =DK or BA002 =RF, skip BA003. BA002 BA003 .]
BA003. Is your answer to BA002 based on the solar or the lunar calendar? （） （）

(1) Solar calendar （）
(2) Lunar calendar （）

[If the person does not know the date of birth, BA002 =DK or BA002 =RF, ask BA004 BA002 BA004 .]
BA004. What is your age? ________1...120years old
[IWER: You can refer to the year for some major events or born in which year during the republic of China. (begin in 1912) (1912)]
F11912+1911=

BA005. Do you attend school/work away from home but return to this home almost every week? /

(1) Yes
(2) No

[INTRO: Next are some questions about your birth place, some changes in your housing locatio n, your Hukou and education.]
BB001. Where were you born?

(1) This village/neighborhood / Skip to BB006 BB006
(2) Another village/neighborhood in this county/city // Skip to BB005 BB005
(3) Another county/city in this province/_____(BB001_1)city_____(BB001_2) county
(4) Another province _____(BB001_3)province //_______(BB001_4)city________(BB001_5)county
[IWER: Choose from the list of provincessee Appendix 2]
(5) Abroad

BB002. What is the type of your birth place? Is it rural village or urban community?

(1) Rural Village
(2) Urban Community

BB003. When did you first live in this county/city? /
_________ 1900..2011 Year
[IWER: Mark the year using four digits. 4]

BB004. When you first moved to this county/city, did you live in the same village/community as you currently do? //

(1) Yes skip BB005 BB005
(2) No

BB005. In what year did you first live in this village/neighborhood? /___________ 1900..2011Year
[IWER: Mark the year using four digits. 4]

BB006. Where did you mainly live before you were 16 years old? Is it in village or city/town? 16
[IWER: City or Village when the person was living there.]
(1) Village
(2) City/Town

[PROCEDURE: If BB001 =1 or BB001 =2 go to BB009 ; else go to BB007] [BB001 =1 or BB001 =2BB009 BB007]

BB007. Ever since you first came to this county/city, have you ever lived outside your present county/city for more than 6 months? //
(1) Yes
(2) No → Go to BC001 BC001

BB008. Ever since you first came to this county/city, how long have you lived outside this county/city in total? // ________(BB008_1)years ________(BB008_2)months
Skip to BB011 BB011

BB009. Have you ever lived outside this county/city for more than 6 months? /
(1) Yes
(2) No →Skip to BC001 BC001

BB010. How long have you lived outside this county/city in total? /
________(BB010_1)years ________(BB010_2)months [hard check: years <= 2011-BB003 <= BB003]

BB011. When did you most recently live in this county/city (after being away for 6 months or longer)? /（）______ 1900..2011year [IWER: Mark the year using four digits. 4]
[hard check: year >=BB003 >= 2011-BB003]

[Show Card 3]
BB012. Before moving to the current county/city, where else did you live for at least six months? (Not less than six months, otherwise ask about previous locations where the respondent lived for at least six months) /（）
(1) Birth place
(2) Another county/city in this province/______(BB012_1)city______(BB012_2) county
(3) Another province ______(BB012_3)province //___________(BB012_4city)___________(BB012_5)county
[IWER: Choose from the list of provincessee appendix 2]
(4) Abroad

BC001 . What is your current HuKou status?
(1) Agricultual Hukou Skip to BC004 BC004
(2) Non-agricultural Hukou Skip to BC004 BC004
(3) Unified Residence Hukou
[IWER: for the place where agricultural hukou is abandoned.]
(4) Do not have Hukou Skip to BC009 BC009

F1

[If BC001 =3, ask BC002 to BC003. BC002 BC003]

BC002. What is your Hukou status before you have the unified residence Hukou?

(1) Agricultural Hukou
(2) Non-agricultual Hukou
(3) Do not have Hukou

F1

BC003. When did you have the unified residence Hukou?
________(BC003_1)1900..2011year ________(BC003_2)0..12 month
F1

Go to BC005 BC005

BC004. When did you get your current Hukou?
_____1900..2011(BC004_1)year ________0..12 (BC004_1)month [hard check: enforce year>=BA002_1 ≥BA002_1 ()]
[IWER: You must fill year[1900 - 2011]. Mark the year using four digits. Take down the month as its actual number. For example, write January as 1 not 01, December as 12. If do not remember month, fill 0. : [1900 - 2011]41101,12120]

BC005. Where is your current HuKou?

[IWER: If birthplace is in this village/neighborhood, pls choose the second choice.]

(1) Same as birthplace
(2) This village/neighborhood /
(3) Another village/neighborhood in this county/city //
(4) Another county/city in this province/_______(BC005_1)city______(BC005_2) county
(5) Another province ________(BC005_3)province //___________(BC005_4)city___________(BC005_5)county

[IWER: Choose from the list of provincessee appendix 2]

[ProcedureIf (BC005 =1 and BB001 =3. 4.5) or BC005 =4 or BC005 =5please ask BC006 . (BC005 =1 BB001 =3.4.5) BC005 =4BC005 =5BC006]
BC006. How long have you been away from your current Hukou county/city?
/ ______（BC006_1）Years_________（BC006_2）Months
[hard check: impose that years<=2011-BA002_1 (birthyear) ≤2011-BA002_1 ()]

BC007. Is your first Hukou same as your current Hukou?（Not including the change of unified Residence Hukou.） （）

(1) Yes Go to BD001 BD001
(2) No

BC008. Were there any other Hukou between your first Hukou and your current Hukou?(Including changes of hukou type and place. Hukou type change only means changes between Agricultural Hukou and non-agricultural hukou.) （）

(1) Yes
(2) No

BC009. Has your Hukou type or place ever changed since your first Hukou? (Hukou type change only means changes between Agricultural Hukou and non-agricultural hukou.) （）

(1)Both Hukou type and place have changed
(2)Only Hukou type has changed
(3)Only Hukou place has changed

[If BC009 =1or 2, ask BC010, if BC009 =3. skip BC010. BC009 =12, BC010 .BC009 =3BC010]

BC010. What was your first HuKou status?

(1) Agricultural Hukou
(2) Non-agricultual Hukou

[If BC008 =1 and (BC009 =1 or 3), ask BC011 ,else skip BC011. BC008 =1BC009 =13BC011 , BC011]

BC011. Where was your last HuKou?

[IWER: If last Hukou is same as birthplace and is in another village/neighborhood in this county/city, then choose 1. /1]

(1) Same as birthplace
(2) Another village/neighborhood in this county/city //
(3) Another county/city in this province/____(BC011_1)city____(BC011_2) county
(4)Another province_________(BC011_3)province //___(BC011_4)city________(BC011_6) county
[IWER: Choose from the list of provincessee appendix 2]

[If BC011 =2 or 3 or 4, ask BC012 , else go to the procedure before BC013. BC011 =2 3 4BC012BC013]

BC012. Why the location of current Hukou is different from your first Hukou?

(1) marriage
(2) go to school
(3) employment
(4) retirement/ revolutionary retirement /
(5) escape of famine
(6) sent down to the countryside to do manual labor
(7) migration of the whole village
(8) others, pls specify____ ____（BC012_1）

[If BC009 =1, ask BC013, then go to procedure before BC015; If BC009 =3, ask BC013 and BC014. If BC009 =2, go to procedure before BC015. BC009 =1, BC013BC015BC009 =3, BC013 BC014 . BC009 =2, BC015]

BC013. What was the location of your first HuKou?

[IWER: If first Hukou is same as birthplace or current Hukou, and is same as other choices, then choose 1 or 2. //12]

(1) Same as birthplace
(2) Same as current Hukou
(3) This village/neighborhood /
(4) Another village/neighborhood in this county/city //
(5) Another county/city in this province/___________(BC013_1)city___________(BC013_2) county
(6) Another province _______(BC013_3)province //_________ (BC013_4)city________(BC013_5) county
[IWER: Choose from the list of provincessee appendix 2]

[If (BC009 =1 or 3 and BC013 =3 or 4 or 5 or 6) or (BC009 =1 or 3 and BC013 =1 and BC005 ≠1), ask BC014 , else go to the procedure before BC015. (BC009 =13BC013 =3456) (BC009 =13 BC013 =1BC005 ≠1)BC014, BC015]

BC014. Why the location of current Hukou is different from your first Hukou?

(1) marriage
(2) go to school
(3) employment
(4) retirement/ revolutionary retirement /
(5) sent down to the countryside to do manual labor
(6) migration
(7) migration of the whole village
(8) escape of famine
(9) others, pls specify____ ____(BC014_1)

[If BC001 =1 and BC009 =1 or 2, ask BC015 to BC016 , then skip to BD001 .BC015 BC016 BD001 .]

BC015. What is the reason that you had the non-agricultural Hukou?
(1) sent down during 1967-1977 and did not come back to city 1967-1977
(2) was a student
(3) marriage
(4) employment
(5) migration of the whole village
(6) escape of famine
(7) others,pls specify ______ ,______(BC015_1)

BC016. When did you have the non-agricultural Hukou?
From________(BC016_1)1900..2011year to ________(BC016_2)1900..2011year

[If BC001=2 and BC009=1 or 2, ask BC017 to BC018, BC017 BC018.]

BC017. What is the reason that you had the agricultural Hukou?
(1) sent down during 1967-1977 and then come back to city 1967-1977
(2) live in rural areas before go to college student
(3) marriage
(4) employment
(5) Land is acquired by the government
(6) migration of the whole village
(7) escape of famine
(8) others ,pls specify ______ ,______(BC017_1)

BC018. When did you have the agricultural Hukou?
From________(BC018_1)1900..2011year to ________(BC018_2)1900..2011year

BD001. What is the highest level of education you have attained?
(1) No formal education (illiterate) （） → Skip to BD007 BD007
(2) Did not finish primary school but capable of reading and/or writing
(3) Sishu/home school
(4) Elementary school
(5) Middle school
(6) High school
(7) Vocational school （）
(8) Two-/Three-Year College/Associate degree
(9) Four-Year College/Bachelors degree
(10) Masters degree
(11) Doctoral degree/Ph.D.

[Procedure: If BD001 =2, ask BD002. BD001 =2BD002]
BD002. What is the highest grade did you finish in primary school ____ 1..6
Skip to BD005 BD005

[Procedure: If BD001 >=4, ask BD003. BD001 >=4BD003 .]
BD003. How many additional years of schooling did you complete after [THE ANSWER CHOSEN IN BD001]?
[BD001]______ years

[PROCEDURE: If BD001 >7, ask BD004] [BD001 >7BD004]

BD004. When did you go to college? /?_______ 1900..2011year

[IWERif there are skipped years, fill in the years needed for the grade rather than the actual number of years; if there are repeated years, fill in the years needed for the grade rather than the actual number of years.]

BD005. At what age did you begin formal schooling? /
_____1…120years old [soft check, <6, >11]

BD006. At what age did you finish schooling? _____1…120years old
[IWERIt asks age when R finishes schooling, not age when R finished elementary school./]

BD007. Have you ever attended school for adult education? （）? （）
(1) None Go to BE001 BE001
(2) TV University
(3) Night School
(4) Zikao (examinations for self-taught students)
(5) Hanshou/Correspondence course/Distance learning
(6) Literacy course
(7) Accelerated education course
(8) Other (explain:)__________ (BD007_1)

BD008. How many years did you spend in adult education?
_______ year [soft check >9]

BD009. Did you get a diploma or degree from the adult education program you attended?
(1) Yes
(2) No Go to BE001 BE001

BD010. When did you receive the diploma? ________1900..2011year

BD011. What is the highest level of schooling you obtained from the adult education program?
(1) Vocational school
(2) Two/Three Year College / Associate degree
(3) Four Year College / Bachelors degree
(4) Others

[Show Card 1]
BE001. RMaritalStatus: What is your marital status?
[IWER: common-law marriage is considered as married]
(1) Married with spouse present
(2) Married but not living with spouse temporarily for reasons such as work
(3) Separated （）
(4) Divorced
(5) Widowed
(6) Never married

[PROCEDURE: If BE001 =3/4/5/6, please ask BE002]
[BE001 =3/4/5/6, BE002]
[Intro: It is common that many people who are not currently married often live with partners. Please bear me a question on this. The answer to this question will be kept strictly confidential and will be used for researchpurposes only.]

BE002. Are you unmarried but Living with a partner?
(1) Yes
(2) No

[PROCEDURE: If the person has never married (BE001 =6(never married), then skip to BF008] [(BE001 =6)BF008]
[INTRO: Many people have more than one marrige through whole life. Please bear me a few mor e questions on this.]

BE003. How many times have you been married? _______ times [soft check >2]

[PROCEDURE:If the person married only once, continue with BE004-BE005 , then go to the procedure before BF001] [BE004-BE005BF001]
[If the person married more than once, continue with BE006 and BE009, BE006 BE009]

BE004.When did you get married?
_____1900..2011 (BE004_1) year ________ 0..12 (BE004_2)month [soft check: year>BA002_1 +16 >BA002_1 +16]
[IWER: You must fill year [1900 - 2011]. Mark the year using four digits. Take down the month as its actual number. For example, write January as 1 not 01, December as 12. If do not remember month, fill 0. : [1900 - 2011]41101,12120]

BE005. When you were married, what was the total value of cash and goods (including housing) that your parents gave to you and your spouse? （） ___________Yuan

Skip to procedure before BF001 BF001

BE006. When did you marry the first time?
_____ 1900..2011 (BE006_1) year _______________0..12(BE006_2) month[soft check: year>BA002_1 +16>BA002_1 +16]
[IWER: You must fill year [1900 - 2011]. Mark the year using four digits. Take down the month as its actual number. For example, write January as 1 not 01, December as 12. If do not remember month, fill 0. : [1900 - 2011]41101,12120]

BE007. In what year did your first marriage end? ___________1900..2011Year [hard check: enforce >=BE006 >=BE006]

BE008. Why did your first marriage end?
(1) death of spouse
(2) divorce

BE009 . When was your most recent marriage?
_____1900..2011(BE009_1) year ________ 0..12 (BE009_2)month
[hard check: enforce year >=BE007 >=BE007]
[IWER: You must fill year[1900 - 2011]. Mark the year using four digits. Take down the month as its actual number. For example, write January as 1 not 01, December as 12. If do not remember month, fill 0. : [1900 - 2011]41101,12120]

[PROCEDURE: Based on the answer to BE001, If the person is married with spouse present, skip to BF008.] [BE001](BE001=1 / 2)BF008]
[If the person is married but not living with spouse for separated, divorced, or widowed, then ask them

to answer BF001 to BF007].[(BE001=3/4/5)BF001 BF007]

[IWER: Id like to ask you a few questions about your current (or most recent) spouse/partner:
]

[INTRO: An important part of this study is understanding how people make decisions during diff erent stages of life, we also need to a general age range for your spouse.]

[Show Card 2]

BF001. What is your spouse/partners Chinese Zodiac sign? ______
[IWER: Choose from the list of Chinese Zodiac signssee Appendix 1]

BF002. When was your spouse/partner born?
_______1900..2011 (BF002_1)year ______0..12 (BF002_2)month [preload spouses birthdate]
[IWER: Mark the year using four digits. Take down the month as its actual number. For example, write January as 1 not 01, December as 12. If do not remember month, fill 0. : 41101,12120]

BF003. Is your spouse/partners date of birth based on the solar calendar or the lunar calendar? （） （）
(1) Solar calendar （）
(2) Lunar calendar （）

BF004. What is the highest level of education your spouse/partner has attained? [Note: Please refer to my corrections/suggestions in the previous section with similar questions]
(1) No formal education (illiterate) （）
(2) Did not finish primary school but capable of reading or writing
(3) Home School
(4) Elementary school
(5) Middle school
(6) High school
(7) Vocational school （）
(8) Two/Three Year College / Associate degree
(9) Four Year College / Bachelors degree
(10) Post-graduate, Masters degree
(11) Post-graduate, Doctoral degree/Ph.D.

[If the person is separated, ask BF005 BF005]
[If the person is divorced, ask BF006 BF006]
[If the person is widowed, ask BF007 BF007]

BF005. When did you separate? ______1900..2011 (BF005_1)year ______0..12 (BF005_2)month [hard check: enforce year>year married (BE004 if _BE003 =1. BE009 if BE003 >1)]
[IWER: Mark the year using four digits. Take down the month as its actual number. For example, write January as 1 not 01, December as 12. If do not remember month, fill 0. : 41101,12120]

Skip toBF008 BF008

BF006. When did you divorce? ______1900..2011 (BF006_1)year ______0..12 (BF006_2)month [hard check: enforce year>year married (BE004 if _BE003=1. BE009 if BE003 >1)]
[IWER: Mark the year using four digits. Take down the month as its actual number. For example, write January as 1 not 01, December as 12. If do not remember month, fill 0. : 41101,12120]

Skip toBF008 BF008

BF007. When did your spouse pass away? ?

_______1900..2011 (BF007_1)year ______0..12 (BF007_2)month

[Hard check: enforce year>year married (BE004 if BE003 =1. BE009 if BE003 >1)]

[IWER: Mark the year using four digits. Take down the month as its actual number. For example, write January as 1 not 01, December as 12. If do not remember month, fill 0. : 41101,12120]

BF008. How often did the respondent receive assistance in answering section A-Demographics?

[IWER: If it is answered by a proxy, please record the respondents reaction.]

(1) Never
(2) A few times
(3) Most or all of the time
(4) The section was completed by a proxy respondent（the respondent is absent） →Skip to BF009 BF009

BF009. [IWER: What is the proxys relationship to R? If unknown, please ask the proxy.]

What is your relationship to R?

(1) Spouse
(2) Mother
(3) Father
(4) Mother-in-law /
(5) Father-in-law /
(6) Sibling
(7) Brother-in-law, sister-in-law /
(8) Child
(9) Spouse of child
(10) Grandchild
(11) Other relative
(12) Helper or other non-relative

BF010. [IWER: Please record the reason for proxy]

What is the main reason for proxy (the respondent is absent)

（1）The respondent has serious physical handicaps _
（2）The respondent has serious mental handicaps
（3）The respondent has rejected this interview.
（4）Other____(BF010_1)

C FAMILY

[IWER: Please conduct this part of the interview (B Family) when the family respondent is at home. Dont allow a proxy to complete the entire section. B]

C1 PARENT CHILDREARING AND SIBLING INFORMATION

In the following three parts: bb parent information, bc childrering information and bd sibling information, Id like to ask you some questions about your family.

CA PARENT INFORMATION

First, Id like to ask you some questions about your parents.

[PROCEDURE: If respondents father is a household member, skip to CA003. CA003]
CA001. Is your father still living?
(1) Yes
(2) No →Skip to CA003 CA003

[IWER: If the respondent reports more than one father, ask about the father who raised the respondent.]

CA002. What is his name? __________
[IWER: Mark 'father' if respondent doesn't want to give the name.]

*****[3]CA003.** Is your father your…….?
(1) Biological father
(2) Adoptive father
(3) Stepfather
(4) A different biological relative who raised you
(5) Another individual who raised you

[PROCEDURE: If CA001=1 or is a household member (means living, but was not asked/interviewed CA001), then ask CA004, CA005 ; otherwise, skip them.][CA001 =1CA004 , CA005]
*****CA004.** Where was your father born?
(1) This village/neighborhood /
(2) Another village/neighborhood in this county/city //
(3) Another county/city in this province/_______(CA004_1)city________(CA004_2) county
(4)Another province __(CA004_3)province /___ (CA004_4)city____(CA004_5)county
[IWER: Choose from the list of provincessee appendix 2]
(5) Abroad

*****CA005.**Did your father grow up in an urban area or a rural area?
(1) City
(2) Village

[PROCEDURE: If respondents father is a household member, skip to CA012 . CA012]
[PROCEDURE: If CA001 =1 ask CA006 ; otherwise, skip it] [CA001 =1CA006]
[Show Card 2]
*****CA006.** What is your fathers Chinese Zodiac sign? __________

[3] *******, Please refer to LOOP questionnaire Parents1.dta. ***LOOPBA

[IWER: Choose from the list of Chinese Zodiac signssee Appendix 1:]

***CA007.** In what year was your father born? _______ 1850..1950Year
[IWER: Mark the year using four digits. : 4]
[IWER: Fill in -9999 if the respondent cannot recall the date. :-9999]

[PROCEDURE: If CA001 =2, ask CA008 ; otherwise, skip them] [: CA001 =2CA008 ;]

***CA008.** In what year did your father pass away? ________1900..2011 (CA008_1)Year or______1…120 (CA008_2)Years old
[IWER: Mark the year using four digits. : 4]
[IWER: Fill in -9999 if the respondent cannot recall the date. :-9999]

[Softcheck if the number entered for the year of death is smaller than the number entered for the year of birth. CA008 CA007]

***CA009.** What is the highest level of education your father has completed?
(1) No formal education (illiterate) （）
(2) Did not finish primary school but capable of reading or writing
(3) Sishu/home school
(4) Elementary school
(5) Middle school
(6) High school
(7) Vocational school （）
(8) Two-/Three-Year College / Associate degree
(9) Four-Year College / Bachelors degree
(10) Post-graduate, Masters degree
(11) Post-graduate, Doctoral degree/Ph.D.

[PROCEDURE: If CA001 =1 ask CA010 ; otherwise, skip it] [CA001 =1CA010]
*****CA010. What is your fathers current marital status? [Notice Common-law marriage is considered as married.]**
(1) Married with my mother
(2) Marriedbut not with my mother
(3) Separated （）
(4) Divorced
(5) Widowed
(6) Never married

[PROCEDURE: If CA010 = 3, 4, 5, 6 and >=45years old, please ask CA011]
[CA010 =3,4,5,645CA011]
[Intro: It is common that many people who are not currently married often live with partners. Please bear me a question on this. The answer to this question will be kept strictly confidential and will be used for researchpurposes only.]
CA011. Is the householdmember unmarried but Living with a partner?
(1) Yes
(2) No

[PROCEDURE: If CA001 =2, skip to procedure before CB001] [: CA001=2, procedure before CB001]

***CA012.** Does your father work currently (work includes agricultural work, earning wage work, self-em ployed activities, and unpaid family business work, et. al.)? ()
(1) Yes

(2) No

***CA013.** How is your fathers health? Very good, good, fair, poor or very poor?
(1) Very good
(2) Good
(3) Fair
(4) Poor
(5) Very poor

***CA014.** Which is/was the highest occupation of your father?
(1) Managers
(2) Professionals and technicians
(3) Clerks
(4) Commercial and service workers
(5) Agricultural, forestry, husbandry and fishery producers
(6) Production and transportation workers
(7)Cant be specified

[PROCEDURE: If CA012 =2, skip CA015] [: CA012 =2, CA015]

***CA015 .**What is your fathers average income at present? （）
____yuan / year or ____yuan/month ____(CA015_1)/___(CA015_2)/

[PROCEDURE: If respondents father is a household member, skip to procedure before CB001. CB001]

[Show Card 4]
***CA016.** Where does your father normally live?
(1) The same or an adjacent dwelling/courtyard with me （）（）
(2) Another household in this village/neighborhood /
(3) Another village/neighborhood in this county/city, how far away ____km //: ______(CA016_1)
(4) Another county/city in this province/_____(CA016_2)city______(CA016_3) county, ____________(CA016_4)
(5) Another province _____(CA016_5)province //__________(CA016_6)city_________(CA016_7)county, ___________(CA016_8)
[IWER: Choose from the list of provincessee appendix 2]
(6) Abroad

[PROCEDURE: If CA016 =3-5, ask CA01 . CA016 =3-5CA017]

***CA017.** What kind of location does your father live in?
(1) City
(2) County
(3) Town
(4) Village

***CA018.** Is his hukou in the same place as his current residence?
(1) Yes Skip to CA020 CA020
(2) No
(3) Does not have Hukou Skip to CA021 CA021

***CA019.** What is the location of your fathers current hukou?
(1) This village/neighborhood /

(2) Another village/neighborhood in this county/city //
(3) Another county/city in this province/_________(CA019_1)city_______(CA019_2) county
(4) Another province _______(CA019_3)province //________(CA019_4)city__________(CA019_5) county
(5) Abroad
[IWER: This village/neighborhood, this county/city and this province each means the respondents one:////]

***CA020.** What is your fathers current hukou status?
(1) Agriculture Hukou
(2) Non-Agriculture Hukou
(3) Unified Residency Hukou
（4）Do not have Hukou
F1

***CA021.** Does your father own a house?
(1) Yes
(2) No skip CA022 and CA022_a CA022CA022_a

***CA022.** Do you know the present value of your fathers house?
_______________10000Yuan

***CA022_a.** Does your father shares the house with others?
(1) Yes
(2) No

[PROCEDURE: Add one more section for respondents mother, with questions identical to those asked about the respondents father.]

[PROCEDURE: Add two more sections for respondents father-in-law and mother-in-law, with questions identical to those asked about the respondents father. //]

[PROCEDURE: if married(B047 =1/2) ask CA023 first:] [: (B047 =1/2)CA023]

If the spouse is at home, let him/her answer the questions about his/her parents. /

CA023 . Who answers the questions about the spouses parents?
[IWER: Record]
(1) The family respondent
(2) The spouse

[PROCEDURE: if widow (CV033 = 5), ask CA024] [: (CV033= 5), CA024]
CA024. Do you keep in contact with your spouses parents? /
(1) Yes
(2) No

[PROCEDURE: If married (B047 =1/2) OR widowed but keeping in contact with spouses parents (CA024 =1), then ask information about spouses parents] [: (CV033 =1/2)/ (CA024 =1)/]

CB CHILDREARING INFORMATION

INTRODUCTION: Next, we will ask some questions about your fostering of your children/about raising your children.

[PROCEDURE: if the respondent is currently unmarried (BE001= 3 - 6), ask CB001-CB016] [: (BE001 = 3 - 6), CB001 -CB016]

CB001. Have you ever given birth to any child? If yes, how many are currently living, who are not living with you? _______ 0..25
[IWER: Non-HHmember child.Mark 0 if none 0]
[CAPI lists the names of HHmember children.] [CAPI]
[PROCEDURE: If not mark 0, ask CB002][0CB002]

CB002. What are the name of your biological children who are not living with you
[IWER: List names of those children]

CB003. How many biological children do you have who have passed away? _______ 0..25Persons [IWER: Mark 0 if none.0]

[PROCEDURE: Repeat questions CB004 ~CB008 for each child who have passed away. CB004 ~CB008]

****CB004.** When was this child born______(CB004_1)1900..2011Year _____(CB004_2)0..12Month

[Procedure: If CB004 =DK or RF, skip CB005 . CB004 =CB005]
****CB005.** Is your answer to CB020 based on the solar or lunar calendar? （） （）
（1） Solar calendar（）
（2） Lunar calendar（）

****CB006.** Sex of this child
（1） Male
（2） Female

****CB007.** When did this child pass away_______(CB007_1)1900..2011Year ______(CB007_2)0..12Month

[Procedure: If CB007 =DK or RF, skip CB008 . CB007 CB008]
CB008. Is your answer to BC000_1_3 based on the solar or lunar calendar? （） （）
（1） Solar calendar（）
（2） Lunar calendar（）

[Softcheck if the number entered for the year of death is smaller than the number entered for the year of birth. CB004 CB007]

CB009. Have you ever adopted or fostered any child or step child? If yes, how many are currently living whore not living with you? _______ 0..25Persons
[IWER: Non-HHmember child.Mark 0 if none 0]
[CAPI lists the names of HHmember children.] [CAPI]
[PROCEDURE: If not mark 0, ask CB010][0CB010]

CB010. What are the name of your adopted or fostered or step children who are not living with you
[IWER: List names of those children]

CB011. How many adopted or foster children or step children do you have who have passed away? _______ 0..25Persons [IWER: Mark 0 if none.0]
[PROCEDURE: Repeat questions CB012 ~CB016 for each child who have passed away. CB012 ~CB016]

******CB012.** When was this child born______(CB012_1)1900..2011Year _____(CB012_2) 0..12Month

[Procedure: If CB012 =DK or RF, skip CB013. CB012 =CB013]
******CB013.** Is your answer to BC000_2 based on the solar or lunar calendar? （）（）
（1）Solar calendar（）
（2）Lunar calendar（）

******CB014.** Sex of this child
（1）Male
（2）Female

******CB015.** When did this child pass away_______(CB015_1)1900..2011Year ______(CB015_2)0..12Month

[Procedure: If CB015 =DK or RF, skip CB016. CB015 CB016]
CB016. Is your answer to CB015 based on the solar or lunar calendar? （）（）
（1）Solar calendar（）
（2）Lunar calendar（）

[Softcheck if the number entered for the year of death is smaller than the number entered for the year of birth. CB012 CB015]

PROCEDURE: if the respondent is currently married (BE001 = 1/2), ask CB017 -CB043][: (BE001 = 1/2), CB017 - CB043]

CB017. How many biological children do you and your （current） spouse have together who are currently living and not living with you? （）_______ 0..25Persons
[IWER: Non-HHmember child.Mark 0 if none 0]
[CAPI lists the names of HHmember children.] [CAPI]
[PROCEDURE: If not mark 0, ask CB018][0CB018]

CB018. What are the names of those children who are not living with you
[IWER: List names of all those biological children.]

CB019. How many biological children do you and your （current）spouse have together who have passed away? （）_______ 0..25Persons [IWER: Mark 0 if none.0]

[PROCEDURE: Repeat questions CB020 ~CB024 for each child who have passed away. CB020 ~CB024]

******CB020.** When was this child born______(CB020_1)1900..2011Year _____(CB020_2)0..12Month

[Procedure: If CB020 =DK or RF, skip CB021. CB020 =CB021]
******CB021.** Is your answer to CB020 based on the solar or lunar calendar? （）（）
（1）Solar calendar（）

（2）Lunar calendar（）

****CB022.** Sex of this child
（1）Male
（2）Female

****CB023.** When did this child pass away_______(CB023_1)1900..2011Year ______(CB023_2)0..12Month

[Procedure: If CB023 =DK or RF, skip CB024. CB023 CB024]
CB024. Is your answer to CB023 based on the solar or lunar calendar? （）（）
（1）Solar calendar（）
（2）Lunar calendar（）

[Softcheck if the number entered for the year of death is smaller than the number entered for the year of birth. CB020 CB023]

[PROCEDURE: If A039 =1, skip to CB033][A039 =1CB033]

CB025. How many additional biological children do you have who are currently living and not living with you? （） _______ 0..25Persons
[IWER: Non-HHmember child.Mark 0 if none 0]
[CAPI lists the names of HHmember children.] [CAPI]
[PROCEDURE: If not mark 0, ask CB026][0CB026]

CB026. What are the names of those children who are not living with you
[IWER: List names of all those children]

CB027. How many additional biological children do you have who have passed away? （） _______ 0..25Persons [IWER: Mark 0 if none.0]

[PROCEDURE: Repeat questions CB028 ~CB032 for each child who have passed away. CB028 ~CB032]

****CB028.** When was this child born______(CB028_1)1900..2011Year _____(CB028_2)0..12Month

[Procedure: If CB028 =DK or RF, skip CB029. CB028 =CB029]
****CB029.** Is your answer to CB028 based on the solar or lunar calendar? （）（）
（1）Solar calendar（）
（2）Lunar calendar（）

****CB030.** Sex of this child
（1）Male
（2）Female

****CB031.** When did this child pass away_______(CB031_1)1900..2011Year ______(CB031_2) 0..12Month

[Procedure: If CB031 =DK or RF, skip CB032. CB031 CB032]
CB032. Is your answer to CB031 based on the solar or lunar calendar? （）（）
（1）Solar calendar（）
（2）Lunar calendar（）

[Softcheck if the number entered for the year of death is smaller than the number entered for the year of

birth. CB028 CB031]

CB033. How many additional biological children does your （current ） spouse have who are currently living and not living with you? （） _______ 0..25Persons
[IWER: Non-HHmember child.Mark 0 if none 0]
[CAPI lists the names of HHmember children.] [CAPI]
[PROCEDURE: If not mark 0, ask CB034][0CB034]

CB034. What are the names of those children who are not living with you
[IWER: List names of all those children]

CB035. How many additional biological children does your spouse have who have passed away? （）_______ 0..25Persons
[IWER: Mark 0 if none.0]

[PROCEDURE: Repeat questions CB036 ~CB040 for each child who have passed away. CB036 ~CB040]

*****CB036.** When was this child born______(CB036_1)1900..2011Year _____(CB036_2)0..12Month

[Procedure: If CB036 =DK or RF, skip CB037 . CB036 =CB037]
*****CB037.** Is your answer to CB036 based on the solar or lunar calendar? （） （）

（1）Solar calendar（）

（2）Lunar calendar（）

*****CB038.** Sex of this child

（1）Male

（2）Female

*****CB039.** When did this child pass away_______(CB039_1)1900..2011Year ______(CB039_2)0..12Month

[Procedure: If CB039 =DK or RF, skip CB040. CB007 CB008]
CB040. Is your answer to CB039 based on the solar or lunar calendar? （） （）

（1）Solar calendar（）

（2）Lunar calendar（）

[Softcheck if the number entered for the year of death is smaller than the number entered for the year of birth. CB036 CB039]

CB041. How many adopted or foster children or step children do you or your （current ）spouse have who are currently living and not living with you? （） _______ 0..25Persons
[IWER: Non-HHmember child..Mark 0 if none 0]
[CAPI lists the names of HHmember children.] [CAPI]
[PROCEDURE: If not mark 0, ask CB042][0CB042]

CB042. What are the names of those children who are not living with you
[IWER: List names of those children]

CB043. How many adopted or foster children do you or your （current）spouse have who have passed away?（）_______10..25Persons [IWER: Mark 0 if none.0]

[PROCEDURE: Repeat questions CB044 ~CB048 for each child who have passed away. CB044 ~CB048]

****CB044.** When was this child born______(CB044_1)1900..2011Year _____(CB044_2)0..12Month

[Procedure: If CB044 =DK or RF, skip CB045. CB044 =CB045]
****CB045.** Is your answer to BC000_2 based on the solar or lunar calendar? （）（）
（1）Solar calendar（）
（2）Lunar calendar（）

****CB046.** Sex of this child
（1）Male
（2）Female

****CB047.** When did this child pass away? _______(CB047_1)1900..2011Year ______（CB047_2）0..12Month

[Procedure: If CB047 =DK or RF, skip CB048. CB047 CB048]
CB048. Is your answer to BC000_1_3 based on the solar or lunar calendar? （）（）
（1）Solar calendar（）
（2）Lunar calendar（）

[Softcheck if the number entered for the year of death is smaller than the number entered for the year of birth. CB044 CB047]

[PROCEDURE: repeat questions CB049 ~CB079 for each living child. CB049 ~CB079]
[PROCEDURE: If the child is a household member, skip to CB064 ; for non-HHmember child, ask the following questionAsk the HHmember child first. We can get the number of hhmember child from CV. CB064;CB049 -CB079]

[PROCEDURE: Preload all the names of the children from the family roster and confirm with the respondent.]

Intro: We have asked some information of HHmember child in A section. Now we will ask the other children who are not living with you now. A

[IWER: Names of HHmember child have already been preloaded from A in CAPI. We only have to ask the non-hhmember childerns name. A]

****CB049 .** Is [CHILD's NAME] a boy or a girl? []
(1) Boy (Son) （）
(2) Girl (Daughter) （）

[Show Card 2]
****CB050.** What is the Chinese Zodiac sign for CHILDn's? []____________
[IWER: Choose from the list of Chinese Zodiac signssee Appendix1.]

****CB051.** Birth month and year for CHILDn's NAME? []_____1900..2011 (CB051_1)year _____0..12 (CB051_2)month

[IWER: Mark the year using four digits. Take down the month as its actual number. For example, write January as 1 not 01, December as 12. If do not remember month, fill 0. : 41101,12120]

****CB052. Is your answer to CB051 based on the solar or lunar calendar? （） （）
（1）Solar calendar（）
（2）Lunar calendar（）

[Show Card 4]

****CB053. Where does this CHILDn's NAME normally live now? []
(1) This household, but economicly independent.
(2) The same or adjacent dwelling/courtyard with me （）（）
(3) Another household in this village/neighborhood /
(4) Another village/neighborhood in this county/city; distance from here: ____km //: ______(CB053_1)_
(5) Another county/city in this province/___________（CB053_2）city______（CB053_3）_____ county, ____________(CB053_4)
(6) Another province _______(CB053_5)province //_________(CB053_6)city_________(CB053_7)county, ________(CB053_8)
[IWER: Choose from the list of provincessee appendix 2.]
(7) Abroad

****CB054. In what type of location does CHILDn's NAME live? []
(1) City
(2) County
(3) Town
(4) Village

****CB055. What is the current hukou status of CHILDn's NAME? []]
(1) Agriculture Hukou
(2) Non-Agriculture Hukou
(3) Unified Residency Hukou
(4) Does not have Hukou Skip to CB058 CB058
F1

****CB056. Is CHILDn's NAMEs hukou location the same as his/her place of residence? []?
(1) Yes Skip to CB081 CB081
(2) No

****CB057. Where is the current hukou place of CHILDn's NAME? []
(1) This household
(2) This village/neighborhood /
(3) Another village/neighborhood in this county/city //
(4) Anothercity in this province, specify _________ (CB057_1)
(5) Another province, province: (preloaded list) （） _________ (CB057_2)
[IWER: Choose from the list of provincessee appendix 2]
(6) Other(specify) () (CB057_3)_________

**** CB081. Where was the birth place of CHILD's NAME? []
(1) This village/neighborhood /
(2) Another village/neighborhood in this county/city //
(3) Another county/city in this province/___________(CB081_1)city__________ (CB081_2) county
(4)Another province _______(CB081_3)province //, ____________(CB081_4)city__________(CB081_5)

county [IWER: Choose from the list of provincessee appendix 2]
(5) Abroad

****CB082. Is [name]s present hukou status and location the same as his/her first Hukou? [

(5) Yes →Skip to precedure before CB058 CB058
(6) No

**** CB083. How did [name]s Hukou status or location change?
[]
(1)Both Hukou status and location have changed
(2)Only Hukou status has changed
(3)Only Hukou location has changed →Skip to CB085 CB085

**** CB084. What was [name]s first HuKou status? []
(3) Agricultural Hukou
(4) Non-agricultual Hukou

[If A083=2, skip CB085. A083=2CB085]

**** CB085. What was the location of [name]s first hukou? []
(1) This village/neighborhood /
(2) Another village/neighborhood in this county/city //
(3) Another county/city in this province/________ (CB085_1)city__________ county(CB085_2)
(4)Another province _______province //(CB085_3), _______ity(CB085_4) ______ county(CB085_5)[IWER: Choose from the list of provincessee appendix 2]
(5) Abroad

[PROCEDURE: If person less than 6, go to next person 6]

****CB058. Is CHILDn's NAME still in school now? []
(1) Yes
(2) No Skip to CB060 CB060

****CB059. What level of schooling and grade is CHILDn's NAME currently enrolled in? []
(1) Primary school grade 1 1
(2) Primary school grade 2 2
(3) Primary school grade 3 3
(4) Primary school grade 4 4
(5) Primary school grade 5 5
(6) Primary school grade 6 6
(7) Middle school grade 1 1
(8) Middle school grade 2 2
(9) Middle school grade 3 3
(10) Middle school grade 4 4
(11) High school, grade 1 1
(12) High school, grade 2 2
(13) High school, grade 3 3
(14) Vocational/technical high school year 1 1
(15) Vocational/technical high school year 2 2
(16) Vocational/technical high school year 3 3
(17) College year 1 /1
(18) College year 2 /2

(19) College year 3 /3
(20) College year 4 /4
(21) College year 5 /5
(22) College year 6 /66
(23) Masters degree
(24) Doctoral degree/Ph.D. degree

Skip to CB063 CB063

[PROCEDURE: If person less than 12, go to next person 12]

****CB060.** What is the highest level of education CHILDn's NAME completed? []
(1) No formal education (illiterate) （） Skip to CB063 . CB063
(2) Did not finish primary school but capable of reading or writing Skip to CB061 CB061
(3) Sishu/home school
(4) Elementary school Skip to CB062 CB062
(5) Middle school Skip to CB062 CB062
(6) High school Skip to CB062 . CB062
(7) Vocational school （） Skip to CB062 CB062
(8) Two-/Three-Year College / Associate degree Skip to CB062 CB062
(9) Four-Year College / Bachelors degree Skip to CB062 CB062
(10) Post-graduate, Masters degree Skip to CB062 CB062
(11) Post-graduate, doctoral degree/Ph.D. Skip to CB063 CB063

****CB061.** How many years did CHILDn's NAME spend in primary school? [] Skip to CB063 CB063

****CB062.** How many additional years of schooling did CHILDn's NAME receive after [THE ANSWER CHOSEN IN CB060]? [][CB060]

[PROCEDURE: If person less than 16, go to next person 16]

****CB063.** What is CHILDn's NAME marital status? []
（1）Married with spouse present
（2）Married but not living with spouse temporarily for reasons such as work
（3）Separated （）
（4）Divorced
（5）Widowed
（6）Never married

[Procedure: ask CB064 if child has any college education, i.e. CB059 =17-24 or CB060 =8,9,10 or 11 (CB059 =17-24CB060 =891011)CB064]
****CB064.** How much did you and your spouse spend to support this CHILDn's NAMEs college education []（）_________Yuan

[PROCEDURE: If the person has never married, please skip CB065 -CB068. CB065 -CB068]

****CB065.** How many sons does CHILDn's NAME have? []______ 0..25Persons

****CB066.** How many adult sons（above age 16）does CHILDn's NAME have? [](16) ______ 0..25Persons
[Softcheck if the number of sons is smaller than the number of adult sons. CB065 <CB066,]

****CB067.** How many daughters does CHILDn's NAME have? [] _ _____ 0..25Persons

****CB068.** How many adult daughters（above age 16） does CHILDn's NAME have? [](16)______ 0..25
[Softcheck if the number of daughters is smaller than the number of adult daughters. CB067 <CB068,]

****CB069.** Which category did the total income of CHILDn's NAME (and his/her spouse) in the past year belong to? [](/)
1) None
2) under 2,000 yuan 2
3) 2000 – 5000 yuan 25
4) 5000 – 10000 yuan 51
5) 10,000 - 20,000 yuan 12
6) 20,000 - 50,000 yuan 25
7) 50,000 - 100,000 yuan 510
8) 100,000 - 150,000 yuan 1015
9) 150,000 - 200,000 yuan 1520
10) 200,000 - 300,000 yuan 2030
11) Above 300,000 yuan 30

****CB070.** Is [Childs Name] working now (work includes agricultural work, earning wage work, self-employed activities, and unpaid family business work, et. al.)? []()
(1) Yes
(2) No Skip to CB074 CB074
[Softcheck if the age of the child is less than 16 and the child is reported to be working. 16CB070 =1]
[Softcheck if the child is reported to be in school and also is reported to be working.]

[Show Card 5]
****CB071.** What is [CHILDns NAME]s main occupation? []
(1) Managers
(2) Professionals and technicians
(3) Clerks
(4) Commercial and service workers
(5) Agricultural, forestry, husbandry and fishery producers
(6) Production and transportation workers

****CB072.** What is the highest administrative level that CHILDn's NAME has attained? []
(1) Team Leader （）
(2) Section Chief
(3) Director of a division
(4) Director-General of a bureau and above
(5) Township Leader
(6) None
F1

****CB073.** What is/was your CHILDn's NAME highest professional/technical level? []/
(1) Technician
(2) Primary level
(3) Intermediate level
(4) Advanced level
(5) None /
F1/

[PROCEDURE: IF CB070 =1, skip CB074 , CB075.] [: CB070 =1, CB074, CB075.]

****CB074.** Has [Childs Name] ever worked before? (Work includes agricultural work, earning wage work, self-employed activities, and unpaid family business work, et. al.) [] ()

(1) Yes
(2) No Skip to CB076 CB076

[Show Card 5]
****CB075.** What sort of work did CHILDn's NAME mainly do? []
(1) Managers
(2) Professionals and technicians
(3) Clerks
(4) Commercial and service workers
(5) Agricultural, forestry, husbandry and fishery producers
(6) Production and transportation workers

****CB076.** Did CHILDn's NAME live with others, away from you, before age 16 for more than six months? []166
(1) Yes
(2) No →Repeat questions for additional children

****CB077.** What was the earliest age that CHILDns NAME lived separately with others for more than six months? []_____ 1…16years of age
[Softcheck if the age reported here is older than the current age of the child. CB077]

****CB078.** With whom did CHILDn's NAME live for the longest period of time when not living with you or your spouse? []
(1) Your parents
(2) Your spouses parents
(3) Your brothers or sisters family
(4) Other family
(5) Dormitory
(6) Other

****CB079.** Cumulatively, how long did CHILDn's NAME live separately with others before age 16? []16______ 0.00..16.00 years _______(CB079_1) 0..11months

[PROCEDURE: Repeat questions for additional children]

[Intro: Now we will ask information about your grand children.]
[Procedure: For all HHMember, if the relationship is grandchild, A006 =9, Loop CB080. (A006 =9)CB080]

CB080. Who are [preload grand children who is HHmember]s parents?(circle all that applies) [CAPI lists the names of grandchildren who are household members.] [][CAPI] （）
(1)-(25)[CAPI lists the names of all the children.] [CAPI]
(26) _____(CB080_1)

CC SIBLINGS

[INTRO: Next I have some questions about your brothers and sisters.]

CC001. How many of your siblings are still alive? ___ 0..25siblings

[PROCEDURE: If CC001 =0 skip to CC003 . CC001 =0, CC003]

CC002. How many of your living siblings are:
Older brothers ______0..25 (CC002_1), Younger brothers _______ 0..25 (CC002_2)
Older sisters ______0..25 (CC002_3), Younger sisters ______0..25 (CC002_4)

[Softcheck if the total number of brothers and sisters reported in BD006 is not equal to the total number of siblings reported in CC001 . (CC002_1 + CC002_2 + CC002_3 +CC002_4) ≠CC001 .]

CC003. How many of your biological siblings have died? ? _______ 0..25 siblings

[PROCEDURE: If CC003 =0, skip to CC005. CC003=0, CC005]

*****__CC004 .__** How many of your deceased siblings were:
Older brothers ______0..25 (CC004_1), Younger brothers _______ 0..25 (CC004_2)
Older sisters ______0..25 (CC004_3), Younger sisters ______0..25 (CC004_4)
[Softcheck if the total number of brothers and sisters reported in CC004 is not equal to the total number of siblings reported in CC003 . (CC004_1 + CC004_2 + CC004_3 + CC004_4) ≠CC003 .]

[PROCEDURE: Repeat all sibling questions for the spouses siblings.]
If the respondent is separated or divorced, skip the questions about his/her spouses siblings. (Note: Even if the respondent is widowed, still ask BD000-1). / （/）

If the spouse is at home, let him/her answer the questions about his/her siblings /
CC005. Who answers the questions about the spouses siblings?
[IWER: Record.]
(1) The family respondent
(2) The spouse

CC006. How many of your spouses siblings are still alive? ___ 0..25

[PROCEDURE:If CC006 =0 skip to CC008. CC006 =0, CC008]

CC007. How many of your spouses living siblings are:
Older brothers ______0..25 (CC007_1), Younger brothers _______0..25 (CC007_2)
Older sisters ______0..25 (CC007_3), Younger sisters ______0..25 (CC007_4)
[Softcheck if the total number of brothers and sisters reported in BD016 is not equal to the total number of siblings reported in BD011. (CC007_1 + CC007_2 + CC007_3 + CC007_4)≠CC006]

CC008. How many biological siblings do your spouse or did your spouse have who died? ? _______ 0..25

[PROCEDURE: If CC008=0, skip to the procedure before CE001. CC008 =0, CE001]

******__CC009.__** How many of your spouses deceased siblings were:
Older brothers ______0..25 (CC009_1), Younger brothers _______ 0..25 (CC009_2)
Older sisters ______0..25 (CC009_3), Younger sisters ______0..25 (CC009_4)
[Softcheck if the total number of brothers and sisters reported in BD012 is not equal to the total number of siblings reported in CC009. (CC009_1 + CC009_2 + CC009_3 + CC009_4)≠CC008]

C2 TIME TRANSFER AND TRANSFERS

Introduction: In the following three parts: be time transfer, bf transfer and bg time spent providing care we will ask you how you contact with parents and children, and economic trancfers.

CD TIME TRANSFER

Contact with parents

[PROCEDURE: Skip to CD003 if father/mother/father-in-law/mother-in-law is not alive OR father/mother/father-in-law/mother-in-law is household member. //()/()//()/()CD003]

[LOOP: Repeat each living and non-resident father/mother/father-in-law/mother-in-law for CD001 -CD002 . CD001-CD002 //()/()]

********[4]CD001.** [If the father/mother/father-in-law/mother-in-law is alive] Whom does your father/mother/father-in-law/mother-in-law live with? [//()/()] //()/()

(1) By him/herself
(2) With my / my spouses older brother /
(3) With my / my spouses younger brother /
(4) With my / my spouses older sister /
(5) With my / my spouses younger sister/
(6) Take turns in childrens homes
(7) Nursing home
(8) Other

[Show Card 6]
********CD002.** How often do you/your spouse see your father/mother/father-in-law/mother-in-law? //()/()

(1) Almost every day
(2) 2-3 times a week2-3
(3) Once a week
(4) Every two weeks
(5) Once a month
(6) Once every three months
(7) Once every six months
(8) Once a year
(9) Almost never
(10) Other

Contact with Children
[PROCEDURE:If respondent has no non-cohabiting children, skip to CF001. CF001]
For each of the non-coresident child, ask the following two questions (CD003 and CD004). （CD003 CD004）

[Show Card 6]
******CD003.** How often do you see CHILDns NAME? []?

(1) Almost every day
(2) 2-3 times a week2-3

---

[4] **********, Please refer to LOOP questionnaire Parents2.dta**.** ******LOOPBE

(3) Once a week
(4) Every two weeks
(5) Once a month
(6) Once every three months
(7) Once every six months
(8) Once a year
(9) Almost never
(10) Other

[PROCEDURE: If CD003=1,2,3, skipCD004. CD003=1,2,3CD004]

[Show Card 6]

****CD004.** How often do you have contact with CHILDns NAME either by phone, text mess-age, mail, or email, when you didnt live with CHILDns NAME? [][]?

(1) Almost every day
(2) 2-3 times a week2-3
(3) Once a week
(4) Every two weeks
(5) Once a month
(6) Once every three months
(7) Once every six months
(8) Once a year
(9) Almost never
(10) Other

CE TRANSFERS

Introduction: Families sometimes help one another in a variety of ways, and each type of help can be important. The next questions are about help you (and your spouse) have given to or received from your non-coresident family members in the past year.

Receipt of Economic Assistance including Cash and in-kind Transfers （）

[PROCEDURE: Skip to the procedure before CE004 if both of father and mother died before 2010, or both of them are househould member, or one died before 2010, the other is houshouldmember, 20102010CE004]

CE001. In the past year, did you or your spouse receive any economics supports from your non-coresident parents?

(1) Yes
(2) No → Skip to CE004 CE004

CE002. How much did you receive from your non-coresident parents in the past year?(specify the amount of each type of economics transfers). （）

(1) Regular monetary or in-kind support (e.g., money or in-kind support every month/quarter/half year/year, at fixed time) （）

Regular monetary support ______ Yuan （CE002_1）

Regular in-kind support ______ Yuan （CE002_2）

(2) Non-regular monetary or in-kind support (e.g., money or in-kind support at Spring Festival or/and Mid-Autumn Festival or/and birthday or/and wedding or/and funeral or/and others) ()

Non-regular monetary support ______ Yuan （CE002_3）

Non-regular in-kind support ______ Yuan （CE002_4）

CE003: add unfolding brackets （ 100/200/400/800/1600yuan ） for each type of money. （100/200/400/800/1600 ）

[PROCEDURE: Skip to the procedure before CE007 if both of father-in-law and mother-in-law died before 2010, or both of them are househould member, or one died before 2010, the other is househouldmember, （） 2010 （）（） 2010CE007]

CE004 . In the past year, did you or your spouse receive any economics supports from your non-coresident parents-in-law? （）

(1) Yes

(2) No → Skip to CE007 CE007

CE005. How much did you receive from your non-coresident parents-in-law in the past year?(specify the amount of each type of economics transfers). （）（）

(1) Regular monetary or in-kind support (e.g., money or in-kind support every month/quarter/half year/year, at fixed time) （）

Regular monetary support ______ Yuan （CE005_1）

Regular in-kind support ______ Yuan （CE005_2）

(2) Non-regular monetary or in-kind support (e.g., money or in-kind support at Spring Festival or/and Mid-Autumn Festival or/and birthday or/and wedding or/and funeral or/and others) ()

Non-regular monetary support ______ Yuan （CE005_3）

Non-regular in-kind support ______ Yuan （CE005_4）

CE006 : add unfolding brackets （ 100/200/400/800/1600yuan ） for each type of money. （100/200/400/800/1600 ）

[PROCEDURE: Skip to the procedure before CE011 if the respondent and spouse have no non-coresident children.] [CE011 .]

CE007. In the past year, did you or your spouse receive any economics supports from your non-coresident children?

(1) Yes

(2) No → Skip to CE011 CE011

CE008 . Which child（ren）?（） (choose all that apply)

[Provide the list of children]

For each of the child checked in CE008 , ask CE009 . CE008 CE009

*******CE009.** How much of the following did you receive from this child [CHILDNAME] in the past year?(specify the amount of each type of economics transfers). （）

(1) Regular monetary or in-kind support (e.g., money or in-kind support every month/quarter/half year/year, at fixed time) （）

Regular monetary support ______ Yuan （CE009_1）

Regular in-kind support ______ Yuan （CE009_2）

(2) Non-regular monetary or in-kind support (e.g., money or in-kind support at Spring Festival or/and Mid-Autumn Festival or/and birthday or/and wedding or/and funeral or/and others) ()

Non-regular monetary support ______ Yuan （CE009_3）

Non-regular in-kind support ______ Yuan （CE009_4）

CE010 : add unfolding brackets （100/200/400/800/1600yuan） for each type of money. （100/200/400/800/1600 ）

[PROCEDURE: Skip to CE015 if the respondent and spouse have no non-coresident grandchildren or grandchildren are younger than 10 years.] [10CE015 .]

CE011. In the past year, did you or your spouse receive any economics supports from your non-coresident grandchildren?

(1) Yes

(2) No → Skip to CE015 CE015

CE012. Which child was the parent of the grandchildren? (choose all that apply)

[Provide the list of children]

For each of the child checked in CE012 , ask CE013 .CE012 CE013

*******CE013.** How much of the following did you receive from this childs [CHILDNAME] children in the past year? (specify the amount of each type of economics transfers). （）

(1) Regular monetary or in-kind support (e.g., money or in-kind support every month/quarter/half year/year, at fixed time) （）

Regular monetary support ______ Yuan （CE013_1）

Regular in-kind support ______ Yuan （CE013_2）

(2) Non-regular monetary or in-kind support (e.g., money or in-kind support at Spring Festival or/and Mid-Autumn Festival or/and birthday or/and wedding or/and funeral or/and others) ()

Non-regular monetary support ______ Yuan （CE013_3）

Non-regular in-kind support ______ Yuan （CE013_4）

CE014 : add unfolding brackets （100/200/400/800/1600yuan） for each type of money. （100/200/400/800/1600 ）

CE015. In the past year, did you or your spouse receive any economics supports from your non-coresident other relatives?

(1) Yes

(2) No → Skip to CE018 CE018

CE016. How much did you receive from your non-coresident other relatives in the past year?(specify the amount of each type of economics transfers). （）

(1) Regular monetary or in-kind support (e.g., money or in-kind support every month/quarter/half year/year, at fixed time) （）

Regular monetary support ______ Yuan （CE016_1）

Regular in-kind support ______ Yuan （CE016_2）

(2) Non-regular monetary or in-kind support (e.g., money or in-kind support at Spring Festival or/and Mid-Autumn Festival or/and birthday or/and wedding or/and funeral or/and others) ()

Non-regular monetary support ______ Yuan （CE016_3）

Non-regular in-kind support ______ Yuan （CE016_4）

CE017 : add unfolding brackets （100/200/400/800/1600yuan） for each type of money. （100/200/400/800/1600 ）

CE018. In the past year, did you or your spouse receive any economics supports from your non-coresident non-relatives (e.g. friends)? ()

(1) Yes

(2) No → Skip to CE021 CE021

CE019. How much did you receive from your non-coresident non-relatives in the past year?(specify the amount of each type of economics transfers). （）

(1) Regular monetary or in-kind support (e.g., money or in-kind support every month/quarter/half year/year, at fixed time) （）

Regular monetary support ______ Yuan （CE019_1）

Regular in-kind support ______ Yuan （CE019_2）

(2) Non-regular monetary or in-kind support (e.g., money or in-kind support at Spring Festival or/and Mid-Autumn Festival or/and birthday or/and wedding or/and funeral or/and others) ()

Non-regular monetary support ______ Yuan （CE019_3）

Non-regular in-kind support ______ Yuan （CE019_4）

CE020 : add unfolding brackets （100/200/400/800/1600yuan） for each type of money. （100/200/400/800/1600 ）

Provision of Economic Assistance including Cash and in-kind Transfers

（）

[PROCEDURE: Skip to the procedure before CE024 if both of father and mother died before 2010, or both of them are househould member, or one died before 2010, the other is househouldmember, 20102010CE024]

CE021. In the past year, did you or your spouse provide any economics supports to your non-coresident parents?

(1) Yes

(2) No → Skip to CE024 CE024

CE022. How much did you provide to your non-coresident parents in the past year?(specify the amount of each type of economics transfers). （）

(1) Regular monetary or in-kind support (e.g., money or in-kind support every month/quarter/half year/year, at fixed time) _ （）

Regular monetary support ______ Yuan （CE022_1）

Regular in-kind support ______ Yuan （CE022_2）

(2) Non-regular monetary or in-kind support (e.g., money or in-kind support at Spring Festival or/and Mid-Autumn Festival or/and birthday or/and wedding or/and funeral or/and others) ()

Non-regular monetary support ______ Yuan （CE022_3）

Non-regular in-kind support ______ Yuan （CE022_4）

CE023: add unfolding brackets（100/200/400/800/1600yuan）for each type of money.（100/200/400/800/1600 ）

[PROCEDURE: Skip to the procedure before CE027 if both of father-in-law and mother-in-law died before 2010, or both of them are househould member, or one died before 2010, the other is househouldmember, （）2010 （）（）2010CE027]

CE024. In the past year, did you or your spouse provide any economics supports to your non-coresident parents-in-law? （）

(1) Yes

(2) No → Skip to CE027 CE027

CE025. How much did you provide to your non-coresident parents-in-law in the past year?(specify the amount of each type of economics transfers). （）（）

(1) Regular monetary or in-kind support (e.g., money or in-kind support every month/quarter/half year/year, at fixed time) （）

Regular monetary support ______ Yuan （CE025_1）

Regular in-kind support ______ Yuan （CE025_2）

(2) Non-regular monetary or in-kind support (e.g., money or in-kind support at Spring Festival or/and Mid-Autumn Festival or/and birthday or/and wedding or/and funeral or/and others) ()

Non-regular monetary support ______ Yuan （CE025_3）

Non-regular in-kind support ______ Yuan （CE025_4）

CE026 : add unfolding brackets（100/200/400/800/1600yuan）for each type of money.（100/200/400/800/1600 ）

[PROCEDURE: Skip to the procedure before CE031 if the respondent and spouse have no non-coresident children, CE031]

CE027. In the past year, did you or your spouse provide any economics supports to your non-coresident children?

(1) Yes

(2) No → Skip to CE031 CE031

*******__CE028.__ Which child（ren）?（） (choose all that apply)

[Provide the list of children]

For each of the child checked in CE028 , ask CE029 .CE028 CE029

*******__CE029.__ How much of the following did you provide to this child [CHILDNAME] in the past year? (specify the amount of each type of economics transfers). []（）

(1) Regular monetary or in-kind support (e.g., money or in-kind support every month/quarter/half year/year, at fixed time) （）

Regular monetary support ______ Yuan （CE029_1）

Regular in-kind support ______ Yuan （CE029_2）

(2) Non-regular monetary or in-kind support (e.g., money or in-kind support at Spring Festival or/and Mid-Autumn Festival or/and birthday or/and wedding or/and funeral or/and others) ()

Non-regular monetary support ______ Yuan （CE029_3）

Non-regular in-kind support ______ Yuan （CE029_4）

CE030 : add unfolding brackets （100/200/400/800/1600yuan）for each type of money.（100/200/400/800/1600）

[PROCEDURE: Skip to CE035 if the respondent and spouse have no non-coresident grandchildren, CE035]

CE031. In the past year, did you or your spouse provide any economics supports to your non-coresident grandchildren?

(1) Yes

(2) No → Skip to CE035 CE035

*******CE032.** Which child was the parent of the grandchildren? (choose all that apply)

[Provide the list of children]

For each of the child checked in CE032 , ask CE033 .CE032 CE033

*******CE033.** How much of the following did you provide to this childs [CHILDNAME] children in the past year?(specify the amount of each type of economics transfers). （）

(1) Regular monetary or in-kind support (e.g., money or in-kind support every month/quarter/half year/year, at fixed time) （）

Regular monetary support ______ Yuan （CE033_1）

Regular in-kind support ______ Yuan （CE033_2）

(2) Non-regular monetary or in-kind support (e.g., money or in-kind support at Spring Festival or/and Mid-Autumn Festival or/and birthday or/and wedding or/and funeral or/and others) ()

Non-regular monetary support ______ Yuan （CE033_3）

Non-regular in-kind support ______ Yuan （CE033_4）

CE034 : add unfolding brackets （ 100/200/400/800/1600yuan ） for each type of money. （100/200/400/800/1600 ）

CE035. In the past year, did you or your spouse provide any economics supports to your non-coresident other relatives?

(1) Yes

(2) No → Skip to CE038 CE038

CE036. How much did you provide to your non-coresident other relatives in the past year?(specify the amount of each type of economics transfers). （）

(1) Regular monetary or in-kind support (e.g., money or in-kind support every month/quarter/half year/year, at fixed time) （）

Regular monetary support ______ Yuan （CE036_1）

Regular in-kind support ______ Yuan （CE036_2）

(2) Non-regular monetary or in-kind support (e.g., money or in-kind support at Spring Festival or/and Mid-Autumn Festival or/and birthday or/and wedding or/and funeral or/and others) ()

Non-regular monetary support ______ Yuan （CE036_3）

Non-regular in-kind support ______ Yuan （CE036_4）

CE037: add unfolding brackets（100/200/400/800/1600yuan）for each type of money.（100/200/400/800/1600 ）

CE038. In the past year, did you or your spouse provide any economics supports to your non-coresident non-relatives (e.g. friends)? ()

(1) Yes

(2) No → Skip to CE041 CE041

CE039. How much did you provide to your non-coresident non-relatives in the past year?(specify the amount of each type of economics transfers). （）

(1) Regular monetary or in-kind support (e.g., money or in-kind support every month/quarter/half year/year, at fixed time) （）

Regular monetary support ______ Yuan （CE039_1）

Regular in-kind support ______ Yuan （CE039_2）

(2) Non-regular monetary or in-kind support (e.g., money or in-kind support at Spring Festival or/and Mid-Autumn Festival or/and birthday or/and wedding or/and funeral or/and others) ()

Non-regular monetary support ______ Yuan （CE039_3）

Non-regular in-kind support ______ Yuan （CE039_4）

CE040: add unfolding brackets（100/200/400/800/1600yuan）for each type of money.（100/200/400/800/1600 ）

CE041. Have you or your spouse ever given a large amount of money or major assets (worth more than 5000 yuan) to any of your children, not including transfers made for the childs college education（Not including the large amount of money or major assets (worth more than 5000 yuan) which was mentioned before）. This can include cash, land, housing, or other assets, given as inheritance, at the time of the childs marriage, to support medical expenses or other emergencies, to support grandchildrens education or welfare, to avoid taxation, or for any other reason. （5000）（5000）（/）

(1) Yes

(2) No skip to CE046 CE046

CE042 . To which children did you give the money or assets? [CAPI: preload list of childrens names] /[CAPI:]

[For each selected child, ask:] [CE042]

*******__CE043.__ What was the value of the money or assets to [CHILDNAME]? []_________ yuan

*******__CE044.__ In what year was the (largest) gift made to [CHILDNAME]? []_______ 1900..2011year

*******__CE045.__ What was the reason for the gift to [CHILDNAME]? []

(1) pay for medical care or other emergency expense,

(2) pay for house

(3) avoid taxation

(4) Inheritance

(5) support grandchildrens education or welfare /

(6) other(explain) ____()________(CE045_1)

CE046 . Have you or your spouse ever received a large amount of money or major assets (worth more than 5000 yuan) including cash, land, or property from any of your children（Not including the large amount of money or major assets (worth more than 5000 yuan) which was mentioned before）? This includes paying for medical expenses or other emergecies, paying for housing, or for any other reason. （5000）（5000）
(1) Yes
(2) No skip to CE051 CE051

CE047 . From which children did you receive the money or assets? [CAPI: preload list of childrens names] / [CAPI:]

[For each selected child, ask:] [CE047]

*********CE048.** What was the value from [CHILDNAME]? []_________ yuan

*********CE049.** In what year was the (largest) gift made from [CHILDNAME]? []_______ 1900..2011year

*********CE050.** What was the reason for the gift from [CHILDNAME]? []?
(1) pay for medical care or other emergency expense
(2) pay for house
(3) avoid taxation
(4) other(explain) ____()________(CE050_1)

*********CE051.** Have you or your spouse ever given a large amount of money or major assets (worth more than 5000 yuan) to your parents（Not including the large amount of money or major assets (worth more than 5000 yuan) which was mentioned before）This can include cash, land, housing, or other assets. （5000）（5000）.
(1)Yes
(2）No skip to CE055 CE055

*********CE052.** What was the value of the money or assets to your parents? _________ yuan

*********CE053.** In what year was the (largest) gift made to your parents? _______ 1900..2011year

*********CE054.** What was the reason for the gift to your parents?
(1) pay for medical care or other emergency expense,
(2) pay for house
(3) avoid taxation
(4) Inheritance
(5) other(explain) ____()________(CE054_1)

*********CE055.** Have you or your spouse ever received a large amount of money or major assets (worth more than 5000 yuan) to your parents（Not including the large amount of money or major assets (worth more than 5000 yuan) which was mentioned before）This can include cash, land, housing, or other assets.（5000）(5000）
(1)Yes
(2）No skip to CE059 CE059

*********CE056.** What was the value of the money or assets from your parents? _________ yuan

*********CE057.** In what year was the (largest) gift made from your parents? _______ 1900..2011year

*********CE058.** What was the reason for the gift from your parents?

(1) pay for medical care or other emergency expense,
(2) pay for house
(3) avoid taxation
(4) Inheritance
(5) other(explain) ____()______

*******CE059 . Have you or your spouse ever given a large amount of money or major assets (worth more than 5000 yuan) to your parents-in-law（Not including the large amount of money or major assets (worth more than 5000 yuan) which was mentioned before）This can include cash, land, housing, or other assets. /（5000）（5000）.
(1) Yes
(2）No　　skip to CE063　　CE063

*******CE060. What was the value of the money or assets to your parents-in-law?　/_________ yuan

*******CE061. In what year was the (largest) gift made to your parents-in-law? /_______ 1900..2011year

*******CE062.　What was the reason for the gift to your parents-in-law? /
(1) pay for medical care or other emergency expense,
(2) pay for house
(3) avoid taxation
(4) Inheritance
(5) other(explain) ____()________ (CE062_1)

*******CE063. Have you or your spouse ever received a large amount of money or major assets (worth more than 5000 yuan) to your parents-in-law（Not including the large amount of money or major assets (worth more than 5000 yuan) which was mentioned before）This can include cash, land, housing, or other assets. /（5000）（5000）
(1)Yes
（2）　skip to CF001　　CF001

*******CE064. What was the value of the money or assets from your parents-in-law?　_________ yuan

*******CE065. In what year was the (largest) gift made from your parents-in-law? _______ 1900..2011year

*******CE066.　What was the reason for the gift from your parents-in-law?
(1) pay for medical care or other emergency expense,
(2) pay for house
(3) avoid taxation
(4) Inheritance
(5) other(explain) ____()______(CE066_1)

CF　TIME SPENT PROVIDING CARE

[PROCEDURE: Based on BC019-BC022, if the respondent has any grandchildren under 16, ask CF001 -CF003 ; otherwise, skip to CF004 . 16/CF001 - CF003 ; CF004 .]

CF001. Did you spend any time taking care of your grandchildren last year? /
(1) Yes

(2) No → Skip to CF004 CF004

********[5]**CF002.** For which childs children did you provide care?
[IWER: Please list all the children including coresident ones, and add a choice deceased children in CAPI list. Select from list displayed by CAPI (childs name)]

[PROCEDURE: Repeat question CF003 according to the list of names in CF002 . : CF002 CF003]
*********CF003.** Approximately how many weeks and how many hours per week did you spend last year taking care of this childs children?
Myself ______(CF003_1)weeks __________(CF003_2) 0.00..168.00hours per week /
My spouse ____ （CF003_3） weeks ______ （CF003_4） 0.00..168.00hours per week /
[IWER: Mark '1' if the period is less than 7 days. 71]

[Softcheck: if more than 52 weeks are reported or more than 140 hours are reported. 52140]

CF004. Did you or your spouse take care of your parents or parents-in-law during the last year in assisting them in their daily activities or other activities (e.g., household chores, meal preparation, laundry, going out, grocery shopping, financial management, etc.)? （）（）

(1) Yes
(2) No → Skip to CG001 CG001

CF005. Approximately how many weeks and how many hours per week did you yourself spend last yea r taking care of your parents or parents-in-law? （）

(1) Your father ____(CF005_1)weeks; _____(CF005_2)0.00..168.00hours per week/
(2) Your mother ____(CF005_3)weeks; _____(CF005_4)0.00..168.00hours per week/
（3）Your father-in-law （） ____(CF005_5)weeks; ___(CF005_6)0.00..168.00hours per week/
（4）Your mother-in-law （） ____(CF005_7)weeks; ___(CF005_8)0.00..168.00hours per week/

[Softcheck: if (1) is checked in CF004 and 0 or missings are reported in CF005.CF004 =1,CF005 =0]
[Softcheck: if more than 52 weeks are reported or more than 140 hours are reported. 52140]

CF006. Approximately how many weeks and how many hours per week did your spouse spend last ye ar taking care of your parents or parents-in-law? （）

(1) Your father ____(CF006_1)weeks; _____(CF006_2)0.00..168.00hours per week/
(2) Your mother ____(CF006_3) weeks; _____(CF006_4)0.00..168.00hours per week/
（3）Your father-in-law （） ____(CF006_5)weeks; ___(CF006_6)0.00..168.00hours per week/
（4）Your mother-in-law （ ____(CF006_7)weeks; ___(CF006_8)0.00..168.00hours per week/

[Softcheck: if (1) is checked in CF004 and 0 or missings are reported in CF006 .CF004 =1,CF006 =0]
[Softcheck: if more than 52 weeks are reported or more than 140 hours are reported. 52140]

CG Living Arrangements preferences :

[PROCEDURE: Main respondent and spouse both answers CG001 and CG002] [CG001 CG002]

[5] ********, Please refer to LOOP questionnaire Dailycare.dta. ********LOOPBh

CG001. Suppose an elderly person has a spouse and adult children, and has good relationship with them What do you think is the best living arrangement for the elderly person? ?

(1) Live with adult children
(2) Dont live with them in the same house, but live in the same community or village. /
(3) Dont live with them in the same house and the same community or village. /
(4) Live in a nursing house
(5) Other

CG002. Suppose an elderly person has no spouse but has adult children, and has good relationship with them. What do you think is the best living arrangement for him/her? ?

(1) Live with adult children
(2) Dont live with them in the same house, but live in the same community or village. /
(3) Dont live with them in the same house and the same community or village./
(4) Live in a nursing house
(5) Other

CG003. How often did the respondent receive assistance in answering section C -FAMILY? [IWER: If it is answered by a proxy, please record the respondents reaction.] C []

(1) Never
(2) A few times
(3) Most or all of the time

D HEALTH STATUS AND FUNCTIONING

DA HEALTH STATUS

SKIP PATTERN CHECKPOINT: SELF-REPORTED HEALTH STATUS
TWO SCALES ARE USED TO MEASURE SELF-REPORTED HEALTH STATUS. R WILL BE ASKED TO RATE THEIR HEALTH STATUS TWICE, ONCE AT THE BEGINNING OF THIS SECTION AND AGAIN AT THE END OF THE SECTION. QUESTION ORDER WILL BE ASSIGNED RANDOMLY.

IF R IS RANDOMLY ASSIGNED TO ORDER 1(SEC_DA _LIST=1), SKIP TO DA001
IF R IS RANDOMLY ASSIGNED TO ORDER 2(SEC_DA _LIST=2), SKIP TO DA002

（DA =1） DA001
（DA =2） DA002

PART I: GENERAL HEALTH STATUS AND DISEASE HISTORY

DA001. Next, I have some questions about your health. Would you say your health is excellent, very good, good, fair, or poor?
[IWERInterviewer should read all the following options]
(1) Excellent
(2) Very good
(3) Good
(4) Fair
(5) Poor

DA002. Next, I have some questions about your health. Would you say your health is very good, good, fair, poor or very poor?
[IWERInterviewer should read all the following options]
(6) Very good
(7) Good
(8) Fair
(9) Poor
(10) Very poor

[IWER: Please do not ask proxy the following questions from CA005-CA006. DA003 -DA004]

DA003. Do you ever feel pain on the left side of your chest?
(1) Yes
(2) No

DA004. Do you ever feel chest pains when climbing stairs/uphill or walking quickly?
(1) Yes
(2) No
(3) Not applicable

DA005. Do you have one of the following disabilities?

(1) Physical disabilities
(2) Brain damage/mental retardation /
(3) Vision problem
(4) Hearing problem
(5) Speech impediment

[Skip patternIf respondent says no to all of the above, skip to DA007 DA007]

DA006. In what year did you become disabled? [preload DA005 answer]____ 1900..2011Year
[IWER: Mark the year using four digits. 4]

[Show Card 7]
DA007. Have you been diagnosed with [conditions listed below, read one by one] by a doctor? [IWER: Read one by one. 1=yes, 2=no. 1=
2=]

(1) Hypertension
(2) Dyslipidemia (elevation of low density lipoprotein, triglycerides (TGs),and total cholesterol, or a low high density lipoprotein level)（（））
(3) Diabetes or high blood sugar（）
(4) Cancer or malignant tumor (excluding minor skin cancers) （）
(5) Chronic lung diseases, such as chronic bronchitis , emphysema (excluding tumors, or cancer) （）
(6) Liver disease (except fatty liver, tumors, and cancer)
（）
(7) Heart attack, coronary heart disease, angina, congestive heart failure, or other heart problems （）
(8) Stroke
(9) Kidney disease (except for tumor or cancer) （）
(10) Stomach or other digestive disease (except for tumor or cancer) （）
(11) Emotional, nervous, or psychiatric problems
(12) Memory-related disease （）
(13) Arthritis or rheumatism
(14) Asthma

[RPOGRAMAsk DA008 only if CA010 = 1, 5, 11, and CA010 = no.] [: DA007 1,5, 11,DA008.]

DA008. Do you know if you have [preload the current choice in DA007]? [DA007 1,5, 11]
(1) Yes
(2) No

CAPI: If diagnosed or known by R:

Ask the following 2 questions for each condition (item 1- 14 in DA007) he/she named. Do not allow anser to these questions if no disease named in DA007
/ DA007 1-14/DA007 /
DA009. When was the condition first diagnosed or known by yourself ? […]_____（DA009_1）
1900..2011Year______（DA009_2） 1…120Age
[IWER: Mark the year using four digits.: 4]

[PROCEDURE: Answer DA010 if DA007 = 2, 5, 6, 7, 9, 10, 12, 13 or DA008=1,5,11.]
[DA007 =2, 5, 6, 7, 9, 10, 12, 13DA008=1,5,11DA010 .]
DA010. Are you now taking any of the following treatments to treat […] or its complications (Check all that apply)? Taking Chinese traditional medicine, taking Western modern medicine, other treatments? […]()（）

[IWER: Read one by one.]
(1) Taking Chinese traditional medicine (DA010_1)
(2) Taking Western morden medicine (DA010_2)
(3) Other treatments (DA010_3)
(4) None of the above (DA010_4)

[ROCEDURE: If DA007 =1 then answerDA011.] [DA007 =1DA011 .]
DA011. Are you now taking any of the following treatments to treat or control your hypertension?(Check all that apply) Taking Chinese traditional medicine, taking Western modern medicine?（）

[IWER: Read one by one. 1=yes,2=no. 1=
2=]
(1) Taking Chinese traditional medicine
(2) Taking Western morden medicine
(3) None of the above

DA012. During last year (last 12 months), how many times have you had blood pressure examination? （12）
└─┴─┴─┘ 0...999Times

DA013. Have your care providers ever given you health education/advice on the following (check all that apply)? Weight control, exercise, diet and/or smoking control? （）
[IWER: Read one by one. 1=yes, 2=no. 1=
2=]
(1) Weight control
(2) Exercise
(3) Diet
(4) Smoking control
(5) None of the above

[PROCEDURE: IfDA007 =3 then answerDA014. DA007 =3DA014]
DA014. Are you now taking any of the folloing treatments to treat or control your diabetes?(Check all that apply) Taking Chinese traditional medicine, taking Western morden medicine ,taking insulin injections?（）

[IWER: Read one by one. 1=yes, 2=no. 1=
2=]
(1) Taking Chinese traditional medicine
(2) Taking Western modern medicine
(3) Taking insulin injections
(4) None of the above

DA015. During last year (last 12 months), how many times have you had the following? （12）
(1) Blood glucose test (DA015_1) └─┴─┴─┘ 0..999Times
(2) Urine glucose test (DA015_2) └─┴─┘ 0..99Times
(3) fundus examination (DA015_3) └─┴─┘ 0..99 Times
(4) micro-albuminuria test (DA015_4) └─┴─┘ 0..99 Times
(5) None of the above

DA016. Have your care providers ever given you health education/advice on the following? (check all that apply) （）
[IWER: Read one by one. 1=yes, 2=no. 1=
2=]
(1) Weight control

(2) Exercise
(3) Diet
(4) Smoking control
(5) Foot self-care
(6) None of the above

[PROCEDURE: If DA007 =4 Cancer or a malignant tumor, excluding minor skin cancers then answer DA018.] [DA007 =4（）DA017 DA018.]

[Show Card 8]

DA017. In which organ or part of your body do you have cancer? Including the origins and metastasis of tumor.（circle all that apply）（）

[IWER: Read one by one. We should still ask R even if he/she has already been cured.]

(1) Brain
(2) Oral cavity
(3) Larynx
(4) Other pharynx
(5) Thyroid
(6) Lung
(7) Breast
(8) Oesophagus
(9) Stomach
(10) Liver
(11) Pancreas
(12) Kidney
(13) Prostate
(14) Testicle
(15) Ovary
(16) Cervix
(17) Endometrium
(18) Colon or rectum
(19) Bladder
(20) Skin
(21) Non-Hodgkin lymphoma （）
(22) Leukemia
(23) Other organ (DA017_1)

DA018. Have you taken any of the following treatments to treat your canser or relieve its/their symptoms (e.g., pain, nausea, etc.) in the past two years? (Check all that apply) Taking Chinese traditional medicine ,taking Western morden medicine ,chemotherapy ,surgery ,radiation therapy?()

[IWER: Read one by one.
]

(1) Taking Chinese traditional medicine
(2) Taking Western morden medicine
(3) Chemotherapy
(4) Surgery
(5) Radiation therapy
(6) None of the above

F1（1）()
（2）
（3）

PROCEDURE: If DA007 =8, then answer DA019. DA007 =8DA019

DA019. Are you now taking any of the folloing treatments because of your stroke?(Check all that apply) Taking Chinese traditional medicine ,taking Western morden medicine , physical therapy, acupuncture and moxibustion , occupational therapy?（）

[IWER: Read one by one. 1=yes, 2=no. 1=
2=]

(1) Taking Chinese traditional medicine
(2) Taking Western morden medicine
(3) Physical therapy
(4) Acupuncture and moxibustion
(5) Occupational therapy
(6) None of the above

F1（1）（）
（2）
（3）//

PROCEDURE: If DA007 =11 or DA008 =11 then answer DA020 DA007 =11DA008=11DA020.

DA020. Are you now taking any of the folloing treatments for your emotional, nervous, or psychiatric problems?(Check all that apply) Receiving psychiatric or psychological treatment, taking anti depressants ,taking tranquilizers or sleeping pills?（）

[IWER: Read one by one. 1=yes, 2=no. 1=
2=]

(1) Receiving psychiatric or psychological treatment
(2) Taking anti depressants
(3) Taking tranquilizers or sleeping pills
(4) None of the above

[IWER: Please do not ask proxy the following questions from DA021 -DA078 . DA021 -DA078]
DA021. Have you ever been in a traffic accident or any other kind of major accidental injury and received medical treatment?
(1)Yes
(2)No → Skip to DA023 DA023

DA022. Does your injury caused by the accident limit your daily activities?
(1) Yes
(2) No

DA023. Have you fallen down in the last two years?
(1) Yes
(2) No → Skip to DA025 DA025

F124

DA024. How many times have you fallen down in the last two years seriously enough to need medical treatment?? ?_________ times

F124

DA025. Have you ever fractured your hip?
(1) Yes
(2) No

F1,

SKIP PATTERN CHECKPOINT: PROSTATE ILLNESS/INCONTINENCE : /
IF R IS MALE, SKIP TO DA029. DA029

DA026. When did you begin the menarche?
_____1900..2011 (DA026_1)Year[IWER: Mark the year using four digits. [: 4]
Or Age______1…120 (DA026_2) Years

DA027. Have you started menopause?
(1) Yes
(2) No →Skip to DA032 DA032

DA028. When did you begin the menopause?
_____1900..2011 (DA028_1)Year[IWER: Mark the year using four digits. : 4]
Or Age______1…120 (DA028_2) Years →Skip to DA032 DA032

DA029. Have you ever been diagnosed with a prostate illness, such as prostate hyperplasia（excluding prostatic cancer）? ()
(3) Yes
(4) No →Skip to DA032 DA032

F1

DA030. When was the condition first diagnosed?
_____1900..2011 (DA030_1)Year[IWER: Mark the year using four digits. : 4]
Or Age______1…120 (DA030_2) Years

DA031. Are you now taking medication or other treatment for your prostate illness?
(1) Yes
(2) No

DA032. Now I have some questions about your eyesight. Do you usually wear glasses or corrective lenses? （）
(1) Yes
(2) Legally blind → Skip to DA038 DA038
(3) No

DA033. How good is your eyesight for seeing things at a distance, like recognizing a friend from across the street (with glasses or corrective lenses if you wear them)? Would you say your eyesight for seeing things at a distance is excellent, very good, good, fair, or poor? ()
(1) Excellent
(2) Very good
(3) Good
(4) Fair
(5) Poor

DA034. How good is your eyesight for seeing things up close, like reading ordinary newspaper print (with glasses or corrective lenses if you wear them)? Would you say your eyesight for seeing things up close is excellent, very good, good, fair, or poor?
(1) Excellent

(2) Very good
(3) Good
(4) Fair
(5) Poor

DA035. Have you ever had cataract surgery?
(1) Yes
(2) No → Skip to DA037 DA037

DA036. Have you had cataract surgery on both eyes or just one?
(1) One eye only
(2) Both eyes

DA037. Has a doctor/nurse/paramedical/ doctor of traditional Chinese medicine doctor ever treated you for glaucoma?
(1)Yes
(2)No

DA038. Now I have some questions about your hearing. Do you ever wear a hearing aid?
(1) Yes
(2) No

DA039. Is your hearing very good, good, fair, poor, or very poor (with a hearing aid if you normally use it and without if you normally dont)? Would you say your hearing is excellent, very good, good, fair, or poor? ()
(1) Excellent
(2) Very good
(3) Good
(4) Fair
(5) Poor

DA040. Have you lost all of your teeth?
(1) Yes
(2) No

DA041. Are you often troubled with any body pains?
(1) Yes
(2) No skip to DA044 DA044

[Show Card 9]
DA042. On what part of your body do you feel pain? Please list all parts of body you are currently feeling pain.
(1) Head (Headache)
(2) Shoulder
(3) Arm
(4) Wrist
(5) Fingers
(6) Chest
(7) Stomach (Stomachache)
(8) Back
(9) Waist
(10) Buttocks
(11) Leg
(12) Knees
(13) Ankle
(14) Toes

(15) Neck

DA043. How bad is your pain (if more than one type of pain, ask about the most severe one among them)? Mild, Moderate or Severe? （）

(1) Mild
(2) Moderate
(3) Severe

[Show Card 10]
DA044. Have you been diagnosed with any of the following infectious diseases?

（1）Tuberculosis
（2）hepatitis B
（3）Malaria
（4）Influenza
（5）Rabies
（6）Schistosomiasis
（7）AIDS patients and infecious ones
（8）Encephalitis B
（9）Dysentery
(10) Measles
(11) Brucellosis
(12) Gonorrhea
(13) Syphilis
(14) Others Pls specify (DA044_1)
(15) None

DA045. Are there any other medical diseases or conditions that are important to your health now that we have not talked about?

(1) Yes →Skip toDA046 DA046
(2) No →Skip toDA047 DA047

DA046.. What illness is that? ________________________________

DA047. Have you gained or lost 5 or more kilograms in the last year? (excluding pregnancy) 1010（）

(1) Yes, I only gained weight
(2) Yes, I only lost weight
(3) Yes, I first gained and then lost weight
(4) Yes, I first lost and then gained weight
(5) No
(6) I dont know

DA048. How would you evaluate your health during childhood, up to and including age 15? Excellent, very good, good,fair, poor? 15（15）

(1) Excellent
(2) Very Good
(3) Good
(4) Fair
(5) Poor

PART II: LIFESTYLE AND HEALTH BEHAVIORS

DA049. During the past month, how many hours of *actual sleep* did you get at night (average hours for one night)? (This may be shorter than the number of hours you spend in bed.) （） _____0.00..24.00hours

DA050. During the past month, how long did you take a nap after lunch? _____minutes
[IWERIf R didnt take a nap,please record for 0. 0.]

[PROCEDURE: DA051 will be presented ONLY to a random subsample of households (half). Main respondent and spouse in the selected households should answer DA051] [DA051 DA051 .]

DA051. Now we would like to ask about the amount of time you spend on different types of physical activities in a usual week.

**PHYSICAL ACTIVITIES**<br>**(KKTYPE)**	**DA051.**<br>During **a usual week**, did you do any [….] for at least 10 minutes continuously? [...]	**DA052.**<br>During a usual week, on how many days did you do [….] for at least 10 minutes?[…]	How much time did you usually spend doing [….] on one of those days? […][…]	
**A.** Now, think about all the **vigorous activities** requiring hard/high-intensity physical effort that you do in **a usual week**. Vigorous activities make you breathe much harder than normal and may include heavy lifting, digging, plowing, aerobics, fast bicycling, and cycling with a heavy load. Think only about those physical activities that you did for at least 10 minutes at a time.	3. No🡻<br>1. Yes🡺	└──┘ 1..7 days	**DA053**<br>1. < 2 hours	**DA054**<br>1. < 30 minutes<br>2. ≥ 30 minutes
			2. ≥ 2 hours	**DA055**<br>3. < 4 hours<br>4. ≥ 4 hours
**B.** Now think about activities which take **moderate physical effort** that you do in a usual week. Moderate physical activities make you breathe somewhat harder than normal and may include carrying light loads, bicycling at a regular pace, or mopping the floor. Again, think about only those physical activities that you did for at least 10 minutes at a time.	3. No🡻<br>1. Yes🡺	└──┘ 1..7days	**DA053**<br>1. < 2 hours	**DA054**<br>1. < 30 minutes<br>2. ≥ 30 minutes

			2. ≥ 2 hours	**DA055**<br>3. < 4 hours<br>4. ≥ 4 hours
**C.** Now think about the time you spend **walking** in a usual week. This includes at work and at home, walking to travel from place to place, and any other walking that you might do solely for recreation, sport, exercise, or leisure.	3. No🡻<br>1. Yes➔	└──┘ 1..7 days	**DA053**<br>1. < 2 hours	**DA054**<br>1. < 30 minutes<br>2. ≥ 30 minutes
			2. ≥ 2 hours	**DA055**<br>3. < 4 hours<br>4. ≥ 4 hours

ACTIVITIES IN LAST MONTH

[Show Card 11]
DA056. Have you done any of these activities in the last month? (Code all that apply) ()

(1) Interacted with friends

(2) Played Ma-jong, played chess, played cards, or went to community club

(3) Provided help to family, friends, or neighbors who do not live with you and who did not pa y you for the help

(4) Went to a sport, social, or other kind of club

(5) Took part in a community-related organization

(6) Done voluntary or charity work

(7) Cared for a sick or disabled adult who does not live with you and who did not pay you f or the help

(8) Attended an educational or training course

(9）Stock investment （）

（10）Used the Internet

（11）other

(12) None of these

[CHECK: You cannot select 'None of these' together with any other answer. Please change your answer.]
LOOP cnt = 1 to 10 **1－10**

|*IF cnt IN* DA056 *(ACTIVITIES IN LAST MONTH)* DA056 1－10（）

DA057. Frequency of activity in the last month
How often in the last month [did/have][you] [do voluntary or charity work/cared for a sick or disabled adult/provided help to family, friends or neighbors/attended an educational or training course/ Interacted with friends /go to a sport,social or other kind of club/taken part in a community-related organization]? Almost daily, almost every week, or not regularly? （）

(1) Almost daily
(2) Almost every week
(3) Not regularly

DA058. How many meals do you normally eat every day? More than 4 meals per day,4 meals per day,3 meals per day ,2 meals per day,1 meal per day or <1 meal per day?4,4,3,2,,?[IWER:Read each choice of the answers.]

(1) More than 4 meals per day 4
(2) 4 meals per day 4
(3) 3 meals per day 3
(4) 2 meals per day 2
(5) 1 meal per day
(6) <1 meal per day

INTRO. Next, I would like to ask whether you have had the habit of smoking cigarettes/smoking a pipe/chewing tobacco, now or in the past. By smoking we mean smoking more than 100 cigarettes in your life) （100）

DA059. Have you ever chewed tobacco, smoked a pipe, smoked self-rolled cigarettes, or smoked cigarettes/cigars? ()

(1) Yes
(2) No Skip to DA067 DA067

DA060. Which products did/do you normally use?

(1) Smoking a pipe （）
(2) Smoking self-rolled cigarettes
(3) Filtered cigarette
(4) Unfiltered cigarette
(5) Cigar
(6) Water cigarettes

DA061. Do you still have the habit or have you totally quit?

(1) Still have Skip DA062 DA062
(2) Quit

DA062 . At what age did you totally quit smoking?

Age______1…120 (DA062_1) years Or _____1900..2011 (DA062_2) Year

[IWER: Mark the year using four digits. : 4]

If DA060 =3 or 4 askDA063 -DA064. DA060 =34DA063 -DA064
DA063. In one day about how many cigarettes do/did you consume [preload:now/before totally quitting]? [/]________ cigarettes

If DA060=3 or 4 ask DA064 . DA060=34DA064
DA064. How much does/did it cost per pack = 20 cigarettes? [/](20) _____Yuan

[IWER: Prompt R: were asking price at that time, not current price][]

DA065. At what age did you start to smoke on a regular basis?
Age______ 1…120 (DA065_1) years Or _____1900..2011 (DA065_2) Year
[IWER: Mark the year using four digits. : 4]

DA066. How soon after you wake up did/do you smoke your first cigarette, cigar, or pipe? [/]
(1) Within 5 minutes 5
(2) Within 6-30 minutes 6 - 30
(3) Within 31-60 minutes 31 - 60
(4) More than 1 hour 1

DA067. Did you drink any alcoholic beverages, such as beer, wine, or liquor in the past year? How often?
(1) Drink more than once a month.
(2) Drink but less than once a month Skip to DA069 DA069
(3) None of these Skip to DA069 DA069

DA068. What type of alcoholic beverages did you drink? Liquor, wine, or beer(code all that apply) （）
(1) Liqor, including white liquor, whisky, and others Skip to DA071 DA071
(2) Beer Skip to DA071 DA071
(3) Wine or rice wine Skip to DA071 DA071

DA069. Did you ever drink alcoholic beverages in the past? How often?
(1) I never had a drink. →Skip to DA079 DA079
(2) I used to drink less than once a month. →Skip to DA079 DA079
(3) I used to drink more than once a month.

DA070. When did you quit or reduce drinking?
___________1900..2011 (DA070 _1)Year
[IWER: Record year in 4 digits. 4]
Or age : __________1…120 (DA070_2) Years

DA071. When did you start drinking?
___________1900..2011 (DA071_1)Year
[IWER: Record year in 4 digits. 4]
Or age : __________1…120 (DA071_2) Years

SKIP PATTERN CHECKPOINT: DRINKING

Now, I am going to ask you how often and how much you drank during the past year. Please tell me how often you drank per month, and how much you drank at a time on average. I will repeat the questions for different types of alcoholic beverages.

[CAPI: If DA068 answered 1, ask DA072 :] [DA068 1DA072 .]
DA072. How often did you drink liquor, including white liquor, whisky, and others per month in the last year
(1) Once a month
(2) 2-3 times a month 2-3
(3) Once a week
(4) 2-3 times a week 2-3
(5) 4-6 times a week 4-6
(6) Once a day
(7) Twice a day

(8) More than twice a day

DA073. The last time you drank liquor last year, how many liang of liquor did you drink? (1 liang=50cc/50 ml)（1=50）______ liang

[CAPI: If DA068=2, ask DA074 :] [DA068 2DA074 .]
DA074. How many times per month did you drink beer in the last year?
(1) Once a month
(2) 2-3 times a month 2-3
(3) Once a week
(4) 2-3 times a week 2-3
(5) 4-6 times a week 4-6
(6) Once a day
(7) Twice a day
(8) More than twice a day

DA075. The last time you drank beer last year, how many bottles of beer did you drink? (1bottle=2.5 mugs, 1mug=220cc) ?（1=2.51=220）______ (DA075_1)0..120bottles or _______(DA075_2)0..300mugs

[CAPI: If DA068 answered 3, ask DA076:] [DA068 3DA076.]
DA076. How often did you drink wine or rice wine per month in the last year?
(1) Once a month
(2) 2-3 times a month 2-3
(3) Once a week
(4) 2-3 times a week 2-3
(5) 4-6 times a week 4-6
(6) Once a day
(7) Twice a day
(8) More than twice a day

DA077. The last time you drank it last year, how many liang of wine did you drink? (1 liang=50cc) （1=50）________ 0.00..100.00liang

DA078. In the last year, have you ever taken a drink first thing in the morning to steady your nerves or get rid of a hangover?
(1) Yes
(2) No

DA079. How would you rate your health status? Would you say your health is very good, good, fair, poor or very poor?
(1) Very good
(2) Good
(3) Fair
(4) Poor
(5) Very poor

DA080. Next I have some questions about your health. Would you say your health is excellent, very good, good, fair, or poor?
[IWERinterviewer should read all the following options.]
(1) Excellent
(2) Very good
(3) Good

(4) Fair
(5) Poor

[IWER: Please do not ask proxy the following question DA079 and DA080 . DA079 and DA080]

**INTERVIEWER CHECK AGE OF RESPONDENT?**	**1<** 65 **YEAR➔COLUMN A A** **6**85**–** 89**YEAR➔ COLUMN** F F<br>**2**65 **–** 69 **YEAR➔COLUMN B B** **7**90**–** 94**YEAR➔ COLUMN** G G<br>370 **–** 74 **YEAR➔COLUMN C C** **9**95**–** 99**YEAR➔ COLUMN** H H<br>**4**75 **–** 79 **YEAR➔ COLUMN D D** **10>=** 100 **YEAR➔ COLUMN** I I<br>**5**80**–** 84**YEAR➔ COLUMN E E**

	A	B	C	D	E	F	G	H	I
AGE	75 years	80 years	85 years	90 years	95 years	100 years	105 years	110 years	115 years
**DA 081 .** **Suppose there are 5 steps, where the lowest step represents the smallest chance and the highest step represents the highest chance, on what step do you think is your chance in reaching the age of [...]?**<br>1 Almost impossible<br>2 Not very likely<br>3 Maybe<br>4 Very likely<br>5 Almost certain	**1<br>2<br>3<br>4<br>5**	**1<br>2<br>3<br>4<br>5**	**1<br>2<br>3<br>4<br>5**	**1<br>2<br>3<br>4<br>5**	**1<br>2<br>3<br>4<br>5**	**1<br>2<br>3<br>4<br>5**	**1<br>2<br>3<br>4<br>5**	**1<br>2<br>3<br>4<br>5**	**1<br>2<br>3<br>4<br>5**

DB FUNCTIONAL LIMITATIONS AND HELPERS

[CAPI: If R is younger than 50(year of birth is after 1960) and If DA001 =1 or 2 or DA002 =1 or 2 and DA003 =2 and DA004 =2 and DA005 =. and DA007 =2 or DA008 =2 skip DB001 -DB015][50(1960)DB001-DB015]

We need to understand difficulties people may have with various activities because of a health or physical problem. Please tell me whether you have difficulty performing any of the following tasks on a regular basis. Exclude any difficulties that you expect to last less than three months. （）.

DB001. Do you have any difficulty with running or jogging about 1 Km? 1
- (1) No, I dont have any difficulty >> Skip to DB004 DB004
- (2) I have difficulty but can still do it.
- (3) Yes, I have difficulty and need help. ,
- (4) I can not do it.

DB002. Do you have difficulty …Walking 1 km…? 1
- (1) No, I dont have any difficulty >> skip DB003 DB003
- (2) I have difficulty but can still do it.
- (3) Yes, I have difficulty and need help. ,
- (4) I can not do it.

DB003. Do you have difficulty … Walking 100 metres…? 100
- (1) No, I dont have any difficulty
- (2) I have difficulty but can still do it.
- (3) Yes, I have difficulty and need help. ,
- (4) I can not do it.

DB004. Do you have difficulty …Getting up from a chair after sitting for a long period…?
- (1) No, I dont have any difficulty
- (2) I have difficulty but can still do it.
- (3) Yes, I have difficulty and need help. ,
- (4) I can not do it.

DB005. Do you have difficulty …Climbing several flights of stairs without resting…?
- (1) No, I dont have any difficulty
- (2) I have difficulty but can still do it.
- (3) Yes, I have difficulty and need help. ,
- (4) I can not do it.

DB006. Do you have difficulty …Stooping, kneeling, or crouching…?
- (1) No, I dont have any difficulty
- (2) I have difficulty but can still do it.
- (3) Yes, I have difficulty and need help. ,
- (4) I can not do it.

DB007. Do you have difficulty …Reaching or extending your arms above shoulder level…? (he/she is regarded as not having difficulty only if he/she can extend both of his/her arms, otherwise he/she is regarded as having difficulty.) （）
- (1) No, I dont have any difficulty
- (2) I have difficulty but can still do it.
- (3) Yes, I have difficulty and need help. ,

(4) I can not do it.

DB008. Do you have difficulty …Lifting or carrying weights over 10 jin, like a heavy bag of groceries…? 10

(1) No, I dont have any difficulty
(2) I have difficulty but can still do it.
(3) Yes, I have difficulty and need help. ,
(4) I can not do it.

DB009. Do you have difficulty …Picking up a small coin from a table…?

(1) No, I dont have any difficulty
(2) I have difficulty but can still do it.
(3) Yes, I have difficulty and need help. ,
(4) I can not do it.

[CAPI: IF (DB001=1&DB003 =1&…&DB009 =1), THEN SKIP TO DB016, DB001 ~DB009 DB016]

Here are a few more everyday acivities. Please tell me if you have any difficulties with these because of a physical, mental, emotional or memory problem. Again, exclude any that you expect to last less than three months.

DB010. Because of health and memory problems, do you have any difficulty with dressing? Dressing includes taking clothes out from a closet, putting them on, buttoning up, and fastening a belt.

(1) No, I dont have any difficulty
(2) I have difficulty but can still do it.
(3) Yes, I have difficulty and need help. ,
(4) I can not do it.

DB011. Because of health and memory problems, do you have any difficulty with bathing or showering?

(1) No, I dont have any difficulty
(2) I have difficulty but can still do it.
(3) Yes, I have difficulty and need help. ,
(4) I can not do it.

DB012. Because of health and memory problems, do you have any difficulty with eating, such as cutting up your food? (Definition: By eating, we mean eating food by oneself when it is ready.)

(1) No, I dont have any difficulty
(2) I have difficulty but can still do it.
(3) Yes, I have difficulty and need help. ,
(4) I can not do it.

DB013. Do you have any difficulty with getting into or out of bed?

(1) No, I dont have any difficulty
(2) I have difficulty but can still do it.
(3) Yes, I have difficulty and need help. ,
(4) I can not do it.

DB014. Because of health and memory problems, do you have any difficulties with using the toilet, including getting up and down?

(1) No, I dont have any difficulty
(2) I have difficulty but can still do it.
(3) Yes, I have difficulty and need help. ,
(4) I can not do it.

DB015. Because of health and memory problems, do you have any difficulties with controlling urination and

defecation? If you use a catheter (conduit) or a pouch by yourself, then you are not considered to have difficulties. （）

(1) No, I dont have any difficulty
(2) I have difficulty but can still do it.
(3) Yes, I have difficulty and need help. ,
(4) I can not do it.

DB016. Because of health and memory problems, do you have any difficulties with doing household chores? (Definition: By doing household chores, we mean house cleaning, doing dishes, making the bed, and arranging the house.)
[IWER: If R cannot mop the floor, but can scrub, or R cannot fold heavy bedding, but is able to do light ones, then mark (3). (3)]

(1) No, I dont have any difficulty
(2) I have difficulty but can still do it.
(3) Yes, I have difficulty and need help. ,
(4) I can not do it.

DB017. Because of health and memory problems, do you have any difficulties with preparing hot meals?
(Definition: By preparing hot meals, we mean preparing ingredients, cooking, and serving food.)
[IWER: If another person prepares ingredients or if R can cook rice, but is not able to prepare side dishes, then mark (3). （3）]

(1) No, I dont have any difficulty
(2) I have difficulty but can still do it.
(3) Yes, I have difficulty and need help. ,
(4) I can not do it.

DB018. Because of health and memory problems, do you have any difficulties with shopping for groceries? By shopping, we mean deciding what to buy and paying for it.

(1) No, I dont have any difficulty
(2) I have difficulty but can still do it.
(3) Yes, I have difficulty and need help. ,
(4) I can not do it.

DB019. Because of health and memory problems, do you have any difficulties with managing your money, such as paying your bills, keeping track of expenses, or managing assets?

(1) No, I dont have any difficulty
(2) I have difficulty but can still do it.
(3) Yes, I have difficulty and need help. ,
(4) I can not do it.

DB020. Because of health and memory problems, do you have any difficulties with taking medications? By taking medications, we mean taking the right portion of medication right on time.

(1) No, I dont have any difficulty
(2) I have difficulty but can still do it.
(3) Yes, I have difficulty and need help. ,
(4) I can not do it.

SKIP PATTERN CHECKPOINT: FUNCTIONAL DIFFICULTIES

IF R NEEDS ANY HELP IN DB010 ~DB020 (BOTH HELP TO SOME EXTENT, (3), AND IN EVERY RESPECT, (4)), SKIP TO DB021. DB010 ~DB020 （(3)(4)）DB021
IF R DOES NOT NEED ANY HELP, SKIP TO DB029 DB029

DB021. Do you use the following auxiliary? (Code all that apply)（）

(1) Walking stick
(2) Travel device
(3) Manual wheelchair
(4) Electric Wheelchair
(5) Not any

DB022. Who most often helps you with [make sure we ask this only once for ALL these activities; do not ask for each problem separately] (dressing, bathing, eating, getting out of bed, using the toilet, controlling urination and defecation, doing chores, preparing hot meals, shopping, managing money, making phone calls, taking medications) (May choose up to 3 persons)? (3)（）
(Select from the list displayed by CAPI) （CAPI）

1 Spouse
2 Mother
3 Father
4 Mother-in-law /
5 Father-in-law /
6 ~ 30 Children
31 Sibling
32 Sibling of spouse
33 Brother-in-law, sister-in-law ///
34 Spouse of child
35 Grandchild
36 Other relative
37 Paid helper(such as nanny) （）
38 Volunteer or Employee of facility
39 Other
40 No one helped →Skip to DB029 DB029

PROCEDURE: For each helper 6-30 or 34 chosen in DB022, ask DB022_a. DB022 6-3034（）DB022_a

DB022_a. Whether it is the [helpers name chosen from DB022] him/herself taking the time? [DB022]
（1） Yes
（2） No

PROCEDURE: For each helper 1-40 chosen in DB022, ask DB023-DB026 DB022 1-40 DB023 -DB026

DB023. During the last month, on about how many days did [helpers name chosen from DB022] help you? [DB022]_____ 1..31Days

DB024. On the days [helpers name chosen from DB022 helps you, about how many hours per day is that? [DB022] / _____ 1..24Hours [IWER: less than an hour=1 1］

DB025. Is he/she living in your home? /
（3） Yes
（4） No

DB026. Is [helpers name chosen from DB022 paid to help you? [DB022]]
(1) Yes
(2) No

SKIP PATTERN CHECKPOINT: PAID HELPER
IF R PAID FOR HELP IN DB026, SKIP TO DB027. DB026 DB027 .

IF R DID NOT PAY FOR HELP, SKIP TO DB029. DB029 .

DB027. About how much in total did you pay (including value of the goods you gave them as repayment for their help) for the help during the past month? （） _______ Yuan

DB028. Who contributed most to paying this cost? Please choose one person who paid the most. (Select from the list displayed by CAPI) （CAPI）

1 Yourself
2 Spouse
3 Mother
4 Father
5 Mother-in-law /
6 Father-in-law /
7 ~ 31 Children
32 Sibling
33 Sibling of spouse
34 Brother-in-law, sister-in-law ///
35 Spouse of child
36 Grandchild
37 Other relative
38 Other

DB029. Do you use the following auxiliary? (Code all that apply)（）

⑴ Walking stick
⑵ Travel device
⑶ Manual wheelchair
⑷ Electric Wheelchair
⑸ catheter, urine collection bag
⑹ Toilet Series
⑺ None of the above

DB030. Suppose that in the future, you needed help with basic daily activities like eating or dressing. Do you have relatives or friends (besides your spouse/partner) who would be willing and able to help you over a long period of time? （）

(1) Yes
(2) No →Skip to DB032 DB032

DB031. What is the relationship to you of that person or those persons? （Choose all that apply） /（） (Select from the list displayed by CAPI) （CAPI）

1 Mother
2 Father
3 Mother-in-law /
4 Father-in-law /
5 ~ 29 Children
30 Sibling
31 Sibling of spouse
32 Brother-in-law, sister-in-law ///
33 Spouse of child
34 Grandchild
35 Other relative
36 Paid helper(such as nanny) （）
37 Volunteer or Employee of facility

38 Other

DB032. How often did the respondent receive assistance in answering this section ?
[IWER: If it is answered by a proxy, please record the respondents reaction.]

(1) Never
(2) A few times
(3) Most or all of the time
(4) The section was completed by a proxy respondent（the respondent is absent） →Skip to DB033
DB033

DB033. What is your relationship to R?
[IWER: What is the proxys relationship to R? If unknown, please ask the proxy.]

(1) Spouse
(2) Mother
(3) Father
(4) Mother-in-law /
(5) Father-in-law
(6) Sibling
(7) Brother-in-law, sister-in-law /
(8) Child
(9) Spouse of child
(10) Grandchild
(11) Other relative
(12) Helper or other non-relative

DB034. [IWER: Please record the reason for proxy]
What is the main reason for proxy (the respondent is absent)

（1）The respondent has serious physical handicaps _
（2）The respondent has serious mental handicaps
（3）The respondent has rejected this interview.
（4）Other____. (DB034_1)

DC COGNITION & DEPPRESSION

[IWER: If DB032 =4, then skip to Section E , health care and insurance. Sections Cc and Cd must not be answered by proxy respondents.] [DB032 =4E DC DE .]

DC001.. Now Im going to ask several simple questions. Some may be easy and some may be hard to answer. Please try to answer as honestly as you can. Are you ready? Please tell me todays date.（Check all that apply） （）
[IWER: R doesn't have to answer in this order. If R is an elderly person and marked the date by lunar calendar, that date is correct if it matches with the solar calendar. You can check the accuracy, using the converter.]

(1) Year is correct
(2) Month is correct
(3) Day is correct

DC002. Please tell me the day of the week. Is it Monday, Tuesday, Wednesday, Thursday, Friday, Saturday, or Sunday?

(1) Day of week OK/correct
(2) Day of week not OK/incorrect

DC003. What is the current season (among Spring, Summer, Fall, or Winter)?

(1) Season OK
(2) Season not OK

DC004. How would you rate your memory at the present time? Would you say it is excellent, very good, good, fair or poor?

(1) Excellent
(2) Very good
(3) Good
(4) Fair
(5) Poor

We are going to read a list consisting of 10 words and we would like you to memorize as many as you can. We deliberately made the list long to make it difficult for anyone to memorize all of the words; most people will only remember a few of them. Please listen carefully as we read the list because we cannot repeat it. When we finish reading the list, we will ask you to recall and tell us as many words as you can remember, and they dont have to be in the order that you heard them. Is this explanation clear?
[IWER: Do not allow proxy answers.] []

(CC006_Version)	**Randomly select a list of words to use.**			
(CC006_wordlis**DC006** **.**	**1. ADC006_A**	**2. BDC006_B**	**3. CCC006_C**	**4.DDC006_D**

Read the list slowly, with an interval of about 2 seconds between each word. 2

LIST A	LIST B	LIST C	LIST D
A01. RICE	B01. STOOL	C01. MOUNTAIN	D01. WATER
A02. RIVER	B02. FOOT	C02. STONE	D02. HOSPITAL
A03 DOCTOR	B03. SKY	C03. BLOOD	D03. TREE
A04. CLOTHES	B04.MONEY	C04. MOTHER	D04. FATHER
A05. EGG	B05. PILLOW	C05. SHOES	D05. FIRE

A06.CAT<br>A07. BOWL<br>A08. CHILD<br>A09. HAND<br>A10. BOOK	B06. DOG<br>B07. HOUSE<br>B08. WOOD<br>B09. SCHOOL<br>B10. TEA	C06. EYE<br>C07. GIRL<br>C08. HOUSE<br>C09. ROAD<br>C10. SUN	D06. TOOTH<br>D07. MOON<br>D08. VILLAGE<br>D09. BOY<br>D10. TABLE

Now please let us know the words you are able to recall. Give R enough time to recall, approximately up to 2 minutes. 2

(CC006) **DC007** . **Circle all the words mentioned by the R on the column.**

LIST A	LIST B	LIST C	LIST D
A01. RICE DC007_A_1	B01. STOOL DC007_B_1	C01. MOUNTAIN DC007_C_1	D01. WATER DC007_D_1
A02. RIVER DC007_A_2	B02. FOOT DC007_B_2	C02. STONE DC007_C_2	D02. HOSPITAL DC007_D_2
A03 DOCTOR DC007_A_3	B03. SKY DC007_B_3	C03. BLOOD DC007_C_3	D03. TREE DC007_D_3
A04. CLOTHES DC007_A_4	B04.MONEY DC007_B_4	C04. MOTHER DC007_C_4	D04. FATHER DC007_D_4
A05. EGG DC007_A_5	B05. PILLOW DC007_B_5	C05. SHOES DC007_C_5	D05. FIRE DC007_D_5
A06.CAT DC007_A_6	B06. DOG DC007_B_6	C06. EYE DC007_C_6	D06. TOOTH DC007_D_6
A07. BOWL DC007_A_7	B07. HOUSE DC007_B_7	C07. GIRL DC007_C_7	D07. MOON DC007_D_7
A08. CHILD DC007_A_8	B08. WOOD DC007_B_8	C08. HOUSE DC007_C_8	D08. VILLAGE DC007_D_8
A09. HAND DC007_A_9	B09. SCHOOL DC007_B_9	C09. ROAD DC007_C_9	D09. BOY DC007_D_9
A10. BOOK DC007_A_10	B10. TEA DC007_B_10	C10. SUN DC007_C_10	D10. TABLE DC007_D_10
**A96. NONE RECALLED**<br>**A97. REFUSE TO RECALL**	**B96. NONE RECALLED**<br>**B97. REFUSE TO RECALL**	**C96. NONE RECALLED**<br>**C97. REFUSE TO RECALL**	**D96.NONE RECALLED**<br>**D97. REFUSE TO RECALL**

DC008. [CAPI automatically record the current time: hour and minute.
CAPI___(DC008_1)_____(DC008_2)（24）]

Try to remember the words I just read to you. I'll ask you to recall them later.
[IWER: Read once more if R did not recall any of the words, up to 3 times, and then go on. If R does not recall any of the words,assure them that it is OK so that R will feel comfortable/at ease.] [13]

The 10 items below refer to how you have felt and behaved during the last week. Choose the appropriate response. 1012

[Show Card 12]
DC009. I was bothered by things that don't usually bother me.
(1) Rarely or none of the time (<1 day) （<1）
(2) Some or a little of the time (1-2 days) （1-2）

(3) Occasionally or a moderate amount of the time (3-4 days) （3-4）
(4) Most or all of the time (5-7 days) (5-7)

DC010. I had trouble keeping my mind on what I was doing.
(1) Rarely or none of the time (<1 day) （<1）
(2) Some or a little of the time (1-2 days) （1-2）
(3) Occasionally or a moderate amount of the time (3-4 days) （3-4）
(4) Most or all of the time (5-7 days) （5-7）

DC011. I felt depressed.
(1) Rarely or none of the time (<1 day) （<1）
(2) Some or a little of the time (1-2 days) （1-2）
(3) Occasionally or a moderate amount of the time (3-4 days) （3-4）
(4) Most or all of the time (5-7 days) （5-7）

DC012. I felt everything I did was an effort.
(1) Rarely or none of the time (<1 day) （<1）
(2) Some or a little of the time (1-2 days) （1-2）
(3) Occasionally or a moderate amount of the time (3-4 days) （3-4）
(4) Most or all of the time (5-7 days) （5-7）

DC013. I felt hopeful about the future.
(1) Rarely or none of the time (<1 day) （<1）
(2) Some or a little of the time (1-2 days) （1-2）
(3) Occasionally or a moderate amount of the time (3-4 days) （3-4）
(4) Most or all of the time (5-7 days) （5-7）

DC014. I felt fearful.
(1) Rarely or none of the time (<1 day) （<1）
(2) Some or a little of the time (1-2 days) （1-2）
(3) Occasionally or a moderate amount of the time (3-4 days) （3-4）
(4) Most or all of the time (5-7 days) （5-7）

DC015. My sleep was restless.
(1) Rarely or none of the time (<1 day) （<1）
(2) Some or a little of the time (1-2 days) （1-2）
(3) Occasionally or a moderate amount of the time (3-4 days) （3-4）
(4) Most or all of the time (5-7 days) （5-7）

DC016. I was happy.
(1) Rarely or none of the time (<1 day) （<1）
(2) Some or a little of the time (1-2 days) （1-2）
(3) Occasionally or a moderate amount of the time (3-4 days) （3-4）
(4) Most or all of the time (5-7 days) （5-7）

DC017. I felt lonely.
(1) Rarely or none of the time (<1 day) （<1）
(2) Some or a little of the time (1-2 days) （1-2）
(3) Occasionally or a moderate amount of the time (3-4 days) （3-4）
(4) Most or all of the time (5-7 days) （5-7）

DC018. I could not get "going."
(1) Rarely or none of the time (<1 day) （<1）
(2) Some or a little of the time (1-2 days) （1-2）

(3) Occasionally or a moderate amount of the time (3-4 days) （3-4）
(4) Most or all of the time (5-7 days) （5-7）

[IWER: Try to persuade R to answer if R refuses at first. Record the exact number R says.] []
DC019. Let's try some subtraction of numbers this time. What does 100 minus 7 equal?
1007______________

DC020. And 7 from that? 7____________

DC021. And 7 from that? 7____________

DC022. And 7 from that? 7____________

DC023. And 7 from that? 7____________

DC024. [IWER: Please indicate whether the respondent used paper and pencil or any other aid whe n completing the number subtraction.] []
(1) used aid
(2) did not use aid

[Show Card 32]
DC025. Do you see this picture? Please draw that picture on this paper.
[IWER: Show the picture of two pentagons overlapped.] []
(1) Drew the picture
(2) Failed to draw the picture

DC026. [CAPI automatically record the current time: hour and minute. ___(DC026_1)_____(DC026_2)（24）]

DC027. A little while ago, I read you a list of words and you repeated the ones you could remember. Please tell me any of the words that you remember now.
[IWER: Answers are displayed only for interviewer. Please do not show the screen to R.] []

Circle all the words mentioned by R in the column and list all of the words mentioned which are not on the list in row 51-55.

LIST A	LIST B	LIST C	LIST D
A01. RICE DC027_A_1	B01. STOOL DC027_B_1	C01. MOUNTAIN DC027_C_1	D01. WATER DC027_D_1
A02. RIVER DC027_A_2	B02. FOOT DC027_B_2	C02. STONE DC027_C_2	D02. HOSPITAL DC027_D_2
A03 DOCTOR DC027_A_3	B03. SKY DC027_B_3	C03. BLOOD DC027_C_3	D03. TREE DC027_D_3
A04. CLOTHES DC027_A_4	B04.MONEY DC027_B_4	C04. MOTHER DC027_C_4	D04. FATHER DC027_D_4
A05. EGG DC027_A_5	B05. PILLOW DC027_B_5	C05. SHOES DC027_C_5	D05. FIRE DC027_D_5
A06.CAT DC027_A_6	B06. DOG DC027_B_6	C06. EYE DC027_C_6	D06. TOOTH DC027_D_6
A07. BOWL DC027_A_7	B07. HOUSE DC027_B_7	C07. GIRL DC027_C_7	D07. MOON DC027_D_7
A08. CHILD DC027_A_8	B08. WOOD DC027_B_8	C08. HOUSE DC027_C_8	D08. VILLAGE DC027_D_8
A09. HAND DC027_A_9	B09. SCHOOL DC027_B_9	C09. ROAD DC027_C_9	D09. BOY DC027_D_9

A10. BOOK DC027_A_10	B10. TEA DC027_B_10	C10. SUN DC027_C_10	D10. TABLE DC027_D_10
**A96. NONE RECALLED**<br>**A97. REFUSE TO RECALL**	**B96. NONE RECALLED**<br>**B97. REFUSE TO RECALL**	**C96. NONE RECALLED**<br>**C97. REFUSE TO RECALL**	**D96. NONE RECALLED**<br>**D97. REFUSE TO RECALL**

DC028. Please think about your life-as-a-whole. How satisfied are you with it? Are you completely satisfied, very satisfied, somewhat satisfied, not very satisfied, or not at all satisfied?
（1）Completely satisfied
（2）Very satisfied
（3）Somewhat satisfied
（4）Not very satisfied
（5）Not at all satisfied

DE SELF-REPORTED HEALTH &VIGNETTES

[IWER: Sections DE must NOT be answered by proxy respondents. DE]
Vignettes will be presented ONLY to a random subsample of households (half). All selected Rs will answer the first 6 questions, and will then get 2 randomly-selected domains out of the 6 domains (each domain has 3 questions). All eligible members in the selected households get the same 12 questions. Do NOT allow proxy answers. 662（）12

We would now like to ask you some questions about your health. Every subject of the options are the same, as shown in card 13, including none, mild, moderate, severe and extreme, please select the appropriate answer. Please choose one of the five answers for every question.13

[Show Card 13]

DE001. Overall in the last month, how much bodily aches or pains did you experience?
(1) None
(2) Mild
(3) Moderate
(4) Severe
(5) Extreme

DE002. In the last month, how much difficulty did you have with sleeping, such as having trouble falling asleep, waking up frequently during the night, or waking up too early in the morning?
(1) None
(2) Mild
(3) Moderate
(4) Severe
(5) Extreme

DE003. Overall in the last month, how much of a problem did you have with moving around?
(1) None
(2) Mild
(3) Moderate

(4) Severe
(5) Extreme

DE004. Overall in the last month, how much difficulty did you have with concentrating or remembering things?

(1) None
(2) Mild
(3) Moderate
(4) Severe
(5) Extreme

DE005. In the last month, how much of a problem did you have with shortness of breath?

(1) None
(2) Mild
(3) Moderate
(4) Severe
(5) Extreme

DE006. Overall in the last month, how much of a problem did you have with feeling sad, low, or depressed?

(1) None
(2) Mild
(3) Moderate
(4) Severe
(5) Extreme

We will now give you some examples of persons with serious and less serious health problems. We would like to know how you evaluate the health of these persons. Please assume that the persons have the same age and background as you.

[PROCEDURE: In DE007 -DE024 , there are 2 first names: one male and one female. The program should randomly choose which gender is used for each question, but use the same gender in a question for all persons within a HH. So if domains pain and sleep are chosen for HH A, then for each of the 6 questions for the 2 domains, 1 gender is randomly selected. That choice is used for the main respondent. DE007 -DE024 A6]

Pain Domain

DE007. Zhang Jun/Wang Hong has a headache once a month that is relieved after taking a pill. During the headache he/she can carry on with his/her day-to-day affairs. Overall in the last month, how much of bodily aches or pains did Zhang San/Wang Hong have? / / /

(1) None
(2) Mild
(3) Moderate
(4) Severe
(5) Extreme

DE008. Zhou Wei/Li Li has pain that radiates down his/her right arm and wrist during his/her day at work. This is slightly relieved in the evenings when he/she is no longer working on his/her computer. Overall in the last month, how much of bodily aches or pains did Zhou Wei/Li Li have? / / /

(1) None
(2) Mild
(3) Moderate
(4) Severe
(5) Extreme

DE009. Zhao Liang/Zhou Yan has pain in his/her knees, elbows, wrists, and fingers, and the pain is present almost all the time. Although medication helps, he/she feels uncomfortable when moving around and holding and lifting things. Overall in the last month, how much of bodily aches or pains did Zhao Liang/Zhou

Yan have? / / /

(1) None
(2) Mild
(3) Moderate
(4) Severe
(5) Extreme

Sleep Domain

DE010. Zhao Yong/Wang Hua falls asleep easily at night, but two nights a week he/she wakes up in the middle of the night and cannot go back to sleep for the rest of the night. In the last month, how much difficulty did Zhao Yong/Wang Hua have with sleeping, such as falling asleep, waking up frequently during the night, or waking up too early in the morning? / /

(1) None
(2) Mild
(3) Moderate
(4) Severe
(5) Extreme

DE011. Li Gang/Li Hong takes about two hours every night to fall asleep. He/She wakes up once or twice a night feeling panicked and takes more than one hour to fall asleep again. In the last month, how much difficulty did Li Gan/Li Hong have with sleeping, such as falling asleep, waking up frequently during the night, or waking up too early in the morning? / /

(1) None
(2) Mild
(3) Moderate
(4) Severe
(5) Extreme

DE012. Wang Qiang/Xu Lin wakes up almost once every hour during the night. When he/she wakes up in the night, it takes around 15 minutes for him/her to go back to sleep. In the morning he/she does not feel well-rested. In the last month, how much difficulty did Wang Qiang/Xu Lin have with sleeping, such as falling asleep, waking up frequently during the night, or waking up too early in the morning? / 15 / /

(1) None
(2) Mild
(3) Moderate
(4) Severe
(5) Extreme

Mobility Domain

DE013. Luo Hai/Hong Mei is able to walk distances of up to 200 meters without any problems but feels tired after walking one kilometer or climbing more than one flight of stairs. He/She has no problems with day-to-day activities, such as carrying food from the market. Overall in the last month, how much of a problem did Luo Hai/Hong Mei have with moving around? / 200 / /

(1) None
(2) Mild
(3) Moderate
(4) Severe
(5) Extreme

DE014. Chen Shi/Chen Hong does not exercise. He/She cannot climb stairs or do other physical activities because he/she is obese. He/She is able to carry the groceries and do some light household work. Overall in the last month, how much of a problem did Chen Shi/Chen Hong have with moving around? / / /

(1) None
(2) Mild
(3) Moderate

(4) Severe
(5) Extreme

DE015. Li Bin/Zhang Lan has a lot of swelling in his/her legs due to his/her health condition. He/She has to make an effort to walk around his/her home as his/her legs feel heavy. Overall in the last month, how much of a problem did Li Bin/Zhang Lan have with moving around? / /

(1) None
(2) Mild
(3) Moderate
(4) Severe
(5) Extreme

Cognition Domain

DE016. Zhang Liang/Liu Jia can concentrate while watching TV, reading a magazine, or playing a game of cards or chess. Once a week he/she forgets where his/her keys or glasses are, but finds them within five minutes. Overall in the last month, how much difficulty did Zhang Liang/Liu Jia have with concentrating or remembering things? / 5 /

(1) None
(2) Mild
(3) Moderate
(4) Severe
(5) Extreme

DE017. Liu Jun/Song Li is keen to learn new recipes but finds that he/she often makes mistakes and has to reread a recipe several times before he/she is able to do make a new dish properly. Overall in the last month, how much difficulty did Liu Jun/Song Li have with concentrating and remembering things? / / /

(1) None
(2) Mild
(3) Moderate
(4) Severe
(5) Extreme

DE018. Li Wei/Yang Wen cannot concentrate for more than 15 minutes and has difficulty paying attention to what is being said to him/her. Whenever he/she starts a task, he/she never manages to finish it and often forgets what he/she was doing. He/She is able to learn the names of people he/she meets. Overall in the last month, how much difficulty did Li Wei/Yang Wen have with concentrating or remembering things? / 15/ / / /

(1) None
(2) Mild
(3) Moderate
(4) Severe
(5) Extreme

Breathing Domain

DE019. Zhou Yang/Huang Rong has no problems with walking slowly. He/She gets out of breath easily when climbing uphill for 20 meters or a flight of stairs. In the last month, how much of a problem did Zhou Yang /Huang Rong have with shortness of breath? / 20 /

(1) None
(2) Mild
(3) Moderate
(4) Severe
(5) Extreme

DE020.. Peng Lai/Sun Yue suffers from respiratory infections about once every year. He/She is short of breath 3 or 4 times a week and had to be admitted to the hospital twice in the past month with a bad cough that required treatment with antibiotics. In the last month, how much of a problem did Peng Lai/Sun Yue

have with shortness of breath? / / / /

(1) None
(2) Mild
(3) Moderate
(4) Severe
(5) Extreme

DE021. He Ming/Zhang Fang has been a heavy smoker for 30 years and wakes up with a cough every morning. He/She gets short of breath even while resting and does not leave the house anymore. He/She often needs to be put on oxygen. In the last month, how much of a problem did He Ming/Zhang Fang have with shortness of breath? / 30 / /

(1) None
(2) Mild
(3) Moderate
(4) Severe
(5) Extreme

Affect Domain

DE022. Wang Dong/Tang Jing enjoys his/her work and social activities and is generally satisfied with his/her life. He/She gets depressed every 3 weeks for a day or two and loses interest in what he/she usually enjoys but is able to carry on with his/her day-today activities. Overall in the last month, how much of a problem did Wang Dong/Tang Jing have with feeling sad, low, or depressed? / / / / /

(1) None
(2) Mild
(3) Moderate
(4) Severe
(5) Extreme

DE023. Li Feng/Zhang Yan feels nervous and anxious. He/She worries and thinks negatively about the future, but feels better in the company of people or when doing something that really interests him/her. When he/she is alone he/she tends to feel useless and empty. Overall in the last month, how much of a problem did Li Feng/Zhang Yan have with feeling sad, low, or depressed? / / / /

(1) None
(2) Mild
(3) Moderate
(4) Severe
(5) Extreme

DE024. Zheng Bo/Wu Na feels depressed most of the time. He/She weeps frequently and feels hopeless about the future. He/She feels that he/she has become a burden on others and that he/she would be better dead. Overall in the last month, how much of a problem did Zheng Bo/Wu Na have with feeling sad, low, or depressed? / / / /

(1) None
(2) Mild
(3) Moderate
(4) Severe
(5) Extreme

E HEALTH CARE AND INSURANCE

CARD21: Health Facilities

(1) General hospital （）
(2) Specialized hospital （）
(3) Chinese medicine hospital
(4) Community healthcare center
(5) Township hospital
(6) Health care post
(7) Village clinic/Private clinic /
(8) Other

CARD20: Health Insurance

(1) Urban employee medical insurance (yi-bao) （）
(2) Urban resident medical insurance
(3) New cooperative medical insurance (he-zuo-yi-liao) （）
(4) Urban and rural resident medical insurance （）
(5) Government medical insurance (gong-fei)
(6) Medical aid
(7) Private medical Insurance: Purchased by Rs union :
(8) Private medical Insurance: Purchased by Individual :
(9) Other medical insurance (specify) （）
(10) No insurance

CARD23: Health Facilities for Inpatient Care

(1) General Hospital （）
(2) Specialized hospital （）
(3) Chinese Medicine Hospital
(4) Community Healthcare Center
(5) Township Hospital
(6) Health care post
(7) Other

PART I MEDICAL INSURANCE

Now we would like to know about health insurance or benefits that you might have.

[Show Card 14]

EA001. Are you the policy holder/primary beneficiary of any of the types of health insurance listed below? (circle all that apply) ()

(1) Urban employee medical insurance (yi-bao) ()
(2) Urban resident medical insurance
(3) New cooperative medical insurance (he-zuo-yi-liao) （）
(4) Urban and rural resident medical insurance （）
(5) Government medical insurance (gong-fei)
(6) Medical aid
(7) Private medical Insurance: Purchased by Rs union :
(8) Private medical Insurance: Purchased by Individual :
(9)Other medical insurance (specify) , _______ (EA001_1)
(10)No insurance

[Soft check: If pick 10, cannot pick any other, you chose no insurance and a specific type of insurance, this is not possible 10, ,]

F1（1）
（2）2007779（）
（3）
（4）
（5）
（6）
（7）
（8）
（9）
（10）
（11）

For each circled type of insurance (1-11), ask the following questions EA002 --- EA008 .
1-11 EA002 --- EA008

EA002. Do you have supplemental insurance to this plan? （）
（1） Yes
（2） No
F1

EA003. Where did you set up your insurance account/policy? ?
(1)This county /
(2) (if it is not in this county) the place of your HuKou (/）
(3) other_______(EA003_1)province_________(EA003_2)county/

EA004. Method of reimbursement
(1) Get reimbursement immediately
(2) You pay first and get reimbursed later.

EA005. Through which agency did you purchase your primary plan?
(1) Community committee/ village committee /
(2) Rs union
(3) Agency of Social insurance
(4) Private insurance company
(5) Other

EA006. Whats your out-of-pocket yearly premium? ()____Yuan [soft check upper limit: 15,000 for choice 1, 3,000 choice 2, 1,000 choice 3, 1,500 choice 4, 4,000 choice 7, 2,500 choice 9 115,00023,00031,00041,50074,00092,500]

EA007. Who pay the premium for you? (choose all that apply) ()
(1) Myself
(2) Children
(3) Relatives
(4) Government
(5) Rs union （）
(6) Loan
(7) Donate
(8) Others Specify (EA007_1)

EA008. When did this benefit begin?

[IWER: Mark the year using four digits. Take down the month as its actual number. For example, write January as 1 not 01, December as 12. If do not remember month, fill 0. : 41101,12120]
1900..2011Year (EA008_1) └─┴─┴─┴─┘ 0..12Month (EA008_2) └─┴─┘

Skip to EC001 EC001

PART II HEALTH CARE COSTS AND UTILIZATION

[IWER: Please do not allow proxy to answer Part II.]

EC001. When did you take the last physical examination?
[IWER: Mark the year using four digits. Take down the month as its actual number. For example, write January as 1 not 01, December as 12. If do not remember month, fill 0. : 41101,12120]
1900..2011Year (EC001_1) └─┴─┴─┴─┘ 0..12Month (EC001_2) └─┴─┘

EC002. Who paied the physical examination cost?
(1) Myself
(2) Children
(3) Relatives
(4) Government
(5) Rs union （）
(6) Rs insurance
(7) Loan
(8) Donate
(9) Others ____ Specify ____(EC002_1)

The next questions pertain to medical facilities or medical providers you may have visited for outpatient care during the past 1 month (excluding hospitalization). （）

ED001. In the last month have you visited a public hospital, private hospital, public health center, clinic, or health workers or doctors practice, or been visited by a health worker or doctor for outpatient care?
(1) Yes
(2) No

ED002. Have you been ill in the last month?
(1) Yes
(2) No

If ED001 =2 and ED002 =2 skip to EE001 ED001 =2 ED002 =2EE001
If ED001 =1 skip to ED004 ED001 =1ED004
If ED002 =1 and ED001=2 go to ED003. ED002=1ED001 =2ED003

ED003. Whats the main reason for not seeking medical treatment?
(1) Already under treatment.
(2) Illness is not serious. Dont need treatment
(3) Poor
(4) No time
(5) Inconvenient traffic
(6) Poor service
(7) No available treatment
(8) Other

Skip to EE003 EE003

[Show Card 15]
ED004. Which types of medical facilities have you visited in the last 4 weeks for outpatient treatment?（circle

all that apply）()

(1) General hospital ()
(2) Specialized hospital ()
(3) Chinese medicine hospital
(4) Community healthcare center
(5) Township hospital
(6) Health care post
(7) Village clinic/Private clinic /
(8) Other

PROCEDURE: For each item 1-7 checked in ED004 , ask ED005ED004 1-7 ED005
ED005. How many times did you visit / been visited by [...] during the last month? └─┴─┘ Times

If sum(ED005)>1, then ask ED006; otherwise, skip ED006ED005>1, ED006ED006

ED006. How much did all the visits to [ED004 answer] cost during the last month? [ED004 answer]?

IWER: If possible, please check the list of cost.

A. 1. Total cost _____(ED006_1)Yuan ; [soft check upper bound: 30,000 30,000]

2. Didnt pay anything

PROCEDURE: IF ED006 = 1, ASK ED007

ED007.
B. 1. Self-paid part _____ (ED007_1)Yuan
2. Didnt pay anything.

Now Id like to ask you some questions about your most recent visit to a health care provider in the last month.

ED008. Which health care provider did you visit most recently during the past month?
[CAPIPreload the health care providers in ED004. ED004]

ED009. Is this facility public or private?
(1) Public
(2) Private

If ED008 =1-3, ask ED010 ED008 =1-3ED010
ED010. Whats the level of this facility?
(1) County/district //
(2) Regional /city /
(3) Provincial/ affiliated to a ministry /
(4) Military
(5) Others
(6) Not applicable

ED011. What is the name of this health care provider? ______
[IWER: If unknown, please write unknown.]

ED012. Did the provider visit you at home?
（1） Yes → skip ED013 to ED016 and ED022ED013 ED016 ED022
（2） No

ED013. How many kilometers is it from the medical facility to your residence? └─┴─┴─┘ . └─┴─┘ Km
[softcheck upper bound: 200 200]

ED014. What is the travel time (one-way) to that facility?

└─┴─┘ (ED014_1)
01. Minute [softcheck range:1-59 1-59]
02. Hour [softcheck upper limit: 10 10]

(ED014_2)
1 Walk
2 Bus
3 Car
4.Ambulance
5 Bicycle or other manual vehicles
6.Electric bicycle/electric tricycle /
7. Motorcycle
8. Tractor
9. Train
10. Animal or animal-pulled cart

If ED014 =1, skip ED015 ED014 =1ED015
ED015. What was the total transportation cost to the facility (including fuel cost, one way trip)? （）
└─┴─┴─┘ , └─┴─┴─┘ RMB [softcheck upper limit: 600 600]

ED016. Where is the health care provider located?
1. province (ED016_1)
   A. This province
   B. Other province, (preload province) （(ED016_1_1)

[IWER: Choose from the list of provincessee appendix 2]
2. county/city/(ED016_2)
   A. This county / city /
   B. Other county / city, specify /, ______(ED016_2_1) city,______(ED016_2_2)county
3. township/district /(ED016_3)
   A. This township/district /
   B. Other township/district, specify /_______ (ED016_3_1) /
4. village/street /(ED016_4)
   A. This village/street /
   B. Other village/street, specify /_______ (ED016_4_1) /

ED017. What was the purpose of your visit? (circle all that apply) ()
(1) Immunization
(2) Consultation
(3) Medical check-up
(4) Treatment for Illness
(5) Other

If ED017 =4, then ask ED018 —ED021, ELSE ASK ED022.
ED017 4ED018 —ED021 ED022

ED018. Could you tell me the disease name? _________

ED019. Was the visit a first visit or a follow-up visit for the symptom?
（1） First

（2） Follow-up

ED020 . Was the visit for ordinary outpatient service or an emergency?
（1）
（2）

[Show Card 16]
ED021. What kind of treatment did you receive? (circle all that apply) ()
(1) Injection
(2) Laboratorium test
(3) Surgery
(4) X-ray, CT, B ultrasonic, MRI X-CTB
(5) Medications and purchase medical
(6) IV (Drip Infusion)
(7) Traditional treatment ,eg massage, acupuncture
(8) Other

ED022. Upon arrival, how long did you have to wait to be examined? └─┴─┘

01. Minute [range 1-59]

02. Hour [softcheck upper limit : 6]
(ED022_1)
ED023. What was the total cost of this visit, including both treatment and medication cost (includes prescriptions you received)? ()

1. └─┴─┘ , └─┴─┴─┘ , └─┴─┴─┘ RMB (ED023_1) [softcheck upper limit : 30,000]

2. There was no cost

PROCEDURE: IF ED023 = 1, ASK ED024
ED024. how much did you pay out of pocket, after reimbursement from insurance?
1. └─┴─┘ , └─┴─┴─┘ , └─┴─┴─┘ RMB (ED024_1) [soft check upper limit: 30,000, also ED025 <=ED023_1, else Pay out of pocket cannot be more than total cost 30,000ED025 <=ED023_1]
2. Did not pay anything Go to ED026 ED026

ED025. Who contribute most for paying the out-of-pocket cost?
(1) Myself
(2) Children
(3) Relatives
(4) Government
(5) Rs union （）
(6) Loan
(7) Donate
(8) Others Specify

ED026. What was the total medication cost for this visit, including prescriptions you received? ()
1.________ RMB (ED026_1) [soft check upper limit: 5,000, and must be no more than ED023_1. 5,000ED023_1]

2. Doctor did not write a prescription skip ED027 ED027
3. Didnt fill prescription skip ED027 ED027

ED027. How much will you eventually pay out of pocket for the medications from this visit, including prescriptions you received? ()
1.________ RMB (ED027_1) [softcheck upper limit: 5,000 and ED027_1 must be no more than ED026_1 and no more than ED023_1. 5,000 ED027_1 ED026_1ED023_1]

2. Didnt pay anything

[Show Card 14]

ED028. What insurance did you use or will you use?(circle all that apply) （）（）

(1) Urban employee medical insurance (yi-bao) （）
(2) Urban resident medical insurance
(3) New cooperative medical insurance (he-zuo-yi-liao) （）
(4) Urban and rural resident medical insurance （）
(5) Government medical insurance (gong-fei)
(6) Medical aid
(7) Private medical Insurance: Purchased by Rs union :
(8) Private medical Insurance: Purchased by Individual :
(9) Other medical insurance (specify) ,_______
(10) Reimbursed by Rs union
(11) No insurance
(12) Not revelent to me .

F1（1）
（2）2007779（）
（3）
（4）
（5）
（6）
（7）
（8）
（9）
（10）
（11）
（12）

ED029. Did you give any red envelopes to the doctors for this visit?
（1）Yes
（2）No

The following questions pertain to hospitalization (inpatient care) that you have had during the past year.

EE001. In the past year, did a doctor suggest that you needed inpatient care but you did not get hospitalized?
(1) Yes
(2) No Skip to EE003 EE003

EE002. Whats the main reason for not seeking hospitalization?
(1) Not enough money
(2) Not willing to go to the hospital
(3) Felt that hospital was unlikely to cure problem—hospital quality poor
(4) Felt that care was unlikely to cure the problem—problem too serious
(5) No ward available.
(6) Other

EE003. Have you received inpatient care in the past year?
(1) Yes
(2) No Skip to EF001 EF001

EE004. How many times have you received inpatient care during the past year? |__|__| Times [If EE004 =1, skip to EE007. EE004 =1EE007]

EE005. What was the medical cost for all the hospitalizations you received during the past year? (Only include fees paid to the hospital, including ward fees but excluding wages paid to a hired nurse, transportation costs, and accommodation costs for yourself or family members.) ? （）

A. 1. Total cost _________(EE005_1)Yuan ;

2. Didnt pay anything. [softcheck upper limit : 300,000]

PROCEDURE: IF EE005 = 1 ASK EE006

EE006.

B. 1. Self-paid part _________(EE006_1)Yuan

2. Didnt pay anything. [softcheck upper limit: 300,000 and EE005 B.1<=EE005 A.1.300,000EE005 B.1<=EE005 A.1.]

We want details about the last hospitalization you had in the past year.

If ED001 = 1 ask EE007. ED001 = 1EE007.

EE007. Is this the same facility as mentioned in ED011 for outpatient care? [ED011]

(1) Yes Skip to EE016 EE016

(2) No

If EE007= 2 or EC003= 2 ask EE008 -EE015. EE007 = 2 EC003 = 2 EE008-EE015

[Show Card 17]

EE008. What is the type of health or service facility which you visited for last inpatient care (hospital admissions)/for your most recent hospitalization in the past year?

(1) General Hospital （）

(2) Specialized hospital （）

(3) Chinese Medicine Hospital

(4) Community Healthcare Center

(5) Township Hospital

(6) Health care post

(7) Other

EE009. Is this facility public or private?

(1) Public

(2) Private

If EE008 =1-3, ask EE010, EE008 =1-3EE010

EE010. Whats the level of this facility?

(1) County/district //

(2) Regional /city /

(3) Provincial/ affiliated to a ministry /

(4) Military

(5) Others

EE011. What is the name of this facility? ____________

EE012.. What is the location of this facility?

1. province (EE012_1)

A. This province

B. Other province, (preload province) （） (EE012_1_1)

[IWER: Choose from the list of provincessee appendix 2]

2. county/city/(EE012_2_1)
   A. This county / city /
   B. Other county / city, specify / ______/(EE012_2_2)
3. township/district /(EE012_3_1)
   A. This township/district /
   B. Other township/district, specify /_______ (EE012_3_2) /
4. village/street /(EE012_4_1)
   A. This village/street /
   B. Other village/street, specify /_______ (EE012_4_2)

EE013. How many kilometers is it from the medical facility to your residence? └─┴─┴─┘.└─┴─┘ Km
[softcheck upper limit : 3000]

EE014. What is the travel time (one-way) to that facility?
(EE014_1)
└─┴─┘

01. Minute [check range : 1-59]
02. Hour [softcheck upper limit : 20]

(EE014_2)
1 Walk
2 Bus
3 Car
4.Ambulance
5 Bicycle or other manual vehicles
6.Electric bicycle/electric tricycle /
7. Motorcycle
8. Tractor
9. Train
10. Animal or animal pulled cart

IF EE014_2 = 2-10, ask EE015
EE015. What was the total transportation cost to the facility (including fuel cost, one way trip)? （）
└─┴─┴─┘, └─┴─┴─┘ RMB [soft check upper limit : 600]

EE016. How many nights were you hospitalized there?
└─┴─┴─┘ Nights [softcheck upper limit : 40]

EE017. What was the starting date of your hospital stay?
______1900..2011_(EE017_1)Year _________0..12_(EE017_2)Month _______0..31_(EE017_3)Day
[IWER: Mark the year using four digits. Take down the month as its actual number. For example, write January as 1 not 01, December as 12. If do not remember month and day, fill 0. : 41101,12120]

EE018. What was your date of exit?
(1) ______1900..2011_(EE018_1)Year _________0..12_(EE018_2)Month ________0..31_(EE018_3)Day
[IWER: Mark the year using four digits. Take down the month as its actual number. For example, write January as 1 not 01, December as 12. If do not remember month and day, fill 0. : 41101,12120]
(2) Still there
[soft check: date of exit should be not before starting date, exit date is before starting date, please ask R again, also exit date should be within 1 year of today]

EE019.. Why were you hospitalized? （Choose one choice） ()
(1) Sickness
(2) Accident
(3) Violence
(4) Other

EE020 . Could you tell me the name of the disease? _____________

[Show Card 18]
EE021. During hospitalization, what kind of treatment did you receive? (circle all that apply) ()
[IWER: Read one by one.]
(1) Medical check-up/consultation
(2) Injection
(3) Laboratory test
(4) Surgery
(5) X-ray, CT, B ultrasonic, MRI X-CTB
(6) Medications
(7) IV (Drip Infusion)
(8) Traditional treatment, e.g., massage, acupuncture
(9) Delivery
(10) Other

[PROCEDURE: If EE018 =2, please skip EE022 . EE018 =2EE022]
EE022. Under what conditions did you leave the hospital?
(1) Fully recovered from illness, received doctors approval Skip EE023 EE023
(2) Didnt recover from illness, but received doctors suggestion to leave Skip EE023 EE023
(3) Didnt recover from illness, requested to leave without doctors suggestion
(4) Other reasons Skip EE023 EE023

EE023. Why did you want to leave the hospital before you were recovered?
(1) Cant recover from illness
(2) Poor
(3) No space in the hospital
（4）Limited hospital conditions
(5) Poor quality and service from health care providers
(6) Other reasons

EE024. What was the total medical cost of hospitalization? (Only include the fees paid to the hospital, excluding the wage of hired nurse, the fare or rent, but including the ward fees.) （）
1. └─┴─┴─┘,└─┴─┴─┘,└─┴─┴─┘ RMB (EE024_1)[softcheck upper limit : 100,000]
2. There was no cost

EE025. What was the total cost for hired nurse? ?
1. └─┴─┴─┘,└─┴─┴─┘,└─┴─┴─┘ RMB (EE025_1)
2. There was no cost

EE026. What was the total cost for transportation, food and accommodation of patient and relatives? ?
1. └─┴─┴─┘,└─┴─┴─┘,└─┴─┴─┘ RMB (EP026_1)
2. There was no cost

EE027. How much did you or will you eventually pay out of pocket for the total costs of hospitalization? （）
1. └─┴─┴─┘ , └─┴─┴─┘ , └─┴─┴─┘ RMB (EE027_1) [softcheck upper limit: 100,000 and EE027_1 must be no more than EE024_1 100,000EE027_1EE024_1]
2. Didnt pay anything.

EE028. Who contributes most for paying the out-o- pocket cost?
(1) Myself
(2) Children
(3) Relatives
(4) Government
(5) Rs union （）
(6) Loan
(7) Donate
(8) Others Specify

EE029. What was the total medication cost during this visit?
1. └─┴─┴─┘ , └─┴─┴─┘ , └─┴─┴─┘ RMB (EE029_1) [softcheck upper limit: 60,000 and EE029_1 should be no more than EE024_160,000EE029_1 EE024_1]
2. Didnt receive Skip EE030 EE030

EE030. How much did you pay out of pocket for medication costs during this visit?
1. └─┴─┴─┘ , └─┴─┴─┘ , └─┴─┴─┘ RMB (EE030_1) [softcheck upper limit: 60,000 and EE030_1 should be no more than EE029_1 and EE024_1 60,000EE031_1 EE029_1 EE024_1]
2. Didnt pay anything.

[Show Card 14]
EE031. (Preload from EA001 or EB002) What insurance did you use or will you use? (circle all that apply)()
(1) Urban employee medical insu rance (yi-bao) （）
(2) Urban resident medical insurance
(3) New cooperative medical insurance (he-zuo-yi-liao) （）
(4) Urban and rural resident medical insurance （）
(5) Government medical insurance (gong-fei)
(6) Medical aid
(7) Private medical Insurance: Purchased by Rs union :
(8) Private medical Insurance: Purchased by Individual :
(9) Other medical insurance (specify) , _______
(10) Reimbursed by Rs union
(11) No insurance
(12) Not revelent to me

F1（1）
（2）2007779（）
（3）
（4）
（5）
（6）
（7）
（8）
（8）
（10）
（11）
（12）

EE032. Did you pay any Red Envelopes to the doctors for this visit?
(1) Yes
(2) No .

Now wed like to know whether you have treated yourself during the past month.
[Show Card 19]
EF001. How did you treat yourself during the past month? (circle all that apply) ( ) ( ) [check, if choose 7 cannot choose other options 7]

(1) Consumed over-the-counter modern medicines
(2) Consumed prescription medicines
(3) Consumed traditional herbs or traditional medicines as treatment
(4) Tonic/Health supplement /
(5) Use health care equipment
(6) Other
(7) None Skip to EF006 EF006

For each circled self-treatment method (1-5), ask questions EF002 , EF003 , EF005 1-5 EF002 , EF003 , EF005
EF002. What is the approximate total cost to [preload EF001] during the last month? [preload EF001]
1. └─┘, └─┴─┴─┘, └─┴─┴─┘ RMB (EF002_1) [softcheck upper limits: 2,000 2,000]
2. There was no cost.

PROCEDURE: IF EF002 = 1 ASK EF003

EF003. How much did you pay out-of-pocket?

1. └─┘, └─┴─┴─┘, └─┴─┴─┘ RMB (EF003_1) [softchecks upper limits: 2,000 and EF003_1 should be no more than EF002_1 . 2,000EF003_1 EF002_1]

2. Didnt pay anything. Go to EF005 EF005

EF004. Who contribute most for paying the out-o- pocket cost?
(1) Myself
(2) Children
(3) Relatives
(4) Government
(5) Rs union ( )
(6) Loan
(7) Donate
(8) Others Specify

[Show Card 14]

EF005. What insurance did you use? (circle all that apply) ( )
(1) Urban employee medical insurance (yi-bao) ( )
(2) Urban resident medical insurance
(3) New cooperative medical insurance (he-zuo-yi-liao) ( )
(4) Urban and rural resident medical insurance ( )
(5) Government medical insurance (gong-fei)
(6) Medical aid
(7) Private medical Insurance: Purchased by Rs union :
(8) Private medical Insurance: Purchased by Individual :

(9) Other medical insurance (specify) , _______
(10) Reimbursed by Rs union
(11) No insurance
(12) Not revelent to me .

F1（1）
（2）2007779（）
（3）
（4）
（5）
（6）
（7）
（8）
（9）
（10）
（11）
（12）
（14）

EF006. How often did the respondent receive assistance in answering section D-Health care and insurance?
[IWER: If it is answered by a proxy, the respondents reaction.]
(1) Never
(2) A few times
(3) Most or all of the time
(4) The section was completed by a proxy respondent（the respondent is absent） → Skip to EF007 EFA007

EF007. What is your relationship to R?
[IWER: What is the proxys relationship to R? If unknown, please ask the proxy.]
(1) Spouse
(2) Mother
(3) Father
(4) Mother-in-law /
(5) Father-in-law /
(6) Sibling
(7) Brother-in-law, sister-in-law /
(8) Child
(9) Spouse of child
(10) Grandchild
(11) Other relative
(12) Helper or other non-relative

EF008. [IWER: Please record the reason for proxy]
What is the main reason for proxy (the respondent is absent)

（1）The respondent has serious physical handicaps__
（2）The respondent has serious mental handicaps,
（3）The respondent has rejected this interview.
（4）Other____. ____(EF008_1)

F WORK, RETIREMENT AND PENSION

FA JOB STATUS

FA001. Did you engage in agricultural work (including farming, forestry, fishing, and husbandry for your own family or others) for more than 10 days in the past year? 10（）

(1) Yes → Skip to FB001 FB001
(2) No

FA002. Did you work for at least one hour last week? We consider any of the following activities to be work: earn a wage, run your own business and unpaid family business work, et. al. Work does not include doing your own housework or doing activities without pay, such as voluntary work.

(1) Yes → Skip to FB001 FB001
(2) No

FA003 . Do you have a job but are temporarily laid-off, or on sick or other leave, or in-job training?

(1) Yes
(2) No → Skip to FA007 FA007

FA004. In what month and year did you leave or attend training?

______1900..2011 (FA004_1) Year_______0..12 (FA004_2) Month

[IWER: Mark the year using four digits. Take down the month as its actual number. For example, write January as 1 not 01, December as 12. If do not remember month, fill 0. : 41101,12120]

[Double check those stop work for a long time. (Interview Year – FA004_1) + (Interview month – FA004_2)/12 > 1?]

FA005. Do you expect to go back to this job at a definite time in the future or within 6 months? 6

(1) Yes → Skip to FB001 FB001
(2) No

FA006. Do you still receive any salary from this job? ?

(1) Yes →Skip to FB001 FB001
(2) No

FA007. Have you worked for at least three months during your lifetime (work includes agricultural w ork,earning wage work, self-employed activities, and unpaid family business work, et. al.)? ()

(1) Yes →Skip to FB001 FB001
(2) No

FA008. Work includes all kinds of labour excluding doing your own housework, whether you earn wages or not. Are you sure that you didnt work at least three months during your lifetime?

（1） Yes, never worked before
（2） No, ever worked. →Skip to FB001 FB001

FA009. What is the main reason for you not to work in your lifetime?

(1) Disabled (physical or psychological) （）
(2) Homemaker
(3) My family is too rich that I dont need to work
(4) Taking care of siblings
(5) Other

[PROCEDURE: If FA007 = 2[never worked], then go to FN001 [pension section]. FA007 = 2FN001]

FB WORK HISTORY

FB001. At what age (or in which year) did you start working (or farming), excluding part-time job during school time? () （）
Age ___ 1…120 (FB001_1)Yearsor ___1900..2011 (FB001_1) Year
[IWER: Mark the year in four digits.4]

FB002. What type of work unit was your first job, A government organization, shiye danwei, firm, NGO, inidivdual farmer, or household enterprise?
(1) Government
(2) Institutions
(3) NGO （）
(4) Firm
(5) Individual firm
(6) Farmer
(7) Individual household
(8) Other

[PROCEDURE: If FB002 = 4, then ask FB003, otherwise go on to FB004 . FB002 =4FB003FB004]

[Show Card 20]
FB003. What is the ownership type of the business?
(1) 100% State owned firm
(2) State-controlled firm
(3) 100% Collective-owned firm
(4) Collective-controlled firm
(5) 100% Private firm /
(6) Private-controlled firm
(7) 100% foreign-owned
(8) Joint venture
(9) Other joint-ownership
(10) Other

FB004. Where was your first workplace located?
(1) this village/community /
(2) other village/community in this county/city //
(3)Another county/city in this province/________ (FB004_1)city_______ (FB004_2) county
(4)Another province ________(FB004_3)province //___________(FB004_4)city__________(FB004_5) county
[IWER: Mark 0 if there is no fixed address. 0]
[IWER: Choose from the list of provincessee appendix 2.]

[PROCEDURE: If FB002 = 4 and FB003= 1/ 2/3/4, then skip FB005 and FB006. FB002= 4FB003 =1/ 2/3/4, FB005FB006 .]

FB005. Over your career did you ever have employment in a state owned, state controlled or collectively owned enterprise?
(1) Yes
(2) No Go to FB007 FB007

FB006. Specific ownership type
(1) 100% State owned firm
(2) State-controlled firm
(3) 100% Collective-owned firm
(4) Collective-controlled firm

FB007. Not counting current non-employment or retirement, did you stop working for an extended period of

time （more than 1 year once）due to reasons of family, health, school, etc.? （1）
(1) Yes
(2) No →Skip to FB011 FB011

FB008. How long were the interruptions in all?
____ 1…120 (FB008_1) Years ___0..11(FB008_2)Months
[IWER: Mark the month and year using integert; if 0, please fill 0. 0]

FB009 When was the longest time period that you stopped working? From____ 1900..2011 (FB009_2)year____ 0..12 (FB009_3)month to____ 1900..2011 (FB009_5)year___0..12 (FB009_6)month
[IWER: Mark the year using four digits. Take down the month as its actual number. For example, write January as 1 not 01, December as 12. If do not remember month, fill 0. : 41101,12120]
[Soft Check: Reprompt for FB008 and FB009 if longest time period is greater than Total from FB008 or (FB009_3 +FB009_4 /12)-(FB009_1 +FB009_2 /12) > FB008_1 +FB008_2 /12.]
[Another check to avoid that interruption began before starting work. FB009_1 < FB001_1 or FB009_1 < CV009_a + FB001_1]

FB010. The reason was:
(1) Family （）
(2) Health （）
(4) School
(5) Unemployment/layoff /
(6) Other

FB011. Have you completed retirement procedures（including early retirement）or internal retirement（Note: Retirement from government departments, enterprises and institutions, not including retirement in the sense of getting agricultural insurance）? （）（）
(1) Yes → skip FB012 FB012
(2) No

FB012. Have you completed receding position procedures
(1) Yes
(2) No
F1

[PROCEDURE: If FA007=1, please skip to FK001. FA007 =1FK001.]
[PROCEDURE: If FA008=2, please skip to FL001. FA008 =2FL001.]

FC CURRENT PRIMARY JOB/OCCUPATION

SKIP PATTERN: If R did farming last year (FA001=1), ask FC001; if R did not do farming last year, but worked last week (FA001 =2 and (FA002 =1 OR [FA002 =2 & FA003 =1 & FA005 =1]), ask FC019.
FC001（FA001 =2 and (FA002 =1 OR [FA002 =2 & FA003 =1 & FA005 =1]）FC019

FARM ENPLOYED

FC001. Did you work for other famers in wage for at least ten days in the past year（Agricultural work in wages）10（）
(1) Yes
(2) No →Skip to FC008 FC008
F1

FC002. How many employers did you work for in the past month?
____

FC003. Where is your workplace for most time?

(1)this village/community /
(2)other village/community in this county/city //
(3)Another county/city in this province/_______ (FC003_1)city________ (FC003_2)county
(4)Another province ________(FC003_3)province //___________(FC003_4)city___________(FC003_5) county

[IWER: Mark 0 if there is no fixed address. 0]
[IWER: Choose from the list of provincessee appendix 2.]

FC004. How many months did you work on cropping (forestry), livestock, and fishing in wage for other famers in the past year? [()][()] [()] _____ 0..12Months

FC005. How many days did you work in wage for other famers per week on average during a normal work month in the past year? [()] [()][()] ______ 0..7Days

FC006. How many hours did you usually work in wage for other famers per day during a normal work day in the past year? [()] [()] [()] _____ 0..16 Hours
[Soft Check: Verify if number of hours per day is unreasonable, e.g., FC006 >16]

FC007. What is the average monthly wage did you get in your working months in the past year? ___ Yuan

HOUSEHOLD AGRICULTURAL WORK

FC008. Did you work for your own household for at least ten days in the past year10
(1) Yes
(2) No →Skip to FC013 FC013

FC009. How many months did you work on [cropping (forestry), livestock, and fishing] for your own householdin the past year? [()][()] [()] _____ 0..12Months

FC010. How many days did you work for your own household per week on average during a normal work month in the past year? [()] [()] [()] _____0..7 Days

FC011. How many hours did you usually work for your own household per day during a normal work day in the past year? [()] [()] [()] ______ 0..24Hours
[A check similar to the one for EP062. FC011 > 16]

FC012. Where is your workplace for most time? (preload sampling community ID)
(1)this village/community /
(2)other village/community in this county/city //
(3)Another county/city in this province/,_______ (FC012_1)city______(FC012_2)county
(4)Another province _______(FC012_3)province //__________(FC012_4)city________(FC012_5)county

[IWER: Mark 0 if there is no fixed address. 0]
[IWER: Choose from the list of provincessee appendix 2.]

FC013. How many days of work did you miss last year due to health problems? ______ 0..366 Days
[IWER: Mark 0 if you didn't miss any work days. 0]

FC014. Besides agricultural work, did you work for at least one hour last week in wage or self-employed work or umpaid family business? ()
(1) Yes →Skip to FC019 FC019

（2）No

FC015. Do you have wage or self-employed work but are temporarily laid-off or are on sick, seasonal, or other leave or in-job training? （）

(1) Yes
(2) No →Skip to FC018 FC018

FC016. In what month and year did leave or attend training? //
______1900..2011 (FC016_1) Year_______0..12 (FC016_2) Month
[IWER: Mark the year using four digits. Take down the month as its actual number. For example, write January as 1 not 01, December as 12. If do not remember month, fill 0. : 41101,12120]
[Same check as the one for FA004 . (Interview Year –FC016_1 + (Interview month – FC016_2)/12 > 1?]

FC017. Do you expect to go back to this job at a definite time in the future or within 6 months? 6

(1) Yes →Skip to FC019 FC019
(2) No

FC018. At what age do you plan to stop working? Stopping work in this context shall refer to having stopped all income-related activities, unpaid family business and having no intention of engaging in anything more serious than small pastime work. ?______ 1…120Years old
[IWER: Please tell me the approximate age. Mark 0 if you plan to keep working until you are physically able. 0]
F1

→Skip to pension and social security section (FN001) if have not processed retirement and receding (FB011 ==2 and FB012 ==2); or skip to retirement section (FM001) if processed retirement or receding (FB011 ==1 or FB012 ==1)（FB011 ==2 FB012 ==2）FN001 (FB011 ==1 FB012 ==1)FM001

FC019. Besides agricultural work, do you currently hold more than one non-agricultural job? （）
[IWER: non-agricultural job includes paid jobs, self-employed activities, unpaid family business work, et. al. Activities without pay, such as voluntary work, are not included. 2]

(1) Yes Skip to FC020 FC020
(2) No Skip to FC021 FC021

FC020. Among all your jobs, which one is your main job? [Main job is defined as the job at which you work the longest hours] Do you earn a wage or do you run your own business or work for unpaid family business? []

(1) Employed →Skip to FD001 FD001
(2) Self-employed →Skip to FH001 FH001
(3) unpaid family business →Skip to FH001 FH001

FC021. How do you describe your non-agricultural job? Do you earn a wage or do you run your own business or work for unpaid family business?

(1) Employed →Skip to FD001 FD001
(2) Self-employed →Skip to FH001 FH001
(3) unpaid family business →Skip to FH001 FH001

FD EMPLOYED

FD001. Do you receive wages from your current workplace or receive them from a dispatch/contract company?

(1) Place of work
(2) Labor dispatch company

F1

[CAPI: For dispatched/contract workers (FD001 =2), mention the following for questions FD002 -FD016 (FD001=2)FD002-FD016] The next few questions pertain to the situation at your current workplace, and not to the company that has dispatched/contracted you out.

FD002. Do you work for a government organization, institution, firm, NGO, individual farmer, or resident household? （）

(1) Government
(2) Institutions → Skip FD006 FD006
(3) NGO （） → Skip FD006 , FD010 FD006 , FD010
(4) Firm → Skip FD006 FD006
(5) Individual firm → Skip FD006 , FD010 FD006 , FD010
(6) Farmer → Skip FD005 ,FD006 , FD010 FD005 ,FD006 ,FD010
(7) Individual household → Skip FD005 ,FD006 , FD010 FD005 ,FD006 , FD010
(8) Other → Skip FD006 , FD010 FD006 , FD010

[INTRO: We will ask your work history later.In order to distinguish the work units, we need the full name and address of your employer.]

FD003. What is the name of your workplace/employer? Please state specifically the name of your company or institution. （） ________________________

[IWER: Write the name of the household head if R works for a family.]

FD004. Where is your workplace located? (preload sampling community ID)

(1)this village/community /
(2)other village/community in this county/city //
(3)Another county/city in this province/__________(FD004_1)city__________(FD004_2) county
(4)Another province ________(FD004_3)province //_______ (FD004_4)city_____ ______ **(**FD004_5) county

[IWER: Mark 0 if there is no fixed address. 0]
[IWER: Choose from the list of provincessee appendix 2.]

FD005. What kind of business or industry do you work in—that is, what does your workplace primarily make or do? ______________________________

[IWER: Type of business]

If government employee (FD002 =1), ask:

FD006. Are you a civil servant?

(1) Yes
(2) No

FD007. Are you a formal employee of an establishment?

(1) Yes
(2) No

Skip to FD011 FD011

If institution (FD002 =2), ask:

FD008. Is your institution operated as a firm or as a government unit?

(1) As a firm
(2) As a government unit

FD009. Are you regular worker?

(1) Yes

(2) No

If firm (FD002=4), ask:
[Show Card 20]
FD010. What is the ownership type of the business?
(1) 100% State owned firm
(2) State-controlled firm
(3) 100% Collective-owned firm
(4) Collective-controlled firm
(5) 100% Private firm /
(6) Private-controlled firm
(7) 100% foreign-owned
(8) Joint venture
(9) Other joint-ownership
(10) Other

FD011. When did you start working for this employer?
______1900..2011 (FD011_1)Year_______ 0..12 (FD011_2)Month
[IWER: Mark the year using four digits. Take down the month as its actual number. For example, write January as 1 not 01, December as 12. If do not remember month, fill 0. : 41101,12120]
[Soft Check: Consistency of employment start date. Make Sure that the individual was at least a minimum age when he/she started working for this employer (e.g., prompt to check if under 16?). Specifically (FD011_1+ FD011_2 /12)- (CV009_a+ CV009_b/12) <16 prompts a soft check.]

[Replace 16 in the above soft check with FB001. (FD011_1+ FD011_2 /12)- (CV009_a+ CV009_b/12) < FB001_1 or FD011_1 <FB001_2]

FD012. What sort of work do you do? (E.g., cleaner, accountant, teacher, etc.) () ______
____
[IWER: Ask about the specific work that R does.]

FD013. What is your current position?
(1) Clerk/worker
(2) Team Leader （）
(3) Section Chief
(4) Director of a division
(5) Director-General of a bureau and above
(6) Village Leader
(7) Township Leader
(8) Division manager
(9) Overall/General manager
(10) Others

FD014. What is your current professional/technical level? /
(1) Technician
(2) Primary level
(3) Intermediate level
(4) Advanced level
(5) No professional/technical level
F1/
[PROCEDURE:Skip this question if answer to FD013 is 2-9. FD013 2-9]
FD015. Are you in a position to supervise others?
(1) Yes
(2) No → Skip to FD017 FD017

FD016. How many people are there under your supervision?
(1) 1~5 people 1~5
(2) 6~10 people 6~10

(3) 11~15 people 11~15
(4) 16~30 people 16~30
(5) 31~99 people 31~99
(6) More than 100 people 100

[CAPI: For dispatched worker(FD001 =2), prompt for FD017 -FD030 (FD001 =2)FD017 -FD030]
The next few questions are about your dispatch work unit.

[PROCEDURE:Do not ask FD017 to FD019 if FD013 =3-5 （government officials）. FD013 =3-5FD017 , FD018 , FD019]
FD017. What is your employment type at your current workplace?
(1) Regular worker
(2) Contract worker
(3) Casual/Part-time worker /

FD018. Do you have a personnel file?
(1) Yes
(2) No → Skip to FD020 FD020

FD019. Where is your personnel file kept?
(1) With my current employer
(2) Other work unit but not current employer ()
(3) With the Job Service Center in this city /
(4) HuKou place in other city
(5) Other city

FD020. Did you receive a labor contract (or employment contract) in written form from your current workplace(or labor dispatch company)? /
(1) Yes
(2) No Skip to FD024 FD024

FD021. What is the agreed period of employment (labor contract period)?
(1) Defined period _______ 0..100 (FD021_1)Years _______0..11(FD021_2)Months [IWER: if do not remember months, please fill '0'. 0]
(2) Not defined → Skip to FD024 FD024
(3) Same as the term of the project

FD022. Has the current employment contract ever been renewed?
(1) Yes
(2) No (Current contract is the first contract) ()
→ Skip to FD024 FD024

FD023. How many times has the contract been renewed? ____ 1..50times

FD024. How long do you expect to work at your current workplace?
(1) Less than one year 1
(2) One to two years 1-2
(3) Two to three years 2-3 Skip FD025 FD025
(4) More than three years 3 Skip FD025 FD025

FD025. Why do you expect so?
(1) Because the predefined contract period will expire
(2) Because typically the contract expires (although theres no written contract) （）
(3) Because I was hired under the condition that I would resign upon the request of my employer
(4) Because the current job/project will be completed /
(5) Because the person I am substituting/replacing will return to work
(6) Because I can only work during certain seasons

(7) Because I plan to find another job that better suits my job aptitude, abilities, and preferences
(8) Because I will reach retirement age as set by regulations/practice
(9) Because of family care responsibilities, poor health, etc.
(10) Other

[If R has not processed retirement (FB011 =2) ask: :]
FD026. Is this work unit going to process retirement for you?
(1) Yes
(2) No →Skip to FD029 FD029

FD027. At what age will you process retirement? ____ 45..120 Years old
Skip to FD029 FD029
[Soft Check for Reasonable Age: If (FD027 < 50 & CV004==2) or (FD027 < 55 & CV004==1) prompt for verification.]

[Add another check to see if the reported age above is smaller than actual age. To be specific, FD027 < (Interview Year – CV009_a)]

If R has processed retirement (FB011 =1) ask: （）
FD028. Did you process retirement through this work unit?
(1) Yes
(2) No

FD029. Except for national/public holidays, how many days of paid vacation do you have this year at your current workplace? _____ 0..366Days
[IWER: Mark 0 if there is no paid vacation. 0]
[Soft Check for reasonable range: If FD029 >30?, prompt for verification]

FD030. How many days of work did you miss at this current job in the past year due to health problems? _______ 0..366Days
[IWER: Mark 0 if you didn't miss work. 0]

[PROCEDURE: If FD030 =0, skip FD031] [FD030 =0FD031]
FD031. In these days, how many did not deduct wage or bonus? ______ 0..366Days
[Soft Check for reasonable range: If FD031 > FD030, prompt for verification]

FE QUESTIONS ABOUT LABOR SUPPLY

[CAPI: For dispatched worker (FD001 =2), prompt for FE001 - FE003 :] The next few questions about labor supply are about the situation of your work place, not dispatch work unit. [CAPI: (FD001 =2)FE001 -FE003]

FE001. Counting paid vacations and sick leave not deducting wage as work, how many months did you work in the past year? （） ______ 0..12Months

FE002. How many days a week did you work on average in the past year? _______ 0..7Days

FE003. How many hours did you work per day on average in the past year, excluding meal breaks but including any paid or unpaid overtime? （） ______ 0..24Hours
[Soft Check: Verify if number of hours per day is unreasonable, e.g., FE003 >16]

FF QUESTIONS ABOUT WAGES:

[CAPI: For dispatched worker (FD001 =2), prompt for FF001 -FG014 : (FD001=2)FF001 -FG014] **The following questions about salary and benefits refer to what you receive from the dispatch company.**

FF001. How is your wage paid mainly? Is it regularly paid, contract-based, performance-based, or other? If it is regularly paid, please tell me how often you receive your wages. Do you have a yearly contract, monthly, weekly, daily, or hourly? Please select one.

(1) Yearly salary
(2) Monthly salary → Skip to FF004 FF004
(3) Weekly salary → Skip to FF006 FF006
(4) Daily salary → Skip to FF008 FF008
(5) Hourly salary → Skip to FF010 FF010
(6) Contract-based → Skip to FF012 FF012
(7) Performance-based → Skip to FF012 FF012
(8) Other → Skip to FF012 FF012

FF002. What is the after-tax salary including bonus in the last year? （） _______ Yuan
[Soft Check: Prompt for clarification if under a low threshold, e.g. under 1200 RMB annual. Specifically, prompt to clarify if FF002 <1200RMB.]

FF003. [IWER: If R is unwilling to answer or does not remember, ask unfolding bracket questions here.]10,000 /30,000 /50,000 /100,000 /200,000 yuan

Skip to FF014 FF014

FF004. What is the after-tax salary including bonus last month? （） ___ Yuan
Soft Check: Prompt for clarification if under a low threshold, e.g. under 100 RMB per month. Specifically, prompt to clarify if FF004 <100RMB.

FF005. [IWER: If R is unwilling to answer or does not remember, ask unfolding bracket questions here.]: 500 /1,000 /2,500 /5,000 /10,000 yuan

Skip to FF014 FF014

FF006. What is the wage including bonus last week? （） _____ Yuan
[Soft Check: Prompt for clarification if under a low threshold, e.g. under 25 RMB per week. Specifically, prompt to clarify if FF006 <25RMB.]

FF007. [IWER: If R is unwilling to answer or does not remember, ask unfolding bracket questions here.]: 200 /500 /10,00 /25,00 /5,000 yuan
Skip to FF012 FF012

FF008. What is the usual daily wage? _____ Yuan .
Soft Check: Prompt for clarification if under a low threshold, e.g. under 5 RMB per day. Specifically, prompt to clarify if FF008 <5 RMB.
FF009. [IWER: If R is unwilling to answer or does not remember, ask unfolding bracket questions here.]: 20 /50 /100 /200 /500 yuan

Skip to FF012 FF012

FF010. What is your hourly wage? ____Yuan
Soft Check: Prompt for clarification if under a low threshold, e.g. under 1 RMB per hour. Specifically, prompt to clarify if FF010 <1 RMB.

FF011. [IWER: If R is unwilling to answer or does not remember, ask unfolding bracket questions here.]: 10 /30 /50 /100 /200 yuan

Skip to FF012 FF012

FF012. How much on average do you receive last month after taxes (including bonus)? , _____Yuan
Soft Check: Prompt for clarification if under a low threshold, e.g. under 100 per month. Specifically,

prompt to clarify if FF012 <100 RMB.

FF013. [IWER: If R is unwilling to answer or does not remember, ask unfolding bracket questions here.]: 1,000 /3,000 /5,000 /10,000 /20,000 yuan

FF014. What is the value of all other bonuses (not paid at same time as regular wage) received in the past year? （） （） ______Yuan
Soft Check: Prompt on bonus and monthly earnings if bonus is more than five times monthly net income, e.g., re-ask some questions if FF014 >5*FF012 | FF014 >5*FF012
[JGuo: delete the end part of the above sentence?]

FF015. [IWER: If R is unwilling to answer or does not remember, ask unfolding bracket questions here.]1,000 /3,000 /5,000 /10,000 /20,000 yuan

FG Fringe Benefits

[Show Card 21]
FG001. The following are fringe benefits which may be provided by a company. Please answer if the following are provided by your current workplace and whether you benefit from the following. (Check all that apply) ()
(1) Free lunch
(2) Free breakfast
(3) Free dinner
(4) Meal cash subsidy
(5) Transportation cash subsidizations
(6) Free housing
(7) Subsidization of housing
(8) Company car
(9) Company bus
(10) Other subsidies （）
(11) None Skip to FG003 FG003

For each choice, ask: FG002
FG002. How much is the value of the subsidy per month? （） __ Yuan

[CAPI: If FD006 = 1 or FD007 = 1 or FD009 =1, skip FG003 -FG014. FD006 = 1 or FD007 = 1 or FD009 =1FG003 -FG014]

[PROCEDURE: If R has not processed retirement（FB011 =2）, ask: FG003 -FG014. （FB011 =2）FG003 -FG014]
[Show Card 22]
FG003. Does your employer provide pension insurance, health insurance, unemployment insurance, workers injury insurance and maternity insurance (Choose all that apply) （）
(1) Pension → Skip to FG009 FG009
(2) Health insurance → Skip to FG009 FG009
(3) Unemployment insurance → Skip to FG009 FG009
(4) Workers injury insurance → Skip to FG009 FG009
(5) Maternity insurance → Skip to FG009 FG009
(6) None

FG004. Why are you not covered by the above mentioned social insurance through your employer?（Choose all that apply）？ （）？（）
(1) There is no social insurance through employment in my local area → Skip to FG014 FG014

(2) Social insurance through work is available in my local area but my employer does not provide it to me → Skip to FG014 FG014
(3) My employer offers it but I am unwilling to join → Skip to FG014 FG014
(4) I am in the trial period → Skip to FG014 FG014
(5) I have social insurance from elsewhere → Skip to FG005 FG005
(6) I have passed retirement age → Skip to FG014 FG014

FG005. From where do you have/receive the above mentioned social insurance? （）
(1) I am covered through another work unit → Skip to FG006 FG006
(2) I contribute through the job service center → Skip to FG008 FG008

FG006. Why do you contribute the above mentioned social insurance fees through another work unit? （）
(1) I was laid off by this work unit but my employment contract has not terminated
(2) I am on leave from this employer
(3) I keep my position but do not receive pay from this work unit
(4) A friend in this work unit is helping me this way
(5) Other

FG007. Where is this work unit located?
(1) Same city
(2) Different city, same province
(3) Different province

Skip to FG009 FG009

FG008. Who pays for your contribution through the job service center? （）
(1) My employer
(2) Myself
(3) My employer and myself

All those paying social insurance through whatever means, ask（FG003=1-4 or FG004=5）FG009 – FG013

FG009. Is the salary you told me about earlier the net amount after paying for the above benefits?
(1) Yes
(2) No

FG010. On what income base is the contribution to the above mentioned social insurance determined? （）
________________ Yuan/month/

FG011. Do you know how much you or your employer (another work unit) contributes to the above benefits? （） （）
(1) I know the amount by myself and the employer
(2) I know the amount by myself but not the employer→ Skip to FG013 FG013
(3) I know neither my contribution nor my employers contribution. Skip to FG014 FG014

FG012. How much is your employers contribution in total?
_______ Yuan/month /
Soft Check Income Report and FG012 if Contribution is Greater than Monthly Income

FG013. How much is your own contribution in total? （） ___Yuan/month /

Soft Check Income Report and FG013 if Contribution is Greater than Monthly Income

FG014. Does your employer provide funding for public housing?
(1) Yes
(2) No

F1
Soft Check: Verify if FG014 > than some threshold.

FG015. At what age do you plan to stop working? Stopping work in this context shall refer to having stopped all income-related activities and unpaid family business and having no intention of engaging in anything more serious than small pastime work. ?_____ 0..120Years old
F1
[IWER: Please tell me the approximate age. Mark 0 if you plan to keep working until you are physically able. 0]
Soft Check: Prompt for correction if current age is greater than response, e.g. (Interview Year+ Interview month/12) – (CV009_a+CV009_b/12) > FG015
→ Skip to FJ001 FJ001

FH NON-FARM SELF-EMPLOYED AND UNPAID FAMILY BUSINESS

FH001. How many months did you work in the past year? ______ 0..12 Months

FH002. How many days did you work per week on average in the past year? _____ 0..7Days

FH003. How many hours did you work per day on average in the past year, excluding meal breaks but including any paid or unpaid overtime on a normal work month? ______ 0..24hours
Soft Check: Verify if number of hours per day is unreasonable, e.g., FH003 >16

FH004. How many days of work did you miss in the past year due to health problems? ______0..366Days
[IWER: Mark 0 if you didn't miss any work days. 0]

Next are some questions about the main self-employed work.

IF FC020 =2 or FC021 =2[Self-employed], askFC020 =2 FC021 =2:

FH005. What is the name of your company or workplace?
[IWER: If there is more than one company, ask about the main one. Mark 0 if there is no name. 0]
____

FH006. Where is your company or workplace located? /
(1)this village/community /
(2)other village/community in this county/city //
(3)Another county/city in this province/_____ (FH006_1)city________(FH006_2) county
(4)Another province ________(FH006_3)province //___________(FH006_4)city__________(FH006_5) county
[IWER: Mark 0 if there is no fixed address. 0]
[IWER: Choose from the list of provincessee appendix 2.]

FH007. What kind of business or industry do you work in—that is, what does your company do or make?
____
[IWER: Type of business]

FH008. When did you start working at the current company or workplace? /______ 1900..2011 (FH008_1)Year_______0..12 (FH006_2) Month
[IWER: Mark the year using four digits. Take down the month as its actual number. For example, write January as 1 not 01, December as 12. If do not remember month, fill 0. : 41101,12120]
[**A check similar to the one for FD011**. **(FD011_1 + FD011_2 /12)- (CV009_a+ CV009_b/12) < 16 or (FD011_1 + FD011_2 /12)-(CV009_a+ CV009_b/12) < FB001_1 or FH008_1 < FB001_2**]

FH009. Do any other household members work in the same self-employed activity?
(1) Yes Skip to FI001 FI001
(2) No

[IWER: Income from self-employment if R is the single operator （FH009 =2）. If other family member involved, income is asked in household section. FI001 ,]

FH010. Not including spending on fixed capital, what is your best estimate of net income earned from this activity in the last year? Remember to consider the following types of costs: energy, housing or equipment rental, raw materials, transportation, marketing, wages, taxes, and other fees. /________Yuan
Soft Check: Prompt for verification if net income is less than 1200 RMB / year. Or, prompt if FH010 <1200RMB

FH011. [IWER: If R is unwilling to answer or does not remember, ask unfolding bracket questions here.]: 5,000 /10,000 /50,000 /100,000 /200,000 yuan

Next are some questions about your unpaid family business.

IF FC020 =3 or FC021 =3[Unpaid family business], ask:
FC020 =3 FC021 =3[], :

FH012. What is the name of company or workplace that you work in without wage? [IWER:Mark 0 if there is no name. 0.]
____

FH013. Where is this company or workplace loacted? /
(1)this village/community /
(2)other village/community in this county/city //
(3)Another county/city in this province/___________(FH013_1)city_______ (FH013_2)county
(4)Another province ________(FH013_3)province //___________(FH013_4)city___________（FH013_5）county
[IWER: Mark 0 if there is no fixed address. 0]
[IWER: Choose from the list of provincessee appendix 2]

FH014. What kind of business or industry do you work in—that is, what does this company do or make?
____
[IWER: Type of business]

FH015. What sort of work did you do? (E.g., cleaner, accountant, etc.) ()
____
[IWER: Ask the specific work.]

FH016. How many family members, relatives or friends who work without payment are there including yourself? /（）
___ People

FH017. When did this company or workplace start operation//
______ 1900..2011 (FH017_1)Year_______0..12 (FH017_2) Month

FH018. When did you start working at the current company or workplace? /______1900..2011 (FH018_1)Year_______0..12 (FH018_2) Month

FH019. Do any other household members work in the same company or workplace? /
（1）Yes Skip to FI001 FI001
（2）No

[IWER: Income from self-employment if no other household members work in（FH019 =2）. If other family member involved, income is asked in household section. /FI001 ,]

FH020. Not including spending on fixed capital, what is your best estimate of net income earned from this

activity in the last year? Remember to consider the following types of costs: energy, housing or equipment rental, raw materials, transportation, marketing, wages, taxes, and other fees. /________ Yuan
Soft Check: Prompt for verification if net income is less than 1200 RMB / year. Or, prompt if FH020 <1200RMB

FH020_bracket. [IWER: If R is unwilling to answer or does not remember, ask unfolding bracket questions here.]: 5,000 /10,000 /50,000 /100,000 /200,000 yuan

FI Social insurance questions for the self-employed.

[PROCEDURE: If R has not processed retirement（FB011 =2）, ask: FI001 -FI011 . （FB011 =2）FI001 -FI011]

[Show Card 22]
FI001. Next are questions about your social insurance. Are you covered by pension insurance, health insurance, unemployment insurance, workers injury insurance or maternity insurance through your own self-employment(Choose all that apply) （）

(1) Pension → Skip to FI005 FI005
(2) Health insurance → Skip to FI005 FI005
(3) Unemployment insurance → Skip to FI005 FI005
(4) Workers injury insurance → Skip to FI005 FI005
(5) Maternity Insurance → Skip to FI005 FI005
(6) None

FI002. Why are you not covered by the above mentioned social insurance through self-employment? （）?

(1) It is not possible for the self-employed to participate in social insurance in my local area → Skip to FI012 FI012
(2) Social insurance is available to the self-employed but I do not participate → Skip to FI012 FI012
(3) I have social insurance from elsewhere
(4) I have passed retirement age → Skip to FI012 FI012

FI003. From where do you have the above mentioned social insurance? （）

(1) I am covered through another work unit
(2) I contribute through job service center → Skip to FI005 FI005

FI004. You contribute through another work unit, why? （）

(1) I was laid-off by this work unit but employment contract is not terminated
(2) I am on leave from this employer
(3) I keep my position but do not receive pay from this work unit
(4) A friend in this work unit is helping me this way
(5) Other

Skip to FI005 . FI005

[PROCEDURE: If R has social insurance, either from self-employment or by job service center, ask: FI005 – FI011. FI005 – FI011]

FI005. How much is your own contribution to the above mentioned social insurance last year? （）____Yuan
Soft Check: Prompt if FI005 =0 or FI005 > FH010 /2.

If FH009 =2skip FI006 . FH009 =2FI006

FI006. Is the income you told us about earlier net after paying for the above benefits?

(1) Yes
(2) No

FI007. On what income base is the contribution to the above mentioned social insurance determined? （）
_______________ Yuan/month/

[PROCEDURE: If R participates in social insurance through another work unit（FI003 =1）, ask: FI008 -FI010 . （FI003 =1） FI008 -FI010]

FI008. Do you know how much you or that work unit contributes to the above benefits? （）
(1) I know the amount by myself and the work unit → Skip to FI009 FI009
(2) I know the amount by myself but not the work unit → Skip to FI010 FI010
(3) I do not know either my contribution or the work units contribution at all. → Skip to FI011 FI011

FI009. How much is work units contribution in total? ______ Yuan/month /
Soft Check: Prompt for Verification if Contribution is Greater than Monthly Earnings, e.g., FI009 >FH010

FI010. How much is your own contribution to pension in total? （） ______ Yuan/month /

Soft Check: Prompt for Verification if Contribution is Greater than Monthly Earnings, e.g., FI010 >FH010

FI011. Do you expect to process retirement at this business/organization?
(1) Yes
(2) No

FI012. At what age do you plan to stop working? Stopping work in this context shall refer to having stopped all income-related activities and unpaid family business and having no intention of engaging in anything more serious than small pastime work. ?
______ 0..120Years old
F1

[IWER: Please tell me the approximate age. Mark 0 if you plan to keep working until you are physically able. 0]
Soft Check: Prompt for correction if current age is greater than response, e.g. (Interview Year+ Interview month/12) – (CV009_a+CV009_b/12) > FI012

FJ SIDE JOB (EMPLOYED OR SELF-EMPLOYED) （）

If FC019 =1 (more than one job), proceed with the following section. FC019 =1 （）

FJ001. How many jobs do you currently hold, excluding your main job? ______ 1..20

FJ002. How many hours a week do you work on average at your side job(s), not considering your main job?
________ 0..168 Hours per week /

FJ003. What is the average monthly income or wage that you get from side job(s) other than your main job?
___________ Yuan per month /

FJ004. [IWER: If R is unwilling to answer or does not remember, ask unfolding bracket questions here.]:
500 /1,000 /2,500 /5,000 /10,000 yuan

END OF CURRENT JOB

FK UNEMPLOYMENT AND JOB SEARCH ACTIVITIES

For those not currently working but once employed (FA007 =1) (FA007 =1)
FK001. Next are some questions about circumstances about your non-employment and job search activities. In what month and year did you last work?
______ 1900..2011 (FK001_1)Year_______0..12 (FK001_2) Month
[IWER: Mark the year using four digits. Take down the month as its actual number. For example, write January as 1 not 01, December as 12. If do not remember month, fill 0. : 41101,12120]
[**Compare with the time when started work. FK001_1 < FB001_1 or (FK001_1 – CV009_a) < FB001_1**]

FK002. Did you search for a new job during the last month?
(1) Yes
(2) No Skip to FL001 FL001

FK003. At what age do you plan to stop working? Stopping work in this context shall refer to having stopped all income-related activities and unpaid family business and having no intention of engaging in anything more serious than small pastime work. ?
____0..120Years old
F1
[IWER: Please tell me the approximate age. Mark 0 if you plan on working until you are physically capable. 0]
[Soft check: Compare with current age. FK003 < (Interview Year – CV009_a)

FL MOST RECENT JOB

— RESPONDENT NOT CURRENTLY WORKING
——
Ask if FA007 =1 or (FA007=2 and FA008 =2)
IWERThe next questions are about the last main job you had, which could be farming, earning a wage, running your own business or working for unpaid family business. It does not include doing your own housework or doing activities without pay, such as voluntary work. If you have more than one job, we are interested in the job at which you worked the longest hours. Were interested in your situation near the termination of this job.

FL001. Did you work for someone else(including work for unpaid family business), were you self-employed, did you farm, or were you otherwise employed? （）
(1) Employed （）
(2) Self-employed（）
(3) Unpaid family business
(4) Farming （）

FL002. In which year and month did you start working at that job?
______1900..2011 (FL002_1) Year_______0..12 (FL002_2) Month
[IWER: Mark the year using four digits. Take down the month as its actual number. For example, write January as 1 not 01, December as 12. If do not remember month, fill 0. : 41101,12120]
Soft Check: Prompt for Verification/Clarification if the Respondent was Less than 16 at time of starting this job, e.g. Prompt if (FL002_1+FL002_2 /12)-(CV009_a+CV009_b/12)<16

[Replace 16 in the above check with FB001 . FL002_1 < FB001_2 or FL002_1 < (CV009_a + FB001_1)]

FL003. In which year and month did you stop working at that job?
______1900..2011 (FL003_1) Year_______0..12 (FL003_2) Month
[IWER: Mark the year using four digits. Take down the month as its actual number. For example, write

January as 1 not 01, December as 12. If do not remember month, fill 0. : 41101,12120]
Soft Check: Prompt for Verification/clarification if End Date is Before the Start Date, e.g. FL003_1 <FL002_1

FL004. Where was the job located?
(1)this village/community /
(2)other village/community in this county/city //
(3)Another county/city in this province/___________(FL004_1)city___ ________ (FL004_2)county
(4)Another province ________(FL004_3)province //___________(FL004_4)city___________(FL004_5)county
[IWER: Mark 0 if there is no fixed address. 0]
[IWER: Choose from the list of provincessee appendix 2 :]

If FL001 =4 Skip to FL018 FL001 =4FL018

[PROCEDURE:If non-farmer（FL001 =1or 2 or 3）, ask FL005 to FL008 . （FL001 =123）FL005 FL008]
FL005. What was the name of your workplace/employer? Please state specifically the name of your company or business. （） ____

FL006. What kind of business or industry was it—that is, what did they make or do at the place where you worked? ____
[IWER: Type of business:]
FL007. Is this employer still in existence?
(1) Yes
(2) No

FL008. How many hours a week did you usually work [for this employer/in this business]? []_____ 0..168hours per week/
Soft Check: Prompt for Verification if FL008 >80

Ask only if employed.（FL001 =1）
FL009. What were the monthly wages, bonuses, and subsidies from this jobbefore you stopped working at this job? （） ______Yuan
[IWER: Mark 0 if there is no net income, and mark 999997 if running a deficit.0999997.]
Soft Check: Prompt for Verification if FL009 < 100

[IWER: If R is unwilling to answer or does not remember, ask unfolding bracket questions here.]
FL010. [IWER: If R is unwilling to answer or does not remember, ask unfolding bracket questions here.]: 500 /1,000 /2,500 /5,000 /10,000 yuan

FL011. What was the value of other bonuses not paid with regular wages each year? （） ______yuan

Skip to FL013 FL013

Ask if self-employed （FL001 =2）:
FL012. Do you have employees?
(1) Self-employed with employees
(2) Self-employed without employees
(3) Family business worker without pay

Skip to FL018 FL018

Ask the following only if employed（FL001=1）:FL013-FL017
FL013. Were you a regular worker, a temporary worker, or a casual worker?
(1) Regular wage worker
(2) Contract worker
(3) Temporary wage worker
(4) Casual wage worker

FL014. Did you work for the government, institution, firm, NGO, individual farmer or a resident household?
( )

(1) Government
(2) Institutions →Skip to FL017 FL017
(3) NGO ( ) →Skip to FL017 FL017
(4) Firm →Skip to FL016 FL016
(5) Individual firm Skip to FL017 FL017
(6) Individual farmer →Skip to FL017 FL017
(7) Individual household →Skip to FL017 FL017
(8) Other →Skip to FL017 FL017

If government employee [FL014 =1], ask: [FL014=1],
FL015. Were you a civil servant?
(1) Yes
(2) No
Skip to FL017 FL017

If firm [FL014 ＝4], ask: [FL014 ＝4],
[Show Card 20]
FL016. What was the ownership type of the business?
(1) 100% State owned firm
(2) State-controlled firm
(3) 100% Collective-owned firm
(4) Collective-controlled firm
(5) 100% Private firm /
(6) Private-controlled firm
(7) 100% foreign-owned
(8) Joint venture
(9) Other joint-ownership
(10) Other

FL017. What sort of work did you do? (E.g., cleaner, accountant, etc.) ()
____
[IWER: Ask the specific work.]

[IWER: If R has processed retirement [FB011=1], ask : :]
FL018. Is this the business/organization where you processed retirement?
(1) Yes Skip to FL021 FL021
(2) No Skip to FL020 FL020

[IWER: If R has not processed retirement [FB011 =2], ask: :]
FL019. Do you expect to process retirement from this business/organization?
(1) Yes
(2) No

FL020. Why did you leave that employer?
[IWER: Do not probe but check all that apply.]
(1) Business closed / (FL020_1)
(2) Quit (FL020_2)
(3) I was laid off (FL020_3)
(4) I was fired (FL020_4)
(5) I went to school (FL020_5)
(6) I went abroad (FL020_6)
(7) I stopped working for health reasons (FL020_7)
(8) I stopped working for family reasons (FL020_8)

(9) I was transferred to another job (FL020_9)
(10) I was sent down to the countryside to do manual labor (FL020_10)
(11) I started working off-farm locally (FL020_11)
(12) I went to work away from home (FL020_12)
(13) Better job in local area (FL020_13)
(14) Better job in another location (FL020_14)
(15) I retired (FL020_15)
(16) Other (FL020_16)

FL021. Did you receive any payments other than the legal retirement allowance upon leaving your last job? (For example, condolence paymentworkers compensation, etc.) （）
(1) Yes
(2) No
F1

If FL021 =1 ask:FL021.
FL022. How much was the compensation and for how many years of work?________ Yuan____ years ?_______ (FL022_1)_______0..120(FL022_2)
FL022_bracket. [IWER: If R is unwilling to answer or does not remember, ask unfolding bracket questions here.]: 1000 /2000 /5000 /10,000 /20,000 yuan

End of Last Job

FM RETIREMENT

[IWER: If R has processed retirement [FB011 or FB012 =1], ask: FM001 –FM059.
[FB011=1][FB012=1]//FM001 –FM059]

FM001. Is the work unit that processed your [preload: retirement /receding position] the one you told us about (preload: name of current employer if employed EP027, name of last employer if non-employed EP146)? [/][preload work unit name,]
[IWER: If R has no work unit (no answer to FD003 or FL005), please choose (2) No. FD003 FL005 （2）]
(1) Yes Skip to procedure before FM005 FM005
(2) No

If answer to FM001 =2 is no, then ask FM002 -FM004 :FM001 =2FM002 -FM004
FM002. What is the name of the employer that processed your [preload: retirement /receding position]? [/]______________

FM003. What was the type of your work unit at [preload: retirement /receding position]? [/]
(1) Government
(2) Institutions
(3) NGO （）
(4) Firm
(5) Individual
(6) Other

FM004. Where is this work unit located?
(1)this village/community /
(2)other village/community in this county/city //
(3)Another county/city in this province/_________(FM004_1)city_________ (FM004_2) county
(4)Another province ______(FM004_3)province //_________(FM004_4)city_______(FM004_5) county

[IWER: Mark 0 if there is no fixed address. 0]
[IWER: Choose from the list of provincessee appendix 2]

If FB012 =1（Receding, ask FM005 -FM009 .FB012 =1（）FM005-FM009

FM005. In what month and year did you recede from your position? ______
(FM005_1)1900..2011Year_______(FM005_2)0..12 Month
[IWER: Mark the year using four digits. Take down the month as its actual number. For example, write January as 1 not 01, December as 12. If do not remember month, fill 0. : 41101,12120]

FM006. What was the main reason you receded from your position?

（3） Due to poor health, I couldn't continue my work any more, at the same time I wasn't eligible for retirement
（4） Years of eligible work are less than threeand time of stopping work due to diseases or injures not related to work are more than one year
（5） I'm recruit worker within 6 months, but I had serious chronic disease once and can't tstick to work any more
（6） I receded from my position voluntary
（7） Reach retirement age, but not eligible working age.
（8） Other

FM007. Your pre-receding total salary was _______Yuan a month (including basic wage, bonus, et. al).
________/,

FM008. Did you receive any payments for leaving your job
（1）Yes
（2）No

FM009. If FM008 =1 ask: FM008
How much was the compensation?_ _______ Yuan
?_______

FM010. Are you currently receiving pension? （）
（1）Yes skip to FM018 FM018
（2）No

Skip to FM042 FM042

If FB011 =1（Retirement）, ask FM011- FM041FB011 =1（）FM011- FM041

FM011. Was your retirement normal retirement; early retirement; or internal retirement initially, followed by regular retirement? ,
(1) Normal retirement
(2) Early retirement
(3) Internal retirement first, then regular retirement
(4) Internal retirement, but not yet regular retirement

FM012. Did you retire as a worker or as a cadre?
(1) Worker
(2) Cadre

[IWER: If year of first job was before 1952 (compute from FB001), then ask: 1952]
FM013. Are you an ordinary retiree or revolutionary retiree?
(1) Ordinary retiree
(2) Revolutionary retiree
F1

If FM011 =1 or 2, ask FM014 -FM024 :FM011 =1 or 2FM014 -FM024 ;
FM014. In what month and year did you take [preload: normal/early] retirement? [/]______ 1900..2011 (FM014_1)Year_______0..12 (FM014_2) Month
[IWER: Mark the year using four digits. Take down the month as its actual number. For example, write January as 1 not 01, December as 12. If do not remember month, fill 0. : 41101,12120]
Soft Check: Prompt for Verification/Correction if Age of Early Retirement is Young, e.g.

Prompt if ((FM014_1 +FM014_2 /12)-(CV009_a+CV009_b/12) <45 & CV004==2) | ((FM014_1 +FM014_1 /12)-(CV009_a+CV009_b/12) <50 & CV004==1)

Ask if FM011 =2 (early retirement)
FM015. What was the main reason you processed early retirement?
(1) I have 30 years of job experience, which is enough for early retirement. 30
(2) My work unit belonged to the category of high-risk and hard manual labor and thus was eligible for offering early retirement
(3) My work unit was restructuring / bankrupt, so it offered early retirement
(4) Due to poor health
(5) Due to family reason
(6) Other

FM016. Your pre-retirement salary was _______Yuan a month (including bonus and subsidy, et. al). ________/

FM017. [IWER: If R is unwilling to answer or does not remember, ask unfolding bracket questions here.]: 500 /1,000 /2,500 /5,000 /10,000 yuan

FM018. In what month and year did you start to receive you pension benefits?
()_____(FM018_1)1900..2011Year_____(FM018_2)0..12
[IWER: Mark the year using four digits. Take down the month as its actual number. For example, write January as 1 not 01, December as 12. If do not remember month, fill 0. : 41101,12120]

FM019. Are you receiving pension benefits from the government, a previous work unit, or a social insurance agency? (Choose all that apply) () ()
(1) Government
(2) work unit
(3) Social insurance agency
(4) Commercial insurance company
(5) Other

FM020. How much were the benefits (including subsidy) when you retired? ()
____________ Yuan per month

FM021. [IWER: If R is unwilling to answer or does not remember, ask unfolding bracket questions here.]: 500 /1,000 /2,000 /3,500 /5,000 yuan

FM022. What is your monthly pension (including subsidy)? ()
____________ Yuan
Soft Check: Verify if monthly benefits are low or high, e.g., prompt if FM022< 200 yuan/month

FM023. [IWER: If R is unwilling to answer or does not remember, ask unfolding bracket questions here.]: 500 /1,000 /2,000 /3,500 /5,000 yuan

FM024. How many years of eligible work did you have at the time of retirement?
___________0.00..100.00Years

Skip to FM042 FM042

[PROCEDURE: Ask (FM025 – FM029) if FM011 =3 or 4 (internal retirement). FM011 =3 or 4FM025–FM029]
FM025 In what month and year did you take internal retirement? ______ (FM025_1)1900..2011Year_______(FM025_2) 0..12Month
[IWER: Mark the year using four digits. Take down the month as its actual number. For example, write January as 1 not 01, December as 12. If do not remember month, fill 0. : 41101,12120]
Soft Check: Prompt for Verification/Correction if Age of Retirement is Young, e.g.

Prompt if (FM030_1 +FM030_2 /12)-(CV009_a+CV009_b/12) <45 & CV004==2) | ((FM030_1 +FM030_2 /12)-(CV009_a+CV009_b/12) <50 & CV004==1)

FM026. What was the main reason you processed internal retirement?
(1) 5years less than the legal retirement age 5
(2) My work unit was restructuring / bankrupt
(3) Due to poor health
(4) Due to family reason
(5) Other

FM027. Your pre-internal retirement salary was _______Yuan a month everything included. ?_______/

FM028. How much was the internal retirement wage (everything included) when you processed internal retirement?
____________ Yuan per month /

FM029. [IWER: If R is unwilling to answer or does not remember, ask unfolding bracket questions here.]: 500 /1,000 /2,000 /3,500 /5,000 yuan

Ask(FM030– FM036) if FM011 =3:FM011 =3(FM030 – FM036)
FM030. In what month and year did you process formal retirement? ______ 1900..2011 (FM030_1)Year_______0..12 (FM030_2) Month
[IWER: Mark the year using four digits. Take down the month as its actual number. For example, write January as 1 not 01, December as 12. If do not remember month, fill 0. : 41101,12120]
Soft Check: Prompt for Verification/Correction if Age of Retirement is Young, e.g.
Prompt if ((FM030_1 +FM030_2 /12)-(CV009_a+CV009_b/12) <45 & CV004==2) | ((FM030_1 +FM030_2 /12)-(CV009_a+CV009_b/12) <50 & CV004==1)

FM031. How much were the benefits (including subsidy) when you formally retired? （） ___________Yuan per month /

FM032. [IWER: If R is unwilling to answer or does not remember, ask unfolding bracket questions here.]: 500 /1,000 /2,000 /3,500 /5,000 yuan

FM033. Are you receiving pension benefits from the government, a previous work unit, or a social insurance agency?
（1） Government
（2） Work unit
（3） Social insurance agency
（4） Commercial insurance company
（5） Other

FM034. What is your monthly pension?
____________ Yuan
Soft Check: Verify if monthly benefits are low or high, e.g., prompt if FM034 < 200 yuan/month or FM034 >5000

FM035. [IWER: If R is unwilling to answer or does not remember, ask unfolding bracket questions here.]: 500 /1,000 /2,000 /3,500 /5,000 yuan

FM036. How many years of eligible work did you have at the time of formal retirement? ______________ 0.00..100.00Years

Skip to FM042 FM042

Ask(FM037 – FM040) if FM011=4:FM011=4 FM037- FM040
FM037. In what month and year are you going to process formal retirement? ______ 1900..2011 (FM037_1)Year_______ 0..12 (FM037_2)Month
[IWER: Mark the year using four digits. Take down the month as its actual number. For example, write January as 1 not 01, December as 12. If do not remember month, fill 0. : 41101,12120]
Soft Check: Verify if Age of Respondent will be outside the legal retirement range, e.g.,

Prompt for verification if ((FM037_1 +FM037_2 /12)-(CV009_a+CV009_b/12)<50 | (FM037_1 +FM037_2 /12)-(CV009_a+CV009_b/12)>55) & CV004==2) | (FM037_1+FM037_2 /12)-(CV009_a+CV009_b/12)<55 | (FM037_1 +FM037_2 /12)-(CV009_a+CV009_b/12)>60) & CV004==1)

FM038 How much are the benefits going to be when you formally retire? ____________(FM038_1) Yuan per month / or _________0.00..100.00 (FM038_2) % of salary before retirement _____%
Soft Check: Verify if monthly benefits are low or high, e.g., prompt if FM038_1 < 200 yuan/month or FM038_2 >5000
[Also compare with current wage]

FM039. [IWER: If R is unwilling to answer or does not remember, ask unfolding bracket questions here.]: 500 /1,000 /2,000 /3,500 /5,000 yuan

FM040. How many years of eligible work will you have at the time of retirement? _____________0.00..100.00Years

FM041. How many years of eligible work do you currently have? __________ 0.00..100.00years
[FM041 > FM040]

FM042. Did you have a spouse when you processed [preload: normal retirement / early retirement / internal retirement/receding position]? [///]
(1) Yes
(2) No → Skip to to FM047 FM047

FM043. How was your health at the time of your [preload: normal retirement / early retirement / internal retirement/receding position], excellent, very good, good, fair or poor? [///]?
(1) Excellent
(2) Very good
(3) Good
(4) Fair
(5) Poor

FM044. Had your spouse already processed retirement when you processed [preload: normal retirement / early retirement / internal retirement/receding position]? [///]
(1) Yes
(2) No

FM045. What kind of economic activities was your spouse engaged in at the time of your [preload: retirement /receding position]? [/]
(1) Employed by another person or company and received a wage
(2) Ran own business

(3) Non-employed and looking for a job
(4) Non-employed and not looking for a jobor only doing household work ,
(5) Farming

FM046. How was your spouses health at the time of your [preload: normal retirement / early retirement / internal retirement/receding position], excellent, very good, good, fair or poor? [///]?
(6) Excellent
(7) Very good
(8) Good
(9) Fair
(10) Poor

FM047. Was your father alive at the time of your [preload: normal retirement / early retirement / internal retirement/receding position]? [///]
(1) Yes
(2) No Skip to FM049 FM049

FM048. How about the health of your father at the time of your [preload: normal retirement / early retirement / internal retirement/receding position], excellent, very good, good, fair or poor? [///]?
(1) Excellent
(2) Very good
(3) Good
(4) Fair
(5) Poor

FM049. Did your mother alive when your [preload: normal retirement / early retirement / internal retirement/receding position]? [///]
(1) Yes
(2) No Skip to FM051 FM051

FM050. How about the health of your mother at the time of your [preload: normal retirement / early retirement / internal retirement/receding position], excellent, very good, good, fair or poor? [///]?

(1) Excellent
(2) Very good
(3) Good
(4) Fair
(5) Poor

FM051. How many grandchildren below age 6 did you have at the time of your [preload: normal retirement / early retirement / internal retirement/receding position]? [///]_____ __0..50persons
[IWER: if none, fill '0'. 0]

[PROCEDURE: If R is currently not working（FA001 =2 & FA002 =2 &FA003=2）, ask FM052 . （FA001 =2 & FA002 =2 & FA003 =2）FM052

FM052. Did you work after you processed [preload: normal retirement / early retirement / internal retirement/receding position]? We consider any of the following activities to be work: agricultural work, earn a wage, run your own business and unpaid family business work, et. al. Work does not include doing your own housework or doing activities without pay, such as voluntary work.[///]?
(1) Yes
(2) No

[PROCEDURE: If FM052=1 or If R is currently working, ask FM053). （FM052 =1 or FA001 =1 or FA002 =1or FA003 =1）FM053]

FM053. After you processed [preload: normal retirement / early retirement / internal retirement/receding position] How long did you start to work again? [///]
____ 0.00..100.00Years (allow for decimal points)

Skip to FN001 FN001

［IWER: If R is not working [FA001 =2 & FA002 =2 & FA003 =2], ask the following(FM054 – FM059)］
FM054. Are you currently engaged in paid small pastime work?
(1) Yes
(2) No → Skip to FN001 FN001

FM055. What kind of pastime job are you engaged in? ?

____

FM056. When did you start this job?
___1900..2011（FM056_1） Year___ 0..12（FM056_2）Month
[IWER: Mark the year using four digits. Take down the month as its actual number. For example, write January as 1 not 01, December as 12. If do not remember month, fill 0. : 41101,12120]

FM057. How many days per week do you usually work for your pastime job? An average of _____ days per week ____ 0..7

FM058. How many hours per week do you usually work at your pastime job? An average of _____ hours per week ___0.00..168.00

FM059. What is your monthly income from the pastime work?
______ YUAN
[IWER: Mark 0 if there is no net income, and mark 999997 if running a deficit. 0999997]
Skip to FN001 FN001
End of Retirement

FN PENSION INSURANCE

[Intro: Next well ask you some questions about your pension insurance. Its important to access existing pension policy and revise it in the future.]
[:]

FN001. Are you currently receiving at least one kind of pension as followings? Pension here refers to income from such pension programs as supplemental pension insurance of the firms, residents'pension insurance, rural pension insurance, Urban residents pension and commercial pension insurance, and pension subsidy for the oldest old and so onexcluding wages from government and institutionsbasic pension insurance provided by the firms. （）（）
(1) Yes
(2) No Skip to procedure before FN024 FN024
F1（1）
（2）
（3）1992
（4）
（5）
（6）（）60,

[Show Card 23]

FN002. What type(s) of pension do you receive? (choose all that apply) （） （）
[IWER: Read out all the choices.]
（1） Supplemental pension insurance of the firm Skip to FN003 FN003
（2） Commercial pension →Skip to FN006 FN006
（3） Rural pension →Skip to FN009 FN009
（4） Residents pension →Skip to FN009 FN009
（5） Urban residents pension →Skip to FN009 FN009
（6） Pension subsidy to the oldest old （） Skip to FN017 FN017
（7） Other ___(FN002_1) Skip to FN020 FN020

F1（1）
（2）
（3）1992
（4）
（5）
（6）（）60,

[PROCEDURE: Ask FN003-FN005 if the respondent is receiving pension benefits from supplemental pension insurance of the firms FN002 =1. FN003 -FN005 .]

FN003. In what month and year did you start to receive pension benefits from supplement pension insurance of the firms? ______(FN003_1)1900..2011Year_______(FN003_2) 0..12Month
F1
[IWER: Mark the year using four digits. Take down the month as its actual number. For example, write January as 1 not 01, December as 12. If do not remember month, fill 0. : 41101,12120]

FN004. What are your monthly benefits?
___________ Yuan per month/

FN005. [IWER: If R is unwilling to answer or does not remember, ask unfolding bracket questions here.]:
500 /1,000 /2,000 /3,500 /5,000 yuan

Skip to FN024 if the respondent does not receive money fromother pension programs（FN002_2 =.& FN002_3 =.& FN002_4 =.&FN002_5 =.&FN002_6 =. FN002_7 =.&)（ FN002_2 =.& FN002_3 =.& FN002_4 =.&FN002_5 =.&FN002_6 =. FN002_7 =.&）FN024 .

Ask FN006 -FN008 if the respondent is receiving commercial pension benefits FN002 =2. FN002 =2FN006 -FN008
FN006. In what month and year did you start to receive commercial pension benefits? ______1900..2011（FN006_1） Year_______ 0..12（FN006_2）Month
[IWER: Mark the year using four digits. Take down the month as its actual number. For example, write January as 1 not 01, December as 12. If do not remember month, fill 0. : 41101,12120]

FN007. What is your monthly benefit?
____________ Yuan per month/

FN008. [IWER: If R is unwilling to answer or does not remember, ask unfolding bracket questions here.]:
500 /1,000 /2,000 /3,500 /5,000 yuan

Skip to FN024 if the respondent does not receive money from other pension programs（FN002_3 =.& FN002_4 =.&FN002_5 =.&FN002_6 =. FN002_7 =.&）（FN002_3 =.& FN002_4 =.&FN002_5 =.&FN002_6 =. FN002_7 =.&）FN024

Ask FN009 -FN014 if the respondent is receiving rural pension benefits FN002 =3 or 4 or 5 FN002 =3 4 5FN009 – FN014

FN009. Have you ever contributed to your [preload FN002]? [FN002][CAPI: If FN002 =4, prompt: If your residents pension was transferred from other pension programs, your contribution to these other programs also counts.FN002 =4]

(1) Yes
(2) No Skip to FN012 FN012

FN010. In what month and year did you start to contribute to your rural pension? ______(FN010_1) 1900..2011Year______(FN010_2)0..12Month
[IWER: Mark the year using four digits. Take down the month as its actual number. For example, write January as 1 not 01, December as 12. If do not remember month, fill 0. : 41101,12120]

FN011. Your annual contribution was____Yuan, annual subsidy from the collective was____yuan, annual subsidy from the government was____Yuan; or your lump sum contribution was____Yuan, lump sum subsidy from the collective was_____Yuan, lump sum subsidy from the government was_____Yuan ____(FN011_1)_____(FN011_2)______(FN011_3)____(FN011_4)______(FN011_5)_____(FN011_6)

FN012. In what month and year did you start to receive your [preload FN002]? [F228]________ (FN012_1)1900..2011Year _____(FN012_2)0..12Month
[IWER: Mark the year using four digits. Take down the month as its actual number. For example, write January as 1 not 01, December as 12. If do not remember month, fill 0. : 41101,12120]

FN013. How much do you receive now? (as an amount per month?) __________ Yuan per month /

FN014. [IWER: If R is unwilling to answer or does not remember, ask unfolding bracket questions here.]: 500 /1,000 /2,000 /3,500 /5,000 yuan

Ask FN015 -FN016 if the respondent is receiving residents pension FN002 =4.FN002 =4FN015 – FN016

FN015. Was your residents pension transferred from other pension insurances like rural pension and basic pension of the firms?

（1）Yes
（2）No Skip FN016 FN016

F1（1）1992
（2）

FN016. Which of the following pension program was your residents pension transferred from?
（1） Rural pension
（2） Basic pension of the firms
（3） Other _________(FN016_1)

F1（1）1992
（2）

[PROCEDURE: Skip to FN024 if the respondent does not receive money fromother pension programs（FN002_6 =. FN002_7 =.&）. （FN002_6 =.& FN002_7 =.）FN024]

[PROCEDURE:Ask FN017 -FN019 if the respondent is receiving pension subsidy for the oldest old FN002 =6. FN002 =6FN017 – FN019]

FN017. In what month and year did you start to receive the pension subsidy for the oldest old? ________ (FN017_1)1900..2011Year _____(FN017_2)0..12Month
F1（）60,
[IWER: Mark the year using four digits. Take down the month as its actual number. For example, write January as 1 not 01, December as 12. If do not remember month, fill 0. : 41101,12120]

FN018. How much do you receive now? ____ ______Yuan per month /

FN019. [IWER: If R is unwilling to answer or does not remember, ask unfolding bracket questions here.]: 500 /1,000 /2,000 /3,500 /5,000 yuan

[PROCEDURE:Skip to FN024 if the respondent does not receive money fromother pension programs （FN002_7 =.）. （FN002_7 =.）FN024]

[PROCEDURE: Ask FN020 -FN023 if the respondent is receiving other pension benefits FN002 =7 . FN002=7FN020 – FN023]
FN020. You just told us you are receiving benefits from other pension program, what is the name of this pension program? （） __ ______

FN021. In what month and year did you start to receive this pension benefits? （） ________(FN021_1) 1900..2011Year ____(FN021_2)0..12Month
[IWER: Mark the year using four digits. Take down the month as its actual number. For example, write January as 1 not 01, December as 12. If do not remember month, fill 0. : 41101,12120]

FN022. How much do you receive now? ________Yuan per month /

FN023. [IWER: If R is unwilling to answer or does not remember, ask unfolding bracket questions here.]: 500 /1,000 /2,000 /3,500 /5,000 yuan

Skip to FN024 FN024

[PROCEDURE: If FB011 =2ask FN024 -FN037 , FB011 =2 （） FN024-FN037]

FN024. Are you enrolled in pension program of the government and institutions or basic pension of the firms?
(1) Yes, pension program of the government and institutions
(2) Yes, basic pension insurance of the firms
(3) No Skip to FN038 FN038
F1

FN025. From which of the following work units did you get the pension insurance you just told us[preload pension program of government and institutions if FN024 =1; or preload Basic pension insurance of the firms if FN024 =2]?[FN024 =1,FN024 =2,]
[[IWER: Choose (4) None of the above if the respondent has no work unit （4）]
(1) Current work unit [preload FD003] FD003 [FD003] Skip to FN029 FN029
(2) Last work unit [preload FL005] FL005 [FL005] Skip to FN029 FN029
(3) The work unit that processed retirement for the respondent [preload FM002] FM002 [FM002] Skip to FN029 FN029
(4) None of the above

If answer to FN025 =4 is no, then ask FN026 – FN028: FN025 =4FN026 -FN028
FN026. What is the name of the unit that provides you this pension insurance? ?____
____

FN027. What was the type of the unit that provides you the pension insurance?
(1) Government
(2) Institutions
(3) NGO （）
(4) Firm
(5) Individual
(6) Other

FN028. In which province and county/city is this work unit located?
_______(FN028_1)Province//_______(FN028_2) city_______(FN028_3) county

Ask FN029 -FN031 only if the respondent have participated in basic pension insurance of the firms (FN024 =2) FN029 -FN031

FN029. In what month and year did you start to participate in the basic pension insurance of the firms through this work unit? _____(FN029_1)1900..2011Year_____(FN029_2)0..12Month
F1
[IWER: Mark the year using four digits. Take down the month as its actual number. For example, w rite January as 1 not 01, December as 12. If do not remember month, fill 0. : 41101,12120]

FN030. Before the above mentioned time, have you ever participated in basic pension insurance of the firms through other work unit for more than ten years? 10
（1）Yes
（2）No Skip to FN032 FN032
F1

FN031. Among all of work units that you have participated in basic pension insurance of the firms f or more than 10 years, in which province was the lastest work unit located? 10_____ Province//
F1

FN032. For how many years altogether have you been included in this program? [Include years with other employers if the same plan.] （） []_______ 0.00..100.00Years

FN033. For how many years altogether will you have been included in this program when you retire? [Include years with other employers if the same plan.] （） [] ________0.00..100.00Years

FN034. Will this be enough years to receive pension?
(1) Yes →Skip to FN036 FN036
(2) No →Skip to FN035 FN035

FN035. What do you plan to do?
(1) I will pay the remaining premiums all in one payment at retirement to qualify for pension →Skip to FN036 FN036
(2) I will receive a one-time payment at retirement and not get pension
(3) I will not receive pension

If R answered yes to pension [FN034 =1 or FN035 =1], ask:
FN036. About how much do you expect your benefits to be? (as a percentage of your pay at retirement, or as an amount per month or year?)
_________ （FN036_1）Yuan per month/
Or _________ 0.00..100.00（FN036_2）% of final pay

FN037. [IWER: If R is unwilling to answer or does not remember, ask unfolding bracket questions here.]: 500 /1,000 /2,000 /3,500 /5,000 yuan

Ask all respondents,

FN038. Are you enrolled in at least one kind of pension programs as lists? Pension programmes here includes supplemental pension of the firms, commercial pension insurance, rural pension, residents pension and so onexcluding pension insurance of government and institutions, basic pension of the firms.
（）
(1) Yes
(2) No Skip to FN071 FN071
F1（1）
（2）
（3）1992
（4）

FN039. What type(s) of pension insurance do you have? (Choose all that apply) （）
[IWER: Read out all the choices.]
（1） Supplemental pension insurance of the firm Skip to FN040 FN040
（2） Commercial pension Skip to FN050 FN050
（3） Rural pension Skip to FN060 FN060
（4） Residents pension Skip to FN060 FN060
（5） Urban residents pension Skip to FN060 FN060
（6） Other pension Skip to FN067 FN067
F1（1）
（2）
（3）1992
（4）
（5）

Supplementary enterprise pension

[PROCEDURE: If FN039 =1 ask FN040 –FN049. FN039 =1FN040 –FN049]
FN040 . Next are questions about your supplementary enterprise pension. What type of retirement pension plan is/was your employers supplement pension?
(1) Defined Benefit (DB) Retirement Pension
(2) Defined Contribution (DC) Retirement Pension
F1

DB Retirement Pension Plan: A worker's retirement pension is determined in advance and the amount paid by the user shall change based on how well the savings are managed.
DC Retirement Pension Plan: The amount paid by the user is determined in advance and the retirement pension paid to a worker shall change based on how well the savings are managed.

FN041. For how many years altogether have you been included in this plan? [Include years with other employers if the same plan.] []______ 0.00..100.00 Years

FN042. At what age do you expect to start receiving benefits from this plan?
(1) At age _______0..120 (FN042_1) or in ______0..120 (FN042_2) years
(2) I do not expect receiving these benefits because I have received cash settlements → Skip to FN049 FN049
(3) I do not expect receiving these benefits because I have lost benefits → Skip to FN050 FN050
(4) Other → Skip to FN050 FN050

FN043. What is the combined monthly contribution from you and your employer? Of which, how much do you pay? _______ （FN043_1）Yuan
_____ 0.00..100.00（FN043_2）%

If FN040 =1ask:FN040 =1
FN044. For a DB plan, do you know how much you are entitled to at (age in EP226)? [preload EP226]
_________(FN044_1) Yuan per month /
Or _______0.00..100.00 (FN044_2) % of retirement wage %
Or _________ (FN044_3)Yuan （Lump sum amount ）

FN045. [IWER: If R is unwilling to answer or does not remember, ask unfolding bracket questions here.]:
500 /1,000 /2,000 /3,500 /5,000 yuan

Skip to FN050 . FN050 .

If FN040 =2 ask: FN040 =2
FN046. For a DC plan, have you ever checked your account balance?

(1) Yes ______ （FN046_1）Yuan in ______ 1900..2011（FN046_2）Year _______0..12 （FN046_3） Month
[IWER: Mark the year using four digits. Take down the month as its actual number. For example, write January as 1 not 01, December as 12. If do not remember month, fill 0. : 41101,12120]
(2) No

FN047. What is the earliest age at which you could leave this employer and start to receive pension benefits? ________ 45..120Years old

FN048 . By how much would your pension be reduced from full benefits if you left this job at (AGE IN FN047)? [preload FN047]By ________ 0.00..100.00（FN048_1）% or _______ （FN048_2）Yuan
Skip to FN050 FN050

FN049. How much cash settlements did you receive?
_______________ Yuan

Skip to FN080 if the respondent participates in no other pension programs (FN039_2 =.&FN039_3 =.&FN039_4 =.&FN039_5 =.&FN039_6 =.)(FN039_2 =.&FN039_3 =.&FN039_4 =.&FN039_5 =.&FN039_6 =.)FN080

Commercial Pension

[PROCEDURE: If FN039 =2 ask FN050 -FN059 . FN039 =2FN050 -FN059]
FN050 . Next are questions about your Commercial Pension. Who paid for the commercial pension insurance?
(1) Myself
(2) My employer
(3) My family or relative
(4) Other person
F1

FN051. When did you start paying for the commercial pension?
___（FN051_1） 1900..2011Year___0..12（FN051_2） Month
F1
[IWER: Mark the year using four digits. Take down the month as its actual number. For example, write January as 1 not 01, December as 12. If do not remember month, fill 0. : 41101,12120]

FN052. How do you contribute to the commercial pension? ?
(1) Annual payment
(2) Lump sum amount Skip to FN055 FN055

FN053. You contribute _______yuan/ year to the commercial insurance _______Yuan/ year/

FN054. How many years do you need to pay? ?_________Years
Soft Check: Prompt for verification if greater than a legal maximum
[PROCEDURE: Skip FN055 if FN052 =1. FN052 =1, FN055]
FN055. How much premium do you need to pay in total? _________Yuan

FN056. How do you receive the pension?
(1) Lump sum amount SkipFN057 and FN058 FN057 -FN058
(2) Yearly
(3) Monthly

If FN056 =2 ask FN057
FN057. How much do you expect to receive _______ yuan/ year after your retirement? _________ Yuan/year/

Soft Check: Prompt for verification if low or high, e.g. FN057 < 1200 per year or FN057 > 60000 per year

If FN056 =3 ask FN058
FN058. How much do you expect to receive _______ yuan/month in the future? _________ Yuan/ month/
Soft Check: Prompt for verification if low or high, e.g. FN058 < 100 per month or FN058 > 5000 per year
FN059. How much do you expect to receive _______ yuan in total? ________ Yuan

Skip to FN080 if the respondent participates in no other pension programs (FN039_3 =.&FN039_4 =.&FN039_5 =.&FN039_6 =.)(FN039_3 =.&FN039_4 =.&FN039_5 =.&FN039_6 =.)FN080

[PROCEDURE: If FN039 =3 or 4 or 5 ask FN060 -FN064. FN039 =345FN060-FN064]

FN060. In what month and year did you start to pay for your [preload FN039]?
[FN039]________(FN060_1)1900..2011 Year ____(FN060_2)0..12Month

[IWER: Mark the year using four digits. Take down the month as its actual number. For example, write January as 1 not 01, December as 12. If do not remember month, fill 0. : 41101,12120]

FN061. Your annual contribution is _____ Yuan, annual subsidy from the collective is ______ Yuan, annual subsidy from the government is ____ Yuan_____（FN061_1 ）_____（FN061_2）___(FN061_3)

FN062. When do you expect to receive pension
At age _______45..120（FN062_1）years old or in ______0.00..100.00（FN062_2） years

Soft Check: Prompt or Verify if FN062_1 <50 or FN062_2 >60

FN063. About how much do you expect your benefits to be? (as an amount per month or year or a lump sum?)
____________ （FN063_1）Yuan per month/
Or _________ （FN063_2）Yuan （Lump sum amount ）
[Soft Check: Prompt or Verify if These Benefits are Low or High, e.g., FN063_1 <100 | FN063_1 >5000 per month.]

FN064. [IWER: If R is unwilling to answer or does not know, ask unfolding bracket questions here.] 250 / 500 / 1000 / 2000

Ask FN065 and FN066 if the respondent is enrolled in residents pension FN039 =4 FN039 =4FN065 FN066

FN065. Was your residents pension transferred from other pension insurances like rural pension and basic pension of the firms?
(1) Yes
(2) No Skip FN066 FN066
F1（1）1992
（2）

FN066. Which of the following pension program was your residents pension transferred from?
(1) Rural pension
(2) Basic pension insurance of the firms
(3) Other __________(FN066_1)
F1（1）1992
（2）

Skip to FN080 if the respondent participates in no other pension programs (FN039_6 =.) (FN039_6 =.)FN080

Ask FN067 -FN068 if the respondent is enrolled in other pension program FN039 =6 FN039 =6FN067 -FN068

FN067 . You just told us you are receiving benefits from other pension program, what is the name of this pension program? ________

FN068. When do you expect to receive pension
At age _______(FN068_1)45..120 or in ______(FN068_2)years

FN069. About how much do you expect your benefits to be? (as an amount per month or year or a lump sum?)
____________(FN069_1) Yuan per month/
Or _________ (FN069_2)Yuan （Lump sum amount ）

FN070. [IWER: If R is unwilling to answer or does not know, ask unfolding bracket questions here.] 250 / 500 / 1000 / 2000

New Rural Social Pension Insurance

FN071. Do you participate in the New Rural Social Pension Insurance program?
(1) Yes →Skip to FN073 FN073
(2) No

[Procedure: Ask FN072 if FN071 =2, and then end this part FN071=2FN072]

FN072. Why dont you participate in the New Rural Social Pension Insurance program? (Circle all th at applies) （）
（1） The New Rural Social Pension has not been introduced in my local area(FN072_1)
（2） I lack money(FN072_2)
（3） I am not satisfied with the benefits because it isnt cost-effective. (FN072_3)
（4） The benefits are poor, and mean nothing to my life. (FN072_4)
（5） Application and payment arrangements make it inconvenient to participate (FN072_5)
（6） The mechanism for making contributions is not justifiable. (FN072_6)
（7） I do not have a local Hukou. (FN072_7)
（8） I am already covered by other social pension insurance. (FN072_8)
（9） Other (FN072_9)

→Skip to FN080 FN080

FN073. In what month and year were you first covered by New Rural Social Pension Insurance? （）
___(FN073_1)2008..2011Year ___(FN073_2)0..12Month

[IWER: Mark the year using four digits. Take down the month as its actual number. For example, wr ite January as 1 not 01, December as 12. If do not remember month, fill 0. : 41101, 12120]

FN074. How do you contribute to New Rural Pension Insurance?
（1） Annual payment ___(FN074_1) Yuan/Year /
（2） Lump sum amount ___(FN074_2)Yuanequals to annual paymentFN074_3___ Yuan/Year/
→ Skip to FN076 FN076
（3） I dont need to pay myself. I am over 60 years old, and I am covered through my childrens p articipation in the New Rural Social Pension Insurance. 60 →Skip to FN077 FN077

FN075. How many years do you need to pay to receive benefits? ? __Years

FN076. Who pays for your participation in New Rural Social Pension Insurance? ?
(1) Myself
(2) My children
(3) Other family member or relative
(4) Others

FN077. Did you start to receive pension benefit? （）
1. Yes
2. No →Skip to FN080 FN080

FN078. In what month and year did you start to receive pension benefits? ___(FN078_1)2008..2011Year ___(FN078_2)0..12Month
[IWER: Mark the year using four digits. Take down the month as its actual number. For example, write January as 1 not 01, December as 12. If do not remember month, fill 0. : 41101,12120]

FN079. How much do you receive every month? ____ Yuan/ month/

Ask all R
FN080. Whom do you think you can rely on for old-age support
(1) Children
(2) Savings
(3) Pension or retirement salary
(4) Commercial pension insurance
(5) Other
F1

End of Pension

FN081. IWER: How often did the respondent receive assistance in answering section Section E－Work?
[IWER: If it is answered by a proxy, please record the respondents reaction.]E[]
(1) Never
(2) A few times
(3) Most or all of the time
(4) The section was completed by a proxy respondent（the respondent is absent） → Skip to FN082 FN082

FN082. [IWER: What is the proxys relationship to R? If unknown, please ask the proxy.]
What is your relationship to R?
(1) Spouse
(2) Mother
(3) Father
(4) Mother-in-law /
(5) Father-in-law /
(6) Sibling
(7) Brother-in-law, sister-in-law /
(8) Child
(9) Spouse of child
(10) Grandchild
(11) Other relative
(12) Helper or other non-relative

FN083.[IWER: Please record the reason for proxy]
What is the main reason for proxy (the respondent is absent)

（1）The respondent has serious physical handicaps _
（2）The respondent has serious mental handicaps
（3）The respondent has rejected this interview.
（4）Other____. (FN083_1)

G &H INCOME, EXPENDITRUES AND ASSETS

G1 VIGNETTS ON INCOME

[For main respondent only FA006---FA008] The following vignette questions are on subjective inequality [G001 ---G003]:
[Show Card 24]

G001. Mr. Wang owns an apartment in a large city. He lives with his wife and father, who is retire d. He and his wife earn 3000 yuan per month. Overall, how would you rate the standard of living of Mr. Wang's family? Is it very high, relatively high, average, relatively poor or poor? 3000

(1) Very high
(2) Relatively high
(3) Average
(4) Relatively poor
(5) Poor

G002. Mr. Zhang works as a migrant and earns 1000 yuan per month.His wife and father live in a rural village. Their land can produce 2000kg of grain each year, and they also raise 2 pigs. Overall, how would you rate the standard of living of Mr. Wang's family? Is it very high, relatively high, average, relatively poor or poor? 10002000

(1) Very high
(2) Relatively high
(3) Average
(4) Relatively poor
(5) Poor

G003. Overall, how would you rate your own standard of living? Is it very high, relatively high, aver age, relatively poor or poor?

(1) Very high
(2) Relatively high
(3) Average
(4) Relatively poor
(5) Poor

G2 HOUSEHOLD INCOME AND EXPENDITURES

[IWER: Except for Part 1_1, this section is asked of the financial respondent, from questionCV031 Do not allow a proxy respondent to answer the entire section. Part 1_1（）CV031]

Part 1: Household Wage Income and Individual-based transfers

Part 1_1: Main Respondent and Spouses Wage Income and Individual-based transfers

[IWER: Please conduct Part 1_1 when the main respondent and spouse are at home. Dont allow a proxy to complete the part. Part 1_1]

GA001 . Did you receive any wage and bonus income in the past year? （）

(1) Yes
（2）No skip to GA003 _GA003

GA002. How much did you receive last year? ____yuan or F001_b_1 ____yuan/month

______ __ ____ (GA002_1)

Wage income: yuan [soft check >200,000] or yuan/month [soft check>15,000]

GA002_bracket. [IWER: If R is unwilling to answer or does not remember, ask unfolding bracket questions here.]: 10,000 /30,000 /50,000 /100,000 /200,000 yuan

[Show Card 25]
GA003. Did you receive any of the following types of individual income in the past year? (check all that apply) ( )

(1) Pensions (including wages from government institutions and firms, supplemental pension of the firms,and income from such programs as rural pension insurance, Urban residents pension and commercial pension insurance, and pension subsidy for the oldest old) ( )
(2) unemployment compensation
(3) pension subsidy
(4) Workers compensation from Industrial Accident Compensation Insurance includes wage-replacement benefits, disability benefits, and survivors' benefits
(5) elderly family planning subsidies
(6) medical aid
(7) other government subsidies
(8) social assistance
(9) other income sources (including alimony, child support) ( )
(10) None of the above Skip GA004 GA004

F1

GA004. How much did you receive last year? ____yuan or ___yuan/month
______(GA004_1) _______(GA004_2)

If GA003=1 and GA004=DK,
GA004_bracket. [IWER: If R is unwilling to answer or does not remember, ask unfolding bracket questions here.]: 10,000 /30,000 /50,000 /100,000 /200,000 yuan

Part 1_2: Other Household Members Wage Income and Individual-based transfers

[IWER reminder: make sure others are not present.]
[Intro: Wed like to ask you some questions about the income and assets of OTHER members of your household.]
[IWER: The names of other household members are preloaded from the cover screen information. For each member（excluding main respondent and spouse）, answer the following questions:] [( )]

GA005. Did [preload household member name] receive any wage and bonus income in the past year? [preload household member name] ( )

(2) Yes
（2）No skip to GA007 _GA007

GA006. How much did he/she receive last year? ____yuan or ____yuan/month
______(GA006_1) _______(GA006_2)

Wage income: yuan [soft check >200,000] or yuan/month [soft check>15,000]

GA006_bracket. [IWER: If R is unwilling to answer or does not remember, ask unfolding bracket questions here.]: 10,000 /30,000 /50,000 /100,000 /200,000 yuan

[Show Card 25]
GA007. Did [preload household member name] receive any of the following types of individual income in the

past year? (check all that apply) [preload household member name] （）

(1) Pensions (including wages from government institutions and firms, supplemental pension of the firms,and income from such programs as rural pension insurance, Urban residents pension and commercial pension insurance, and pension subsidy for the oldest old) （）
(2) unemployment compensation
(3) pension subsidy
(4) Workers compensation from Industrial Accident Compensation Insurance includes wage-replacement benefits, disability benefits, and survivors' benefits
(5) elderly family planning subsidies
(6) medical aid
(7) other government subsidies
(8) social assistance
(9) other income sources (including alimony, child support) （）
(10) None of the above Skip GA008 GA008

F1

GA008. How much did he/she receive last year? ____yuan or ____yuan/month
______(GA008_1) [soft check >10000] _______ (GA008_2) [soft check >3000]
If GA007=1 and GA008=DK,
GA008_bracket. [IWER: If R is unwilling to answer or does not remember, ask unfolding bracket questions here.]: 10,000 /30,000 /50,000 /100,000 /200,000 yuan

[PROCEDURE: Skip to next person]

PART 2 HOUSEHOLD AGRICULTURAL INCOME AND EXPENDITURE

[Intro: Next we will ask some questions about your household agricultural income and expenditure.]

GB001. Did your household engage in agricultural work (including cropping, forestry, livestock, and fish) last year? （）
(1) Yes
(2) No skip to GC001 GC001

GB002. [IWER: The names of other household members not including respondent and spouse are preloaded from the cover screen information. [] [preload other household member name] Did [name] engage in agricultural work (including cropping, forestry, livestock, and fish) last year? [name]（）
(1) Yes
(2) No

Crops and forestry products :

GB003. Did your household engage in cropping or forestry last year? （）
(1) Yes
(2) No Skip to GB007 GB007

GB004. When was the most recent harvest?
_______2009..2011 (GB004_1)year ________0..12 (GB004_2)month
[IWER: Mark the year using four digits. Take down the month as its actual number. For example, write January as 1 not 01, December as 12. If do not remember month, fill 0. : 41101,12120]

GB005. What is the value of all crops and forestry products (including sold lumber) produced in the past year? （） ___ Yuan [soft check: 75000 yuan]

GB005_bracket. [IWER: If R is unwilling to answer or does not remember, ask unfolding bracket questions here.]: 1,000 /3,000 /5,000 /7,000 /10,000 yuan

GB006. What was the total cost of producing crops (including vegetables and Chinese herbs) and forestry products in the past year? (including Seeds (including home produced), Fertilizer, Organic fertilizer, Pesticide, Plastic sheets, Hiring labor (including with machine or animals), Land rents, Rents (excluding land rents), Irrigation, Fuel,Transportation, Processing, Marketing (including packaging, management fee)) （）（） [(）（）（）（）) ______ Yuan [soft check: 50,000 yuan]

GB006_bracket. [IWER: If R is unwilling to answer or does not remember, ask unfolding bracket questions here.]: 3,00 /6,00 /1,000 /2000 /5,000 yuan

Livestock and fisheries

GB007. Did your household grow any livestock or aquatic life last year?
(1) Yes
(2) No Skip to GC001 GC001

GB008. What is the current value of all livestock（including chicken, duck, cattle, pig, sheep, etc.）and aquatic life? （） ____Yuan [soft check: 100,000 yuan]

GB008_bracket. [IWER: If R is unwilling to answer or does not remember, ask unfolding bracket questions here.]: 5,00 /1,500 /3,000 /4,500 /9,000 yuan

GB009. What was the value of all livestock and aquatic life at this time last year? ______Yuan [soft check: 100,000 yuan]

GB009_bracket. [IWER: If R is unwilling to answer or does not remember, ask unfolding bracket questions here.]: 5,00 /1,500 /2,500 /4,000 /8,000 yuan

GB010. How much did you spend purchasing new livestock and aquatic life in the past year? ______(Yuan [soft check: 50,000]

GB011. What was the value of all livestock and aquatic life that were sold or consumed in the past year? ? ______Yuan [soft check: 100,000 yuan]

GB011_bracket. [IWER: If R is unwilling to answer or does not remember, ask unfolding bracket questions here.]: 2,00 /9,00 /1,500 /2,500 /5,000 yuan

GB012. What was the value of all livestock products produced（including the self consumption value） in the past year, including milk, wool (including cashmere, sheep or goat skin), and eggs) （）（） ______ Yuan [soft check: 50,000 yuan]

GB012_bracket. [IWER: If R is unwilling to answer or does not remember, ask unfolding bracket questions here.]: 100 /200 /300 /500 /1000 yuan

GB013. What was the cost of producing livestock and aquatic life in the past year, including the value of all feed, medicine, pasture fees, animal pens, wages, etc.) ______ Yuan [soft check: 50,000 yuan]

[soft check: reported raising livestock but no evidence of such activity if GB008 =0 and GB009 ==0 GB008 =0 GB009 =0]

PART 3. Self-employed Activities

GC001. Did your household members engage in any self-employed activities last year?
(1) Yes
(2) No Skip to GD001 GD001

GC002. How many types of activities did your household members participate in the last year? _____ Activities.

For each each self-employed activity in GC002 , ask GC003 - GC005 .
GC003 - GC005

GC003. Who engage in this self-employment businesse in the past year?
[IWER: All the names of household members are preloaded from the cover screen information （）] ()

[Show Card 26]
GC004 . Which types of activities? （）
(1) Services (cooking, sewing, private clinic etc.) （） (GC004_1)
(2) Transportation (GC004_2)
(3) Construction (GC004_3)
(4) Mining (GC004_4)
(5) Processing production (GC004_5)
(6) Business (GC004_6)
(7) Others (GC004_7)

GC005. Not including fixed capital costs, what is your best estimate of the net income earned from this activity by your household members last year?[If the activity was conducted jointly with non-household members, report only the net income earned by household members. Remember to consider the following types of costs: energy, housing or equipment rental, raw materials, transportation, marketing, wages, taxes or fees.] _______ Yuan [soft check: 500,000 yuan]

GC005_bracket. [IWER: If R is unwilling to answer or does not remember, ask unfolding bracket questions here.]: 5,000 /10,000 /50,000 /100,000 /200,000 yuan

PART 4 HOUSEHOLD PUBLIC TRANSFER INCOME

We ask the public transfers received by the households (with household as the unit). Public transfers have characteristic of welfare, such as Wubaohu Subsidy and Tekunhu Subsidy gived by government.

GD001. How much Dibao assistance did your household receive last year? (if not applicable, fill in 0 yuan). （0）
_______________Yuan
F1

[Show Card 27]
GD002. Did your household receive any of the following government subsidies in the past year? (check all that apply) () [soft checks for each category: 20,000 yuan20,000]
(1) Reforestation : how much? ______(GD002_1)Yuan
(2) Agricultural subsidies : how much? ______(GD002_2)Yuan
(3) Wubaohu (targets low-income, blind, disabled, aged persons, and young persons that have no means to support themselves. （: how much?______(GD002_3)Yuan
(4) Tekunhu : how much? ______(GD002_4)Yuan

(5) Work injury subsidies to the immediate family members how much? ______(GD002_5)Yuan
(6) Emergency or disaster relief (jiujikuan, jiuzaikuan) last year? （ ）（ ） : how much? ______(GD002_6)Yuan
(7) Other : how much? ______(GD002_7)Yuan
(8) None **Skip to GD003 GD003**

[Show Card 28]
GD003. Did your household receive any income from the following sources in the past year? (check all that apply) （）
(1) Donations from the society (including cash, and items like food, clothing, etc.)（）: how much? _____(GD003_1)Yuan [soft check: 20,000 yuan]
(2) Compensation for land seizure last? : how much? _______(GD003_2)Yuan [soft check: 100,000 yuan]
(3) Compensation to pulling down your house or apartment last year? (FD003S3)_: how much? ______(GD003_3)Yuan [soft check: 100,000 yuan]
(4) None

PART 5 HOUSEHOLD LIVING EXPENDITURE

GE001. We wish to know your family food expenditure for the last week. Are you the primary person who purchases food for the household?
(1) Yes Skip to GE004 GE004
(2) No

GE002. Who is the primary person purchasing food for the household? [CAPI: Preload all the HHmember list]

[IWER: If possible, the primary person who purchases food for the household should answer the questions about expenditures FE003-FE009][]

GE003. [IWERPlease record who answered these questions.]
(1)Primary person who purchases food for the household （not Financial respondent）（）
(2)Financial respondent
(3)Other

GE004. In the past week, how many people usually ate meals together in your household (not including guests)? （）______Persons [soft check: 10]

GE005.. Last week how many meals did you provide to guests? （）________ meals [soft check: 100]

Food :
[Intro: The next questions are about your household living expenditure, including your household members (preloaded names of household members) living expenditure. If one attends school/work outside and comes home almost every week, GE006 –GE008 includes his/her expenditureon food and meals outside. If one attends school/work outside but not come home every week, GE006 –GE008 excludes his/her expenditureon food and meals outside.]
[（） //]

GE006. In the past week, what was the value of household consumption of food, including both food purchased and food eaten from your own production (excluding eating out expenditure, alcohol, Cigarettes, cigars and tobacco expenditure) ? _______ Yuan [soft check: 6000 yuan]

GE007. Among it, how much did your household spend on eating out? ______Yuan [soft check: 3000 yuan]

GE008 Among it, how much did your household spend on alcohol, Cigarettes, cigars and tobacco? _____

_Yuan [soft check: 3000 yuan]

[Show Card 29]
GE009. Please tell me the expenditure last month for your household for the following items. [soft check for each category: 5000 yuan]
[IWER: fill in 0 if no corresponding expenditure; fill in -9999 if the respondent cannot recall the expenditure.: 0-9999]

	Item	Expenditure (Yuan) （）
1	Communication fees (including post, internet usage, telephone and cell phone usage) （）	(GE009_1)
2	Utilities: Water and electricity	(GE009_2)
3	Fuels (including gas, coal, etc.) （）	(GE009_3)
4	Fees for Matron, housekeepers and servants	(GE009_4)
5	Local Transportation	(GE009_5)
6	Household items and personal toiletries that are used daily plus beauty treatments (e.g., detergent, soap, toothpaste, toothbrush, cosmetics, beauty salon, etc.) （）	(GE009_6)
7	Entertainment (including fees to buy books, newspapers, VCCs, DVDs, going to cinema and bars) （）	(GE009_7)

[Show Card 30]
GE010. In the last year how much did your household spend on the following items?
[IWER: fill in 0 if no corresponding expenditure; fill in -9999 if the respondent cannot recall the expenditure. : 0-9999] [soft check: >=100,000 yuan]

	Item	Expenditure (Yuan) （）
1	Clothing and bedding	(GE010_1)
2	Long distance traveling expenses (including travel fees through train, car, bus, plane and ship) （）	(GE010_2)
3	Heating(centrally heated) ()	(GE010_3)
4	Furniture and consumption of durable goods, includes refrigerator, washing machine, TV and expensive instruments like pianio.	GE010_4)
5	Education and training(including tuition, training fees, etc.) （）	(GE010_9)
6	Medical expenditure （）	(GE010_10)
7	Fitness expenditures （）	(GE010_5)
8	Beauty (including make-ups, facials, massages, etc.) （）	(GE010_6)
9	Purchase, Maintenance and repair (of transportation vehicles, appliances, communication products, etc.) （）	(GE010_7)
10	Taxes and fees turned over to the government （）	（GE010_8）
11	Automobiles	(GE010_11)
12	Electronics (laptops, computers and accessories, vedio games, etc.) （）	(GE010_12)
13	Property management fees (including parking fee) （）	(GE010_13)
14	Donations to the society (including cash, and items like food, clothing, etc.) （）	(GE010_14)

GE011. How often did the respondent receive assistance in answering section Household income and expenditure?
[IWER: If it is answered by a proxy, please record the respondents reaction.]

(1) Never
(2) A few times
(3) Most or all of the time

HA HOUSEHOLD ASSETS

[IWER: This section is asked of the financial respondent, from question CV025. Do not allow a prox y respondent to answer the entire section.]

PART 1 Current Residence

The following questions pertain to your current residence.

HA001. When did your household start to live at your current residence? _________ 1900..2011Year
[IWER: Mark the year using four digits. : 4]

HA002. Do you pay rent for your current residence?
(1) Yes
(2) No Skip to HA007 HA007

HA003. How much rent do you pay each month? ______ Yuan/month/ [soft check: <100, >10,000 yuan]

HA004. Did you pay less than the market rental value?
(1) Yes
(2) No Skip to HA007 HA007

HA005. If you rented the same housing unit from the market, what is the rent per month you would have to pay? ______________ Yuan/Month /[soft check: <100, >10,000 yuan]

HA006. How much of the rent was paid by a housing subsidy from the employer of a household member? ____yuan [soft check >10,000 yuan], which household member?_____[preloaded list] _______(HA006_1)_______(HA006_2) []

HA007. Who owns your current residence?
(1)
(2)
(3) Skip to HA010 HA010

HA008. Which household member(s) own the house? (preloaded names of household members)/_______ []

HA009. What share of the house is owned by (preloaded names of household members)? （HA008 ） ________ 0.00..100.00% [hard check: range 0-100]

Skip to HA011 HA011

HA010. What non-household members own all or part of your current residence? (circle all that apply) （）
(1) working unit of household member, which household member?_____ (HA010_1) [preloaded list][]
(2) government indemnificatory housing
(3) child(non- household member) of main respondent or spouse （）, which child?___(HA010_2) [preloaded list]
(4) parent(non- household member) of main respondent or spouse （） ___________(HA010_3) [preload list]
(5) nonresident other relatives
(6) friends
(7) other

F1

HA011. What is the present market value of your house? Or, what is the present market value of a similar

housing unit within its neighborhood?
Total price _______________(HA011_1) 10000Yuan [soft check <0.1, >500] or unit price_____________(HA011_2)1000Yuan/m^2 / [soft check: <0.1, >25]
※IWER: skip to HA013 if R answered HA011. If not, ask unfolding brackets. HA011HA013

[PROCEDURE: If HA011_1>=100,ask HA011_check][HA011_1100HA011_check]
HA011_check. Are you sure the present market value of your house is [preload HA011_1] 10000Yuan? [HA011_1]

(1) Yes
(2) No go back HA011 HA011

HA012. [IWER: If R is unwilling to answer or does not remember, ask unfolding bracket questions here.] 20,000/50,000/100,000/200,000/500,000 yuan

[If HA007 =2 or 3, then skip to HA027 HA007 =2 or 3HA027]

HA013. Do you or other household members take out a bank loan to finance the purchase, construction, or decoration of your house now? （）

(3) Yes
(4) No → Skip to HA016 HA016

F1

HA014. What is the outstanding amount of the loans? _____________ Yuan [soft check: >500,000 yuan]

HA015. What is the monthly mortgage payment? ___Yuan [soft check: >10000]

HA016. How was this housing unit obtained?

(1) Purchased from market
(2) Purchased from working unit of household member(s) ______(HA016_1) [preloaded list]
(3) Purchased from child(non-household member) of main respondent or spouse, which child? （）
___(HA016_2) [preloaded list]
(4) Purchased from Parents(non- household member), of who（main respondent or spouse）? （）
（）______(HA016_3)
(5) Purchased from Other relatives
(6) Self-built → Skip toHA025 HA025
(7) Inherited, bequeathed, or given （） → Skip to HA020 HA020
(8) Received home as compensation for demolition of old home Skip to HA021
(9) Other → Skip to HA020 HA020

HA017. When did you purchase it? __________ 1900..2011 Year Skip to HA018 HA018
[IWER: Mark the year using four digits. : 4]

HA018. How much of your own money did you spend on the new house?
______________10000Yuan [soft check >500]
If HA016 = 8, skip to HA022 HA016 = 8 HA022

HA019. Was it purchased at market price, subsidized by working unit, or as purchased as economical housing?

(1) Market price → Skip to HA025 HA025
(2) Subsidized by working unit
(3) Economic housing
(4) Other

F1

HA020. What would you have to pay if you had paid a market-set price for the same housing?
Total price _______________(HA020_1)10000Yuan [soft check <0.1, >500] or unit price_____________(HA020_2)1000Yuan/m^2 / [soft check: <0.1, >25]
Skip to HA025 if HA016 not equal to (8) HA016 （8）HA025

[PROCEDURE: If HA020_1>=100,ask HA020_check][HA020_1100HA020_check]
HA020_check. Are you sure you had paid a market-set price [preload HA020_1] 10000Yuan for the same housing? [HA020_1]
(1)Yes
(2)No go back HA020 HA020

HA021. When did you receive the housing? ________ 1900..2011year [IWER: Mark the year using four digits. : 4]

HA022. How much was the market value of the old house at that time?
Total price _______________ 10000Yuan [soft check: <0.1, >500]

[PROCEDURE: If HA022_1>=100,ask HA022_check][HA022_1100HA022_check]
HA022_check. Are you sure you had paid a market-set price [preload HA022 _1] 10000Yuan for the old house? [HA022_1]
(1)Yes
(2)No go back HA022 HA022

HA023. How much was the market value of the new house at that time?
Total price _______________(HA023_1) 10000Yuan [soft check: <0.1, >500] or unit price_____________(HA023_2)1000Yuan/m^2 / [soft check: <0.1, >25]

[PROCEDURE: If HA023_1>=100,ask HA023_check][HA023_1100HA023_check]
HA023_check. Are you sure the market value of the new house is [preload HA023 _1] 10000Yuan at that time? [HA023_1]
(1)Yes
(2)No go back HA023 HA023

HA024. Can you sell the house freely?
（1） Yes
（2） No, restricted by work unit

HA025. How much did you spend on decorating or renovating your house (exclude expenditures on furniture)? （） __________________ Yuan [soft check: >500,000 yuan]

HA026. When was it decorated/renovated (if more than once, choose time of greatest expendithure)? （）
__________ 1900..2011year
[IWER: Mark the year using four digits. : 4]

HA027. Excluding the house in which you live, do you or members of your household own any other residential properties? （）
(1) Yes
(2) No → Skip to HA054 HA054

HA028. How many other housing units do you or members of your household currently own? ____________ 0..10

PART 2 Other Residences

For every other housing unit owned by house members, ask the following questions,

HA029. Now we want to know other housing unit owned by house members, nnGA036 load
Where is this house located? _______Province__________District, Zip Code_____________

_______(HA029_1)__________(HA029_2) ____________ (HA029_3)

HA030. Who owns this residence?
(1) Owned completely by your household member(s).
(2) Owned partly by your household member(s).

HA031. Which household member(s) own the house? (preloaded names of household members)/_______ []

HA032. What share of the house is owned by (preloaded names of household members)? (GA042_2) _______0.00..100.00% [hard check: range 0-100]
[If HA030 =1, skip to the procedure before HA034. HA030 =1HA034]

HA033. What non-household members own all or part of your current residence? (circle all that apply) （）
(1) working unit of household member, which household member?_____ (HA033_1)______[preloaded list][]
(2) government indemnificatory housing
(3) child(non- household member) of main respondent or spouse （）, which child?___(HA033_2) [preloaded list]
(4) parent(non- household member) of main respondent or spouse （） ___________(HA033_3) [preload list]
(5) nonresident other relatives
(6) friends
(7) other

[PROCEDURE: If HA031 does not include respondent or spouse, SKIP TO Next house. HA031]

HA034. What is the present market value of your house? Or, what is the present market value of a similar housing unit within its neighborhood?
Total price _______________(HA034_1)10000Yuan [soft check <0.1, >500] or unit price_____________(HA034_2)1000Yuan/m^2 / [<0.1, >30]
[CAPI Skip to HA036 if R answered HA034. If not, ask unfolding brackets. HA034HA036]

[PROCEDURE: If HA034_1>=100,ask HA034_check][HA034_1100HA034_check]
HA034_check. Are you sure your present market value of your house is [preload HA034_1] 10000Yuan? [HA034_1]
（1）Yes
（2）No go back HA034 HA034

HA035. [IWER: If R is unwilling to answer or does not remember, ask unfolding bracket questions here.] 20,000/50,000/100,000/200,000/500,000 yuan

HA036. Does your household members take out a bank loan to finance the purchase, construct, or decorate your house? （）
(1) Yes
(2) No → Skip to HA039 HA039
F1

HA037. What is the outstanding amount of the loans? _______________ 10,000 Yuan [soft check >500]

HA038. What is the monthly mortgage payment? _____ Yuan soft check >20,000]

HA039. How was this housing unit obtained?
(1) Purchased from market
(2) Purchased from working unit of respondent or spouse ______(HA039_1) [preloaded list]
(3) Purchased from child of main respondent or spouse, which one? （） _____(HA039_2)preload[]

(4) Purchased from parents of main respondent or spouse, of who(main respondent or spouse)? ()
() ______ (HA039_3)
(5) Purchased from Other relatives
(6) Self-built → Skip to HA041 HA041
(7) Inherited, bequeathed, or given () → Skip to HA045 HA045
(8) Received home as compensation for demolition of old home, Skip to HA046 Skip to HA046
(9) Other → Skip to HA045 HA045

HA040. Was it purchased at market price, subsidized by working unit, or as purchased as economical housing?
(1) Market price → Skip to HA045 HA045
(2) Subsidized by working unit
(3) Economic housing
(4) Other
F1

HA041. When did you purchase/build it? / _________ 1900..2011Year
[IWER: Mark the year using four digits. : 4]
Skip to HA043 HA043

HA042. Can you sell the house freely if you want?
(1) Yes
(2) No, restricted by work unit

HA043. How much of your own money did you spend on the house (including loan-financed)?
//_____________ 10000Yuan [soft check<0.1, >500]
[IWER: Skip to HA045 if R answered HA043 . If not, ask unfolding brackets. HA043 HA045]

[PROCEDURE: If HA043_1>=100,ask HA043_check][HA043_1100HA043_check]
HA043_check. Are you sure you had spend [preload HA043_1] 10000Yuan on the house? //[HA043_1]
(1)Yes
(2)No go back HA043 HA043

HA044. [IWER: If R is unwilling to answer or does not remember, ask unfolding bracket questions here.] 10,000/20,000/50,000/100,000/200,000 yuan

If HA039 = 8, Skip to HA046. HA039 = 8 HA046

CAPIIf HA039 = 6, Skip to HA049
HA045. What would you have to pay if you had paid a market-set price for the same housing?
Total price _______________(HA045_1) 10000Yuan [soft check >500] or unit price_____________(HA045_2) 1000Yuan/m^2 / [soft check >30]
Skip to HA049 HA049 if HA039 does not equal to (8)
[PROCEDURE: If HA045_1>=100,ask HA045_check][HA045_1100HA045_check]
HA045_check. Are you sure you had paid a market-set price [preload HA045_1] 10000Yuan for the same housing? [HA045_1]
(1)Yes
(2)No go back HA045 HA045

HA046. When did you receive the new housing? _____ 1900..2011Year [IWER: Mark the year using four digits. : 4]

HA047. How much was the market value of the old house at that time?
Total price _____________ 10000Yuan [soft check <0.1, >500]
[PROCEDURE: If HA047_1>=100,ask HA047_check][HA047_1100HA047_check]
HA047_check. Are you sure the market-set price of your old house is [preload HA047_1] 10000Yuan?

[HA047_1]
(1)Yes
(2)No go back HA047 HA047

HA048. How much was the market value of the new house at that time?
Total price _______________(HA048_1) 10000Yuan [soft check<0.1, >500] or unit price_____________(HA048_2) 1000Yuan/m^2 /
[PROCEDURE: If HA048_1>=100,ask HA048_check][HA048_1100HA048_check]

HA048_check. Are you sure the market value of the new house at that time is [preload HA048_1] 10000Yuan? [HA048_1]
(1)Yes
(2)No go back HA048 HA048

HA049. How much did you spend on decorating or renovating your house (exclude expenditures on furniture)? （） ________________ Yuan [soft check <1000, >500,000]

HA050. When was it decorated/renovated (if more than once, choose time of greatest expendithure)? （） _________ 1900..2011Year
[IWER: Mark the year using four digits. : 4]

HA051. What is the construction area of the house? ___________ m^2 [soft check<10 or >500]

[Skip to next house]

HA052. What is the monthly rental income for all room or houses owned by main respondent or spouse, that you are currently leasing? （）
(1) _______________ (HA052_1)Yuan/month / [soft check >20,000]
(2) Not applicable

HA053. What is the monthly rental income for all room or houses owned by other household members that are currently being leased?
(1) _______________ (HA053_1)Yuan/month /
(2) Not applicable

PART 3 Land
The following questions pertain to your land. .

HA054. Does your household have any collective distributing or rent cultivated land, forest land, pasture and/or pond? (Choose all that apply) （）
（1） Cultivated land (HA054_1)
（2） Forest land (HA054_2)
（3） Pasture (HA054_3)
（4） Pond (HA054_4)
（5） None (HA054_5) Skip to HA064 HA064

[PROCEDURE: According to all options choosed in HA054 , ask HA055 -HA063 in loop] [HA054 HA055 - HA063]

HA055. How many mu of [preload answer from HA054] do you have? [HA054] ________ Mu [soft check>50]

[PROCEDURE: If HA054 =1,ask HA056] [HA054 =1HA056]

HA056. How many mu of them are irrigable? _______Mu [hard check cannot be >HA055]

HA057. What is the rent per mu per year you would get if you rent out all your [preload answer from HA054]? [HA054][soft check <10, >4000]

________ Yuan per mu per year

HA058. Did you rent out any of your [preload answer from HA054] in the past year? [HA054]
(1) Yes
(2) No >>Skip to HA061 HA061

HA059. How much [preload answer from HA054 did you rent out the past year? [HA054]_____ Mu [soft check >50] [hard check, cannot be >HA055]

HA060. How much rental income did you earn in the past year? [HA054]____ Yuan

HA061. Did you rent in any [preload answer from HA054] from others (including the collective) in the past year? （） [HA054]
(1) Yes
(2) No >>Skip to HA064 HA064

HA062. How much did you rent in the past year? [HA054]_____ Mu [soft check >100]

HA063. How much rent did you pay in the past year? [HA054]____ Yuan [soft check >20000]

HA064. How much rental income did you earn for any other household assets other than housing or land? (trees, use of fixed capital, durables, or livestock)? （）
(1) _______(HA064_1)Yuan
(2) Not applicable
F11

PART 4 Equipments, Consumption durables, and Valuables.

[Show Card 31]
HA065. Do members of your household own the following assets? (Choose all that apply) （）

For all categories, add [soft check <100 or >30,000] unless other check is written	[For each asset owned by the household] what is the assets current value? (Yuan) （）
Automobile [soft check <3000, >500,000]	(HA065_1[1])
Electric Bicycle	(HA065_1[2])
Motorcycle	(HA065_1[3])
Refrigerator	(HA065_1[4])
Washing machine	(HA065_1[5])
TV	(HA065_1[6])
Computer	(HA065_1[7])
Stereo system	(HA065_1[8])
Video camera	(HA065_1[9])
Camera	(HA065_1[10])
Air conditioner	(HA065_1[11])
Mobile phone	(HA065_1[12])
Furniture	(HA065_1[13])
Music instrument	(HA065_1[14])
Valuable decorations, ornaments, vases	(HA065_1[15])
Treasures and precious metal (such as gold) you own? （）	(HA065_1[16])

Antiques, valuable paintings and calligraphic work, and other artistic work you own?	(HA065_1[17])
None	

F1（1）
（2）
（3）（）

HA066. Do members of your household own the following fixed capital assets? How much are the assets worth? (check all that apply) [ask same questions as in GA063] （）[GA063]
(1) Tractor, current value_______Yuan______(HA066_1) [soft check <1000, >30,000]
(2) Thresher, current value_______Yuan() ______(HA066_2)[soft check <100 or >10,000]
(3) Tractor tools, current value_______Yuan______(HA066_3) [soft check <100 or >10,000]
(4) Water pump, current value_______Yuan ()_____(HA066_4)[soft check <100 or >10,000]
(5) Processing equipment, current value_______Yuan_____(HA066_5)[soft check <100 or >10,000]
(6) None (HA066_6)
F1（1）
（2）120002

HA067. What is the current value of other fixed capital assets used in household production or self-employed activities? ______ Yuan
F1120002
[IWER: Be sure to ask about fixed capital assets used in all self-employment activities, do not count assets already reported above.]

HA068. Does your household have any other durable or fixed assets worth 500 yuan or more? 500
(1) Yes How much are the assets worth? ___(HA068_1)Yuan [hard check>500] [soft check>50,000]
(2) No
F1（1）120002
（2）1

HA069. Have you lent to other families or individuals and not been repaid by them?
(1) Yes
(2) No Skip to HA071 HA071

HA070. What is the total amount of the loans? ___________________ Yuan [soft check>500,000]

HA071. How much interest income from what you lent to others in past year? __________ Yuan [soft check >50000]

HA072. What is the total amount of loans that you are still owing to other families, individuals, or your work unit? (not including mortgage loans) （）__________________ Yuan [soft check >500,000]
[IWER: Skip to HA074 if R answered HA072 If not, ask unfolding brackets HA073. HA072HA074HA073]

HA073. [IWER: If R is unwilling to answer or does not remember, ask unfolding bracket questions here.]5,000/10,000 /50,000 /100,000 /200,000 /500,000 yuan

[Intro: Next we will ask your household members, other than the main respondent and spouse, some financial questions.
[IWER: The names of other household members not including respondent and spouse are preloaded from the cover screen information. For each member, answer the following questions:] []
HA074. What is the value of all financial assets of [preload household member name] (includes cash,

savings, stocks, funds)? [preload household member name] （） ____Yuan [soft check >1,000,000]

[PROCEDURE: If HA074_1>=1000000,ask HA074_check][HA074_1100HA074_check]
HA074_check. Are you sure the value of all financial assets of [preload household member name] (includes cash, savings, stocks, funds) is [preload HA074_1] Yuan? [][HA074_1] （）

(1)Yes
(2)No go back HA074 HA074

HA075. What is the value of all outstanding (unpaid) loans from banks or financial institutions (not including mortgages) of [name]? [name] （） __________ Yuan
[soft check >500,000]

HA076. How often did the respondent receive assistance in answering section Household assets?
[IWER: If it is answered by a proxy, please record the respondents reaction.]

(1) Never
(2) A few times
(3) Most or all of the time

HB INDIVIDUAL ASSETS

[IWER: Please conduct sections HB and HC when the main respondent and his/her spouse are at home. Dont allow a proxy to complete the entire sections. HBHC]

PART 1 Housing Reform

The following questions pertain to the housing reform.

HB001. Have you ever purchase d a housing unit from your work unit?
(1) Yes
(2) No → Skip to HB018 HB018

HB002. Did you live in the housing unit before you purchased it from your work unit?
(1) Yes
(2) No

HB003. Did you obtain the deed of the housing unit that you purchased from your work unit?
(1) Yes
(2) No

F1

HB004. Is this your current residence?
(1) Yes
(2) No

HB005 . Do you still own the house that you purchased from your work unit?
(1) Yes Skip to HC001 HC001
(2) No

HB006. When did you purchase the housing unit from your work unit?
_____ 1949..2011Year
[IWER: Mark the year using four digits. :]

HB007 . At what price did you purchase the housing unit?
Total price____________(HB007_1)10000Yuan [soft check: <0.1, >500] or unit price________(HB007_2)1000Yuan/m^2 /[soft check: <0.1, >25]
[IWER: Skip to HB009 if R answered HB007. If not, ask HB008 HB007HB009 HB008]
[PROCEDURE: If HB007_1>=100,ask HB007_check][HB007_1100HB007_check]
HB007_check. Are you sure you had paid [preload HB007_1] 10000Yuan for the housing unit? [HB007_1]
(1)Yes
(2)No go back HB007 HB007

HB008. Which of the following is close to the amount you spent on purchasing the house form your work unit then?
(1) Less than 5000
(2) 5,000 to 10,000
(3) 10,000 to 20,000
(4) 20,000 to 50,000
(5) 50,000 to 100,000
(6) More than 100,000

HB009. Was it purchased at market price, subsidized by working unit, or as purchased as economical housing?
(1) Market price → Skip toHB011 HB011

(2) Subsidized by working unit
(3) Economic housing
(4) Other

F1

HB010. What would you have had to pay if you had paid a market-set price for the same housing?
Total price __________(HB010_1)10000Yuan [soft check <0.1, >500] or unit price_________(HB010_2)1000Yuan/m^2 /[soft check <0.1, >25]
[PROCEDURE: If HB010_1>=100,ask HB010_check][HB010_1100HB010_check]
HB010_check. Are you sure the market-set price of the same housing was [preload HB010_1] 10000Yuan at that time? [HB010_1]
(1)Yes
(2)No go back HB010 HB010

HB011. What was the construction area of the housing unit? _________ m^2 [soft check <10, >500]

HB012. When did you sell or give away the housing unit? _______ 1949..2011Year
[IWER: Mark the year using four digits. :]

HB013. At what price did you sell it （0 if gave away）? (0)
Total price _________ (HB013_1)10000Yuan [soft check <0.1, >500] or unit price __________(HB013_2)1000Yuan /m^2 / [soft check <0.1, >25]

[PROCEDURE: If HB013_1>=100,ask HB013_check][HB013_1100HB013_check]
HB013_check. Are you sure you sold it at a price of [preload HB013_1] 10000Yuan? [HB013_1]
(1)Yes
(2)No go back HB013 HB013

[IWER: Skip to HB015 if R answered HB013. If not, ask HB014. HB013 HB015HB014]
HB014. Which of the following is close to the total price you sold?
(1) Less than 20,000
(2) 20,000 to 50,000
(3) 50,000 to 100,000
(4) 100,000 to 200,000
(5) 200,000 to 500,000
(6) More than 500,000

HB015. Who did you sell or give away the housing unit to?
(1) Back to work unit
(2) Commercial market
(3) Child, which one? _____(HB015_1)preload[]
(4) Parents, of who（respondent or spouse）? （） ______ (HB015_2)
(5) Other relative, specify______(HB015_3)
(6) Other

HB016. Did you sell at the market price or at a price below the market price?
(1) Market price Skip to HC001 HC001
(2) Below market price

HB017. What was the market value of the housing unit when you sold it?
Total price __________(HB017_1)10000Yuan [soft check <0.1, >500] or unit price_________(HB017_2)1000Yuan/m^2 / [soft check <0.1, >25]
[PROCEDURE: If HB017_1>=100,ask HB017_check][HB017_1100HB017_check]
HB017_check. Are you sure the market value of the housing unit was [preload HB017_1] 10000Yuan when you sold it? [HB017_1]
(1)Yes

(2)No go back HB017 HB017

Skip to HC001 HC001

HB018. Why didnt you purchase housing unit from your work unit?
(1) I have no work unit
(2) Not eligible to purchase
(3) Eligible but no housing unit was made available
(4) Eligible but could not afford
(5) Eligible but decided not to purchase
(6) Other

PART 2 Financial Assets
The following questions pertain to your financial asset.

[IWER reminder: make sure others are not present, IWER read following instructions: The following questions pertain to your financial asset. The answers to these questions will be kept strictly confidential and will be used for research purposes only.]

HC001. How much cash is held by you and your spouse at home? （） __________ Yuan [soft check >50,000]
[IWER: Skip to HC003 if R answered HC001. If not, ask unfolding brackets. HC001 HC003]

HC002. [IWER: If R is unwilling to answer or does not remember, ask unfolding bracket questions here.]500 /1,000 /2,000 /5,000 /10,000 yuan

If married (= 1 or 2), ask: (BE001 = 1 or 2)
HC003. Of which, how much do you own (if jointly owned with spouse then count 50%)? （50%）
________(HC003_1)yuan [hard check <GM019] [soft check > 50,000] or _______(HC003_2) % [range 0-100]

[CAPI: prompt for HC004 -HC019 IWER: for deposit, bondsstocks, and funds, only include assets legally in his/her name.HC004 -HC013]

HC004. Are you currently holding any deposits in financial institutions in your name? （）
(1) Yes
(2) No Skip to HC007 HC007

HC005. What is the total amount of deposits you are currently holding in financial institutions（eg:bank）? ()_______________ Yuan [soft check >500,000]

[IWER: Skip to HC007 if R answered HC005 . If not, ask unfolding brackets. HC005HC007]

HC006. [IWER: If R is unwilling to answer or does not remember, ask unfolding bracket questions here.] 2,000/5,000/10,000/50,000 /100,000/200,000/500,000 yuan

HC007. Do you have any government bonds (e.g.Treasury bills) in your name? （）（）
(1) Yes
(2) No -〉Skip to HC010 HC010

HC008. What is the total face value of government bonds that you are currently holding? ()? __________________ Yuan [soft check >50,000]

[IWER: Skip to HC010 if R answered HC008. If not, ask unfolding brackets HC009. HC008HC010HC009]

HC009. [IWER: If R is unwilling to answer or does not remember, ask unfolding bracket questions here.] 10,000 /50,000 /<u>100,000</u> /200,000 /500,000 yuan

HC010. Have you held any stocks in the past year in your name, excluding the equity or stock of your work unit? （）

(1) Yes
(2) No -〉Skip to HC015 HC015

HC011. During the past year, overall did you earn money from stock dividends and increases in the value of your stocks or did you lose money?

(1) Earned
(2) Lost -〉Skip to HC012_2 HC012_2
(3) Unchanged -〉Skip to HC013 HC013

HC012. How much did you earn? __Yuan [soft check >200,000]
HC012_2. How much did you lose? __Yuan [soft check >200,000

HC013. What is the present market value of all the stocks you are currently holding? _________________ Yuan [soft check >200,000]

[IWER: Skip to HC015 if R answered HC013. If not, ask unfolding brackets. HC013HC015]

HC014. [IWER: If R is unwilling to answer or does not remember, ask unfolding bracket questions here.] 10,000 /50,000 /<u>100,000</u> /200,000 /500,000 yuan

HC015. Have you held any funds in your name in the past year? （）

(1) Yes
(2) No -〉Go to PROGRAM before HC020 HC020

HC016. During the past year, overall did you earn money from holding funds or did you lose money?

(1) Earned
(2) Lost
(3) Unchanged -〉Skip to HC018 HC018

HC017. How much did you earn or lose? /____ Yuan [soft check >200,000]

HC018. What is the present market value of all the mutual funds you are currently holding? ___ _______________ Yuan [soft check >200,000]
[IWER: Skip to HC020 if R answered HC018. If not, ask unfolding brackets. HC018HC020]

HC019. [IWER: If R is unwilling to answer or does not remember, ask unfolding bracket questions here.]10,000 /50,000 /<u>100,000</u> /200,000 /500,000 yuan]

[PROGRAM: If HC004 =1 or HC007 =1 or HC010 =1 or HC015 =1, go to HC020; if HC004 =2 and HC007 =2 and HC010 =2 and HC015 =2, then go to HC021. HC004 =1 or HC007 =1 or HC010 =1 or HC015 =1HC020HC004HC007 HC010 HC015 2HC021]
HC020. What percentage of the deposits, bonds, stocks, and funds held in your name is fully controlled by you and not your spouse? (%) __ _____0..100％ [hard check >=0, <=100]

HC021. Do you have any other deposits, bonds, stocks, or funds that belong to you but which are held in a persons name other than you or your spouse?

(1) Yes
(2) No Skip to HC023 HC023

HC022. What is the value of such assets? ____yuan [soft check >200,000]

HC023. Other than income you have already told me about, did you receive any other income from other investments in past year?

(1) Yes
(2) No → Skip to HC027 HC027

HC024. How much did you receive altogether from other investments in the past year? ________ Yuan [soft check >200,000]

[IWER: Skip to HC027 .if R answered HC024. If not, ask unfolding brackets HC025. HC024HC027HC025]

HC025. [IWER: If R is unwilling to answer or does not remember, ask unfolding bracket questions here.]1,000 /5,000 /10,000 /20,000 /50,000 yuan]

HC026. What percentage of the other investments is owned jointly with your spouse? ________0..100% [hard check <=0, >=100]

HC027. Do you have public housing funding?

(1) Yes
(2) No Skip to HC030 HC030

F1

HC028. What is the total amount of money in your public housing fund? ________________ Yuan [soft check >100,000]

F1

[IWER: Skip to HC030 .if R answered HC028. If not, ask unfolding brackets HC029. HC028HC030 HC029]

HC029. [IWER: If R is unwilling to answer or does not remember, ask unfolding bracket questions here.]5,000/10,000 /50,000 /100,000 /200,000 /500,000 yuan

HC030. Do you have Jizikuan that your work unit or other work units have collected from you and are still holding (Jizikuan is fund individuals provided to the work unit for the purpose of investment, building apartments, etc.)?

(1) Yes
(2) No Skip to HC033 HC033

F1,

HC031. What is the amount of your jizikuan? _________________ Yuan [soft check >200,000]

F1,

[IWER: Skip to HC033 .if R answered HC031. If not, ask unfolding brackets HC032. HC031HC033HC032]

HC032. [IWER: If R is unwilling to answer or does not remember, ask unfolding bracket questions here.]5,000/10,000 /50,000 /100,000 /200,000 /500,000 yuan

HC033. Do you have any unpaid salary that your work unit still owes you?

(1) Yes
(2) No Skip to HC036 HC036

HC034. What is the amount of your unpaid salary? ______________ Yuan

[soft check >100,000]

[IWER: Skip to HC036.if R answered HC034. If not, ask unfolding brackets HC035. HC034HC036HC035]

HC035. [IWER: If R is unwilling to answer or does not remember, ask unfolding bracket questions here.]5,000/10,000 /50,000 /100,000 /200,000 yuan

HC036. Have you participated in any rotating savings and credit association during the past year? ?

(1) Yes

(2) No → HD001 → HD001

F1,

HC037. What is the total amount of funds that you are still obligated to pay to the rotating savings and credit association ____ yuan

PART 3 DEBTS
The following questions pertain to your debt.

HD001. What is the total amount of loan that you havent repaid yet (not including loans for house)? () ________________ Yuan [soft check>500,000]

[IWER: Skip to HD003 if R answered HD001. If not, ask unfolding brackets HD002. HD001 HD003 HD002]

HD002. [IWER: If R is unwilling to answer or does not remember, ask unfolding bracket questions here.]5,000/10,000 /50,000 /100,000 /200,000 /500,000 yuan

HD003. What is the amount of your credit card balance? ________________ Yuan [soft check >50,000]

[IWER: Skip toHD005 if R answered HD003, If not, ask unfolding brackets HD004. HD003 HD005 HD004]

HD004 . [IWER: If R is unwilling to answer or does not remember, ask unfolding bracket questions here.]5,000/10,000 /50,000 /100,000 /200,000 /500,000 yuan

HD005. Have you ever inherited anything

(1) Yes
(2) No → Skip to HD012 HD012

HD006. How much in total have you inherited ________ Yuan （） [soft check >200,000]
[CAPI: Skip to HD008 if R answered HD006 If not, ask unfolding brackets HD007. HD006 HD008 HD007]

HD007. [IWER: If R is unwilling to answer or does not remember, ask unfolding bracket questions here.]5,000/10,000 /50,000 /100,000 /200,000 /500,000 yuan

HD008. From whom you inherited(Choose all that apply) （）
(1)Parents
(2)Parents-in-law
(3)Children
(4)Relatives
(5)Others

HD009. When did the largest inheritance occur_______ 1900..2011 Year
[IWER: Mark the year using four digits. :]

HD010. What was the value of that inheritance? _______________ Yuan
[CAPI: Skip to HD012 if R answered HD010. If not, ask unfolding brackets HD011..HD010HD012HD011] [soft check >200,000]

HD011. [IWER: If R is unwilling to answer or does not remember, ask unfolding bracket questions here.]5,000/10,000 /50,000 /100,000 /200,000 /500,000 yuan

HD012. How often did the respondent receive assistance in answering section G ASSETS?
[IWER: If it is answered by a proxy, please record the respondents reaction.]

(1) Never
(2) A few times
(3) Most or all of the time

I. HOUSING CHARACTERISTICS

[PROCEDURE: For main respondent only I001 --- I026] [I001 --- I026]
I001. What is the construction area of your residence? ______m^2
[soft check: <10 or >500]

I002. [What is the total housing land area (*zhaijidi,* including both the house construction area and t he yard area) ? （） __m^2 [soft check <10, >1000]

I003. Is your residence used for business as well?
(1)
(2)

I004. What type of structure is this building? Is it concrete and steel, mixed structure, bricks and wo od, wood, bamboo, grass or other?
（1） Concrete and steel
（2） Bricks and wood
（3） Mixed structure
（4） Wood, bamboo, grass
（5） Woolen felt
（6） Sheet iron
（7） Cave dwelling
（8） Tent
（9） Adobe
（10） Other

I005. When was this house built?
____year
If R is unclear about year, choose:
（1）0-5 years 0-5
（2）5-10 years 5-10
（3）10-20 years 10-20
（4）20-30 years 20-30
（5）30-40 years 30-40
（6）more than 40 years 40

I006. Is the building one story or multi-level building, how many stories?
（1） One-story building skip to I007
（2） Multi-story building skip to I008

I007. Is the story independent or compound?
(1) Independent story
(2) Compound

I008. Which story is this building on?
____

[PROCEDURE: If I008 >1, ask I009.] [I008 >1I009 .]
I009. Does it has elevator
（1）Yes
（2）No

I010. Are there any handicapped facilities (e.g., non-stair ramp)? （）

(1) Yes
(2) No

[PROCEDURE: If I010 =2, ask I011.] [I010 =2I011]

I011. How many steps had to be climbed to get to the main entrance of the household's flat?
[IWER: Do not count steps if an elevator is available.]

(1) 1- 5 1-5
(2) 6 to 15 6-15
(3) 16 to 25 16-25
(4) More than 25 25

I012. How many bedrooms, living rooms, bathrooms, and kitchens are there in your residence? _________(I012_1)bedrooms________(I012_2)living rooms_________(I012_3)toilets （ ）________(I012_4)kitchens________(I012_5)balcony [soft check: >20]

[PROCEDURE: Ask I013 if no toilets in answer to I012.] [I012 I013]

I013. How far is the nearest toilet to your house? ___ meters [soft check: >500]

I014. What is the type of toilet?
（1） Toilet without a seat
（2） Toilet with a seat

I015. Is the toilet flushable?
（1）
（2）

I016. Does your residence have electricity?
(1) Yes
(2) No

I017. Does your residence have running water?
(1) Yes
(2) No

I018. Is there in-house shower or bath facility? What type?
(1) Hot water provided
(2) Water heater installed by the household
(3) No

I019. Does your residence have coal gas or natural gas supply?
(1) Yes
(2) No

I020. Does your residence have heating? （）
(1) Yes → Skip to I022 I022
(2) No

I021. What is the main heating energy source?
(1) Solar
(2) Coal
(3) Natural gas
(4) Liquefied Petroleum Gas
(5) Electric
(6) Crop residue/Wood buring
(7) Other

I022. What is the main source of cooking fuel?
(1) Coal
(2) Natural gas
(3) Marsh gas
(4) Liquefied Petroleum Gas
(5) Electric
(6) crop residue/Wood burning
(7) other

I023. Does your residence have a telephone connection?
(1) Yes
(2) No

I024. Does your residence have broad-band internet connection?
(1) Yes
(2) No

I025. [Interviewer records it] [] How clear and tity is in this household
(1) Excellent
(2) Very clear
(3) Clear
(4) Fair
(5) Poor

I026. [Interviewer records it] []How is the temperature in this household
(1) Very hot
(2) Hot
(3) Bearable
(4) Cold
(5)Very cold

HOUSEHOLD CONTACTS

INTRO: We will inform you blood tests report. And in the future, we will visit this household again.Your continue participation is very important to our studay. In order to find you in the future survey, we have to get your and your friendfamily members contact information. All the contact information will be kept strictly confidential.Thanks for your understanding and help.

AIK1. Could you please tell me your contact information?

Name [preload main respondent name] :[]

Home └─┴─┴─┴─┘ (AIK1_2_1)- └─┴─┴─┴─┴─┴─┴─┴─┘ (AIK1_2_2)

INTRO: I make a call to your home telephone to check whether it is right.

[IWER:Call the number to check.If the number is right, then continue,if wrong, then go back and correct.]

AIK2. IWER:Record whether the home telephone number is right.

(1)right

(2)wrong

10000000000.0..19999999999.0**Cell phone number :** └─┴─┴─┴─┴─┴─┴─┴─┴─┴─┴─┘ (AIK1_3)

Intro: I make a call to your cell phone to check whether it is right.

[IWER:Call the number to check.If the number is right, then continue,if wrong, then go back and correct.]

AIK3. WER:Record whether the cell phone number is right.

(1)right

(2)wrong

AIK4. Email address :____

AIK5. Could you give us your mailing address, so we can send you blood test result later

Mailing Address : ____ Zip Code ____________________ (AIK1_7)

INTRO: We will call on you two years later, could you give us the name and contact information of a friend/family member who can tell us where you are? 2013/

AIK6. Name :____________(AIK6_1)What is his relationship to you? ____(AIK6_1_0)

Telephone number : Home └─┴─┴─┴─┘ (AIK6_2_1)- └─┴─┴─┴─┴─┴─┴─┴─┘ (AIK6_2_2)

10000000000.0..19999999999.0Cellphone number : └─┴─┴─┴─┴─┴─┴─┴─┴─┴─┴─┘ (AIK6_3)

Email address :____(AIK6_3_1)

Mailing Address : ____(AIK6_4)_ Zip Code ____________________ (AIK6_7)

AIK7. Could you give us the name and contact information of a second friend/family member who can tell us where you are? /

Name :____________________________(AIK7_1) What is his relationship with you? ____(AIK7_0)

Telephone number : Home └─┴─┴─┴─┘ (AIK7_2_1)- └─┴─┴─┴─┴─┴─┴─┴─┘ (AIK7_2_2)

10000000000.0..19999999999.0 Cellphone number : └─┴─┴─┴─┴─┴─┴─┴─┴─┴─┴─┘ (AIK7_3)

Email address :____(AIK7_3_1)

II. HOUSEHOLD CONTACTS

Mailing Address : ______________________________ (AIK7_4) Zip Code ____________ (AIK7_7)

**AIK8a.** Did you move within last two years?<br>1.Yes 2.No skip AIK8b AIK8b	1. Move ↓ 2. NOT MOVE ➔next section 8. DONT KNOW
**AIK8b.** Did you move here because of demolition, stay here temporary or just move back because of demolition?<br>1. move here because of demolition<br>2. stay here temporary<br>3. move back because of demolition<br>4. other___	↓ Skip to the next section
**AIK8.** Do you intend to move within the next two years?	1. Not same ↓
**AIK9.** If you move, where will you move? /	
Country :Province :	1______________ 2same ➔next section<br>8 DONT KNOW
City, district/county /	1______________ 2same ➔next section<br>8 DONT KNOW
Street/Township /	1______________ 2same / ➔next section<br>8 DONT KNOW
Community/Village /:	1______________ 2same / ➔next section<br>8 DONT KNOW
**AIK10.** Could you please tell us a contact information for us to contact you in the future if you move, the phone number of your hometown is the best.<br>Name____________ (AIK10_1)<br>what is his/her relationship with you______________________ (AIK10_2)<br>phone number____________ (AIK10_3)	1______________ 2same / ➔next section

J. INTERVIEWER OBSERVATION

[IWER. This section is about your observations during the interview and should be filled out after each completed individual interview.] [.]

J001. Were any third persons, except proxy respondents, present during (parts of) the interview with ^FL Respondent Name? (circle all that apply) ( )

(1) Nobody Skip to J003 J003
(2) Spouse or partner
(3) Parent or parents
(4) Child or children
(5) Other relatives
(6) Other persons present

J002. Did these persons intervene during the interview? /

(1) Yes, often
(2) Yes, occasionally
(3) No

J003. How would you describe the willingness of ^FLRespondentName to answer?

(1) Very good Skip to J005 J005
(2) Good Skip to J005 J005
(3) Fair Skip to J005 J005
(4) Bad Skip to J005 J005
(5) Good in the beginning, got worse during the interview
(6) Bad in the beginning, got better during the interview Skip OV005 OV005

J004. Why did the respondent's willingness to answer get worse during the interview? (Choose all that apply)
( )

(1) The respondent was losing interest
(2) The respondent was losing concentration or was getting tired
(3) Other, please specify _______

J005. Did ^FLRespondentName ask for clarification on any questions?

(1) Never
(2) Almost never
(3) Now and then
(4) Often
(5) Very often
(6) Always

J006. Overall, did you feel that ^FLRespondentName understood the questions?

(1) Never
(2) Almost never
(3) Now and then
(4) Often
(5) Very often
(6) Always

J007. Did the respondent need any help reading the showcards during the interview?

(1) Yes, due to sight problems
(2) Yes, due to literacy problems
(3) No

APPENDIX

Appendix 1: List of Chinese Zodiac signs :

**1**	**Rat charm**
**2**	**Ox patient**
**3**	**Tiger sensitive**
**4**	**Rabbit articulate**
**5**	**Dragon healthy**
**6**	**Snake deep**
**7**	**Horse popular**
**8**	**Goat elegant**
**9**	**Monkey clever**
**10**	**Rooster deep thinkers**
**11**	**Dog loyalty**
**12**	**Pig chivalrous**

Appendix 2: List of provinces

1	Anhui
2	Beijing
3	Chongqing
4	Fujian
5	Gansu
6	Guangdong
7	Guangxi
8	Guizhou
9	Hainan
10	Henan
11	Hebei
12	Heilongjiang
13	Hunan
14	Hubei
15	Inner mongolia
16	Jiangsu
17	Jiangxi
18	Jilin
19	Liaoning
20	Ningxia
21	Qinghai
22	Shandong
23	Shanghai
24	Shannxi
25	shanxi
26	Sichuan
27	Tianjin
28	Tibet
29	Xinjiang
30	Yunnan
31	Zhejiang
32	Hongkong
33	Macao

Appendix 3: List of Occupation

1	**Agriculture, Forestry, herd, fishing**
2	**Mining**
3	**Manufacture**
4	**Energy sector(including electricity, gas and water)**
5	**Construction**
6	**Transportation, warehousing industry and postal industry**
7	**Information transmission , computer service and software industry**
8	**Wholesale and Retail**
9	**Lodging and catering sector**
10	**Finance**
11	**Real estate business**
12	**Leasing industry and Business Service Industry**
13	**Science research, technical service and geology prospecting industry**
14	**Water conservancy , environments and public facilities management**
15	**Personal Services and other service industry**
16	**Education**
17	**Public healthcare, social security and public welfare**
18	**Culture , sports and entertainment**
19	**Common administration and society organize**
20	**International organization**
