# Supplementary material for: Association of total sleep duration variability with risk of new stroke in the middle-aged and elderly Chinese population
Source: BMC Neurol. 2024 Jun 25;24:217. doi: 10.1186/s12883-024-03727-8 (PMC11197293; doi:10.1186/s12883-024-03727-8)
Supplement: Supplementary file 2 — Supplementary Material 2 [file 12883_2024_3727_MOESM2_ESM.pdf]

China Health and Retirement Longitudinal Study

Followup Questionnaire 2013

2013

Jan 2015

Revised: Nov 2015

China Center for Economic Research
Institute of Social Science Survey
Peking University

Contents

B DEMOGRAPHIC BACKGROUNDS 1

C FAMILY 21

CM LISTED HOUSEHOLD MEMBERS 21

C1 PARENT, CHILDREARING AND SIBLING INFORMATION 22

CA PARENT INFORMATION 22

CC SIBLINGS 28

CG Living Arrangements preferences 28

CB CHILDREARING INFORMATION 30

A OTHER HOUSEHOLD MEMBER 48

C2 TIME TRANSFER AND TRANSFERS 51

CD TIME TRANSFER 51

CE TRANSFERS 53

CF TIME SPENT PROVIDING CARE 61

D HEALTH STATUS AND FUNCTIONING 65

DA HEALTH STATUS 65

DA HEALTH STATUS 66

PART I: GENERAL HEALTH STATUS AND DISEASE HISTORY 67

PART II: LIFESTYLE AND HEALTH BEHAVIORS 85

DB FUNCTIONAL LIMITATIONS AND HELPERS 92

DC COGNITION & DEPPRESSION 104

E HEALTH CARE AND INSURANCE 109

PART I MEDICAL INSURANCE 109

PART II HEALTH CARE COSTS AND UTILIZATION 115

F WORK, RETIREMENT AND PENSION 135

FA JOB STATUS 137

FB WORK HISTORY 145

FC CURRENT PRIMARY JOB/OCCUPATION 148

FARM EMPLOYED 148
HOUSEHOLD AGRICULTURAL WORK 149
FD EMPLOYED 152
FE QUESTIONS ABOUT LABOR SUPPLY 163
FF QUESTIONS ABOUT WAGES 163
FG Fringe Benefits 165
FH NON-FARM SELF-EMPLOYED AND UNPAID FAMILY BUSINESS 169
FI SOCIAL INSURANCE QUESTION FOR THE SELF-EMPLOYED 175
FJ SIDE JOB (EMPLOYED OR SELF-EMPLOYED) ( ) 177
FK UNEMPLOYMENT AND JOB SEARCH ACTIVITIES . 178
FL LAST JOB 179
FM RETIREMENT 184
FN PENSION INSURANCE 194
Part 1 PENSION PROGRAM OF THE GOVERNMENT AND INSTITUTIONS OR BASIC PENSION OF THE FIRMS 196
Part 2 SUPPLEMENT PENSION INSURANCE OF THE FIRM 201
Part 3 COMMERCIAL PENSION INSURANCE 204
Part 4 LIFE INSURANCE 207
Part 5 RURAL PENSION, RESIDENTS PENSION, URBAN RESIDENTS PENSION ( ) 208
Part 6 New Rural Social Pension Insurance 210
Part 7 ENDOWMENT INSURANCE FOR THE LAND-LOSING FARMERS (/) 212
Part 8 OLD AGE PENSION ALLOWANCE 214
Part 9 OTHER PENSION 214
G&H INCOME, EXPENDITURES AND ASSETS 219
G1 RELATIVE INCOME 219
G2 HOUSEHOLD INCOME AND EXPENDITURES 220

PART 1 Household Wage Income and Individual-based transfers 220
PART 2 HOUSEHOLD AGRICULTURAL INCOME AND EXPENDITURE 224
PART 3 Self-employed Activities 227
PART 4 HOUSEHOLD PUBLIC TRANSFER INCOME 228
PART 5 HOUSEHOLD LIVING EXPENDITURE 229
HA HOUSEHOLD ASSETS 232
PART 1 Current Residence 232
PART 2 Other Residences 241
PART 3 Land 246
PART 4 Equipments, Consumption durables, and Valuables. 247
HB INDIVIDUAL ASSETS 250
PART 1 Financial Assets 250
PART 2 DEBTS 254

I HOUSING CHARACTERISTICS 257

J INTERVIEWER OBSERVATION 261

B DEMOGRAPHIC BACKGROUNDS

**NOTE**: ONLY THOSE WHO WERE BORN BEFORE JULY 1, 1968 AND THEIR SPOUSES WILL BE INTERVIEWED. 1968 7 1

Type of Interview R	
XRType = REIW	This is a reinterview R
XRType = NEWIW	This is a new interview R

BA000_W2_1 We record your name is [name], is it right? []
[**PROCEDURE**: Preload the name of R.]
1. Yes → Skip to BA000_W2_3 BA000_W2_3
2. No

BA000_W2_2 Whats your name? __________

BA000_W2_3 Interviewer record Rs gender.
[**PROCEDURE**: Preload Rs gender.]
1. Male
2. Female

**BA001 BRANCHPOINT**:<br>IF XRType = REIWAND CHINESE ZODIAC SIGN IN THE LAST WAVE IS MISSING, GO TO BA001.<br>IF XRType = REIWAND CHINESE ZODIAC SIGN IN THE LAST WAVE IS NOT MISSING, GO TO BA002 BRANCHPOINT.<br>IF XRType = NEWIW, GO TO BA001.

[Show Card 2]

BA001 What is your Chinese Zodiac sign? ________
[IWER: Choose from the list of Chinese Zodiac signssee Appendix 1]

**BA002 BRANCHPOINT**:<br>IF XRType = REIW AND DATE OF BIRTH IN THE LAST WAVE IS MISSING, GO TO BA002.<br>IF XRType = REIW AND DATE OF BIRTH IN THE LAST WAVE IS NOT MISSING, PRELOAD THE BIRTH TIME AND GO TO BA001_W2_1.<br>IF XRType = NEWIW, GO TO BA002.

BA001_W2_1 We record your birth time is [Birth Time], is it right? []

1. Yes → Skip to BA005 BA005
2. No

BA002 When were you born?

____ 1900…2013 (**BA002_1**) year __0…12 (**BA002_2**) month __0..31 (**BA002_3**) day

[IWERThe year must be a number in the range [1900 – 2013]. Mark the year using four digits. Take down the month as its actual number. For example, write January as 1 not 01, December as 12. If do not remember month and day, fill 0. [1900 — 2013] 4 1 101,12 12 ‘0]

[CAPI: Check date ofbirth by Zodiac.]

PROCEDURE :
If the person does not know the date ofbirth, BA002 = DK or BA002 = RF, skip BA003.
BA002BA003.

BA003 Is your answer to BA002 based on the solar or the lunar calendar?

( ) ( )

1. Solar calendar ( )
2. Lunar calendar ( )

PROCEDURE :
If the person does not know the date ofbirth, BA002 = DK or BA002 = RF, ask BA004.
BA002 BA004

BA004 What is your age? ________1…120 years old

[IWER: You can refer to the year for some major events or born in which year during the republic of China. (Begin in 1912). (1912)]

F1 1912 +1911=

BA005 Do you go home almost every day, or attend school or work away from home but return to this home almost every week, or go home once two weeks or more than two weeks? , /,

1. go home almost everyday
2. go home almost every weekend /
3. go home once two weeks or more than two weeks

BA006_W2_1 Are you communist?

1. Yes
2. No

BA007_W2_1 Whats your nationality?

1. Han
2. Hui
3. Zhuang
4. Weiwuer
5. Yi
6. Zang
7. Miao
8. Mongol
9. Dai
10. Other _____ (**BA007_W2_1_1**)

[INTRO: Next are some questions about your birth place, some changes in your housing location, your Hukou and education.]

**PROCEDURE** :<br>If XRType = REIW, preload the permanent address. in the last wave<br>If XRType = NEWIW, preload the map address. this wave.

BB000_W2_1 According to our recorded [permanent address], whats your current permanent address? []

1. The same address
2. ______(**BB000_W2_1_1**) province_city_county/city/district _ _ // __ (**BB000_W2_1_2**) village/neighborhood /
3. Abroad

[IWER: Confirm the permanent address with respondents, and fill in the changed. If do not know the name, please fill in other]

[IWER: county/city includes county-level administrative units/county-level city/district. ///.]

BB000_W2_2 Whats type of the permanent address?

1. Family housing
2. Nursing home
3. Hospital

4. Other ____ ____ （**BB000_W2_2_1**）

BB001 Where were you born?

1. The same as permanent address
2. Another village/neighborhood in permanent address‘s county/city/district /// (**BB001_1**) village/neighborhood /
3. Other ____(**BB001_2**) province_city_county/city/district _ _ // _(**BB001_3**) village/neighborhood /
4. Abroad

[IWER: county/city includes county-level administrative units/county-level city/district. ///.]

BB002 BRANCHPOINT:

IF XRType = REIWAND TYPE OF BIRTH PLACE IN THE LAST WAVE IS MISSING, GO TO BB002.

IF XRType = REIWAND TYPE OF BIRTH PLACE IN THE LAST WAVE IS NOT MISSING, GO TO BB003 BRANCHPOINT.

IF XRType = NEWIW, GO TO BB002.

BB002 What is the type of your birth place? Is it rural village or urban community?

1. Rural Village
2. Urban Community

PROCEDURE :

If BB001 = 1, then skip to BB006 BRANCHPOINT.

If BB001 = 2, then skip to BB005.

BB003 When did you first live in permanent addresss county/city/district? // ____
1900..2013 Year

[IWER: Mark the year using four digits. 4]

BB004 When you first moved to permanent address, did you live in the same village/community as you currently do? /

1. Yes → skip to BB006 BRANCHPOINT BB006
2. No

BB005 In what year did you first live in permanent addresss village/community? / ____
1900..2013 Year

[IWER: Mark the year using four digits. 4]

BB006 BRANCHPOINT:

IF XRType = REIW AND BB006 IN THE LAST WAVE IS NOT MISSING, THEN SKIP TO PROCEDURE BEFORE BB007.

BB006 Where did you mainly live before you were 16 years old? Is it in village or city/town? 16

[IWER: City or Village when the person was living there.]

1. Village
2. City/Town

PROCEDURE :

If BB001 = 1 or BB001 = 2 go to BB009. BB001 = 1 or BB001 = 2BB009.

BB007 Ever since you first came to permanent city/county/district, have you ever lived outside your permanent city/county/district for more than 6 months? ////

1. Yes
2. No → Go to BC001 BC001

BB008 Ever since you first came to your permanent county/city/district, how long have you lived outside your permanent county/city/districtin total?

// // ____ (**BB008_1**) years _____

(**BB008_2**) months

→ Skip to BB011 BB011

BB009 Have you ever lived outside your permanent county/city/district for more than 6 months? //

1. Yes
2. No → Skip to BC001 BC001

BB010 How long have you lived outside your permanent county/city/district in total? //

____ (**BB010_1**) years ____ (**BB010_2**) months

[hard check: years <= 2013-BB003 <= 2013-BB003]

BB011 When did you most recently live in your permanent county/city/district (after being away for 6 months or longer)? // ( ) ____ 1900..2013 year

[IWER: Mark the year using four digits. 4]

[hard check: year >= BB003 >= BB003]

[Show Card 3]

BB012 Before moving to the current permanent county/city/district, where else did you live for at least six months? (Not less than six months, otherwise ask about previous locations where the respondent lived for at least six months) // ( )

1. Birth place skip to BC001
2. Other ____ (**BB012_1**) province_city_county/city/district _ _ //
   ____(**BB012_2**) village/neighborhood /
3. Abroad

[IWER: county/city includes county-level administrative units/county-level city/district. ///]

BB013_W2_1 Why did you live in other county/city? /

1. School
2. Marriage
3. Look after relatives
4. Live in childrens house
5. Travel
6. Work
7. In the hospital
8. Other ________ （**BB013_W2_1_1**）

BC001 What is your current HuKou status?

1. Agricultural Hukou
2. Non-agricultural Hukou
3. Unified Residence Hukou
   [IWER: for the place where agricultural hukou is abandoned.]
4. Do not have Hukou → Skip to procedure before BD001_W2_1 BD001_W2_1
   F1

**PROCEDURE** :<br>IF XRType = NEWIW, skip to procedure before BC002.

**PROCEDURE** : Preload the answer ofBC001in last wave, then compare the two answers. BC001.<br>If they are different then ask BC001_W2_1. BC001_W2_1 If they are the same then skip to BC005_W2_1 BRANCHPOINT. BC005_W2_1

BC001_W2_1 We record your hukou status is [hukou status] in last wave, your hukou status has changed during this two years, right? [ ]

1. Yes

2. No → SKIP TO BC005_W2_1 BRANCHPOINT BC005_W2_1

PROCEDURE :
If BC001 = 1 then ask BC001_W2_2. BC001 = 1BC001_W2_2
If BC001 = 2 then ask BC001_W2_3. BC001 = 2BC001_W2_3 If BC001 = 3 then ask BC001_W2_4. BC001 = 3BC001_W2_4

BC001_W2_2 What is the reason that you changed to the agricultural Hukou?

1. live in rural areas aftergraduate from college
2. marriage
3. employment
4. migration of the whole village
5. No change → SKIP TO BC005_W2_1 BRANCHPOINT
6. others, pls specify _____ , ____ （**BC001_W2_2_1**）

PROCEDURE :
SKIP TO BC001_W2_4

BC001_W2_3 What is the reason that you changed to the non-agricultural Hukou?

1. Go to a college
2. marriage
3. employment
4. Land is acquired by the government
5. migration of the whole village
6. No change → SKIP TO BC005_W2_1 BRANCHPOINT
7. others, pls specify _____ ____ (**BC001_W2_3_1**)

BC001_W2_4 When did you change the status of Hukou? ____ year

PROCEDURE :
IF XRType = REIW, SKIP TO BC005_W2_1 BRANCHPOINT.

PROCEDURE :
If BC001 = 3, ask BC002. BC001 = 3BC002BC003 to BC003.

BC002 What is your Hukou status before you have the unified residence Hukou?

1. Agricultural Hukou
2. Non-agricultural Hukou

3. Do not have Hukou
F1

BC003 When did you have the unified residence Hukou? ____ (**BC003_1**)
1900…2013 year ____ (**BC003_2**) 0…12 month

PROCEDURE :
Go to BC005_W2_1 BRANCHPOINT. BC005_W2_1

BC004 When did you get your current Hukou? ____ 1900…2013 (**BC004_1**)
year ____ 0…12 (**BC004_2**) month
[hard check: enforce year >= BA002_1 >= BA002_1 ()]

[IWER: You must fill year [1900 - 2013]. Mark the year using four digits. Take down the month as its actual number. For example, write January as 1 not 01, December as 12. If do not remember month, fill 0. [1900-2013] 4 1 10112 12 ‘0]

BC005_W2_1 BRANCHPOINT:
IF XRType = REIWAND HUKOU LOCATION IS NOT MISSING IN THE LAST WAVE, GO TO BC005_W2_1.
IF XRType = REIW AND HUKOU LOCATION IS MISSING IN THE LAST WAVE, GO TO BC005.
IF XRType = NEWIW, GO TO BC005.

BC005_W2_1 Since last interview, have your Hukou location changed?
1. Yes
2. No → skip to procedure before BD001_W2_1 BD001_W2_1

[CAPI: Preload the permanent address.]

BC005 Where is your current HuKou location?
[IWER: If birthplace is Rs permanent address, pls choose the first choice.]
1. Permanent address
2. Birthplace
3. Another village/neighborhood in permanent address county/city/district /// ___ (**BC005_1**) village/neighborhood /
4. Other ____ (**BC005_2**) province_city_county/city/district _ _ // _ (**BC005_3**) village/neighborhood /

[IWER: county/city includes county-level administrative units/county-level city/district. : ///]

**BC005_W2_2 BRANCHPOINT**:<br>IF XRType = REIW AND BC005_W2_1 = 1, GO TO BC005_W2_2.<br>IF XRType = NEWIW, GO TO THE PROCEDURE BEFORE BC006.

BC005_W2_2 Why the location of current Hukou is different from your lastwave?

1. marriage
2. go to school
3. employment
4. retirement/revolutionary retirement /
5. migration of the whole village
6. others, pls specify_____ ____ （**BC005_W2_2_1**）

BC005_W2_3 When did you change the location of Hukou? _1900…2013 year

**PROCEDURE** :<br>If (BC005 = 2 and BB001 = 3/4) or BC005 = 4, please ask BC006. (BC005 = 2 BB001 = 3/4) BC005 = 4BC006

BC006 How long have you been away from your current Hukou county/city/distric? // _ （**BC006_1**） 1900…2013 Years ____ （**BC006_2**） ___0…12 Months

[hard check: impose that years <= 2013-BA002_1 (birth year) <= 2013-BA002_1 ()]

**PROCEDURE** :<br>IF XRType = REIW, skip to the procedure before BD001_W2_1. BD001_W2_1

BC007 Is your first Hukou the same as your current Hukou?（Not including the change of unified Residence Hukou.） （ ）

1. Yes
2. No

BC008 Were there any other Hukou between your first Hukou and your current Hukou? (Including changes of hukou type and place. Hukou type change only means changes between Agricultural Hukou and non-agricultural hukou.) （ ）

1. Yes
2. No

PROCEDURE :
If BC007 = 1 and BC008 = 2, go to the procedure before BD001_W2_1. BC007 = 1 BC008 = 2 BD001_W2_1

BC009 Has your Hukou type or place ever changed since your first Hukou? (Hukou type change only means changes between Agricultural Hukou and non-agricultural hukou.) ( )

1. Both Hukou type and place have changed 2. Only Hukou type has changed
3. Only Hukou place has changed

PROCEDURE :
If BC009 = 1/2, ask BC010. BC009 = 1/2, BC010
If BC009 = 3. skip BC010. BC009 = 3. BC010

BC010 What was your first HuKou status?

1. Agricultural Hukou
2. Non-agricultural Hukou

PROCEDURE :
If BC008 = 1 and (BC009 = 1/3), ask BC011, else skip BC011. BC008 = 1 BC009 = 1/3BC011, BC011

[CAPI: Preload the permanent address]

BC011 Where was your last HuKou location?
[IWER: If last HuKou location is Rs birthplace, and both of them are in the county/city of Rs permanent address, pls choose the first choice. R]

1. Birthplace
2. Permanent address
3. Another village/neighborhood in permanent address county/city/district /// ___ (**BC011_1**) village/neighborhood /
4. Other ____ (**BC011_2**) province_city_county/city/district _ _ // _ (**BC011_3**) village/neighborhood /

[IWER: county/city includes county-level administrative units/county-level city/district. : ///]

PROCEDURE :
If BC011 = 2/3/4, ask BC012 , else go to the procedure before BC013. BC011 = 2/3/4BC012BC013

BC012 Why the location of last Hukou different from your birth Hukou location?

1. marriage
2. go to school
3. employment
4. retirement/ revolutionary retirement /
5. escape of famine
6. sent down to the countryside to do manual labor
7. migration of the whole village
8. With the parents migration
9. others, pls specify_____ ____ （**BC012_1**）

**PROCEDURE** :<br>If BC009 = 2, go to procedure before BC015. BC009 = 2, BC015

[CAPI: Preload the permanent address.]

BC013 What was the location of your first HuKou?

[IWER: If the location of Rs first hukou is the same as Rs birthplace/current hukou/permanent address, the choose the first answer.
1]

1. Birthplace
2. Same as current Hukou
3. permanent address
4. Another village/neighborhood in permanent address county/city/district /// ___ (**BC013_1**) village/neighborhood /
5. Other ____ (**BC013_2**) province_city_county/city/district _ _ // _ (**BC013_3**) village/neighborhood /

[IWER: county/city includes county-level administrative units/county-level city/district. : ///]

**PROCEDURE** :<br>If BC001 = 1 and BC009 = 1/2, ask BC015 to BC016, then skip to procedure before BD001_W2_1. BC001 = 1 BC009 = 1/2BC015BC016 BD001_W2_1

BC015 What is the reason that you had the non-agricultural Hukou?

1. sent down during 1967-1977 and did not come back to city 1967-1977
2. was a student
3. marriage

4. employment
5. migration of the whole village
6. escape of famine
7. others, pls specify_____ ____ (**BC015_1**)

BC016 When did you have the non-agricultural Hukou?
From _____(**BC016_1**) 1900...2013 year to _____(**BC016_2**) 1900...2013 year

PROCEDURE :
If BC001 = 2 and BC009 = 1/2, ask BC017 to BC018. BC001 = 2 BC009 = 1/2 BC017BC018

BC017 What is the reason that you had the agricultural Hukou? 1. sent down during 1967-1977 and then come back to city 1967-1977 2. born in rural areas
3. marriage
4. employment
5. escape of famine
6. others, pls specify ____ , ____(**BC017_1**)

BC018 When did you have the agricultural Hukou?
From_______(**BC018_1**) 1900...2013 year to ______(**BC018_2**) 1900...2013 year

PROCEDURE :
If XRType = NEWIW, ask BD001.
If XRType = REIW, and BD001 in last wave is missing, ask BD001.
If XRType = REIW, and BD001 in last wave is not missing, ask BD001_W2_1.

[CAPI: Preload the Rs highest level of education in last wave]

BD001_W2_1 We record your highest level of education is [education level] in last wave, is it right? []
1. Yes
2. No → skip to BD001_W2_3 BD001_W2_3

BD001_W2_2 Is the highest level of education contains adult education in last wave?
1. Yes
2. No → skip to BD001_W2_4

BD001_W2_3 Whats the highest level of education in last wave? (not including adult education) ( )

1. No formal education (illiterate) ( )
2. Did not finish primary school but capable of reading and/or writing
3. Sishu/home school
4. Elementary school
5. Middle school
6. High school
7. Vocational school ( )
8. Two-/Three-Year College/Associate degree
9. Four-Year College/Bachelors degree
10. Masters degree
11. Doctoral degree/Ph.D.

BD001_W2_4 Have your highest level of education changed from last wave? If so, what is the highest level of education you have attained now? (not including adult education) ( )

1. No formal education (illiterate) ( ) → skip to the procedure before BD007_W2_1 BD007_W2_1
2. Did not finish primary school but capable of reading and/or writing
3. Sishu/home school
4. Elementary school
5. Middle school
6. High school
7. Vocational school ( )
8. Two-/Three-Year College/Associate degree
9. Four-Year College/Bachelors degree
10. Masters degree
11. Doctoral degree/Ph.D.
12. No change

**PROCEDURE** :<br>Skip to the procedure before BD002

BD001 What is the highest level of education you have attained? (not including adult education) ( )

1. No formal education (illiterate) ( ) → Skip to the procedure before BD007_W2_1 BD007_W2_1
2. Did not finish primary school but capable of reading and/or writing
3. Sishu/home school

4. Elementary school
5. Middle school
6. High school
7. Vocational school （）
8. Two-/Three-Year College/Associate degree
9. Four-Year College/Bachelors degree
10. Masters degree
11. Doctoral degree/ Ph.D.

PROCEDURE :
If BD001 = 3 or BD001_W2_4 = 3, ask BD002. BD001 = 3 BD001_W2_4 = 3 BD002

BD002 What is the highest grade did you finish in primary school? __ 1...6

PROCEDURE :
If BD001 = 4 or BD001_W2_4 = 4, ask BD002_W2_1. BD001 = 4 BD001_W2_4 = 4BD002_W2_1

BD002_W2_1 How many years did you spend in sishu/home school? __

PROCEDURE :
If BD001 >= 5 or BD001_W2_4 >= 5, ask BD003. BD001 >= 5 BD001_W2_4 >= 5, BD003.

BD003 How many additional years of schooling did you complete after [THE ANSWER CHOSEN IN BD001]? [BD001] ____ years

PROCEDURE :
If BD001 > 7 or BD001_W2_4 > 7, ask BD004. BD001 > 7 BD001_W2_4 > 7, BD004
If XRType = REIW and BD004 is not missing in last IW, skip to the procedure before BD005. XRType = REIW BD004 , BD005
If XRType = REIW and BD004 is missing in last IW, ask BD004. XRType = REIW BD004 , BD004
If XRType = NEWIW, ask BD004. XRType = NEWIW, BD004

BD004 When did you go to college? /? ____1900...2013 year

PROCEDURE :
IF XRType = REIW and BD005 is not missing in last IW, skip to BD006. XRType = REIW BD005 , BD006
IF XRType = REIW and BD005 is missing in last IW, ask BD005. XRType = REIW BD005 , BD005.
IF XRType = NEWIW, ask BD005. XRType = NEWIW, BD005.

BD005 At what age did you begin formal schooling? / _____1…120 years old [soft check, < 6, > 11]

BD006 At what age did you finish schooling? ____1…120 years old

[IWERIt asks age when R finishes schooling, not age when R finished elementary school. /]

BD007_W2_1 BRANCHPOINT:

IF XRType = REIW AND BD007 IN THE LAST WAVE IS NOT MISSING, GO TO BD007_W2_1.

IF XRType = REIW AND BD007 IN THE LAST WAVE IS MISSING, GO TO BD007.

IF XRType = NEWIW, GO TO BD007

BD007_W2_1 Have you attended school for adult education since last interview? ( ) ? ( )

1. None → Go to BE001 BE001
2. TV University → Go to BD008_W2_1
3. Night School → Go to BD008_W2_1
4. Zikao (examinations for self-taught students) → Go to BD008_W2_1
5. Hanshou/Correspondence course/Distance learning → Go to BD008_W2_1
6. Literacy course → Go to BD008_W2_1
7. Accelerated education course → Go to BD008_W2_1
8. Other (explain:) ____(**BD007_W2_1**) → Go to BD008_W2_1

BD007 Have you ever attended school for adult education? ( ) ? ( )

1. None → Go to BE001 BE001
2. TV University
3. Night School
4. Zikao (examinations for self-taught students)
5. Hanshou/Correspondence course/Distance learning
6. Literacy course
7. Accelerated education course
8. Other (explain:) ____(**BD007_1**)

BD008 How many years did you spend in adult education? __year [soft check > 9] → GO TO BD009

BD008_W2_1 How many years did you spend in adult education since last interview? ______year [soft check > 2] → GO TO BD009_W2_1

BD009 Did you get a diploma or degree from the adult education program you attended?

1. Yes → GO TO BD010
2. No → GOTO BE001 BE001

BD009_W2_1 Did you get a diploma or degree from the adult education program you attended since last interview?

1. Yes → GO TO BD010
2. No → GOTO BE001 BE001

BD010 When did you receive the diploma?

______1900...2013 year

BD011 BD011] What is the highest level of schooling you obtained from the adult education program?

1. Vocational school
2. Two/Three Year College / Associate degree
3. Four Year College / Bachelors degree
4. Others

[Show Card 1]

BE001 RMaritalStatus: What is your marital status?

[IWER: common-law marriage is considered as married,]

1. Married with spouse present
2. Married but not living with spouse temporarily for reasons such as work
3. Separated ( )
4. Divorced
5. Widowed
6. Never married
7. Cohabitated

**PROCEDURE** :   If BE001 = 6, then skip to BF008. BE001 = 6 BF008.

[INTRO: Many people have more than one marriage through whole life. Please bear me a few more questions on this.]

**PROCEDURE** :   IF XRType = REIW, ask BE003_W2_1   IF XRType = NEWIW, ask BE003

BE003 How many times have you been married? ____ times [soft check > 2]

PROCEDURE :
IF XRType = NEWIW, skip to the procedure before BE004.

BE003_W2_1 How many times have you been married since last interview? ____ times
[soft check > 2]

PROCEDURE :
If BE003 = 1, continue with BE004 - BE005, then go to the procedure before BF001.
BE004 - BE005BF001
If BE003_W2_1 = 0, go to the procedure before BF001. BE003_W2_1 = 0, BF001
If BE003 > 1, continue with BE009. BE003 > 1, BE009.
If BE003_W2_1 > 0, continue with BE009. BE003_W2_1 > 0, BE009.

BE004 When did you get married?
______1900..2013 (**BE004_1**) year ____ 0..12 (**BE004_2**) month [soft check: year > BA002_1 + 16, >BA002_1 + 16]

[IWER: You must fill year[1900 - 2013]. Mark the year using four digits. Take down the month as its actual number. For example, write January as 1 not 01, December as 12. If do not remember month, fill 0. : [1900 - 2013] 4 1 10112 12 ‘0]

BE005 When you were married, what was the total value of cash and goods (including housing) that your parents gave to you and your spouse? ( ) _______Yuan

PROCEDURE :
Skip to procedure before BF001 BF001

BE009 When was your most recent marriage? 1900...2013 (**BE009_1**) year ____ 0...12 (**BE009_2**) month
[hard check: enforce year>=BE007, >=BE007]

[IWER: You must fill year[1900 - 2013]. Mark the year using four digits. Take down the month as its actual number. For example, write January as 1 not 01, December as 12. If do not remember month, fill 0. : [1900-2013] 4 1 10112 12 ‘0]

[IWER: Id like to ask you a few questions about your current (or most recent) spouse/partner.]

[INTRO: An important part of this study is understanding how people make decisions during different stages of life, we also need to a general age range for your spouse.]

**BF001 BRANCHPOINT**:
IF THIS R HAS SPOUSE (BE001 = 1/2/7), THEN SKIP TO BF008.
IF R DID NOT CHANGE HIS/HER SPOUSE (BE003_W2_1=0) AND SPOUSE/PARTNERS CHINESE ZODIAC SIGN IS NOT MISSING AT LAST WAVE, SKIP TO BF002 BRANCHPOINT.

[Show Card 2]

BF001 What is your spouse/partners Chinese Zodiac sign? _____
[IWER: Choose from the list of Chinese Zodiac signssee Appendix 1.]

**BF002 BRANCHPOINT**:
IF THIS R DID NOT CHANGE HIS/HER SPOUSE (BE003_W2_1=0) AND SPOUSE/PARTNERS BIRTH DATE IS NOT MISSING AT LAST WAVE, SKIP TO BF004.

BF002 When was your spouse/partner born?
______1900...2013 (**BF002_1**) year ____0...12 (**BF002_2**) month [preload spouses birthdate]

[IWER: Mark the year using four digits. Take down the month as its actual number. For example, write January as 1 not 01, December as 12. If do not remember month, fill 0. : 4 1 1 0112 12 ‘0]

BF003 Is your spouse/partner‘s date of birth based on the solar calendar or the lunar calendar? ( ) ( )

1. Solar calendar ( )
2. Lunar calendar ( )

BF004 What is the highest level of education your spouse/partner has attained? (not including adult education) ( ) 1. No formal education (illiterate) ( )

2. Did not finish primary school but capable of reading and/or writing
3. Sishu/home school
4. Elementary school
5. Middle school
6. High school
7. Vocational school ( )
8. Two-/Three-Year College/Associate degree
9. Four-Year College/Bachelors degree
10. Masters degree

11. Doctoral degree/Ph.D.

**BF005 BRANCHPOINT**:
IF (BE001 = 3), THEN ASK BF005.

BF005 When did you separate?

____ 1900...2013 (**BF005_1**) year __0...12 (**BF005_2**) month [hard check: enforce year > year married BE004 IF BE003 = 1) BE009 IF BE003 > 1)

[IWERMark the year using four digits. Take down the month as its actual number. For example, write January as 1 not 01, December as 12. If do not remember month and day, fill 0. 4 1 101,12 12 ‘0]

**BF006 BRANCHPOINT**:
IF (BE001 = 4), THEN ASK BF006.

BF006 When did you divorce?

____ 1900...2013 (**BF006_1**) year __0...12 (**BF006_2**) month [hard check: enforce year > year married BE004 IF BE003 = 1) BE009 IF BE003 > 1)

[IWERMark the year using four digits. Take down the month as its actual number. For example, write January as 1 not 01, December as 12. If do not remember month and day, fill 0. 4 1 101,12 12 ‘0]

BF006_W2_1 What is the reason of your divorce?

1. Emotional feud
2. Live in different places
3. In order to facilitate the property purchase transactions 4. Other ____ (**BF006_W2_1_1**)

BF006_W2_2 Divided assets, including real estate, how much is yours(yuan) and how much is your ex-wife/husband(yuan)? () ____ (**BF006_W2_2_1**) / ____ (**BF006_W2_2_2**)

BF006_W2_3 Do you have any infancy children? Owed to whom?

1. Myself
2. My last spouse /
3. None → Skip to BF007

BF006_W2_4 How much money you or your ex-wife/husband pay for upbringing the children(yuan)? / _______Yuan

**BF007 BRANCHPOINT**:
IF (BE001 = 5), THEN ASK BF007.

BF007 When did your spouse pass away?

____ 1900...2013 (**BF007_1**) year __0...12 (**BF007_2**) month [hard check: enforce year > year married BE004 IF BE003 = 1) BE009 IF BE003 > 1)

[IWERMark the year using four digits. Take down the month as its actual number. For example, write January as 1 not 01, December as 12. If do not remember month and day, fill 0. 4 1 101,12 12 ‘0]

BF008 How often did the respondent receive assistance in answering section Demographics?

[IWER: If it is answered by a proxy, please record the respondents reaction.]

1. Never → Skip to next module
2. A few times → Skip to next module
3. Most or all of the time → Skip to next module
4. The section was completed by a proxy respondent → Skip to next module

BF009 What is your relationship to R?

[IWER: What is the proxys relationship to R? If unknown, please ask the proxy.]

1. Spouse
2. Mother
3. Father
4. Mother-in-law /
5. Father-in-law /
6. Sibling
7. Brother-in-law, sister-in-law /
8. Child
9. Spouse of child
10. Grandchild
11. Other relative
12. Helper or other non-relative

BF010 What is the main reason for proxy (the respondent is absent)?

1. The respondent has serious physical handicaps 2. The respondent has serious mental handicaps
3. The respondent has rejected this interview
4. Other ____(**BF010_1**)

C FAMILY

Type of Interview R	
XRType = REIW	This is a reinterview R
XRType = NEWIW	This is a new interview R

CM LISTED HOUSEHOLD MEMBERS

PROCEDURE :

If this is a reinterview household, please preload the household member list in last wave, if the number of household member is 0, skip to CM002_W2_1. 0CM002_W2_1. If this is a new household, please preload the household member list recorded in coverscreen, if the number of household members is 0, skip to CM002_W2_1. 0CM002_W2_1.

[INTRO: Types of household members are as followed: People who live together (in respondents permanent address) and share daily expenses; parents who rotate amongst their children but stay mostly in this household; those who do not live here, but live in dormitories, workplaces, or other places that are not normal residential areas; live-in nannies, drivers or any other service people. ( )]

CM001_W2_1 I will go through a list of people living in this household that our system recorded. Please let me know whether this list is correct.

[IWER: MARK THE MEMBER THAT LIVE IN THE HH.]

1. Prev wave hhmember[1]
2. Prev wave hhmember[2]
3. …

CM002_W2_1 Is there any other household member that we havent recorded? ( ) ( )

[IWER: Record each additional household members name.]

PROCEDURE :

For each household member that is not selected in CM001_W2_1, please ask CM003_W2_1. CM001_W2_1CM003_W2_1.

CM003_W2_1 I know this might be difficult to talk about, but what happened to [hhmember name] that didnt choose from CM001_W2_1? [CM001_W2_1]

1. Household member moved out
2. Household member passed away
3. Household member never lived here
4. Other ____________ (**CM003_W2_1_1**)

**NOTE**: DEFINITIONS FOR MOTHER, FATHER-IN-LAW AND MOTHER-IN-LAW ARE SIMILAR TO FATHERS. FOR THE LOOPS ON FATHER, MOTHER, FATHER-IN-LAW AND MOTHER-IN- LAW, THEY ARE NAMED AS VARNAME[1],VARNAME[2],VARNAME[3] AND VARNAME[4] RESPECTIVELY.

[IWER: Section CA and Section CC and Section CG are asked for each Rs and Section CB is only asked for Family R. If Family R is not in or couldnt answer the questions, spouse can be a proxy, but other people are not allowed. CA/CC/CG CBCDCE CF]

C1 PARENT, CHILDREARING AND SIBLING INFORMATION

[In the following three parts: CA parent information, CB childrering information and CC sibling information, Id like to ask you some questions about your family.]

CA PARENT INFORMATION

[Id like to ask you some questions about your parents.]

**CA000_W2_1 BRANCHPOINT**:<br>IF WE KNOW THAT Rs FATHER WAS NOT LIVING AT Rs LAST IW, ASK CA000_W2_1, OTHERWISE GO TO CA001.

CA000_W2_1 We recorded that your father was dead at last wave, is it right? ()

1. Yes → Skip to CA002 CA002
2. No

CA001 Is your father still living?

1. Yes
2. No

[IWER: If the respondent reports more than one father, ask about the father who raised the respondent.]

**CA001_W2_ 1 BRANCHPOINT**:   IF WE KNOW RS FATHERS NAME AT LAST IW, ASK CA001_W2_1, OTHERWISE, GO TO CA002.

CA001_W2_ 1 Is your fathers name is [Father Name]? [] 1. Yes → Skip to CA003
BRANCHPOINT CA003
2. No

[CAPI: Preload the household members name list.]

CA002 What is his name? _________

[IWER: Choose from the list, if there is none, choose other and fill in, be careful the near sound words.

IWER: Mark father if respondent doesnt want to give the name.]

**CA003 BRANCHPOINT**:   If CA000_W2_1 = 1, THEN SKIP TO CA014 AND THEN SKIP ALL THE OTHER QUESTIONS ABOUT FATHER.   IF WE KNOW RS RELATIONSHIP WITH FATHER AT RS LAST IW, GOTO THE PROCEDURE BEFORE CA004.

CA003 Is your father your… …?

1. Biological father
2. Adoptive father
3. Stepfather
4. A different biological relative who raised you
5. Another individual who raised you

**PROCEDURE** :   If CA001 = 1, then ASK CA004 AND CA005; otherwise, skip them.

[CAPI: Preload the Rs permanent address]

CA004 Where was your father born?

1. Your permanent address
2. Another village/neighborhood in your permanent addresss county/city/district /// ________ (**CA004_ 1**) village/neighborhood /

3. Other ______ (**CA004_2**) province_city_county/city/district _ _ // _____ (**CA004_3**) village/neighborhood /
4. Abroad

[IWER: county/city includes county-level administrative units/county-level city/district. : ///]

CA005 BRANCHPOINT:
IF WE KNOW WHETHER RS FATHER HAD GROWN UP IN AN URBAN OR A RURAL AREA AT RS LAST IW, GO TO CA006 BRANCHPOINT.

CA005 Did your father grow up in an urban area or a rural area?
1. City
2. Village

CA006 BRANCHPOINT:
IF WE KNOW WHAT RS FATHERS ZODIAC SIGN WAS AT RS LAST IW, GO TO CA007 BRANCHPOINT

[Show Card 2]

CA006 What is your father s Chinese Zodiac sign? ________
[IWER: Choose from the list of Chinese Zodiac signssee Appendix 1. :]

CA007 BRANCHPOINT:
IF WE KNOW THE BIRTH YEAR OF RS FATHER AT RS LAST IW, GO TO CA008 BRANCHPOINT

CA007 In what year was your father born? ____1850...1950 Year
[IWER: Mark the year using four digits. : 4]

CA008 BRANCHPOINT:
IF WE KNOW THE DEATH YEAR OF RS FATHER AT RS LAST IW, GO TO CA009 BRANCHPOINT

PROCEDURE :
If CA001 = 2, ask CA008; otherwise, skip it.

CA008 In what year did your father pass away? ___ 1900...2013 (**CA008_1**) year or ___ 1...120 (**CA008_2**) Years old .
[IWER: Mark the year using four digits. : 4]
[hard check: year > CA008. > CA007]

**CA009 BRANCHPOINT**: IF WE KNOW THE EDUCATION OF RS FATHER AT RS LAST IW, GO TO CA010 BRANCHPOINT

CA009 What is the highest level of education your father has completed? (not including adult education) ()

1. No formal education (illiterate) ( )
2. Did not finish primary school but capable of reading and/or writing
3. Sishu/home school
4. Elementary school
5. Middle school
6. High school
7. Vocational school ( )
8. Two-/Three-Year College/Associate degree
9. Four-Year College/Bachelors degree
10. Masters degree
11. Doctoral degree/ Ph. D.

**PROCEDURE** : If CA001 = 1, ask CA010-CA022_a; otherwise, ask CA014 and then skip to procedure before CA024

CA010 What is your fathers current marital status?

[IWERCommon- law marriage is considered as married.]

1. Marriedwith my mother
2. Marriedbut not with my mother
3. Separated ( )
4. Divorced
5. Widowed
6. Never married
7. Cohabitated

CA012 Does your father work currently (work includes agricultural work, earning wage work, self-employed activities, and unpaid family business work, et al.)? ()

1. Yes
2. No

CA013 How is your father s health? Very good, good, fair, poor or very poor?

1. Very good

2. Good
3. Fair
4. Poor
5. Very poor

CA014 Which is/was the highest occupation of your father? 1. Managers
2. Professionals and technicians
3. Clerks
4. Commercial and service workers
5. Agricultural, forestry, husbandry and fishery producers
6. Production and transportation workers 7. Cant be specified

CA015 What is your fathers average income at present? (not including the money from children, but including other all income like pension and government allowance). ( ) ______ yuan/year / (**CA015_1**) or _____ yuan/month / (**CA015_2**).

[Show Card 4]

[CAPI: Preload the Rs permanent address.]

CA016 Where does your father normally live?
1. The same household
2. The same or an adjacent dwelling/courtyard with me () ()
3. His birthplace
4. Another household in your permanent addresss village/neighborhood /
5. Another village/neighborhood in your permanent addresss county/city/district ______ village/neighborhood, how far away ______ km // / ______ / (**CA016_1**): ____ (**CA016_2**)
6. Other ______(**CA016_3**) province_city_county/city/district _ _ // ______(**CA016_4**) village/neighborhood /
7. Abroad

[IWER: county/city includes county-level administrative units/county-level city/district. : ///]

**PROCEDURE** :
If CA016 = 5/6/7, ask CA017.

CA017 What kind of location does your father live in?
1. City
2. County
3. Town
4. Village

CA018 Is his hukou in the same place as his current residence?
1. Yes → Skip to CA020 CA020
2. No
3. Does not have Hukou → Skip to CA021 CA021

[CAPI: Preload the Rs permanent address.]

CA019 What is the location of your fathers current hukou? 1. His birthplace
2. Your birthplace
3. Your permanent address s village/neighborhood /
4. Another village/neighborhood in your permanent addresss county/city/district ______ village/ neighborhood /// ______ / (**CA019_ 1**)
5. Other ______(**CA019_2**) province_city_county/ city/ district _ _ // _(**CA019_3**) village/neighborhood /
6. Abroad

[IWER: county/city includes county-level administrative units/county-level city/district. : ///]

CA020 What is your fathers current hukou status? 1. Agriculture Hukou
2. Non-Agriculture Hukou
3. Unified Residency Hukou
4. Do not have Hukou

[F1]

CA021 Does your father own a house?
1. Yes
2. No → Skip CA022 and CA022_a CA022 CA022_a

CA022 Do you know the present value of your fathers house? ___ 10000 Yuan

CA022_a Does your father shares the house with others?

1. with my mother
2. with other people
3. with my mother and other people
4. No

**PROCEDURE** :<br>Add one more section for respondents mother, with questions identical to those asked about the respondents father.

**PROCEDURE** :<br>if CV033 = 5, ask CA024

CA024 Do you keep in contact with your spouses parents? ()
1. Yes
2. No

**PROCEDURE** :<br>If widowed but keeping in contact with spouses parents CA024 = 1, then ask the same information about spouses parents. () (CA024 = 1)/.<br>If there are more than one spouses parents, then ask the last spouses parents. ()

CC SIBLINGS

[INTRO: Next I have some questions about your(spouses) brothers and sisters. ()]

CC002 How many of your living siblings are: Older brothers __0…25 (**CC002_1**), Younger brothers __0…25 (**CC002_2**) Older sisters __0…25 (**CC002_3**), Younger sisters __0…25 (**CC002_4**)

CC004 How many of your deceased siblings were: Older brothers __0…25 (**CC004_1**), Younger brothers __0…25 (**CC004_2**) Older sisters __0…25 (**CC004_3**), Younger sisters __0…25 (**CC004_4**)

CG Living Arrangements preferences

**PROCEDURE** :<br>Main respondent and spouse both answers CG001_W2 and CG002_W2 and no proxy is allowed for these two questions. CG001_W2 CG002_W2

CG001_W2 Suppose an elderly person has a spouse and adult children, and has good relationship with them, what do you think is the best living arrangement for the elderly person? ?

1. Live with adult children
2. Dont live with them in the same house, but live in the same community or village. /
3. Dont live with them in the same house and the same community or village. /
4. Live in a nursing house
5. Other

CG002_W2 Suppose an elderly person has no spouse but has adult children, and has good relationship with them. What do you think is the best living arrangement for him/her? ?

1. Live with adult children
2. Dont live with them in the same house, but live in the same community or village. /
3. Dont live with them in the same house and the same community or village. /
4. Live in a nursing house
5. Other

CC005_W2_1 If the main Rs spouse is in, please ask the spouse about spouses parents and siblings questions; if the spouse is not in, please ask main R for proxy. For the following questions about spouse s parents and siblings (Section CA/Section CC/Section CG), who answered the questions? Please record. / / /

1. Response answered him/herself
2. Spouse

CG003_W2 How often did the respondent receive assistance in answering section CA/CC/CG? CA/CC/CG

[IWER: If it is answered by a proxy, please record the respondents reaction.]

1. Never
2. A few times
3. Most or all of the time

CB CHILDREARING INFORMATION

[INTRO: Next we will ask some questions about your fostering of your children/about raising your children.]

PROCEDURE:
If the respondent is currently unmarried BE001 = 3 – 6, ask CB001-CB016. BE001 = 3 – 6CB001-CB016

CB003 How many biological children do you have who have passed away? __ 0…25 Persons
[IWER: Mark 0 if none. 0]

PROCEDURE:
If CB003= 0, then skip to CB001. CB003= 0CB001.

[CAPI: Preload all the living childrens name in the last wave.]

CB003_W2_1 What are the names of your deceased biological children?

[IWER: Choose all from the list; if it is not on the list, please choose other, and then fill in the names of those children. Pay attention to the near sound words.]

PROCEDURE:
Repeat questions CB004-CB008 for each child marked in CB003_W2_1 ; If the name is chosed from the list, then skip to CB007. CB004-CB008CB007.

CB004 When was CHILDns NAME born? [
____ 1900…2013 (**CB004_1**) year __0…12 (**CB004_2**) month

PROCEDURE:
If the person does not know the date ofbirth, CB004 = DK or CB004 = RF, skip CB005. CB004CB005.

CB005 Is your answer to CB020 based on the solar or the lunar calendar?
() ()
1. Solar calendar ( )
2. Lunar calendar ( )

CB006 Sex of CHILDns NAME []

1. Male
2. Female

CB007 When did CHILDns NAME pass away[] ____ 1900...2013 (**CB007_1**) year __0...12 (**CB007_2**) month

PROCEDURE :
If the person does not know the date ofbirth, CB007 = DK or CB007 = RF, skip CB008. CB007CB008.

CB008 Is your answer to CB007 based on the solar or the lunar calendar?
( ) ( )
1. Solar calendar ( )
2. Lunar calendar ( )

CB001 Have you ever given birth to any (other) child? If yes, how many are currently living? ( ) __ 0...25 Persons
[IWER: Mark 0 if none. 0]

PROCEDURE :
If CB001 ≠ 0, ask CB002; If CB001 = 0, go to CB011. CB001 ≠ 0CB002 CB001 = 0CB011.

CB001_W2_1 We recorded that [read all the preloaded childrens name] last wave, which [childrens name] is your biological children? [ ]
[IWER: Choose names of those children. Choose all that apply.]

[CAPI: Preload all the household members name.]

CB002 What are the name of your biological children
[IWER: Choose from the household member list, choose all that apply; if there is none, then fill in names of those children.]

CB011 How many adopted or foster children or step children do you have who have passed away? __ 0...25 Persons
[IWER: Mark 0 if none. 0]

PROCEDURE :
If CB011 = 0, then skip to CB009. CB011 = 0CB009.

[CAPI: Preload all the living childrens name in the last wave.]

CB011_W2_1 What are the names of your deceased adopted foster or step children?

[IWER: Choose all from the list; if it is not on the list, please choose other, and then fill in the names of those children. Pay attention to the near sound words.]

> **PROCEDURE** :
> Repeat questions CB012-CB016 for each child marked in CB011_W2_1who has passed away; If the name is chosed from the list, then skip to CB015. CB012-CB016CB015.

CB012 When was CHILDns NAME born []
____ 1900...2013 (**CB012_1**) year __0...12 (**CB012_2**) month

> **PROCEDURE** :
> If the person does not know the date ofbirth, CB012 = DK or CB012 = RF, skip CB013. CB012CB013.

CB013 Is your answer to CB012 based on the solar or the lunar calendar?

() ()

1. Solar calendar ()
2. Lunar calendar ()

CB014 Sex of CHILDns NAME []

1. Male
2. Female

CB015 When did CHILDns NAME pass away [] ____ 1900...2013 (**CB015_1**) year __0...12 (**CB015_2**) month

> **PROCEDURE** :
> If the person does not know the date ofbirth, CB015 = DK or CB015 = RF, skip CB016. CB015CB016.

CB016 Is your answer to CB015 based on the solar or the lunar calendar?

() ()

1. Solar calendar ()
2. Lunar calendar ()

CB009 Have you ever adopted or fostered any (other) child or (other) step child? If yes, how many are currently living? () () __ 0...25 Persons

[IWER: Mark 0 if none. 0]

**PROCEDURE** :<br>If CB009 ≠ 0, ask CB010; If CB009 = 0, go to the procedure before CB019. CB009 ≠ 0CB010; CB009 = 0CB019.

CB009_W2_1 We recorded that [read all the preloaded childrens name] last wave, which [childrens name] is your adopted or foster or step children? []

[IWER: Choose names of those children, choose all that apply.]

[CAPI Preload all the household members name.]

CB010 Whats the name of your adopted or foster children

[IWER: Choose from the household member list, choose all that apply; if there is none, then fill in names of those children.]

**PROCEDURE** :<br>If the respondent is currently married BE001 = 1/2/7, ask CB017 -CB048. BE001 = 1/2/7, CB017 -CB048

CB019 How many biological children do you and your (current) spouse have together who have passed away? ( ) __ 0…25 Persons

[IWER: Mark 0 if none. 0]

**PROCEDURE** :<br>If CB019 = 0, then skip to CB017. CB019 = 0CB017.

[CAPI: Preload all the living childrens name in the last wave.]

CB019_W2_1 What are the names of you and your (current) spouses deceased biological children?

[IWER: Choose all from the list; if it is not on the list, please choose other, and then fill in the names of those children. Pay attention to the near sound words.]

**PROCEDURE** :<br>Repeat questions CB020-CB024 for each child marked in CB019_W2_1who has passed away; If the name is chosed from the list, then skip to CB023. CB020-CB024CB023.

CB020 When was CHILDns NAME born []

____ 1900...2013 (**CB020_1**) year __0...12 (**CB020_2**) month

**PROCEDURE** :<br>If the person does not know the date ofbirth, CB020 = DK or CB020 = RF, skip CB021. CB020CB021.

CB021 Is your answer to CB020 based on the solar or the lunar calendar?

( ) ( )

1. Solar calendar ( )
2. Lunar calendar ( )

CB022 Sex of CHILDns NAME []

1. Male
2. Female

CB023 When did CHILDns NAME pass away [] ____ 1900...2013 (**CB023_1**) year __0...12 (**CB023_2**) month

**PROCEDURE** :<br>If the person does not know the date ofbirth, CB023 = DK or CB023 = RF, skip CB024. CB023CB024.

CB024 Is your answer to CB023 based on the solar or the lunar calendar?

( ) ( )

1. Solar calendar ( )
2. Lunar calendar ( )

CB017 Do you and your (current) spouse have any (other) biological children? If yes, how many are currently living? ( ) ( ) __ 0...25 Persons

**PROCEDURE** :<br>If CB017 ≠ 0, askCB018; ifCB017 = 0, go toCB027. CB017 ≠ 0CB018 CB017 = 0CB027.

CB017_W2_1 We recorded that [read all the preloaded childrens name] last wave, which [childrens name] is you and your (current) spouse have together? []

[IWER: Choose names of those children, choose all that apply.]

[CAPI: Preload all the household members name.]

CB018 What are the names of those children

[IWER: Choose from the household member list, choose all that apply; if there is none, then fill in names of those children]

CB027 How many additional biological children do you have who have passed away? （ ） __ 0...25 Persons

[IWER: Mark 0 if none. 0]

**PROCEDURE** :
If CB027 = 0, then skip to CB025. CB027 = 0CB025.

[CAPI: Preload all the living childrens name in the last wave.]

CB027_W2_1 What are the names of your deseased biological children?

[IWER: Choose all from the list; if it is not on the list, please choose other, and then fill in the names of those children. Pay attention to the near sound words.

**PROCEDURE** :
Repeat questions CB028-CB032 for each child marked in CB027_W2_1who has passed away; If the name is chosed from the list, then skip to CB031. CB028-CB032CB031.

CB028 When was CHILDns NAME born []

____ 1900...2013 (**CB028_1**) year __0...12 (**CB028_2**) month

**PROCEDURE** :
If the person does not know the date ofbirth, CB028 = DK or CB028 = RF, skip CB029. CB028CB029.

CB029 Is your answer to CB028 based on the solar or the lunar calendar?

() ()

1. Solar calendar （）
2. Lunar calendar （）

CB030 Sex of CHILDns NAME []

1. Male
2. Female

CB031 When did CHILDns NAME pass away [] ____ 1900…2013 (**CB031_1**) year __0…12 (**CB031_2**) month

PROCEDURE :
If the person does not know the date ofbirth, CB031 = DK or CB031 = RF, skip CB032. CB031CB032.

CB032 Is your answer to CB031 based on the solar or the lunar calendar?
() ()
1. Solar calendar ( )
2. Lunar calendar ( )

CB025 How many (other) additional biological children do you have who are currently living? __ 0…25 Persons

PROCEDURE :
If CB025 ≠ 0, ask CB026; if CB025 = 0, go to CB035. CB025 ≠ 0CB026 CB025 = 0CB035.

CB025_W2_1 We recorded that [read all the preloaded childrens name] last wave, which [childrens name] is your additional biological children? [ ]
[IWER: Choose names of those children, choose all that apply.]

[CAPI: Preload all the household members name.]

CB026 What are the names of those children
[IWER: Choose from the household member list, choose all apply; if there is none, then fill in names of those children]

CB035 How many additional biological children does your (current) spouse have who have passed away? () __ 0…25 Persons
[IWER: Mark 0 if none. 0]

PROCEDURE :
If CB035 = 0, then skip to CB033. CB035 = 0CB033.

[CAPI: Preload all the living childrens name in the last wave.]

CB035_W2_1 What are the names of your (current) spouses deceased biological children?

[IWER: Choose all from the list; if it is not on the list, please choose other, and then fill in the names of those children. Pay attention to the near sound words.]

PROCEDURE :
Repeat questions CB036-CB040 for each child marked in CB035_W2_1who has passed away; If the name is chosed from the list, then skip to CB039. CB036-CB040CB039.

CB036 When was CHILDns NAME born []
____ 1900…2013 (**CB036_1**) year __0…12 (**CB036_2**) month

PROCEDURE :
If the person does not know the date ofbirth, CB036 = DK or CB036 = RF, skip CB037. CB036CB037.

CB037 Is your answer to CB036 based on the solar or the lunar calendar?
( ) ( )
1. Solar calendar ( )
2. Lunar calendar ( )

CB038 Sex of CHILDns NAME []
1. Male
2. Female

CB039 When did CHILDns NAME pass away [] ____ 1900…2013 (**CB039_1**) year __0…12 (**CB039_2**) month

PROCEDURE :
If the person does not know the date ofbirth, CB039 = DK or CB039 = RF, skip CB040. CB039CB040.

CB040 Is your answer to CB039 based on the solar or the lunar calendar?
( ) ( )
1. Solar calendar ( )
2. Lunar calendar ( )

CB033 How many (other) additional biological children does your (current) spouse have who are currently living? ( ) ( ) __ 0…25 Persons

PROCEDURE :
If CB033 ≠ 0, ask CB034; if CB033 = 0, go to CB043. CB033 ≠ 0CB034 CB033 = 0CB043.

CB033_W2_1 We recorded that [read all the preloaded childrens name] last wave, which [childrens name] is your spouses additional biological children?

[IWER: Choose names of those children, choose all apply.]

[CAPI: Preload all the household members name.]

CB034 What are the names of those children

[IWER: Choose from the household member list, choose all that apply; if there is none, then fill in names of those children]

CB043 How many adopted or foster children do you or your (current) spouse have who have passed away? ( ) __ 0…25 Persons

[IWER: Mark 0 if none. 0]

PROCEDURE :
If CB043 = 0, then skip to CB041. CB043 = 0CB041.

[CAPI: Preload all the living childrens name in the last wave.]

CB043_W2_1 What are the names of your or your (current) spouses deceased adopted, foster or step children?

[IWER: Choose all from the list; if it is not on the list , please choose other, then fill in the names of those children. Pay attention to the near sound words.

PROCEDURE :
Repeat questions CB044-CB048 for each child marked in CB043_W2_1who has passed away; If the name is chosed from the list, then skip to CB047. CB044-CB048CB047.

CB044 When was CHILDns NAME born []

____ 1900…2013 (**CB044_1**) year __0…12 (**CB044_2**) month

PROCEDURE :
If the person does not know the date ofbirth, CB044 = DK or CB044 = RF, skip CB045. CB044CB045.

CB045 Is your answer to CB044 based on the solar or the lunar calendar?
( ) ( )
1. Solar calendar ( )
2. Lunar calendar ( )

CB046 Sex of CHILDns NAME []
1. Male
2. Female

CB047 When did CHILDns NAME pass away[] ____ 1900...2013 (**CB047_ 1**) year __0...12 (**CB047_2**) month

PROCEDURE :
If the person does not know the date ofbirth, CB047 = DK or CB047 = RF, skip CB048. CB047CB048.

CB048 Is your answer to CB047 based on the solar or the lunar calendar?
( ) ( )
1. Solar calendar ( )
2. Lunar calendar ( )

CB041 How many (other) adopted or foster children or step children do you or your（current）spouse have who are currently living? ( ) ( ) __ 0...25 Persons

PROCEDURE :
If CB041 ≠ 0, ask CB042; if CB041 = 0, go to procedure before CB049. CB041 ≠ 0 CB042, CB041 = 0, CB049.

CB041_W2_1 We recorded that [read all the preloaded childrens name] last wave, which [childrens name] is you or your spouses adopted or foster children? []
[IWER: Choose names of those children, choose all that apply.]

[CAPI: Preload all the household members name.]

CB042 What are the names of those children
[IWER: Choose from the household member list, choose all that apply; if there is none, then fill in names of those children]

PROCEDURE :
Repeat questions CB049 - CB079 for each living child. CB049 - CB079.

CB049 Is [CHILDs NAME] a boy or a girl? []

1. Boy (Son) ()
2. Girl (Daughter) ()

[Show Card 2]

CB050 What is the Chinese Zodiac sign for CHILDns NAME? [] ________

[IWER: Choose from the list of Chinese Zodiac signssee Appendix 1 :]

CB051 Birth month and year for CHILDns NAME? []

____ 1900...2013 (**CB051_1**) year __0...12 (**CB051_2**) month

[IWERThe year must be a number in the range [1900 – 2013]. Mark the year using four digits. Take down the month as its actual number. For example, write January as 1 not 01, December as 12. If do not remember month and day, fill ‘0. [1900 — 2013] 4 1 101,12 12 ‘0]

CB052 Is your answer to CB051 based on the solar or the lunar calendar?

() ()

1. Solar calendar ()
2. Lunar calendar ()

[Show Card 4]

[CAPI: Preload the Rs permanent address.]

CB053 Where does this CHILDns NAME normally live now? []

1. This household, and economicly dependent.
2. This household, but economicly independent.
3. The same or adjacent dwelling/courtyard with you () ()
4. Another household in your permanent addresss village/neighborhood /
5. Another village/neighborhood in your permanent addresss county/city/district; distance from here: ____km /// ______ / (**CB053_1**): ____(**CB053_2**)
6. Other ______(**CB053_3**) province_city_county/city/district _ _ // _(**CB053_4**) village/neighborhood /

7. Abroad

CB051 BRANCHPOINT:
If CB053 = 5 – 7, ASK CB054; OTHERWISE, GO TO CB055

CB054 In what type of location does CHILDns NAME live? []
1. City
2. County
3. Town
4. Village

CB055 What is the current hukou status of CHILDns NAME? [] 1. Agriculture Hukou → Skip to CB056 CB056
2. Non-Agriculture Hukou → Skip to CB056 CB056
3. Unified Residency Hukou
4. Does not have Hukou → Skip to CB058 CB058
F1

CB055_W2_1 What is [child name]s Hukou status before he/she has the unified residency hukou? []
1. Agriculture Hukou
2. Non-Agriculture Hukou
3. Do not have Hukou
F1

CB055_W2_2 When did [child name] have the unified residence Hukou? [] ____ 2000…2013 year
F1

CB056 Is CHILDns NAMEs hukou location the same as his/her place of residence? [ ] ?
1. Yes → Skip to CB081 BRANCHPOINT CB081 BRANCHPOINT 2. No

[CAPI: Preload the Rs permanent address.]

CB057 Where is the current hukou location of CHILDns NAME? []
1. This household
2. His|her birthplace /

3. Your permanent address s village/neighborhood /
4. Another village/neighborhood in your permanent addresss county/city/district /// ____ village/neighborhood / (**CB057_ 1**)
5. Other ______(**CB057_2**) province_city_county/city/district _ _ // _(**CB057_3**) village/neighborhood /
6. Other(specify) () ______(**CB057_4**)

[IWER: county/city includes county-level administrative units/county-level city/ district. : //.]

CB081 Where was the birth place of CHILDs NAME? [ ]

1. Your permanent address ‘s village/neighborhood /
2. Another village/neighborhood in your permanent address ‘s county/city/district /// ____ village/neighborhood / (**CB081_ 1**)
3. Other ______(**CB081_2**) province_city_county/city/district _ _ // _(**CB081_3**) village/neighborhood /
4. Abroad

CB082 Is [CHILDns NAME]s present hukou status and location the same as his/her first Hukou? [] /

1. Yes → Skip to CB058 BRANCHPOINT CB058 BRANCHPOINT 2. No

CB083 How did [CHILDns NAME]s Hukou status or location change? [ ]

1. Both Hukou status and location have changed 2. Only Hukou status has changed
3. Only Hukou location has changed → Skip to CB085 BRANCH- POINT CB085 BRANCHPOINT

CB084 What was [CHILDns NAME]s first HuKou status? [] 1. Agricultural Hukou
2. Non-agricultual Hukou

**CB085 BRANCHPOINT**:
IF CB083 = 2, GO TO CB058 BRANCHPOINT
IF WE KNOW THE FIRST HUKOU LOCATION OF [CHILDns NAME] AT THE LAST IW, GO TO CB058 BRANCHPOINT

CB085 What was the location of [CHILDns NAME]s first hukou? [ ]

1. Your permanent addresss village/neighborhood /

2. Another village/neighborhood in your permanent addresss county/city/district /// ______ (**CB085_1**)

3. Other ______ (**CB085_2**) province_city_county/city/district _ _ // ______ (**CB085_3**) village/neighborhood /

4. Abroad

**CB058 BRANCHPOINT**:
IF [CHILDns AGE] IS LESS THAN 6, GO TO NEXT CHILD

CB058 Is CHILDns NAME still in school now? [] 1. Yes

2. No → Skip to CB060 BRANCHPOINT CB060 BRANCHPOINT

CB059 What level of schooling and grade is CHILDns NAME currently enrolled in? [ ]

1. Primary school grade 1 1
2. Primary school grade 2 2
3. Primary school grade 3 3
4. Primary school grade 4 4
5. Primary school grade 5 5
6. Primary school grade 6 6
7. Middle school grade 1 1
8. Middle school grade 2 2
9. Middle school grade 3 3
10. Middle school grade 4 4
11. High school, grade 1 1
12. High school, grade 2 2
13. High school, grade 3 3
14. Vocational/technical high school year 1 1
15. Vocational/technical high school year 2 2
16. Vocational/technical high school year 3 3
17. College year 1 / 1
18. College year 2 / 2
19. College year 3 / 3
20. College year 4 / 4
21. College year 5 / 5
22. College year 6 / 6 6
23. Master s degree
24. Doctoral degree/Ph.D. degree

**PROCEDURE** :
Skip to CB063 BRANCHPOINT CB063 BRANCHPOINT

**CB060 BRANCHPOINT**:
IF [CHILDns AGE] IS LESS THAN 12, GO TO NEXT CHILD

CB060 What is the highest level of education CHILDns NAME completed? (not including adult education) [] ()

1. No formal education (illiterate) ( ) → Skip to CB063 BRANCH- POINT CB063 BRANCHPOINT
2. Did not finish primary school but capable of reading or writing → Skip to CB061 BRANCHPOINT CB061 BRANCHPOINT
3. Sishu/home school
4. Elementary school → Skip to CB062 BRANCHPOINT CB062 BRANCH- POINT
5. Middle school → Skip to CB062 BRANCHPOINT CB062 BRANCH- POINT
6. High school → Skip to CB062 BRANCHPOINT CB062 BRANCH- POINT
7. Vocational school ( ) → Skip to CB062 BRANCHPOINT CB062 BRANCHPOINT
8. Two-/Three-Year College / Associate degree → Skip to CB062 BRANCH- POINT CB062 BRANCHPOINT
9. Four-Year College / Bachelor s degree → Skip to CB062 BRANCHPOINT CB062 BRANCHPOINT
10. Post-graduate, Master s degree → Skip to CB062 BRANCHPOINT CB062 BRANCHPOINT
11. Post-graduate, doctoral degree/Ph.D. → Skip to CB063 BRANCHPOINT CB063 BRANCHPOINT

CB060_W2_1 How many years did CHILDNs NAME spend in sishu/home school? [ ] __ → Skip to CB063 BRANCHPOINT CB063 BRANCHPOINT

CB061 How many years did CHILDns NAME spend in primary school? [] __ → Skip to CB063 BRANCHPOINT CB063 BRANCHPOINT

CB062 How many additional years of schooling did CHILDns NAME receive after [THE AN- SWER CHOSEN IN CB060]? [] [CB060] __

**CB063 BRANCHPOINT**:<br>IF [CHILDns AGE] IS LESS THAN 16, GO TO NEXT CHILD

CB063 What is CHILDns NAME marital status? []

1. Married with spouse present
2. Married but not living with spouse temporarily for reasons such as work
3. Separated ( )
4. Divorced
5. Widowed

6. Never married
7. Cohabitated

**PROCEDURE** :<br>Ask CB064 if child has any college education, i.e. CB059 = 17 − 24 or CB060 = 8 − 11<br>(CB059 = 17 − 24 CB060 = 8 − 11) CB064

CB064 How much did you and your spouse spend to support this [CHILDns NAME]s college education [] ( ) _____ Yuan

**PROCEDURE** :<br>If the children has never married(CB063 = 6), please skip CB065 -CB068. (CB063 = 6)CB065 -CB068

CB065 How many children does [CHILDns NAME] have? [] __ 0...25 Persons

**PROCEDURE** :<br>If CB065 = 0, skip to CB067. CB065 = 0CB067.

CB066 How many children under age 16 does CHILDns NAME have? [] 16 __ 0...25 Persons

[Softcheck: if the number of sons is smaller than the number of adult sons. CB065 < CB066,]

CB067 How many grandchildren does CHILDns NAME have? [] __ 0...25 Persons

**PROCEDURE** :<br>If CB067 = 0, skip to CB069. CB067 = 0CB069.

CB068 How many grandchildren under age 16 does CHILDns NAME have? [] 16 __ 0...25 Persons

[Softcheck: if the number of sons is smaller than the number of adult sons. CB067 <CB068,]

CB069 Which category did the total income of CHILDns NAME (and his/her spouse) in the past year belong to? [](/)

1. None
2. under 2,000 yuan 2
3. 2 000 – 5 000 yuan 2 5
4. 5 000 – 10 000 yuan 5 1
5. 10,000 - 20,000 yuan 1 2
6. 20,000 - 50,000 yuan 2 5

7. 50,000 - 100,000 yuan 5 10
8. 100,000 - 150,000 yuan 10 15
9. 150,000 - 200,000 yuan 15 20
10. 200,000 - 300,000 yuan 20 30
11. Above 300,000 yuan 30

CB070 Is [Childs Name] working now (work includes agricultural work, earning wage work, self-employed activities, and unpaid family business work, et al.)? [] ()

1. Yes
2. No → Skip to CB074 CB074

[Softcheck: if the child is reported to be in school and also is reported to be working.]

[Show Card 5]

CB071 What is [CHILDns NAME]s main occupation? [] 1. Managers

2. Professionals and technicians
3. Clerks
4. Commercial and service workers
5. Agricultural, forestry, husbandry and fishery producers
6. Production and transportation workers

CB072 What is the highest administrative level that [CHILDns NAME] has attained? [ ]

1. Team Leader ( )
2. Section Chief
3. Director of a division
4. Director-General of a bureau and above
5. Township Leader
6. None

F1

CB073 What is/was your [CHILDns NAME] highest professional/technical level? [] /

1. Technician
2. Primary level
3. Intermediate level
4. Advanced level
5. None /

F1/

CB074 Has [Childs Name] ever worked before? (Work includes agricultural work, earning wage work, self-employed activities, and unpaid family business work, et al.) [ ] ( )

1. Yes
2. No → Skip to CB076 BRANCHPOINT CB076 BRANCHPOINT

[Show Card 5]

CB075 What sort of work did [CHILDns NAME] mainly do? [] 1. Managers

2. Professionals and technicians
3. Clerks
4. Commercial and service workers
5. Agricultural, forestry, husbandry and fishery producers
6. Production and transportation workers

CB076 Did [CHILDns NAME] live with others, away from you and your spouse, before age 16 for more than six months? [] 16 6

1. Yes
2. No → Repeat questions for additional children

CB077 What was the earliest age that [CHILDns NAME] lived separately with others for more than six months? [] ____ 1...16 years of age

[Softcheck: if the age reported here is older than the current age of the child. CB077]

CB078 With whom did [CHILDns NAME] live for the longest period of time when not living with you nor your spouse? []

1. Your parents
2. Your spouses parents
3. Your brothers or sisters family
4. Other family
5. Dormitory
6. Other

CB079 Cumulatively, how many years did CHILDns NAME live separately with others before age 16? []16

____0.00...16.00 (**CB079_1**)years __ 0...11 (**CB079_2**) months

PROCEDURE :
Repeat questions for additional children.

A OTHER HOUSEHOLD MEMBER

[INTRO: Relatives can have important effects on your life. Wed like to ask you some questions about other members of your household.]

[IWER: The names of other household members excluding MR, spouse, and parents/parents-in-law/children who have been asked in PART C, should be preloaded from PART CM. Ask the following questions on each of the other household members. PART C （） CM]

A006 What is the relationship of [household member name] to you? [] 1. Mother
2. Father
3. Mother-in-law /
4. Father-in-law /
5. Sibling
6. Brother-in-law, sister-in-law /
7. Child
8. Spouse of child /
9. Grandchild
10. Nanny
11. Driver
12. Other relative（specify）（） _____(**A006_1**)

A002 BRANCHPOINT:
IF THIS IS A NEW HOUSEHOLD MEMBER, GO TO A002.
IF THIS IS AN OLD HOUSEHOLD MEMBER AND GENDER IS NOT MISSING, GO TO A003 BRANCHPOINT.
IF THIS IS AN OLD HOUSEHOLD MEMBER AND GENDER IS MISSING, GO TO A002.

A002 Gender of [household member name] []
1. Male
2. Female

A003 BRANCHPOINT:

IF THIS IS A NEW HOUSEHOLD MEMBER, GO TO A003.

IF THIS IS AN OLD HOUSEHOLD MEMBER AND DATE OF BIRTH IS NOT MISSING, GO TO A004.

IF THIS IS AN OLD HOUSEHOLD MEMBER AND DATE OF BIRTH IS MISSING, GOTO A003.

A003 When was [household member name] born? []

____(**A003_1**)1900…2013 (HBirthyear) Year __(**A003_2**)0…12 (HBirthmonth) Month

[Show Card 1]

A004 What is [household member name]s marital status? []

[IWERCommon-law marriage is considered as married.]

1. Married with spouse present
2. Married but not living with spouse temporarily for reasons such as work
3. Separated ()
4. Divorced
5. Widowed
6. Never married
7. Cohabitated

A009 What is the current hukou status of [household member name]? []

1. Agriculture Hukou
2. Non-Agriculture Hukou
3. Unified Residency Hukou
   [IWER: for the place where agricultural hukou is abandoned.]
4. Do not have Hukou

F1

PROCEDURE :

Ask each person on the list.

PROCEDURE :

If the person is less than 6 years old, skip to the next person. 6

A013 Is [household member name] still in school now? []

1. Yes
2. No → Skip to procedure before A015 A015

[IWER: Fulltime student, not on-the-job student.]

A014 What level of schooling and grade is [household member name] currently enrolled in? []

1. Primary school grade 1 1
2. Primary school grade 2 2
3. Primary school grade 3 3
4. Primary school grade 4 4
5. Primary school grade 5 5
6. Primary school grade 6 6
7. Middle school grade 1 1
8. Middle school grade 2 2
9. Middle school grade 3 3
10. Middle school grade 4 4
11. High school, grade 1 1
12. High school, grade 2 2
13. High school, grade 3 3
14. Vocational/technical high school year 1 1
15. Vocational/technical high school year 2 2
16. Vocational/technical high school year 3 3
17. College year 1 / 1
18. College year 2 / 2
19. College year 3 / 3
20. College year 4 / 4
21. College year 5 / 5
22. College year 6 / 6 6
23. Master s degree
24. Doctoral degree/ Ph.D. degree

PROCEDURE :

If the person is less than 12 years old, skip to the next person. 12

A015 What is [household member name]s highest level of education completed? (not including adult education) [] () 1. No formal education (illiterate) ( )

2. Did not finish primary school but capable of reading or writing
3. Sishu/home school
4. Graduate from elementary school

5. Graduate from middle school
6. Graduate from high school
7. Graduate from vocational school ( )
8. Graduate from Two/Three Year College / Associate degree 9. Graduate from Four Year College / Bachelor s degree
10. Graduate from Post-graduate, Master s degree
11. Graduate from Post-graduate, Doctoral degree/Ph.D.

**PROCEDURE** : Go/Proceed to the next person.

C2 TIME TRANSFER AND TRANSFERS

[Introduction: In the following three parts: CD time transfer, CE transfer and CF time spent providing care. we will ask you how you contact with parents and children, and economic trancfers.]

CD TIME TRANSFER

CONTACT WITH PARENTS

**PROCEDURE** : Skip to CD003 if father/mother/father-in-law/mother-in-law is not alive OR father/mother/father-in-law/mother-in-law is household member. // ()/ () // ()/ () CD003.

**PROCEDURE** : Repeat each living and non-resident father/mother/father-in-law/mother-in-law for CD002-CD002_W2_1. // ()/ () CD002-CD002_W2_1.

**PROCEDURE** : IF PARENTS OR PARENTS-IN-LAW LIVE TOGETHER, THEN ASK CD002_W2_1. CD002_W2_1.

[Show Card 6]

CD002 How often do you/your spouse see your father/mother/ father-in-law/mother-in-law? // ()/ ()
1. Almost every day
2. 2-3 times a week 2-3
3. Once a week

4. Every two weeks
5. Once a month
6. Once every three months
7. Once every six months
8. Once a year
9. Almost never
10. Other

[Show Card 6]

CD002_W2_1/CD002_W2_2 How often do you/your spouse see your parents/ parents-in-law? / ( )

1. Almost every day
2. 2-3 times a week 2-3
3. Once a week
4. Every two weeks
5. Once a month
6. Once every three months
7. Once every six months
8. Once a year
9. Almost never
10. Other

[IWER: If the father and mother or father-in-law and mother-in-law live together, do not repeat the other one. /]

CONTACT WITH CHILDREN

**PROCEDURE** : If respondent has no non-cohabiting children, skip to CF001. CF001

**PROCEDURE** : For each of the non-coresident child, ask the following two questions CD003-CD004. CD003-CD004.

[Show Card 6]

CD003 How often do you see CHILDns NAME? [ ]?

1. Almost every day
2. 2-3 times a week 2-3
3. Once a week

4. Every two weeks
5. Once a month
6. Once every three months
7. Once every six months
8. Once a year
9. Almost never
10. Other

PROCEDURE :

IF CD003 = 1 – 3, skip CD004. CD003 = 1 – 3CD004.

[Show Card 6]

CD004 How often do you have contact with CHILDns NAME either by phone, text mess-age, mail, or email, when you didnt live with CHILDns NAME? [] [] ?

1. Almost every day
2. 2-3 times a week 2-3
3. Once a week
4. Every two weeks
5. Once a month
6. Once every three months
7. Once every six months
8. Once a year
9. Almost never
10. Other

CE TRANSFERS

[Introduction: Families sometimes help one another in a variety of ways, and each type of help can be important. The next questions are about help you (and your spouse) have given to or received from your non-coresident family members in the past year.]

[IWER: money/in-kind support means support living expenses / foodstuff / vegetables / clothes / water and electricity / telephone rate and other daily consumption; Marriage and funeral / move to new house / in hospital/ go to university / new born and other economic transfer. /]

CE002 BRANCHPOINT:

IF BOTH PARENTS WERE NOT LIVING AT LAST IW OR BOTH OF THEM ARE HOUSEHOLD MEMBERS , OR ONE PARENT WERE NOT LIVING ATLASTIW AND THE OTHER IS A HOUSEHOLD MEMBER , GO TO CE005 BRANCHPOINT

CE002 In the past year, how much economic supports did you or your spouse receive from your non-coresident parents?

1. Total money support _____ yuan (**CE002_1**), among which how much is regular ____ yuan (**CE002_2**). _____ (**CE002_1**) _____ (**CE002_2**) (for example, support living expenses/water and electricity/telephone rate/return loan or other cost in regular. /)
2. Total in-kind support _____ yuan (**CE002_3**), among which how much is regular _____ yuan (**CE002_4**). _____ (**CE002_3**) ____ (**CE002_4**) (for example, support food/vegetables/clothes or other in-kind sup- port in regular.)

[IWER: Regular means supporting at fixed time such as per month/quarter of a year/half of a year/year, etc.]

[IWER: If give no money or in-kind support, please fill in 0; if respondent answer RF or DK, then ask CE003, or skip to CE021. 0 CE003 CE021.]

CE003 Add unfolding brackets (100/200/400/800/1600 yuan) for CE002_1 or CE002_3 answered RF or DK. CE002_1CE002_3 （100/200/400/800/1600 ）

CE022 In the past year, how much economic supports did you or your spouse provide to your non-coresident parents?

1. Total money support _____ yuan (**CE022_1**), among which how much is regular _____ yuan (**CE022_2**). _____ (**CE022_1**) ____ (**CE022_2**) (for example, support living expenses/water and electricity/telephone rate/return loan or other cost in regular. /)
2. Total in-kind support _____ yuan (**CE022_3**), among which how much is regular _____ yuan (**CE022_4**). _____ (**CE022_3**) _____ (**CE022_4**) (for example, support food/vegetables/clothes or other in-kind sup- port in regular.)

[IWER: Regular means supporting at fixed time such as per month/quarter of a year/half of a year/year, etc.]

[IWER: If give no money or in-kind support, please fill in 0; if respondent answer RF or DK, then ask CE023, otherwise skip to CE005 BRANCHPOINT. 0CE023CE005 BRANCHPOINT.]

CE023 Add unfolding brackets (100/200/400/800/1600 yuan) for CE022_1 or CE022_3 answered RF or DK. CE022_1CE022_3 （100/200/400/800/1600 ）

CE005 BRANCHPOINT:
IF BOTH PARENTS-IN-LAW WERE NOT LIVING AT LAST IEW OR BOTH OF THEM ARE HOUSEHOLD MEMBERS, OR ONE PARENT-IN-LAW WERE NOT LIVING AT LAST IW AND THE OTHER IS A HOUSEHOLD MEMBER, GO TO CE009 BRANCHPOINT

CE005 In the past year, how much economic supports did you or your spouse receives from your non-coresident parents-in-law? （）

1. Total money support _____ yuan (**CE005_1**), among which how much is regular _____ yuan (**CE005_2**). _____ (**CE005_1**) ____ (**CE005_2**) (for example, support living expenses/water and electricity/telephone rate/return loan or other cost in regular. /)
2. Total in-kind support _____ yuan (**CE005_3**), among which how much is regular _____ yuan (**CE005_4**). _____ (**CE005_3**) _____ (**CE005_4**) (for example, support food/vegetables/clothes or other in-kind sup- port in regular.)

[IWER: Regular means supporting at fixed time such as per month/quarter of a year/half of a year/year, etc.]

[IWER: If give no money or in-kind support, please fill in 0; if respondent answer RF or DK, then ask CE006, otherwise skip to CE025. 0 CE006CE025.]

CE006 Add unfolding brackets (100/200/400/800/1600 yuan) for CE005_1 or CE005_3 answered RF or DK. CE005_1CE005_3 （100/200/400/800/1600 ）

CE025 In the past year, how much economic supports did you or your spouse provide to your non-coresident parents-in-law? （）

1. Total money support _____ yuan (**CE025_1**), among which how much is regular _____ yuan (**CE025_2**). _____ (**CE025_1**) ____ (**CE025_2**) (for example, support living expenses/water and electricity/telephone rate/return loan or other cost in regular. /)
2. Total in-kind support _____ yuan (**CE025_3**), among which how much is regular _____ yuan (**CE025_4**). _____ (**CE025_3**) _____ (**CE025_4**) (for example, support food/vegetables/clothes or other in-kind sup- port in regular.)

[IWER: Regular means supporting at fixed time such as per month/quarter of a year/half of a year/year, etc.]

[IWER: If give no money or in-kind support, please fill in 0; if respondent answer RF or DK, then ask CE026, otherwise skip to CE009 BRANCHPOINT. 0CE026CE009 BRANCHPOINT.]

CE026 Add unfolding brackets (100/200/400/800/1600 yuan) for CE025_1 or CE025_3 answered RF or DK. CE025_1CE025_3 （100/200/400/800/1600 ）

CE009 BRANCHPOINT:

IF THE RESPONDENT AND SPOUSE HAVE NO NON-CORESIDENT CHILDREN, GO TO CE012 BRANCHPOINT.

[CAPI: Preload all living childrens names.]

PROCEDURE :

For each non-coresident child ask CE009-CE030. CE009-CE030.

CE009 In the past year, how much economic supports did you or your spouse receive from your non-coresident children [child name]? []

1. Total money support _____ yuan (**CE009_1**), among which how much is regular _____ yuan (**CE009_2**). _____ (**CE009_1**) ____ (**CE009_2**) (for example, support living expenses/water and electricity/telephone rate/return loan or other cost in regular. /)
2. Total in-kind support _____ yuan (**CE009_3**), among which how much is regular _____ yuan (**CE009_4**). _____ (**CE009_3**) _____ (**CE009_4**) (for example, support food/vegetables/clothes or other in-kind sup- port in regular.)

[IWER: Regular means supporting at fixed time such as per month/quarter of a year/half of a year/year, etc.]

[IWER: If give no money or in-kind support, please fill in 0; if respondent answer RF or DK, then ask CE010, or skip to CE029. 0 CE010CE029.]

CE010 Add unfolding brackets (100/200/400/800/1600 yuan) for CE009_1 or CE009_3 answered RF or DK. CE009_1CE009_3 （100/200/400/800/1600 ）

CE029 In the past year, how much economic supports did you or your spouse provide to your non-coresident children [child name]? []

1. Total money support _____ yuan (**CE029_1**), among which how much is regular _____ yuan (**CE029_2**). _____ (**CE029_1**) ____ (**CE029_2**) (for example, support living expenses/water and electricity/telephone rate/return loan or other cost in regular. /)
2. Total in-kind support _____ yuan (**CE029_3**), among which how much is regular _____ yuan (**CE029_4**). _____ (**CE029_3**) _____ (**CE029_4**) (for example, support food/vegetables/clothes or other in-kind sup- port in regular.)

[IWER: Regular means supporting at fixed time such as per month/quarter of a year/half of a year/year, etc.]

[IWER: If give no money or in-kind support, please fill in 0; if respondent answer RF or DK, then ask CE030, or skip to CE012 BRANCHPOINT. 0CE030CE012 BRANCHPOINT.]

CE030 Add unfolding brackets (100/200/400/800/1600 yuan) for CE029_1 or CE029_3 answered RF or DK. CE029_1CE029_3 （100/200/400/800/1600 ）

CE012 BRANCHPOINT:

IF THE RESPONDENT AND SPOUSE HAVE NO NON-CORESIDENT GRANDCHILDREN OR GRANDCHILDREN AGE YOUNGER THAN 16, GO TO CE072_W2.

[CAPI: Preload all the childrens name. .]

CE012 Including both giving to you and receiving from you, did any of your non-coresident grandchildren have economic supports with you or your spouse in last year, if so, which child is the parent of him/her/them?

1. Choose childrens name
99 No grandchildren had economic support → Skip to CE072_W2 CE072_W2

[IWER: If there is no grandchildren had economics supports last year, choose the third choice. 3 .]

[IWER: If there are two or more than two children of Rs one child, then ask the total amount of the grandchildren from the same child.]

**PROCEDURE** :<br>Ask CE013-CE034 for each child recorded in CE012. CE012 CE013-CE034

CE013 In the past year, how much economic supports did you or your spouse receive from your non-coresident [child name]s children? []

1. Total money support _____ yuan (**CE013_1**), among which how much is regular _____ yuan (**CE013_2**). _____ (**CE013_1**) _____ (**CE013_2**) (for example, support living expenses/water and electricity/telephone rate/return loan or other cost in regular. /)
2. Total in-kind support _____ yuan (**CE013_3**), among which how much is regular _____ yuan (**CE013_4**). _____ (**CE013_3**) _____ (**CE013_4**) (for example, support food/vegetables/clothes or other in-kind sup- port in regular.)

[IWER: Regular means supporting at fixed time such as per month/quarter of a year/half of a year/year, etc.]

[IWER: If give no money or in-kind support, please fill in 0; if respondent answer RF or DK, then ask CE014, or skip to CE031. 0 CE014CE031.]

CE014 Add unfolding brackets (100/200/400/800/1600 yuan) for CE013_1 or CE013_3 answered RF or DK. CE013_1CE013_3 （100/200/400/800/1600 ）

CE031 In the past year, how much economic supports did you or your spouse provide to your non-coresident [child name]s children? []

1. Total money support _____ yuan (**CE031_1**), among which how much is regular _____ yuan (**CE031_2**). _____ (**CE031_1**) ____ (**CE031_2**) (for example, support living expenses/water and electricity/telephone rate/return loan or other cost in regular. /)
2. Total in-kind support _____ yuan (**CE031_3**), among which how much is regular _____ yuan (**CE031_4**). _____ (**CE031_3**) _____ (**CE031_4**) (for example, support food/vegetables/clothes or other in-kind sup- port in regular.)

[IWER: Regular means supporting at fixed time such as per month/quarter of a year/half of a year/year, etc.]

[IWER: If give no money or in-kind support, please fill in 0; if respondent answer RF or DK, then ask CE034, or skip to CE072_W2. 0 CE034CE072_W2.]

CE034 Add unfolding brackets (100/200/400/800/1600 yuan) for CE031_1 or CE031_3 answered RF or DK. CE031_1CE031_3 （100/200/400/800/1600 ）

CE072_W2 In the past year, how much economic supports did you or your spouse receive from your non-coresident siblings? Total money and in-kind support _______ yuan. _____

[IWER: If give no money nor in-kind support, please fill in 0; if respondent answered RF or DK, please ask CE073_W2, or skip to CE074_W2. 0 CE073_W2CE074_W2.]

CE073_W2 Add unfolding brackets (100/200/400/800/1600 yuan) for CE072_W2. CE072_W2（100/200/400/800/1600 ）

CE074_W2 In the past year, how much economic supports did you or your spouse provide to your non-coresident siblings? Total money and in-kind support _______ yuan. ______

[IWER:If give no money nor in-kind support, please fill in 0; if respondent answered RF or DK, please ask CE075_W2, or skip to CE016. 0 CE075_W2CE016.]

CE075_W2 Add unfolding brackets (100/200/400/800/1600 yuan) for CE074_W2. CE074_W2（100/200/400/800/1600 ）

CE016 In the past year, how much economic supports did you or your spouse receive from your non-coresident other relatives or friends? Total money and in-kind support ________ yuan. (for example, marriage and funeral/move to new house/new born/go to university, and economic aid for fall ill or difficult to live, but not including borrowing money). _____ ( )

[IWER: If give no money nor in-kind support, please fill in 0; if respondent answered RF or DK, please ask CE017, or skip to CE036. 0 CE017CE036.]

CE017 Add unfolding brackets (100/200/400/800/1600 yuan) forCE016. CE016 （100/200/400/800/1600 ）

CE036 In the past year, how much economic supports did you or your spouse provide to your non-coresident other relatives or friends? Total money and in-kind support ______ yuan. (for example, marriage and funeral/move to new house/new born/go to university, and economic aid for fall ill or difficult to live, but not including borrowing money). ( )

[IWER:If give no money nor in-kind support, please fill in 0; if respondent answered RF or DK, please ask CE037, or skip to CE066_W2. 0 CE037CE066_W2.]

CE037 Add unfolding brackets (100/200/400/800/1600 yuan) forCE036. CE036 （100/200/400/800/1600 ）

**CE066_W2 BRANCHPOINT**:<br>IF [CHILDNS NAME] HAS MARITAL HISTORY (CB063 = 1/2/3/4/5/7), ASK CE066_W2

[CAPI: Preload each child that is not unmarried.]

[IWER: please ask about each child CE066_W2-CE070_W2. CE066_W2-CE070_W2]

[IWER: If the child got married more than one times, please ask about the first one. 1 1]

CE066_W2 When did your [childns name] get married? []
____1900…2013 (**CE066_W2_1_1**) year __0…12 (**CE066_W2_1_2**) month __0…31 (**CE066_W2_1_3**) day

CE067_W2_1 Did you give betrothal gifts when [child name] got married? []

1. Yes
2. No → Skip to CE069_W2_1 CE069_W2_1

CE068_W2_1 At that time, how much was the total value of the betrothal gifts? ____

CE069_W2_1 Did you buy a house for him/her when [child name] got married? []

1. Yes
2. No → Skip to CF001 CF001

CE070_W2_1 At that time, how much was the total value of the house? ____

CF TIME SPENT PROVIDING CARE

PROCEDURE :
If the respondent has any grandchildren under 16, ask CF001 - CF003; otherwise, skip to CF004. 16 / CF001 - CF003 ; CF004 .

CF001 Did you spend any time taking care of your grandchildren or great-grandchildren last year?

1. Yes
2. No → Skip to the procedure before CF004 CF004

CF002 For which childs children or grandchildren did you provide care?

[IWER: Please list all the children including coresident ones, and add a choice deceased children in CAPI list. Select from list displayed by CAPI (childs name)]

PROCEDURE :
Repeat question CF003 according to the list of names in CF002. CF002 CF003

CF003 Approximately how many weeks and how many hours per week did you spend last year taking care of this child s children or grandchildren? Myself ____ (**CF003_1**)weeks ____ (**CF003_2**) 0.00...168.00 hours per week /, My spouse _____

(**CF003_3**) weeks ____ (**CF003_4**) 0.00…168.00 hours per week /
[IWER: Mark 1 if the period is less than 7 days. 7 1] [Hardcheck: if more than 52 weeks are reported or more than 140 hours are reported. 52 140]

PROCEDURE :

If both parents were not living at last IW or both of them are household members, or one parent were not living at last IW and the other is a household member, go to CF007_W2.

CF004 Did you or your spouse take care of your parents or parents-in-law during the last year in assisting them in their daily activities or other activities (e.g., household chores, meal preparation, laundry, going out, grocery shopping, financial management, etc.)? ( ) ( )

1. Yes
2. No → Skip to next module

CF005 Approximately how many weeks and how many hours per week did you yourself spend last year taking care of your parents or parents-in-law? ( )

1. Your father __ (**CF005_1**) weeks ; ___ (**CF005_2**) 0.00…168.00 hours per week /
2. Your mother __ (**CF005_3**) weeks ; ____ (**CF005_4**) 0.00…168.00 hours per week /
3. Your father-in-law ( ) __ (**CF005_5**) weeks ; ___ (**CF005_6**) 0.00…168.00 hours per week /
4. Your mother-in-law ( ) __ (**CF005_7**) weeks ; ___ (**CF005_8**) 0.00…168.00 hours per week /

[Softcheck: if (1) is checked in CF004 and 0 or missings are reported in CF005. CF004 = 1, CF005 = 0]

[Softcheck: if more than 52 weeks are reported or more than 140 hours are reported. 52 140]

CF006 Approximately how many weeks and how many hours per week did your spouse spend last year taking care of your parents or parents-in-law? ( )

1. Your father __ (**CF006_1**) weeks ; ___ (**CF006_2**) 0.00…168.00 hours per week /
2. Your mother __ (**CF006_3**)weeks ; ___ (**CF006_4**) 0.00…168.00 hours per week /
3. Your father-in-law ( ) __ (**CF006_5**) weeks ; ___ (**CF006_6**) 0.00…168.00 hours per week /

4. Your mother-in-law （） __ (**CF006_7**) weeks ; ___ (**CF006_8**) 0.00...168.00 hours per week /

[Softcheck: if (1) is checked in CF004 and 0 or missings are reported in CF006. CF004 = 1, CF006 = 0]

[Softcheck: if more than 52 weeks are reported or more than 140 hours are reported. 52 140]

CF007_W2 How often did the respondent receive assistance in answering sections CB, CD, CE and CF? CB, CD, CE CF

[IWER: If it is answered by a proxy, please record the respondents reaction. If the Family R isnt in or cant answer questions, and proxyed by his/her spouse, please choose the (4). （4）**]**

1. Never
2. A few times
3. Most or all of the time
4. Proxy by the spouse.

D HEALTH STATUS AND FUNCTIONING

DA HEALTH STATUS

Type of Interview R	
XRType = REIW	This is a reinterview R
XRType = NEWIW	This is a new interview R
XRType = EXITIW	This is a exit R

Gender of Interview R		
R IS MALE	(XRGender = 1)	(XRGender = 1) (XRGender =
R IS FEMALE	(XRGender = 2)	2)

Rs LAST IW Time (ZIWTime) (ZIWTime)

HEALTH CONDITIONS REPORTED IN LAST WAVE INTERVIEW:

ZDA005[i]	IF ZDA005[i]=Yes, R had kind of disabilities listed in DA005 at ZIWTime
ZDA006[i]	IF ZDA006[i]=Yes, R reported disabled time at ZIWTime
ZDA007[1]	IF DA007[1]=Yes, ZDA007[1]=Yes, R had hypertension at ZIWTime
ZDA007[2]	IF DA007[2]=Yes, ZDA007[2]=Yes, R had dyslipidemia at ZIWTime
ZDA007[3]	IF DA007[3]=Yes, ZDA007[3]=Yes, R had diabetes or high blood sugar at ZIWTime
ZDA007[4]	IF DA007[4]=Yes, ZDA007[4]=Yes, R had cancer or malignant tumor (excluding minor skin cancers) at ZIWTime
ZDA007[5]	IF DA007[5]=Yes or DA008[5]=Yes, ZDA007[5]=Yes, R had chronic lung diseases, such as chronic bronchitis, emphysema at ZIWTime
ZDA007[6]	IF DA007[6]=Yes, ZDA007[6]=Yes, R had liver disease at ZIWTime
ZDA007[7]	IF DA007[7]=Yes, ZDA007[7]=Yes, R had heart attack, coronary heart disease, angina, congestive heart failure, or other heart problems at ZIWTime
ZDA007[8]	IF DA007[8]=Yes, ZDA007[8]=Yes, R had stroke at ZIWTime
ZDA007[9]	IF DA007[9]=Yes, ZDA007[9]=Yes, R had kidney disease at ZIWTime
ZDA007[10]	IF DA007[10]=Yes, ZDA007[10]= Yes, R had stomach or other digestive disease (except for tumor or cancer) at ZIWTime
ZDA007[11]	IF DA007[11]= Yes, ZDA007[11]= Yes, R had emotional, nervous, or psychiatric problems at ZIWTime
ZDA007[12]	IF DA007[12]= Yes, ZDA007[12]= Yes, R had memory-related disease at ZIWTime

ZDA007[13]	IF DA007[13]= Yes, ZDA007[13]= Yes, R had arthritis or rheumatism at ZIWTime
ZDA007[14]	IF DA007[14]= Yes, ZDA007[14]= Yes, R had asthma at ZIWTime
ZDA008[1]	IF ZDA008[1]= Yes, R had known R had hypertension at ZIWTime
ZDA008[5]	IF ZDA008[5]= Yes, R had known R had chronic lung diseases at ZIWTime
ZDA008[11]	IF ZDA008[11]= Yes, R had known R had emotional problems at ZIWTime
ZDA009[i]	When was the condition first dignosed or known by yourself?
ZDA026	Began menarche time
ZDA027	IF DA027=Yes, ZDA027=Yes, R had started menopause at Rs last interview
ZDA028	Time of menopause
ZDA036	IF DA036= 1, ZDA036= 1, R had cataract surgery for one eye at ZIWTime
ZDA037	IF DA037=Yes, ZDA037=Yes, R had Glaucoma at ZIWTime
ZDA038	IF DA038=Yes, ZDA038=Yes, R had ever wear hearing aid at ZIWTime
ZDA040	IF ZDA040=Yes, R had lost all teeth at ZIWTime
ZDA048	How would you evaluate your health during childhood, up to and including age 15?
ZDA059	IF DA059=Yes, ZDA059=Yes, R had ever smoked at Rs last interview

NOTE: NOTE ON PRELOADED HEALTH CONDITIONS:

IN THIS SECTION MUCH OF THE FLOW OF THE INTERVIEW AND THE PHRASING OF THE QUESTIONS DEPENDS ON WHETHER THE RESPONDENT REPORTED HAVING CERTAIN HEALTH CONDITIONS IN A PREVIOUS IW AND/OR CONFIRMED THEM IN THE LAST IW.

DA HEALTH STATUS

PROCEDURE

SKIP PATTERN CHECKPOINT: SELF-REPORTED HEALTH STATUS

TWO SCALES ARE USED TO MEASURE SELF-REPORTED HEALTH STATUS. R WILL BE ASKED TO RATE THEIR HEALTH STATUS TWICE, ONCE AT THE BEGINNING OF THIS SECTION AND AGAIN AT THE END OF THE SECTION. QUESTION ORDER WILL BE ASSIGNED RANDOMLY.

IF R IS RANDOMLY ASSIGNED TO ORDER 1(SEC_DA_LIST= 1), SKIP TO DA001
IF R IS RANDOMLY ASSIGNED TO ORDER 2(SEC_DA_LIST= 2), SKIP TO DA002

（DA = 1）DA001
（DA = 2）DA002

PART I: GENERAL HEALTH STATUS AND DISEASE HISTORY

DA001 Next, I have some questions about your health. Would you say your health is excellent, very good, good, fair, or poor? [IWERInterviewer should read all the following options]

1. Excellent
2. Very good
3. Good
4. Fair
5. Poor

DA002 Next, I have some questions about your health. Would you say your health is very good, good, fair, poor or very poor? [IWERInterviewer should read all the following options]

1. Very good
2. Good
3. Fair
4. Poor
5. Very poor

**PROCEDURE** : IF XRType = REIW, ASK DA002_W2_1. DA002_W2_1

DA002_W2_1 Compared with your health when we talked with you in Rs LAST IW MONTH, YEAR, would you say that your health is better now, about the same, or worse? []

1. Better
2. About the same
3. Worse

[IWER: Please do not ask proxy the following questions from DA003-DA004. DA003- DA004]

DA003 **PROCEDURE** If XRType = NEWIW

Do you ever feel pain on the left side of your chest? **PROCEDURE** If XRType = REIW

Do you ever feel pain on the left side of your chest [since Rs LAST IW MONTH, YEAR/ in the last two years]? （）

1. Yes
2. No

DA004 **PROCEDURE** If XRType = NEWIW

Do you ever feel chest pains when climbing stairs/uphill or walking quickly?

PROCEDURE If XRType = REIW

Do you ever feel chest pains when climbing stairs/uphill or walking quickly [since Rs LAST IW MONTH, YEAR/ in the last two years]? （ ）

1. Yes
2. No
3. Not applicable

DA005 BRANCHPOINT:

IF XRType = REIW WHO DID HAVE DISABILITIE IN LAST IW TIME (ZDA005[i]=yes), GO TO DA007. DA007

IF THIS IS A NEW INTERVIEW R OR THIS IS A REINTERVIEW R WHO REPORTED NO IN LAST WAVE (ZAD005[i]=no).

DA005 Do you have one of the following disabilities? 1. Physical disabilities

2. Brain damage/mental retardation /
3. Vision problem
4. Hearing problem
5. Speech impediment

DA006 **PROCEDURE** If XRType = NEWIW AND DA005[i] = 1

In what year did you become disabled? [preload DA005]

PROCEDURE If XRType = REIW AND ZDA005[i] = null DA006

Our records from your last interview in Rs LAST IW MONTH, YEAR show that you have had [preload ZDA005] [...], In what year did you become disabled? [preload ZDA005]

____Year

[IWER: Mark the year using four digits. 4]

PROCEDURE :

IF XRType = NEWIW

[Show Card 7]

DA007 Have you been diagnosed with [conditions listed below, read one by one] by a doctor?

1. Hypertension
2. Dyslipidemia (elevation of low density lipoprotein, triglycerides (TGs),and total cholesterol, or a low high density lipoprotein level) （） 3. Diabetes or high blood sugar （）
4. Cancer or malignant tumor (excluding minor skin cancers) （ ）
5. Chronic lung diseases, such as chronic bronchitis , emphysema (excluding tumors, or cancer) （）
6. Liver disease (except fatty liver, tumors, and cancer) （ ）
7. Heart attack, coronary heart disease, angina, congestive heart failure, or other heart problems （）
8. Stroke
9. Kidney disease (except for tumor or cancer) （）
10. Stomach or other digestive disease (except for tumor or cancer) （）
11. Emotional, nervous, or psychiatric problems
12. Memory-related disease （）
13. Arthritis or rheumatism
14. Asthma

NOTE: The screen displays whether or not this condition was reported in Rs LAST IW

DA007_W2_1 Our records from your last interview in Rs LAST IW MONTH, YEAR show that you have had/not had [conditions listed below], is this right? / [],

1. Agree
2. Disagree

PROCEDURE :

For R reported in last iw that he/she had [conditions listed below] (ZDA007[i] = Yes OR ZDA008[i] = Yes) []:

If DA007_W2_1 = 1, skip to next loop

If DA007_W2_1 = 2, skip to DA007_W2_2 DA007_W2_2

PROCEDURE :

For R reported in last iw that he/she did not have [conditions listed below],(ZDA007[i] = not yes OR ZDA008[i] = not yes) []

If DA007_W2_1 = 1, skip to DA007_W2_2 DA007_W2_2

If DA007_W2_1 = 2, skip to EXDA009 DA009

DA007_W2_2 Have you been diagnosed with [conditions listed below, read one by one] by a doctor [since Rs LAST IW MONTH, YEAR/ in the last two years]? （ ）

1. Yes skip to DA008_W2_1 DA008_W2_1
2. No skip to next loop

DA008 BRANCHPOINT:

For XRType = REIW and XRType = NEWIW, ask DA008 if i = 1; 5; 11; DA007DA007_W2_1 15 11 DA008

DA008 Do you know if you have [preload the current choice in DA007]? [DA007 1 5 11]

1. Yes
2. No
3. Dont know

PROCEDURE :

IF DA007[i] = 1 or DA007_W2_1[i] = 1 or DA008[i] = 1

DA008_W2_1 How did you know that you had had [preload disease], through routine or charls physical examination, or any other? [ ] [] charls

1. Physical examination after had [preload disease] attack [] 2. Physical examination after had ill
3. Physical examination organized by work unit
4. Physical examination organized by community
5. charls physical examination charls
6. other

DA009 BRANCHPOINT:

For XRType = REIW, XRType = NEWIW and XRType = EXITIW, ask DA009, if DA007[i]= 1 or DA008[i]= 1 or ZDA009[i]= null. DA009 DA009

DA009 When was the condition first diagnosed or known by yourself? […] ____(**DA009_1**) Year ____(**DA009_2**) Age [IWER: Mark the year using four digits. : 4]

**PROCEDURE** :<br>Answer DA010 if you have No. 2; 5; 6; 7; 9; 10; 12; 13 chronic dieases of DA007. DA007 25679 10 12 13 DA010

DA010 Are you now taking any of the following treatments to treat […] or its complications (Check all that apply)? Taking Chinese traditional medicine, taking Western modern medicine, other treatments? […] ( ) () [IWER: Read one by one.]

1. Taking Chinese traditional medicine (**DA010_1**)
2. Taking Western morden medicine (**DA010_2**)
3. Other treatments (**DA010_3**)
4. None of the above (**DA010_4**)

**PROCEDURE** :<br>Answer DA010_W2_1 if you have hypertension or diabetes. DA010_W2_1

DA010_W2_1 Is your [Blood pressure/ sugar] generally under control? [/]

1. Yes
2. No

**PROCEDURE** :<br>IF XRType = REIW that had condition in last wave (ZDA007[i] = yes and ZDA007_W2_1[i]= 1): […]

DA010_W2_2 Compared to when we interviewed you in Rs LAST IW MONTH, YEAR, is your condition better, about the same as it was then or worse? ( ) […]

1. Better
2. Same
3. Worse

**PROCEDURE** : If respondents have hypertension then answer DA011. DA011

DA011 Are you now taking any of the following treatments to treat or control your hypertension?(Check all that apply) Taking Chinese traditional medicine, taking Western modern medicine? () [IWER: Read one by one. 1 = yes, 2 = no. 1 = 2 =]

1. Taking Chinese traditional medicine
2. Taking Western modern medicine
3. None of the above

DA011_W2_1 **PROCEDURE** If XRType = REIW

Since Rs LAST IW MONTH,YEAR, have you had your blood pressure checked by a doctor or nurse? []

PROCEDURE OTHERWISE:

Have you ever had your blood pressure checked by a doctor or nurse?

1. Yes
2. No skip DA011_W2_2 and DA012

DA011_W2_2 When did you last have it checked? Year ____ Month _____

DA012 During last year (last 12 months), how many times have you had blood pressure examination? （ 12 ）

____0 : : : 999 Times

DA013 Have your care providers ever given you health education/advice on the following (check all that apply)? Weight control, exercise, diet and/or smoking control? （）
[IWER: Read one by one. 1=yes, 2=no. 1= 2=]

1. Weight control
2. Exercise
3. Diet
4. Smoking control
5. None of the above

**PROCEDURE** If respondents have diabetes, then answer DA014-DA016. DA014-DA016

DA014 Are you now taking any of the folloing treatments to treat or control your diabetes?(Check all that apply) Taking Chinese traditional medicine, taking Western morden medicine ,taking insulin injections? （） [IWER: Read one by one.]

1. Taking Chinese traditional medicine
2. Taking Western modern medicine
3. Taking insulin injections

4. None of the above

DA015 During last year (last 12 months), how many times have you had the following?
(12)

1. Blood glucose test (**DA015_1**)____ 0...999 Times
2. Urine glucose test (**DA015_2**)_____ 0...99 Times
3. Fundus examination (**DA015_3**)_____ 0...99 Times
4. Micro-albuminuria test (**DA015_4**)_____ 0...99 Times 5. None of the above

DA016 Have your care providers ever given you health education/advice on the following? (check all that apply) ( ) [IWER: Read one by one. 1=yes, 2=no. 1= 2=]

1. Weight control
2. Exercise
3. Diet
4. Smoking control
5. Foot self-care
6. None of the above

**PROCEDURE** If reinterview and exit respondents have heart attack, then answer DA007_W2_5. DA007_W2_5

DA007_W2_5 [Since Rs LAST IW MONTH, YEAR/In the last two years], have you had a heart attack?

1. Yes
2. No → Skip to DA007_W2_6

DA007_W2_6 When was [his/her] (most recent) heart attack?
____ Year ____Month

**PROCEDURE** If respondents have cancer or maligant tumor (excluding minor skin cancers), answer DA017 and DA018. ( ) DA017 DA018

[Show Card 8]

DA017 In which organ or part of your body do you have cancer? Including the origins and metastasis of tumor. (circle all that apply) ( ) [IWER: Read one by one. We should still ask R even if he/she has already been cured.]

1. Brain

2. Oral cavity
3. Larynx
4. Other pharynx
5. Thyroid
6. Lung
7. Breast
8. Oesophagus
9. Stomach
10. Liver
11. Pancreas
12. Kidney
13. Prostate
14. Testicle
15. Ovary
16. Cervix
17. Endometrium
18. Colon or rectum
19. Bladder
20. Skin
21. Non-Hodgkin lymphoma （）
22. Leukemia
23. Other organ (**DA017_1**)

DA018 Have you taken any of the following treatments to treatyour cancer or relieve its/their symptoms (e.g., pain, nausea, etc.) in the past two years? (Check all that apply) Taking Chinese traditional medicine ,taking Western modern medicine ,chemotherapy ,surgery ,radiation therapy? ()

[IWER: Read one by one.]

1. Taking Chinese traditional medicine
2. Taking Western morden medicine
3. Chemotherapy
4. Surgery
5. Radiation therapy
6. None of the above

[F1 (1) ()

(2)

(3)]

**PROCEDURE** If respondents have stroke, then answer DA019. DA019

DA019 Are you now taking any of the folloing treatments because of your stroke?(Check all that apply) Taking Chinese traditional medicine, taking Western modern medicine, physical therapy, acupuncture and moxibustion, occupational therapy? （）
[IWER: Read one by one. 1 = yes, 2 = no. 1 = 2 =]

1. Taking Chinese traditional medicine
2. Taking Western morden medicine
3. Physical therapy
4. Acupuncture and moxibustion
5. Occupational therapy
6. None of the above

F1（1）（）

（2）

（3） / /]

PROCEDURE If reinterview respondents have stroke :

DA019_W2_1 Since Rs LAST IW MONTH, YEAR, has a doctor told you that you had another stroke? []

1. Yes
2. No

DA019_W2_2 When was your most recent stroke? _ Year ____Age

PROCEDURE If respondents have emotional, nervous, or psychiatric problems, then answer DA020 DA020

DA020 Are you now taking any of the folloing treatments for your emotional, nervous, or psychiatric problems?(Check all that apply) Receiving psychiatric or psychological treatment, taking anti depressants, taking tranquilizers or sleeping pills? （） [IWER: Read one by one. 1 =yes, 2 =no. 1 = 2 =]

1. Receiving psychiatric or psychological treatment 2. Taking anti depressants

3. Taking tranquilizers or sleeping pills
4. None of the above

[IWER: Please do not ask proxy the following questions from DA021-DA078. DA021- DA078]

DA021 **PROCEDURE** If XRType = NEWIW

Have you ever been in a traffic accident or any other kind of major accidental injury and received medical treatment?

PROCEDURE If XRType = REIW

Have you ever been in a traffic accident or any other kind of major accidental injury and received medical treatment [since Rs LAST IW MONTH, YEAR/ in the last two years]? (),

1. Yes
2. No → Skip to DA023 DA023

DA022 Does your injury caused by the accident limit your daily activities?

1. Yes
2. No

DA023 Have you fallen down since Rs LAST IW MONTH, YEAR? []

1. Yes
2. No → Skip to DA025 DA025

DA024 How many times have you fallen down seriously enough to need medical treatment?

?____ times

DA025 **PROCEDURE** If XRType = NEWIW

Have you ever fractured your hip?

PROCEDURE If XRType = REIW

PREVIOUS WAVE (ZDA025): [Yes/No/Unknown] (ZDA025): [//]

1. Yes
2. No

F1 ,

SKIP PATTERN CHECKPOINT: PROSTATE ILLNESS/ INCONTINENCE : /

**PROCEDURE** IF R IS MALE, SKIP TO DA029. DA029

**BRANCHPOINT**:<br>IF XRType = REIW REPORTED IN PREVIOUS WAVE THAT HAS NOT STARTED MENOPAUSE , SKIP TO DA027 DA027<br>IF XRType = REIW REPORTED IN PREVIOUS WAVE THAT HAS STARTED MENOPAUSE, SKIP TO DA032 DA032<br>IF XRType = NEWIW DA026-DA028

DA026 When did you begin the menarche?

____1900...2011 (**DA026_1**) Year Or Age ____1...120 (**DA026_2**) Years

[IWER: Mark the year using four digits. : 4]

DA027 Have you started menopause?

1. Yes
2. No → Skip to DA032 DA032

DA028 When did you begin the menopause?

____ (**DA028_1**) Year Or Age ____ (**DA028_2**) Years → Skip to DA032 DA032

[IWER: Mark the year using four digits. : 4]

DA029 **PROCEDURE** If XRType = NEWIW AND IS MALE

Have you ever been diagnosed with a prostate illness, such as prostate hyperplasia （excluding prostatic cancer） ? ( )

1. Yes Skip to DA030 DA030
2. No

PROCEDURE If XRType = REIW AND IS MALE

Have you ever been diagnosed with a prostate illness, such as prostate hyperplasia （excluding prostatic cancer） since we talked (in Rs LAST IW MONTH,YEAR/in the last two years)? （） ()

1. Yes → Skip to Skip to DA030 DA030
2. No

F1

DA029_W2_1 Do you know if you had a prostate illness, such as prostate hyperplasia （excluding prostatic cancer）? ( )

1. Yes
2. No → Skip to DA032 DA032
3. Dont know → Skip to DA032 DA032

DA030 When was the condition first diagnosed? __(**DA030_1**)Year Or Age ____ (**DA030_2**) Years [IWER: Mark the year using four digits. : 4]

DA031 Are you now taking medication or other treatment for your prostate illness?

1. Yes
2. No

DA032 Now I have some questions about your eyesight. Do you usually wear glasses or corrective lenses? ( )

1. Yes
2. Legally blind → Skip to DA038 DA038
3. No
4. Sometimes

DA033 How good is your eyesight for seeing things at a distance, like recognizing a friend from across the street (with glasses or corrective lenses if you wear them)? Would you say your eyesight for seeing things at a distance is excellent, very good, good, fair, or poor? ()

1. Excellent
2. Very good
3. Good
4. Fair
5. Poor

DA034 How good is your eyesight for seeing things up close, like reading ordinary newspaper print (with glasses or corrective lenses if you wear them)? Would you say your eyesight for seeing things up close is excellent, very good, good, fair, or poor?

1. Excellent
2. Very good
3. Good
4. Fair
5. Poor

**PROCEDURE** If XRType = NEWIW:

DA035 Have you ever had cataract surgery?

1. Yes
2. No → Skip to DA037 DA037

**PROCEDURE** IfXRType = REIWAND R REPORTED IN LAST IW THAT HAD CATARACT SURGERY ON ONE EYE(ZDA036 = 1): (ZDA036 = 1):

[IWER: PREVIOUSLY REPORTED CATARACT SURGERY ON ONE EYE]

DA035_W2_1 Have you had another cataract surgery since we last talked to you (in Rs LAST IW MONTH, YEAR) other than what you told us about then? ( ) ( )

1. Yes
2. No

**PROCEDURE** If XRType = REIW AND R DID NOT REPORT IN LAST IW THAT HAD CATARACT SURGERY ((ZDA035 ! = 1) :

DA035_W2_2 Have you had cataract surgery (since Rs LAST IW MONTH, YEAR/in the last two years)? ( )

1. Yes
2. No

DA036 Have you had cataract surgery on both eyes or just one?

1. One eye only
2. Both eyes

**PROCEDURE** If XRType = NEWIW OR XRType = REIW AND R DID NOT REPORT IN LAST IW THAT HAD GLAUCOMA(ZDA037 = 2): (ZDA037 ! = 1)

DA037 Has a doctor/nurse/paramedical/ doctor of traditional Chinese medicine doctor ever treated you for glaucoma?

1. Yes
2. No

**PROCEDURE** If XRType = REIW AND R REPORTED IN LAST IW THAT HAD GLAUCOMA(ZDA037 = 1): (ZDA037 = 1)

DA037_W2 You told us you had glaucoma, has a doctor/nurse/paramedical/ doctor of traditional Chinese medicine doctor ever treated you for glaucoma relapses since last iw time? []

1. Yes
2. No
3. Never had glaucoma

**PROCEDURE** IF R REPORTED IN PREVIOUS IW THAT WEARS HEARING AID (ZDA038 = 1), SKIP TO DA039 DA039

DA038 Now I have some questions about your hearing. Do you ever wear a hearing aid?

1. Yes
2. No

DA039 Is your hearing very good, good, fair, poor, or very poor (with a hearing aid if you normally use it and without if you normally don t)? Would you say your hearing is excellent, very good, good, fair, or poor? ( )

1. Excellent
2. Very good
3. Good
4. Fair
5. Poor

**PROCEDURE** If XRType = NEWIW or XRType = REIW that did not lost all teeth in last iw(ZDA040 ≠ 1), ask DA040: , DA040

DA040 Have you lost all of your teeth?

1. Yes
2. No

[Now, please pause briefly to think about YESTERDAY, from the morning until the end of the day. Think about where you were, what you were doing, who you were with, and how you felt.]

WB01 What day-of-the-week was it yesterday? (Mark (X) one box.)

1. Right
2. Wrong
3. Dont know

WB02 What was the date yesterday? (Month / day)

1. Month correct
2. Day correct
3. Both error

WB03 What time did you wake up yesterday? (Hour, Minute, AM/PM, 12hours system) （12 ）

____ ____
____ ____

WB04 What time did you go to sleep at the end of the day yesterday? (Hour, Minute, AM/PM, 12hours system) （12 ）

____ ____
____ ____

[The next questions are about your experiences yesterday. Mark (X) in one box for each line for the extent you felt the following.]
[Show Card 10]

WB05 Yesterday, did you feel Frustrated

1. Not at all
2. A little
3. Somewhat
4. Quite a bit
5. Very

[Show Card 10]

WB06 Yesterday, did you feel Sad?

1. Not at all
2. A little
3. Somewhat
4. Quite a bit
5. Very

[Show Card 10]

WB07 Yesterday, did you feel Enthusiastic? 1. Not at all

2. A little
3. Somewhat
4. Quite a bit
5. Very

[Show Card 10]

WB08 Yesterday, did you feel Lonely?

1. Not at all
2. A little
3. Somewhat
4. Quite a bit
5. Very

[Show Card 10]

WB09 Yesterday, did you feel Content?

1. Not at all
2. A little
3. Somewhat
4. Quite a bit
5. Very

[Show Card 10]

WB10 Yesterday, did you feel Worried?

1. Not at all
2. A little
3. Somewhat
4. Quite a bit
5. Very

[Show Card 10]

WB11 Yesterday, did you feel Bored?

1. Not at all
2. A little
3. Somewhat
4. Quite a bit
5. Very

[Show Card 10]

WB12 Yesterday, did you feel Happy?

1. Not at all
2. A little
3. Somewhat
4. Quite a bit
5. Very

[Show Card 10]

WB13 Yesterday, did you feel Angry?

1. Not at all
2. A little
3. Somewhat
4. Quite a bit
5. Very

[Show Card 10]

WB14 Yesterday, did you feel Tired?

1. Not at all
2. A little
3. Somewhat
4. Quite a bit
5. Very

[Show Card 10]

WB15 Yesterday, did you feel Stressed?

1. Not at all
2. A little
3. Somewhat
4. Quite a bit
5. Very

WB16 Yesterday, did you feel any pain?

1. None → Skip DA042 and DA042_W2_1 DA042 DA042_W2_1 2. A little
3. Some
4. Quite a bit
5. A lot

[Show Card 9]

DA042 On what part of your body do you feel pain? Please list all parts of body you are currently feeling pain.

1. Head (Headache)
2. Shoulder
3. Arm
4. Wrist
5. Fingers

6. Chest
7. Stomach (Stomachache)
8. Back
9. Waist
10. Buttocks
11. Leg
12. Knees
13. Ankle
14. Toes
15. Neck

DA042_W2_1 Are you taking measures to reduce the pain? ()

1. Taking Chinese traditional medicine
2. Taking Western modern medicine
3. Acupuncture treatment
4. Professional massage therapy
5. Other
6. None

DA045 Are there any other medical diseases or conditions that are important to your health now that we have not talked about?

1. Yes → Skip to DA046 DA046
2. No → Skip to DA047 DA047

DA046 What illness is that? _____

DA047 Have you gained or lost 5 or more kilograms in the last year? (excluding pregnancy) 10 10 ()

1. Yes, I only gained weight
2. Yes, I only lost weight
3. Yes, I first gained and then lost weight
4. Yes, I first lost and then gained weight
5. No
6. I dont know

**PROCEDURE** If XRType = NEWIW(ZDA048 = null):

DA048 How would you evaluate your health during childhood, up to and including age 15? Excellent, very good, good,fair, poor? 15 (15)

1. Excellent
2. Very Good

3. Good
4. Fair
5. Poor

PART II: LIFESTYLE AND HEALTH BEHAVIORS

DA049 During the past month, how many hours of actual sleep did you get at night (average hours for one night)? (This maybe shorter than the number of hours you spend in bed.) ( )

____0...24 hours

DA050 During the past month, how long did you take a nap after lunch? ____minutes [I WERIfR didnt take a nap, please record for 0. 0]

**PROCEDURE** DA051 will be presented ONLY to a random subsample of households (half). Main respondent and spouse in the selected households should answer DA051 DA051DA051

DA051 Now we would like to ask about the amount of time you spend on different types of physical activities in a usual week.

DA051_1 Whats the purpose for doing these physical activities, for entertainment, job demand or exercise in doing these physical activities?

1. Job demands
2. Entertainments
3. Exercise
4. Other

[ACTIVITIES IN LAST MONTH]
[Show Card 11]

DA056 Have you done any of these activities in the last month? (Code all that apply) ()

1. Interacted with friends
2. Played Ma-jong, played chess, played cards, or went to community club
3. Provided help to family, friends, or neighbors who do not live with you and who did not pay you for the help
4. Went to a sport, social, or other kind of club

PHYSICAL ACTIVITIES (KKTYPE)	DA051 During a usual week, did you do any [...] for at least 10 minutes continuously? [...]	DA052 During a usual week, on how many days did you do [...] for at least 10 minutes? [...]	How much time did you usually spend doing [...] on one of those days? [...] [...]	
A. Now, think about all the vigorous activities requiring hard/high-intensity physical effort that you do in a usual week. Vigorous activities make you breathe much harder than normal and may include heavy lifting, digging, plowing, aerobics, fast bicycling, and cycling with a heavy load. Think only about those physical activities that you did for at least 10 minutes at a time.	1 Yes !<br>2 No #	____ 1 ... 7 days	DA053<br>1. < 2 hours<br>2. ≥ 2 hours	DA054<br>1. < 30 minutes<br>2. ≥ 30 minutes<br>DA055<br>3. < 4 hours<br>4. ≥ 4 hours
B. Now think about activities which take moderate physical effort that you do in a usual week. Moderate physical activities make you breathe somewhat harder than normal and may include carrying light loads, bicycling at a regular pace, or mopping the floor. Again, think about only those physical activities that you did for at least 10 minutes at a time.	2 No #<br>1 Yes !	____ 1 ... 7 days	DA053<br>1. < 2 hours<br>2. ≥ 2 hours	DA054<br>1. < 30 minutes 2. ≥ 30 minutes<br>DA055<br>3. < 4 hours<br>4. ≥ 4 hours
C. Now think about the time you spend walking in a usual week. This includes at work and at home, walking to travel from place to place, and any other walking that you might do solely for recreation, sport, exercise, or leisure.	2 No #<br>1 Yes !	____ 1 ... 7 days	DA053<br>1. < 2 hours<br>2. ≥ 2 hours	DA054<br>1. < 30 minutes<br>2. ≥ 30 minutes<br>DA055<br>3. < 4 hours<br>4. ≥ 4 hours

5. Took part in a community-related organization 6. Done voluntary or charity work
7. Cared for a sick or disabled adult who does not live with you and who did not pay you for the help
8. Attended an educational or training course 9. Stock investment （ ）
10. Used the Internet
11. Other
12. None of these

[CHECK: You cannot select None of these together with any other answer. Please change your answer.]

PROCEDURE IF cnt INDA056 (ACTIVITIES IN LAST MONTH) DA056 1-11 （）

DA057 Frequency of activity in the last month

How often in the last month [did/have][you] [do voluntary or charity work/cared for a sick or disabled adult/provided help to family, friends or neighbors/attended an educational or training course/ Interacted with friends /go to a sport,social or other kind of club/taken part in a community-related organization]? Almost daily, almost every week, or not regularly? ( )

1. Almost daily
2. Almost every week
3. Not regularly

DA058 How many meals do you normally eat every day? More than 4 meals per day,4 meals per day,3 meals per day ,2 meals per day,1 meal per day or <1 meal per day? 4 , 4 , 3 , 2 , , ?

[IWER: Read each choice of the answers.]

1. More than 4 meals per day 4
2. 4 meals per day 4
3. 3 meals per day 3
4. 2 meals per day 2
5. 1 meal per day
6. <1 meal per day

[INTRO: Next, I would like to ask whether you have had the habit of smoking cigarettes/smoking a pipe/chewing tobacco, now or in the past. By smoking we mean smoking more than 100 cigarettes in your life) (100)]

BRANCHPOINT:

IF XRType = REIWAND R REPORTED EVER SMOKED(ZDA050 = 1), SKIP TO DA061. DA061

IF XRType = REIW AND R DID NOT REPORT EVER SMOKED(ZDA050 not 1); OR XRType = NEWIW, ASK DA059: hyperref[DA059]DA059

DA059 Have you ever chewed tobacco, smoked a pipe, smoked self-rolled cigarettes, or smoked cigarettes/cigars? ()

1. Yes
2. No Skip to DA067 DA067

DA061 **PROCEDURE** If XRType = REIW

Our records from your last interview in Rs LAST IW MONTH, YEAR show that you have ever smoked,

Do you still have the habit or have you totally quit? **PROCEDURE** If XRType = NEWIW

Do you still have the habit or have you totally quit?

1. Still have Skip DA062 DA062
2. Quit

DA062 At what age did you totally quit smoking? Age ____1...120 (**DA062_1**) years Or _ 1900...2011 (**DA062_2**) Year [IWER: Mark the year using four digits. : 4]

DA063 In one day about how many cigarettes do/did you consume [preload:now/before totally quitting]? [/] ____cigarettes

DA064 How much does/did it cost per pack = 20 cigarettes? [/] (20) ____Yuan [IWER: Prompt R: were asking price at that time, not current price]

**PROCEDURE** If XRType = REIW THAT HAS BEEN ASKED WHEN STARTED SMOKING(ZDA065 not null), SKIP DA065. DA065

DA065 At what age did you start to smoke on a regular basis?

Age ____ 1...120 (**DA065_1**) years Or ____ 1900...2011 (**DA065_2**) Year [IWER: Mark the year using four digits. : 4]

DA066 How soon after you wake up did/do you smoke your first cigarette, cigar, or pipe? [/]

1. Within 5 minutes 5
2. Within 6-30 minutes 6-30
3. Within 31-60 minutes 31-60
4. More than 1 hour 1

DA067 Did you drink any alcoholic beverages, such as beer, wine, or liquor in the past year? How often?

1. Drink more than once a month.
2. Drink but less than once a month Skip to DA069 DA069
3. None of these Skip to DA069 DA069

DA068 What type of alcoholic beverages did you drink? Liquor, wine, or beer(code all that apply) ( )

1. Liqor, including white liquor, whisky, and others Skip to DA071 DA071

2. Beer Skip to DA071 DA071
3. Wine or rice wine Skip to DA071 DA071

**PROCEDURE** If XRType = REIW THAT HAS BEEN ASKED WHETHER DRINKING ALCOHOLIC BEVERAGES IN THE PAST, SKIP DA069. DA069

DA069 Did you ever drink alcoholic beverages in the past? How often?

1. I never had a drink. → Skip to procedure before DA079 DA079
2. I used to drink less than once a month. → Skip to procedure before DA079 DA079
3. I used to drink more than once a month.

**PROCEDURE** IfXRType = REIW THAT HAS BEEN ASKED WHEN QUITING ALCOHOLIC BEVERAGES IN THE PAST, SKIP DA070. DA070

DA070 When did you quit or reduce drinking?

____1900...2011 (**DA070_1**)Year Or age : _____1...120 (**DA070_2**) Years

[IWER: Record year in 4 digits. 4]

**PROCEDURE** IfXRType = REIW THAT HAS BEEN ASKED WHEN STARTED DRINKING, SKIP DA071. DA071

DA071 When did you start drinking?

____1900...2011 (**DA071_1**)Year Or age : _____1...120 (**DA071_2**) Years

[IWER: Record year in 4 digits. 4]

SKIP PATTERN CHECKPOINT: DRINKING

[INTRO: Now, I am going to ask you how often and how much you drank during the past year. Please tell me how often you drank per month, and how much you drank at a time on average. I will repeat the questions for different types of alcoholic beverages.]

**CAPI** If DA067 answered 1, ask DA072: DA067 1DA072

DA072 How often did you drink liquor, including white liquor, whisky, and others per month in the last year

1. Once a month
2. 2-3 times a month 2-3
3. Once a week
4. 2-3 times a week 2-3

5. 4-6 times a week 4-6
6. Once a day
7. Twice a day
8. More than twice a day

DA073 The last time you drank liquor last year, how many liang of liquor did you drink? (1 liang = 50cc/50ml) （1 = 50 ） ____liang

**CAPI** If DA068 = 2, ask DA074. DA068 2DA074]

DA074 How many times per month did you drink beer in the last year ?

1. Once a month
2. 2-3 times a month 2-3
3. Once a week
4. 2-3 times a week 2-3
5. 4-6 times a week 4-6
6. Once a day
7. Twice a day
8. More than twice a day

DA075 The last time you drank beer last year, how many bottles of beer did you drink? (1bottle= 2:5 mugs, 1mug= 220cc) ?（ 1 =2.5 1 =220 ） ____ (**DA075_1**)0…120bottles or ____(**DA075_2**)0…300mugs

**CAPI** If DA068 = 3, ask DA076. DA068 3DA076]

DA076 How often did you drink wine or rice wine per month in the last year?

1. Once a month
2. 2-3 times a month 2-3
3. Once a week
4. 2-3 times a week 2-3
5. 4-6 times a week 4-6
6. Once a day
7. Twice a day
8. More than twice a day

DA077 The last time you drank it last year, how many liang of wine did you drink? (1 liang=50cc) （1 =50 ） ____0.00…100.00liang

DA078 In the last year, have you ever taken a drink first thing in the morning to steady your nerves or get rid of a hangover?

1. Yes
2. No

[IWER: Please do not ask proxy the following question DA079 and DA080. DA079 and DA080]

DA079 How would you rate your health status? Would you say your health is very good, good, fair, poor or very poor?

1. Very good
2. Good
3. Fair
4. Poor
5. Very poor

DA080 Next I have some questions about your health. Would you say your health is excellent, very good, good, fair, or poor? [IWERinterviewer should read all the following options.]

1. Excellent
2. Very good
3. Good
4. Fair
5. Poor

**INTERVIEWER CHECK AGE OF RESPONDENT?**	**1.** < 65 **YEAR !COLUMN A A** **2.** 65 - 69 **YEAR !COLUMN B B** 74 **3.** 70 - **YEAR !COLUMN C C** 79 **4.** 75 - **YEAR !COLUMN D D** 84 **5.** 80 - **YEAR !COLUMN E E**				**6.** 85 - 89 **YEAR !COLUMN F F 7.** 90 - 94 **YEAR !COLUMN G G 8.** 95 - 99 **YEAR !COLUMN H H 9.** ≥ 100 **YEAR !COLUMN I I**			
	A	B	C	D	E	F	G	H	I
AGE	75 years	80 years	85 years	90 years	95 years	100 years	105 years	110 years	115 years
**DA081** Suppose there are 5 steps, where the lowest step represents the smallest chance and the highest step represents the highest chance, on what step do you think is your chance in reaching the age of [...]? 1 Almost impossible 2 Not very likely 3 Maybe 4 Very likely 5 Almost certain	1 2 3 4 5	1 2 3 4 5	1 2 3 4 5	1 2 3 4 5	1 2 3 4 5	1 2 3 4 5	1 2 3 4 5	1 2 3 4 5	1 2 3 4 5

DB FUNCTIONAL LIMITATIONS AND HELPERS

CAPI IfR is younger than 50(year of birth is after 1963) and If DA001 = 1 or 2 or DA002 = 1 or 2 and DA003 = 2 and DA004 = 2 and DA005 = 2 and DA007 = 2 and DA008 = 2 skip DB001- DB015. 50 (1963) DB001-DB015]

[We need to understand difficulties people may have with various activities because of a health or physical problem. Please tell me whether you have difficulty performing any of the following tasks on a regular basis. Exclude any difficulties that you expect to last less than three months. ( ) .]

DB001 Do you have any difficulty with running or jogging about 1 Km? 1
1. No, I dont have any difficulty → Skip to DB004 DB004 2. I have difficulty but can still do it.
3. Yes, I have difficulty and need help. ,
4. I can not do it.

DB002 Do you have difficulty …Walking 1 km…? 1 1. No, I dont have any difficulty → Skip DB003 DB003 2. I have difficulty but can still do it.
3. Yes, I have difficulty and need help. ,
4. I can not do it.

DB003 Do you have difficulty …Walking 100 metres…? 100 1. No, I dont have any difficulty
2. I have difficulty but can still do it.
3. Yes, I have difficulty and need help. ,
4. I can not do it.

DB004 Do you have difficulty …Getting up from a chair after sitting for a long period…?
1. No, I dont have any difficulty
2. I have difficulty but can still do it.
3. Yes, I have difficulty and need help. ,
4. I can not do it.

DB005 Do you have difficulty …Climbing several flights of stairs without resting…?

1. No, I dont have any difficulty
2. I have difficulty but can still do it.
3. Yes, I have difficulty and need help. ,
4. I can not do it.

DB006 Do you have difficulty ...Stooping, kneeling, or crouching...?

1. No, I dont have any difficulty
2. I have difficulty but can still do it.
3. Yes, I have difficulty and need help. ,
4. I can not do it.

DB007 Do you have difficulty ...Reaching or extending your arms above shoulder level...? (he/she is regarded as not having difficulty only if he/she can extend both of his/her arms, otherwise he/she is regarded as having difficulty.) ()

1. No, I dont have any difficulty
2. I have difficulty but can still do it.
3. Yes, I have difficulty and need help. ,
4. I can not do it.

DB008 Do you have difficulty ...Lifting or carrying weights over 10 jin, like a heavy bag of groceries...? 10 ()

1. No, I dont have any difficulty
2. I have difficulty but can still do it.
3. Yes, I have difficulty and need help. ,
4. I can not do it.

DB009 Do you have difficulty ...Picking up a small coin from a table...?

1. No, I dont have any difficulty
2. I have difficulty but can still do it.
3. Yes, I have difficulty and need help. ,
4. I can not do it.

CAPI IF (DB001 = 1 & DB003 = 1 : : : DB009 = 1), THEN SKIP TO DB016, DB001~ DB009 DB016

Here are a few more everyday acivities. Please tell me if you have any difficulties with these because of a physical, mental, emotional or memory problem. Again, exclude any that you expect to last less than three months.

DB010 Because of health and memory problems, do you have any difficulty with dressing? Dressing includes taking clothes out from a closet, putting them on, buttoning up, and fastening a belt.

1. No, I dont have any difficulty → Skip to DB011 DB011 2. I have difficulty but can still do it.
3. Yes, I have difficulty and need help. ,
4. I can not do it.

DB010_W2 Does anyone ever help you dress? 1. Yes
2. No

DB011 Because of health and memory problems, do you have any difficulty with bathing or showering?

1. No, I dont have any difficulty → Skip to DB012 DB012 2. I have difficulty but can still do it.
3. Yes, I have difficulty and need help. ,
4. I can not do it.

DB011_W2 Does anyone ever help you bathe?

1. Yes
2. No

DB012 Because of health and memory problems, do you have any difficulty with eating, such as cutting up your food? (Definition: By eating, we mean eating food by oneself when it is ready.)

1. No, I dont have any difficulty → Skip to DB013 DB013 2. I have difficulty but can still do it.
3. Yes, I have difficulty and need help. ,
4. I can not do it.

DB012_W2 Does anyone ever help you eat?

1. Yes
2. No

DB013 Do you have any difficulty with getting into or out of bed? 1. No, I dont have any difficulty → Skip to DB014 DB014
2. I have difficulty but can still do it.
3. Yes, I have difficulty and need help. ,
4. I can not do it.

DB013_W2 Does anyone ever help you get in or out of bed? 1. Yes
2. No

DB014 Because of health and memory problems, do you have any difficulties with using the toilet, including getting up and down?

1. No, I dont have any difficulty → Skip to DB022_W2_1 DB022_W2_1 2. I have difficulty but can still do it.
3. Yes, I have difficulty and need help. ,
4. I can not do it.

DB014_W2 Does anyone ever help you use the toilet? 1. Yes
2. No

DB015 Because of health and memory problems, do you have any difficulties with controlling urination and defecation? If you use a catheter (conduit) or a pouch by yourself, then you are not considered to have difficulties. ( )

1. No, I dont have any difficulty
2. I have difficulty but can still do it.
3. Yes, I have difficulty and need help. ,
4. I can not do it.

DB022_W2_1 LOOP BRANCHPOINT: IF THIS IS A CORE INTERVIEW and R DID NOT REPORT THAT RECEIVES HELP WITH LAST SERIES OF ADLs (DB010_W2 and DB011_W2 and DB012_W2 and DB013_W2 and DB014_W2 NOT 1), GO TO DB016

PRODUCERE BEGINNING OF DB022_W2_1 LOOP: QUESTIONS DB022_W2_1 THROUGH DB022_W2_3 ARE REPEATED FOR UP TO SEVEN HELPERS. THESE QUESTIONS ARE BASED ON THE ACTIVITIES WITH WHICH THE R REPORTED RECEIVING HELP IN THE ADL SERIES, DB010_W2 THROUGH DB014_W2 (DB010_W2 or DB011_W2 or DB012_W2 or DB013_W2 or DB014_W2 = 1): [////]

DB022_W2_1 PROCEDURE IF THIS IS THE FIRST TIME THROUGH THE ADL HELPER LOOP: ADL

Who most often helps you with [dressing/bathing/eating/getting (in/out of) bed/using the toilet]? (7)

PROCEDURE IF THIS IS THE SECOND OR LATERTIME THROUGH THE ADL HELPER LOOP: ADL

Who is that?

1. Spouse
2. Ex-spouse /
3. Mother
4. Father
5. Mother-in-law /
6. Father-in-law /
7. Children[preload name] []
8. Sibling
9. Sibling of spouse
10. Brother-in-law, sister-in-law ///
11. Grandson /
12. Granddaughter /
13. Other relative
14. Paid helper(such as nanny) ( )
15. Volunteer or Employee of facility
16. Nursing home
17. Other

NOTE: EMPLOYEE(S) OF FACILITY APPEARS ON LIST ONLY FOR AN R CURRENTLY.LIVING IN A NURSING HOME OR WHO WAS LIVING IN A NURSING HOME OR HOSPICE WHEN S/HE DIED. :

**PROCEDURE** IF helper is grandchild (DB022_W2_1 = 35 or 36)

DB022_W2_2 Which child was the parent of the grandchildren? (choose all that apply)

1-20 Provide the list of children

21 Dead child

**PROCEDURE** IF helper is other (DB022_W2_1 = 41)

DB022_W2_3 Whats that persons relationship with you? _____

**DB022_W2_4 BRANCHPOINT**: IF SEVEN ADL HELPERS HAVE ALREADY BEEN DESCRIBED, GO TO DB016 (OUT OF LOOP)

DB022_W2_4 Does anyone else help you with (this activity/these activities)?

1. Yes Go to DB022_W2_1 loop
2. No

DB016 Because of health and memory problems, do you have any difficulties with doing household chores? (Definition: By doing household chores, we mean house cleaning, doing dishes, making the bed, and arranging the house.) [IWER: If R cannot mop the floor, but can scrub, or R cannot fold heavy bedding, but is able to do light ones, then mark (3). (3)]

1. No, I dont have any difficulty → skip to DB017 DB017 2. I have difficulty but can still do it.
3. Yes, I have difficulty and need help. ,
4. I can not do it.

DB016_W2 Does anyone help you do household chores? 1. Yes
2. No

DB017 Because of health and memory problems, do you have any difficulties with preparing hot meals? (Definition: By preparing hot meals, we mean preparing ingredients, cooking, and serving food.) [IWER: If another person prepares ingredients or if R can cook rice, but is not able to prepare side dishes, then mark (3). (3)]

1. No, I dont have any difficulty → skip to DB018 DB018 2. I have difficulty but can still do it.
3. Yes, I have difficulty and need help. ,
4. I can not do it.

**DB017_W2 BRANCHPOINT**: IF R LIVES IN NURSING HOME, GO TO DB018

DB017_W2 Does anyone help you prepare hot meals? 1. Yes
2. No

DB018 Because of health and memory problems, do you have any difficulties with shopping for groceries? By shopping, we mean deciding what to buy and paying for it.

1. No, I dont have any difficulty → skip to DB035 DB035
2. I have difficulty but can still do it.
3. Yes, I have difficulty and need help. ,

4. I can not do it.

DB018_W2 Does anyone help you shop for groceries?

1. Yes
2. No

DB035 Because of health and memory problems, do you have any difficulties with making phone calls?

1. No, I dont have any difficulty → skip to DB020 DB020 2. I have difficulty but can still do it.
3. Yes, I have difficulty and need help. ,
4. I can not do it.
5. Not relevant to me (no phone) → skip to DB020 DB020

DB035_W2 Does anyone help you make telephone calls?

1. Yes
2. No

DB020 Because of health and memory problems, do you have any difficulties with taking medications? By taking medications, we mean taking the right portion of medication right on time.

1. No, I dont have any difficulty → skip to DB022_W2_5 DB022_W2_5 2. I have difficulty but can still do it.
3. Yes, I have difficulty and need help. ,
4. I can not do it.

DB020_W2 Does anyone help you take medications?

1. Yes
2. No

DB022_W2_5 LOOP BRANCHPOINT: IF THIS IS A CORE INTERVIEW and R DID NOT REPORT THAT SOMEONE HELPED WITH ANY OF THE 4 IADLs (DB016_W2 and DB018_W2 and DB035_W2 and DB020_W2 NOT 1), GO TO DB021

PROCEDURE : BEGINNING OF DB022_W2_5 LOOP: QUESTIONS DB022_W2_5 THROUGH DB022_W2_8 ARE REPEATED FOR UP TO SIX HELPERS. THESE QUESTIONS ARE BASED ON THE ACTIVITIES WITH WHICH THE R REPORTED RECEIVING HELP IN THE IADL SERIES (DB016_W2 or DB017_W2 or DB018_W2 or DB020_W2 = 1): [///]

DB022_W2_5 PROCEDURE IF THIS IS THE FIRST TIME THROUGH THE IADL HELPER LOOP: IADL

Who most often helps you with [do household chroces/preparing hot meals/shopping/making telephone calls/taking medications]? [////] (6)

PROCEDURE IF THIS IS THE SECOND OR LATERTIME THROUGH THE ADL HELPER LOOP: ADL

Who is that?

1. Spouse
2. Ex-spouse /
3. Mother
4. Father
5. Mother-in-law /
6. Father-in-law /
7. Children[preload name] []
8. Sibling
9. Sibling of spouse
10. Brother-in-law, sister-in-law ///
11. Grandson /
12. Granddaughter /
13. Other relative
14. Paid helper(such as nanny) ( )
15. Volunteer or Employee of facility
16. Nursing home
17. Other

NOTE: EMPLOYEE(S) OF FACILITY APPEARS ON LIST ONLY FOR AN R CURRENTLY.LIVING IN A NURSING HOME OR WHO WAS LIVING IN A NURSING HOME OR HOSPICE WHEN S/HE DIED. :

PROCEDURE IF helper is grandchild(DB022_W2_5 = 35 or 36)

DB022_W2_6 Which child was the parent of the grandchildren? (choose all that apply)

1-20 Provide the list of children

21 Dead child

PROCEDURE IF helper is other (DB022_W2_5 = 41)

DB022_W2_7 Whats that persons relationship with you? 1-20 Provide the list of children

21 Dead child

DB022_W2_8 BRANCHPOINT: IF SIX IADL HELPERS HAVE ALREADY BEEN DESCRIBED, GO TO DB021 (OUT OF LOOP)

DB022_W2_8 Does anyone else help you with (this activity/these activities)?
1. Yes Go to DB022_W2_5 loop
2. No

DB021 Do you use the following auxiliary? (Code all that apply) () 1. Walking stick
2. Travel device
3. Manual wheelchair
4. Electric Wheelchair
5. Not any

DB019 Because of health and memory problems, do you have any difficulties with managing your money, such as paying your bills, keeping track of expenses, or managing assets?
1. No, I dont have any difficulty
2. I have difficulty but can still do it.
3. Yes, I have difficulty and need help. ,
4. I can not do it.

DB019_W2 Does anyone help you manage your money? 1. Yes
2. No skip DB022_W2_9 DB022_W2_9

**NOTE**: QUESTIONS DB022_W2_9 THROUGH DB022_W2_12 ARE REPEATED FOR UP TO TWO HELPERS.

DB022_W2_9 **PROCEDURE** IF THIS IS THE FIRST TIME THROUGH THE MANAGE MONEY HELPER LOOP:

Who most often helps you manage your money? (2) **PROCEDURE** IF THIS IS THE SECOND OR LATERTIME THROUGH THE MAN- AGE MONEY HELPER LOOP:

Who is that?

1. Spouse
2. Ex-spouse /
3. Mother
4. Father
5. Mother-in-law /
6. Father-in-law /
7. Children[preload name] []
8. Sibling
9. Sibling of spouse
10. Brother-in-law, sister-in-law ///

11. Grandson /
12. Granddaughter /
13. Other relative
14. Paid helper(such as nanny) （）
15. Volunteer or Employee of facility
16. Nursing home
17. Other

**PROCEDURE** IF helper is grandchild (DB022_W2_9 = 35 or 36)

DB022_W2_10 Which child was the parent of the grandchildren? (choose all that apply)

1-20 Provide the list of children

21 Dead child

**PROCEDURE** IF helper is other(DB022_W2_9 = 41)

DB022_W2_11 Whats that persons relationship with you? / _____

**DB022_W2_12 BRANCHPOINT**: IF MANAGE MONEY HELPERS HAVE ALREADY BEEN DESCRIBED, GO TO DB022_1 (OUT OF LOOP)

DB022_W2_12 Does anyone else help you with (this activity/these activities)?

1. Yes Go to DB022_W2_9 loop
2. No

**PROCEDURE** For 7-30 or 31 chosen inDB022_W2_1, DB022_W2_5 and DB022_W2_9, ask DB022_1. DB022_W2_1, DB022_W2_5DB022_W2_9 7-30 31 （ ）DB022_1

DB022_1 Whether it is the [helpers name chosen from DB022] him/herself taking the time? [DB022]

1. Yes
2. No

**PROCEDURE** For each helper chosen in DB022, ask DB023-DB026 DB022 DB023-DB026

DB023 During the last month, on about how many days did [helpers name chosen from DB022] help you? [DB022] ____1...31 Days

DB024 On the days [helpers name chosen from DB022 helps you, about how many hours per day is that? [DB022] / ____ 1...24Hours [IWER: less than an hour = 1 1]

DB025 Is he/she living in your home? /

1. Yes
2. No

DB026 Is [helpers name chosen from DB022 paid to help you? [DB022]]

1. Yes
2. No

SKIP PATTERN CHECKPOINT: PAID HELPER

**PROCEDURE** IF R PAID FOR HELP IN DB026, SKIP TO DB027. DB026 DB027IF R DID NOT PAY FOR HELP, SKIP TO DB029. DB029

DB027 About how much in total did you pay (including value of the goods you gave them as repayment for their help) for the help during the past month?

____ Yuan

DB028 Who contributed most to paying this cost? Please choose one person who paid the most. (Select from the list displayed by CAPI) (CAPI)

1. Yourself
2. Spouse
3. Mother
4. Father
5. Mother-in-law /
6. Father-in-law /
7. Children
8. Spouse of child
9. Sibling
10. Sibling of spouse
11. Brother-in-law, sister-in-law ///
12. Grandson /
13. Granddaughter /
14. Other relative

15. Other

DB029 Do you use the following auxiliary? (Code all that apply) ( )

1. Walking stick
2. Travel device
3. Manual wheelchair
4. Electric Wheelchair
5. Catheter, urine collection bag
6. Toilet Series
7. None of the above

DB030 Suppose that in the future, you needed help with basic daily activities like eating or dressing. Do you have relatives or friends (besides your spouse/partner) who would be willing and able to help you over a long period of time? ()

1. Yes
2. No → Skip to DB032 DB032

DB031 What is the relationship to you of that person or those persons? (Choose all that apply) / () (Select from the list displayed by CAPI) (CAPI)

1: Spouse // 2: Mother // 3: Father // 4: Mother-in-law /// 5: Father- in-law /// 6 - 16: Children[Preload] []// 31: Spouse of child // 32: Sibling // 33: Sibling of spouse // 34: Brother-in-law, sister-in-law ///// 35: Grandson / // 36: Granddaughter /// 37: Other relative // 38: Hire caregivers (eg.nurse home) () // 39: Other //

DB032 How often did the respondent receive assistance in answering this section? [IWER: If it is answered by a proxy, please record the respondents reaction.]

1. Never
2. A few times
3. Most or all of the time
4. The section was completed by a proxy respondent (the respondent is absent)
 → Skip to DB033 DB033

DB033 What is your relationship to R? [IWER: What is the proxys relationship to R? If unknown, please ask the proxy.]

1. Spouse
2. Mother

3. Father
4. Mother-in-law /
5. Father-in-law
6. Sibling
7. Brother-in-law, sister-in-law /
8. Child
9. Spouse of child
10. Grandchild
11. Other relative
12. Helper or other non-relative

DB034 [IWER: Please record the reason for proxy]
What is the main reason for proxy (the respondent is absent)

1. The respondent has serious physical handicaps 2. The respondent has serious mental handicaps
3. The respondent has rejected this interview.
4. Other____. (**DA034_1**)

DC COGNITION & DEPPRESSION

[IWER: If DB032 = 4, then skip to Section E , health care and insurance. Sections Cc and Cd must not be answered by proxy respondents.] [DB032 = 4 E DCDE .]

DC001 Now Im going to ask several simple questions. Some may be easy and some may be hard to answer. Please try to answer as honestly as you can. Are you ready? Please tell me todays date. (Check all that apply) ()

[IWER: R doesnt have to answer in this order. IfR is an elderly person and marked the date by lunar calendar, that date is correct if it matches with the solar calendar. You can check the accuracy, using the converter.]

1. Year is correct
2. Month is correct
3. Day is correct

DC002 Please tell me the day of the week. Is it Monday, Tuesday, Wednesday, Thursday, Friday, Saturday, or Sunday?

1. Day of week OK/correct

2. Day of week not OK/incorrect

DC003 What is the current season (among Spring, Summer, Fall, or Winter)?

1. Season OK
2. Season not OK

DC004 How would you rate your memory at the present time? Would you say it is excellent, very good, good, fair or poor?

1. Excellent
2. Very good
3. Good
4. Fair
5. Poor

We are going to read a list consisting of 10 words and we would like you to memorize as many as you can. We deliberately made the list long to make it difficult for anyone to memorize all of the words; most people will only remember a few of them. Please listen carefully as we read the list because we cannot repeat it. When we finish reading the list, we will ask you to recall and tell us as many words as you can remember, and they dont have to be in the order that you heard them. Is this explanation clear?

[IWER: Do not allow proxy answers.] []

DC008 [CAPI automatically record the current time: hour and minute. CAPI ___(**DC008_1**) _____(**DC008_2**) （24 ）]

Try to remember the words I just read to you. Ill ask you to recall them later. [IWER: Read once more ifR did not recall any of the words, up to 3 times, and then go on. IfR does not recall any of the words,assure them that it is OK so that R will feel comfortable/at ease.] [1 3]

DC006_1 [IWER: Record the times you read to the respondent] ！

Try to remember the words I just read to you. Ill ask you to recall them later.

The 10 items below refer to how you have felt and behaved during the last week. Choose the appropriate response. 10 12

[Show Card 12]

DC009 I was bothered by things that dont usually bother me.

1. Rarely or none of the time (< 1 day) （< 1 ） 2. Some or a little of the time (1 – 2 days) （1 – 2 ）
3. Occasionally or a moderate amount of the time (3 – 4 days) （3 – 4 ）
4. Most or all of the time (5 – 7 days) (5 – 7)

DC010 I had trouble keeping my mind on what I was doing.

1. Rarely or none of the time (< 1 day) （< 1 ） 2. Some or a little of the time (1 – 2 days) （1 – 2 ）
3. Occasionally or a moderate amount of the time (3 – 4 days) （3 – 4 ）
4. Most or all of the time (5 – 7 days) (5 – 7)

DC011 I felt depressed.

1. Rarely or none of the time (< 1 day) （< 1 ） 2. Some or a little of the time (1 – 2 days) （1 – 2 ）
3. Occasionally or a moderate amount of the time (3 – 4 days) （3 – 4 ）
4. Most or all of the time (5 – 7 days) (5 – 7)

DC012 I felt everything I did was an effort.

1. Rarely or none of the time (< 1 day) （< 1 ） 2. Some or a little of the time (1 – 2 days) （1 – 2 ）
3. Occasionally or a moderate amount of the time (3 – 4 days) （3 – 4 ）
4. Most or all of the time (5 – 7 days) (5 – 7)

DC013 I felt hopeful about the future.

1. Rarely or none of the time (< 1 day) （< 1 ） 2. Some or a little of the time (1 – 2 days) （1 – 2 ）
3. Occasionally or a moderate amount of the time (3 – 4 days) （3 – 4 ）
4. Most or all of the time (5 – 7 days) (5 – 7)

DC014 I felt fearful.

1. Rarely or none of the time (< 1 day) （< 1 ） 2. Some or a little of the time (1 – 2 days) （1 – 2 ）
3. Occasionally or a moderate amount of the time (3 – 4 days) （3 – 4 ）
4. Most or all of the time (5 – 7 days) (5 – 7)

DC015 My sleep was restless.

1. Rarely or none of the time (< 1 day) （< 1 ） 2. Some or a little of the time (1 - 2 days) （1 - 2 ）
3. Occasionally or a moderate amount of the time (3 - 4 days) （3 - 4 ）
4. Most or all of the time (5 - 7 days) (5 - 7)

DC016 I was happy.
1. Rarely or none of the time (< 1 day) （< 1 ） 2. Some or a little of the time (1 - 2 days) （1 - 2 ）
3. Occasionally or a moderate amount of the time (3 - 4 days) （3 - 4 ）
4. Most or all of the time (5 - 7 days) (5 - 7)

DC017 I felt lonely.
1. Rarely or none of the time (< 1 day) （< 1 ） 2. Some or a little of the time (1 - 2 days) （1 - 2 ）
3. Occasionally or a moderate amount of the time (3 - 4 days) （3 - 4 ）
4. Most or all of the time (5 - 7 days) (5 - 7)

DC018 I could not get going.
1. Rarely or none of the time (< 1 day) （< 1 ） 2. Some or a little of the time (1 - 2 days) （1 - 2 ）
3. Occasionally or a moderate amount of the time (3 - 4 days) （3 - 4 ）
4. Most or all of the time (5 - 7 days) (5 - 7)

[IWER: Try to persuade R to answer if R refuses at first. Record the exact number R says.]
[ ]

DC019 Lets try some subtraction of numbers this time. What does 100 minus 7 equal?
100 7 _____

DC020 And 7 from that? 7 _____

DC021 And 7 from that? 7 _____

DC022 And 7 from that? 7 _____

DC023 And 7 from that? 7 _____

DC024 [IWER: Please indicate whether the respondent used paper and pencil or any other aid when completing the number subtraction.] [ ]

1. used aid
2. did not use aid

[Show Card 32]

DC025 Do you see this picture? Please draw that picture on this paper. [IWER: Show the picture of two pentagons overlapped.] []

1. Drew the picture
2. Failed to draw the picture

DC026 [CAPI automatically record the current time: hour and minute. ___(**DC026_1**) _(**DC026_2**) (24)]

DC027 A little while ago, I read you a list of words and you repeated the ones you could remember. Please tell me any of the words that you remember now. [IWER: Answers are displayed only for interviewer. Please do not show the screen to R.] [ ]

DC028 Please think about your life-as-a-whole. How satisfied are you with it? Are you completely satisfied, very satisfied, somewhat satisfied, not very satisfied, or not at all satisfied?

1. Completely satisfied
2. Very satisfied
3. Somewhat satisfied
4. Not very satisfied
5. Not at all satisfied

E HEALTH CARE AND INSURANCE

CARD21 Health Facilities

1. General hospital （）
2. Specialized hospital （）
3. Chinese medicine hospital
4. Community healthcare center
5. Township hospital
6. Health care post
7. Village clinic/ Private clinic /
8. Other

CARD20 : Health Insurance

1. Urban employee medical insurance (yi-bao) （） 2. Urban resident medical insurance
3. New cooperative medical insurance (he-zuo-yi-liao) （ ）
4. Urban and rural resident medical insurance （ ）
5. Government medical insurance (gong-fei)
6. Medical aid
7. Private medical Insurance: Purchased by Rs union : 8. Private medical Insurance: Purchased by Individual : 9. Urban non-employed personss health insurance 10. Other medical insurance (specify) （）
11. No insurance

CARD23 : Health Facilities for Inpatient Care

1. General Hospital （）
2. Specialized hospital （）
3. Chinese Medicine Hospital
4. Community Healthcare Center
5. Township Hospital
6. Health care post
7. Other

PART I MEDICAL INSURANCE

Now we would like to know about health insurance or benefits that you might have.
[Show Card 14]

EA001 Are you the policyholder/primary beneficiary of any of the types of health insurance listed below? (circle all that apply) ( )

1. Urban employee medical insurance (yi-bao) ( ) 2. Urban resident medical insurance
3. New cooperative medical insurance (he-zuo-yi-liao) ( )
4. Urban and rural resident medical insurance ( )
5. Government medical insurance (gong-fei)
6. Medical aid
7. Private medical Insurance: Purchased by Rs union : 8. Private medical Insurance: Purchased by Individual : 9. Urban non-employed personss health insurance 10. Other medical insurance (specify) ____ (**EA001_1**)
11. No insurance Skip to EA009 EA009

[Soft check: If pick 11, cannot pick any other, you chose no insurance and a specific type of insurance, this is not possible 11]

F1: (1)

(2) 2007 7 79

()

(3) (4)

(5)

(6)

(7)

(8)

(9)

(10)

(11)

（12）， 16 60 16 50 ， 700 600 100

For each circled type of insurance (1-10), ask the following questions EA002 - EA008. 1-10 EA002 - EA008

**PROCEDURE** IF EA001 = 7 or 8, skip EA002

EA002 Do you have supplemental insurance to this plan? （ ）

1. Yes
2. No

F1

EA003 Where did you set up your insurance account/policy? ? 1. This county /

2. (if it is not in this county) the place of your HuKou （/）
3. Other _______(**EA003_ 1**)province _________(**EA003_2**)county /

EA004 Method of reimbursement

1. Get reimbursement immediately
2. You pay first and get reimbursed later.

EA005 Through which agency did you purchase your primary plan?

1. Community committee/ village committee /
2. Rs union
3. Agency of Social insurance
4. Private insurance company
5. Other

EA006 Whats your out-of-pocket yearly premium? ( )

____Yuan [soft check upper limit: 15,000 for choice 1, 3,000 choice 2, 1,000 choice 3, 1,500 choice 4, 4,000 choice 7, 2,500 choice 9 1 15,000 23,000 3 1,000 4 1,500 74,000 92,500]

EA007 Who pay the premium for you? (choose all that apply) ()

1. Myself
2. Children
3. Relatives
4. Government
5. Rs union ( )
6. Loan
7. Donate
8. Others Specify (**EA007_1**)

EA008 When did this benefit begin? [IWER: Mark the year using four digits. Take down the month as its actual number. For example, write January as 1 not 01, December as 12. If do not remember month, fill '0. : 4 1 10112 12 '0]

1900...2013Year (**EA008_1**)____ 0...12Month (**EA008_2**)_____

**PROCEDURE** Skip to EC001 for new R EC001

**PROCEDURE** For XRType = REIW who had health insurance in last wave

EA010[i]_W2 Our records from your last interview in Rs LAST IW MONTH, YEAR show that you have had [preload health insurance], is this right? [] []

1. Yes
2. No

**PROCEDURE** IF change to another health insurance program in this wave : IF DEA010_W2 = 1, ask EB003,EB004 EB003,EB004IF DEA010_W2 = 2, ask EB002 - EB004 EB002 - EB004

**PROCEDURE** IF uninsured this wave : IF DEA010_W2 = 1, ask EA009,EB003,EB004 EA009,EB003,EB004 IF DEA010_W2 = 2, ask EA009,EB002 - EB004 EA009,EB001 - EB004

EA009 What is your main reason for not having health insurance?

1. I do not need it
2. Cannot afford it
3. Do not know where or from whom to get it

4. Do not trust the institutions that offer health insurance 5. Do not have suitable programs for me to buy

6. Do not know/never thought of it

7. Others

EB001 Did you have health insurance at any time? 1. Yes

2. No → Skip to EC001 EC001

F1:

[Show Card 14]

EB002 What type of insurance did you have ( ) 1. Urban employee medical insurance (yi-bao) ( )

2. Urban resident medical insurance

3. New cooperative medical insurance (he-zuo-yi-liao) ( )

4. Urban and rural resident medical insurance ( )

5. Government medical insurance (gong-fei)

6. Medical aid

7. Private medical Insurance: Purchased by Rs union : 8. Private medical Insurance: Purchased by Individual : 9. Urban non-employed personss health insurance 10. Other medical insurance (specify) _____

[Soft check: If pick 11, cannot pick any other, you chose no insurance and a specific type of insurance, this is not possible 11]

F1 (1)

(2) 2007 7 79

( )

(3) (4)

(5)

(6)

(7)

(8)

(9)

(10)

(11) (12) , 16 60 16 50 , 700 600 100

**PROCEDURE** For each circled type of insurance (1-10), ask EA008_W2_1 and EB003 1-10 EA008_W2_1 and EB003

EA008_W2_1 When did this benefit begin? [IWER: Mark the year using four digits. Take down the month as its actual number. For example, write January as 1 not 01, December as 12. If do not remember month, fill ‘0. : 4 1 101,12 120]

1900: : :2013 Year (**EA008_W2_1_1**)____0: : :12 Month (**EA008_W2_1_2**)_____

EB003 When did you lose it? [IWER: Mark the year using four digits. Take down the month as its actual number. For example, write January as 1 not 01, December as 12. If do not remember month, fill ‘0. : 4 1 101,12 12 0]

1900: : :2013 Year (**EB003_1**)____0: : :12Month (**EB003_2**)_____

EB004 Why did you lose it?

1. Employer no longer exists.
2. Such insurance will not be provided locally anymore. 3. I resigned/was fired.
/
4. Other(please specify_____ ____(**EB004_1**)

PART II HEALTH CARE COSTS AND UTILIZATION

[IWER: Please do not allow proxy to answer Part II.]

EC001 **PROCEDURE** For XRType = NEWIW

When did you take the last physical examination? **PROCEDURE** For XRType = REIW

When did you take the last physical examination since Rs LAST IW MONTH, YEAR/in the last two years?

[IWER: Mark the year using four digits. Take down the month as its actual number. For example, write January as 1 not 01, December as 12. If do not remember month, fill ‘0. : 4 1 1 01,12 12 ‘0]

1. 1900...2013 Year (**EC001_1**)____0...12 Month (**EC001_2**)____ 2. Didnt ever take physical examination yet
3. Didnt take physical examination last two years

EC002 Who paid the physical examination cost?

1. Myself
2. Children
3. Relatives
4. Government
5. Rs union ()
6. Rs insurance
7. Loan
8. Donate
9. Others ____ Specify ____(**EC002_1**)

The next questions pertain to medical facilities or medical providers you may have visited for outpatient care during the past 1 month (excluding hospitalization). ()

ED001 In the last month have you visited a public hospital, private hospital, public health center, clinic, or health workers or doctors practice, or been visited by a health worker or doctor for outpatient care? ()

1. Yes Skip to ED004ED004
2. No

ED002 Have you been ill in the last month?

1. Yes
2. No

PROCEDURE
If ED001 = 2 and ED002 = 2 skip to EE001 ED001 = 2 ED002 = 2 EE001
If ED001 = 1 skip to ED004 ED001 = 1 ED004
If ED002 = 1 and ED001 = 2 go to ED003. ED002 = 1 ED001 = 2 ED003

ED003 Whats the main reason for not seeking medical treatment?

1. Already under treatment.
2. Illness is not serious. Dont need treatment 3. Poor
4. No time
5. Inconvenient traffic
6. Poor service
7. No available treatment
8. Other

[Show Card 15]

ED004 Which types of medical facilities have you visited in the last 4 weeks for outpatient treatment? (circle all that apply) ( )

1. General hospital ( )
2. Specialized hospital ( )
3. Chinese medicine hospital
4. Community healthcare center
5. Township hospital
6. Health care post
7. Village clinic/ Private clinic /
8. Other

**PROCEDURE** For each item 1-7 checked in ED004, ask ED005ED004 1-7 ED005

ED005 How many times did you visit/been visited by [...] during the last month? ____ Times

**PROCEDURE** If sum(ED005)> 1, then ask ED006; otherwise, skip ED006ED005 > 1, ED006ED006

ED006 How much did all the visits to [ED004 answer] cost during the last month? [ED004 answer] ? [IWER: If possible, please check the list of cost.]

1. Total cost ____(ED006_ 1) Yuan ; [soft check upper bound: 30,000 30,000. Brackets50/100/200/500/1000]
2. Didnt pay anything

**PROCEDURE** IF ED006 = 1, ASK ED007

ED007 1. Self-paid part ____ (ED007_ 1)Yuan [Brackets50/100/200/500/1000] 2. Didnt pay anything.

Now Id like to ask you some questions about your most recent visit to a health care provider in the last month.

ED008 Which health care provider did you visit most recently during the past month? [CAPIPreload the health care providers in ED004. ED004]

ED009 Is this facility public or private?

1. Public
2. Private

**PROCEDURE** If ED008 = 1 – 3, ask ED010 ED008 = 1 – 3 ED010

ED010 Whats the level of this facility?

1. County/district //
2. Regional /city /
3. Provincial/ affiliated to a ministry /
4. Military
5. Others
6. Not applicable

ED012 Did the provider visit you at home?

1. Yes skip ED013 to ED016 and ED022 ED013 ED016 ED022 2. No

ED014 What is the travel time (one-way) to that facility? Minutes ____(**ED014_1**)

How to go to the facility? (**ED014_2**)

1. Walk
2. Bus
3. Car

4. Ambulance
5. Bicycle or other manual vehicles
6. Electric bicycle/electric tricycle /
7. Motorcycle
8. Tractor
9. Train
10. Animal or animal-pulled cart

**PROCEDURE** If ED014 = 1, skip ED015 ED014 = 1 ED015

ED015 What was the total transportation cost to the facility (including fuel cost, one way trip)? ( ) ____ RMB [softcheck upper limit: 600 600]

ED017 What was the purpose of your visit? (circle all that apply) ()

1. Immunization
2. Consultation
3. Medical check-up
4. Treatment for Illness
5. Other

**PROCEDURE** IfED017 = 4, then ask ED018 —ED021, ELSE ASK ED023. ED017 4ED018 — ED021 ED023

ED018 Could you tell me the disease name? _____

ED019 Was the visit a first visit or a follow-up visit for the symptom?

1. First
2. Follow-up

ED020 Was the visit for ordinary outpatient service or an emergency?

1.
2.

[Show Card 16]

ED021 What kind of treatment did you receive? (circle all that apply) ()

1. Injection
2. Laboratorium test
3. Surgery

4. X-ray, CT, B ultrasonic, MRI X- CTB
5. Medications and purchase medical
6. IV (Drip Infusion)
7. Traditional treatment, eg massage, acupuncture 8. Other

ED023 What was the total cost of this visit, including both treatment and medication cost (includes prescriptions you received)? ( )

1. _____ RMB (**ED023_1**) [softcheck upper limit: 30,000]
[Brackets 25/50/120/400/1200]
2. There was no cost

**PROCEDURE** IF ED023 = 1, ASK ED024

ED024 How much did you pay out of pocket, after reimbursement from insurance?

1. ____RMB (**ED024_1**) [soft check upper limit: 30,000, also ED025 <= ED023_1, else Pay out of pocket cannot be more than total cost 30,000ED025 <= ED023_1]
2. Did not pay anything Go to ED026 ED026
[Brackets 15/10/100/300/1000]

ED025 Who contribute most for paying the out-of-pocket cost? 1. Myself

2. Children
3. Relatives
4. Government
5. Rs union ( )
6. Loan
7. Donate
8. Others Specify

ED026 What was the total medication cost for this visit, including prescriptions you received? ()

1. ____ RMB (**ED026_1**) [soft check upper limit: 5,000, and must be no more than ED023_1. 5,000ED023_1] [Brackets 10/30/80/250/600]
2. Doctor did not write a prescription skip ED027 ED027 3. Didnt fill prescription skip ED027 ED027

ED027 How much will you eventually pay out of pocket for the medications from this visit, including prescriptions you received? ( )

1. ____RMB (**ED027_1**) [softcheck upper limit: 5,000 and ED027_1 must be no more than ED026_1 and no more than ED023_1. 5,000 ED027_1 ED026_1ED023_1] [Brackets 10/20/70/200/500]
2. Didnt pay anything

[Show Card 14]

ED028 What insurance did you use or will you use?(circle all that apply) ( ) ()

1. Urban employee medical insurance (yi-bao) () 2. Urban resident medical insurance
3. New cooperative medical insurance (he-zuo-yi-liao) ( )
4. Urban and rural resident medical insurance ( )
5. Government medical insurance (gong-fei)
6. Medical aid
7. Private medical Insurance: Purchased by Rs union : 8. Private medical Insurance: Purchased by Individual : 9. Urban non-employed personss health insurance 10. Other medical insurance (specify) , _____
11. Reimbursed by Rs union
12. No insurance
13. Not revelent to me .

F1 (1)

(2) 2007 7 79

()

(3) (4)

(5)

(6)

(7)

(8)

(9)

(10)

(11) (12)

(13) , 16 60 16 50 , 700 600 100

ED029 Did you give any red envelopesto the doctors for this visit?

1. Yes
2. No

The following questions pertain to hospitalization (inpatient care) that you have had during the past year.

EE001 In the past year, did a doctor suggest that you needed inpatient care but you did not get hospitalized?

1. Yes
2. No Skip to EE003 EE003

EE002 Whats the main reason for not seeking hospitalization?

1. Not enough money
2. Not willing to go to the hospital
3. Felt that hospital was unlikely to cure problem—hospital quality poor
4. Felt that care was unlikely to cure the problem—problem too serious
5. No ward available
6. Other

EE003 Have you received inpatient care in the past year? 1. Yes

2. No Skip to EF001 EF001

EE004 How many times have you received inpatient care during the past year? ____Times

**PROCEDURE** If EE004 = 1, skip to EE007. EE004 = 1EE007

EE005 What was the medical cost for all the hospitalizations you received during the past year? (Only include fees paid to the hospital, including ward fees but excluding wages paid to a hired nurse, transportation costs, and accommodation costs for yourself or family members.) ? ( )

1. Total cost ____(**EE005_1**)Yuan ; [Brackets 1500/3000/7000/15000/30000]

2. Didnt pay anything. [softcheck upper limit : 300,000]

**PROCEDURE** IF EE005 = 1 ASK EE006.

EE006

1. Self-paid part ____(**EE006_1**)Yuan
2. Didnt pay anything.

[softcheck upper limit: 300,000 and EE005 B.1<=EE005 A.1. 300,000 EE005 B.1<=EE005 A.1.] [Brackets 600/1500/4000/8000/18000]

We want details about the last hospitalization you had in the past year.

**PROCEDURE** If ED001 = 1 ask EE007. ED001 = 1 EE007

EE007 Is this the same facility as mentioned in ED008 for outpatient care? [ED008]

1. Yes Skip to EE016 EE016
2. No

**PROCEDURE** If EE007 = 2 or ED001 = 2 ask EE008 -EE015. EE007 = 2 ED001 = 2 EE008-EE015

[Show Card 17]

EE008 What is the type of health or service facility which you visited for last inpatient care (hospital admissions)/for your most recent hospitalization in the past year?

1. General Hospital ( )
2. Specialized hospital ( )
3. Chinese Medicine Hospital
4. Community Healthcare Center
5. Township Hospital
6. Health care post
7. Other

EE009 Is this facility public or private?

1. Public
2. Private

**PROCEDURE** If EE008 = 1 - 3, ask EE010, EE008 = 1 - 3EE010

EE010 Whats the level of this facility?

1. County/district //
2. Regional /city /
3. Provincial/ affiliated to a ministry /
4. Military
5. Others

EE012 What is the location of this facility? 1. province (**EE012_1**)

A. This province

B. Other province, (preload province) ( ) (**EE012_1_1**) [IWER: Choose from the list of provincessee appendix 2]

2. county/city / (**EE012_2_1**)

A. This county / city /

B. Other county / city, specify // (**EE012_2_2**)

3. township/district / (**EE012_3_1**)

A. This township/district /

B. Other township/district, specify / (**EE012_3_2**) /

4. village/street / (**EE012_1_1**)

A. This village/street /

B. Other village/street, specify / (**EE012_4_2**)

EE013 How many kilometers is it from the medical facility to your residence? ____ Km [softcheck upper limit : 3000]

EE014 What is the travel time (one-way) to that facility?

Unit (**EE014_1**)

1. _____Minute [check range : 1-59]
2. ____Hour [softcheck upper limit : 20]

How to go to the facility? (**EE014_2**)

1. Walk
2. Bus
3. Car
4. Ambulance
5. Bicycle or other manual vehicles
6. Electric bicycle/electric tricycle /

7. Motorcycle
8. Tractor
9. Train
10. Animal or animal pulled cart

**PROCEDURE** IF EE014_2 = 2 - 10, ask EE015

EE015 What was the total transportation cost to the facility (including fuel cost, one way trip)? () ____RMB [soft check upper limit : 600]

EE016 How many nights were you hospitalized there? ____Nights [softcheck upper limit : 40]

EE017 What was the starting date of your hospital stay?
____1900...2013 (**EE017_1**)Year ____0...12 (**EE017_2**)Month ____0...31 (**EE017_3**)Day [IWER: Mark the year using four digits. Take down the month as its actual number. For example, write January as 1 not 01, December as 12. If do not remember month and day, fill '0. : 4 1 10112 12 '0]

EE018 What was your date of exit?
1. ____1900...2011 (**EE018_1**) Year ____0...12 (**EE018_2**) Month ____0...31 (**EE018_3**) Day
2. Still there

[soft check: date of exit should be not before starting date, exit date is before starting date, please ask R again, also exit date should be within 1 year of today]

EE019 Why were you hospitalized? (Choose one choice) () 1. Sickness
2. Accident
3. Violence
4. Other

EE020 Could you tell me the name of the disease?

____

[Show Card 18]

EE021 During hospitalization, what kind of treatment did you receive? (circle all that apply) () [IWER: Read one by one.]

1. Medical check-up/consultation
2. Injection
3. Laboratory test
4. Surgery
5. X-ray, CT, B ultrasonic, MRI X- CTB
6. Medications
7. IV (Drip Infusion)
8. Traditional treatment, e.g., massage, acupuncture 9. Delivery
10. Other

**PROCEDURE** IF EE018 = 2, please skip EE022. EE018 = 2EE022

EE022 Under what conditions did you leave the hospital?

1. Fully recovered from illness, received doctors approval Skip EE023 EE023
2. Didnt recover from illness, but received doctors suggestion to leave Skip EE023 EE023
3. Didnt recover from illness, requested to leave without doctors suggestion
4. Other reasons Skip EE023 EE023

EE023 Why did you want to leave the hospital before you were recovered?

1. Cant recover from illness
2. Poor
3. No space in the hospital
4. Limited hospital conditions
5. Poor quality and service from health care providers 6. Other reasons

EE024 What was the total medical cost of hospitalization? (Only include the fees paid to the hospital, excluding the wage of hired nurse, the fare or rent, but including the ward fees.) ( )

1. ____RMB (**EE024_1**)[softcheck upper limit : 100,000] [Brackets 700/1500/3500/8000/15000]
2. There was no cost

EE025 What was the total cost for hired nurse? ?1. ____RMB (**EE025_1**)

2. There was no cost

EE026 What was the total cost for transportation, food and accommodation of patient and relatives? ?

1. ____RMB (**EE026_1**)
2. There was no cost

EE027 How much did you or will you eventually pay out of pocket for the total costs of hospitalization? ( )

1. ____RMB (**EE027_1**) [softcheck upper limit: 100,000 and EE027_1 must be no more than EE024_1 100,000EE027_1 EE024_1]
2. Didnt pay anything. Skip EE028 EE028

EE028 Who contributes most for paying the out-of- pocket cost? 1. Myself

2. Children
3. Relatives
4. Government
5. Rs union ( )
6. Loan
7. Donate
8. Others Specify

EE029 What was the total medication cost during this visit? 1. ____RMB (**EE029_1**) [softcheck upper limit: 60,000 and EE029_1 should be no more than EE024_1 60,000EE029_1 EE024_1] 2. Didnt receive Skip EE030 EE030

[Brackets 200/500/1800/4000/8000]

EE030 How much did you pay out of pocket for medication costs during this visit?

1. ____RMB (**EE030_1**) [softcheck upper limit: 60,000 and EE030_1 should be no more than EE029_1 and EE024_1 60,000 EE030_1 EE029_1 EE024_1] [Brackets 100/300/1000/2500/5000]
2. Didnt pay anything.

[Show Card 14]

EE031 (Preload from EA001 or EB002) What insurance did you use or will you use? (circle all that apply) ( )

1. Urban employee medical insurance (yi-bao) ( ) 2. Urban resident medical insurance

3. New cooperative medical insurance (he-zuo-yi-liao) ( )

4. Urban and rural resident medical insurance （ ）

5. Government medical insurance (gong-fei)

6. Medical aid

7. Private medical Insurance: Purchased by Rs union : 8. Private medical Insurance: Purchased by Individual : 9. Urban non-employed personss health insurance 10. Other medical insurance (specify) , _____

11. Reimbursed by Rs union

12. No insurance

13. Not revelent to me

F1（1）

（2） 2007 7 79

（）

（3） （4）

（5）

（6）

（7）

（8）

（9）

（10）

（11） （12）

（13）， 16 60 16 50 ， 700 600 100

EE032 Did you pay any Red Envelopesto the doctors for this visit?

1. Yes
2. No

Now wed like to know whether you have treated yourself during the past month.
[Show Card 19]

EF001 How did you treat yourself during the past month? (circle all that apply) （ ）（ ） [check, if choose 7 cannot choose other options 7]

1. Consumed over-the-counter modern medicines 2. Consumed prescription medicines
3. Consumed traditional herbs or traditional medicines as treatment
4. Tonic/ Health supplement //
5. Use health care equipment
6. Other
7. None Skip to EH001 EH001

**PROCEDURE** For each circled self-treatment method (1-6), ask questions EF002, EF003, EF005. 1-6 EF002EF003EF005

EF002 What is the approximate total cost to [preload EF001] during the last month? [preload EF001]

1. _____RMB (**EF002_1**) [softcheck upper limits: 2,000 2,000] [Brackets 10/30/100/200/300]
2. There was no cost.

**PROCEDURE** IF EF002 = 1 ASK EF003. EF002 = 1EF003

EF003 How much did you pay out-of-pocket?

1. ____RMB (**EF003_1**) [softchecks upper limits: 2,000 and EF003_1 should be no more than EF002_1. 2,000 EF003_1 EF002_1] [Brackets 10/30/100/200/300]
2. Didnt pay anything. Go to EF005 EF005

EF004 Who contribute most for paying the out-of-pocket cost? 1. Myself

2. Children
3. Relatives
4. Government

5. Rs union （）
6. Loan
7. Donate
8. Others Specify

[Show Card 14]

EF005 What insurance did you use? (circle all that apply) （ ）

1. Urban employee medical insurance (yi-bao) （） 2. Urban resident medical insurance
3. New cooperative medical insurance (he-zuo-yi-liao) （ ）
4. Urban and rural resident medical insurance （ ）
5. Government medical insurance (gong-fei)
6. Medical aid
7. Private medical Insurance: Purchased by Rs union : 8. Private medical Insurance: Purchased by Individual : 9. Urban non-employed personss health insurance 10. Other medical insurance (specify) _____
11. Reimbursed by Rs union
12. No insurance
13. Not revelent to me

F1（1）
（2） 2007 7 79
（）
（3） （4）
（5）
（6）
（7）
（8）

(9)
(10)
(11) (12)
(13), 16 60 16 50, 700 600 100

The following questions pertain to dental care that you have had during the past year.

EH001 In the past year, have you seen a dentist for dental care, including dentures?
1. Yes
2. No skip this part

EH002 How many times have you received dental care during the past year? ____Times

EH003 What was the medical cost for all the dental care in the past year? ?
1. Total cost ____Yuan;
2. Didnt pay anything. [softcheck upper limit: 300,000]

**PROCEDURE** IF EH003 = 1 ASK EH004

EH004
1. Self-paid part _______(**EG004_1**)Yuan
2. Didnt pay anything

**PROCEDURE** IF EH004 = Didnt pay anything, skip EH005

EH005 Who contribute most for paying the out-of-pocket cost? 1. Myself
2. Children
3. Relatives
4. Government
5. Rs union ()

6. Loan
7. Donate
8. Others Specify

[Show Card 14]

EH006 What insurance did you use? (circle all that apply) （ ）

1. Urban employee medical insurance (yi-bao) （） 2. Urban resident medical insurance

3. New cooperative medical insurance (he-zuo-yi-liao) （ ）

4. Urban and rural resident medical insurance （ ）

5. Government medical insurance (gong-fei)

6. Medical aid

7. Private medical Insurance: Purchased by Rs union : 8. Private medical Insurance: Purchased by Individual : 9. Urban non-employed personss health insurance 10. Other medical insurance (specify) , _____

11. Reimbursed by Rs union
12. No insurance
13. Not revelent to me.

F1（1）
（2） 2007 7 79
（）
（3） （4）
（5）
（6）
（7）
（8）
（9）

(10)

(11) (12)

(13), 16 60 16 50, 700 600 100

EF006 How often did the respondent receive assistance in answering section D-Health care and insurance? [IWER: If it is answered by a proxy, the respondents reaction.]

1. Never
2. A few times
3. Most or all of the time
4. The section was completed by a proxy respondent (the respondent is absent)
   → Skip to EF007 EF007

EF007 What is your relationship to R? [IWER: What is the proxys relationship to R? If unknown, please ask the proxy.]

1. Spouse
2. Mother
3. Father
4. Mother-in-law /
5. Father-in-law /
6. Sibling
7. Brother-in-law, sister-in-law /
8. Child
9. Spouse of child
10. Grandchild
11. Other relative
12. Helper or other non-relative

EF008 [IWER: Please record the reason for proxy] What is the main reason for proxy (the respondent is absent)

1. The respondent has serious physical handicaps

2. The respondent has serious mental handicaps, 3. The respondent has rejected this interview

4. Other

F WORK, RETIREMENT AND PENSION

Type of Interview R	
XRType = REIW	This is a reinterview R
XRType = NEWIW	This is a new interview R

PRELOAD VARIABLE ACCORDING TO LAST WAVE INTERVIEW

**ZF1**= 1	Old R was only doing agricultural work in the last IW
**ZF1**= 2	Old R was only doing non-agricultural work in the last IW
**ZF1**= 3	Old R was doing both agricultural and non-agricultural work in the last IW
**ZF1**= 4	Old R was not working in the last IW
**ZF5**= 1	Old R was doing employed work in the last IW
**ZF6**= 1	Old R was doing self-employed work in the last IW
**ZF7**= 1	Old R was doing unpaid family business in the last IW
**ZF8**	The name of old Rs employer in the last IW
**ZF8_1**	The information of the type of business in the last IW
**ZF9**	The name of old Rs company or business in the last IW
**ZF9_1**	The name of old Rs company or business is same with the last IW
**ZF10**	The name of company or workplace old R worked without wage in the last IW
**ZF10_1**	The name of company or workplace old R worked without wage is the same with last IW
**ZF12**= 1	Old R answered work history question in the last IW
**ZF13**= 1	Old R was working in the last IW

ZF14= 1 Old R had not worked prior to the last IW

ZF14= 2 Old R had worked prior to the last IW

ZF15= 1 Old R had completed retirement procedure (including early retirement) or internal retirement in the last IW.
( )

ZF16= 1 Old R had completed receding position procedure in the last IW

ZF17= 1 The information of the kind of business in the last IW

ZF18 Old Rs position in employed work in the last IW

ZF19 Old Rs proffessional/technical level in employed work in the last IW
/

ZF19_1 Old Rs proffessional/technical level in the same employed work in the last IW
/ ( )

ZF22 The information of old R worked without wage in the last IW

ZF22_1= 1 Old R s last job is not missing, FL001, FL004 and FL005 are not missing
（FL001 FL004FL005）

ZF22_1= 2 Old R s last job is missing, FL001, FL004 or FL005 are missing
（FL001, FL004 and FL005）

ZF23= 1 Old R had completed internal retirement but not yet regular retirement prior to the last IW.

ZF24= 1 Old R had not completed retirment procedure or receding position procedure prior to the last IW.

ZF25 Old Rs last job name

ZF25_1= 1 Old R had completed retirment or receding position procedure in the last IW, but the work unit is missing.
/

ZF25_2= 1 Old R had been receding position procedure in the last IW, but the time is missing.

ZF25_3= 1 Old R had been receding position procedure in the last IW, but the wage is missing.

ZF25_4= 1 Old R had completed retirement or early retirement.
[/]

ZF25_5= 1 Old R had completed retirement or early retirement, but the time is missing.
[/]

ZF25_6= 1 Old R had completed retirement or early retirement, but the wage is missing.
[/]

ZF25_8= 1 Old R had completed internal retirement.

ZF25_9= 1 Old R had completed internal retirement, but the time is missing.

ZF25_ 10= 1 Old R had completed internal retirement, but the wage prior internal retirement is missing.

ZF25_ 11= 1 Old R had completed internal retirement, but the wage at internal retirement is missing.

ZF26= 1 Old R had competed regular retirment in the last IW

ZF27= 1 Old R had not worked after processing retirement procedure or receding position procedure by the last IW.
/

XF1= 1 Old R is working in the current IW

XF1= 2 Old R is not working in the current IW

XF2= 1 R (including new R and old R) did not do faming last year but did non-agricultural work last week. （）

FA JOB STATUS

FA001 Did you engage in agricultural work (including farming, forestry, fishing, and husbandry for your own family or others) for more than 10 days in the past year? 10 （ ）

1. Yes → NEWIW skips to FB001; REIW skips to FA006_w2_1 BRANCHPOINT FB001FA006_w2_1 BRANCHPOINT
2. No

FA002 Did you work for at least one hour last week? We consider any of the following activities to be work: earn a wage, run your own business and unpaid family business work, et. al. Work does not include doing your own housework or doing activities without pay, such as voluntary work.

1. Yes → NEWIW skips to FB001; REIW skips to FA006_w2_1 BRANCHPOINT FB001; FA006_w2_1 BRANCHPOINT
2. No

FA003 Do you have a job but are temporarily laid-off, or on sick or other leave, or in-job training?

1. Yes
2. No → NEWIW skips to FA007; REIW skips to FA006_w2_1 BRANCHPOINT FA007; FA006_w2_1 BRANCHPOINT

FA004 In what month and year did you leave or attend training?

____ 1900...2013 (**FA004_1**) year __0...12 (**FA004_2**) month

[IWERMark the year using four digits. Take down the month as its actual number. For example, write January as 1 not 01, December as 12. If do not remember month, fill '0. : 4 1 1 01,12 12**O**]

FA005 Do you expect to go back to this job at a definite time in the future or within 6 months? 6

1. Yes → NEWIW skips to FB001; REIW skips to FA006_w2_1 BRANCHPOINT FB001; FA006_w2_1 BRANCHPOINT
2. No

FA006 Do you still receive any salary from this job? ?

1. Yes → NEWIW skips to FB001; REIW skips to FA006_w2_1 BRANCHPOINT FB001; FA006_w2_1 BRANCHPOINT
2. No → NEWIW skips to FA007; REIW skips to FA006_w2_1 BRANCHPOINT FA007; FA006_w2_1 BRANCHPOINT

FA006_w2_1 BRANCHPOINT:

For REIW doing agricultuarl work (FA001= 1): If he/she was only doing agricultural work in the last IW (ZF1= 1), SKIP TO FA006_w2_5; If he/she was only doing non-agricultural work (ZF1= 2) or not doing any work in the last IW (ZF1= 4), SKIP TO FA006_w2_2; If he/she was doing both agricultural and non-agricultural work in the last IW(ZF1= 3), skip to FA006_w2_2a Branchpoint.

For REIW not doing agricultural work but doing non-agricultural work (FA001= 2 & FA002= 1): If he/she was only doing agricultural work in the last IW (ZF1= 1) or doing both agricultural and non-agricultural work (ZF1= 3) in the last IW, skip to FA006_w2_1; If he/she was only doing non-agricultural work in the last IW (ZF1= 2), skip to FA006_w2_2a Branchpoint; If he/she was not doing any work in the last IW (ZF1= 4), SKIP TO FA006_w2_5;

For REIW not doing any work(ZF1= 2): If he/she was only doing agricultural work in the last IW (ZF1= 1), or doing both agricultural and non-agricultural work in the last IW (ZF1= 3), skip to FA006_w2_1; If he/she was only doing non-agricultural work in the last IW (ZF1= 2), skip to FA006_w2_2a Branchpoint; If he/she was not doing any work in the last IW (ZF1= 4), skip to FA006_w2_5.

FA006_w2_1 You told us that you were doing agricultural work (ZF1= 1 or ZF1= 3) in the last IW, when did you stop doing it? [ZIWTime] （ZF1= 1 ZF1= 3）

1. ____ (**FA006_w2_1_1**) year ____ (**FA006_w2_1_2**) month
2. I was not doing agricultural work in the last IW → Skip to FA006_w2_1a Branchpoint FA006_w2_1a Branchpoint

FA006_w2_1a What was the reason for you to stop doing this job?

1. Business closed /
2. Quit
3. I was laid off
4. I was fired
5. I went to school
6. I went abroad
7. I stopped working for health reasons
8. I stopped working for family reasons
9. I was transferred to another job
10. I started working off-farm locally
11. I went to work away from home
12. Better job in local area
13. Better job in another location
14. I retired
15. Other

FA006_w2_1a BRANCHPOINT:

IF XRType = REIWAND HE/SHE was only doing agricultural work IN THE LAST WAVE, THEN SKIP TO FA006_w2_5.

IF XRType = REIW AND HE/SHE was doing both agricultural and non-agricultural work IN THE LAST WAVE, THEN SKIP TO FA006_w2_2a Branchpoint.

FA006_w2_2 You told us that you were not doing agricultural work in the last IW (ZF1= 2 or ZF1= 4), when did you starting doing agricultural work? [ZIWTime] （ZF1= 2 ZF1= 4）

1. ____ (**FA006_w2_2_1**) year ____ (**FA006_w2_2_2**) month
2. I was doing agriclutural work in the last IW

PROCEDURE :

If XRType = REIW and he/she was only doing non-agricultural work in the last IW (ZF1= 2), skip to FA006_w2_2a Branchpoint;

If XRType = REIW and he/she was not doing any work in the last IW (ZF1= 4), skip to FA006_w2_5;

FA006_w2_2a BRANCHPOINT:

For old R who was doing employed work in the last IW (ZF5= 1): If the name of the employer is not missing in the last IW (ZF8≠ :), skip to FA006_w2_2a; If the name of the employer is missing in the last IW (ZF8= :), skip to FA006_w2_2a_2.

For old R who was doing self-employed work in the last IW (ZF6= 1): If the name of the company/business is not missing in the last IW (ZF9≠ :), skip to FA006_w2_2b; If the name of the company/business is missing in the last IW(ZF9= :), skip to FA006_w2_2b_2.

For old R who was working for unpaid family business (ZF7= 1): If the name of the family business is not missing in the last IW (ZF10≠ :), skip to FA006_w2_2c. If the name of the family business is missing in the last IW ZF10= :),skip to FA006_w2_2c_2.

FA006_w2_2a You told us that you were working for[Preload the name of the employer from the last IW (ZF8 in the last IW) , is the name correct? [ZIWTime] [(ZF8)]

1. Yes → Skip to FA006_w2_2a_3 FA006_w2_2a_3
2. No

[( ) (2)]

FA006_w2_2a_1 What is the correct name?

[IWER: Write the name of the household head if R works for a family.]

1. Fill in the employers name __________(**FA006_w2_2a_1_1**) (1 2) Name of the department __________ (1 2) → Skip to FA006_w2_2a_3 FA006_w2_2a_3
2. Last IW, you ran your own business, fill in the firms name / / __________(**FA006_w2_2a_1_2**) (1 2) → Skip to FA006_w2_5 FA006_w2_5
3. Last IW, you were not employed and did work for family business without being paid, fill in the firms name / __________(**FA006_w2_2a_1_3**) (1 2) → Skip to FA006_w2_5 FA006_w2_5
4. Last IW, you were not employed and did farm work → Skip to FA006_w2_5 FA006_w2_5
5. Last IW, you did not work → Skip to FA006_w2_5 FA006_w2_5

FA006_w2_2a_2 You told us that you were working for someone else in the last IW, but you did not tell us the name of the employer in the last IW, what is the name of the employer you were working for in the last IW? [ZIWTime]

[IWER: Write the name of the household head if R works for a family.]

1. Fill in the employers name __________(**FA006_w2_2a_2_1**) （ 1 2） Name of the department __________ （ 1 2 ）

2. Last IW, you ran your own business, fill in the firms name / / __________(**FA006_w2_2a_2_2**) （ 1 2 ） → Skip to FA006_w2_5 FA006_w2_5

3. Last IW, you were not employed and did work for family business without being paid, fill in the firms name / __________(**FA006_w2_2a_2_3**) （ 1 2 ） → Skip to FA006_w2_5 FA006_w2_5

4. Last IW, you were not employed and did farm work → Skip to FA006_w2_5 FA006_w2_5

5. Last IW, you did not work → Skip to FA006_w2_5 FA006_w2_5

FA006_w2_2a_3 Are you still working for this employer?

1. Yes → Skip to FA006_w2_5 FA006_w2_5
2. No → Skip to FA006_w2_3 FA006_w2_3

FA006_w2_2b You told us that you were running ________ [Preload the name of the company/workplace from the last IW (ZF9), is the name correct? [ZIWTime] / [/（ZF9）] /

1. Yes → Skip to FA006_w2_2b_3 FA006_w2_2b_3
2. No

[(/) (2)]

FA006_w2_2b_ 1 What is the correct name?

1. Fill in the firms name / __________(**FA006_w2_2b_1_1**) （ 1 2） → Skip to FA006_w2_2b_3 FA006_w2_2b_3

2. Last IW, you were employed, fill in the firms name / __________(**FA006_w2_2b_1_2**) （ 1 2 ） Name of the department ____________ （ 1 2） → Skip to FA006_w2_5 FA006_w2_5

3. Last IW, you were not self-employed and did work for family business without being paid, fill in the firms name / ____________(**FA006_w2_2b_1_3**) （ 1 2 ） → Skip to FA006_w2_5 FA006_w2_5

4. Last IW, you were not self-employed and did farm work → Skip to FA006_w2_5 FA006_w2_5

5. Last IW, you did not work → Skip to FA006_w2_5 FA006_w2_5

FA006_w2_2b_2 You told us that you were running a company/business in the last IW, but you did not tell us the name of the company/business in the last IW, what is the name of the company/business you were running in the last IW? [ZIWTime] / / /

1. Fill in the firms name / __________(**FA006_w2_2b_2_1**) (1 2)
2. Last IW, you were employed, fill in the firms name / __________(**FA006_w2_2b_2_2**) (1 2) Name of the department ____________ (1 2) → Skip to FA006_w2_5 FA006_w2_5
3. Last IW, you were not self-employed and did work for family business without being paid, fill in the firms name / ____________(**FA006_w2_2b_2_3**) (1 2) → Skip to FA006_w2_5 FA006_w2_5
4. Last IW, you were not self-employed and did farm work → Skip to FA006_w2_5 FA006_w2_5
5. Last IW, you did not work → Skip to FA006_w2_5 FA006_w2_5

FA006_w2_2b_3 Are you still running this company/business? /

1. Yes → Skip to FA006_w2_5 FA006_w2_5
2. No → Skip to FA006_w2_3 FA006_w2_3

FA006_w2_2c You told us that you were working for an unpaid family business [Preload the name of the unpaid family business from the last IW (ZF10)], is the name correct [ZIWTime] [(ZF10)]

1. Yes → Skip to FA006_w2_2c_3 FA006_w2_2c_3
2. No

[(2)]

FA006_w2_2c_1 What is the correct name?

1. Fill in the firms name / __________(**FA006_w2_2c_1_1**) (1 2) → Skip to FA006_w2_2c_3 FA006_w2_2c_3
2. Last IW, you were employed, fill in the firms name / __________(**FA006_w2_2c_1_2**) (1 2) Name of the department ____________ (1 2) → Skip to FA006_w2_5 FA006_w2_5

3. Last IW, you ran your own business, fill in the firms name // ____(**FA006_w2_2c_1_3**) （ 1 2） → Skip to FA006_w2_5FA006_w2_5

4. → Skip to FA006_w2_5 FA006_w2_5 5. → Skip to FA006_w2_5 FA006_w2_5

FA006_w2_2c_2 You told us that you were working for an family business without a wage in the last IW, but you did not tell us the name of the name of the family business, what is the name of this family business you were working for without a wage in the last IW? [ZIWTime]

1. Fill in the firms name / __________(**FA006_w2_2c_2_1**) （ 1 2 ）
2. Last IW, you were employed, fill in the firms name / __________(**FA006_w2_2c_2_2**) （ 1 2 ） Name of the department ____________ （ 1 2） → Skip to FA006_w2_5 FA006_w2_5

3. Last IW, you ran your own business, fill in the firms name // ____(**FA006_w2_2c_2_3**) （ 1 2） → Skip to FA006_w2_5FA006_w2_5
4. → Skip to FA006_w2_5 FA006_w2_5
5. → Skip to FA006_w2_5 FA006_w2_5

FA006_w2_2c_3 Are you still working for this family business?

1. Yes → Skip to FA006_w2_5 FA006_w2_5
2. No → Skip to FA006_w2_3 FA006_w2_3

FA006_w2_3 When did you stop doing this job? ____ (**FA006_w2_3_1**) year ____ (**FA006_w2_3_2**) month

FA006_w2_4 What was the reason for you to stop doing this job?

1. Business closed /
2. Quit
3. I was laid off
4. I was fired
5. I went to school
6. I went abroad
7. I stopped working for health reasons

8. I stopped working for family reasons
9. I was transferred to another job
10. I started working off-farm locally
11. I went to work away from home
12. Better job in local area
13. Better job in another location
14. I retired
15. Other

FA006_w2_5 Starting from last IW [ZIWTime] to the current IW, how many other jobs have you been doing? (Note: the work is defined as every work you had been working for at least one month, excluding the work you were doing in the last IW and the work you are doing in the current IW) ____ Skip to FA007 Branchpoint if the answer is 0 0FA007 Branchpoint

FA006_w2_6 How many months were you working on these jobs? __Months ?

FA006_w2_7 While you were doing these jobs, how many weeks were you working on average per month? ___Weeks

FA006_w2_8 While you were doing these jobs, how many days were you working on average per week? _Days

FA006_w2_9 While you were doing these jobs, how many hours were you working on average per day? __Hours

FA006_w2_10 While you were doing these jobs, what is the average income you earned per month (excluding income tax and insurance payment)? ( ) ______Yuan .

FA007 BRANCHPOINT:

If old R answered work history questions in the last IW (ZF12= 1), then skip to FB011 (ZF12= 1) FB011

If old R did not answer work history questions in the last IW (ZF12≠ 1), and is working now(XF1= 1), then skip to FB001 (ZF12≠ 1) (XF1= 1) FB001

If old R did not answer work histroy questions in the last IW(ZF12≠ 1), and was working in the last IW(ZF13= 1), then skip to FB001 (ZF12≠ 1) (ZF13= 1)FB001

If old R had not worked prior to the last IW (ZF14= 1), and is not working now(XF1= 2), then skip to FA007 (ZF14= 1) (XF1= 2)FA007

FA007 Have you worked for at least three months during your lifetime (work includes agricultural work,earning wage work, self-employed activities, and unpaid family business work, et. al.)? ( )

1. Yes → Skip to FB001 FB001
2. No

FA008 Work includes all kinds of labour excluding doing your own housework, whether you earn wages or not. Are you sure that you didn t work at least three months during your lifetime?

1. Yes, never worked before
2. No, ever worked. → Skip to FB001 FB001

FA009 What is the main reason for you not to work in your lifetime?

1. Disabled (physical or psychological) ( ) 2. Homemaker
3. My family is too rich that I don t need to work 4. Taking care of siblings
5. Other

PROCEDURE :
Skip to FN001_w2 FN001_w2

FB WORK HISTORY

FB001 At what age (or in which year) did you start working (or farming), excluding part-time job during school time? () ( ) Age ____1 ··· 120 (**FB001_1**)Years or ____1900...2013 (**FB001_2**) Year

[IWER: Mark the year in four digits. 4]

FB002 What type of work unit was your first job,A government organization,shiye danwei, firm, NGO, inidivdual farmer, or household enterprise?

1. Government
2. Institutions
3. NGO ( )
4. Firm
5. Individual firm
6. Farmer
7. Individual household

8. Other

**PROCEDURE** : IfFB002= 4, then ask FB003, otherwise go on to FB004 . FB002=4 FB003FB004

[Show Card 20]

FB003 What is the ownership type of the business? 1. 100% State owned firm

2. State-controlled firm
3. 100% Collective-owned firm
4. Collective-controlled firm
5. 100% Private firm /
6. Private-controlled firm
7. 100% foreign-owned
8. Joint venture
9. Other joint-ownership
10. Other

FB004 Where was your first workplace located? 1. The same as permanent address

2. Another village/neighborhood in permanent addresss county/city/district /// _ (**FB004_1**) village/neighborhood /
3. Other ____ (**FB004_2**) province_city_county/city/district _ _ //
   ____(**FB004_3**) village/neighborhood /
4. Abroad

[IWER:]

**PROCEDURE** : If FB002= 4 and FB003= 1/2/3/4, then skip FB005 and FB006 FB002= 4 FB003= 1/2/3/4, FB005 FB006

FB005 Over your career did you ever have employment in a state owned, state controlled or collectively owned enterprise?

1. Yes
2. No → go to FB007 FB007

FB006 Specific ownership type

1. 100% State owned firm
2. State-controlled firm
3. 100% Collective-owned firm
4. Collective-controlled firm

FB007 Not counting current non-employment or retirement, did you stop working for an extended period of time （more than 1 year once） due to reasons of family, health, school, etc.? （ 1 ）

1. Yes
2. No → skip to FB011 FB011

FB008 How long were the interruptions in all? ____1...120(**FB008_1**) Years ____ 0...11(**FB008_2**) Months

[IWER: Mark the month and year using integert; if 0, please fill '0. 0]

FB009 When was the longest time period that you stopped working? From ____ 1900...2013 (**FB009_1**) year ____0...12 (**FB009_2**) month to ____1900...2013 (**FB009_3**) year ____0...12 (**FB009_4**) month

[IWER: Mark the year using four digits. Take down the month as its actual number. For example, write January as 1 not 01, December as 12. If do not remember month, fill '0. : 4 1 1 01,12 12**O** **]**

[Soft Check: Reprompt for FB008 and FB009 if longest time period is greater than Total from FB008 or (FB009_3 + FB009_4/12)-(FB009_1 + FB009_2/12) > FB008_1 + FB008_2/12.]

[Another check to avoid that interruption began before starting work. FB009_1 < FB001_1 or FB009_1 < CV009_a + FB001_1]

FB010 The reason was:

1. Family （）
2. Health （）
3. School
4. Unemployment/layoff /
5. Other

FB011 BRANCHPOINT:

If old R had completed retirement procedure (including early retirement) or internal retirement (ZF15= 1), skip FB011 and FB012 （ ） （ZF15= 1）FB011 FB012

FB011 Have you completed retirement procedures （including early retirement） or internal retirement（Note: Retirement from government departments, enterprises and institutions, not including retirement in the sense of getting agricultural insurance）? （ ） （ ）

1. Yes → skip to FB012 FB012

2. No

FB012 Have you completed receding position procedures 1. Yes

2. No

F1

FB012 BRANCHPOIN:

If new R is not working but has worked before (FA007= 1 or FA008= 2), skip to FK001. （FA007= 1 FA008= 2)）FK001.

If old R is not working (XF1= 2) but worked before (FA006_w2_5> 0 orZF13= 1 or ZF14= 2), skip to FK001. （XF1= 2） （FA006_w2_5> 0 ZF13= 1 ZF14= 2）FK001

FC CURRENT PRIMARY JOB/ OCCUPATION

FC001 BRANCHPOINT]:

IfR (including new R and old R) did farming last year (FA001= 1), ask FC001 （） （ FA001= 1 ）FC001

ifR did not do farming last year, but did non-agricultural work last week (XF2= 1), ask FC019. （XF2= 1） FC019.

FARM EMPLOYED

FC001 Did you work for other famers in wage for at least ten days in the past year（Agricultural work in wages） 10 （ ）

1. Yes
2. No → skip to FC008 FC008

F1

FC002 How many employers did you work for in the past month?

FC003 Where is your workplace for most time? 1. The same as permanent address

2. Another village/neighborhood in permanent addresss county/city/district /// _ (**FC003_1**) village/neighborhood /
3. Other ____(**FC003_2**) province_city_county/city/district _ _ // _(**FC003_3**) village/neighborhood /

4. Abroad
[IWER:]

FC004 How many months did you work on cropping (forestry), livestock, and fishing in wage for other famers in the past year? [( )] [()] [( )] ____0…12 Months

FC005 How many days did you work in wage for other famers per week on average during a normal work month in the past year? [( )] [()] [( )] ____0…7 Days

FC006 How many hours did you usually work in wage for other famers per day during a normal work day in the past year? [( )] [()] [( )] ____0…16 Hours

[Soft Check: Verify if number of hours per day is unreasonable, e.g., FC006 >16]

FC007 What is the average monthly wage did you get in your working months in the past year? ____Yuan

HOUSEHOLD AGRICULTURAL WORK

FC008 Did you work for your own household for at least ten days in the past year 10

1. Yes
2. No → skip to FC013 FC013

FC009 How many months did you work on [cropping (forestry), livestock, and fishing] for your own householdin the past year? [( )] [( )] [( )]___ 0…12 Months

FC010 How many days did you work for your own household per week on average during a normal work month in the past year? [( )] [()] [( )] ____ 0…7 Days

FC011 How many hours did you usually work for your own household per day during a normal work day in the past year? [( )] [()] [( )] __ 0…24 Hours

[A check similar to the one for FC006. FC011 > 16 is unreasonable]

FC012 Where is your workplace for most time? [preload sampling community ID]

1. The same as permanent address
2. Another village/neighborhood in permanent addresss county/city/district /// _ (**FC012_1**) village/neighborhood /
3. Other ____(**FC012_2**) province_city_county/city/district _ _ // _ (**FC012_3**) village/neighborhood /
4. Abroad

[IWER:]

FC013 How many days of work did you miss last year due to health problems? ____ 0...366 Days

[IWER: Mark 0 if you didnt miss any work days. 0]

FC014 Besides agricultural work, did you work for at least one hour last week in wage or self-employed work or umpaid family business? ()

1. Yes → skip to FC019 FC019
2. No

FC015 Do you have wage or self-employed work but are temporarily laid-off or are on sick, seasonal, or other leave or in-job training? ( )

1. Yes → skip to FC018 FC018
2. No

FC016 In what month and year did leave or attend training? // ____1900...2013 (**FC016_1**) Year __0...12 (**FC016_2**) Month

[IWER: Mark the year using four digits. Take down the month as its actual number. For example, write January as 1 not 01, December as 12. If do not remember month, fill '0. : 4 1 1 01,12 12**O** **]**

[Same check as the one for FA004 . (Interview Year –FC016_1 + (Interview month – FC016_2)/12 > 1?]

FC017 Do you expect to go back to this job at a definite time in the future or within 6 months? 6

1. Yes → skip to FC019 FC019
2. No

FC018 At what age do you plan to stop working? Stopping work in this context shall refer to having stopped all income-related activities, unpaid family business and having no

intention of engaging in anything more serious than small pastime work. ?
1… 120 Years old

[IWER: Please tell me the approximate age. Mark 0 if you plan to keep working until you are physically able. 0]

F1

FC018 BRANCHPOINT:

Skip to pension and social security section (FN001_w2) if have not processed retirement and receding (FB011= 2 and FB012= 2) （FB011= 2 FB012= 2）（FN001_w2）

Skip to retirement section (FM001) if processed retirement or receding (FB011= 1 and FB012= 1 or processed retirement or receding in the last IW (ZF15= 1 or ZF16= 1) （FB011= 1 FB012= 1 (ZF15= 1 or ZF16= 1）（FM001）

FC019 Besides agricultural work, do you currently hold more than one non-agricultural job? （）

[IWER: non-agricultural job includes paid jobs, self-employed activities, unpaid family business work, et. al. Activities without pay, such as voluntary work, are not included. 2]

1. Yes → skip to FC020 FC020
2. No → skip to FC021 FC021

FC020 Among all your jobs, which one is your main job? [Main job is defined as the job at which you work the longest hours] Do you earn a wage or do you run your own business or work for unpaid family business? [ ]

1. Employed → skip to FD001 FD001
2. Self-employed → skip to FH001 FH001
3. unpaid family business → skip to FH001 FH001

FC021 How do you describe your non-agricultural job? Do you earn a wage or do you run your own business or work for unpaid family business?

1. Employed → skip to FD001 FD001
2. Self-employed → skip to FH001 FH001
3. unpaid family business → skip to FH001 FH001

FD EMPLOYED

FD001 Do you receive wages from your current workplace or receive them from a dispatch/contract company?

1. Place of work
2. Labor dispatch company

F1

[CAPI: For dispatched/contract workers (FD001= 2), mention the following for questions FD002 -FD016. (FD001= 2) FD002-FD016] The next few questions pertain to the situation at your current workplace, and not to the company that has dispatched/contracted you out.

FD002 Do you work for a government organization, institution, firm, NGO, individual farmer, or resident household? （ ）

1. Government
2. Institutions
3. NGO （）
4. Firm
5. Individual firm
6. Farmer
7. Individual household
8. Other

**FD003 BRANCHPOINT**:<br>For old R: if old R is working for the same employer (FA006_w2_2a_3= 1), skip FD003.<br>（FA006_w2_2a_3= 1）FD003

[INTRO: We will ask your work history later.In order to distinguish the work units, we need the full name and address of your employer.]

FD003 What is the name of your workplace/employer? Please state specifically the name of your company or institution. （） ______**(FD003_1)** （ 1 2 ）

________**(FD003_2)** （ 1 2 ）

[IWER: Write the name of the household head if R works for a family.]

FD004 Where is your workplace located? (preload sampling community ID)

1. The same as permanent address
2. Another village/neighborhood in permanent addresss county/city/district /// _ (**FD004_ 1**) village/neighborhood /
3. Other ____(**FD004_2**) province_city_county/city/district _ _ // _(**FD004_3**) village/neighborhood /
4. Abroad

[IWER:]

**PROCEDURE**
IfR is working for individual farmer or resident household (FD002= 6; 7),skip to FD011.<br>（FD002= 6; 7）FD011

**FD005 BRANCHPOINT**:
If old R is working for a different employer (FA006_w2_2a_3= 2),skip to FD005 （ FA006_w2_2a_3= 2）FD005<br><br>If old R is working for the same employer but the information of the type of business is missing in the last IW (FA006_w2_2a_3= 1 & ZF8_1= :), skip to FD005_w2_2. （ FA006_w2_2a_3= 1 & ZF8_1= :） FD005_w2_2.<br><br>If old R is working for the same employer and the information of the type of business is not missing (FA006_w2_2a_3= 1 & ZF8_1= :), skip to FD005_w2_1. （ FA006_w2_2a_3= 1 & ZF8_1= :） FD005_w2_1<br><br>New R please answer FD005. FD005

FD005 What kind of business or industry do you work in—that is, what does your workplace primarily make or do? ___________ （ 1 2）

[IWER: Type of business]

**PROCEDURE**
Skip to FD006. FD006.

FD005_w2_1 You told us in the last IW that your workplace did or made [ZIW- Time] [preload information of the type of business of workplace from last IW (ZF8_1)]is the information correct?

1. Yes, it is correct → skip to FD005_w2_3 FD005_w2_3 2. No, it is not correct

FD005_w2_2 What did your workplace do or make in the last IW? [ZIWTime] ___________
1 2)
[IWER: Type of business]

FD005_w2_3 Is your workplace still doing or making it? 1. Yes → skip to FD006
FD006
2. No

FD005_w2_4 What is your workplace doing or making now? ___________ 1 2)
[IWER: Type of business]

FD005_w2_5 When did your workplace start doing this? _____ (**FD005_w2_5_1**) year ___ (**FD005_w2_5_2**) month

> **PROCEDURE**
> If government employee (FD002= 1), ask FD006 to FD007, then skip to FD011.
> FD006 to FD007 FD011

FD006 Are you a civil servant?
1. Yes
2. No

FD007 Are you a formal employee of an establishment? 1. Yes
2. No

> **PROCEDURE**
> If institution (FD002 = 2), ask FD008 to FD009, then skip to FD011. FD008 to FD009
> FD011

FD008 Is your institution operated as a firm or as a government unit?
1. As a firm
2. As a government unit

FD009 Are you a formal employee of an establishment? 1. Yes
2. No

> **PROCEDURE**
> If firm (FD002= 4), ask from FD010, FD010

[Show Card 20]

FD010 What is the ownership type of the business? 1. 100% State owned firm
2. State-controlled firm
3. 100% Collective-owned firm
4. Collective-controlled firm
5. 100% Private firm /
6. Private-controlled firm
7. 100% foreign-owned
8. Joint venture
9. Other joint-ownership
10. Other

FD011 When did you start working for this employer? _1900...2013 (**FD011_1**) Year ____ 0...12 (**FD011_2**) Month

[IWER: Mark the year using four digits. Take down the month as its actual number. For example, write January as 1 not 01, December as 12. If do not remember month, fill '0. : 4 1 1 01,12 12**O** **]**

[Soft Check: Consistency of employment start date. Make Sure that the individual was at least a minimum age when he/she started working for this employer (e.g., prompt to check if under 16?). Specifically (FD011_ 1+FD011_2/12)-(CV009_a+CV009_b/12)<16 prompts a soft check.]

[Replace 16 in the above soft check with FB001. (FD011_1 + FD011_2/12)-(CV009_a + CV009_b/12) < FB001_ 1 or FD011_ 1 < FB001_2]

**PROCEDURE** :<br>（ZF1 = 4）FD011 FD011_1FD011_2 [ZIWTime]FD011_w2_2

FD011_w2_2 When did you begin working in this unit? ____1900...2013 (**FD011_w2_2_1**) Year ____0...12 (**FD011_w2_2_2**) Month

[FD011_w2_2（ZIWTime）]

FD012 BRANCHPOINT :

If old R is working for a different employer (FA006_w2_2a_3= 2), skip to FD012 （ FA006_w2_2a_3= 2） FD012

If old R is working for the same employer but the information of the type of work is missing in the last IW (FA006_w2_2a_3= 1 & ZF17= :), skip to FD012_w2_2. （ FA006_w2_2a_3= 1 &ZF17= :） FD012_w2_2

If old R is working for the same employer and the information of the type of work is not missing (FA006_w2_2a_3= 1 & ZF17 ≠ :), skip to FD012_w2_1. （ FA006_w2_2a_3= 1 & ZF17≠ :） FD012_w2_1

New R please answer FD012. FD012

FD012 What sort of work do you do? ___________(**FD012_ 1**) 1 2） ___________(**FD012_2**)（ 1 2） → Skip to FD013. FD013

[IWER: Ask about the specific work that R does.]

FD012_w2_1 You told us in the last IW that you were doing [ZIWTime] [preload information of the work from last IW (ZF17)] is the information correct?

1. Yes, it is correct → skip to FD012_w2_3 FD012_w2_3 2. No, it is not correct

FD012_w2_2 What were you doing in the last IW? [ZIWTime] _____(**FD012_w2_2_1**) _____ (**FD012_w2_2_2**) （ 1 2）

[IWER: Ask about the specific work that R does.]

FD012_w2_3 Are you still doing this work?

1. Yes I am → skip to FD013 branchpoint FD013 branchpoint 2. No I am not

FD012_w2_4 What sort of work are you doing now? ____________ (**FD012_w2_4_1**)（ 1 2 ） __________(**FD012_w2_4_2**)（ 1 2）

[IWER: Ask about the specific work that R does.]

FD012_w2_5 When did you start doing your current work? ____ (**FD012_w2_5_1**) year _ (**FD012_w2_5_2**) month

FD013 BRANCHPOINT :

If old R is working for a different employer (FA006_w2_2a_3= 2),skip to FD013 （FA006_w2_2a_3= 2）FD013

If old R is working for the same employer but the information of the type of business is missing in the last IW (FA006_w2_2a_3= 1 & ZF18= :), skip to FD013_w2_2. （FA006_w2_2a_3= 1 &ZF18= :）FD013_w2_2

If old R is working for the same employer and the information of the type of business is not missing (FA006_w2_2a_3= 1 & ZF18 ≠ :), skip to FD013_w2_1. （FA006_w2_2a_3= 1 & ZF18≠ :） FD013_w2_1

New R please answer FD013. FD013

FD013 What is your current position?

1. Clerk/worker
2. Team Leader （）
3. Section Chief
4. Director of a division
5. Director-General of a bureau and above
6. Village Leader
7. Township Leader
8. Division manager
9. Overall/General manager
10. Others

PROCEDURE

Skip to FD014. FD014.

FD013_w2_1 You told us that in the last IW you were [ZIWTime] ______ [Preload information from the last IW （ZF18)], is the information correct?

1. Yes → skip to FD013_w2_3 FD013_w2_3
2. No

FD013_w2_2 What were your position in the last IW? [ZIWTime] ______

1. Clerk/worker
2. Team Leader （）
3. Section Chief
4. Director of a division
5. Director-General of a bureau and above
6. Village Leader
7. Township Leader

8. Division manager
9. Overall/General manager
10. Others

FD013_w2_3 Are you still holding this position?

1. Yes → skip to FD014 branchpoint FD014 branchpoint
2. No

FD013_w2_4 What is your current position?

1. Clerk/worker
2. Team Leader （）
3. Section Chief
4. Director of a division
5. Director-General of a bureau and above
6. Village Leader
7. Township Leader
8. Division manager
9. Overall/General manager
10. Others

FD013_w2_5 When did you start doing your current work? ____ (**FD013_w2_5_1**) year _ (**FD013_w2_5_2**) month

FD014 BRANCHPOINT :

If old R is working for a different employer (FA006_w2_2a_3= 2),skip to FD014 （FA006_w2_2a_3= 2）FD014

If old R is working for the same employer but the information of the professional/technical level is missing in the last IW (FA006_w2_2a_3= 1 & ZF19_1= :), skip to FD014_w2_2. /（FA006_w2_2a_3= 1 &ZF19_1= :）FD014_w2_2

If old R is working for the same employer and the information of prefessional/technical level is not missing (FA006_w2_2a_3= 1 & ZF19≠= :), skip to FD014_w2_1. /（FA006_w2_2a_3= 1 & ZF19≠= :） FD014_w2_1

New R please answer FD014. FD014

FD014 What is your current professional/technical level? / 1. Technician

2. Primary level
3. Intermediate level
4. Advanced level
5. No professional/technical level

F1/

PROCEDURE
Skip to Procedure prior FD015. FD015.

FD014_w2_1 You told us in the last IW that your professional/technical level was [ZIWTime] / ______[preload the information of professional/ technical level from the last IW / (ZF19)], is the information correct?

1. Yes → skip to FD014_w2_3 FD014_w2_3
2. No

FD014_w2_2 What was your professional/technical level in the last IW? [ZIW- Time] /

1. Technician
2. Primary level
3. Intermediate level
4. Advanced level
5. No professional/technical level

F1/

FD014_w2_3 Is it still your professional/technical level? / 1. Yes, it is → Skip to Procedure prior FD015. FD015 . 2. Noit is not

FD014_w2_4 What is your current professional/technical level? /

1. Technician
2. Primary level
3. Intermediate level
4. Advanced level
5. No professional/technical level

F1/

FD014_w2_5 When did you start having the current professional/technical level? / ____ (**FD014_w2_5_1**) year ____ (**FD014_w2_5_2**) month

PROCEDURE :
For new R: Skip FD015-FD016 if answer to FD013 is 2 − 9. FD013 2 − 9FD015FD016
For old R: Skip FD015-FD016 if old Rs position in the last IW (ZF18) is 2 − 9 & FD013_w2_1= 1 & FD013_w2_3= 1 or ifFD013_w2_2= 2 − 9 or ifFD013_w2_4= 2 − 9. (ZF18) 2 − 9 FD013_w2_1= 1 FD013_w2_3= 1 FD013_w2_2= 2 − 9FD013_w2_4= 2 − 9 FD015-FD016

FD015 Are you in a position to supervise others?

1. Yes
2. No → Skip to FD017. FD017

FD016 How many people are there under your supervision? 1. 1~5 people 1~5

2. 6~10 people 6~10
3. 11~15 people 11~15
4. 16~30 people 16~30
5. 31~99 people 31~99
6. More than 100 people 100

[CAPI: For dispatched worker(FD001 = 2), prompt for FD017-FD030 (FD001= 2) FD017-FD030

The next few questions are about your dispatch work unit.]

PROCEDURE :

For new R: Do not ask FD017 to FD019 if FD013= 3 – 5 (government officials).
FD013= 3 – 5 ( ) FD017 , FD018 , FD019.
For old R: Do not ask FD017 to FD019 if old Rs position in the last IW (ZF18) is 3-5 & FD013_w2_1= 1 & FD013_w2_3= 1 or ifFD013_w2_2= 3 – 5 or ifFD013_w2_4= 3 – 5.
(ZF18) 3 – 5 FD013_w2_1= 1 FD013_w2_3= 1 FD013_w2_2= 3 – 5 FD013_w2_4= 3 – 5 FD017 , FD018 , FD019.

FD017 What is your employment type at your current workplace?

1. Regular worker
2. Contract worker
3. Casual/Part-time worker /

FD018 Do you have a personnel file?

1. Yes
2. No → Skip to FD020. FD020

FD019 Where is your personnel file kept?

1. With my current employer
2. Other work unit but not current employer () 3. With the Job Service Center in this city /
4. HuKou place in other city
5. Other city

FD020 Did you receive a labor contract (or employment contract) in written form from your current workplace(or labor dispatch company)? /

1. Yes
2. No → Skip to FD024. FD024

FD021 What is the agreed period of employment (labor contract period)?
1. Defined period ____0...100 (**FD021_1**) Years __0...11 (**FD021_2**) Months
   [IWER: if do not remember months, please fill '0. 0]
2. Not defined → Skip to FD024. FD024
3. Same as the term of the project

FD022 Has the current employment contract ever been renewed?
1. Yes
2. No → Skip to FD024. FD024

FD023 How many times has the contract been renewed? _____ 1...50 times

FD024 How long do you expect to work at your current workplace?
1. Less than one year 1
2. One to two years 1-2
3. Two to three years 2-3 → Skip FD025. FD025
4. More than three years 3 → Skip FD025. FD025

FD025 Why do you expect so?
1. Because the predefined contract period will expire
2. Because typically the contract expires (although there s no written contract) （ ）
3. Because I was hired under the condition that I would resign upon the request of my employer
4. Because the current job/project will be completed /
5. Because the person I am substituting/replacing will return to work
6. Because I can only work during certain seasons
7. Because I plan to find another job that better suits my job aptitude, abilities, and preferences
8. Because I will reach retirement age as set by regulations/practice
9. Because of family care responsibilities, poor health, etc.

10. Other

PROCEDURE :
If R has not processed retirement (FB011= 2) ask: :

FD026 Is this work unit going to process retirement for you?
1. Yes
2. No → Skip to FD029. FD029

FD027 At what age will you process retirement? __ 45...120 Years old → Skip to FD029. FD029

[Soft Check for Reasonable Age: If (FD027< 50 & CV004== 2) or (FD027< 55 & CV004== 1) prompt for verification.] [Add another check to see if the reported age above is smaller than actual age. To be specific, FD027<(Interview Year – CV009_a)]

PROCEDURE :
IfR has processed retirement (FB011= 1 or ZF15= 1) ask: :

FD028 Did you process retirement through this work unit?
1. Yes
2. No

FD029 Except for national/public holidays, how many days of paid vacation do you have this year at your current workplace? ___ 0...366 Days

[IWER: Mark 0 if there is no paid vacation. 0] [Soft Check for reasonable range: If FD029> 30?, prompt for verification]

FD030 How many days of work did you miss at this current job in the past year due to health problems? ___ 0...366 Days

[IWER: Mark 0 if you didnt miss work. 0]

PROCEDURE :
If FD030= 0, skip FD031. FD030= 0FD031

FD031 In these days, how many did not deduct wage or bonus? ___ 0...366 Days
[Soft Check for reasonable range: If FD031>FD030?, prompt for verification]

FE QUESTIONS ABOUT LABOR SUPPLY

[CAPI: For dispatched worker(FD001= 2), prompt for FE001-FE003 (FD001= 2) FE001-FE003] The next few questions about labor supply are about the situation of your work place, not dispatch work unit.

FE001 Counting paid vacations and sick leave not deducting wage as work, how many months did you work in the past year? ( ) __ 0...12 Months

FE002 How many days a week did you work on average in the past year? __0...7 Days

FE003 How many hours did you work per day on average in the past year, excluding meal breaks but including any paid or unpaid overtime? ( ) __0...24 Hours

[Soft Check: Verify if number of hours per day is unreasonable, e.g., FE003 >16]

FF QUESTIONS ABOUT WAGES

[CAPI: For dispatched worker(FD001= 2), prompt for FF001-FG014 (FD001= 2) FF001-FG014] The following questions about salary and benefits refer to what you receive from the dispatch company.

FF001 How is your wage paid mainly? Is it regularly paid, contract-based, performance-based, or other? If it is regularly paid, please tell me how often you receive your wages. Do you have a yearly contract, monthly, weekly, daily, or hourly? Please select one.

1. Yearly salary
2. Monthly salary → Skip to FF004. FF004
3. Weekly salary → Skip to FF006. FF006
4. Daily salary → Skip to FF008. FF008
5. Hourly salary → Skip to FF010. FF010
6. Contract-based → Skip to FF012. FF012
7. Performance-based → Skip to FF012. FF012
8. Other → Skip to FF012. FF012

FF002 What is the after-tax salary including bonus in the last year? ( ) ____**(FF002_1)** Yuan

____(**FF002_2**) Yuan __(**FF002_3**)%

[Soft Check: Prompt for clarification if under a low threshold, e.g. under 1200 RMB annual. Specifically, prompt to clarify if FF002< 1200 RMB.]

FF003 [IWER: If R is unwilling to answer or does not remember, ask unfolding bracket questions here.] ( ) 10,000 /30,000 /50,000 /100,000 /200,000 yuan

FF003_w2 [IWER: If R is unwilling to answer or does not remember, ask unfolding bracket questions here.] 10,000 /30,000 /50,000 /100,000 /200,000 yuan

**PROCEDURE** :<br>Skip to FF014. FF014

FF004 What is the after-tax salary including bonus in the last month? ( ) ____(**FF004_1**) Yuan ____(**FF004_2**) Yuan __(**FF004_3**)%

[Soft Check: Prompt for clarification if under a low threshold, e.g. under 100 RMB per month. Specifically, prompt to clarify if FF004 < 100 RMB.]

FF005 [IWER: If R is unwilling to answer or does not remember, ask unfolding bracket questions here.] ( ) 500 /1,000 /2,500 /5,000 /10,000 yuan

FF005_w2 [IWER: If R is unwilling to answer or does not remember, ask unfolding bracket questions here.] 500 /1,000 /2,500 /5,000 /10,000 yuan

**PROCEDURE** :<br>Skip to FF014. FF014

FF006 What is the wage including bonus last week? ( ) ____Yuan

[Soft Check: Prompt for clarification if under a low threshold, e.g. under 25 RMB per week. Specifically, prompt to clarify if FF006 < 25 RMB.]

**PROCEDURE** :<br>Skip to FF012. FF012

FF008 What is the usual daily wage? ____Yuan

[Soft Check: Prompt for clarification if under a low threshold, e.g. under 5 RMB per day. Specifically, prompt to clarify if FF008 < 5 RMB.]

FF009 [IWER: If R is unwilling to answer or does not remember, ask unfolding bracket questions here.] 20 /50 /100 /200 /500 yuan

PROCEDURE :
Skip to FF012. FF012

FF010 What is your hourly wage? ____Yuan

[Soft Check: Prompt for clarification if under a low threshold, e.g. under 1 RMB per hour. Specifically, prompt to clarify if FF010 < 1 RMB.]

PROCEDURE :
Skip to FF012. FF012

FF012 How much on average do you receive last month after taxes (including bonus)? , _ (**FF012_1**) Yuan ____(**FF012_2**) Yuan __(**FF012_3**)%

[Soft Check: Prompt for clarification if under a low threshold, e.g. under 100 RMB per month. Specifically, prompt to clarify if FF012 < 100 RMB.]

FF013 [IWER: If R is unwilling to answer or does not remember, ask unfolding bracket questions here.] , 1,000 /3,000 /5,000 /10,000 /20,000 yuan

FF013_w2 [IWER: If R is unwilling to answer or does not remember, ask unfolding bracket questions here.] 1,000 /3,000 /5,000 /10,000 /20,000 yuan

FF014 What is the value of all other bonuses (not paid at same time as regular wage) received in the past year? （ ） （） ____Yuan

[Soft Check: Prompt on bonus and monthly earnings if bonus is more than five times monthly net income]

FF015 [IWER: If R is unwilling to answer or does not remember, ask unfolding bracket questions here.] 1,000 /3,000 /5,000 /10,000 /20,000 yuan

FG Fringe Benefits

[Show Card 21]

FG001 The following are fringe benefits which maybe provided by a company. Please answer if the following are provided by your current workplace and whether you benefit from the following. (Check all that apply) ()

1. Free lunch
2. Free breakfast
3. Free dinner
4. Meal cash subsidy
5. Transportation cash subsidizations
6. Free housing
7. Subsidization of housing
8. Company car
9. Company bus
10. Other subsidies （）
11. None → Skip to FG003 FG003

**PROCEDURE** :
For each choice of FG001, ask FG002. FG002

FG002 How much is the value of the subsidy per month? （） _Yuan

**PROCEDURE** :
If FD006 = 1 or FD007 = 1 or FD009 = 1 , then skip FD003-FD014 FD006 = 1 FD007 = 1 FD009 = 1 , FD003-FD014

**PROCEDURE** : If R has not processed retirement(FB011= 2), ask FD003-FD014 （FB011= 2） FD003-FD014

[Show Card 22]

FG003 Does your employer provide pension insurance, health insurance, unemployment insurance, workers injury insurance and maternity insurance (Choose all that apply) （）

1. Pension → Skip to FG009 FG009
2. Health insurance → Skip to FG009 FG009
3. Unemployment insurance → Skip to FG009 FG009 4. Workers injury insurance → Skip to FG009 FG009
5. Maternity insurance → Skip to FG009 FG009
6. None

FG004 Why are you not covered by the above mentioned social insurance through your employer?（Choose all that apply）?（ ） ?（）

1. There is no social insurance through employment in my local area → Skip to FG014 FG014
2. Social insurance through work is available in my local area but my employer does not provide it to me → Skip to FG014 FG014
3. My employer offers it but I am unwilling to join → Skip to FG014 FG014
4. I am in the trial period → Skip to FG014 FG014
5. I have social insurance from elsewhere → Skip to FG005 FG005
6. I have passed retirement age → Skip to FG014 FG014

FG005 From where do you have/receive the above mentioned social insurance? （）

1. I am covered through another work unit → Skip to FG006 FG006
2. I contribute through the job service center → Skip to FG008 FG008

FG006 Why do you contribute the above mentioned social insurance fees through another work unit? （ ）

1. I was laid off by that work unit but my employment contract has not terminated
2. I am on leave from that employer
3. I keep my position but do not receive pay from that work unit
4. A friend in that work unit is helping me this way 5. Other

FG007 Where is this work unit located?

1. Same city
2. Different city, same province
3. Different province

**PROCEDURE** :
Skip to FF009. FF009

FG008 Who pays for your contribution through the job service center? （）

1. My employer
2. Myself
3. My employer and myself

**PROCEDURE** :
All those paying social insurance through whatever means, ask(FG003 = 1−4 orFG004 = 5). FG009 – FG013

FG009 Is the salary you told me about earlier the net amount after paying for the above benefits?
1. Yes
2. No

FG010 On what income base is the contribution to the above mentioned social insurance determined? （ ） ____ Yuan/month /

FG011 Do you know how much you or your employer (another work unit) contributes to the above benefits? （） （ ）
1. I know the amount by myself and the employer
2. I know the amount by myself but not the employer → Skip to FG013 FG013
3. I know neither my contribution nor my employer s contribution. → Skip to FG014 FG014

FG012 How much is your employers contribution in total? _Yuan/month /
Soft Check Income Report and FG012 if Contribution is Greater than Monthly Income

FG013 How much is your own contribution in total? （ ） ____Yuan/month / Soft Check Income Report and FG013 if Contribution is Greater than Monthly Income

FG014 Does your employer provide funding for public housing?
1. Yes
2. No

F1 Soft Check: Verify if FG014 > than some threshold.

FG015 At what age do you plan to stop working? Stopping work in this context shall refer to having stopped all income-related activities and unpaid family business and having no intention of engaging in anything more serious than small pastime work. ?____
0…120 Years old

F1 Soft

Check: Prompt for correction if current age is greater than response, e.g. (Interview Year+ Interview month/12) – (CV009_a+CV009_b/12) > FG015

**PROCEDURE** :
Skip to FJ001. FJ001

FH NON-FARM SELF-EMPLOYED AND UNPAID FAMILY BUSINESS

FH001 How many months did you work in the past year? __ 0…12 Months

FH002 How many days did you work per week on average in the past year? __ 0…7 Days

FH003 How many hours did you work per day on average in the past year, excluding meal breaks but including any paid or unpaid overtime on a normal work month?
__ 0…24 hours

Soft Check: Verify if number of hours per day is unreasonable, e.g., FH003 >16

FH004 How many days of work did you miss in the past year due to health problems? ___
0…366 Days

[IWER: Mark 0 if you didnt miss any work days. 0]

[Next are some questions about the main self-employed work.]

**PROCEDURE** :
IF FC020= 2 or FC021= 2[Self-employed], ask FC020= 2 FC021= 2 :
If old R is running the same company or workplace (FA006_w2_2b_3= 1), skipFH005 /FH005

FH005 What is the name of your company or workplace? ( ) ____________ (1 2)

[IWER: If there is more than one company, ask about the main one. Mark 0 if there is no name. 0]

FH006 Where is your company or workplace located? / 1. The same as permanent address

2. Another village/neighborhood in permanent addresss county/city/district /// _ (**FH006_1**) village/neighborhood /

3. Other ____(**FH006_2**) province_city_county/city/district _ _ // ____(**FH006_3**) village/neighborhood /

4. Abroad

[IWER:]

**FH007 BRANCHPOINT]**:
If old R is running the same company or workplace (FA006_w2_2b_3= 1) and the information of the type of business is not missing, skip to FH007_w2_1 /FH007_w2_1
If old R is running the same company or workplace (FA006_w2_2b_3= 1 & ZF9_1= :) and the information of the type of business is missing, skip to FH007_w2_2 . / FH007_w2_2
If old R is running the different company or workplace (FA006_w2_2b_3= 1 & ZF9_1≠= :), skip to FH007. / FH007 New R please answerFH007. FH007

FH007 What kind of business or industry do you work in—that is, what does your company do or make? ____________ (1 2)

[IWER: Type of business]

**PROCEDURE** :
Skip to FH008. FH008

FH007_w2_1 In the last IW, you told us that your company did or made [ZIW- Time]/ ______ [preload information of type of busi- ness of company or business (ZF9_1) /], is the information correct?

1. Yes → skip to FH007_w2_3 FH007_w2_3
2. No

FH007_w2_2 What did your company do or make in the last IW? [ZIWTime] / _________. (1 2)

[IWER: Type of business]

FH007_w2_3 Is your company still doing or making it? / 1. Yes → skip to FH009 FH009

2. No

FH007_w2_4 What is your company doing or making now? / ___. (1 2)
[IWER: Type of business]

**PROCEDURE** :<br>If old R is running the same company or business(FA006_w2_2b_3= 1), skipFH008 /FH008

FH008 When did you start working at the current company or workplace? / ____ 1900…2013 (**FH008_1**) Year __0…12 (**FH008_2**) Month

[IWER: Mark the year using four digits. Take down the month as its actual number. For example, write January as 1 not 01, December as 12. If do not remember month, fill '0. : 4 1 1 01, 12 12**O** **]**

[A check similar to the one for FD011. (FD011_1 + FD011_2 /12)- (CV009_a + CV009_b/12) < 16 or (FD011_1 + FD011_2 /12)- (CV009_a + CV009_b/12) < FB001_1 or FH008_1 < FB001_2]

FH009 Do any other household members work in the same self-employed activity?

1. Yes → skip to FI001 FI001
2. No

[IWER: Income from self-employment ifR is the single operator（FH009= 2）. If other family member involved, income is asked in household section. FI001,]

FH010 Not including spending on fixed capital, what is your best estimate of net income earned from this activity in the last year? Remember to consider the following types of costs: energy, housing or equipment rental, raw materials, transportation, marketing, wages, taxes, and other fees. / ____ Yuan

Soft Check: Prompt for verification if net income is less than 1200 RMB / year. Or, prompt if FH010 <1200 RMB

FH011 [IWER: If R is unwilling to answer or does not remember, ask unfolding bracket questions here.] 5,000 /10,000 /50,000 /100,000 /200,000 yuan

PROCEDURE :
Skip to FI001 FI001

Next are some questions about your unpaid family business.

FH012 What is the name of company or workplace that you work in without wage? _____
(1 2)
[IWER:Mark 0 if there is no name. 0 .]

FH013 Where is this company or workplace loacted? / 1. The same as permanent address
2. Another village/neighborhood in permanent addresss county/city/district /// _ (**FH013_ 1**) village/ neighborhood /
3. Other ____(**FH013_2**) province_city_county/ city/ district _ _ // _(**FH013_3**) village/neighborhood /
4. Abroad
[IWER:]

FH014 BRANCHPOINT]:
If old R is working in a different unpaid family business(FA006_w2_2c_3= 2) skip to FH014 FH014
If old R is working in a same unpaid family business but the information of the type of business is missing in the last IW(FA006_w2_2c_3= 1 & ZF10_1= :) skip to FH014_w2_2. FH014_w2_2
If old R is working in a same unpaid family business and the information of the type of business is not missing in the last IW (FA006_w2_2c_3= 1 & ZF10_1≠ :), skip to FH014_w2_1. / FH014_w2_1
New R please answerFH014. FH014

FH014 What kind of business or industry do you work in—that is, what does this company do or make? _________. (1 2)
[IWER: Type of business]

PROCEDURE :
Skip to FH015 FH015

FH014_w2_1 You told us in the last IW that the type of business of company or workplace you worked without wage did or made [ZIWTime] / _________[preload information of the type of business from last IW (ZF10_1) /]is the information correct?

1. Yes, it is correct → skip to FH014_w2_3 FH014_w2_3 2. No, it is not correct

FH014_w2_2 What did the company or workplace do or make in the last IW? [ZIWTime] / . （1 2）

[IWER: Type of business]

FH014_w2_3 Is the company or workplace still doing or making it? /

1. Yes → skip to FH015 Branchpoint FH015 Branchpoint 2. No

FH014_w2_4 What is the company or workplace doing or making now? / _________. （1 2）

[IWER: Type of business]

FH014_w2_5 When did the company or workplace start doing this? / ____ (**FB014_w2_5_1**) Years _____(**FB014_w2_5_2**) Months

FH015 BRANCHPOINT :

If old R is working in a different unpaid family business FA006_w2_2c_3= 2) skip to FH015 FH015

If old R is working in a same unpaid family business but the information of the work is missing in the last IW (FA006_w2_2c_3= 1 & ZF22= :) skip to FH015_w2_2. FH015_w2_2 If old R is working in a same unpaid family business and the information of the work is not missing in the last IW (FA006_w2_2c_3= 1 & ZF22≠ :), skip to FH015_w2_1. FH015_w2_1

New R please answerFH015. FH015

FH015 What sort of work did you do? _________. （ 1 2 ）

[IWER: Ask the specific work.]

PROCEDURE :

Skip to FH016 FH016

FH015_w2_1 You told us in the last IW that you were doing [ZIWTime] ________[preload information of the work from last IW (ZF22)]is it correct?

1. Yes → skip to FH015_w2_3 FH015_w2_3

2. No

FH015_w2_2 What were you doing in the last IW? [ZIWTime] _. (1 2)
[IWER: Ask the specific work.]

FH015_w2_3 Are you still doing this work?
1. Yes → skip to FH016 FH016
2. No

FH015_w2_4 What sort of work are you doing now? _________. (1 2)
[IWER: Ask about the specific work that R does.

FH015_w2_5 When did you start doing your current work? ____ (**FB015_w2_5_1**) Years (**FB015_w2_5_2**) Months

FH016 How many family members, relatives or friends who work without payment are there including yourself? / ( ) __ People

FH017 When did this company or workplace start operation/ / ____1900...2013 (**FH017_1**) Year __0...12 (**FH017_2**) Month

FH018 When did you start working at the current company or workplace? / ____ 1900...2013 (**FH018_1**) Year __0...12 (**FH018_2**) Month

FH019 Do any other household members work in the same company or workplace? /
1. Yes → skip to FI001 FI001
2. No

[IWER: Income from self-employment if no other household members work in (FH019= 2) . If other family member involved, income is asked in household section. / FI001,]

FH020 Not including spending on fixed capital, what is your best estimate of net income earned from this activity in the last year? Remember to consider the following types of costs: energy, housing or equipment rental, raw materials, transportation, marketing, wages, taxes, and other fees.

/ ____ Yuan

Soft Check: Prompt for verification if net income is less than 1200 RMB / year. Or, prompt if FH020 < 1200 RMB

FH020_bracket [IWER: If R is unwilling to answer or does not remember, ask unfolding bracket questions here.]

5,000 /10,000 /50,000 /100,000 /200,000 yuan

FI SOCIAL INSURANCE QUESTION FOR THE SELF-EMPLOYED

PROCEDURE : If R has not processed retirement(FB011 = 2), ask: FI001-FI011 . （ FB011 = 2） FI001-FI011

[Show Card 22]

FI001 Next are questions about your social insurance. Are you covered by pension insurance, health insurance, unemployment insurance, worker s injury insurance or maternity insurance through your own self-employment? (Choose all that apply) （ ）

1. Pension → skip to FI005 FI005
2. Health insurance → skip to FI005 FI005
3. Unemployment insurance → skip to FI005 FI005
4. Workers injury insurance → skip to FI005 FI005
5. Maternity Insurance → skip to FI005 FI005
6. None

FI002 Why are you not covered by the above mentioned social insurance through self-employment? （ ） ?

1. It is not possible for the self-employed to participate in social insurance in my local area → skip to FI012 FI012
2. Social insurance is available to the self-employed but I do not participate → skip to FI012 FI012
3. I have social insurance from elsewhere
4. I have passed retirement age → skip to FI012 FI012
5. I don t know how to participate in social insurance → skip to FI012 FI012
6. Other → Skip to FI012 → skip to FI012 FI012

FI003 From where do you have the above mentioned social insurance? ()

1. I am covered through another work unit
2. I contribute through job service center → skip to FI005 FI005

FI004 You contribute through another work unit, why? ()

1. I was laid-off by that work unit but employment contract is not terminated
2. I am on leave from that employer
3. I keep my position but do not receive pay from that work unit
4. A friend in that work unit is helping me this way 5. Other

**PROCEDURE** : If R has social insurance, either from self-employment or by job service center, ask: FI005–FI011 . FI005–FI011

FI005 How much is your own contribution to the above mentioned social insurance last year? () ____ Yuan

Soft Check: Prompt if FI005 = 0 or FI005>FI010/2.

**PROCEDURE** :<br>If FI009 = 2 skip FI006 . FI009= 2 FI006

FI006 Is the income you told us about earlier net after paying for the above benefits?

1. Yes
2. No

FI007 On what income base is the contribution to the above mentioned social insurance determined? ( ) ____ Yuan/month /

**PROCEDURE** : If R participates in social insurance through another work unit (FI003 = 1), askFI008–FI010 . (FI003 = 1) FI008–FI010

FI008 Do you know how much you or that work unit contributes to the above benefits? ( )

1. I know the amount by myself and the work unit → skip to FI009 FI009
2. I know the amount by myself but not the work unit → skip to FI010 FI010
3. I do not know either my contribution or the work units contribution at all. → skip to FI011 FI011

FI009 How much is work units contribution in total? __ Yuan/month /

Soft Check: Prompt for Verification if Contribution is Greater than Monthly Earnings, e.g., FI009 >FH010

FI010 How much is your own contribution to pension in total? ( ) ____ Yuan/month /

Soft Check: Prompt for Verification if Contribution is Greater than Monthly Earnings, e.g., FI010 > FH010

FI011 Do you expect to process retirement at this business/organization?

1. Yes
2. No

FI012 At what age do you plan to stop working? Stopping work in this context shall refer to having stopped all income-related activities and unpaid family business and having no intention of engaging in anything more serious than small pastime work. ?____ 0…120 Years old

F1

[IWER: Please tell me the approximate age. Mark 0 if you plan to keep working until you are physically able. 0] Soft Check: Prompt for correction if current age is greater than response, e.g. (Interview Year+ Interview month/12) – (CV009_a+CV009_b/12) > FI012

FJ SIDE JOB (EMPLOYED OR SELF- EMPLOYED) ( )

If FC019 = 1(more than one job), proceed with the following section. FC019 = 1( )

FJ001 How many jobs do you currently hold, excluding your main job? __ 1…20

FJ002 How many hours a week do you work on average at your side job(s), not considering your main job? ____ 0...168 Hours per week /

FJ003 What is the average monthly income or wage that you get from side job(s) other than your main job? ____ Yuan per month /

FJ004 [IWER: IfR is unwilling to answer or does not remember, ask unfolding bracket questions here. :] 500 /1,000 /2,500 /5,000 /10,000 yuan

FK UNEMPLOYMENT AND JOB SEARCH ACTIVITIES

PROCEDURE :

- If new R is not working but has worked before (FA007= 1 or FA008= 2), answer FK. FK
- If old R is not working (XF1= 2) but worked before (FA006_w2_5> 0 or ZF13= 1 or ZF14= 2), answer FK FK

FK001 Next are some questions about circumstances about your non-employment and job search activities. In what month and year did you last work? ____ 1900...2013 (**FK001_1**) Year __0...12 (**FK001_2**) Month

[IWER: Mark the year using four digits. Take down the month as its actual number. For example, write January as 1 not 01, December as 12. If do not remember month, fill '0. : 4 1 1 01,12 12**O** **]**

FK002 Did you search for a new job during the last month? 1. Yes

2. No → Skip to FL001 FL001

FK003 At what age do you plan to stop working? Stopping work in this context shall refer to having stopped all income-related activities and unpaid family business and having no intention of engaging in anything more serious than small pastime work. ? ___ 0...120 Years old

[IWER: Please tell me the approximate age. Mark 0 if you plan on working until you are physically capable. 0]

FL LAST JOB

FL001 BRANCHPOINT :

For Old R: If old Rs last job is not missing (ZF22_1 = 1), skip FL. FL If old Rs last job is missing (ZF22_1 = 2), please skip to procedure before FL001. FL001

For new R: Please skip to procedure before FL001. FL001

PROCEDURE :

RESPONDENT NOT CURRENTLY WORKING Ask if FA007 = 1 or FA008 = 2

[IWERThe next questions are about the last main job you had, which could be farming, earning a wage, running your own business or working for unpaid family business. It does not include doing your own housework or doing activities without pay, such as voluntary work. If you have more than one job, we are interested in the job at which you worked the longest hours. Were interested in your situation near the termination of this job.]

FL001 Did you work for someone else(including work for unpaid family business), were you self-employed, did you farm, or were you otherwise employed? ( )

1. Employed ( )
2. Self-employed ( )
3. Unpaid family business
4. Farming ( )

FL002 In which year and month did you start working at that job? ____ 1900...2013 (**FL002_1**) Year __0...12 (**FL002_2**) Month

[IWER: Mark the year using four digits. Take down the month as its actual number. For example, write January as 1 not 01, December as 12. If do not remember month, fill ‘0. : 4 1 1 01,12 12 0] [Soft Check: Prompt for Verification/Clarification if the Respondent was Less than 16 at time of starting this job, e.g. Prompt if (FL002_1+FL002_2 /12)-(CV009_a+CV009_b/12)<16] [Replace 16 in the above check with FB001 . FL002_1 < FB001_2 or FL002_1 < (CV009_a + FB001_1)]

FL003 In which year and month did you stop working at that job? ____ 1900…2013 (**FL003_1**) Year __0…12 (**FL003_2**) Month

[IWER: Mark the year using four digits. Take down the month as its actual number. For example, write January as 1 not 01, December as 12. If do not remember month, fill '0. : 4 1 1 01,12 12 0] [Soft Check: Prompt for Verification/clarification if End Date is Before the Start Date, e.g. FL003_1 < FL002_1]

**FL004 BRANCHPOINT** :<br>For old R, if FL003_1<Last IW Year （ZIWYear） or FL003_1=Last IW Year （ZIWYear） and FL003_2<Last IW Month （ZIWMonth）, then skip to Section FM. FL003_1< （ZIWYear） FL003_1= （ZIWYear） FL003_2< （ZIWMonth） FM

FL004 Where was the job located?

1. The same as permanent address
2. Another village/neighborhood in permanent addresss county/city/district /// _ (**FL004_1**) village/neighborhood /
3. Other ____(**FL004_2**) province_city_county/city/district _ _ // _(**FL004_3**) village/neighborhood /
4. Abroad

[IWER:]

**PROCEDURE** :<br>If FL001 = 4 Skip to FL020 FL001 = 4FL020<br>If non-farmer (FL001= 1/2/3), ask FL005 to FL008 . （FL001= 1/2/3） FL005 to FL008

FL005 What was the name of your workplace/employer? Please state specifically the name of your company or business. () __________________ (**FL005_1**) （1 2）, _______ (**FL005_2**) （1 2）

FL006 What kind of business or industry was it—that is, what did they make or do at the place where you worked? ___________ （1 2）

[IWER: Type of business]

FL007 Is this employer still in existence?

1. Yes
2. No

FL008 How many hours a week did you usually work [for this employer/in this business]?
[___0...168 Hours per week /
[Soft Check: Prompt for Verification if FL008 > 80]

**PROCEDURE** :<br>Ask only if employed. （FL001= 1）

FL009 What were the monthly wages, bonuses, and subsidies from this jobbefore you stopped working at this job? （ ） ______Yuan
[IWER: Mark 0 if there is no net income, and mark 999997 if running a deficit. 0 999997.] [Soft Check: Soft Check: Prompt for Verification if FL009 < 100]

FL010 [IWER: IfR is unwilling to answer or does not remember, ask unfolding bracket questions here. :] 500 /1,000 /2,500 /5,000 /10,000 yuan

FL011 What was the value of other bonuses not paid with regular wages each year? （ ） ___Yuan

FL011_bracket [IWER: If R is unwilling to answer or does not remember, ask unfolding bracket questions here. :]
500 /1,000 /2,500 /5,000 /10,000 yuan

**PROCEDURE** :<br>Ask FL012 if self-employed (FL001 = 2). （FL001 = 2） FL012 Otherwise skip to FL013. FL013

FL012 Do you have employees?
1. Self-employed with employees
2. Self-employed without employees
3. Family business worker without pay

**PROCEDURE** :<br>Ask FL013－FL017 only if employed (FL001 = 1). FL013－FL017 （FL001 = 1）<br>Otherwise skip to FL020. FL020

FL013 Were you a regular worker, a temporary worker, or a casual worker?

1. Regular wage worker
2. Contract worker

3. Temporary wage worker
4. Casual wage worker

FL014 Did you work for the government, institution, firm, NGO, individual farmer or a resident household? （ ）

1. Government
2. Institutions → Skip to FL017 FL017
3. NGO （） → Skip to FL017 FL017 4. Firm Skip to → FL016 FL016
5. Individual firm → Skip to FL017 FL017
6. Individual farmer → Skip to FL017 FL017
7. Individual household → Skip to FL017 FL017
8. Other → Skip to FL017 FL017

PROCEDURE :
If government employee (FL014= 1), ask FL015. FL015

FL015 Were you a civil servant?

1. Yes
2. No

PROCEDURE :
If firm (FL014= 1), ask FL016. (FL014= 1)FL016
Otherwise skip to FL017. FL017

[Show Card 20]

FL016 What was the ownership type of the business? 1. 100% State owned firm

2. State-controlled firm
3. 100% Collective-owned firm
4. Collective-controlled firm
5. 100% Private firm /
6. Private-controlled firm
7. 100% foreign-owned
8. Joint venture
9. Other joint-ownership
10. Other

FL017 What sort of work did you do? ______**(FL017_1)**（ 1 2） ____**FL017_2)**（ 1 2）
[IWER: Ask the specific work.]

PROCEDURE :
IfR has processed retirement FB011= 1, ask FL018. FL018

FL018 Is this the business/organization where you processed retirement?
1. Yes → Skip to FL021 FL021
2. No → Skip to FL020 FL020

PROCEDURE :
If R has not processed retirement FB011= 2, ask FL019. FL019

FL019 Do you expect to process retirement from this business/organization?
1. Yes
2. No

FL020 Why did you leave that employer?
[IWER: Do not probe but check all that apply.]
1. Business closed / (**FL020s1**)
2. Quit (**FL020s2**)
3. I was laid off (**FL020s3**)
4. I was fired (**FL020s4**)
5. I went to school (**FL020s5**)
6. I went abroad (**FL020s6**)
7. I stopped working for health reasons (**FL020s7**) 8. I stopped working for family reasons (**FL020s8**) 9. I was transferred to another job (**FL020s9**)
10. I was sent down to the countryside to do manual labor (**FL020s10**) 11. I started working off-farm locally (**FL020s11**)
12. I went to work away from home (**FL020s12**)
13. Better job in local area (**FL020s13**)
14. Better job in another location (**FL020s14**)
15. I retired (**FL020s15**)
16. Other (**FL020s16**)

FL021 Did you receive any payments other than the legal retirement allowance upon leaving your last job? (For example, condolence paymentworkers compensation, etc.) ()
1. Yes
2. No

F1

PROCEDURE :
If FL021 = 1, ask FL022. FL021= 1 FL022

FL022 How much was the compensation and for how many years of work?____Yuan ____years ?_____ (**FL022_1**) ____0…120 (**FL022_2**)

FL022_bracket [IWER: If R is unwilling to answer or does not remember, ask unfolding bracket questions here.]:
1000 /2000 /5000 /10,000 /20,000 yuan

FM RETIREMENT

PROCEDURE :
If R has processed retirement [FB011 = 1 or FB012 = 1 or ZF15 = 1 or ZF16 = 1], ask FM001 – FM059 . [FB011 = 1 ZF15 = 1] [FB012 = 1 ZF16 = 1] / /FM001 –FM059

FM001_1 BRANCHPOINT :
（ZF16= 1） ZF25_1 ZF25_2 ZF25_3
ZF25_1= 1FM004 ZF25_2= 1 FM005 ZF25_3= 1
FM007 FM001_2 BRANCHPOINT
[/] （ZF25_4= 1） ZF25_1
ZF25_5 ZF25_6ZF25_1= 1 FM004 ZF25_5= 1FM014 ZF25_6= 1 FM016 - FM017
FM001_2 BRANCHPOINT
（ZF25_8= 1）ZF25_1ZF25_9ZF25_10 ZF25_11 ZF25_1= 1FM004 ZF25_9= 1 FM025ZF25_10= 1 FM027 ZF25_11= 1 FM028-FM029 FM001_2 BRANCHPOINT

FM001_2 BRANCHPOINT :

If old R had completed receding position procedure prior to the last IW (ZF16= 1), or had completed regular retirement in the last IW (ZF16= 1), skip to FM052 BRANCHPOINT. FM052 BRANCHPOINT.

If old R had completed internal retirement but not yet regular retirement prior to the last IW （ZF23= 1）, skip to FM037_w2 BRANCHPOINT. FM037_w2 BRANCHPOINT

If old R had not completed retirment procedure or receding position procedure prior to the last IW (ZF24= 1), continue with FM001 . FM001 .
All new R continues with FM001 FM001

FM001 Which of the following is the work unit that processed your [preload: retirement /receding position] [/]
[IWER: If R has no work unit (no answer to FD003), please choose (3) None of the above. （3）]

1. Current work unit [for new R: preload FD003; for old R: ZF8 if FA006_w2_2a= 1 & FA006_w2_2a_3= 1, orFA006_w2_2a_1= 1 if FA006_w2_2a= 2 & FA006_w2_2a_3= 1, or FM003 if FA006_w2_2a_3= 2] → Skip to procedure before FM005 FM005
2. Last work unit [preload FL005 or ZF25] FL005 [FL005 or ZF25] → Skip to procedure before FM005 FM005 3. None of the above

PROCEDURE :
If answer to FM001is (3) None of the above, then ask FM002-FM004 : FM001 (3) FM002-FM004

FM002 What is the name of the employer that processed your [preload: retirement /receding position]? [/] ________

FM003 What was the type of your work unit at [preload: retirement /receding position]? [/]

1. Government
2. Institutions
3. NGO （）
4. Firm
5. Individual firm
6. Farmer
7. Individual household
8. Other

FM004 Where is this work unit located?

1. The same as permanent address
2. Another village/neighborhood in permanent addresss county/city/district /// _ (**FB004_1**) village/neighborhood /
3. Other ____(**FB004_2**) province_city_county/city/district _ _ // _(**FB004_3**) village/neighborhood /
4. Abroad

[IWER:]

**PROCEDURE** :<br>If FB012= 1 (Receding), ask FM005-FM009 FB012= 1 （） FM005- FM009

FM005 In what month and year did you recede from your position? ____ (**FM005_1**) 1900…2013 year __ (**FM005_2**) 0…12 month

[IWERMark the year using four digits. Take down the month as its actual number. For example, write January as 1 not 01, December as 12. If do not remember month, fill ‘0. : 4 1 1 01,12 12**O** **]**

FM006 What was the main reason you receded from your position?

1. Due to poor health, I couldnt continue my work any more, at the same time I wasnt eligible for retirement
2. Years of eligible work are less than threeand time of stopping work due to diseases or injures not related to work are more than one year
3. Im recruit worker within 6 months, but I had serious chronic disease once and cant tstick to work any more
4. I receded from my position voluntary
5. Reach retirement age, but not eligible working age. 6. Other

FM007 Your pre-receding total salary was____ Yuan a month (including basic wage, bonus, et. al). ____ /,

FM007_bracket [IWER: If R is unwilling to answer or does not remember, ask unfolding bracket questions here.]:

500 /1,000 /2,000 /3,500 /5,000 yuan

FM008 Did you receive any payments for leaving your job

1. Yes
2. No

PROCEDURE :
If FM008 = 1, ask FM009. FM008 = 1 FM009

FM009 How much was the compensation?_____ Yuan ?_____

FM009_bracket [IWER: If R is unwilling to answer or does not remember, ask unfolding bracket questions here.]:
500 /1,000 /2,000 /3,500 /5,000 yuan

PROCEDURE :
If FB011 = 1 (Retirement), askFM011-FM041. FB011= 1 ( )FM011- FM041
Otherwise skip to FM042, FM042

FM011 Was your retirement normal retirement; early retirement; or internal retirement initially, followed by regular retirement? ,
1. Normal retirement
2. Early retirement
3. Internal retirement first, then regular retirement
4. Internal retirement, but not yet regular retirement

FM012 Did you retire as a worker or as a cadre? 1. Worker
2. Cadre

PROCEDURE :
If year of first job was before 1952 (compute from FB001), then ask FM013. 1952 FM013

FM013 Are you an ordinary retiree or revolutionary retiree? 1. Ordinary retiree
2. Revolutionary retiree
F1

PROCEDURE :
If FM011 = 1 or FM011 = 2 , ask FM014-FM024. FM011 = 1/2 FM014-FM024

FM014 In what month and year did you take [preload: normal/early] retirement? [/] ____ 1900…2013 (**FM014_1**) year __0…12 (**FM014_2**) month
[IWERMark the year using four digits. Take down the month as its actual number.

For example, write January as 1 not 01, December as 12. If do not remember month, fill ‘0. : 4 1 1 01,12 12**O**]

Soft Check: Prompt for Verification/Correction if Age of Early Retirement is Young, e.g. Prompt if ((FM014_ 1 +FM014_2 /12)-(CV009_a+CV009_b/12) < 45 & CV004==2) | ((FM014_ 1 +FM014_ 1 /12)-(CV009_a+CV009_b/12) <50 & CV004== 1)

PROCEDURE :
Ask FM015 if FM011 = 2 (early retirement). FM015

FM015 What was the main reason you processed early retirement?

1. I have 30 years job experience, which is enough for early retirement. 30
2. My work unit belonged to the category of high-risk and hard manual labor and thus was eligible for offering early retirement
3. My work unit was restructuring/bankrupt, so it offered early retirement
4. Due to poor health
5. Due to family reason
6. Other

FM016 Your pre-retirement salary was____ Yuan a month (including bonus and subsidy, et. al). ____ /

FM017 [IWER: If R is unwilling to answer or does not remember, ask unfolding bracket questions here.]: 500 /1,000 /2,500 /5,000 /10,000 yuan

FM024 How many years of eligible work did you have at the time of retirement? ____ 0.00...100.00 Years

PROCEDURE :
Ask FM025-FM029 if FM011 = 3 or FM011 = 4 (internal retirement). FM011=3/4 FM025-FM029
Otherwise skip to FM042, FM042

FM025 In what month and year did you take internal retirement? ____ 1900...2013 (**FM025_1**) year __0...12 (**FM025_2**) month

[IWERMark the year using four digits. Take down the month as its actual number. For example, write January as 1 not 01, December as 12. If do not remember month, fill ‘0. : 4 1 1 01,12 12**O**]

Soft Check: Prompt for Verification/Correction if Age of Retirement is Young, e.g. Prompt if (FM030_ 1 +FM030_2 /12)-(CV009_a+CV009_b/12) <45 & CV004==2) | ((FM030_ 1 + FM030_2 /12)-(CV009_a+CV009_b/12) <50 & CV004== 1)

FM026 What was the main reason you processed internal retirement?

1. 5 years less than the legal retirement age 5
2. My work unit was restructuring/bankrupt
3. Due to poor health
4. Due to family reason
5. Other

FM027 Your pre-internal retirement salary was ____ Yuan a month everything included. ?
/

FM027_bracket [IWER: If R is unwilling to answer or does not remember, ask unfolding bracket questions here.]:

500 /1,000 /2,000 /3,500 /5,000 yuan

FM028 How much was the internal retirement wage (everything included) when you processed internal retirement? ___ /

FM029 [IWER: If R is unwilling to answer or does not remember, ask unfolding bracket questions here.]: 500 /1,000 /2,000 /3,500 /5,000 yuan

**PROCEDURE** :
Ask FM030–FM036 if FM011 = 3. FM011= 3FM030–FM036

FM030 In what month and year did you process formal retirement? ____ 1900...2013 (**FM030_ 1**) year __0...12 (**FM030_2**) month

[IWERMark the year using four digits. Take down the month as its actual number. For example, write January as 1 not 01, December as 12. If do not remember month, fill '0. : 4 1 1 01,12 12**O** **]**

Soft Check: Prompt for Verification/Correction if Age of Retirement is Young, e.g. Prompt if ((FM030_ 1 +FM030_2 /12)-(CV009_a+CV009_b/12) <45 & CV004==2) | ((FM030_ 1 +FM030_2 /12)-(CV009_a+CV009_b/12) <50 & CV004== 1)

FM036 How many years of eligible work did you have at the time of formal retirement? ___ 0.00...100.00 Years

PROCEDURE :
Skip to FM042 FM042

FM037_w2 BRANCHPOINT :
If R had completed internal retirement but not yet regular retirement prio to the last IW (ZF23= 1), then ask FM037_w2- FM040_w2. FM037_w2FM040_w2.

FM037_w2 Did you proceed formal retirement? 1. Yes
2. No → Skip to FM037 FM037

FM038_w2 In what month and year did you process formal retirement? ____
1900…2013 (**FM038_w2_1_1**) year __0…12 (**FM038_w2_1_2**) month

[IWERMark the year using four digits. Take down the month as its actual number. For example, write January as 1 not 01, December as 12. If do not remember month, fill '0. : 4 1 1 01,12 12**O**]

Soft Check: Prompt for Verification/Correction if Age of Retirement is Young, e.g. Prompt if ((FM030_ 1 +FM030_2 /12)-(CV009_a+CV009_b/12) <45 & CV004==2) | ((FM030_ 1 +FM030_2 /12)-(CV009_a+CV009_b/12) <50 & CV004==1)

FM040_w2 How many years of eligible work did you have at the time of formal retirement?
0.00…100.00 Years

Skip to FM042 FM042

PROCEDURE :
Ask FM037–FM040 if FM011 = 4 or FM037_w2 = 2. FM011 = 4 FM037_w2 = 2FM037–FM040
Otherwise Skip to FM042. FM042

FM037 In what month and year are you going to process formal retirement? ____
1900…2013 (**FM037_1**) year __0…12 (**FM030_2**) month

[IWERMark the year using four digits. Take down the month as its actual number. For example, write January as 1 not 01, December as 12. If do not remember month, fill '0. : 4 1 1 01,12 12**O**]

Soft Check: Verify if Age of Respondent will be outside the legal retirement range, e.g., Prompt for verification if ((FM037_ 1 + FM037_2/12) - (CV009_a + CV009_b/12) < 50 | (FM037_ 1 + FM037_2/12) - (CV009_a + CV009_b/12) > 55) & CV004 == 2) | (FM037_ 1 + FM037_2 /12) - (CV009_a + CV009_b/12) < 55 | (FM037_ 1 + FM037_2/12) - (CV009_a + CV009_b/12) > 60) & CV004 == 1)

FM040 How many years of eligible work will you have at the time of retirement? ____
0.00…100.00 Years

FM041 How many years of eligible work do you currently have? __0.00…100.00 Years
[FM041 > FM040]

**PROCEDURE** :
Skip (FM042–FM051) if FM037_w2 = 2. FM037_w2 = 2FM042–FM051

FM042 Did you have a spouse when you processed [preload: normal retirement / early retirement / internal retirement / receding position]?
[/// (FM011 FM037_w2 = 1)]

1. Yes
2. No

FM043 How was your health at the time of your [preload: normal retirement / early retirement / internal retirement / receding position], excellent, very good, good, fair or poor?
[///] ?

1. Excellent
2. Very good
3. Good
4. Fair
5. Poor

**PROCEDURE** :
Skip FM044–FM046 if FM042 = 2. FM042 = 2 FM044–FM046

FM044 Had your spouse already processed retirement when you processed [preload: normal retirement / early retirement / internal retirement / receding position]? [///]

1. Yes
2. No

FM045 What kind of economic activities was your spouse engaged in at the time of your [preload: retirement / receding position]? [/]

1. Employed by another person or company and received a wage
2. Ran own business
3. Non-employed and looking for a job
4. Non-employed and not looking for a job or only doing household work ,
5. Farming

FM046 How was your spouses health at the time of your [preload: normal retirement / early retirement / internal retirement / receding position], excellent, very good, good, fair or poor? [///] ?

1. Excellent
2. Very good
3. Good
4. Fair
5. Poor

FM047 Was your father alive at the time of your [preload: normal retirement / early retirement / internal retirement / receding position]? [///]

1. Yes
2. No → Skip to FM049 FM049

FM048 How about the health of your father at the time of your [preload: normal retirement / early retirement / internal retirement / receding position], excellent, very good, good, fair or poor? [///] ?

1. Excellent
2. Very good
3. Good
4. Fair
5. Poor

FM049 Did your mother alive when your [preload: normal retirement / early retirement / internal retirement / receding position]? [///]

1. Yes
2. No → Skip to FM051 FM051

FM050 How about the health of your mother at the time of your [preload: normal retirement / early retirement / internal retirement / receding position], excellent, very good, good, fair or poor? [///] ?

1. Excellent
2. Very good
3. Good
4. Fair
5. Poor

FM051 How many grandchildren below age 6 did you have at the time of your [preload: normal retirement / early retirement / internal retirement / receding position]? [///]
0…50 persons
[IWER: if none, fill '0. 0]

FM052 BRANCHPOINT:
For old R who had processed receding position procedure in the last IW (ZF16 = 1) or had processed internal but not regular retirement in the last IW (ZF23 = 1), or had processed regular retirement in the last IW (ZF26 = 1): if old R had not worked after processing retirement/receding position procedure until the last IW (ZF26 = 1) and is currently not working (XF1 = 2), ask FM052_w2; if old R had not worked after processing retirement/receding position procedure until the last IW (ZF27 = 1) and is currently working (XF1 = 1), ask FM053. / FM052_w2/ FM053FM052_w2 New R If R is currently not working (FA001 = 2 & FA002 = 2 & FA003 = 2), ask FM052. （ FA001 = 2 & FA002 = 2 & FA003 = 2 ） FM052If FA052 = 1 or If R is currently working, ask FM053. （FA052 = 1 or FA001 = 1 or FA002 = 1 or FA003 = 1）FM053

FM052 Did you work after you processed [preload: normal retirement / early retirement / internal retirement / receding position]? We consider any of the following activities to be work: agricultural work, earn a wage, run your own business and unpaid family business work, et. al. Work does not include doing your own housework or doing activities without pay, such as voluntary work. [///]

1. Yes → Skip to FM053 FM053
2. No → Skip to FM054 FM054

FM052_w2 Did you work in the last two years? We consider any of the following activities to be work: agricultural work, earn a wage, run your own business and unpaid family business work, et.al. Work does not include doing your own householdwork or doing activities without pay, such as voluntary work.

1. Yes
2. No → Skip to FM054 FM054

FM053 After you processed [preload: normal retirement / early retirement / internal retirement / receding position] How long did you start to work again? [

///] ____0.00…100.00 Years (allow for decimal points)

PROCEDURE :
Skip to FN001_w2 FN001_w2

FM054 Are you currently engaged in paid small pastime work?
1. Yes
2. No → Skip to FN001_w2 FN001_w2

FM055 What kind of pastime job are you engaged in? ?_______________
[IWER: Ask the specific work.]

FM056 When did you start this job? ____ 1900…2013 (**FM056_1**) year __0…12 (**FM056_2**) month
[IWERMark the year using four digits. Take down the month as its actual number. For example, write January as 1 not 01, December as 12. If do not remember month, fill '0. : 4 1 1 0112 12**O** **]**

FM057 How many days per week do you usually work for your pastime job? An average of __ days per week __0…7

FM058 How many hours per week do you usually work at your pastime job? An average of_____ hours per week ____0.00…168.00

FM059 What is your monthly income from the pastime work? ____ YUAN
[IWER: Mark 0 if there is no net income, and mark 999997 if running a deficit. 0 999997]

FM059_bracket [IWER: If R is unwilling to answer or does not remember, ask unfolding bracket questions here.]:
500 /1,000 /2,000 /3,500 /5,000 yuan

FN PENSION INSURANCE

[Intro: Next we ll ask you some questions about your pension insurance. It s important to assess existing pension policy and revise it in the future. :]

[Show Card 23]

FN001_w2 Are you currently participating in or receiving at least one kind of pension as followings? Pension here refers to income from such pension programs as supplemental pension insurance of the firms, residents pension insurance, rural pension insurance, Urban residents pension and commercial pension insurance, and pension subsidy for the oldest old and so on? (choose all that apply) / ( )

[IWER: Read out all the choices.]

1. Pension program of the government or institutions 2. Basic pension of the firms
3. Supplemental pension insurance of the firm ( ) 4. Commercial pension
5. Life insurance
6. Rural pension ( )
7. Residents pension
8. Urban residents pension
9. New Rural Social Pension Insurance ( )
10. Endowment insurance for the land-losing farmers ( ) 11. Old age pension allowance ( )
12. Other
13. None

[F1 (1) (2) (3) (4) (5) ( ) 1992 (6) (7) (8) ( ) (9) ( ) 60 ,]

Part 1 PENSION PROGRAM OF THE GOVERNMENT AND INSTITUTIONS OR BASIC PENSION OF THE FIRMS

Ask all R

FN002_w2 Are you currently receiving pension as follows (Check all that apply)? / ()

1. Pension program of the government ( )
2. Pension program of institutions
3. Basic pension program of the firms
4. None → Skip to FN006_w2 FN006_w2

**PROCEDURE** :
FN002_w2FN003_w2-FN005_w2.

FN003_w2 In what month and year did you start to receive you pension benefits? () ____ 1900…2013 (**FN003_w2_1**) year __0…12 (**FN003_w2_2**) month

[IWERMark the year using four digits. Take down the month as its actual number. For example, write January as 1 not 01, December as 12. If do not remember month, fill '0. : 4 1 1 0112 12**O**]

FN004_w2 How much were the benefits (including subsidy) when you retired? / () ____ Yuan per month /

FN005_w2 What is your monthly pension (including subsidy)? / ()_____ Yuan

[Soft Check: Verify if monthly benefits are low or high, e.g., prompt if FN005_w2 <200 yuan/month]

**PROCEDURE** :
Skip to FN030_w2 FN030_w2

FN006_w2 Are you currently enrolled in pension program of the government and institutions or basic pension of the firms?

1. Yes, pension program of the government and institutions
2. Yes, basic pension insurance of the firms 3. Yes, both of them. /
4. No / → Skip to FN022_w2 FN022_w2

[F1（1） （2）]

PROCEDURE :
FN006_w2 = 3FN007_w2－FN014_w2.

FN007_w2 From which of the following work units did you get the pension insurance you just told us?
[preloadpension program of government and institutions if FN006_w2 = 1; or preload Basic pension insurance of the firms if FN006_w2 = 2] [FN006_w2 = 1, FN006_w2 = 2,]
[IWER: Choose (4) None of the above if the respondent has no work unit （4）]

1. Current work unit [for new R: preload FD003; for old R: ZF8 if FA006_w2_2a = 1 & FA006_w2_2a_3= 1, or FA006_w2_2a_1 if FA006_w2_2a= 2 & FA006_w2_3= 1, or FD003 if FA006_w2_2a_3= 2] → Skip to FN011_w2 FN011_w2
2. Last work unit [preload FL005 or ZF25] FL005 [FL005 or ZF25] → Skip to FN011_w2 FN011_w2
3. The work unit that processed retirement for the respondent [preload FM002] FM002 [FM002] → Skip to FN011_w2 FN011_w2
4. None of the above

PROCEDURE :
If answer to FN007_w2 = 4 (None of the above), then ask FN008_w2 – FN010_w2.
FN007_w2 = 4 () FN008_w2 – FN010_w2.

FN008_w2 What is the name of the unit that provides you this pension insurance? /? ___

FN009_w2 What was the type of the unit that provides you the pension insurance?
1. Government
2. Institutions
3. NGO （）
4. Firm
5. Individual
6. Other

FN010_w2 Where is this work unit located?

1. The same as permanent address
2. Another village/neighborhood in permanent addresss county/city/district /// _ (**FN010_ 1**) village/neighborhood /
3. Other ____ (**FN010_2**) province_city_county/city/district _ _ // _ (**FN010_3**) village/neighborhood /
4. Abroad

[IWER:]

**PROCEDURE** :
FN011_w2

FN011_w2 In what month and year did you start to participate in the basic pension insurance of the firms through this work unit? ____ 1900...2013 (**FN011_w2_ 1**) year __0...12 (**FN011_w2_2**) month

[IWER: Mark the year using four digits. Take down the month as its actual number. For example, write January as 1 not 01, December as 12. If do not remember month, fill ‘0. : 4 1 1 0112 12**O** **]**

FN012_w2 Do you need to pay the premium by yourself?

1. Yes
2. No → Skip to FN015_w2 FN015_w2

FN013_w2 How much is the premium you paid? ____ (**FN013_w2_ 1**) Yuan per month / Or 0.00...100.00 (**FN013_w2_2**) percent

FN014_w2 How much the premium your unit paid? ____ (**FN014_w2_ 1**) Yuan per month / Or ____0.00...100.00 (**FN014_w2_2**)

**PROCEDURE** :
Skip FN015_w2 -FN016_w2 if FN006_w2 = 1. FN006_w2 = 1FN015_w2 - FN016_w2

FN015_w2 Before the above mentioned time, have you ever participated in basic pension insurance of the firms through other work unit for more than ten years? 10

1. Yes
2. No → Skip to FN017_w2 FN017_w2

FN016_w2 Among all of work units that you have participated in basic pension insurance of the firms for more than 10 years, in which province was the lastest work unit located? 10 ____ Province //

FN017_w2 For how many years altogether have you been included in this program? [Include years with other employers if the same plan.] （ ） [ ]____0.00...100.00 Years

FN018_w2 For how many years altogether will you have been included in this program when you retire? [Include years with other employers if the same plan.] （） [ ]____ 0.00...100.00 Years

FN019_w2 Will this be enough years to receive pension?

1. Yes → Skip to FN021_w2 FN021_w2
2. No

FN020_w2 What do you plan to do?

1. I will pay the remaining premiums all in one payment at retirement to qualify for pension
2. I will receive a one-time payment at retirement and not get pension → Skip to FN030_w2 FN030_w2
3. I will not receive pension → Skip to FN030_w2 FN030_w2

PROCEDURE：

If R answered yes to pension [FN019_w2 = 1 or FN020_w2 = 1], ask FN021_w2 - FN021_w2_bracket, then skip to FN030_w2. [FN019_w2= 1 or FN020_w2= 1] FN021_w2 - FN021_w2_bracket FN030_w2

FN021_w2 About how much do you expect your benefits to be? (as a percentage of your pay at retirement, or as an amount per month or year?) ____（**FN021_w2_1**）Yuan per month / Or ____0.00..100.00（**FN021_w2_2**）

FN021_w2_bracket [IWER: IfR is unwilling to answer or does not remember, ask unfolding bracket questions here.]:

500 /1,000 /2,000 /3,500 /5,000 yuan

FN022_w2 Are you ever enrolled in pension program of the government and institutions or basic pension of the firms?

1. Yes, pension program of the government and institutions
2. Yes, basic pension insurance of the firms

3. Yes
4. No → Skip to FN030_w2 FN030_w2

F1

FN023_w2 From which of the following work units did you get the pension insurance you just told us[preload pension program of government and institutions

Preload pension program of government and institutions if FN022_w2 = 1; or preload Basic pension insurance of the firmsif FN022_w2 = 2; FN022_w2 = 1, FN022_w2 = 2,]

[IWER: Choose (4) None of the above if the respondent has no work unit （4）]

1. Current work unit [for new R: preload FD003; for old R: ZF8 if FA006_w2_2a = 1 & FA006_w2_3 = 1, or FA006_w2_2a_1 if FA006_w2_2a = 2 & FA006_w2_3 = 1, or FD003 if FA006_w2_2a_3 = 2] → Skip to FN027_w2 FN027_w2
2. Last work unit [preload FL005 or ZF25] FL005 [FL005 or ZF25] → Skip to FN027_w2 FN027_w2
3. The work unit that processed retirement for the respondent [preload FM002] FM002 [FM002] → Skip to FN027_w2FN027_w2
4. None of the above

PROCEDURE :

If answer to FN023_w2 = 4 (None of the above), then ask FN024_w2 – FN026_w2.
FN023_w2 = 4 () FN024_w2 – FN026_w2

FN024_w2 What is the name of the unit that provides you this pension insurance? ? ____

FN025_w2 What was the type of the unit that provides you the pension insurance?

1. Government
2. Institutions
3. NGO （）
4. Firm
5. Individual
6. Other

FN026_w2 Where is this work unit located? 1. The same as permanent address

2. Another village/neighborhood in permanent addresss county/city/district /// _ (**FN026_w2_1**) village/neighborhood /

3. Other ____ (**FN026_w2_2**) province_city_county/city/district _ _ // ____ (**FN026_w2_3**) village/neighborhood /

4. Abroad

[IWER:]

FN027_w2 In what month and year did you start to participate in the basic pension insurance of the firms through this work unit? ____ 1900...2013 (**FN027_w2_1**) year __0...12 (**FN027_w2_2**) month

[IWER: Mark the year using four digits. Take down the month as its actual number. For example, write January as 1 not 01, December as 12. If do not remember month, fill ‘0. : 4 1 1 0112 12**O** **]**

FN028_w2 In what month and year did you start to participate in the basic pension insurance of the firms through this work unit? ____ 1900...2013 (**FN028_w2_1**) year __0...12 (**FN028_w2_2**) month

[IWER: Mark the year using four digits. Take down the month as its actual number. For example, write January as 1 not 01, December as 12. If do not remember month, fill ‘0. : 4 1 1 0112 12**O** **]**

FN029_w2 What was the reason for you to stop participating in this pension? （Multiple choice ）

1. Business closed
2. I changed job/I was fired /
3. I moved to other city, pension can not be transferred 4. I thought it was not worth and exited

Part 2 SUPPLEMENT PENSION INSURANCE OF THE FIRM

FN030_w2 Did you participate in supplemental pension insurance of the firm or receive the pension?

1. Yes, but I dont receive it
2. Yes, I receive it
3. No → Skip to FN043_w2 FN043_w2

FN031_w2 Where did you participate in the insurance? ____ (**FN031_w2_1**) Province // _ (**FN031_w2_2**) city ____ (**FN031_w2_3**) county

FN032_w2 What type of retirement pension plan is/was your employer s supplement pension?

1. Defined Benefit (DB) Retirement Pension
2. Defined Contribution (DC) Retirement Pension

DB Retirement Pension Plan: A workers retirement pension is determined in advance and the amount paid by the user shall change based on how well the savings are managed.

DC Retirement Pension Plan: The amount paid by the user is determined in advance and the retirement pension paid to a worker shall change based on how well the savings are managed.

PROCEDURE :
Skip FN033_w2 - FN040_w2 if FN030_w2 = 2. FN030_w2 = 2, FN033_w2-FN040_w2

FN033_w2 For how many years altogether have you been included in this plan? [Include years with other employers if the same plan.] [ ]____ 0.00...100.00 Years

FN034_w2 At what age do you expect to start receiving benefits from this plan?
1. At age ____ 0...120 (**FN034_w2_1**) or in ____0...120 (**FN034_w2_2**) years
2. I do not expect receiving these benefits because I have received cash settlements → Skip to FN040_w2 FN040_w2
3. I do not expect receiving these benefits because I have lost benefits → Skip to FN043_w2 FN043_w2
4. Other → Skip to FN043_w2 FN043_w2

FN035_w2 What is the combined monthly contribution from you and your employer? Of which, how much do you pay? ____ (**FN035_w2_1**) Yuan ____ 0.00...100.00 (**FN035_w2_2**) %

FN035_w2_bracket [IWER: IfR is unwilling to answer or does not remember, ask unfolding bracket questions here.]:
500 /1,000 /2,000 /3,500 /5,000 yuan

PROCEDURE :
If FN032_w2 = 1 ask: FN032_w2 = 1

FN036_w2 For a DB plan, do you know how much you are entitled to at (age in FN034_w2)? [preload FN034_w2] ____ (**FN036_w2_1**) Yuan per month / Or ____0.00...100.00 (**FN036_w2_2**) Or ____ (**FN036_w2_3**) Yuan (Lump sum amount)

FN036_w2_bracket [IWER: IfR is unwilling to answer or does not remember, ask unfolding bracket questions here.]:

500 /1,000 /2,000 /3,500 /5,000 yuan

PROCEDURE :
Skip to FN043_w2 FN043_w2

PROCEDURE :
If FN032_w2 = 2 ask: FN032_w2 = 2

FN037_w2 For a DC plan, have you ever checked your account balance?

1. Yes ____ （**FN037_w2_1**） Yuan in_____ （ 1900...2013 （**FN037_w2_2**） Year ____ （0...12（**FN037_w2_3**） Month

   [IWER: Mark the year using four digits. Take down the month as its actual number. For example, write January as 1 not 01, December as 12 . If do not remember month, fill '0. : 4 1 10112 12**O** **]**
2. No

FN038_w2 What is the earliest age at which you could leave this employer and start to receive pension benefits?

____45...120 Years old

FN039_w2 By how much would your pension be reduced from full benefits if you left this job at (AGE IN FN038_w2)? [preload FN038_w2] By ____0.00...100.00 (**FN039_w2_1**) % or ____ (**FN039_w2_2**) Yuan

FN039_w2_bracket [IWER: IfR is unwilling to answer or does not remember, ask unfolding bracket questions here.]:

500 /1,000 /2,000 /3,500 /5,000 yuan

PROCEDURE :
Skip to FN043_w2 FN043_w2

FN040_w2 How much cash settlements did you receive? _______ Yuan

PROCEDURE :
Skip to FN043_w2 FN043_w2

FN041_w2 In what month and year did you start to receive pension benefits from supplement pension insurance of the firms? ____ 1900...2013 (**FN041_w2_1**) year __0...12 (**FN041_w2_2**) month

[F1] [IWER: Mark the year using four digits. Take down the month as its actual number. For example, write January as 1 not 01, December as 12. If do not remember month, fill '0. : 4 1 10112 12**O**]

FN042_w2 What are your monthly benefits? ____ Yuan per month /

Part 3 COMMERCIAL PENSION INSURANCE

FN043_w2 Did you participate in commercial pension insurance, did someone buy commercial pension insurance for you, or did you get commercial pension insurance? /

1. Yes, but I dont receive it /
2. Yes, I receive it
3. No → Skip to FN057_w2 FN057_w2

FN044_w2 Where did you participate in this insurance? 1. The same as permanent address

2. Another village/neighborhood in permanent addresss county/city/district /// _ (**FN044_w2_1**) village/neighborhood /
3. Other ____ (**FN044_w2_2**) province_city_county/city/district _ _ // ____ (**FN044_w2_3**) village/neighborhood /
4. Abroad [IWER:]

FN045_w2 Who paid for the commercial pension insurance? 1. Myself

2. My employer
3. My family or relative
4. Other person

F1

PROCEDURE :

Skip FN046_w2-FN054_w2_bracket if FN043_w2 = 2. FN043_w2 = 2 , FN046_w2-FN054_w2_bracket

FN046_w2 When did you start paying for the commercial pension? ____ 1900...2013 (**FN046_w2_1**) year __0...12 (**FN046_w2_2**) month

F1 [IWER: Mark the year using four digits. Take down the month as its actual number. For example, write January as 1 not 01, December as 12. If do not remember month, fill ‘0. : 4 1 10112 12O]

FN047_w2 How do you contribute to the commercial pension? ? 1. Annual payment

2. Lump sum amount → Skip to FN050_w2 FN050_w2

FN048_w2 You contribute ____ yuan/ year to the commercial insurance ____ Yuan/ year /

FN048_w2_bracket [IWER: IfR is unwilling to answer or does not remember, ask unfolding bracket questions here.]:

500 /1,000 /2,000 /3,500 /5,000 yuan

FN049_w2 How many years do you need to pay? ? ____ Years

Soft Check: Prompt for verification if greater than a legal maximum

**PROCEDURE** :<br>Skip FN050_w2-FN050_w2_bracket if FN047_w2 = 2. FN047_w2 = 2 , FN050_w2-FN050_w2_bracket

FN050_w2 How much premium do you need to pay in total? ____Yuan

FN050_w2_bracket [IWER: IfR is unwilling to answer or does not remember, ask unfolding bracket questions here.]:

500 /1,000 /2,000 /3,500 /5,000 yuan

FN051_w2 How do you receive the pension?

1. Lump sum amount → Skip FN052_w2-FN053_w2_bracketFN052_w2-FN053_w2_bracket
2. Yearly
3. Monthly

**PROCEDURE** :<br>If FN051_w2 = 2 ask FN052_w2 and FN052_w2_bracket

FN052_w2 How much do you expect to receive_____ yuan/ year after your retirement? __ Yuan/year /

Soft Check: Prompt for verification if low or high, e.g. FN052_w2 < 1200 per year or FN052_w2 > 60000 per year

FN052_w2_bracket [IWER: IfR is unwilling to answer or does not remember, ask unfolding bracket questions here.]:

500 /1,000 /2,000 /3,500 /5,000 yuan

**PROCEDURE** :
If FN051_w2 = 3 ask FN053_w2 and FN053_w2_bracket

FN053_w2 How much do you expect to receive _____ yuan/month in the future? ____ Yuan/ month /

Soft Check: Prompt for verification if low or high, e.g. FN053_w2 < 100 per month or FN053_w2 >5000 per year

FN053_w2_bracket [IWER: IfR is unwilling to answer or does not remember, ask unfolding bracket questions here.]:

500 /1,000 /2,000 /3,500 /5,000 yuan

FN054_w2 How much do you expect to receive ______ yuan in total? ____ Yuan

FN054_w2_bracket [IWER: IfR is unwilling to answer or does not remember, ask unfolding bracket questions here.]:

500 /1,000 /2,000 /3,500 /5,000 yuan

**PROCEDURE** :
Skip to FN057_w2. FN057_w2

FN055_w2 In what month and year did you start to receive commercial pension benefits?
1900...2013 (**FN055_w2_1**) year __0...12 (**FN055_w2_2**) month

[IWER: Mark the year using four digits. Take down the month as its actual number. For example, write January as 1 not 01, December as 12. If do not remember month, fill ‘0. : 4 1 1 0112 12**O** **]**

FN056_w2 What is your monthly benefit? ? ____ Yuan per month /

FN056_w2_bracket [IWER: IfR is unwilling to answer or does not remember, ask unfolding bracket questions here.]:

500 /1,000 /2,000 /3,500 /5,000 yuan

Part 4 LIFE INSURANCE

FN056_w2_1 Did you participate in life insurance?

1. Yes
2. No → Skip to FN057_w2 FN057_w2

FN056_w2_2 When did you start to participate in life insurance? _ 1900...2013 (**FN056_w2_2_1**) year __0...12 (**FN056_w2_2_2**) month

FN056_w2_3 What kind of life insurance did you participate in? 1. Term life insurance
2. Whole life insurance
3. Survivorship insurance
4. Endowment insurance
5. Other, please specify （ ____） (**FN056_w2_3_1**)

FN056_w2_4 Who paid for your life insurance?

1. Myself
2. My children
3. Other family member or relative
4. Others

FN056_w2_5 How to pay the insurance premium?

1. Monthly
2. Quaterly
3. Half a year
4. Annually

FN056_w2_6 How much is the payment amount?

1. If pay it monthly ____Yuan/Month / (**FN056_w2_6_1**)
2. If pay it quarterly ____Yuan/Quarter / (**FN056_w2_6_2**)
3. If pay it every six months ____Yuan/Half a year / (**FN056_w2_6_3**) 4. If pay it annually Yuan/Year / (**FN056_w2_6_4**)

FN056_w2_7 Did you receive reimbursement from your life insurance?

1. Yes
2. No → Skip to FN056_w2_9 FN056_w2_9

FN056_w2_8 How much did you get? ____Yuan

FN056_w2_9 How much do you expect to receive? _____ (**FN056_w2_9_1**) Yuan/Month / Or _ (**FN056_w2_9_2**) Yuan （Lump sum amount ）

Part 5 RURAL PENSION, RESIDENTS PENSION, URBAN RESIDENTS PENSION ( )

FN057_w2 What kind of pension did you participate in or receive? (multiple choice) / ( )

1. Rural pension ( )
2. Residents pension
3. Urban residents pension
4. None of above → Skip to FN069_w2 FN069_w2

**PROCEDURE** :
If FN057_w2 is multiple choiced, ask FN058_w2-FN068_w2 for every answer FN057_w2 FN058_w2-FN068_w2

FN058_w2 For every answers ofFN057_w2, do you participate in or receive the pension? [FN057_w2] ( )

1. Participate in /
2. Receive the pension

FN059_w2 Where did you participate in the pension? [FN057_w2]

1. The same as permanent address
2. Another village/neighborhood in permanent addresss county/city/district /// _ (**FN059_w2_1**) village/neighborhood /
3. Other ____ (**FN059_w2_2**) province_city_county/city/district _ _ // ____ (**FN059_w2_3**) village/neighborhood /
4. Abroad

[IWER:]

**PROCEDURE** :
Skip FN060_w2-FN066_w2 if FN058_w2= 2. FN058_w2= 2, FN060_w2-FN066_w2

FN060_w2 Have you ever contributed to your [preload FN057_w2]? [FN057_w2]

[CAPI: (1) (2) IfFN057_w2 = 2, prompt: If your residents pension was transferred from other pension programs, your contribution to these other programs also counts. FN057_w2 = 2]

1. Yes
2. No → Skip FN061_w2 and FN062_w2 FN061_w2 and FN062_w2

FN061_w2 In what month and year did you start to pay for your [preload FN057_w2]? [FN057_w2] ____ 1900…2013 (**FN061_w2_1**) year __0…12 (**FN061_w2_2**) month

FN062_w2 Your annual contribution was ____ Yuan, annual subsidy from the collective was ____ yuan, annual subsidy from the government was ____ Yuan; or your lump sum contribution was ____ Yuan, lump sum subsidy from the collective was ____ Yuan, lump sum subsidy from the government was ____ Yuan. ____ (**FN062_w2_1**) ____ (**FN062_w2_2**) ____ (**FN062_w2_3**) ____ (**FN062_w2_4**) ____ (**FN062_w2_5**) ____ (**FN062_w2_6**)

PROCEDURE:
Ask FN063_w2 and FN064_w2 if the respondent is enrolled in residents pension FN057_w2 = 2. FN057_w2 = 2 FN063_w2FN064_w2

FN063_w2 Was your residents pension transferred from other pension insurances like rural pension and basic pension of the firms?

1. Yes
2. No → Skip FN064_w2 FN064_w2

F1 (1) 1992 (2)

FN064_w2 Which of the following pension program was your residents pension transferred from?

1. Rural pension
2. Basic pension insurance of the firms
3. Other ____(**FN064_w2_1**)

F1 (1) 1992 (2)

PROCEDURE:
If FN058_w2 = 1 ask FN065_w2-FN066_w2.

FN065_w2 When do you expect to receive pension At age ____45...120 (**FN065_w2_1**) years old or in ____ 0.00...100.00 (**FN065_w2_2**) years

Soft Check: Prompt or Verify if FN065_w2_1 <50 or FN065_w2_2 >60

FN066_w2 About how much do you expect your benefits to be? (as an amount per month or year or a lump sum?) ____ (**FN066_w2_1**) Yuan per month / Or ____ (**FN066_w2_2**) Yuan (Lump sum amount)

[Soft Check: Prompt or Verify if These Benefits are Low or High, e.g., FN066_w2_1 <100 | FN066_w2_1 >5000 per month.]

PROCEDURE :
If FN058_w2 = 2 ask FN067_w2-FN068_w2
Otherwise skip to FN069_w2. FN069_w2

FN067_w2 In what month and year did you start to receive your [preload FN057_w2]?
[FN057_w2]____ 1900…2013 (**FN067_w2_1**) year __0…12 (**FN067_w2_2**) month

[IWER: Mark the year using four digits. Take down the month as its actual number. For example, write January as 1 not 01, December as 12. If do not remember month, fill '0. : 4 1 1 01,12 12**O** **]**

FN068_w2 How much do you receive now? (as an amount per month?) Yuan per month
/

Part 6 New Rural Social Pension Insurance

FN069_w2 Do you currently participate in the New Rural Social Pension Insurance program?

1. Yes → Skip to FN071_w2 FN071_w2
2. No

FN070_w2 Why don t you participate in the New Rural Social Pension Insurance program? (Circle all that applies) ( )

1. The New Rural Social Pension has not been introduced in my local area (**FN070_w2_1**)
2. I lack money (**FN070_w2_2**)
3. I am not satisfied with the benefits because it isn t cost-effective. (**FN070_w2_3**)
4. The benefits are poor, and mean nothing to my life. (**FN070_w2_4**)
5. Application and payment arrangements make it inconvenient to participate (**FN070_w2_5**)
6. The mechanism for making contributions is not justifiable. (**FN070_w2_6**) 7. I do not have a local Hukou. (**FN070_w2_7**)
8. I am already covered by other social pension insurance. (**FN070_w2_8**)
9. Other (**FN070_w2_9**)

PROCEDURE :
Skip to FN080_w2 FN080_w2

FN071_w2 In what month and year were you first covered by New Rural Social Pension Insurance? ( ) ____ 2008…2013 (**FN071_w2_1**) year __0…12 (**FN071_w2_2**) month

[IWER: Mark the year using four digits. Take down the month as its actual number. For example, write January as 1 not 01, December as 12. If do not remember month, fill '0. : 4 1 1 0112 12**O**]

FN072_w2 How do you contribute to New Rural Pension Insurance? 1. Annual payment (**FN072_w2_1**) Yuan/Year /

2. Lump sum amount ___ (**FN072_w2_2**) Yuan equals to annual payment ____ (**FN072_w2_3**) Yuan/Year / → Skip to FN074_w2 FN074_w2
3. 15 ____ (**FN072_w2_4**) Yuan ____ (**FN072_w2_5**) Yuan/Year /
4. I don t need to pay myself. I am over 60 years old, and I am covered through my children s participation in the New Rural Social Pension Insurance. 60 → Skip to FN075_w2 FN075_w2

FN073_w2 How many years do you need to pay to receive benefits? ? ( ) ____ Years

FN074_w2 Who pays for your participation in New Rural Social Pension Insurance? ?

1. Myself
2. My children
3. Other family member or relative
4. Others

FN075_w2 Did you start to receive pension benefit? ()

1. Yes
2. No → Skip to FN078_w2 FN078_w2

FN076_w2 In what month and year did you start to receive pension benefits? ____ 2008…2013 (**FN076_w2_1**) year __0…12 (**FN076_w2_2**) month

[IWER: Mark the year using four digits. Take down the month as its actual number. For example, write January as 1 not 01, December as 12. If do not remember month, fill '0. : 4 1 1 0112 12**O**]

FN077_w2 How much do you receive every month? ____ Yuan/ month /

FN077_w2_bracket [IWER: IfR is unwilling to answer or does not remember, ask unfolding bracket questions here.]:

500 /1,000 /2,000 /3,500 /5,000 yuan

PROCEDURE :

Skip to FN080_w2 FN080_w2

FN078_w2 When do you expect to receive pension benefit? At age ____ (**FN078_w2_1**) 45…120 or in ___ (**FN078_w2_2**) years

FN079_w2 About how much do you expect your benefits to be? ____ Yuan per month /

FN079_w2_bracket [IWER: IfR is unwilling to answer or does not remember, ask unfolding bracket questions here.]:

500 /1,000 /2,000 /3,500 /5,000 yuan

Part 7 ENDOWMENT INSURANCE FOR THE LAND-LOSING FARMERS （/）

FN079_w2_1 Whether your land was acquired?

1. Yes
2. No → Skip to FN080_w2 FN080_w2

FN079_w2_2 When was your land acquired? ____ 1900…2013 Year

FN079_w2_3 Did you participate inland expropriation pension insurance, or did you receive the pension? /

1. Yes, but I dont receive it
2. Yes, I receive it → Skip to FN079_w2_10FN079_w2_10 3. No → Skip to FN080_w2 FN080_w2

FN079_w2_4 Do you need to pay the premium by yourself?

1. Yes
2. No → Skip to FN079_w2_7 FN079_w2_7

FN079_w2_5 Who paid for this pension insurance? 1. Myself

2. My children
3. Other family member or relative
4. Others

FN079_w2_6 How much did you need to pay? ____ (**FN079_w2_6_1**) Yuan per month / Or ____ (**FN079_w2_6_2**) Yuan per year /

FN079_w2_6_bracket [IWER: IfR is unwilling to answer or does not remember, ask unfolding bracket questions here.]: 10 /50 /100 /500 /1,000 yuan /

FN079_w2_7 How much did government and community subsidize? Government paid ____ (**FN079_w2_7_1**) Yuan per month / Or ____ (**FN079_w2_7_2**) Yuan per year / Community paid ____ (**FN079_w2_7_3**) Yuan per month / Or ___ (**FN079_w2_7_4**) Yuan per year /

FN079_w2_7_bracket1 [IWER: If R is unwilling to answer or does not remember, ask unfolding bracket questions here.]: 10 /50 /100 /500 /1,000 yuan /

FN079_w2_7_bracket2 [IWER: If R is unwilling to answer or does not remember, ask unfolding bracket questions here.]: 10 /50 /100 /500 /1,000 yuan /

FN079_w2_8 When do you expect to receive this pension At age ____ 45…120 (**FN079_w2_8_1**) years old or in ____ 0.00…100.00 (**FN079_w2_8_2**) years

FN079_w2_9 About how much do you expect your benefits to be? (as an amount per month or year or a lump sum?) ____ (**FN079_w2_9_1**) Yuan per month / Or ____ (**FN079_w2_9_2**) Yuan （Lump sum amount ）

FN079_w2_9_bracket [IWER: IfR is unwilling to answer or does not remember, ask unfolding bracket questions here.]: 10 /50 /100 /500 /1,000 yuan /

PROCEDURE :

If FN079_w2_3 = 2 ask FN079_w2_10-FFN079_w2_11_bracket.

Otherwise skip to FN080_w2. FN080_w2

FN079_w2_10 In what month and year did you start to receive this pension? ____ 1990...2013 (**FN079_w2_10_1**) year __0...12 (**FN079_w2_10_2**) month

[IWER: Mark the year using four digits. Take down the month as its actual number. For example, write January as 1 not 01, December as 12. If do not remember month, fill '0. : 4 1 1 0112 12**O**]

FN079_w2_11 How much do you receive now? (as an amount per month?) ____ Yuan per month /

FN079_w2_11_bracket [IWER: If R is unwilling to answer or does not remember, ask unfolding bracket questions here.]: 10 /50 /100 /500 /1,000 yuan /

Part 8 OLD AGE PENSION ALLOWANCE

FN080_w2 Did you receive old age pension allowance? 1. Yes

2. No → Skip to FN083_w2 FN083_w2

FN081_w2 In what month and year did you start to receive the pension subsidy for the oldest old? ____ 1990...2013 (**FN081_w2_1**) year __0...12 (**FN081_w2_1**) month

F1 ( ) 60 , [IWER: Mark the year using four digits. Take down the month as its actual number. For example, write January as 1 not 01, December as 12. If do not remember month, fill '0. : 4 1 1 0112 12**O**]

FN082_w2 How much do you receive now? ____ Yuan per month /

FN082_w2_bracket [IWER: IfR is unwilling to answer or does not remember, ask unfolding bracket questions here.]:

500 /1,000 /2,000 /3,500 /5,000 yuan

Part 9 OTHER PENSION

FN083_w2 You just told us you are receiving benefits from other pension program, what is the name of this pension program? /

1. Yes /

2. Yes
3. No → Skip to FN097_w2 FN097_w2

FN084_w2 What is the name of the program? _________
[IWER:. /]

FN085_w2 where did you participate in the pension program? 1. The same as permanent address
2. Another village/neighborhood in permanent address s county/city/district /// _ (**FN085_w2_1**) village/neighborhood /
3. Other ____ (**FN085_w2_2**) province_city_county/city/district _ _ // ___ (**FN085_w2_3**) village/neighborhood /
4. Abroad
[IWER:]

**PROCEDURE** :<br>Skip FN086_w2-FN094_w2_bracket if FN083_w2 = 2. FN083_w2 = 2,<br>FN086_w2-FN094_w2_bracket

FN086_w2 When did you start to participate in this pension? ____
1990...2013(**FN086_w2_1**)year __0...12 (**FN086_w2_2**)month
[IWER: Mark the year using four digits. Take down the month as its actual number. For example, write January as 1 not 01, December as 12. If do not remember month, fill ‘0.
: 4 1 1 01,12 12**O**]

FN087_w2 Did you need to pay the premium? 1. Yes
2. No → Skip to FN093_w2 FN093_w2

FN088_w2 Who paid for the this pension insurance? 1. Myself
2. My employer
3. My family or relative
4. Other person

FN089_w2 How do you contribute to the commercial pension? ? 1. Annual/Monthly payment /
2. Lump sum amount → Skip to FN092_w2 FN092_w2

FN090_w2 You contribute ____ Yuan/ year to the commercial insurance ____ Yuan/ year
/

FN090_w2_bracket [IWER: IfR is unwilling to answer or does not remember, ask unfolding bracket questions here.]:

500 /1,000 /2,000 /3,500 /5,000 yuan

FN091_w2 How many years do you need to pay? ? ____ Years

[Soft Check: Prompt for verification if greater than a legal maximum]

**PROCEDURE** :<br>Skip FN092_w2-FN092_w2_bracket if FN089_w2 = 1. FN089_w2 = 1,<br>FN092_w2-FN092_w2_bracket

FN092_w2 How much premium do you need to pay in total? ____Yuan

FN092_w2_bracket [IWER: IfR is unwilling to answer or does not remember, ask unfolding bracket questions here.]:

500 /1,000 /2,000 /3,500 /5,000 yuan

FN093_w2 When do you expect to receive pension At age ___ (**FN093_w2_1**) 45..120 or in ___ (**FN093_w2_2**) years

FN094_w2 About how much do you expect your benefits to be? (as an amount per month or year or a lump sum?) ____ (**FN094_w2_1**) Yuan per month / Or ____ (**FN094_w2_1**) Yuan （Lump sum amount ）

FN094_w2_bracket [IWER: IfR is unwilling to answer or does not remember, ask unfolding bracket questions here.]:

500 /1,000 /2,000 /3,500 /5,000 yuan

**PROCEDURE** :<br>If FN083_w2 = 2 ask FN095_w2 - FN096_w2_bracket . FN083_w2 = 2<br>FN095_w2 - FN096_w2_bracket<br>Otherwise skip to FN097_w2. FN097_w2

FN095_w2 In what month and year did you start to receive this pension benefits? （） ___ 1990…2013 (**FN095_w2_1**) year __0…12 (**FN095_w2_2**) month

[IWER: Mark the year using four digits. Take down the month as its actual number. For example, write January as 1 not 01, December as 12. If do not remember month, fill ‘0. : 4 1 1 0112 12**O** **]**

FN096_w2 How much do you receive now? ____ Yuan per month /

FN096_w2_bracket [IWER: IfR is unwilling to answer or does not remember, ask unfolding bracket questions here.]:

500 /1,000 /2,000 /3,500 /5,000 yuan

Ask all R

FN097_w2 Who do you think you can rely on financially for old-age support

1. Children → Skip to FN098_w2 FN098_w2
2. Savings
3. Pension or retirement salary
4. Commercial pension insurance
5. Other

F1

FN098_w2 Which child(ren)? / (choose all that apply) [ ]

G&H INCOME, EXPENDITURES AND ASSETS

PROCEDURE :
For new R, please skip section G1. G1

G1 RELATIVE INCOME

[INTRO: The following questions are on subjective feelings :]

[Show Card 24]

G001_W2 Compared to the average living standard of your relatives, how would you rate your standard of living?

1. Much better
2. a little better
3. about the same
4. a little worse
5. much worse
6. I do not know

G002_W2 Compared to the average living standard of your schoolmates who are at the same level of education with you, how would you rate your standard of living?

1. Much better
2. a little better
3. about the same
4. a little worse
5. much worse
6. I do not know
7. Not applicable

G003_W2 Compared to the average living standard of your colleagues, how would you rate your standard of living?

1. Much better
2. a little better
3. about the same
4. a little worse
5. much worse

6. I do not know
7. Not applicable

G004_W2 Compared to the average living standard of your neighbors or others in your village or neighborhood, how would you rate your standard of living? /

1. Much better
2. a little better
3. about the same
4. a little worse
5. much worse
6. I do not know

G005_W2 Compared to the average living standard of people in your city or county, how would you rate your standard of living? //

1. Much better
2. a little better
3. about the same
4. a little worse
5. much worse
6. I do not know

G2 HOUSEHOLD INCOME AND EXPENDITURES

[IWER: Part 1_1 is asked of the main respondent and spouse respectively. Other parts in this section is asked of the family respondent. Do not allow a proxy respondent to answer the entire section. Part 1_1]

PART 1 Household Wage Income and Individual-based transfers

Part 1_1: Main Respondent and Spouses Wage Income and Individual-based transfers

[IWER: Please conduct Part 1_1 when the main respondent and spouse are at home. Dont allow a proxy to complete the part. Part 1_1]

GA001 Did you receive any wage and bonus income in the past year? ()

1. Yes

2. No → Skip to GA003 GA003

GA002 How much did you receive last year? _____ (**GA002_1**) yuan _____ (**GA002_2**) yuan/month

Wage income: yuan [soft check >240,000] or yuan/month [soft check>18,000]

GA002_bracket [IWER: If R is unwilling to answer or does not remember, ask unfolding bracket questions here.]

10,000 /30,000 /50,000 /100,000 /200,000 yuan

GA002_W2_1 Does the above mentioned wage exclude any insurance, income tax, public housing funds and other fees?

1. Yes
2. No

GA002_W2_2 What is the total amount ofyour insurance, income tax, public housing funds and other fees? / _______ (**GA002_w2_2a**) Yuan/year / or ______ (**GA002_w2_2b**) Yuan/month / or about ___ (**GA002_w2_2c**) % of wage

[IWER: If R is unwilling to answer or does not remember, ask unfolding bracket questions here. :] 300 /500 /1,000 /2,000 /3,000 yuan/month /

Among it, :

1. Income tax _______ (**GA002_w2_2_1a**) Yuan/Yea / ______ (**GA002_w2_2_1b**) Yuan/Month /, or about ___ (**GA002_w2_2_1c**) % of wage.

   [IWER: If R is unwilling to answer or does not remember, ask unfolding bracket questions here. : 0 /50 /100 /500 /1,000 yuan /

2. Various insurance (pension insurance, health insurance, unemployment insurance,worker s injury insurance, maternity insurance) ( ) _______ (**GA002_w2_2_2a**) Yuan/Year / _____ (**GA002_w2_2_2b**) Yuan/Month /, or about __ (**GA002_w2_2_2c**) % of wage.

   [IWER: If R is unwilling to answer or does not remember, ask unfolding bracket questions here. :] ( ) 0 /50 /100 /500 /1,000 yuan/month /

3. Public housing fund _______ (**GA002_w2_2_3a**) Yuan/Year / _____ (**GA002_w2_2_3b**) Yuan/Month /, or about ___ (**GA002_w2_2_3c**)

% of wage

[IWER: If R is unwilling to answer or does not remember, ask unfolding bracket questions here. :] 0 /50 /100 /500 /1,000 yuan/month /

[Show Card 25]

GA003 Did you receive any of the following types of individual income in the past year? (check all that apply) ()

1. Pensions (including wages from government institutions and firms, supplemental pension of the firms,and income from such programs as rural pension insurance, Urban residents pension and commercial pension insurance, new rural social pension insurance and pension subsidy for the oldest old) ( )
2. unemployment compensation
3. pension subsidy
4. Workers compensation from Industrial Accident Compensation Insurance includes wage-replacement benefits, disability benefits, and survivors benefits
5. elderly family planning subsidies
6. medical aid
7. other government subsidies
8. social assistance
9. other income sources
10. None of the above → Skip GA004 GA004

GA004 How much did you receive last year? ____ (**GA004_1**) Yuan ____ (**GA004_2**) / Yuan/Month

**PROCEDURE** :<br>If GA003 = 1 and GA004 = DK, ask GA004_bracket. GA004_bracket

GA004_bracket [IWER: If R is unwilling to answer or does not remember, ask unfolding bracket questions here. :]
10,000 /30,000 /50,000 /100,000 /200,000 yuan

Part 1_2: Other Household Members Wage Income and Individual-based transfers

[IWER reminder: make sure others are not present.]

[Intro: We d like to ask you some questions about the income and assets of OTHER members of your household.]

PROCEDURE :

The names of other household members are preloaded from the cover screen information. For each member (excluding main respondent and spouse)ask GA005 to GA008_bracket. （ ） GA005 GA008_bracket

GA005 Did [preload household member name] receive any wage and bonus income in the past year? [preload household member name] （ ）

1. Yes
2. No → Skip to GA007 GA003

GA006 After tax and various social insurances, how much did he/she receive in the past year year? / ______ (**GA006_1**) yuan or _____ (**GA006_2**) yuan/month

Wage income: yuan [soft check >200,000] or yuan/month [soft check>15,000]

GA006_bracket [IWER: If R is unwilling to answer or does not remember, ask unfolding bracket questions here.]

10,000 /30,000 /50,000 /100,000 /200,000 yuan

GA006_w2 [preload household member name] ____ (**GA006_w2_1**) yuan/month / or _____ (**GA006_w2_2**) yuan /

GA006_w2_bracket [IWER: IfR is unwilling to answer or does not remember, ask unfolding bracket questions here.]

300 /500 /1,000 /2,000 /3,000 yuan /

[Show Card 25]

GA007 Did [preload household member name] receive any of the following types of individual income in the past year? (check all that apply) [preload household member name] （）

1. Pensions (including wages from government institutions and firms, supplemental pension of the firms,and income from such programs as rural pension insurance, Urban residents pension and commercial pension insurance, new rural social pension insurance and pension subsidy for the oldest old) （ ）

2. unemployment compensation
3. pension subsidy
4. Workers compensation from Industrial Accident Compensation Insurance includes wage-replacement benefits, disability benefits, and survivors benefits
5. elderly family planning subsidies
6. medical aid
7. other government subsidies
8. social assistance
9. other income sources
10. None of the above → Skip to GA008 GA008

[F1]

GA008 How much did he/she receive last year? ____ (**GA008_1**) yuan [soft check >10000] or (**GA008_2**) yuan/month / [soft check >3000]

> **PROCEDURE** :
> If GA007 = 1 and GA008 = DK, ask GA008_bracket. GA008_bracket

GA008_bracket [IWER: If R is unwilling to answer or does not remember, ask unfolding bracket questions here. :]

10,000 /30,000 /50,000 /100,000 /200,000 yuan

> **PROCEDURE** :
> Skip to next person.

PART 2 HOUSEHOLD AGRICULTURAL INCOME AND EXPENDITURE

[Intro: Next we will ask some questions about your household agricultural income and expenditure.]

GB001 Did your household engage in agricultural work (including cropping, forestry, livestock, and fish) last year?

1. Yes
2. No → Skip to GC001 GC001

GB002 [IWER: The names of other household members not including respondent and spouse are preloaded from the cover screen information:] [preload other household member name] Who engaged in agricultural work in the past year? ()

Crops and forestry products

GB003 Did your household engage in cropping or forestry last year? ()

1. Yes
2. No → Skip to GB007 GB007

GB004 When was the most recent harvest? ____2009...2013 (**GB004_ 1**) year ____0...12 (**GB004_2**) month

[IWER: Mark the year using four digits. Take down the month as its actual number. For example, write January as 1 not 01, December as 12. If do not remember month, fill 0. : 4 1 1 0112 12**O** **]**

GB005 What is the total value of all crops and forestry products produced in the past year? (**GB005_1**) Yuan [soft check: 75000 yuan] Among it, what is the value of the crops and forestry products that is home consumed? ____ (**GB005_2**)Yuan __ (**GB005_3**) %

GB005_bracket [IWER: If R is unwilling to answer or does not remember, ask unfolding bracket questions here. :]
1,000 /3,000 /5,000 /7,000 /10,000 yuan

GB005_w2_bracket [IWER: IfR is unwilling to answer or does not remember, ask unfolding bracket questions here. :]
1,000 /3,000 /5,000 /7,000 /10,000 yuan

GB006 What was the total cost of producing crops (including vegetables and Chinese herbs) and forestry products in the past year? (including Seeds (including home-used seeds), Fertilizer, Organic fertilizer, Pesticide, Plastic sheets, Hiring labor (including with machine or animals), Land rents, Rents (excluding land rents), Irrigation, Fuel, Transportation, Processing, Marketing (including packaging, management fee)) () ( ) [() () ( ) ( )] ___ Yuan [soft check: 50,000 yuan]

GB006_bracket [IWER: If R is unwilling to answer or does not remember, ask unfolding bracket questions here. :]

3,00 /6,00 /1,000 /2000 /5,000 yuan

Livestock and fisheries

GB007 Did your household grow any livestock or aquatic life last year? （）

1. Yes
2. No → Skip to GC001 GC001

GB008 What is the current value of all livestock（including chicken, duck, cattle, pig, sheep, etc.）and aquatic life? （） ____ Yuan [soft check: 100,000 yuan]

GB008_bracket [IWER: If R is unwilling to answer or does not remember, ask unfolding bracket questions here. :]

5,00 /1,500 /3,000 /4,500 /9,000 yuan

GB009 What was the value of all livestock and aquatic life at this time last year? ____ Yuan [soft check: 100,000 yuan]

GB009_bracket [IWER: If R is unwilling to answer or does not remember, ask unfolding bracket questions here. :]

5,00 /1,500 /2,500 /4,000 /8,000 yuan

GB010 How much did you spend purchasing new livestock and aquatic life in the past year? Yuan [soft check: 50,000]

GB011 What was the value of all livestock and aquatic life that were sold or consumed in the past year? ?____ (**GB011_1**) Yuan [soft check: 100,000 yuan] Among it, what is the amount or percent consumed at your home? ____ (**GB011_2**) Yuan ___ (**GB011_3**) %

GB011_bracket [IWER: If R is unwilling to answer or does not remember, ask unfolding bracket questions here. :]

2,00 /9,00 /1,500 /2,500 /5,000 yuan

GB011_w2_bracket [IWER: IfR is unwilling to answer or does not remember, ask unfolding bracket questions here. :]

2,00 /9,00 /1,500 /2,500 /5,000 yuan

GB012 What was the value of all livestock products produced (including the self consumption value) in the past year, including milk, wool (including cashmere, sheep or goat skin), and eggs? （ ） （） ____ (**GB012_1**) Yuan [soft check: 50,000 yuan] Among it, what is the amount or percent consumed at your home? ____ (**GB012_2**) Yuan ___ (**GB012_3**) % [soft check: 50,000 yuan]

GB012_bracket [IWER: If R is unwilling to answer or does not remember, ask unfolding bracket questions here. :]
（） 100 /200 /300 /500 /1000 yuan

GB012_bracket_2 [IWER: If R is unwilling to answer or does not remember, ask unfolding bracket questions here. :]
100 /200 /300 /500 /1000 yuan

GB013 What was the cost of producing livestock and aquatic life in the past year, including the value of all feed, medicine, pasture fees, animal pens, wages, etc. ____ Yuan
[soft check: 50,000 yuan] [soft check: reported raising livestock but no evidence of such activityif GB008 = 0 and GB009 = 0 GB008 = 0 GB009 = 0]

PART 3 Self-employed Activities

GC001 Did your household members engage in any self-employed activities last year?
1. Yes
2. No → Skip to GD001 GD001

GC002 How many types of activities did your household members participate in the past year? ____ activities.

**PROCEDURE** :<br>For each each self-employed activity in GC002, ask GC003 - GC005. GC003 - GC005

GC003 Who engaged in this self-employment businesse in the past year?
[IWER: All the names of household members are preloaded from the cover screen information （）] ( )

[Show Card 26]

GC004 Which types of activities?

1. Services (cooking, sewing, private clinic etc.) ( ) (**GC004_1**)
2. Transportation (**GC004_2**)
3. Construction (**GC004_3**)
4. Mining (**GC004_4**)
5. Processing production (**GC004_5**)
6. Business (**GC004_6**)
7. Others (**GC004_7**)

GC005 Not including fixed capital costs, what is your best estimate of the net income earned from this activity by your household members last year? [If the activity was conducted jointly with non-household members, report only the net income earned by household members. Remember to consider the following types of costs: energy, housing or equipment rental, raw materials, transportation, marketing, wages, taxes or fees.]
Yuan

[soft check: 500,000 yuan]

GC005_bracket [IWER: If R is unwilling to answer or does not remember, ask unfolding bracket questions here. :]
5,000 /10,000 /50,000 /100,000 /200,000 yuan

PART 4 HOUSEHOLD PUBLIC TRANSFER INCOME

[We ask the public transfers received by the households (with household as the unit). Public transfers have characteristic of welfare, such as Wubaohu Subsidy and Tekunhu Subsidy gived by government.]

GD001 How much Dibao assistance did your household receive last year? (if not applicable, fill in 0 yuan). (0) ____ Yuan

[Show Card 27]

GD002 Did your household receive any of the following government subsidies in the past year? (check all that apply) ()
[soft checks for each category: 20,000 yuan 20,000]

1. Reforestation : how much? ____ (**GD002_1**) Yuan
2. Agricultural subsidies : how much? ____ (**GD002_2**) Yuan

3. Wubaohu (targets low-income, blind, disabled, aged persons, and young persons that have no means to support themselves. (: how much? ____ (**GD002_3**) Yuan
4. Tekunhu : how much? ____ (**GD002_4**) Yuan
5. Work injury subsidies to the immediate family members how much? ____ (**GD002_5**) Yuan
6. Emergency or disaster relief (jiujikuan, jiuzaikuan) last year? () () : how much? (**GD002_6**) Yuan
7. Other : how much? ____ (**GD002_7**) Yuan
8. None → Skip to GD003 GD003

[Show Card 28]

GD003 Did your household receive any income from the following sources in the past year? (check all that apply) ()

1. Donations from the society (including cash, and items like food, clothing, etc.) ( ) : how much? ____ (**GD003_1**) Yuan [soft check: 20,000 yuan]
2. Compensation for land seizure last? : how much? ____ (**GD003_2**) Yuan [soft check: 100,000 yuan]
3. Compensation to pulling down your house or apartment last year? : how much? _ (**GD003_3**) Yuan [soft check: 100,000 yuan]
4. None

PART 5 HOUSEHOLD LIVING EXPENDITURE

GE001 We wish to know your family food expenditure for the last week. Are you the primary person who purchases food for the household?

1. Yes → skip to GE004 GE004
2. No

GE002 Who is the primary person purchasing food for the household?

[CAPI: Preload all the HHmember list]

[IWER: If possible, the primary person who purchases food for the household should answer the questions about expenditures FE003-FE009] [ ]

GE004 In the past week, how many people usually ate meals together in your household (not including guests)? () __ Persons

[soft check: 10]

GE005 Last week how many meals did you provide to guests? ( ) ____ meals [soft check: 100]

[Intro: The next questions are about your household living expenditure, including your household members '(preloaded names of household members) living expenditure. If one attends school/work outside and comes home almost every week, GE006 –GE008 includes his/her expendituréon food and meals outside. If one attends school/work outside but not come home every week, GE006 –GE008 excludes his/her expendituréon food and meals outside.] [/ /]

GE006 In the past week, how much did your household spend on food (excluding eating out expenditure, alcohol, cigarettes, cigars and tobacco expenditure)? ____ Yuan [soft check: 6000 yuan]

GE006_W2 Does your household produce agricultural products yourself (including plants, meat, eggs, aquatic lives, oil, vegetables and fruits, cigaretts and wine, drinks and milk products, produced food, seasonings, etc.)? ( )

1. Yes
2. No → skip to GE007 GE007

GE006_W2_1 In the past week, what was the market value of the food that members of the household consumed that you grew yourselves? ____ Yuan

GE007 Among it, how much did your household spend on eating out? ____ Yuan [soft check: 3000 yuan]

GE008 Among it, how much did your household spend on alcohol, Cigarettes, cigars and tobacco? ____ Yuan [soft check: 3000 yuan]

[Show Card 29]

GE009 Please tell me the expenditure last month for your household for the following items. [soft check for each category: 5000 yuan] [IWER: fill in 0 if no corresponding expenditure; fill in -9999 if the respondent cannot recall the expenditure. : 0 -9999]

1. Communication fees (including post, internet usage, telephone and cell phone usage) ( ) ____ **(GE009_1)** Yuan

2. Utilities: Water and electricity ____ (**GE009_2**) Yuan
3. Fuels (including gas, coal, etc.) （ ） ____ (**GE009_3**) Yuan
4. Fees for Matron, housekeepers and servants __(**GE009_4**) Yuan
5. Local Transportation ____ (**GE009_5**) Yuan
6. Household items and personal toiletries that are used daily plus beauty treatments (e.g., detergent, soap, toothpaste, toothbrush, cosmetics, beauty salon, etc.) （） ____ (**GE009_6**) Yuan
7. Entertainment (including fees to buy books, newspapers, VCCs, DVDs, going to cinema and bars) （） ____ (**GE009_7**) Yuan

[Show Card 30]

GE010 In the last year how much did your household spend on the following items?

[IWER: fill in 0 if no corresponding expenditure; fill in -9999 if the respondent cannot recall the expenditure. : 0 -9999] [soft check: >=100,000 yuan]

1. Clothing and bedding ____ (**GE010_1**) Yuan
2. Long distance traveling expenses ____ (**GE010_2**) Yuan
3. Heating(centrally heated) ()_____ (**GE010_3**) Yuan
4. Furniture, consumption of durable goods and electronics, includes refrigerator, washing machine, TV, computers and expensive instruments like pianio. （） ___ (**GE010_4**) Yuan
5. Education and training(including tuition, training fees, etc.) （ ） ____ (**GE010_5**) Yuan
6. Medical expenditure （ ） ____ (**GE010_6**) Yuan
7. Fitness expenditures （） ____ (**GE010_7**) Yuan
8. Beauty (including make-ups, facials, massages, etc.) （ ） ____ (**GE010_8**) Yuan
9. Automobiles ____ (**GE010_9**) Yuan
10. Purchase, Maintenance and repair (of transportation vehicles, appliances, communication products, etc.) （ ） （） ____ (**GE010_10**) Yuan
11. Property management fees (including parking fee) （） ____ (**GE010_11**) Yuan

12. Taxes and fees turned over to the government （ ） ____ (**GE010_12**) Yuan
13. Donations to the society (including cash, and items like food, clothing, etc.) （ ） ____ (**GE010_13**) Yuan

GE011 How often did the respondent receive assistance in answering section Household income and expenditure?

[IWER: If it is answered by a proxy, please record the respondents reaction.]

1. Never
2. A few times
3. Most or all of the time

HA HOUSEHOLD ASSETS

[IWER: This section is asked of the family respondent. Do not allow a proxy respondent to answer the entire section.]

PART 1 Current Residence

The following questions pertain to your current residence.

HA000_W2 Where is your current house located? ___________ (**HA000_1_PSU/HA000_1_PSU_OTHER**) Province/District / Zip Code _(**HA000_2**)/ ____________ (**HA000_3**)

HA000_W2_0

1.
2. / ____ (**HA000_w2_0_1**) → skip to HA026_W2 Branchpoint HA026_W2 Branchpoint
3. ____ (**HA000_w2_0_2**) → skip to HA026_W2 Branchpoint HA026_W2 Branchpoint
4. ____ (**HA000_w2_0_3**) → skip to HA026_W2 Branchpoint HA026_W2 Branchpoint
5. ____ (**HA000_w2_0_4**) → skip to HA026_W2 Branchpoint HA026_W2 Branchpoint

HA000_W2_1 BRANCHPOINT :

IF THIS IS A NEW INTERVIEWED HOUSEHOLD, GO TO HA001 HA001.

IF THIS IS A REINTERVIEWED HOUSEHOLD, GO TO HA000_W2_1 HA000_W2_1.

HA000_W2_1 [ZIWTime] [ ]

1. Yes → skip to HA001_W2 HA001_W2
2. No

HA001 When did your household start to live at your current residence? ____1900…2013 Year

[IWER: Mark the year using four digits. : 4]

HA001_W2 What is the construction area of the house? ____ m^2 [soft check<10 or >500]

HA002 Do you pay rent for your current residence? 1. Yes

2. No → skip to HA005 HA005

HA003 How much rent do you pay each month? ______ Yuan / month / [soft check: <100, >10,000 yuan]

HA004 Did you pay less than the market rental value? 1. Yes

2. No → skip to HA006 HA006

HA005 If you rented the same housing unit from the market, what is the rent per month you would have to pay? ____ Yuan/month / [soft check: <100, >10,000 yuan]

HA006 How much of the rent was paid by a housing subsidy from the employer of a household member? which household member? _____ (**HA006_1**)yuan [soft check >10,000 yuan] ____ (**HA006_2**) [preloaded list]

HA007 Who owns your current residence?

1.
2.
3. → skip to HA010 HA010

HA007_W2_1 [CAPI: Ask this question only when the earlier records show that ONE of the respondent (main respond OR the spouse passed away in the last two years] Has the owndership status changes following the death of [preload the name of the deceased main respondent or spouse] []

1. Yes

2. No → skip to HA008 HA008

HA007_W2_2 [] __ %.

HA007_W2_3 [] ?

[60

5050]

[Hard check: 100]

1. ___ (**HA007_w2_3_1[1]**)
2. ___ (**HA007_w2_3_1[2]**)
3. ___ (**HA007_w2_3_1[3]**)
4. ___ (**HA007_w2_3_1[4]**)
5. / ___ (**HA007_w2_3_1[5]**)
6. ___ (**HA007_w2_3_1[6]**)
7. ___ (**HA007_w2_3_1[7]**)
8. ___ (**HA007_w2_3_1[8]**)

PROCEDURE :
（HA007_W2_3 = 2） HA007_W2_4 HA007_W2_5

HA007_W2_4 [Load proxy]

HA007_W2_5 [HA007_W2_4] [ ] ___ %?

PROCEDURE :
（HA007_W2_3 = 5） HA007_W2_6 HA007_W2_7

HA007_W2_6 [Load proxy]

HA007_W2_7 [HA007_W2_6] [ ] __ %

HA008 Which household member(s) own the house? (preloaded names of household members) / ____ []

HA009 What share of the house is owned by [preloaded names of household members]? （ HA008 ） ____ 0.00...100.00 % [hard check: range 0-100]

PROCEDURE :
Skip to HA011. HA011

HA010 Which non-household members own all or part of your current residence? (circle all that apply) （）

1. Working unit of household member, which household member? ____ (**HA010_1**) [preloaded list] []
2. Government indemnificatory housing
3. Child(non- household member) of main respondent or spouse （ ）, which child? (**HA010_2**) [preloaded list] [ ]
4. Parent(non- household member) of main respondent or spouse （ ） ____ (**HA010_3**) [preload list] []
5. Nonresident other relatives
6. Friends
7. Other

HA011 What is the present market value of your house? Or, what is the present market value of a similar housing unit within its neighborhood?
Total price ____ (**HA011_1**) 10000 Yuan [soft check < 10, > 500] Or unit price ____ (**HA011_2**) 1000 Yuan/m^2 /
[IWER: Skip to HA013 if R answered HA011. If not, ask unfolding brackets. HA011HA013]

HA012 [IWER: If R is unwilling to answer or does not remember, ask unfolding bracket questions here.]
20,000/50,000/100,000/200,000/500,000 yuan

**PROCEDURE** :
If HA007 = 2/3 , then skip to HA025. HA007 = 2/3 HA025

HA013 Do you or other household members take out a bank loan to finance the purchase, construction, or decoration of your house now? （）
1. Yes
2. No → go back HA016 HA016

HA014 What is the outstanding amount of the loans? _ 10,000 Yuan [soft check: >500,000 yuan]

HA014_W2 How much is the unpaid interest? ____ 10,000 Yuan

HA015 What is the monthly mortgage payment? ____ Yuan [soft check: >20000]

HA016 How was this housing unit obtained?
1. Purchased from market

2. Purchased from working unit of household member(s) . ____(**HA016_1**) [preloaded list] []
3. Purchased by child (non-household member) of main respondent or spouse, which child? ( ) ____ (**HA016_2**) [preloaded list] []
4. Purchased by Parents(non- household member), of who (main respondent or spouse) ? ( ) ( ) ____ (**HA016_3**)
5. Purchased from Other relatives
6. Self-built
7. Inherited, bequeathed, or given ( )
8. Received home as compensation for demolition of old home 9.

Other → skip to HA020 HA020

HA017 When did you purchase it? //// ____ 1900...2013 Year [IWER: Mark the year using four digits. : 4]

**PROCEDURE** :<br>If HA016 = 7 , then skip to HA020. HA016 = 7 HA020.

HA018 How much of your own money did you spend on this housing? // ____ 10,000 Yuan [soft check >500]

**PROCEDURE** :<br>If HA016 = 6 , then skip to HA024. HA016 = 6 HA024. If HA016 = 8 , then skip to HA022. HA016 = 8 HA022.

HA019 Was it purchased at market price, subsidized by working unit, or as purchased as economical housing?

1. Market price → skip to HA024 HA024
2. Subsidized by working unit
3. Economic housing
4. Other

HA020 What would you have to pay if you had paid a market-set price for the same housing? Total price ____ (**HA020_1**) 10,000 Yuan [soft check < 10, > 500] or unit price ____ (**HA020_2**) 1,000 Yuan/m^2 / [soft check: < 1, > 25]

**PROCEDURE** :<br>If HA020_1 >= 500, or HA020_1 < 10, or HA020_2 < 1, or HA020_2 > 25, ask HA020_check. HA020_1 >= 500HA020_1 < 10 HA020_2 < 1 HA020_2 > 25 HA020_check

HA020_check I heard that the total price of your have paid a market-set price of [preload HA020_1] 10,000 Yuan, is this correct? [Or] I heard that the unit price of your house is [preload HA020_2] 1,000 Yuan/square meter, is this correct? [preload HA020_1] / [preload HA020_2]

1. Yes
2. No → go back HA020 HA020

PROCEDURE :
Ask HA022 – HA023_check only if HA016 chooses 8.

HA022 How much was the market value of the old house at that time? Total price ____ 10,000 Yuan [soft check: < 1, > 500]

PROCEDURE :
If HA022 < 1, or HA022 >= 500, ask HA022_check. HA022 < 1HA022 >= 500 HA020_check

HA022_check I heard that you paid a total price of [preload HA022] 10,000 Yuan, is this correct? [HA022]

1. Yes
2. No → go back HA022 HA022

HA023 How much was the market value of the new house at that time? Total price ____ (**HA023_1**) 10,000 Yuan [soft check: <10, >500] or unit price ____ (**HA023_2**) 1,000 Yuan/m^2 / [soft check: <1, >25]

PROCEDURE :
If HA023_1 ≥ 500, or HA023_1 < 10, or HA023_2 < 1, or HA023_2 > 25, ask HA023_check. HA023_1 ≥ 500HA023_1 < 10 HA023_2 < 1HA023_2 > 25 HA023_check

HA023_check I heard that the market value of the new house is [preload HA023_1] 10,000 Yuan at that time, is this correct? [HA023_1]

1. Yes
2. No → go back HA023 HA023

HA024 Can you sell the house freely?

1. Yes
2. No, restricted by work unit

HA025 How much did you spend on decorating or renovating all of your houses (exclude expenditures on furniture) in the past year? ( ) ____ Yuan [soft check: >500,000 yuan]

HA025_W2 How much did you spend on remodelling all of your houses (exclude expenditures on decorating) in the past year? ( ) _________ Yuan [soft check: >500,000 yuan]
[ ]

HA026_W2 BRANCHPOINT:
HA027
HA000_W2_1 = 1 PART 2.

[(HA026_W2_ 1[25]-HA026_W2_ 14_bracket[25])
HA026_W2_1-HA026_W2_14_bracket]

HA026_W2_1 []

1. →
2. Empty →
3. Sold
4. Rented out →
5. Being torn down → skip to HA026_W2_6 HA026_W2_6
6. My child(ren) stay in it → skip to HA026_W2_9 HA026_W2_9
7. My parent(s) stay in it → skip to HA026_W2_9 HA026_W2_9
8. Others (relatives, friends) stay in it () → skip to HA026_W2_9 HA026_W2_9
9. Give to others as gift → skip to HA026_W2_12 HA026_W2_12
10. Last residence is rented, no longer rent it →
11. Other, please specify ____ (**HA026_W2_1a**) →
12. Last record is wrong, the mentioned residence is not ours →

HA026_W2_2 When did you sell that house? ______ Year __ Month ____ (**HA026_W2_2_1**) __ (**HA026_W2_2_2**)

HA026_W2_3 What is the net income you got from selling the house, excluding relative fees? Total price ____ 10,000 Yuan [soft check < 0:1, > 500]

HA026_W2_4 [IWER: IfR is unwilling to answer or does not remember, ask unfolding bracket questions here.]
20,000/50,000/100,000/200,000/500,000 yuan

HA026_W2_5 How did you distribute the income from selling this house? (Can be multiple choice, ask the respondent to fill in corresponding percentages.) []

1. Deposits in bank account ___ (**HA026_W2_5a**) % of total income
2. Everyday living expenses ___ (**HA026_W2_5b**) % of total income
3. Buying new houses ___ (**HA026_W2_5c**) % of total income
4. Medical expenditure ___ (**HA026_W2_5d**) % of total income
5. Other important expenses ___ (**HA026_W2_5e**) % of total income
6. Trasfer to children ___ (**HA026_W2_5f**) % of total income
7. Transfer to parents ___ (**HA026_W2_5g**) % of total income
8. Transfer to relatives ___ (**HA026_W2_5h**) % of total income
9. Other, please specify ___ (**HA026_W2_5i**) ___ (**HA026_W2_5j**) % of total income

[CAPI:]

HA026_W2_6 Did you receive any compensation for tearing down the house?
1. Yes
2. No →

HA026_W2_7 When did you receive the compensation for tearing down the house? ____ Year __ Month

HA026_W2_8 What is the total amount of the compensation? ____ 10,000 Yuan

HA026_W2_8_bracket [IWER: If R is unwilling to answer or does not remember, ask unfolding bracket questions here.] 20,000/50,000/100,000/200,000/500,000 yuan

[CAPI:]

HA026_W2_9 Have those who live in your house pay you any cash, or provide you in-kind payment?
1. Yes
2. No →

HA026_W2_10 How did those who live in your house pay you the cash or the in-kind payment?
1. One lump-sum payment

2. Monthly payment
3. Irregular payment over the year

HA026_W2_11 What is the total amount did those who live in your house pay you in the past year? ____ (**HA026_W2_11a**) Yuan Among it, cash is ____ (**HA026_W2_11b**) Yuan in-kind payment is ____ (**HA026_W2_11c**) Yuan

HA026_W2_11_bracket [IWER: If R is unwilling to answer or does not remember, ask unfolding bracket questions here.] 20,000/50,000/100,000/200,000/500,000 yuan

[CAPI:]

HA026_W2_12 Have those who got your house as gifts pay you any cash, or provide you in-kind payment?
1. Yes
2. No →

HA026_W2_13 How did those who got your house as gifts pay you the cash or the in-kind payment?
1. One lump-sum payment
2. Monthly payment
3. Irregular payment over the year

HA026_W2_14 What is the total amount did those who got your house as a gift pay you in the past year? ____ (**HA026_W2_14a**) Yuan Among it, cash is ____ (**HA026_W2_14b**) Yuan in-kind payment is ____ (**HA026_W2_14c**) Yuan

HA026_W2_14_bracket [IWER: If R is unwilling to answer or does not remember, ask unfolding bracket questions here.] 20,000/50,000/100,000/200,000/500,000 yuan

[CAPI:]

**PROCEDURE** : HA027 - HA028

HA027 Excluding the house in which you live, do you or members of your household own any other residential properties? ( )
1. Yes
2. No → skip to HA054 HA054

HA028 How many other housing units do you or members of your household currently own?
__ 0…10

PART 2 Other Residences

[For every other housing unit owned by house members, ask the following questions,]

HA029_W2 BRANCHPOINT:
IF THE HOUSEHOLD IS A NEW INTERVIEW HOUSEHOLD, THEN SKIP TO HA029.
HA029
IF THE HOUSEHOLD IS A RE-INTERVIEW HOUSEHOLD, SKIP TO HA029_W2.
HA029_W2

HA029_W2 In the past two years since [preload the year of last wave] Year [preload the month of last wave] month, have your household obtained (purchased/built/inherited/received as gift/got new house due to tearing down) any new houses? [ZIWTime] （////） （ ）

1. Yes, obtained __ 0...10 (**HA029_W2_1**) houses
2. No → skip to HA051_W2_1 Branchpoint HA051_W2_1 Branchpoint

[CAPIHA029-HA051]

HA029 Now we want to know information about other housing unit(s) owned by your household members. n （ ） [CAPI: If this is new respondent, then n starts with 1, loop until the number recorded in HA028. n 1 HA028 If this is R from last wave and select 1 in HA029_W2, then n starts from 1, ends at the number given by HA029_w2_1. HA029_W2=1 n 1 HA029_w2_1 If this is R from last wave and select 2 in HA029_W2, then skip to HA051_W2_1. HA029_W2=2 HA051_W2_1]

Where is this house located? ___________ (**HA029_1_PSU/HA029_1_PSU_OTHER**)
Province/District / Zip Code _______ (**HA029_2**)/ ___ (**HA029_3**)

HA030 Who owns this residence?

1. Owned completely by your household member(s).
2. Owned partly by your household member(s).

HA030_W2_1 [CAPI: Ask this question only when the earlier records show that ONE of the respondent (main respond OR the spouse passed away in the last two years] Has the owndership status changes following the death of [preload the name of the deceased main respondent or spouse]? []

1. Yes

2. No → skip to HA031 HA031

HA030_W2_2 [] __ %.

HA030_W2_3 [] ? [60% 50% 50% 50%, 50% HA030]

[Hard check: 100]

1. ___ (**HA030_w2_3_1[1]**)
2. ___ (**HA030_w2_3_1[2]**)
3. ___ (**HA030_w2_3_1[3]**)
4. ___ (**HA030_w2_3_1[4]**)
5. / ___ (**HA030_w2_3_1[5]**)
6. ___ (**HA030_w2_3_1[6]**)
7. ___ (**HA030_w2_3_1[7]**)
8. ___ (**HA030_w2_3_1[8]**)

PROCEDURE :
HA030_W2_3 HA030_W2_4 HA030_W2_5

HA030_W2_4 [Load proxy]

HA030_W2_5 [HA030_W2_4] [ ] __ % ?

PROCEDURE :
（HA030_W2_3 =5） HA030_W2_6 HA030_W2_7

HA030_W2_6 [Load proxy]

HA030_W2_7 [HA030_W2_6] [ ] __ % ?

HA031 Which household member(s) own the house? [preloaded names of household members] / ____ []

HA032 What share of the house is owned by [preloaded names of household members]? [HA031] _____ %0.00…100.00% [hard check: range 0-100]

PROCEDURE :
If HA030 = 1, skip HA033

HA033 What non-household members own part of your current residence? [circle all that apply] ()

1. Working unit of household member, which household member? [preloaded list][] _ (**HA033_1**)
2. Government indemnificatory housing
3. Child(non- household member) of main respondent or spouse ( ) , which child? (**HA033_2**) [preloaded list]
4. Parent(non- household member) of main respondent or spouse ( ) ____ (**HA033_3**) [preload list]
5. Nonresident other relatives
6. Friends
7. Other

PROCEDURE :
If HA031 does not include respondent or spouse, skip to Next house. HA031

HA034 What is the present market value of your house? Or, what is the present market value of a similar housing unit within its neighborhood? Total price ____ (**HA034_1**) 10,000 Yuan [soft check <10, >500] or unit price ____ (**HA034_2**) 1,000 Yuan/m^2 / [soft check<1, >25]

[CAPI: Skip to HA036 if R answered HA034. If not, ask unfolding brackets. HA034HA036]

PROCEDURE :
If HA034_1 >= 500 or HA034_1 < 10, or HA034_2 < 1 or HA034_2 > 25, ask HA034_check. HA034_1 >= 500 HA034_1 < 10 HA034_2 < 1 HA034_2 > 25 HA034_check

HA034_check I heard that the present market value of your house is [preload HA034_1] 10,000 Yuan, is this correct? [HA034_1]

1. Yes
2. No → go back HA034 HA034

HA035 [IWER: If R is unwilling to answer or does not remember, ask unfolding bracket questions here.]
20,000/50,000/100,000/200,000/500,000 yuan

HA036 Do your household members take out a bank loan to finance the purchase, construct, or decorate this house? ( )

1. Yes
2. No → Skip to HA039 HA039

HA037 What is the outstanding amount of the loans? ____ 10,000 Yuan [soft check >500]

HA038 What is the monthly mortgage payment? ____ Yuan [soft check >20,000]

HA039 How was this housing unit obtained?

1. Purchased from market
2. Purchased from working unit of respondent or spouse ____ (**HA039_1**)[preloaded list] []
3. Purchased by child of main respondent or spouse, which one? ( ) ____ (**HA039_2**) [preload list] []
4. Purchased by parents of main respondent or spouse, of who (main respondent or spouse)? ( ) ( ) ____ (**HA039_3**)
5. Purchased from Other relatives
6. Self-built → Skip to HA041 HA041
7. Inherited, bequeathed, or given ( ) → Skip to HA041 HA041
8. Received home as compensation for demolition of old home, → Skip to HA041 HA041
9. Other → Skip to HA045 HA045

HA040 Was it purchased at market price, subsidized by working unit, or as purchased as economical housing?

1. Market price
2. Subsidized by working unit
3. Economic housing
4. Other

HA041 When did you purchase/build/inherit it? //// ____ 1900...2013 Year
[IWER: Mark the year using four digits. : 4]

HA042 Can you sell the house freely if you want? 1. Yes
2. No, restricted by work unit

PROCEDURE :
If HA039 = 7 , then skip to HA045. HA039 = 7 HA045

HA043 How much of your own money did you spend on the house (including loan-financed)? // ____ 10,000Yuan [soft check<10, >500]

[IWER: Skip to HA045 if R answered HA043. If not, ask unfolding brackets. HA043 HA045]

**PROCEDURE** :<br>IF HA043 >= 500 or HA043 < 10, ask HA043_check. HA043 >= 500 HA043 < 10HA043_check

HA043_check I heard that you had spend [preload HA043] 10,000 Yuan on the house, is this correct? // [HA043]

1. Yes
2. No → go back HA043 HA043

HA044 [IWER: If R is unwilling to answer or does not remember, ask unfolding bracket questions here.]

10,000/20,000/50,000/100,000/200,000 yuan

**PROCEDURE** :<br>If HA040 = 1, then skip to HA051. HA040 = 1 HA051 If HA039 = 6, then skip to HA051. HA039 = 6 HA051

HA045 What would you have to pay if you had paid a market-set price for the same housing? Total price ____ (**HA045_1**) 10,000 Yuan [soft check >500] or unit price ____ (**HA045_2**) 1,000 Yuan/m^2 / [soft check >30]

**PROCEDURE** :<br>If HA045_1 >= 500ask HA045_check. HA045_1 >= 500 HA045_check

HA045_check I heard that you had paid a market-set price [preload HA045_1] 10,000 Yuan for the same housing, is this correct? [HA045_1]

1. Yes
2. No → go back HA045 HA045

HA051 What is the construction area of the house? ____m^2 [soft check<10 or >500]

[Skip to next house until the last house]

[CAPI HA026_W2_1-HA026_W2_14_bracket (1)HA052]

HA051_W2_1 BRANCHPOINT:
IF THE HOUSEHOLD IS A NEW INTERVIEW HOUSEHOLD, SKIP TO HA052 HA052

[ ]

HA052 What is the monthly rental income for all room or houses owned by main respondent or spouse, that you are currently leasing? ( )

1. ____ (**HA052_1**) Yuan/month / [soft check >20,000]
2. Not applicable

HA053 What is the monthly rental income for all room or houses owned by other household members that are currently being leased?

1. ____ (**HA053_1**) Yuan/month /
2. Not applicable

PART 3 Land

[The following questions pertain to your land.]

HA054 Does your household have any collective distributing or rent cultivated land, forest land, pasture and/or pond? (Choose all that apply) ( )

1. Cultivated land
2. Forest land
3. Pasture
4. Pond
5. None → Skip to HA064 HA064

PROCEDURE :
According to all options choosed in HA054 , ask HA055 -HA063 in loop. HA054 HA055 -HA063

HA055 How many mu of [preload answer fromHA054] do you have? [HA054] ____ Mu [soft check > 50]

HA056 How many mu of them are irrigable? ____Mu [hard check cannot be > HA055]

HA057 What is the rent per mu per year you would get if you rent out all your [preload answer from HA054]? [HA054] ____ Yuan per mu per year [soft check < 10; > 4000]

HA058 Did you rent out any of your [preload answer from HA054] in the past year? [HA054]

1. Yes
2. No → Skip to HA061 HA061

HA059 How much [preload answer from HA054] did you rent out the past year? [HA054] Mu [hard check, cannot be > HA055]

HA060 How much rental income did you earn in the past year? [HA054] ____ Yuan

HA061 Did you rent in any [preload answer from HA054] from others (including the collective) in the past year? ( ) [HA054]

1. Yes
2. No → Skip toHA064 HA064

HA062 How much did you rent in the past year? [HA054]____ Mu [soft check > 100]

HA063 How much rent did you pay in the past year? [HA054] ____ Yuan [soft check >20000]

HA064 How much rental income did you earn for any other household assets other than housing or land? (trees, use of fixed capital, durables, or livestock)? ( )

1. ____ (**HA064_1**) Yuan
2. Not applicable

PART 4 Equipments, Consumption durables, and Valuables.

[Show Card 31]

HA065 Do members of your household own the following assets? (Choose all that apply) ( )

For all categories, add [soft check <100 or >30,000] unless other check is written

[For each asset owned by the household] what is the asset s current value? (Yuan) ( )

1. Automobile [soft check <3000, >500,000] _____ (**HA065_1[1]**) 2. Electric Bicycle ____ (**HA065_1[2]**)
3. Motorcycle ____ (**HA065_1[3]**)

4. Refrigerator ____ (**HA065_1[4]**)
5. Washing machine ____ (**HA065_1[5]**)
6. TV ____ (**HA065_1[6]**)
7. Computer ____ (**HA065_1[7]**)
8. Stereo system ____ (**HA065_1[8]**)
9. Video camera ____ (**HA065_1[9]**)
10. Camera ____ (**HA065_1[10]**)
11. Air conditioner ____ (**HA065_1[11]**)
12. Mobile phone ____ (**HA065_1[12]**)
13. Furniture ____ (**HA065_1[13]**)
14. Music instrument ____ (**HA065_1[14]**)
15. Valuable decorations, ornaments ____ (**HA065_1[15]**))
16. Treasures and precious metal (such as gold) () __________ (**HA065_1[16]**)
17. Antiques, valuable paintings and calligraphic work, and other artistic work ____ (**HA065_1[17]**)
18. None

HA065_w2_[1] You just told us that your household owns an automobile/ automobiles, do you or your spouse own the automobile(s)?

1. Yes
2. No

HA065_w2_[16] You just told us that your household own treasures and precious metal, what percentage among the total value is owned by you and/or your spouse? ____ %

[IWER: 10,000 67% 10000 100%]

HA065_w2_[17] You just told us that your household own antiques, valuable paintings and calligraphic work, and other artistic work, what percentage among the total value is owned by you and/or your spouse? ____ %

[IWER: 10000 67% 10000 100%]

HA066 Do members of your household own the following fixed capital assets? How much are the assets worth? (check all that apply) ( )

1. Tractor, current value_____ Yuan ____ (**HA066_1**) [soft check < 1000; > 30; 000]
2. Thresher, current value_____ Yuan () ____ (**HA066_2**) [soft check < 100 or > 10; 000]
3. Tractor tools, current value_____ Yuan ____ (**HA066_3**) [soft check < 100 or > 10; 000]
4. Water pump, current value_____ Yuan () ____ (**HA066_4**) [soft check < 100 or > 10; 000]
5. Processing equipment, current value_____ Yuan ____ (**HA066_5**) [soft check < 100 or > 10; 000]
6. None ____ (**HA066_6**)

HA067 What is the current value of other fixed capital assets used in household production or self-employed activities? ____ Yuan

[IWER: Be sure to ask about fixed capital assets used in all self-employment activities, do not count assets already reported above.]

HA068 Does your household have any other durable or fixed assets worth 500 yuan or more? 500

1. Yes How much are the assets worth? ____ (**HA068_1**) Yuan [hard check > 500] [soft check > 50; 000]
2. No

HA069 Have you lent to other families or individuals and not been repaid by them?

1. Yes
2. No → Skip to HA071 HA071

HA070 What is the total amount of the loans? ______ Yuan [soft check > 500; 000]

HA071 How much interest income from what you lent to others in past year? _____ Yuan [soft check > 50; 000]

HA072 What is the total amount of loans that you are still owing to other families, individuals, or your work unit? (not including mortgage loans) () ____ Yuan [soft check > 500; 000]

[IWER: Skip to HA074, if R answered HA072, If not, ask unfolding brackets HA073. HA072 HA074 HA073]

HA073 [IWER: IfR is unwilling to answer or does not remember, ask unfolding bracket questions here.] 5,000/10,000 /50,000 /100,000 /500,000 yuan

[Intro: Next we will ask your household members, other than the main respondent and spouse, some financial questions.]

[IWER: The names of other household members not including respondent and spouse are preloaded from the cover screen information. For each member, answer the following questions.]

HA074 What is the value of all financial assets of [preload household member name] (includes cash, savings, stocks, funds)? [preload household member name] ( ) ? ____ Yuan [soft check > 1; 000; 000]

HA075 What is the value of all outstanding (unpaid) loans from banks or financial institutions (not including mortgages) of [name]? [name] ( ) ____ Yuan [Soft check > 500; 000]

HA076 How often did the respondent receive assistance in answering section Household assets?

[IWER: If it is answered by a proxy, please record the respondents reaction.]

1. Never
2. A few times
3. Most or all of the time

HB INDIVIDUAL ASSETS

[IWER: Please conduct sections HB and HC when the main respondent and his/her spouse are at home. Dont allow a proxy to complete the entire sections. HB HC]

PART 1 Financial Assets

[The following questions pertain to your financial asset.]

[IWER reminder: make sure others are not present, IWER read following instructions: the following questions pertain to your financial asset, the answers to these questions will be kept strictly confidential and will be used for research purposes only.]

HC001 How much cash is held by you and your spouse at home? （） _____ Yuan [soft check > 50; 000 or < 100]
[IWER: Skip to HC003 if R answered HC001 and HC001 ≠ 0. If not, ask unfolding brackets. HC001 0 HC003]

HC002 [IWER: If R is unwilling to answer or does not remember, ask unfolding bracket questions here.] 500 /1,000 /2,000 /5,000 /10,000 yuan

HC003 Of which, how much do you own (if jointly owned with spouse then count 50%)? （ 50%） ____ (**HC003_1**) Yuan [soft check > 50; 000] or __ (**HC003_2**) % [range 0 − 100]

[CAPI: prompt for HC004 - HC019: IWER: for deposit, bondsstocks, and funds, only include assets legally in his/her name. HC004 - HC019]

HC005 What is the total amount of deposits you are currently holding in financial institutions（eg: bank）? () ______ Yuan [soft check > 500; 000 or < 100]

**PROCEDURE** :
Skip to HC007 if R answered HC005. If not, ask unfolding brackets. HC005 HC007]

HC006 [IWER: If R is unwilling to answer or does not remember, ask unfolding bracket questions here.]
2,000/10,000/50,000 /100,000/500,000 yuan

HC007 Do you have any government bonds (e.g. Treasury bills) in your name? （ ） ()
1. Yes
2. No → go back HC010 HC010

HC008 What is the total face value of government bonds that you are currently holding? () ?_____ Yuan [soft check > 50; 000]

**PROCEDURE** :
Skip to HC0010 if R answered HC008. If not, ask unfolding brackets HC009. HC008 HC0010HC009

HC009 [IWER: If R is unwilling to answer or does not remember, ask unfolding bracket questions here.] 10,000 /50,000 /100,000 /200,000 /500,000 yuan

HC010 Have you held any stocks in the past year in your name, excluding the equity or stock of your work unit? ( )

1. Yes
2. No → Skip to HC015 HC015

HC013 What is the present market value of all the stocks you are currently holding? ___ Yuan [soft check > 200; 000]

**PROCEDURE** :<br>Skip to HC015 if R answered HC013. If not, ask unfolding brackets. HC013 HC015

HC014 [IWER: If R is unwilling to answer or does not remember, ask unfolding bracket questions here.] 10,000 /50,000 /100,000 /200,000 /500,000 yuan

HC015 Have you held any funds in your name in the past year? ( )

1. Yes
2. No → Go to PROGRAM before HC020 HC020

HC018 What is the present market value of all the mutual funds you are currently holding? Yuan [soft check > 200; 000]
[IWER: Skip to HC020 if R answered HC018. If not, ask unfolding brackets. HC018HC020]

HC019 [IWER: If R is unwilling to answer or does not remember, ask unfolding bracket questions here.] 10,000 /50,000 /100,000 /200,000 /500,000 yuan

**PROCEDURE** :<br>if HC005 = 0 and HC007 = 2 and HC010 = 2 and HC015 = 2, then skip HC020.<br>HC005 = 0HC007HC010HC015 2 HC020

HC020 What percentage of the deposits, bonds, stocks, and funds held in your name is fully controlled by you and not your spouse? (%) ____ 0...100 % [hard check ≥ 0, ≤ 100]

HC021 Do you have any other deposits, bonds, stocks, or funds that belong to you but which are held in a persons name other than you or your spouse?

1. Yes
2. No → Skip to HC023 HC023

HC022 What is the value of such assets? ______ yuan [soft check > 200; 000]

HC023 Other than income you have already told me about, did you receive any other income from other investments in past year?

1. Yes
2. No → Skip toHC027 HC027

[IWER:. :]

HC024 How much did you receive altogether from other investments in the past year? __ Yuan [soft check > 200; 000]

**PROCEDURE** :<br>Skip to HC027 if R answered HC024. If not, ask unfolding brackets HC025. HC024 HC027HC025]

HC025 [IWER: If R is unwilling to answer or does not remember, ask unfolding bracket questions here.] 1,000 /5,000 /10,000 /20,000 /50,000 yuan

HC026 What percentage of the other investments is owned jointly with your spouse? __ 0…100% [hard check ≤ 0, ≥ 100]

HC027 Do you have public housing funding?

1. Yes
2. No → Skip to HC030 HC030

HC028 What is the total amount of money in your public housing fund? ______ Yuan [soft check > 100; 000]

**PROCEDURE** :<br>Skip to HC030 if R answered HC028. If not, ask unfolding brackets HC029. HC028 HC030HC029

HC029 [IWER: IfR is unwilling to answer or does not remember, ask unfolding bracket questions here.] 5,000/10,000 /50,000 /100,000 /200,000 yuan

HC030 Do you have Jizikuan that your work unit or other work units have collected from you and are still holding (Jizikuan is fund individuals provided to the work unit for the purpose of investment, building apartments, etc.)?

1. Yes
2. No → Skip to HC033 HC033

HC031 What is the amount of your jizikuan? ______Y uan [soft check > 200; 000]

**PROCEDURE** :<br>Skip to HC033 if R answered HC031. If not, ask unfolding brackets HC032. HC031 HC033 HC032

HC032 [IWER: IfR is unwilling to answer or does not remember, ask unfolding bracket questions here.] 5,000/10,000 /50,000 /100,000 /200,000 yuan

HC033 Do you have any unpaid salary that your work unit still owes you?
1. Yes
2. No → Skip to HC036 HC036

HC034 What is the amount of your unpaid salary? ______ Yuan [soft check > 100; 000]

**PROCEDURE** :<br>Skip to HC036 if R answered HC034. If not, ask unfolding brackets HC035. HC034 HC036 HC035

HC035 [IWER: IfR is unwilling to answer or does not remember, ask unfolding bracket questions here.] 5,000/10,000 /50,000 /100,000 /200,000 yuan

HC036 Have you participated in any rotating savings and credit association during the past year?
1. Yes
2. No → Skip to HD001 HD001

HC037 What is the total amount of funds that you are still obligated to pay to the rotating savings and credit association ______ yuan

PART 2 DEBTS

[The following questions pertain to your debt.]

HD001 What is the total amount of loan that you havent repaid yet (not including loans for house)? ( ) ______ Yuan [soft check > 500; 000]

HD002_W2_1 What is the total amount of personal loan that you havent repaid yet? _____ Yuan [soft check > 500; 000]

PROCEDURE :
Skip to HD003 if R answered HD001 and HD002_W2_1. If not, ask unfolding brackets HD002 and HD002_W2. HD001HD002_W2_1 HD003 HD002 HD002_W2

HD002 [IWER: IfR is unwilling to answer or does not remember, ask unfolding bracket questions here.] 5,000/10,000 /50,000 /100,000 /500,000 yuan

HD002_W2 [IWER: IfR is unwilling to answer or does not remember, ask unfolding bracket questions here.]
5,000/10,000 /50,000 /100,000 /500,000 yuan

HD003 What is the amount of your credit card balance? _____ Yuan [soft check > 50; 000]（ 0 ）

PROCEDURE :
Skip to HD005 ifR answered HD003 . If not, ask unfolding brackets HD004. HD003 HD005 HD004

HD004 [IWER: If R is unwilling to answer or does not remember, ask unfolding bracket questions here.] 500/1,000 /5,000 /10,000 /50,000 yuan

HD005 Have you ever inherited anything
1. Yes
2. No → Skip toHD012 HD012

HD006 How much in total have you inherited ______ Yuan （） [soft check > 200; 000]

PROCEDURE :
Skip to HD008 if R answered HD006. If not, ask unfolding brackets HD007. HD006 HD008 HD007]

HD007 [IWER: IfR is unwilling to answer or does not remember, ask unfolding bracket questions here.] 5,000/10,000 /50,000 /100,000 /500,000 yuan

HD008 From whom you inherited(Choose all that apply) （） 1. Parents
2. Parents-in-law

3. Children
4. Relatives
5. Others

HD009 When did the largest inheritance occur ____ 1900...2013 Year
[IWER: Mark the year using four digits. :]

HD010 What was the value of that inheritance? ______ Yuan [soft check > 200; 000]

PROCEDURE :
Skip to HD012 if R answered HD010. If not, ask unfolding brackets HD011. HD010 HD012 HD011

HD011 [IWER: IfR is unwilling to answer or does not remember, ask unfolding bracket questions here.] 5,000/10,000 /50,000 /100,000 /500,000 yuan

HD012 How often did the respondent receive assistance in answering section H ASSETS?
[IWER: If it is answered by a proxy, please record the respondents reaction.]
1. Never
2. A few times
3. Most or all of the time

I HOUSING CHARACTERISTICS

**PROCEDURE** : Only main respondent answer I001 - I026. I001 - I026

I001 What is the construction area of your residence? ____ m^2 [soft check: < 10 or > 500]

I002 What is the total housing land area? ? ( ) ____ m^2 [soft check
< 10, > 1000]
[0]

I003 Is your residence used for business as well?
1. Yes
2. No

I004 What type of structure is this building? Is it concrete and steel, mixed structure, bricks and wood, wood, bamboo, grass or other?
1. Concrete and steel
2. Bricks and wood
3. Mixed structure
4. Wood, bamboo, grass
5. Woolen felt
6. Sheet iron
7. Cave dwelling
8. Tent
9. Adobe
10. Other

I005 When was this house built? ____ Year

I005_1 If R is unclear about year, please choose among following items.
1. 0 − 5 years 0 − 5
2. 5 − 10 years 5 − 10
3. 10 − 20 years 10 − 20
4. 20 − 30 years 20 − 30
5. 30 − 40 years 30 − 40
6. more than 40 years 40

I006 Is the building one story or multi-level building? 1. One-story building → skip to I007
2. Multi-story building → skip to I008

I007 Is the story independent or compound?

1. Independent story
2. Compound

I008 Which story is this building on? ____

**PROCEDURE**: IfI008 > 1, ask I009. I008 > 1I009

I009 Does it has elevator

1. Yes
2. No

I010 Are there any handicapped facilities (e.g., non-stair ramp)? ( )

1. Yes
2. No

**PROCEDURE**: IfI010 = 2, ask I011. I010 = 2I011

I011 How many steps had to be climbed to get to the main entrance of the households flat? [IWER: Do not count steps if an elevator is available.]

1. 0 0
2. 1 to 5 1 – 5
3. 6 to 15 6 – 15
4. 16 to 25 16 – 25
5. More than 25 25

I012 How many bedrooms, living rooms, bathrooms, and kitchens are there in your residence? __ (**I012_1**) bedrooms __ (**I012_2**) living rooms __ (**I012_3**) toilets ( ) __ (**I012_4**) kitchens __ (**I012_5**) balcony [soft check: > 20]

**PROCEDURE**: Ask I013 if no toilets in the answer to I012. I012 I013

I013 How far is the nearest toilet to your house? ____ (**I013**) meters [soft check: > 500]

I014 What is the type of toilet?

1. Toilet without a seat
2. Toilet with a seat

I015 Is the toilet flushable?

1. Yes
2. No

I016 Does your residence have electricity?

1. Yes
2. No

I017 Does your residence have running water?

1. Yes
2. No

I018 Is there in-house shower or bath facility? What type? 1. Hot water provided

2. Water heater installed by the household
3. No

I019 Does your residence have coal gas or natural gas supply? 1. Yes

2. No

I020 Does your residence have heating? ( ) 1. Yes → skip to I022 I022

2. No

I021 What is the main heating energy source? 1. Solar

2. Coal
3. Natural gas
4. Liquefied Petroleum Gas
5. Electric
6. Crop residue/Wood buring
7. Other

I022 What is the main source of cooking fuel?

1. Coal
2. Natural gas
3. Marsh gas
4. Liquefied Petroleum Gas
5. Electric
6. crop residue/Wood burning
7. other

I023 Does your residence have a telephone connection?

1. Yes
2. No

I024 Does your residence have broad-band internet connection?

1. Yes
2. No

I025 [Interviewer records it] [] How clear and tity is in this household

1. Excellent
2. Very clear
3. Clear
4. Fair
5. Poor
6. Not applicable

I026 [Interviewer records it] [] How is the temperature in this household

1. Very hot
2. Hot
3. Bearable
4. Cold
5. Very cold
6. Not applicable

J INTERVIEWER OBSERVATION

[IWER. This section is about your observations during the interview and should be filled out after each completed individual interview.]

J001 Were any third persons, except proxy respondents, present during (parts of) the interview with [Respondent Name]? (circle all that apply) ( )

1. Nobody → Skip to J003 J003
2. Spouse or partner
3. Parent or parents
4. Child or children
5. Other relatives
6. Other persons present

J002 Did these persons intervene during the interview? / 1. Yes, often

2. Yes, occasionally
3. No

J003 How would you describe the willingness of [Respondent Name] to answer?

1. Very good → Skip to J005 J005
2. Good → Skip to J005 J005
3. Fair → Skip to J005 J005
4. Bad → Skip to J005 J005
5. Good in the beginning, got worse during the interview
6. Bad in the beginning, got better during the interview → Skip to J005 J005

J004 Why did the respondents willingness to answer get worse during the interview? (Choose all that apply) ( )

1. The respondent was losing interest
2. The respondent was losing concentration or was getting tired
3. Other, please specify _______

J005 Did [Respondent Name] ask for clarification on any questions?

1. Never
2. Almost never
3. Now and then

4. Often
5. Very often
6. Always

J006 Overall, did you feel that [Respondent Name] understood the questions?
1. Never
2. Almost never
3. Now and then
4. Often
5. Very often
6. Always

J007 Did the respondent need any help reading the showcards during the interview?
1. Yes, due to sight problems
2. Yes, due to literacy problems
3. No

J008_W2 During the visit, the respondents primarily use which of the following languages?
1. Mandarin → J009_W2
2. Dialect → J010_W2

J009_W2 What about the level of mandarin of the respondent?
1. Very Good → J010_W2 - J011_W2
2. Good → J010_W2 - J011_W2
3. Fair → J010_W2 - J011_W2
4. Mixed with Some Local Accent → J011_W2 5. Mixed with Lot of Local Accent → J011_W2

J010_W2 During the visit, the respondents primarily use which dialect? ____ _____
[( ) ( )]

J011_W2 During the visit, the respondents mixed with which local accent? _____ _____
[( ) ( )]

**PROCEDURE** : If Proxy, ask J012_W2 - J013_W2. J012_W2 - J013_W2

J012_W2 Name of Proxy ______

J013_W2 Relationship between proxy and respondent

1. Spouse
2. Mother
3. Father
4. Mother-in-law /
5. Father-in-law /
6. Sibling
7. Brother-in-law, sister-in-law /
8. Child
9. Spouse of child
10. Grandchild
11. Other relative
12. Helper or other non-relative
