# Supplementary material for: Association of total sleep duration variability with risk of new stroke in the middle-aged and elderly Chinese population
Source: BMC Neurol. 2024 Jun 25;24:217. doi: 10.1186/s12883-024-03727-8 (PMC11197293; doi:10.1186/s12883-024-03727-8)
Supplement: Supplementary file 3 — Supplementary Material 3 [file 12883_2024_3727_MOESM3_ESM.pdf]

China Health and Retirement Longitudinal Study

Followup Questionnaire 2015

2015

June 2015

China Center for Economic Research
Institute of Social Science Survey
Peking University

This page intentionally left blank

Contents

B DEMOGRAPHIC BACKGROUNDS **1**

C FAMILY **13**

C1 RARENT, CHLIDREARING AND SIBLING INFORMATION 13

CA PARENT INFORMATION 13

CB CHILDREARING INFORMATION 25

CC SIBLINGS 31

C2 TIME TRANSFER AND TRANSFERS 37

CD TIME TRANSFER 37

CE TRANSFERS 40

CF TIME SPENT PROVIDING CARE 46

A HOUSEHOLD MEMBER 48

D HEALTH STATUS AND FUNCTIONING **51**

DA HEALTH STATUS 52

PART I: GENERAL HEALTH STATUS AND DISEASE HISTORY 52

PART II: LIFESTYLE AND HEALTH BEHAVIORS 67

DB FUNCTIONAL LIMITATIONS AND HELPERS 74

DC COGNITION & DEPPRESSION 89

E HEALTH CARE AND INSURANCE **97**

PART I MEDICAL INSURANCE 97

PART II HEALTH CARE COSTS AND UTILIZATION 102

F WORK, RETIREMENT AND PENSION **121**

FA JOB STATUS 123

FC AGRICULTURE WORK 133

FARM EMPLOYED 133

HOUSEHOLD AGRICULTURAL WORK 133

FD EMPLOYED 136

FE QUESTIONS ABOUT LABOR SUPPLY 142
FF QUESTIONS ABOUT WAGES 142
FG Fringe Benefits 145
FH NON-FARM SELF-EMPLOYED AND UNPAID FAMILY BUSINESS 145
FJ SIDE JOB (EMPLOYED OR SELF-EMPLOYED) ( ) 149
FK UNEMPLOYMENT AND JOB SEARCH ACTIVITIE . 149
FL LAST JOB 151
FM RETIREMENT 156
FN PENSION INSURANCE 166
Part 1 ( ) 167
Part 2 ( ) 172
Part 3 175
Part 4 ( ) 181
Part 5 182
Part 6 183
Part 7 ( ) 184
Part 8 187

G&H INCOME, EXPENDITURES AND ASSETS 191

G2 HOUSEHOLD INCOME AND EXPENDITURES 191
PART 1 Household Wage Income and Individual-based transfers 191
PART 2 HOUSEHOLD AGRICULTURAL INCOME AND EXPENDITURE 195
PART 3 Self-employed Activities 198
PART 4 HOUSEHOLD PUBLIC TRANSFER INCOME 199
PART 5 HOUSEHOLD LIVING EXPENDITURE 199
HA HOUSEHOLD ASSETS 202
PART 1 Current Residence 202
PART 2 Other Residences 208
PART 3 Land 212

PART 4 Equipments, Consumption durables, and Valuables. 214
HB INDIVIDUAL ASSETS 215
PART 1 Financial Assets 215
PART 2 DEBTS 220

I HOUSING CHARACTERISTICS 223

This page intentionally left blank

B DEMOGRAPHIC BACKGROUNDS

[**NOTE**: ONLY THOSE WHO WERE BORN BEFORE JULY 1, 1970 AND THEIR SPOUSES WILL BE INTERVIEWED 1970 7 1]

Type of Interview R	
XRType = REIW	This is a reinterview R
XRType = NEWIW	This is a new interview R

BA000_W2_1 We record your name is [name], is it right? []

[**PROCEDURE**: Preload the name of R]

1. Yes → Skip to BA000_W2_3 BA000_W2_3
2. No

BA000_W2_2 Whats your name? ___________

BA000_W2_3 Interviewer record Rs gender. .

[**PROCEDURE**:Preload Rs gender]

1. Male
2. Female

BA004_W3 Whats your date ofbirth on ID card or Household register?

1. _____ (**BA004_W3_1**) Year _____ (**BA004_W3_2**) Month _____ (**BA004_W3_3**) Day
2. Does not have Hukou or ID card

[IWERThe year must be a number in the range [1900 – 2015]. Mark the year using four digits. Take down the month as its actual number. For example, write January as 1 not 01, December as 12. If do not remember month and day, fill 0 [1900 — 2015]. 4 . 1 1 01,12 12. 0]

BA002 Whats your actual date ofbirth?

1. The same as on Household or ID card
2. _____ (**BA002_1**) Year _____ (**BA002_2**) Month _____ (**BA002_3**) Day

[IWER:The year must be a number in the range [1900 – 2015]. Mark the year using four digits. Take down the month as its actual number. For example, write January as 1 not 01, December as 12. If do not remember month and day, fill 0 [1900 — 2015]. 4 . 1 1 01,12 12. 0]

PROCEDURE :

If the person does not know the date ofbirth, BA002 = .d or BA002 = .r, skip BA003. BA002 BA003

BA003 Is your answer to BA002 based on the solar or the lunar calendar?

() ()

1. Solar calendar ( )
2. Lunar calendar ( )

[INTRO: Next are some questions about your birth place, some changes in your housing location, your Hukou and education]

PROCEDURE :

If XRType = REIW, then preload the information of address in last wave, if missing, then ask BB000_W3. , BB000_W3

If XRType = NEWIW, go to BB001_W3. BB001_W3

BB000_W3 Whats your address in [the time of last interview]? []

1. [Address in last interview]
2. Other

   _____ (**BB000_W3_a_1**) province/city/county //
   _____ (**BB000_W3_a_2**) township //
   _____ (**BB000_W3_a_3**) village/neighborhood /
3. Abroad

[IWER: Confirm the permanent address with respondents, and fill in the changed. If do not know the name, please fill in other .]

[IWER: county/city includes county-level administrative units/county-level city/district ///]

BB000_W3_1 What is the type of the address?

1. Family housing
2. Nursing home
3. Hospital
4. Other, please specify ______ (**BB000_W3_1_1**)

BB000_W3_2 Was it village or city/town?

1. Main city zone

2. Combination zone between urban and rural areas
3. The town center
4. ZhenXiang area
5. special area
6. Township central
7. Village

BB001_W3 Whats your address now?

1. [Preload address]
2. Other
   _____ (**BB001_W3_a_1**) province/city/county //
   _____ (**BB001_W3_a_2**) township //
   _____ (**BB001_W3_a_3**) village/neighborhood /
3. Abroad

[IWER: Confirm the permanent address with respondents, and fill in the changed. If do not know the name, please fill in other .]

[IWER: county/city includes county-level administrative units/county-level city/district ///]

BB001_W3_1 What is the type of the address?

1. Family housing
2. Nursing home
3. Hospital
4. Other, please specify ______ （**BB001_W3_1_1**）

BB001_W3_2 Was it village or city/town?

1. Main city zone
2. Combination zone between urban and rural areas
3. The town center
4. ZhenXiang area
5. special area
6. Township central
7. Village

PROCEDURE :

If BB001_W3 ! = 1 or XRType = NEWIW, then skip to BB003. Otherwise, skip to BB005_W3_1. BB001_W3 ! = 1 XRType = NEWIW, BB003. , BB005_W3_1

BB003 When did you first live in permanent addresss county/city/district? // _____
1900..2015 Year
[IWER: Mark the year using four digits 4]

BB004 When you first moved to permanent address, did you live in the same village/community as you currently do? /

1. Yes → skip to BB005_W3_1 BB005_W3_1
2. No

BB005 In what year did you first live in permanent addresss village/community? / _____
1900..2015 Year
[IWER: Mark the year using four digits 4]

BB005_W3_1 Would you change your address in next two years? 2 , ?

1. Yes
2. No → skip to BB001 BB001

BB005_W3_2 Where would your new address?

1. I am not sure
2. Another house in the same village/community /, 3. Other

_____ (**BB005_W3_2_1**) province/city/county //
_____ (**BB005_W3_2_2**) township //
_____ (**BB005_W3_2_3**) village/neighborhood /

BB001 Where were you born?

1. The same as permanent address
2. Another village/neighborhood in permanent address s county/city/district ///
   _____ (**BB001_ 1**) township //
   _____ (**BB001_2**) village/neighborhood /
3. Other
   _____ (**BB001_3**) province_city_county/city/district _ _ // ___ (**BB001_4**) township //
   _____ (**BB001_5**) village/neighborhood /
4. Abroad

[IWER: county/city includes county-level administrative units/county-level city/district ///]

PROCEDURE :
If ZBC001 = nonresponse, answer BC002_W3_1 & BC002_W3_4, then skip to BD001_W2_4. ZBC001 = nonresponse BC002_W3_1 BC002_W3_4 BD001_W2_4

BC001_W3_1 We record your hukou type is [ZHukou Type], right? []

1. Yes → SKIP TO BC001_W3_3 BC001_W3_3
2. No

BC001_W3_2 Whats your hukou type in last interview? 1. Agricultural Hukou

2. Non-agricultural Hukou
3. Unified Residence Hukou
4. Do not have Hukou

BC001_W3_3 Whats your Hukou location in last interview? [ ]

1. The same as permanent address
2. Other:

_____ (**BC001_W3_3_1**) province_city_county/city/district _ _ // _ (**BC001_W3_3_2**) township //

_____ (**BC001_W3_3_3**) village/neighborhood /

3.

BC002_W3 Since [ZIWTime], have you Hukou type and Hukou location changed? [ZIWTime]

1. Only Hukou type has changed
2. Only Hukou place has changed
3. Both Hukou type and place have changed 4. Both havent changed

PROCEDURE :
If BC002_W3 = 1 or = 3, then answer BC002_W3_1 - BC002_W3_3. BC002_W3 = 1 = 3 BC002_W3_1 BC002_W3_3

BC002_W3_1 What is your current Hukou status?

1. Agricultural Hukou
2. Non-agricultural Hukou
3. Unified Residence Hukou
4. Do not have Hukou

BC002_W3_2 What is the reason that you changed to the agricultural Hukou?

1. Go to school
2. Marriage
3. Employment
4. Land is acquired by the government

5. Migration of the whole village
6. _____ (**BC002_W3_2_1**)

BC002_W3_3 When did your type of Hukou change? ___ (**BC002_W3_3_1**) Year _ (**BC002_W3_3_2**) Month

PROCEDURE :
If BC002_W3 = 2 or = 3, then answer BC002_W3_4 - BC002_W3_6. BC002_W3 = 2 = 3 BB005_w3_4 BC002_W3_6

BC002_W3_4 Whats your current location of Hukou?
1. Permanent address
2. Other
   _____ (**BC002_W3_4_1**) province_city_county/city/district _ _ // _ (**BC002_W3_4_2**) township //
   _____ (**BC002_W3_4_3**) village/neighborhood /
3. Abroad

BC002_W3_5 Why did your location of Hukou change?
1. Go to school
2. Marriage
3. Employment
4. Land is acquired by the government
5. Migration of the whole village

BC002_W3_6 When did your location of Hukou change? _____ (**BC002_W3_6_1**) Year __ (**BC002_W3_6_2**) Month

BD001_W2_4 Have your highest level of education changed from last wave? If so, whats the highest level of education your have attained now?(not including adult education) ( )
1. No formal education (illiterate) ( )
2. Did not finish primary school
3. Sishu/home school
4. Elementary school
5. Middle school
6. High school
7. Vocational school ( )
8. Two-/Three-Year College/Associate degree
9. Four-Year College/Bachelors degree
10. Masters degree
11. Doctoral degree/Ph.D.

12. No changing

PROCEDURE :
If BD001_W2_4 = 1/2/3 ask BD001_W3_1, otherwise ask BD002_W3.
BD001_W2_4 = 1/2/3 BD001_W3_1 BD002_W3

BD001_W3_1 Are you literate?
1. Yes
2. No

BD002_W3 How many years at school after your highest level of education changed from last wave? （0） ______

BD006 At what age did you finish schooling? ______1...120 years old
[IWERIt asks age when R finishes schooling, not age when R finished elementary school /]

BD007_W2_1 Have you attended school for adult education? （ ）? （）
1. None
2. TV University
3. Night School
4. Zikao (examinations for self-taught students)
5. Hanshou/Correspondence course/Distance learning
6. Literacy course
7. Accelerated education course
8. Other (explain:) ______(**BD007_W2_1_1**)

BD008_W2_1 How many years did you spend in adult education? _____ year

BD009_W2_1 Did you get a diploma or degree from the adult education program you attended?
1. Yes
2. No → BD012

BD010 When did you receive the diploma?
_____ 1900...2015 year

BD011 What is the highest adult schooling degree? 1. Vocational school
2. Two/Three Year College / Associate degree
3. Four Year College / Bachelors degree

4. Others

BD012 Since [ZIWTime], have you participated in vocational and technical training? [ZIWTime]

1. Yes
2. No → to BE001

BD012 How many times have your participated? _____ Times

BD012 Since [ZIWTime], how many years have you participated? [ZIWTime] ______ Years

[Show Card 1]

BE001 RMaritalStatus: What is your marital status?
[IWER: common-law marriage is considered as married]

1. Married with spouse present
2. Married but not living with spouse temporarily for reasons such as work
3. Separated ( )
4. Divorced
5. Widowed
6. Never married
7. Cohabitated

PROCEDURE :
If BE001 ! = 6 XRType = 2, ask BE003_W2_1 to BE009. BE001 ! = 6 XRType = 2 BE003_W2_1 BE009

[INTRO: Many people have more than one marriage through whole life. Please bear me a few more questions on this]

BE003_W2_1 How many times have you been married since last interview? _ times

PROCEDURE :
If BE003_W2_1 > 0, ask BE009. BE003_W2_1 > 0 BE009

BE009 When was your most recent marriage? ____ 1900…2015 (**BE009_1**) year _____ 0…12 (**BE009_2**) month
[: 4 . 1 10112 12. 0]

PROCEDURE :

If HHseperated = 0, skip to BF008. HHseperated = 0 BF008 If BE001 = 1/2/6/7, skip to BF008. BE001 = 1/2/6/7 BF008

If ZBF001 = nonresponse, answer BF001. ZBF001 = nonresponseBF001

If ZBF002_1 = nonresponse, answer BF002. ZBF002_1 = nonresponseBF002 If BF002_2 ! = nonresponse, answer BF003. BF002_2 ! = nonresponseBF003

[IWER: Id like to ask you a few questions about your current (or most recent) spouse/partner .]

[INTRO: An important part of this study is understanding how people make decisions during different stages of life, we also need to a general age range for your spouse]

BF001 What is your spouse/partners Chinese Zodiac sign?

[.] [Show Card 1]

1.
2.
3.
4.
5.
6.
7.
8.
9.
10.
11.
12.

BF002 When was your spouse/partner born?

[4 . 1 10112 12. 0] _____ 1900...2015 (**BF002_1**) year ________________________________ 0...12 (**BF002_2**) month

BF003 Soloar calendar or lunar calendar? ( ) ( )

1. Solar calendar ( )
2. Lunar calendar ( )

BF004 What is the highest level of education your spouse/partner has attained? (not including adult education) ( )

1. No formal education (illiterate) ( )
2. Did not finish primary school but capable of reading and/or writing
3. Sishu/home school
4. Elementary school

5. Middle school
6. High school
7. Vocational school ( )
8. Two-/Three-Year College/Associate degree
9. Four-Year College/Bachelors degree
10. Masters degree
11. Doctoral degree/Ph.D.

BF004_W3_1 Are your spouse literate?

1. Yes
2. No

**PROCEDURE**:<br>If BE001 = 3, ask BF005. BE001 = 3, BF005

BF005 When did you separate?

[IWERMark the year using four digits. Take down the month as its actual number. For example, write January as 1 not 01, December as 12. If do not remember month and day, fill 0 4 . 1 101,12 12. 0]_____ 1900...2015 (**BF005_ 1**) year 0...12 (**BF005_2**) month

**PROCEDURE**:<br>If BE001 = 4, ask BF006. BE001 = 4 BF006

BF006 When did you divorce?

[IWERMark the year using four digits. Take down the month as its actual number. For example, write January as 1 not 01, December as 12. If do not remember month and day, fill 0 4 . 1 101,12 12. 0]_____ 1900...2015 (**BF006_ 1**) year _______ 0...12 (**BF006_2**) month

BF006_W2_1 What is the reason of your divorce?

1. Emotional feud
2. Live in different places
3. In order to facilitate the property purchase transactions 4. Other ______ (**BF006_W2_1_1**)

BF006_W2_2 Divided assets, including real estate, how much is yours and how much is your ex-wife/husband? ( ) ______ (**BF006_W2_2_1**) /______ (**BF006_W2_2_2**)

BF006_W2_3 Do you have any infancy children? Owed to whom?

1. Myself
2. My last spouse /
3. None → Skip to BF007

BF006_W2_4 How much money you or your ex-wife/husband pay for upbringing the children? / _____ Yuan

**PROCEDURE** :<br>If BE001 = 5, Ask BF007 BE001 = 5, BF007

BF007 When did your spouse pass away?

_____ 1900...2015 (**BF007_ 1**) year 0...12 (**BF007_2**) month [IWERMark the year using four digits. Take down the month as its actual number. For example, write January as 1 not 01, December as 12. If do not remember month and day, fill 0 4 . 1 101,12 12. 0]

BF008 How often did the respondent receive assistance in answering section Demographics?

[IWER: If it is answered by a proxy, please record the respondents reaction]

1. Never → Skip to next module
2. A few times → Skip to next module
3. Most or all of the time → Skip to next module 4. The section was completed by a proxy respondent

BF009 What is your relationship to R?

[IWER: What is the proxys relationship to R? If unknown, please ask the proxy .]

1. Spouse
2. Mother
3. Father
4. Mother-in-law /
5. Father-in-law /
6. Sibling
7. Brother-in-law, sister-in-law /
8. Child
9. Spouse of child
10. Grandchild
11. Other relative
12. Helper or other non-relative
13. Others

BF010 What is the main reason for proxy (the respondent is absent)?

1. The respondent has serious physical handicaps 2. The respondent has serious mental handicaps

3. The respondent has rejected this interview

4. Other ______(**BF010_ 1**)

C FAMILY

Type of Interview R	
XRType = REIW	This is a reinterview R
XRType = NEWIW	This is a new interview R

[IWER: Section CA and Section CC and Section CG and Section A are asked for each Rs and Section CB is only asked for Family R. If Family R is not in or couldnt answer the questions, spouse can be a proxy, but other people are not allowed. CA/CC/CG/A CBCDCE CF . .]

[Section CA and section CC are asked for Family R first, then based on CC005_W3_1, ask questions about spouses parents and siblings. Parents of Family R are encoded as: 1 biological father, 2 biological mother, 3 stepfather, 4 stepmother, 5 adopted father, 6 adopted mother. Parents of spouse are encoded as: 7 biological father, 8 biological mother, 9 stepfather, 10 stepmother, 11 adopted father, 12 adopted mother. Answers of spouses siblings information are recorded with variables with postfix S. CA CC CC005_W3_1. 1 2 3 4 5 6 . 7 8 9 10 11 12 . S.]

C1 RARENT, CHLIDREARING AND SIBLING INFORMATION

[In the following three parts: CA parent information, CB childrearing information and CC sibling information, Id like to ask you some questions about your family. CA CB CC .]

CA PARENT INFORMATION

[Id like to ask you some questions about your parents. .]

PROCEDURE

If this is a REIW, ask the following questions; if this is a NEWIW, then skip to CA000_W3_1. , CA000_W3_1.

PROCEDURE

If ZCA001_1_ $\neq$ 1, skip to procedure before CA001_W3_0

PROCEDURE

If ZCA002_inf_1_ is missing, skip CA000_W3_0

CA000_W3_0 Is [Name] your biological father?

1. Yes → Skip to CA000_W3_2 CA000_W3_2
2. The name is wrong, and other information is right → Skip to CA000_W3_5_1 CA000_W3_5_1
3. The name and other information are wrong

[Preload fathers names. .]

CA000_W3_5 Whats the name of your biological father?

1. Name
3. Name
5. Name
7. Name
9. Name
11. Name
97. Other

PROCEDURE
If CA000_W3_5 ≠ 97, skip to CA000_W3_2

CA000_W3_5_1 Whats the name of your biological father? ________

PROCEDURE
If CA000_W3_0 = 2, skip to CA000_W3_2.

CA000_W3_1 What was your biological fathers birth year? 1850...2014

[IWER:Mark the year using four digits. 4 .]

CA000_W3_2 Is your biological father still living?

1. Yes
2. No

PROCEDURE
If this is a REIW and CA000_W3_2 = 1, skip to procedure before CA001_W3_0; if this is a NEWIW and CA000_W3_2 = 1, skip to CA000_W3_5_1 and then skip to procedure before CA001_W3_0. CA000_W3_2 = 1 CA001_W3_0 , CA000_W3_2 = 1 CA000_W3_5 1 CA001_W3_0 .

CA000_W3_3 When did he pass away? ?

1. Year ____1850...2014 (**CA000_W3_3_1**)
2. Age ___0...120 (**CA000_W3_3_2**)

[IWER:Mark the year using four digits. 4 .]

CA000_W3_4 What was the main cause of your biological fathers death? [( ) , ( ) , .]

**PROCEDURE**<br>If this is a REIW, ask the following questions; if this is a NEWIW, then skip to CA001_W3_1. , CA001_W3_1.

**PROCEDURE**<br>If ZCA001_2_ ≠ 1, skip to procedure before CA002_W3_1.

**PROCEDURE**<br>If ZCA002_inf[2] is missing, skip CA001_W3_0

CA001_W3_0 Is your biological mother [Name]? 1. Yes → Skip to CA001_W3_2 CA001_W3_2

2. The name is wrong, and other information is right → Skip to CA001_W3_5_1 CA001_W3_5_1
3. The name and other information are wrong

[Preload mothers names. .]

CA001_W3_5 Whats the name of your biological mother?

2. Name
4. Name
6. Name
8. Name
10. Name
12. Name
97. Other

**PROCEDURE**<br>If CA001_W3_5 ≠ 97, skip to CA001_W3_2.

CA001_W3_5_1 Whats the name of your biological mother? ________

**PROCEDURE**<br>If CA001_W3_0 = 2, skip to CA001_W3_2.

CA001_W3_1 What was your biological mothers birth year? _____1850...2014

[IWER:Mark the year using four digits. 4 .]

CA001_W3_2 Is your biological mother still living?

1. Yes
2. No

PROCEDURE

If this is a REIW and CA001_W3_2 = 1, skip to procedure before CA002_W3_1; if this is a NEWIW and CA001_W3_2 = 1, skip to CA001_W3_5_1 and then skip to procedure before CA002_W3_1. CA001_W3_2 = 1 CA002_W3_1 , CA001_W3_2 = 1 CA001_W3_5 1 CA002_W3_1 .

CA001_W3_3 In which year did she pass away? ?_____1850...2014 (**CA001_W3_3_1**) Or how old was she then? ____0...120 (**CA001_W3_3_2**)

[IWER:Mark the year using four digits. 4 .]

CA001_W3_4 What was the main cause of your biological mothers death? [() , () , .]

PROCEDURE :

If CA000_W3_2 = 1 and CA001_W3_2 = 1, ask CA002_W3_1. CA000_W3_2 = 1 CA001_W3_2 = 1CA002_W3_1.

CA002_W3_1 Had your biological parents divorced? (,,.)

1. Yes
2. No → Skip to CA006_W3_1

PROCEDURE :

If CA000_W3_2 = 2 or CA002_W3_1 = 1, ask CA004_W3_1; otherwise, skip to procedure before CA005_W3_1. CA000_W3_2 = 2 CA002_W3_1 = 1 CA004_W3_1; CA005_W3_1 .

CA004_W3_1 Have you had a stepfather?

1. Yes
2. No → Skip to procedure before CA005_W3_1 CA005_W3_1

CA004_W3_2 Is your step father living?

[IWER: If the respondent has two and more stepfathers, we ask the last one. .]

1. Yes
2. No → Skip to procedure before CA005_W3_1 CA005_W3_1

PROCEDURE
If ZCA002_inf_3_ is missing, skip CA004_W3_0.

CA004_W3_0 Is your stepfather [Name]?

1. Yes → Skip to procedure before CA005_W3_1 CA005_W3_1
2. The name is wrong, and other information is right → Skip to CA004_W3_4_1 CA004_W3_4_1
3. The name and other information are wrong

[Preload fathers names. .]

CA004_W3_4 Whats the name of your stepfather?

1. Name
3. Name
5. Name
7. Name
9. Name
11. Name
97. Other

PROCEDURE
If CA004_W3_4 ≠ 97, skip to procedure before CA005_W3_1

CA004_W3_4_1 Whats the name of your stepfather? ________

PROCEDURE
If CA004_W3_0 = 2, skip to procedure before CA005_W3_1.

CA004_W3_3 What was your stepfathers birth year? ____1850...2014

[IWER:Mark the year using four digits. 4 .]

PROCEDURE :
If CA001_W3_2 = 2 or CA002_W3_1 = 1, ask CA005_W3_1. CA001_W3_2 = 2 CA002_W3_1 = 1, CA005_W3_1.

CA005_W3_1 Have you had a stepmother?

1. Yes
2. No → Skip to CA006_W3_1 CA006_W3_1

CA005_W3_2 Is your stepmother living?

[IWER: If the respondent has two and more stepmothers, we ask the last one. .]

1. Yes
2. No Skip to CA006_W3_1 CA006_W3_1

PROCEDURE
If ZCA002_inf_4_ is missing, skip CA005_W3_0.

CA005_W3_0 Is your stepmother [Name]?

1. Yes → Skip to CA006_W3_1 CA006_W3_1
2. The name is wrong, and other information is right → Skip to CA005_W3_4_1 CA005_W3_4_1
3. The name and other information are wrong

[Preload mothers names. .]

CA005_W3_4 Whats the name of your stepmother?

2. Name
4. Name
6. Name
8. Name
10. Name
12. Name
97. Other

PROCEDURE
If CA005_W3_4 ≠ 97, skip to CA006_W3_1.

CA005_W3_4_1 Whats the name of your stepmother? ________

PROCEDURE
If CA005_W3_0 = 2, skip to CA006_W3_1.

CA005_W3_3 What was your stepmothers birth year? ____1850...2014

[IWER:Mark the year using four digits. 4 .]

CA006_W3_1 Have you had an adopted father?

1. Yes
2. No → Skip to CA007_W3_1 CA007_W3_1

CA006_W3_2 Is your adopted father living?

1. Yes
2. No → Skip to CA007_W3_1 CA007_W3_1

PROCEDURE
If ZCA002_inf_5_ is missing, skip CA005_W3_0.

CA006_W3_0 Is your adopted father [Name]?

1. Yes → Skip to CA007_W3_1 CA007_W3_1

2. The name is wrong, and other information is right → Skip to CA006_W3_4_1 CA006_W3_4_1
3. The name and other information are wrong

[Preload fathers names. .]

CA006_W3_4 Whats the name of your adopted father?

1. Name
3. Name
5. Name
7. Name
9. Name
11. Name
97. Other

PROCEDURE
If CA006_W3_4 ≠ 97, skip to CA007_W3_1.

CA006_W3_4_1 Whats the name of your adopted father? __________

PROCEDURE
If CA006_W3_0 = 2, skip to CA007_W3_1.

CA006_W3_3 What was your adopted fathers birth year? ____1850...2014

[IWER:Mark the year using four digits. 4 .]

CA007_W3_ 1 Have you had an adopted mother?

1. Yes
2. No → Skip to procedure before CA007_W3_5 CA007_W3_5

CA007_W3_2 Is your adopted mother living?

1. Yes
2. No → Skip to procedure before CA007_W3_5 CA007_W3_5

PROCEDURE
If ZCA002_inf_6_ is missing, skip CA007_W3_0.

CA007_W3_0 Is your adopted mother [Name]?

1. Yes → Skip to procedure before CA007_W3_5 CA007_W3_5
2. The name is wrong, and other information is right → Skip to CA007_W3_4_1 CA007_W3_4_1
3. The name and other information are wrong

[Preload mothers names. .]

CA007_W3_4 Whats the name of your adopted mother?

2. Name
4. Name
6. Name
8. Name
10. Name
12. Name
97. Other

PROCEDURE
If CA007_W3_4 ≠ 97, skip to procedure before CA007_W3_5.

CA007_W3_4_1 Whats the name of your adopted mother? __________

PROCEDURE
If CA007_W3_0 = 2, skip to procedure before CA007_W3_5.

CA007_W3_3 What was your adopted mothers birth year? ____ 1850...2014

[IWER:Mark the year using four digits. 4 .]

PROCEDURE :
If CA006_W3_2 = 1 and CA007_W3_2 = 1, ask CA007_W3_5. CA006_W3_2 = 1 CA007_W3_2 = 1, CA007_W3_5.

CA007_W3_5 Whether your adopted parents are a couple now?

1. Yes
2. No

PROCEDURE :
If the respondent has alive biological father, ask the following questions from CA009 to CA026_W3. CA009 CA026_W3 .

[Show Card 5]

CA009 What is the highest level of education your biological father has completed?

1. No formal education (illiterate)
2. Did not finish primary school
3. Sishu/home school
4. Elementary school
5. Middle school
6. High school
7. Vocational school ( )

8. Two-/Three-Year College / Associate degree
9. Four-Year College / Bachelors degree
10. Post-graduate, Masters degree
11. Post-graduate, Doctoral degree/Ph.D

PROCEDURE :
If CA009_W3 = 1/2/3, then ask the next question. CA009_W3 = 1/2/3 .

CA009_W3 Is your biological father literate?
1. Yes
2. No

CA012 Dose your biological father work currently (work includes agricultural work, earning wage work, self-employed activities, and unpaid family business work, et. al.)? ( )
1. Yes
2. No

CA013 How is your biological father health? Very good, good, fair, poor or very poor?
1. Very good
2. Good
3. Fair
4. Poor
5. Very poor

CA014 Which is/was the highest occupation of your biological father?
1. Managers
2. Professionals and technicians
3. Clerks
4. Commercial and service workers
5. Agricultural, forestry, husbandry and fishery producers
6. Production and transportation workers 7. Cant be specified

CA015 What is your biological father average income at present? (not including the money from children, but including other all income like pension and government allowance). ( ) ___ yuan/year / (**CA015_1**) or _____ yuan/month / (**CA015_2**).

CA016_W3 Where was your biological father born?

1. The same birthplace as mine
2. My permanent addresss village/neighborhood
3. Another village/neighborhood in my permanent addresss county/city/district _____(**CA016_W3_1**) /// _____(**CA016_W3_1**)
4. Other ______(**CA016_W3_2**) province_city_county/city/district _ _ // _(**CA016_W3_3**) township/street //, ______(**CA016_W3_4**) village/ neighborhood /
5. Abroad

[Show Card 7]

CA016 Where does your your biological father normally live? 1. The same household as mine

2. The same or an adjacent dwelling/courtyard with me () ()
3. His birthplace
4. Another household in my permanent addresss village/neighborhood /
5. Another village/neighborhood in my permanent addresss county/city/district ______(**CA016_1**) village/neighborhood, how far away ____(**CA016_2**) km /// ______(**CA016_1**) /: ____(**CA016_2**)
6. Other ______(**CA016_3**) province_city_county/city/district _ _ // ______(**CA016_4**) township/street //, ______(**CA016_5**) village/neighborhood /
7. Abroad

[IWER: county/city includes county-level administrative units/county-level city/district. : ///]

PROCEDURE :
If CA016 = 5/6/7, ask CA017. CA016 = 5/6/7, CA017.

[Show Card 2]

CA017 What kind of location does your biological father live in?

1. Main city zone
2. Combination zone between urban and rural areas
3. The town center
4. ZhenXiang area
5. Special area

6. Township central
7. Village

CA018 Is his hukou in the same place as his current residence?

1. Yes → Skip to CA020 CA020
2. No
3. Does not have Hukou → Skip to CA021 CA021

[CAPI: Preload the Rs permanent address.]

CA019 What is the location of your biological father current hukou?

1. His birthplace
2. My birthplace
3. My permanent addresss village/neighborhood /
4. Another village/neighborhood in my permanent addresss county/city/district ______(**CA019_ 1**) village/neighborhood /// ______ (**CA019_ 1**) /
5. Other ______(**CA019_2**) province_city_county/city/district _ _ // ______(**CA019_3**) township/street //, ______(**CA019_4**) village/neighborhood /
6. Abroad

[IWER: county/city includes county-level administrative units/county-level city/district. : ///]

CA020 What is your biological father current hukou status? 1. Agriculture Hukou

2. Non-Agriculture Hukou
3. Unified Residency Hukou
4. Does not have Hukou

[F1 .]

CA021 Does your biological father own a house?

1. Yes
2. No → Skip CA022 CA022

CA022 Do you know the present value of your biological father house? ____ 10000 Yuan

CA023_W3 Is your biological father a party member?

1. Yes
2. No

CA024_W3 Does your biological father belongs to Ethnic Minorities?
1. Yes
2. No

PROCEDURE :
If CA024_W3 = 1, ask CA024_W3_1. CA024_W3 = 1, CA024_W3_1.

CA024_W3_1 Which Ethnic Minority does your biological father belong to?
1. Hui
2. Zhuang
3. Uighur
4. Man
5. Yi
6. Tibetan
7. Miao
8. Mongol
9. Dai
10. Tujia
11. Lahu
12. Other ______(**CA024_W3_1_1**)

CA025_W3 Does your biological father have any religious belief?
1. Yes
2. No

PROCEDURE :
Ask CA026_W3 if the respondents biological father is older than 60. 60 CA026_W3.

CA026_W3 Can your biological father take care of himself? 1. Yes
2. No

PROCEDURE :
If Family R has alive biological mother/ stepfather/ stepmother/ adopted father/ adopted mother, add one more section with questions identical to those asked about Family Rs alive biological father. / / / / .

PROCEDURE :

If Family Rs spouse has alive biological father/ mother/ stepfather/ stepmother/ adopted father/ adopted mother, add sections with questions identical to those asked the Family R. / / / / / .

CB CHILDREARING INFORMATION

PROCEDURE :

if this is a NEWIW, then ask CB050_W3 and fill in the next table. CB050_W3 .

CB050_W3 How many living children do you have? Including biological and step and adapted children. _____ . ____

[IWER: , 1. 2. , .]

PROCEDURE :

- if this is a REIW, preload all the childrens information, if missing, then fill in the blanks; if not missing, please confirm the answers, if the answers not true, then modify them. .

[, 1. 2. ,

, .]

[.]

[IWER: If the names are not very correctly, then ask if used names; the same sound names or likely sound names are the correct names. .]

CB051_W3_1 Except recorded children, do you have other living children, including your step children and adapted children? ____0…25

[IWER: , , .]

PROCEDURE :

- repeat questions for each living child. .

PROCEDURE :

- Preload the information of children, if there is none, then ask; otherwise, skip. , .

ChildName Child Name is ____ ? ____
ChildBYear (ChildName)s birth year is_____ ?1850...2015 ____ **ChildGender** (ChildName)s gender is ?

1. Male
2. Female

ChildSta What is (ChildName) doing now ?

1. studying in the school
2. working
3. studying while working
4. neither studying nor working

ChildYN Is (ChildName)s record correct ?

1. Yes
2. No

PROCEDURE :
- repeat questions for each passed away. .

CB052_W3_Death When did (ChildName) pass away?The cause is_____?

____(**CB052_W3_Death_1**) .
____(**CB052_W3_Death_2**)

CB052_W3 What is the highest level of education [childs name] have completed? (not including adult education) ( )

1. No formal education (illiterate)
2. Did not finish primary school
3. Sishu/home school
4. Elementary school
5. Middle school
6. High school
7. Vocational school ( )
8. Two-/Three-Year College/Associate degree
9. Four-Year College/Bachelors degree
10. Masters degree
11. Doctoral degree/Ph.D.

PROCEDURE :
If CB052_W3 = 1/2/3, ask the next question. CB052_W3 = 1/2/3, .

CB052_W3_1 Is [childs name] literate?

1. Yes
2. No

PROCEDURE :
• Preload the Rs permanent address

CB053 Where does this [childs name] normally live now? 1. This household, and economicly dependent.

2. This household, but economicly independent.
3. The same or adjacent dwelling/courtyard with you () ()
4. Another household in your permanent addresss village/neighborhood /
5. Another village/neighborhood in your permanent addresss county/city/district; distance from here: ____km /// ______ / (**CB053_1**): ____(**CB053_2**)
6. Other province_city_county/city/district _ _ //______(**CB053_3**) // ______(**CB053_4**) village/neighborhood /
7. Abroad

PROCEDURE :
If CB053 = 5/6/7, ask CB054; otherwise, go to CB055. CB053 = 5/6/7CB054, CB055

CB054 In what type of location does [childs name] live? 1. Main city zone

2. Combination zone between urban and rural areas
3. The town center
4. ZhenXiang area
5. Special area
6. Township central
7. Village

CB055 What is the current hukou status of [childs name]?

1. Agriculture Hukou
2. Non-Agriculture Hukou
3. Unified Residency Hukou
4. Do not have Hukou

[F1 .]

PROCEDURE :
If CB055= 3, ask CB055_W2_1 and CB055_W2_2; otherwise, skip to CB056_W3<br>CB055= 3CB055_W2_1 CB055_W2_2CB056_W3

CB055_W2_1 What is [childs name]s Hukou status before he/she has the unified residency hukou??

1. Agriculture Hukou
2. Non-Agriculture Hukou
3. Do not have Hukou

CB055_W2_2 When did [childs name] have the unified residence Hukou? _____ 2000…2015 2000…2015

CB081 Where was [childs name] born?

1. The same birthplace as me
2. My permanent addresss village/neighborhood
3. Another village/neighborhood_____ in my permanent addresss county/city/district /// _____(**CB081_1**)
4. Other province_city_county/city/district _ _ //______(**CB081_2**) // ______(**CB081_3**) /
5. Abroad

[Preload the Rs permanent address.]

CB057 What is the current hukou location of [childs name]?

1. This household
2. His/her birthplace /
3. Your permanent address s village/neighborhood /
4. Another village/neighborhood ______in your permanent addresss county/city/district /// ______(**CB057_1**)
5. Other province_city_county/city/district _ _ //______(**CB057_2**) //, ______(**CB057_3**) /
6. Other(specify) ( ) ______(**CB057_4**)

[IWER: county/city includes county-level administrative units/county-level city/district. : ///]

[CB063 BRANCHPOINT]

- If [childs name] is less than 16, go to CB063_W3_1 16 CB063_W3_1

CB063 What is [childs name] status?

1. Married with spouse present
2. Married but not living with spouse temporarily for reasons such as work
3. Separated ( )
4. Divorced

5. Widowed
6. Never married
7. Cohabitated

CB063_W3_1 How is [childs name] health? Very good, good, fair, poor or very poor?
1. Very good
2. Good
3. Fair
4. Poor
5. Very poor

CB063_W3_2 Is [childs name] the party member? 1. Yes
2. No

CB063_W3_3 Is [childs name] the Ethnic Minorities?
1. Yes
2. No → Skip to CB063_W3_5 CB063_W3_5

CB063_W3_4 Which Ethnic Minorities of [name of child]? [Pro- cedure: ask each child who was chose from CB063_W3_3. CB063_W3_3 .]
1. Hui
2. Zhuang
3. Weiwuer
4. Man
5. Yi
6. Zang
7. Miao
8. Mongol
9. Dai
10. Tujia
11. Lahu
12. other ______ **(CB063_W3_4_1)**

CB063_W3_5 Does [childs name] have any religious belief?
1. Yes
2. No

PROCEDURE :
- If [childs name] is older than 60, ask CB063_W3_6 60 CB063_W3_6

CB063_W3_6 Can [childs name] take care of him/herself?

1. Yes
2. No

PROCEDURE :
• If the children has never married (CB063= 6), please skip to CB064_W3_2 (CB063= 6)CB064_W3_2

CB065 How many children does [childs name] have? _____ 0...25

PROCEDURE :
• If [childs name] does not have children (CB065 = 0）, please skip to CB064_W3_2 0CB064_W3_2

CB066 How many children under 16 does [childs name] have? 16
____ 0...25

CB067 How many grandchildren does [childs name] have? [] __ 0...25

PROCEDURE :
• If [childs name] havs grandchildren (CB065 > 0), please ask CB068 CB068

CB068 How many grandchildren under age 16 does [childs name] have? [] 16 __ 0...25

[Softcheck: if the number of grandchildren under age 16 daughters is smaller than the number of grandchildrenadult daughters. CB067 <CB068,]

PROCEDURE :
• 65 CB064_W3_2-CB064_W3_4 CB069

CB064_W3_2 Does [childs name] participate in the New Rural Social Pension Insurance?

1. Yes
2. No → Skip to CB069 CB069

CB064_W3_3 In what month and year was [childs name] first cover by New Rural Social Pension Insurance?

( ) ____ 2008...2013 (**CB063_W3_3_1**) year ____ 0...12 (**CB063_W3_3_2**) month

CB064_W3_4 Does [childs name]s participatation related to parents? （）

1. Yes
2. No
3. Other _________(CB064_W3_4_1) （）

CB069 How much did the total income of [childs name] (and his/her spouse) in the past year? （/）

1. 0
2. less than 2000 yuan 2
3. 2000-5000 yuan 2 5
4. 5000-10000 yuan 5 1
5. 10000-20000 yuan 1 2
6. 20000-30000 yuan 2 3
7. 30000-50000 yuan 3 5
8. 50000-100000 yuan 5 10
9. 100000-150000 yuan 10 15
10. 150000-200000 yuan 15 20
11. 200000-300000 yuan 20 30
12. more than 300000 yuan 30

CB071_W3 Does [childs name] own a house?

1. Yes
2. No → Skip to next child

CB072_W3 Do you know the present value of your childs house? _____ 10000 Yuan

PROCEDURE :

- repeat questions for additional children. .

PROCEDURE :

- go/proceed to the next person. .

CC SIBLINGS

[INTRO: Next I have some questions about your brothers and sisters. .]

PROCEDURE :

If this is a REIW andXSiblingNum > 0, ask the following questions displayed as a table. XSiblingNum > 0.

CC000_W3 Is the following information of your living siblings in 2014 correct? If not, modify them in the blanks. 2014 .

[, 1. 2. , 1. 2. 3. 4. , 1. 2. .] [IWER: If the names are not very correctly, then ask if used names; the same sound names or likely sound names are the correct names. .]

[,]

CC001_W3 Is there any of your siblings died since 2014? Choose all those apply. .

1. Siblings name
2. Siblings name

⋮

15. Siblings name

97. None → Skip CC002_W3 CC002_W3

CC002_W3 Whats the cause of [siblings name]s death?

____

CC000_W3_1 Except you, how many living siblings do you have by 2014, including your step siblings and adopted siblings? 2014 ____0...15 person

PROCEDURE :

If CC000_W3_1 > 0, ask CC003_W3_2. CC000_W3_1 > 0, CC003_W3_2.

CC003_W3_2 Ask the information of living siblings from oldest to youngest (except recorded siblings). ( ) .

[, 1. 2. , 1. 2. 3. 4. .]

PROCEDURE :

If XSiblingName is not missing, ask each one the next questions from CC003_W3 to CC019_W3. CC003_W3 CC019_W3 .

CC003_W3 Whats the highest level of education [siblings name] got? (not including adult education) ( )

1. No formal education (illiterate)
2. Did not finish primary school
3. Sishu/home school
4. Elementary school
5. Middle school
6. High school
7. Vocational school ( )
8. Two-/Three-Year College/Associate degree
9. Four-Year College/Bachelors degree
10. Masters degree
11. Doctoral degree/Ph.D.

**PROCEDURE** :<br>If CC003_W3 = 1/2/3, ask the next question. CC003_W3 = 1/2/3, .

CC003_W3_0 Is [siblings name] literate?

1. Yes
2. No

**PROCEDURE** :<br>If CC000_W3_1 > 0, ask the next questions from CC004_W3 to CC018_W3 for each of the newly added siblings. CC000_W3_1 > 0, CC004_W3 CC018_W3 .

CC004_W3 Is [siblings name] the party member? 1. Yes

2. No

CC005_W3 Does [siblings name] belong to the Ethnic Minorities?

1. Yes
2. No → Skip to CC016_W3 CC016_W3

[Show Card 8]

CC006_W3 Which Ethnic Minority does [siblings name] belong to?

1. Hui
2. Zhuang
3. Uighur
4. Man
5. Yi

6. Tibetan
7. Miao
8. Mongol
9. Dai
10. Tujia
11. Lahu
12. other ______(**CC006_W3_1**)

CC016_W3 Did [siblings name] have the experience of Down to the Countryside Movement?
1. Yes
2. No

CC017_W3 Did [siblings name] have the experience of army?
1. Yes
2. No

CC018_W3 Does [siblings name] have any religious belief? 1. Yes
2. No

**PROCEDURE** :<br>Ask CC006_W3_2 for those siblings who are older than 60. 60 CC006_W3_2.

CC006_W3_2 Can [siblings name] take care of him/herself?

1. Yes
2. No

CC007_W3 How many children does [siblings name] have?
___ 0...25 person

**PROCEDURE** :<br>If CC007_W3 = 0, skip to CC011_W3. CC007_W3 = 0CC011_W3.

CC008_W3 How many children under age 16 does [siblings name] have? 16 ___ 0...25 person

CC009_W3 How many grandchildren does [siblings name] have? ___ 0...25 person

**PROCEDURE** :<br>If CC009_W3 = 0, skip to CC011_W3. CC009_W3 = 0CC011_W3.

CC010_W3 How many grandchildren under age 16 does [siblings name] have?
16 ___ 0…25 person

CC011_W3 What is [siblings name] marital status? 1. Married with spouse at present
2. Married but not living with spouse temporarily for reasons such as work

3. Separated ( )
4. Divorced
5. Widowed
6. Never married
7. Cohabitated

CC012_W3 How is [siblings name] health? Very good, good, fair, poor or very poor?
1. Very good
2. Good
3. Fair
4. Poor
5. Very poor

CC013_W3 Does [siblings name] work now?
1. Yes
2. No

[Show Card 9]

CC015_W3 Which was the highest occupation of [siblings name]?
1. Managers
2. Professionals and technicians
3. Clerks
4. Commercial and service workers
5. Agricultural, forestry, husbandry and fishery producers
6. Production and transportation workers 7. Cant be specified

CC019_W3 How about the life of [siblings name] compared with yours?
1. Much better than mine
2. A little better than mine
3. Same with mine

4. A little worse than mine
5. Much worse than mine

PROCEDURE :

If proxy = 0, answer CG003_W2. proxy = 0 CG003_W2.

CG003_W2 How often did the respondent receive assistance in answering section CA/CC? CA/CC

1. Never
2. A few times
3. Most or all of the time

PROCEDURE :

If Spouses name (XSName) is not missing, skip CA024. CA024.

CA024 Do you keep in contact with your parents-in-law? / 1. Yes, keep in contact
2. No, have no contact or no parents-in-law /

PROCEDURE :

If Spouses name (XSName) is not missing and Spouse is not dead (XSDied!=1), or CA024 = 1, plus proxy = 0 and XSDied! = 1, ask CC005_W2_1. CA024 = 1 proxy = 0 XSDied! = 1CC005_W2_1.

CC005_W2_1 If the Family Rs Spouse is in, please ask the Spouse about Spouses parents and siblings questions; if the Spouse is not in, please ask Family R for proxy. For the following questions about Spouses parents and siblings (Section CA/ Section CC), who answered the questions? Please record. / /, ./ .

1. Family R
2. Spouse

PROCEDURE :

If Family Rs Spouse has sibling(s), add one more section with questions identical to those asked about Family Rs siblings (CC000_W3_S to CC019_W3_S). And if CC005_W2_1 = 2 and proxy = 0, answer CG003_W2. (CC000_W3_S CC019_W3_S). CC005_W2_1 = 2 and proxy = 0 CG003_W2.

PROCEDURE :

Variables standing for the information of spouses sibling end up with _S. _S.

C2 TIME TRANSFER AND TRANSFERS

[Introduction: In the following three parts: CD time transfer, CE transfer and CF time spent providing care. we will ask you how you contact with parents and children, and economic trancfers. .]

CD TIME TRANSFER

CONTACT WITH PARENTS

PROCEDURE :

Skip to CD003 if father/mother/father-in-law/mother-in-law is not alive OR father/mother/father-in-law/mother-in-law is household member. // ()/ () // ()/ () CD003.

LOOP:

Repeat each living and non-resident parents couple about biological parents/step parents/adapted parents/parents-in-law, if each couple both have father and mother, then ask CD001
if the couple just have one person, then ask CD001_W3_1/CD001_W3_2
// ( ) CD001, CD001_W3_1/CD001_W3_2

CD001 [If the father/mother/father-in-law/mother-in-law is alive] Whom does your father/mother/father-in-law/mother-in-law live with (choose all that apply)? / ( )

/// ()// () 1. Live alone with him/herself
2. his/her spouse /
3. my spouse and I
4. my siblings
5. siblings of my spouse
6. my children
7. my siblings children
8. my spouses siblings children
9. other relatives
10. Take turns in childrens homes
11. Nursing home
12. Other

PROCEDURE :

If CD001 $\neq$ 2, then ask CD002. CD001 $\neq$ 2, CD002.

CD002 How often do you/your spouse see your father/mother/ father-in-law/mother-in-law? // ()/ ()

1. Almost every day
2. 2-3 times a week 2-3
3. Once a week
4. Every two weeks
5. Once a month
6. Once every three months
7. Once every six months
8. Once a year
9. Almost never
10. Other

PROCEDURE :
- If answer to (**CD001**) is my siblings, please ask CD001_W3_ 1 CD001_W3_ 1

CD001_W3_ 1 Which siblings? ( )

PROCEDURE :
- If answer to (**CD001**) is siblings of my spouse, please ask CD001_W3_2 CD001_W3_2

CD001_W3_2 Which siblings of my spouse? ( )

PROCEDURE :
- If answer to (**CD001**) is my children, please ask CD001_W3_3 CD001_W3_3

CD001_W3_3 Which children? ( )

PROCEDURE :
- If answer to (**CD001**) is my siblings children, please ask CD001_W3_4 CD001_W3_4

CD001_W3_4 Which siblings children? ( )

PROCEDURE :
- If answer to (**CD001**) is my spouses siblings children, please ask CD001_W3_5 CD001_W3_5

CD001_W3_5 Which spouses siblings children? ( )

[IWER: If the father and mother or father-in-law and mother-in-law live together, do not repeat the other one. / .]

CONTACT WITH CHILDREN

PROCEDURE :

If respondent has no non-cohabiting children, skip to CF001. CF001

PROCEDURE :

For each of the non-coresident child, ask the following two questions CD003-CD004. CD003-CD004.

CD003 How often do you see (childs name)? []?

1. Almost every day
2. 2-3 times a week 2-3
3. Once a week
4. Every two weeks
5. Once a month
6. Once every three months
7. Once every six months
8. Once a year
9. Almost never
10. Other

PROCEDURE :

IF (CD003= 1 – 3), skip (CD004). (CD003= 1 – 3), (CD004).

CD004 How often do you have contact with (childs name) either by phone, text message, mail, or email, when you didnt live with (childs name)? [] [] ?

1. Almost every day
2. 2-3 times a week 2-3
3. Once a week
4. Every two weeks
5. Once a month
6. Once every three months
7. Once every six months
8. Once a year
9. Almost never
10. Other

CE TRANSFERS

[Introduction: Families sometimes help one another in a variety of ways, and each type of help can be important. The next questions are about help you (and your spouse) have given to or received from your non-coresident family members in the past year. . .]

Receipt of Economic Assistance including Cash and In-kind Transfers

[CAPI: Preload parents names.

1. [Biological fathers name] [Biological mothers name] if both biological parents are living and not divorced; [Biological fathers name] [Stepmothers name] if biological parents are divorced and names of biological father and stepmother are nonmissing; [Biological fathers name] if biological parents are divorced, biological fathers name is nonmissing and stepmothers name is missing; [Stepmothers name] if biological parents are divorced, biological fathers name is missing and stepmothers name is nonmissing; [] if biological parents are divorced and names of biological father and stepmother are missing

2. [Biological mothers name] [Stepfathers name] if biological parents are divorced and names of biological mother and stepfather are nonmissing; [Biological mothers name] if biological parents are divorced, biological mothers name is nonmissing and stepfathers name is missing; [Stepfathers name] if biological parents are divorced, biological mothers name is missing and stepfathers name is nonmissing; [] if biological parents are divorced and names of biological mother and stepfather are missing

3. [Adopted fathers name] [Adopted mothers name] if adopted parents are the spouse; [Adopted fathers name] if adopted parents are not the spouse and adopted fathers name is nonmissing

4. [Adopted mothers name] if adopted parents are not the spouse and adopted mothers name is nonmissing

5, 6, 7 and 8 display the name(s) of the spouses biological father / biological mother / stepfather / stepmother / adopted father / adopted mother in the similar manner.]

**PROCEDURE** :
Repeat questions from CE002 to CE023 according to the list of the nonmissing parents names. CE002 CE023.

[IWER: money/in-kind support means support living expenses / foodstuff / vegetables / clothes / water and electricity / telephone rate and other daily consumption; Marriage and funeral / move to new house / in hospital/ go to university / new born and other economic transfer. / .]

CE002 In the past year, how much economic supports did you or your spouse receive from

your [fathers name][mothers name]?

1. Total money support _____(**CE002_1**) yuan, among which how much is regular ____(**CE002_2**) yuan (for example, support living expenses/water and electricity/telephone rate/return loan or other cost in regular). _____(**CE002_1**) _____ (**CE002_2**) (/) .
2. Total in-kind support _____(**CE002_3**) yuan, among which how much is regular _____(**CE002_4**) yuan (for example, support food/vegetables/clothes or other in-kind support in regular). _____(**CE002_3**) ____**CE002_4**) () .

[IWER: Regular means supporting at fixed time such as per month/quarter of a year/half of a year/year, etc. .] [IWER: If give no money or in-kind support, please fill in 0; if respondent answer RF or DK, then ask CE003, or skip to CE022. 0, CE003CE022.]

CE003 Add unfolding brackets (100/200/400/800/1600 yuan) for CE002_1, CE002_2, CE002_3 or CE002_4 answered RF or DK. CE002_1, CE002_2, CE002_3 CE002_4 (100/200/400/800/1600) .

CE022 In the past year, how much economic supports did you or your spouse provide to your [fathers name][mothers name]?

1. Total money support _____(**CE022_1**) yuan, among which how much is regular _____(**CE022_2**) yuan (for example, support living expenses/water and electricity/telephone rate/return loan or other cost in regular) . _____(**CE022_1**) _____ (**CE022_2**) (/) .
2. Total in-kind support _____(**CE022_3**) yuan, among which how much is regular _____(**CE022_4**) yuan (for example, support food/vegetables/clothes or other in-kind support in regular). _____(**CE022_3**) _____(**CE022_4**) () .

[IWER: Regular means supporting at fixed time such as per month/quarter of a year/half of a year/year, etc. .] [IWER: If give no money or in-kind support, please fill in 0; if respondent answer RF or DK, then ask CE023, otherwise skip to CE009. 0, CE023CE009.]

CE023 Add unfolding brackets (100/200/400/800/1600 yuan) for CE022_1, CE022_2, CE022_3 or CE022_4 answered RF or DK. CE022_1, CE022_2, CE022_3 CE022_4 (100/200/400/800/1600) .

[CAPI: Preload all living childrens names. .]

CE009 In the past year, how much economic supports did you or your spouse receive from your [childs name]?

.

1. Total money support _____(**CE009_1**) yuan, among which how much is regular _____(**CE009_2**) yuan (for example, support living expenses/water and electricity/telephone rate/return loan or other cost in regular). _____(**CE009_1**) _____ (**CE009_2**) (/) .
2. Total in-kind support _____(**CE009_3**) yuan, among which how much is regular _____(**CE009_4**) yuan (for example, support food/vegetables/clothes or other in-kind support in regular). _____(**CE009_3**) _____(**CE009_4**) () .

[IWER: Regular means supporting at fixed time such as per month/quarter of a year/half of a year/year, etc. .] [IWER: If give no money or in-kind support, please fill in 0; if respondent answer RF or DK, then ask CE010, or skip to CE029. 0, CE010CE029.]

CE010 Add unfolding brackets (100/200/400/800/1600 yuan) for CE009_1, CE009_2, CE009_3 or CE009_4 answered RF or DK. CE009_1, CE009_2, CE009_3 CE009_4 (100/200/400/800/1600) .

CE029 In the past year, how much economic supports did you or your spouse provide to your [child name]? []

1. Total money support _____(**CE029_1**) yuan, among which how much is regular _____(**CE029_2**) yuan (for example, support living expenses/water and electricity/telephone rate/return loan or other cost in regular). _____(**CE029_1**) _____ (**CE029_2**) (/) .
2. Total in-kind support _____(**CE029_3**) yuan, among which how much is regular _____(**CE029_4**) yuan (for example, support food/vegetables/clothes or other in-kind support in regular). _____(**CE029_3**) _____(**CE029_4**) () .

[IWER: Regular means supporting at fixed time such as per month/quarter of a year/half of a year/year, etc. .] [IWER: If give no money or in-kind support, please fill in 0; if respondent answer RF or DK, then ask CE030, or skip to CE072. 0, CE030_1CE072_W3.]

CE030 Add unfolding brackets (100/200/400/800/1600 yuan) for CE029_1, CE029_2, CE029_3 or CE029_4 answered RF or DK. CE029_1, CE029_2, CE029_3 CE029_4 （100/200/400/800/1600 ）.

CE072_W3 Including both giving to you and receiving from you, did any of your non-coresident siblings have economic supports with you or your spouse

1. Siblings name
2. Siblings name

⋮

30. Siblings name
99. None → Skip to CE016_W3

**PROCEDURE** :
Repeat questions from CE072_W2 to CE075_W2 according to the selected names in CE072_W3 if the sibling is living and name is nonmissing. CE072_W3 CE072_W2 CE075_W2.

CE072_W2 In the past year, how much economic supports did you or your spouse receive from your [siblings name]?

1. Total money support ______(**CE072_W2_1**) yuan, among which how much is regular _____(**CE072_W2_2**) yuan (for example, support living expenses/water and electricity/telephone rate/return loan or other cost in regular). _____ **(CE072_W2_1)** （ ） _____**(CE072_W2_2)** （/）
2. Total in-kind support _____(**CE072_W2_3**) yuan, among which how much is regular _____(**CE072_W2_4**) yuan (for example, support food/vegetables/clothes or other in-kind support in regular). _____(**CE072_W2_3**) _____(**CE072_W2_4**) （）.

[IWER: Regular means supporting at fixed time such as per month/quarter of a year/half of a year/year, etc. .] [IWER: If give no money or in-kind support, please fill in 0; if respondent answer RF or DK, then ask CE073_W2, or skip to CE074_W2. 0, CE073_W2CE074_W2.

CE073_W2 Add unfolding brackets (100/200/400/800/1600 yuan) for CE072_W2. CE072_W2（100/200/400/800/1600 ）.

CE074_W2 In the past year, how much economic supports did you or your spouse provide to your [siblings name]?

1. Total money support ______(**CE074_W2_1**) yuan, among which how much is regular _____(**CE074_W2_2**) yuan (for example, support living expenses/water and electricity/telephone rate/return loan or other cost in regular). _____ **(CE074_W2_1)** （ ） _____**(CE074_W2_2)** （/）
2. Total in-kind support _____(**CE074_W2_3**) yuan, among which how much is regular _____(**CE074_W2_4**) yuan (for example, support food/vegetables/clothes or other in-kind support in regular). _____(**CE074_W2_3**) _____**(CE074_W2_4)** （）.

[IWER: Regular means supporting at fixed time such as per month/quarter of a year/half of a year/year, etc. .] [IWER: If give no money or in-kind support, please fill in 0; if respondent answer RF or DK, then ask CE075_W2, or skip to CE016_W3. 0, CE075_W2CE016_W3.]

CE075_W2 Add unfolding brackets (100/200/400/800/1600 yuan) for CE074_W2. CE074_W2（100/200/400/800/1600 ）.

CE016_W3 In the past year, how much cash gift did you or your spouse receive from your non-coresident other relatives or friends? Total money and in-kind support ______ yuan (for example, marriage and funeral/move to new house/new born/go to university, and economic aid for fall ill or difficult to live, but not including borrowing money). ______ （）.

CE017_W3 Add unfolding brackets (100/200/400/800/1600 yuan) for CE016_W3. CE016_W3（100/200/400/800/1600 ）.

CE036_W3 In the past year, how much cash gift did you or your spouse provide to your non-coresident other relatives or friends? Total money and in-kind support ______ yuan (for example, marriage and funeral/move to new house/new born/go to university, and economic aid for fall ill or difficult to live, but not including borrowing money). ______ （）.

[IWER:If give no money nor in-kind support, please fill in 0; if respondent answered RF or DK, please ask CE037_W3, or skip to CE066_W2BR. 0, CE037_W3 CE066_W2BR.]

CE037_W3 Add unfolding brackets (100/200/400/800/1600 yuan) for CE036_W3. CE036_W3（100/200/400/800/1600 ）.

CE016 In the past year, how much economic supports did you or your spouse receive from your non-coresident other relatives or friends, excluding cash gift? Total money and in-kind support _______ yuan. (for example, marriage and funeral/move to new house/new born/go to university, and economic aid for fall ill or difficult to live, but not including borrowing money). ______ （ . ）

[IWER: If give no money nor in-kind support, please fill in 0; if respondent answered RF or DK, please ask CE017, or skip to CE036. 0, CE017CE036.]

CE017 Add unfolding brackets (100/200/400/800/1600 yuan) forCE016. CE016 （100/200/400/800/1600 ）.

CE036 In the past year, how much economic supports did you or your spouse provide to your non-coresident other relatives or friends,excluding cash gift? Total money and in-kind support ______ yuan. (for example, marriage and funeral/move to new house/new born/go to university, and economic aid for fall ill or difficult to live, but not including borrowing money). ______ （ ）.

[IWER: :If give no money nor in-kind support, please fill in 0; if respondent answered RF or DK, please ask CE037, or skip to CE066_W2BR. 0, CE037 CE066_W2BR.]

CE037 Add unfolding brackets (100/200/400/800/1600 yuan) forCE036. CE036 （100/200/400/800/1600 ）.

CE066_W2 BRANCHPOINT:
IF [CHILDS NAME] IS NOT UNMARRIED (CB063 = 1/2/3/4/5/7), ASK CE066_W2

[Preload each child that is not unmarried. .]

[Preload the information of them, if there is none, then ask; otherwise, then skip. .]

[IWER: please ask about each child CE066_W2 - CE070_W2_1. CE066_W2 - CE070_W2_1.]

[IWER: If the child got married more than one times, please ask about the first one. 1 1 .]

CE066_W2 When did your [childns name] get married? []

____1900…2015 (**CE066_W2_1**) year __0…12 (**CE066_W2_2**) month __0…31 (**CE066_W2_3**) day

CE067_W2_1 Did you give betrothal gifts when [child name] got married? []

1. Yes
2. No → Skip to CE069_W2_1 CE069_W2_1

CE068_W2_1 At that time, how much was the total value of the betrothal gifts? _____ yuan.

.

CE069_W2_1 Did you buy a house for him/her when [child name] got married? []

1. Yes
2. No → Skip to procedure before CF001 CF001

CE070_W2_1 At that time, how much was the total value of the house? _____10000 yuan.

.

CF TIME SPENT PROVIDING CARE

PROCEDURE :

If the respondent has any grandchildren, ask CF001 - CF003; otherwise, skip to CF004. /CF001 - CF003 ; CF004 .

CF001 Did you spend any time taking care of your grandchildren last year?

1. Yes
2. No → Skip to the procedure before CF004 CF004

CF002 For which childs children did you provide care? 1. Childs name

2. Childs name

⋮

25. Childs name

99. → Skip to CE074_W3

[IWER: Please list all the children including coresident ones, and add a choice deceased children in CAPI list. Select from list displayed by CAPI (childs name)]

PROCEDURE :

Repeat question CF003 according to the list of names in CF002. CF002 CF003.

CF003 Approximately how many weeks and how many hours per week did you spend last year taking care of this child s children? Myself ____(**CF003_ 1**)weeks , ____(**CF003_2**) hours per week /, My spouse ____(**CF003_3**) weeks , ____(**CF003_4**) hours per week / [IWER: Mark 1 if the period is less than 7 days. 7 1] [Hardcheck: if more than 52 weeks are reported or more than 140 hours are reported. 52 140 .]

**PROCEDURE** :<br>If both parents were not living at last IW or both of them are household members, or one parent were not living at last IW and the other is a household member, go to CF007_W2.

CF004 Did you or your spouse take care of your parents or parents-in-law during the last year in assisting them in their daily activities or other activities (e.g., household chores, meal preparation, laundry, going out, grocery shopping, financial management, etc.)? ( ) ( )

1. Yes
2. No → Skip to next module

CF005 Approximately how many weeks and how many hours per week did you yourself spend last year taking care of your parents or parents-in-law? ( )

1. Your father __(**CF005_ 1**) weeks ; ____(**CF005_2**) hours per week / 2. Your mother __(**CF005_3**) weeks ; _____(**CF005_4**) hours per week / 3. Your father-in-law ( ) __(**CF005_5**) weeks ; ___(**CF005_6**) hours per week /
4. Your mother-in-law ( ) __(**CF005_7**) weeks ; ___(**CF005_8**) hours per week /

[Softcheck: if (1) is checked in CF004 and 0 or missings are reported in CF005. CF004 = 1, CF005 = 0]

[Softcheck: if more than 52 weeks are reported or more than 140 hours are reported. 52 140]

CF006 Approximately how many weeks and how many hours per week did your spouse spend last year taking care of your parents or parents-in-law? ( )

1. Your father __(**CF006_ 1**) weeks ; ____(**CF006_2**) hours per week / 2. Your mother __(**CF006_3**)weeks ; _____(**CF006_4**) hours per week / 3. Your father-in-law ( ) __(**CF006_5**) weeks ; ___(**CF006_6**) hours per week /

4. Your mother-in-law （） __(**CF006_7**) weeks ; ___(**CF006_8**) hours per week /

[Softcheck: if (1) is checked in CF004 and 0 or missings are reported in CF006. CF004 = 1, CF006 = 0]

[Softcheck: if more than 52 weeks are reported or more than 140 hours are reported. 52 140]

CF007_W2 How often did the respondent receive assistance in answering sections CB, CD, CE and CF? CB, CD, CE CF

[IWER: If it is answered by a proxy, please record the respondents reaction. If the Family R isnt in or cant answer questions, and proxyed by his/her spouse, please choose the (4). . （4）. **]**

1. Never
2. A few times
3. Most or all of the time
4. Proxy by the spouse.

A HOUSEHOLD MEMBER

[INTRO: Relatives can have important effects on your life. Wed like to ask you some questions about other members of your household. .] （ ）， .]

A001_W3 Whom do you live together? (preload all the names of spouse/parents/stepparents/children/sib

choose all that apply. .

11 ?

1. Spouse
2. My parents or parents in law
3. Children
4. My Siblings
5. My spouses Siblings
6. None of the above

PROCEDURE :

- If answer to (**A001_W3**) is My parents or parents in law, please ask A001_W3_0 A001_W3_0

A001_W3_0 Which parents? （）

PROCEDURE :
• If answer to (**A001_W3**) is Children, please ask A001_W3_1 A001_W3_1

A001_W3_1 Which children? （）

PROCEDURE :
• If answer to (**A001_W3**) is My Siblings, please ask A001_W3_2 A001_W3_2

A001_W3_2 Which siblings? （）

PROCEDURE :
• If answer to (**A001_W3**) is My spouses Siblings, please ask A001_W3_3 A001_W3_3

A001_W3_3 Which siblings? （）

A002_W3 Except the ones in A001_W3, any other people do you live together now?How many____ 0…10 (Preload the household members name in our system in 2011 and 2013) choose all that apply. （2011 2013 ） ____ (**A002_W3**)

If answer to A002_W3 is larger than 0 record their names. A002_W3 0 ____ (**A002_W3_1_1**)

A004_W3 How many months did you live together in last year? _____ month (Preload all the names, ask each one) ____ （）

PROCEDURE :
• Ask each new one in A002_W3 A002_W3

A005_W3 Gender of [household member name] 1. Male
2. Female

A006_W3 When was [household member name] born? ____ (**A006_W3_1**) 1900…2013 (HBirthyear) Year ___ (**A006_W3_2**) 0…12 (HBirth- month) Month

Is it lunar or solar? (**A006_W3**)

1 ()

2 ()

A006_W3_1 How long have you been lived with [household member name] ? ____ 0...12

A006 What is the relationship of [household member name] to you? 1. Mother

2. Father
3. Mother-in-law /
4. Father-in-law /
5. Sibling
6. Brother-in-law, sister-in-law /
7. Child
8. Spouse of child /
9. Grandchild
10. Nanny
11. Driver
12. Other relative（specify）（） _____(**A006_1**)

D HEALTH STATUS AND FUNCTIONING

Type of Interview R	
XRType = REIW	This is a reinterview R
XRType = NEWIW	This is a new interview R
XRType = EXITIW	This is a exit R

Gender of Interview R		
R IS MALE	(XRGender = 1)	(XRGender = 1)
R IS FEMALE	(XRGender = 2)	(XRGender = 2)

Rs LAST IW Time (ZIWTime) (ZIWTime)

HEALTH CONDITIONS REPORTED IN LAST WAVE INTERVIEW:

ZDA005[i]	IF ZDA005[i]=Yes, R had kind of disabilities listed in DA005 at ZIWTime
ZDA006[i]	IF ZDA006[i]=Yes, R reported disabled time at ZIWTime
ZDA007[1]	IF ZDA007[1]=Yes, R had hypertension at ZIWTime
ZDA007[2]	IF ZDA007[2]=Yes, R had dyslipidemia at ZIWTime
ZDA007[3]	IF ZDA007[3]=Yes, R had diabetes or high blood sugar at ZIWTime
ZDA007[4]	IF ZDA007[4]=Yes, R had cancer or malignant tumor (excluding minor skin cancers) at ZIWTime
ZDA007[5]	IF ZDA007[5]=Yes, R had chronic lung diseases, such as chronic bronchitis, emphysema at ZIWTime
ZDA007[6]	IF ZDA007[6]=Yes, R had liver disease at ZIWTime
ZDA007[7]	IF ZDA007[7]=Yes, R had heart attack, coronary heart disease, angina, congestive heart failure, or other heart problems at ZIWTime
ZDA007[8]	IF ZDA007[8]=Yes, R had stroke at ZIWTime
ZDA007[9]	IF ZDA007[9]=Yes, R had kidney disease at ZIWTime
ZDA007[10]	IF ZDA007[10]= Yes, R had stomach or other digestive disease (except for tumor or cancer) at ZIWTime
ZDA007[11]	IF ZDA007[11]= Yes, R had emotional, nervous, or psychiatric problems at ZIWTime
ZDA007[12]	IF ZDA007[12]= Yes, R had memory-related disease at ZIWTime
ZDA007[13]	IF ZDA007[13]= Yes, R had arthritis or rheumatism at ZIWTime
ZDA007[14]	IF ZDA007[14]= Yes, R had asthma at ZIWTime
ZDA008[1]	IF ZDA008[1]= Yes, R had known R had hypertension at ZIWTime

ZDA008[5]	IF ZDA008[5]= Yes, R had known R had chronic lung diseases at ZIWTime
ZDA008[11]	IF ZDA008[11]= Yes, R had known R had emotional problems at ZIWTime
ZDA009[i]	When was the condition first dignosed or known by yourself?
ZDA027	IF ZDA027=Yes, R had started menopause at Rs last interview
ZDA028	Time of menopause
ZDA036	IF ZDA036= 1, R had cataract surgery<br>for one eye at ZIWTime
ZDA037	IF ZDA037=Yes, R had Glaucoma at ZIWTime
ZDA038	IF ZDA038=Yes, R had ever wear hearing aid at ZIWTime
ZDA040	IF ZDA040=Yes, R had lost all teeth at ZIWTime
ZDA059	IF ZDA059=Yes, R had ever smoked at Rs last interview
ZDA065	At what age did you start to smoke on a regular basis?

NOTE: NOTE ON PRELOADED HEALTH CONDITIONS:

IN THIS SECTION MUCH OF THE FLOW OF THE INTERVIEW AND THE PHRASING OF THE QUESTIONS DEPENDS ON WHETHER THE RESPONDENT REPORTED HAVING CERTAIN HEALTH CONDITIONS IN A PREVIOUS IW AND/OR CONFIRMED THEM IN THE LAST IW

.

DA HEALTH STATUS

PROCEDURE

SKIP PATTERN CHECKPOINT: SELF-REPORTED HEALTH STATUS

TWO SCALES ARE USED TO MEASURE SELF-REPORTED HEALTH STATUS. R WILL BE ASKED TO RATE THEIR HEALTH STATUS TWICE, ONCE AT THE BEGINNING OF THIS SECTION AND AGAIN AT THE END OF THE SECTION. QUESTION ORDER WILL BE ASSIGNED RANDOMLY.

IF R IS RANDOMLY ASSIGNED TO ORDER 1(SEC_DA_LIST= 1), SKIP TO DA001.

IF R IS RANDOMLY ASSIGNED TO ORDER 2(SEC_DA_LIST= 2), SKIP TO DA002.

. , . .

（DA = 1）DA001.

（DA = 2）DA002.

PART I: GENERAL HEALTH STATUS AND DISEASE HISTORY

DA001 Next, I have some questions about your health. Would you say your health is excellent, very good, good, fair, or poor? .

[IWERInterviewer should read all the following options]

1. Excellent
2. Very good
3. Good
4. Fair
5. Poor

DA002 Next, I have some questions about your health. Would you say your health is very good, good, fair, poor or very poor? . [IWERInterviewer should read all the following options]

1. Very good
2. Good
3. Fair
4. Poor
5. Very poor

**PROCEDURE** :
If XRType = REIW, ASK DA002_W2_1 DA002_W2_1

DA002_W2_1 Compared with your health when we talked with you in Rs LAST IW MONTH, YEAR, would you say that your health is better now, about the same, or worse? []

1. Better
2. About the same
3. Worse

**DA005 BRANCHPOINT**:
IF XRType = REIW WHO DID HAVE DISABILITY IN LAST IW TIME (ZDA005[i]=yes) AND (ZDA006[i]≠null), GO TO DA007 DA007.
IF XRType = REIW WHO DID HAVE DISABILITY IN LAST IW TIME (ZDA005[i]=yes) AND (ZDA006[i]=null), GO TO DA006 DA006
IF THIS IS A NEW INTERVIEW R OR THIS IS A REINTERVIEW R WHO DID NOT REPORT YES IN LAST WAVE (ZDA005[i]≠yes)

DA005 Do you have one of the following disabilities? 1. Physical disabilities

2. Brain damage/mental retardation /
3. Vision problem

4. Hearing problem
5. Speech impediment

DA006 BRANCHPOINT:
If DA005[i] = 1, ask DA006 DA006

DA006 In what year did you become disabled? [preload DA005]
If XRType = REIW, ask: Our records from your last interview in Rs LAST IW MONTH, YEARshow that you have had [preload ZDA005], In what year did you become disabled? [...] [preload ZDA005]_____Year
[IWER: Mark the year using four digits. 4]
[IWER: If the records from last wave is wrong, please note -9999. -9999]

PROCEDURE :
IF XRType = NEWIW, ask DA007 DA007

DA007 Have you been diagnosed with [conditions listed below, read one by one] by a doctor?
1. Hypertension
2. Dyslipidemia (elevation of low density lipoprotein, triglycerides (TGs),and total cholesterol, or a low high density lipoprotein level) ( ) 3. Diabetes or high blood sugar ( )
4. Cancer or malignant tumor (excluding minor skin cancers) ( )
5. Chronic lung diseases, such as chronic bronchitis , emphysema (excluding tumors, or cancer) ( )
6. Liver disease (except fatty liver, tumors, and cancer) ( )
7. Heart attack, coronary heart disease, angina, congestive heart failure, or other heart problems ( )
8. Stroke
9. Kidney disease (except for tumor or cancer) ( )
10. Stomach or other digestive disease (except for tumor or cancer) ( )
11. Emotional, nervous, or psychiatric problems
12. Memory-related disease ( )
13. Arthritis or rheumatism
14. Asthma

NOTE: The screen displays whether or not this condition was reported in Rs LAST IW

DA007_W2_1 Our records from your last interview in Rs last IW month, year show that you have had/not had [conditions listed below], is this right? / [],

1. Agree
2. Disagree

PROCEDURE :

For R reported in last iw that he/she had [conditions listed below] (ZDA007[i] = Yes or ZDA008[i] = Yes) []

If DA007_W2_1 = 1, Skip to next loop

If DA007_W2_1 = 2, Skip to DA007_W2_2 DA007_W2_2

PROCEDURE :

For R reported in last iw that he/she did not have [conditions listed below],(ZDA007[i] ≠ yes and ZDA008[i] ≠ yes) []

If DA007_W2_1 = 1, Skip to DA007_W2_2 DA007_W2_2

If DA007_W2_1 = 2, Skip to DA008_W2_1 DA008_W2_1

DA007_W2_2 Have you been diagnosed with [conditions listed below, read one by one] by a doctor [since Rs LAST IW MONTH, YEAR/ in the last two years]? ( )

1. Yes Skip to DA008_W2_1 DA008_W2_1
2. No Skip to next loop

DA008 BRANCHPOINT:

For XRType = REIW and XRType = NEWIW, ask DA008 if i = 1; 5; 11; and DA007[i] ≠ 1 or DA007_W2_2[i] ≠ 1. DA007DA007_W2_2 1511 DA008

DA008 Do you know if you have [preload the current choice in DA007]? [DA007 1511]

1. Yes
2. No
3. Dont know

PROCEDURE :

If DA007[i] = 1 or DA007_W2_2[i] = 1 or DA008[i] = 1

DA008_W2_1 How did you know that you had had [preload disease], through routine or charls physical examination, or any other? [] [] charls

1. Physical examination after had [preload disease] attack []
2. Physical examination after had ill
3. Physical examination organized by work unit
4. Physical examination organized by community
5. Charls physical examination charls
6. Other, please specify ______(**DA008_W2_1_1**)

**DA009 BRANCHPOINT**:<br>For XRType = REIW and XRType = NEWIW, if DA007[i]= 1 or DA007_W2_2[i] = 1 or DA008[i]= 1 or (((ZDA007[i] = 1 or ZDA008[i] = 1) and DA007_W2_1 = 1) and ZDA009[i]= null) or (ZDA007[i] ≠ 1 and ZDA008[i] ≠ 1 and DA007_W2_1 = 2), ask DA009 DA009

DA009 When was the condition first diagnosed or known by yourself? [...] ______ (**DA009_1**) Year ______(**DA009_2**) Age [IWER: Mark the year using four digits : 4]

**PROCEDURE** :<br>Answer DA010 if you have No. 2; 5; 6; 7; 9; 10; 12; 13 chronic dieases of DA007 DA007 25679 10 12 13 DA010

DA010 Are you now taking any of the following treatments to treat [...] or its complications (Check all that apply)? Taking Chinese traditional medicine, taking Western modern medicine, other treatments? [...] ( ) () [IWER: Read one by one]

1. Taking Chinese traditional medicine
2. Taking Western morden medicine
3. Other treatments
4. None of the above

**PROCEDURE** :<br>Answer DA010_W2_1 if you have hypertension or diabetes DA010_W2_1

DA010_W2_1 Is your [Blood pressure/ sugar] generally under control? [/]

1. Yes
2. No

**PROCEDURE** :<br>IF XRType = REIW that had condition in last wave ((ZDA007[i] = 1 or ZDA008[i] = 1) and DA007_W2_1[i] = 1) or (ZDA007[i] ≠ 1 and ZDA008[i] ≠ 1 and DA007_W2_1[i] = 2)<br>[...]

DA010_W2_2 Compared to when we interviewed you in Rs LAST IW MONTH, YEAR, is your condition better, about the same as it was then or worse? ( ) [...]

1. Better
2. Worse
3. Same

**PROCEDURE** : If respondents have hypertension then answer DA011 DA011.

DA011 Are you now taking any of the following treatments to treat or control your hypertension?(Check all that apply) Taking Chinese traditional medicine, taking Western modern medicine? () [IWER: Read one by one]

1. Taking Chinese traditional medicine
2. Taking Western modern medicine
3. None of the above

DA011_W2_1 **PROCEDURE** If XRType = REIW

Since Rs LAST IW MONTH, YEAR, have you had your blood pressure checked by a doctor or nurse? []

PROCEDURE OTHERWISE:

Have you ever had your blood pressure checked by a doctor or nurse?

1. Yes
2. No Skip to DA013 DA013

DA011_W2_2 When did you last have it checked?

Year _____(**DA011_w2_2_1**) Month ______(**DA011_w2_2_2**)

DA012 During last year (last 12 months), how many times have you had blood pressure examination? (12) _____0 : : : 999 Times

DA012_W3 Have you had blood pressure examination by community/village doctors regularly? /

1. Yes
2. No Skip to DA013 DA013

DA012_W3_1 How often did you have blood pressure examination by community/village doctors? /

1. Once a week

2. Once half a month
3. Once a month
4. Once every two months
5. Once every three months
6. Once half a year
7. Once a year

DA012_W3_2 Do you have to pay for the blood pressure examination by community/village doctors? /
1. Yes
2. No

**PROCEDURE** : If respondents have hypertension then answer DA013 DA013.

DA013 Have your care providers ever given you health education/advice on the following (check all that apply)? Weight control, exercise, diet and/or smoking control? () [IWER: Read one by one]
1. Weight control
2. Exercise
3. Diet
4. Smoking control
5. None of the above

**PROCEDURE** If respondents have diabetes, then answer DA014-DA016 DA014-DA016.

DA014 Are you now taking any of the following treatments to treat or control your diabetes? ( ) [IWER: Read one by one]
1. Taking Chinese traditional medicine
2. Taking Western modern medicine
3. Taking insulin injections
4. None of the above

DA015 During last year (last 12 months), how many times have you had the following? (12)
1. Blood glucose test (**DA015_1**)_______ 0…999 Times
2. Urine glucose test (**DA015_2**)______ 0…999 Times
3. Fundus examination (**DA015_3**)_____ 0…999 Times
4. Micro-albuminuria test (**DA015_4**)_______ 0…999 Times 5. None of the above Skip to DA016 DA016

DA016_W3 Have you had diabetes examination by community/village doctors regularly? /

1. Yes
2. No Skip DA016_W3_1 and DA016_W3_2 DA016_W3_1DA016_W3_2

DA016_W3_1 How oftern did you have diabetes examination by community/village doctors? /

1. Once a week
2. Once half a month
3. Once a month
4. Once every two months
5. Once every three months
6. Once half a year
7. Once a year

DA016_W3_2 Do you have to pay for diabetes examination done by community/village doctors? /

1. Yes
2. No

DA016 Have your care providers ever given you health education/advice on the following? (check all that apply) () [IWER: Read one by one]

1. Weight control
2. Exercise
3. Diet
4. Smoking control
5. Foot self-care
6. None of the above

PROCEDURE If reinterview respondents have heart attack, then answer DA007_W2_5 DA007_W2_5.

DA007_W2_5 [Since Rs LAST IW MONTH, YEAR/In the last two years], have you had a heart attack?

1. Yes
2. No → Skip DA007_W2_6 DA007_W2_6

DA007_W2_6 When was [his/her] (most recent) heart attack? ____ Year (**DA007_W2_6_1**) ______ Age (**DA007_W2_6_2**)

PROCEDURE If respondents have cancer or maligant tumor (excluding minor skin cancers), answer DA017 and DA018 ( ) DA017 DA018.

DA017 In which organ or part of your body do you have cancer? Including the origins and metastasis of tumor.（circle all that apply） .（） [IWER: Read one by one. We should still ask R even if he/she has already been cured]

1. Brain
2. Oral cavity
3. Larynx
4. Other pharynx
5. Thyroid
6. Lung
7. Breast
8. Oesophagus
9. Stomach
10. Liver
11. Pancreas
12. Kidney
13. Prostate
14. Testicle
15. Ovary
16. Cervix
17. Endometrium
18. Colon or rectum
19. Bladder
20. Skin
21. Non-Hodgkin lymphoma （）
22. Leukemia
23. Other organ （**DA017_1** ）

DA018 Have you taken any of the following treatments to treatyour cancer or relieve its/their symptoms (e.g., pain, nausea, etc.) in the past two years? (Check all that apply) Taking Chinese traditional medicine, taking Western modern medicine, chemotherapy, surgery, radiation therapy? （）

[IWER: Read one by one]

1. Taking Chinese traditional medicine
2. Taking Western morden medicine
3. Chemotherapy
4. Surgery
5. Radiation therapy
6. None of the above

[F1 ⑴ . （）

.
(2) . (3)]

**PROCEDURE** If respondents have stroke, then answer DA019 DA019.

DA019 Are you now taking any of the folloing treatments because of your stroke?(Check all that apply) Taking Chinese traditional medicine, taking Western modern medicine, physical therapy, acupuncture and moxibustion, occupational therapy? ()
[IWER: Read one by one]

1. Taking Chinese traditional medicine
2. Taking Western morden medicine
3. Physical therapy
4. Acupuncture and moxibustion
5. Occupational therapy
6. None of the above

[F1 (1) () .
(2) . . .
(3) / /]

**PROCEDURE** If reinterview respondents have stroke, then answer DA019_W2_1 and DA019_W2_2 DA019_W2_1DA019_W2_2.

DA019_W2_1 Since Rs LAST IW MONTH, YEAR, has a doctor told you that you had another stroke? []

1. Yes
2. No Skip DA019_W2_2 DA019_W2_2

DA019_W2_2 When was your most recent stroke? _ Year _____ Age

**PROCEDURE** If respondents have emotional, nervous, or psychiatric problems, then answer DA020 DA020

DA020 Are you now taking any of the folloing treatments for your emotional, nervous, or psychiatric problems?(Check all that apply) Receiving psychiatric or psychological treatment, taking anti depressants, taking tranquilizers or sleeping pills? () [IWER: Read one by one]

1. Receiving psychiatric or psychological treatment 2. Taking anti depressants
3. Taking tranquilizers or sleeping pills
4. None of the above

DA021 **PROCEDURE** If XRType = NEWIW

Have you ever been in a traffic accident or any other kind of major accidental injury and received medical treatment?

PROCEDURE If XRType = REIW

Have you ever been in a traffic accident or any other kind of major accidental injury and received medical treatment [since Rs LAST IW MONTH, YEAR/ in the last two years]? (),

1. Yes
2. No → Skip to DA023 DA023

DA022 Does your injury caused by the accident limit your daily activities?

1. Yes
2. No

DA023 **PROCEDURE** If XRType = NEWIW

Have you fallen down?

PROCEDURE If XRType = REIW

Have you fallen down since Rs last IW month, year? [ ]

1. Yes
2. No → Skip to DA025 DA025

DA024 How many times have you fallen down seriously enough to need medical treatment? ?______ times

DA025 **PROCEDURE** If XRType = NEWIW

Have you ever fractured your hip?

PROCEDURE If XRType = REIW

Have you fractured your hip since we talked in Rs last IW month, year? []

1. Yes
2. No

[F1 . , .]

**PROCEDURE** IF R IS MALE, SKIP TO DA029 DA029.

BRANCHPOINT:

IF XRType = REIW reported in previous wave that has not started menopause, skip to DA027 DA027

IF XRType = REIW reported in previous wave that has started menopause, skip to DA032 DA032

IF XRType = NEWIW DA026-DA028

DA026 When did you begin the menarche?

_____ 1900...2015 (**DA026_1**) Year Or Age ______ 1...120 (**DA026_2**) Years

[IWER: Mark the year using four digits : 4]

DA027 Have you started menopause?

1. Yes
2. No → Skip to DA032 DA032

DA028 When did you begin the menopause?

_____ (**DA028_1**) Year Or Age _____ (**DA028_2**) Years → Skip to DA032 DA032

[IWER: Mark the year using four digits : 4]

DA029 **PROCEDURE** If XRType = NEWIW and is male

Have you ever been diagnosed with a prostate illness, such as prostate hyperplasia (excluding prostatic cancer) ? ( )

PROCEDURE If XRType = REIW and is male

Have you ever been diagnosed with a prostate illness, such as prostate hyperplasia (excluding prostatic cancer) since we talked (in Rs LAST IW MONTH,YEAR/in the last two years)? ( ) ( )

1. Yes → Skip to DA030 DA030
2. No

[F1 , ,]

DA029_W2_1 Do you know if you had a prostate illness, such as prostate hyperplasia (excluding prostatic cancer) ? ( )

1. Yes
2. No → Skip to DA032 DA032
3. Dont know → Skip to DA032 DA032

DA030 When was the condition first diagnosed? __ (**DA030_1**)Year Or Age _____ (**DA030_2**) Years [IWER: Mark the year using four digits : 4]

DA031 Are you now taking medication or other treatment for your prostate illness?
1. Yes
2. No

DA032 Now I have some questions about your eyesight. Do you usually wear glasses or corrective lenses? . ( )
1. Yes
2. Legally blind → Skip to DA038 DA038
3. No
4. Sometimes

DA033 How good is your eyesight for seeing things at a distance, like recognizing a friend from across the street (with glasses or corrective lenses if you wear them)? Would you say your eyesight for seeing things at a distance is excellent, very good, good, fair, or poor? ( ) .
1. Excellent
2. Very good
3. Good
4. Fair
5. Poor

DA034 How good is your eyesight for seeing things up close, like reading ordinary newspaper print (with glasses or corrective lenses if you wear them)? Would you say your eyesight for seeing things up close is excellent, very good, good, fair, or poor?
1. Excellent
2. Very good
3. Good
4. Fair
5. Poor

**PROCEDURE** If XRType = NEWIW

DA035 Have you ever had cataract surgery?

1. Yes
2. No → Skip to DA037 DA037

**PROCEDURE** If XRType = REIW and R reported in last IW that had cataract surgery on one eye (ZDA036 = 1) （ZDA036 = 1）:

DA035_W2_1 Have you had another cataract surgery since we last talked to you (in Rs LAST IW MONTH, YEAR) other than what you told us about then? （） （）

1. Yes
2. No

**PROCEDURE** If XRType = REIW and R did not report in last IW that had cataract surgery ((ZDA035 ≠ 1)

DA035_W2_2 Have you had cataract surgery (since Rs LAST IW MONTH, YEAR/in the last two years)? （）

1. Yes
2. No → Skip to DA037 DA037

DA036 Have you had cataract surgery on both eyes or just one?

1. One eye only
2. Both eyes

**PROCEDURE** If XRType = NEWIW or XRType = REIW and R did not report in last IW that had glaucoma (ZDA037 ≠ 1) （ZDA037 ≠ 1）

DA037 Has a doctor/nurse/paramedical/doctor of traditional Chinese medicine doctor ever treated you for glaucoma?

1. Yes
2. No

**PROCEDURE** IfXRType = REIW and R reported in last IW that had glaucoma (ZDA037 = 1) （ZDA037 = 1）

DA037_W2 You told us you had glaucoma, has a doctor/nurse/paramedical/ doctor of traditional Chinese medicine doctor ever treated you for glaucoma relapses since last iw time? []

1. Yes

2. No
3. Never had glaucoma

**PROCEDURE** If R reported in previous IW that wears hearing aid (ZDA038 = 1), skip to DA039 DA039

DA038 Now I have some questions about your hearing. Do you ever wear a hearing aid? .

1. Yes
2. No

DA039 Is your hearing very good, good, fair, poor, or very poor (with a hearing aid if you normally use it and without if you normally dont)? Would you say your hearing is excellent, very good, good, fair, or poor? ( )

1. Excellent
2. Very good
3. Good
4. Fair
5. Poor

**PROCEDURE** If XRType = NEWIW or XRType = REIW that did not lost all teeth in last iw(ZDA040 ≠ 1), ask DA040 , DA040

DA040 Have you lost all of your teeth?

1. Yes
2. No

DA041 Are you often troubled with any body pains? 1. Yes

2. No skip to DA045DA045

DA042 On what part of your body do you feel pain? Please list all parts of body you are currently feeling pain. .

1. Head (Headache)
2. Shoulder
3. Arm
4. Wrist
5. Fingers
6. Chest
7. Stomach (Stomachache)
8. Back

9. Waist
10. Buttocks
11. Leg
12. Knees
13. Ankle
14. Toes
15. Neck
16. Other, please specify ______(**DA042_1**)

DA042_W2_1 Are you taking measures to reduce the pain? ()

1. Taking Chinese traditional medicine
2. Taking Western modern medicine
3. Acupuncture treatment
4. Professional massage therapy
5. Other, please specify
6. None

DA045 Are there any other medical diseases or conditions that are important to your health now that we have not talked about?

1. Yes
2. No → Skip DA046 DA046

DA046 What illness is that? ______

DA048 How would you evaluate your health during childhood, up to and including age 15? Excellent, very good, good,fair, poor? 15 （ 15 ）

1. Excellent
2. Very Good
3. Good
4. Fair
5. Poor

PART II: LIFESTYLE AND HEALTH BEHAVIORS

DA049 During the past month, how many hours of actual sleep did you get at night (average hours for one night)? (This maybe shorter than the number of hours you spend in bed.)
() _ 0...24 hours

DA050 During the past month, how long did you take a nap after lunch? ______Minutes
[IWERIfR didnt take a nap, please record for 0 0]

PROCEDURE DA051 will be presented only to a random subsample of households (half). Main respondent and spouse in the selected households should answer DA051-DA055 DA051-DA055. DA051 .

DA051 Now we would like to ask about the amount of time you spend on different types of physical activities in a usual week.

PHYSICAL ACTIVITIES (KKTYPE)	**DA051**<br>During a usual week, did you do any [...] for at least 10 minutes continuously? [...]	**DA052**<br>During a usual week, on how many days did you do [...] for at least 10 minutes? [...]	How much time did you usually spend doing [...] on one of those days? [...] [...]	
A. Now, think about all the vigorous activities requiring hard/high-intensity physical effort that you do in a usual week. Vigorous activities make you breathe much harder than normal and may include heavy lifting, digging, plowing, aerobics, fast bicycling, and cycling with a heavy load. Think only about those physical activities that you did for at least 10 minutes at a time.<br>.<br>.<br>.	2 No #<br>1 Yes !	________ 1...7 days	**DA053**<br>1. < 2 hours<br>2. ≥ 2 hours	**DA054**<br>1. < 30 minutes<br>2. ≥ 30 minutes<br>**DA055**<br>3. < 4 hours<br>4. ≥ 4 hours
B. Now think about activities which take moderate physical effort that you do in a usual week. Moderate physical activities make you breathe somewhat harder than normal and may include carrying light loads, bi-cycling at a regular pace, or mopping the floor. Again, think about only those physical activities that you did for at least 10 minutes at a time. . . .	2 No #<br>1 Yes !	________ 1...7 days	**DA053**<br>1. < 2 hours<br>2. ≥ 2 hours	**DA054**<br>1. < 30 minutes 2. ≥ 30 minutes<br>**DA055**<br>3. < 4 hours<br>4. ≥ 4 hours
C. Now think about the time you spend walking in a usual week. This includes at work and at home, walk-ing to travel from place to place, and any other walking that you might do solely for recreation, sport, exercise, or leisure. . .	2 No #<br>1 Yes !	________ 1...7 days	**DA053**<br>1. < 2 hours<br>2. ≥ 2 hours	**DA054**<br>1. < 30 minutes<br>2. ≥ 30 minutes<br>**DA055**<br>3. < 4 hours<br>4. ≥ 4 hours

DA051_1 Whats the purpose for doing these physical activities, for entertainment, job de-mand or exercise in doing these physical activities?

1. Job demands
2. Entertainments

3. Exercise
4. Other, please specify

[ACTIVITIES IN LAST MONTH]

DA056 Have you done any of these activities in the last month? (Code all that apply) （）

1. Interacted with friends
2. Played Ma-jong,played chess, played cards, or went to community club
3. Provided help to family, friends, or neighbors who do not live with you
4. Went to a sport, social, or other kind of club 5. Took part in a community-related organization 6. Done voluntary or charity work
7. Cared for a sick or disabled adult who does not live with you
8. Attended an educational or training course 9. Stock investment （ ）
10. Used the Internet
11. Other
12. None of these

[CHECK: You cannot select None of these together with any other answer. Please change your answer .]

**PROCEDURE** If DA056= 10, ask DA056_W3 DA056= 10DA056.

DA056_W3 ?

1. Desktop computer
2. Laptop computer
3. Tablet computer （ IPAD）
4. Cellphone
5. Other devices ______ （DA056_W3_ 1）

**PROCEDURE**

DA057 Frequency of activity in the last month

How often in the last month [did/have][you] [do voluntary or charity work/cared for a sick or disabled adult/provided help to family, friends or neighbors/attended an educational or training course/ Interacted with friends /go to a sport,social or other kind of club/taken part in a community-related organization]? Almost daily, almost every week, or not regularly? （ ）

1. Almost daily
2. Almost every week
3. Not regularly

[INTRO: Next, I would like to ask whether you have had the habit of smoking cigarettes/smoking a pipe/chewing tobacco, now or in the past. By smoking we mean smoking more than 100 cigarettes in your life （ 100 ）]

BRANCHPOINT:
If XRType = REIW and R reported ever smoked (ZDA059 = 1), skip to DA061 DA061
If XRType = REIW and R did not report ever smoked (ZDA059≠ 1); orXRType = NEWIW, ask DA059 DA059

DA059 Have you ever chewed tobacco, smoked a pipe, smoked self-rolled cigarettes, or smoked cigarettes/cigars? （）
1. Yes
2. No Skip to DA067 DA067

DA061_W3 If XRType = REIW, R reported ever smoked (ZDA059= 1) and he/she did not answer DA061in last interview, ask: DA061 In last interview did you have the habit or have you totally quit?
1. Still have
2. Quit
3. Never smoked

DA061 PROCEDURE If XRType = REIW
Our records from your last interview show that you have ever smoked,
Do you still have the habit or have you totally quit? **PROCEDURE** If XRType = NEWIW
Do you still have the habit or have you totally quit?
1. Still have Skip DA062 DA062
2. Quit
3. Never smoked Skip to DA067 DA067

DA060 Which products did/do you normally use?
1. Smoking a pipe （）
2. Smoking self-rolled cigarettes
3. Filtered cigarette
4. Unfiltered cigarette

5. Cigar
6. Water cigarettes

DA062 At what age did you totally quit smoking? Age
______1...120 (**DA062_1**) Years or ______1900...2015 (**DA062_2**) Year [IWER: Mark the year using four digits : 4]

**PROCEDURE** If DA060= 3 or 4, ask DA063 and DA064 DA060= 3 or 4 DA063DA064.

DA063 In one day about how many cigarettes do/did you consume [preload:now/before totally quitting]? [/] ______Cigarettes

DA064 How much does/did it cost per pack = 20 cigarettes? [/] (20) ______Yuan [IWER: Prompt R: we are asking price at that time, not current price]

**PROCEDURE** If XRType = REIW that has been asked when started smoking, skip DA065 DA065.

DA065 At what age did you start to smoke on a regular basis?
Age ______ 1...120 (**DA065_1**) years Or ______ 1900...2015 (**DA065_2**) Year
[IWER: Mark the year using four digits : 4]

DA067 Did you drink any alcoholic beverages, such as beer, wine, or liquor in the past year? How often?

1. Drink more than once a month.
2. Drink but less than once a month Skip to DA069 DA069
3. None of these Skip to DA069 DA069

DA068 What type of alcoholic beverages did you drink? Liquor, wine, or beer? (code all that apply) ( )

1. Liquor, including white liquor, whisky, and others Skip to DA071 DA071
2. Beer Skip to DA071 DA071
3. Wine or rice wine Skip to DA071 DA071

**PROCEDURE** If XRType = REIW that has been asked whether drinking alcoholic bev- erages in the past, skip DA069 DA069.

DA069 Did you ever drink alcoholic beverages in the past? How often?

1. I never had a drink. → Skip to procedure before DA079 DA079
2. I used to drink less than once a month. → Skip to procedure before DA079 DA079
3. I used to drink more than once a month.

**PROCEDURE** IfXRType = REIW that has been asked when quiting alcoholic beverages in the past, skip DA070 DA070.

DA070 When did you quit or reduce drinking?

_____ 1900…2015 (**DA070_1**)Year or Age : _____ 1…120 (**DA070_2**) Years

[IWER: Record year in 4 digits 4]

**PROCEDURE** If XRType = REIW that has been asked when started drinking, skip DA071 DA071 .

DA071 When did you start drinking?

_____ 1900…2015 (**DA071_1**)Year or Age : _____ 1…120 (**DA071_2**) Years

[IWER: Record year in 4 digits. 4]

[INTRO: Now, I am going to ask you how often and how much you drank during the past year. Please tell me how often you drank per month, and how much you drank at a time on average. I will repeat the questions for different types of alcoholic beverages . .]

**CAPI** If DA068= 1, ask DA072 DA068= 1DA072.

DA072 How often did you drink liquor, including white liquor, whisky, and others per month in the last year?

1. Once a month
2. 2-3 times a month 2-3
3. Once a week
4. 2-3 times a week 2-3
5. 4-6 times a week 4-6
6. Once a day
7. Twice a day
8. More than twice a day

DA073 The last time you drank liquor last year, how many liang of liquor did you drink? (1 liang = 50cc/50ml) （1 = 50 ） ____ Liang

**CAPI** If DA068 = 2, ask DA074 DA068 = 2DA074.

DA074 How many times per month did you drink beer in the last year?

1. Once a month
2. 2-3 times a month 2-3
3. Once a week
4. 2-3 times a week 2-3
5. 4-6 times a week 4-6
6. Once a day
7. Twice a day
8. More than twice a day

DA075 The last time you drank beer last year, how many bottles of beer did you drink? (1bottle= 2:5 mugs, 1mug= 220cc) ?（1 =2.5 1 =220 ） _____ (**DA075_1**)0...120 Bottles or _____(**DA075_2**)0...300 Mugs

**CAPI** If DA068 = 3, ask DA076 DA068 = 3DA076.

DA076 How often did you drink wine or rice wine per month in the last year?

1. Once a month
2. 2-3 times a month 2-3
3. Once a week
4. 2-3 times a week 2-3
5. 4-6 times a week 4-6
6. Once a day
7. Twice a day
8. More than twice a day

DA077 The last time you drank it last year, how many liang of wine did you drink? (1 liang=50cc) （1 =50 ） _____0.00...100.00 Liang

[IWER: Please do not ask proxy the following question DA079 and DA080 DA079 and DA080]

DA079 How would you rate your health status? Would you say your health is very good, good, fair, poor or very poor?

1. Very good
2. Good
3. Fair
4. Poor

5. Very poor

DA080 Next I have some questions about your health. Would you say your health is excellent, very good, good, fair, or poor? [IWERinterviewer should read all the following options]

1. Excellent
2. Very good
3. Good
4. Fair
5. Poor

INTERVIEWER CHECK AGE OF RESPONDENT? .	**1.** < 65 **YEAR ! COLUMN A A** **2.** 65 - 69 **YEAR ! COLUMN B B** 74 **3.** 70 - **YEAR ! COLUMN C C** 79 **4.** 75 - **YEAR ! COLUMN D D** 84 **5.** 80 - **YEAR ! COLUMN E E**				**6.** 85 - 89 **YEAR ! COLUMN F F** **7.** 90 - 94 **YEAR ! COLUMN G G** **8.** 95 - 99 **YEAR ! COLUMN H H** **9.** ≥ 100 **YEAR ! COLUMN I I**			
	A	B	C	D	E	F	G	H	I
AGE	75 years	80 years	85 years	90 years	95 years	100 years	105 years	110 years	115 years
**DA081** Suppose there are 5 steps, where the lowest step represents the smallest chance and the highest step represents the highest chance, on what step do you think is your chance in reaching the age of [...]? . 1 Almost impossible 2 Not very likely 3 Maybe 4 Very likely 5 Almost certain	1 2 3 4 5	1 2 3 4 5	1 2 3 4 5	1 2 3 4 5	1 2 3 4 5	1 2 3 4 5	1 2 3 4 5	1 2 3 4 5	1 2 3 4 5

DB FUNCTIONAL LIMITATIONS AND HELPERS

CAPI If R is younger than 50 (year of birth is after 1965) and if DA001 = 1 or 2 or DA002 = 1 or 2 and DA005 = 2 and DA007 = 2 and DA008 = 2, skip DB001- DB015 50 （ 1965 ） DB001-DB015.

[We need to understand difficulties people may have with various activities because of a health or physical problem. Please tell me whether you have difficulty performing any of the following tasks on a regular basis. Exclude any difficulties that you expect to last less than three months . （）. .]

DB001 Do you have any difficulty with running or jogging about 1 Km? 1
1. No, I dont have any difficulty → Skip to DB004 DB004
2. I have difficulty but can still do it
3. Yes, I have difficulty and need help
4. I can not do it

DB002 Do you have difficulty with walking 1 km? 1 1. No, I dont have any difficulty → Skip DB003 DB003
2. I have difficulty but can still do it
3. Yes, I have difficulty and need help
4. I can not do it

DB003 Do you have difficulty with walking 100 metres? 100 1. No, I dont have any difficulty
2. I have difficulty but can still do it
3. Yes, I have difficulty and need help
4. I can not do it

DB004 Do you have difficulty with getting up from a chair after sitting for a long period?
1. No, I dont have any difficulty
2. I have difficulty but can still do it
3. Yes, I have difficulty and need help
4. I can not do it

DB005 Do you have difficulty with climbing several flights of stairs without resting?
1. No, I dont have any difficulty
2. I have difficulty but can still do it
3. Yes, I have difficulty and need help
4. I can not do it

DB006 Do you have difficulty with stooping, kneeling, or crouching?
1. No, I dont have any difficulty
2. I have difficulty but can still do it
3. Yes, I have difficulty and need help
4. I can not do it

DB007 Do you have difficulty with reaching or extending your arms above shoulder level? (he/she is regarded as not having difficulty only if he/she can extend both of his/her arms, otherwise he/she is regarded as having difficulty.) ()

1. No, I dont have any difficulty
2. I have difficulty but can still do it
3. Yes, I have difficulty and need help
4. I can not do it

DB008 Do you have difficulty with lifting or carrying weights over 10 jin, like a heavy bag of groceries? 10 ( )

1. No, I dont have any difficulty
2. I have difficulty but can still do it
3. Yes, I have difficulty and need help
4. I can not do it

DB009 Do you have difficulty with picking up a small coin from a table?

1. No, I dont have any difficulty
2. I have difficulty but can still do it
3. Yes, I have difficulty and need help
4. I can not do it

**CAPI** If (DB001 = 1 & DB003 = 1 : : : DB009 = 1), then skip to DB016 DB001~ DB009 DB016

[Here are a few more everyday acivities. Please tell me if you have any difficulties with these because of a physical, mental, emotional or memory problem. Again, exclude any that you expect to last less than three months . .]

DB010 Because of health and memory problems, do you have any difficulty with dressing? Dressing includes taking clothes out from a closet, putting them on, buttoning up, and fastening a belt. .

1. No, I dont have any difficulty → Skip to DB011 DB011
2. I have difficulty but can still do it
3. Yes, I have difficulty and need help
4. I can not do it

DB010_W2 Does anyone ever help you dress? 1. Yes
2. No

DB011 Because of health and memory problems, do you have any difficulty with bathing or showering?

1. No, I dont have any difficulty → Skip to DB012 DB012
2. I have difficulty but can still do it
3. Yes, I have difficulty and need help
4. I can not do it

DB011_W2 Does anyone ever help you bathe?

1. Yes
2. No

DB012 Because of health and memory problems, do you have any difficulty with eating, such as cutting up your food? (Definition: By eating, we mean eating food by oneself when it is ready) (.)

1. No, I dont have any difficulty → Skip to DB013 DB013
2. I have difficulty but can still do it
3. Yes, I have difficulty and need help
4. I can not do it

DB012_W2 Does anyone ever help you eat?

1. Yes
2. No

DB013 Do you have any difficulty with getting into or out of bed? 1. No, I dont have any difficulty → Skip to DB014 DB014

2. I have difficulty but can still do it
3. Yes, I have difficulty and need help
4. I can not do it

DB013_W2 Does anyone ever help you get in or out of bed? 1. Yes

2. No

DB014 Because of health and memory problems, do you have any difficulties with using the toilet, including getting up and down?

1. No, I dont have any difficulty → Skip to DB015 DB015
2. I have difficulty but can still do it
3. Yes, I have difficulty and need help
4. I can not do it

DB014_W2 Does anyone ever help you use the toilet? 1. Yes

2. No

DB015 Because of health and memory problems, do you have any difficulties with controlling urination and defecation? If you use a catheter (conduit) or a pouch by yourself, then you are not considered to have difficulties. ( )

1. No, I dont have any difficulty
2. I have difficulty but can still do it
3. Yes, I have difficulty and need help
4. I can not do it

DB016 Because of health and memory problems, do you have any difficulties with doing household chores? (Definition: By doing household chores, we mean house cleaning, doing dishes, making the bed, and arranging the house) ( ) [IWER: If R cannot mop the floor, but can scrub, or R cannot fold heavy bedding, but is able to do light ones, then mark (3) (3)]

1. No, I dont have any difficulty → skip to DB017 DB017
2. I have difficulty but can still do it
3. Yes, I have difficulty and need help
4. I can not do it

DB016_W2 Does anyone help you do household chores? 1. Yes
2. No

DB017 Because of health and memory problems, do you have any difficulties with preparing hot meals? (Definition: By preparing hot meals, we mean preparing ingredients, cooking, and serving food) ( ) [IWER: If another person prepares ingredients or ifR can cook rice, but is not able to prepare side dishes, then mark (3) (3)]

1. No, I dont have any difficulty → skip to DB018 DB018
2. I have difficulty but can still do it
3. Yes, I have difficulty and need help
4. I can not do it

DB017_W2 Does anyone help you prepare hot meals? 1. Yes
2. No

DB018 Because of health and memory problems, do you have any difficulties with shopping for groceries? By shopping, we mean deciding what to buy and paying for it.

.

1. No, I dont have any difficulty → skip to DB035 DB035
2. I have difficulty but can still do it
3. Yes, I have difficulty and need help
4. I can not do it

DB018_W2 Does anyone help you shop for groceries? 1. Yes
2. No

DB035 Because of health and memory problems, do you have any difficulties with making phone calls?

1. No, I dont have any difficulty → skip to DB020 DB020
2. I have difficulty but can still do it
3. Yes, I have difficulty and need help
4. I can not do it
5. Not relevant to me (no phone) → skip to DB020 DB020

DB035_W2 Does anyone help you make telephone calls?

1. Yes
2. No

DB020 Because of health and memory problems, do you have any difficulties with taking medications? By taking medications, we mean taking the right portion of medication right on time. .

1. No, I dont have any difficulty → skip to DB019 DB019
2. I have difficulty but can still do it
3. Yes, I have difficulty and need help
4. I can not do it

DB020_W2 Does anyone help you take medications? 1. Yes
2. No

DB019 Because of health and memory problems, do you have any difficulties with managing your money, such as paying your bills, keeping track of expenses, or managing assets?

1. No, I dont have any difficulty → skip DB019_W2 DB019_W2
2. I have difficulty but can still do it
3. Yes, I have difficulty and need help
4. I can not do it

DB019_W2 Does anyone help you manage your money? 1. Yes
2. No

**PROCEDURE** If DB010_W2= 1 or DB011_W2= 1 or DB012_W2= 1 or DB013_W2= 1 or DB014_W2= 1 or DB016_W2= 1 or DB017_W2= 1 or DB018_W2= 1 or DB035_W2= 1 or DB020_W2= 1 or DB019_W2= 1, ask DB022_W3_1; otherwise, skip to DB029

DB022_W3_1 Who most often helps you with [make sure we ask this only once for all these activities; do not ask for each problem separately] (dressing, bathing, eating, getting out of bed, using the toilet, controlling urination and defecation, doing chores, preparing hot meals, shopping, managing money, making phone calls, taking medications) (May choose up to 3 persons)? （ ） （）

1. Spouse
2. Father, Mother, Father-in-law, Mother-in-law
3. Children, Childrens spouses, Grandson, Granddaughter / /
4. Sibling, Brother-in-law, Sister-in-law, Sibling of spouse, Children of sibling, Sibling of spouse, Brother-in-law, Sister-in-law of spouse, Children of brother-in-law, Children of sister-in-law
5. Other relative
6. Paid helper (such as nanny) （） ______ （**DB022_W3_1_1**）
7. Volunteer or Employee of facility
8. Nursing home
9.
10. Other

[Note: employee(s) of facility appears on list only for an R currently living in a nursing home or who was living in a nursing home or hospice when he/she died]

**PROCEDURE** If DB022_W3_1= 2, ask DB023_W3_1

DB023_W3_1 Father, mother, father-in-law, monther-in-law, Who help you most? （）

1. Father
2. Mother
3. Father-in-law /
4. Mother-in-law /

**PROCEDURE** If DB022_W3_1= 3, ask DB023_W3_2

DB023_W3_2 For the children, children-in-law, grandson, granddaughter who helped you, which childrens family are they from? // （）

1-25 [Preload childrens name] []
26 None of the above

[For each helpers from every childrens family, repeat the question]

DB023_W3_3 []
1 [Preload childs name] himself/herself []
2 [Preload childs name] his/her spouse []
3 [Preload childs name] his/her children [] ( ) [] _____ (**DB023_W3_3_1**)

**PROCEDURE** If DB022_W3_1= 4, ask DB023_W3_4

DB023_W3_4 For the siblings, spouse and children of siblings, siblings of your spouse, spouse and children of siblings of your spouse who helped you, which siblings family are they from? ( )
1-15 [Preload siblings name] []
16-30 [Preload spouses siblings name] []
99 None of the above

[For each helpers from every siblings, siblings of spouse, repeat the question]

DB023_W3_5 For the family members of [Preload name of siblings, siblings of spouse], who help you in person? [/]
1 [Preload siblings (of spouse) name] himself/herself [( )]
2 [Preload siblings (of spouse) name] his/her spouse [( )]
3 For the children from [Preload siblings (of spouse) name], how many children from [Preload siblings (of spouse) name] help you in person? [( )] [( )] __ (**DB023_W3_5_1**)

**PROCEDURE** If DB022_W3_1= 5, ask DB023_W3_6

DB023_W3_6 The number of the relatives who help you in person _____Whats their relationship with you _____(**DB023_W3_6_1**)

**PROCEDURE** If DB022_W3_1= 10, ask DB023_W3_7

DB023_W3_7 The number of the others who help you in person _____Whats that persons relationship with you _____(**DB023_W3_7_1**)

**Note**: 7 DB023_W3_9

DB023_W3_9 From all he helpers list below, please select the most important 7 helpers for you 7 .

[Name of all helpers] []

**PROCEDURE** For each helper chosen in DB022_W3_1-DB023_W3_7, ask DB023-DB026 DB022_W3_1-DB023_W3_7 DB023-DB026.

DB023 During the last month, on about how many days did [helpers name chosen from DB022_W3_1-DB023_W3_7] help you? [DB022_W3_1-DB023_W3_7] _____ 1...31 Days

DB024 On the days [helpers name chosen from DB022_W3_1-DB023_W3_7] helps you, about how many hours per day is that? [DB022_W3_1-DB023_W3_7] / _____ 1...24 Hours [IWER: less than an hour, mark 1 1]

DB025 Is he/she living in your home? / 1. Yes

2. No

DB026 Is [helpers name chosen from DB022_W3_1-DB023_W3_7] paid to help you? [DB022_W3_1-DB023_W3_7]

1. Yes
2. No

**PROCEDURE** If R paid for help in DB026, skip to DB027 DB026 DB027. IfR did not pay for help, skip to DB029 DB029.

DB027 About how much in total did you pay (including value of the goods you gave them as repayment for their help) for the help during the past month? ( ) _____ Yuan

DB028 Who contributed most to paying this cost? Please choose one person who paid the most. .

1. Spouse
2. Father, Mother, Father-in-law, Mother-in-law
3. Children, Childrens spouses, Grandson, Granddaughter / /
4. Sibling, Brother-in-law, Sister-in-law, Sibling of spouse, Children of sibling, Sibling of spouse, Brother-in-law, Sister-in-law of spouse, Children of brother-in-law, Children of sister-in-law

5. Other relative
6. Paid helper(such as nanny) ( )
7. Volunteer or Employee of facility
8. Nursing home
9. Community
10. Other

**PROCEDURE** If DB028= 2, ask DB028_W3_1

DB028_W3_1 Parents, parents-in-law, which of them paid the most?
1. Father
2. Mother
3. Father-in-law /
4. Mother-in-law /

**PROCEDURE** If DB028= 3, ask DB028_W3_2

DB028_W3_2 For the families of your children, children-in-law, grandchildren, which family paid the most? //

1-25 [Preload childrens name] []

26 None of the above

[For Payers from childrens family, ask the question]

DB028_W3_3 For the family members of [Preload childrens name], who paid you most? [ ]

1 [Preload name of children] himself/herself []

2 [Preload name of children] his/her spouse []

3 [Preload name of children] his/her children, your grandchildren [] ( )

**PROCEDURE** If DB028= 4, ask DB028_W3_4

DB028_W3_4 For your siblings, spouse and children of your siblings, your spouses siblings, spouse and children of your spouses siblings, who paid you most?

1-15 [Preload name of siblings] []

16-30 [Preload name of siblings of spouse] []

99 None of the above

[For payers from every siblings, siblings of spouse, repeat the question]

DB028_W3_5 For the family members of [Preload name of siblings, siblings of spouse], who paid you most? [/]

1 [Preload name of siblings (of spouse)] himself/herself [( )]

2 [Preload name of siblings (of spouse)] him/her spouse [( )]

3 For the children of [Preload name of siblings (of spouse)], how many chilren of [Preload name of siblings (of spouse)] help you in person [( )] [( )] **(DB028_W3_5_1)**

DB029 Do you use the following auxiliary? (Code all that apply) ( ) 1. Walking stick

2. Travel device
3. Manual wheelchair
4. Electric Wheelchair
5. Catheter, urine collection bag
6. Toilet Series
7. None of the above

DB030 Suppose that in the future, you needed help with basic daily activities like eating or dressing. Do you have relatives or friends (besides your spouse/partner) who would be willing and able to help you over a long period of time? ( )

1. Yes
2. No → Skip to DB035_W3_1 DB035_W3_1

DB031 What is the relationship to you of that person or those persons? (Choose all that apply) / ( )

1. Spouse
2. Father, Mother, Father-in-law, Mother-in-law
3. Children, Children s spouses, Grandson, Granddaughter /
/
4. Sibling, Brother-in-law, Sister-in-law, Sibling of spouse, Children of sibling, Sibling of spouse, Brother-in-law, Sister-in-law of spouse, Children of brother-in-law, Children of sister-in-law
5. Other relative
6. Paid helper (such as nanny) ( )
7. Volunteer or Employee of facility
8. Nursing home
9. Community
10. Other

**PROCEDURE** If DB031 = 2, ask DB031_W3_1

DB031_W3_1 Father, mother, father-in-law, mother-in-law, who will help you in future?

1. Father
2. Mother
3. Father-in-law /
4. Mother-in-law /

**PROCEDURE** If DB031= 3, ask DB031_W3_2

DB031_W3_2 For the children, children-in-law, grandchildren who will help you in future, which childrens family are they from? //

1-25 [Preload childrens name] []

26 None of the above

[For helpers from each childrens family, repeat the question]

DB031_W3_3 For the family members of [Preload childrens name], who will help you in person in future? []

1 [Preload childrens name] himself/herself []

2 [Preload childrens name] his/her spouse []

3 [Preload childrens name] his/her children, your grandchildren, the number of them who will help you in person [] ( ) [] _____ (**DB031_W3_3_1**)

**PROCEDURE** If DB031= 4, ask DB031_W3_4

DB031_W3_4 For the siblings, spouse and children of siblings, spouses siblings, spouse and children of spouses siblings who will help you in future, which childrens family are they from?

1-15 [Preload siblings name] []

16-30 [Preload siblings name of spouse] []

99 None of the above

[For helpers from each siblings family, siblings of spouses family, repeat the question]

DB031_W3_5 For the family members of [Preload name of siblings, siblings of spouse], who will help you in person in future? [/]

1 [Preload name of siblings (of spouse)] himself/herself [( )]

2 [Preload name of siblings (of spouse)] his/her spouse [( )]

3 For the children of [Preload name of siblings (of spouse)] who will help you in person, how many of the children of [Preload name of siblings (of spouse)] will help you [( )] [( )] _____ (**DB031_W3_5_1**)

**PROCEDURE** If DB031= 5, ask DB031_W3_6

DB031_W3_6 The number of other relatives who will help you in person in future, and whats their relationship with you? _____ _____(**DB031_W3_6_1**)

**PROCEDURE** If DB031= 10, ask DB031_W3_7

DB031_W3_7 The number of others who will help you in person in future, whats their relationship with you? _____ _____(**DB031_W3_7_1**)

DB035_W3_1 Did you hire a nurse or hourly worker last month? ( ) [If DB022_W3_1= 6, show IWER: paid helper (such as nanny) provided help for the respondent ( )]

1. Nurse
2. Hourly worker
3. None

[(1) . .
5
. (2)
. (3)]

**PROCEDURE** IfDB035_W3_1= 1, askDB035_W3_2-DB035_W3_3; IfDB035_W3_1= 2, ask DB035_W3_5-DB035_W3_6

DB035_W3_2 How much is the total wage of nurse last month? _____ Yuan

[IWER: The total wage means the amount of money paid to nurse, including the equivalent value of goods paid to nurse]

DB035_W3_3 How many months did this nurse work for you? _____ Month

[IWER: Record one hour if it was less than one hour. If you had more than one nurse, record the working hours of the nurse who worked for the longest time 1 1]

DB035_W3_5 How long did the hourly worker work per week last month? ______ Hour

[IWER: (1) The total working hours of all hourly workers would be recorded one hour if it was less than one hour. (2) If you hired more than one hourly worker, the total working hours means the working time of all hourly workers （1） 1 1 .（2） 1]

DB035_W3_6 How much is the wage per hour of hourly worker last month? ______ Yuan

[IWER: If you hired more than one hourly worker, the wage per hour means the total payments to all hourly workers divided by the total working hours 1 1]

DB036_W3 Do you unable to do some kind of work or cannot do that work for a long time because of disability or health reasons?

1. Yes
2. No
3. Too old for work

PROCEDURE If DB036_W3= 1, skip to DB038_W3_1; If DB036_W3= 2 or 3, ask DB037_W3

DB037_W3 Do you unable to do some kind of housework or cannot do that housework for a long time because of disability or health reasons?

1. Yes → Skip to DB038_W3_2
2. No
3. Too old for work

DB038_W3_1 Do you unable to do work completely because of disability or health reasons?

1. Yes
2. No → Skip to DB039_W3

DB038_W3_2 Do you unable to do housework completely because of disability or health reasons?

1. Yes → Skip DB039_W3 DB039_W3
2. No

DB039_W3 Do you able to work for 8 hours a day? Or just work for a few time? 8

1. Yes
2. No

DB032 How often did the respondent receive assistance in answering this section [IWER: If it is answered by a proxy, please record the respondents reaction]

1. Never
2. A few times
3. Most or all of the time
4. The section was completed by a proxy respondent (the respondent is absent)
   → Skip to DB033 DB033

DB033 What is your relationship to R? [IWER: What is the proxys relationship to R? If unknown, please ask the proxy .]

1. Spouse
2. Mother
3. Father
4. Mother-in-law /
5. Father-in-law
6. Sibling
7. Brother-in-law, sister-in-law /
8. Child
9. Spouse of child
10. Grandchild
11. Other relative
12. Helper or other non-relative

DB034 [IWER: Please record the reason for proxy]

What is the main reason for proxy (the respondent is absent)

1. The respondent has serious physical handicaps 2. The respondent has serious mental handicaps
3. The respondent has rejected this interview.
4. Other ______(**DB034_1**)

DC COGNITION & DEPPRESSION

[IWER: If DB032 = 4, then skip to Section E, health care and insurance. Sections DC must not be answered by proxy respondents DB032 = 4 E . DC]

DC001 Now Im going to ask several simple questions. Some may be easy and some may be hard to answer. Please try to answer as honestly as you can. Are you ready? Please tell me todays date. （Check all that apply） . ()

[IWER: R doesnt have to answer in this order. IfR is an elderly person and marked the date by lunar calendar, that date is correct if it matches with the solar calendar. You can check the accuracy, using the converter]

1. Year is correct
2. Month is correct
3. Day is correct

DC002 Please tell me the day of the week. Is it Monday, Tuesday, Wednesday, Thursday, Friday, Saturday, or Sunday?

1. Day of week is correct
2. Day of week is incorrect

DC003 What is the current season (among Spring, Summer, Fall, or Winter)?

1. Season is correct
2. Season is incorrect

DC004 How would you rate your memory at the present time? Would you say it is excellent, very good, good, fair or poor?

1. Excellent
2. Very good
3. Good
4. Fair
5. Poor

[We are going to read a list consisting of 10 words and we would like you to memorize as many as you can. We deliberately made the list long to make it difficult for anyone to memorize all of the words; most people will only remember a few of them. Please listen carefully as we read the list because we cannot repeat it. When we finish reading the list, we will ask you to recall and tell us as many words as you can remember, and they dont have to be in the

order that you heard them. Is this explanation clear? ... .]

DC008 [CAPI automatically record the current time: hour and minute. CAPI _____ (**DC008_1**) _____(**DC008_2**) （24 ）]

DC006 Please tell me any of the words that you remember now. . [IWER: Answers are displayed only for interviewer. Please do not show the screen to R .]

[Try to remember the words I just read to you. Ill ask you to recall them later]

[The 10 items below refer to how you have felt and behaved during the last week. Choose the appropriate response 10]

DC009 I was bothered by things that dont usually bother me. .

1. Rarely or none of the time (< 1 day) （< 1 ）
2. Some or a little of the time (1 − 2 days) （1 − 2 ）
3. Occasionally or a moderate amount of the time (3 − 4 days) （3 − 4 ）
4. Most or all of the time (5 − 7 days) （5 − 7 ）

DC010 I had trouble keeping my mind on what I was doing. .

1. Rarely or none of the time (< 1 day) （< 1 ）
2. Some or a little of the time (1 − 2 days) （1 − 2 ）
3. Occasionally or a moderate amount of the time (3 − 4 days) （3 − 4 ）
4. Most or all of the time (5 − 7 days) （5 − 7 ）

DC011 I felt depressed. .

1. Rarely or none of the time (< 1 day) （< 1 ）
2. Some or a little of the time (1 − 2 days) （1 − 2 ）
3. Occasionally or a moderate amount of the time (3 − 4 days) （3 − 4 ）
4. Most or all of the time (5 − 7 days) （5 − 7 ）

DC012 I felt everything I did was an effort. .

1. Rarely or none of the time (< 1 day) （< 1 ）
2. Some or a little of the time (1 − 2 days) （1 − 2 ）
3. Occasionally or a moderate amount of the time (3 − 4 days) （3 − 4 ）

4. Most or all of the time (5 – 7 days) （5 – 7 ）

DC013 I felt hopeful about the future. .

1. Rarely or none of the time (< 1 day) （< 1 ）
2. Some or a little of the time (1 – 2 days) （1 – 2 ）
3. Occasionally or a moderate amount of the time (3 – 4 days) （3 – 4 ）
4. Most or all of the time (5 – 7 days) （5 – 7 ）

DC014 I felt fearful. .

1. Rarely or none of the time (< 1 day) （< 1 ）
2. Some or a little of the time (1 – 2 days) （1 – 2 ）
3. Occasionally or a moderate amount of the time (3 – 4 days) （3 – 4 ）
4. Most or all of the time (5 – 7 days) （5 – 7 ）

DC015 My sleep was restless. .

1. Rarely or none of the time (< 1 day) （< 1 ）
2. Some or a little of the time (1 – 2 days) （1 – 2 ）
3. Occasionally or a moderate amount of the time (3 – 4 days) （3 – 4 ）
4. Most or all of the time (5 – 7 days) （5 – 7 ）

DC016 I was happy.

1. Rarely or none of the time (< 1 day) （< 1 ）
2. Some or a little of the time (1 – 2 days) （1 – 2 ）
3. Occasionally or a moderate amount of the time (3 – 4 days) （3 – 4 ）
4. Most or all of the time (5 – 7 days) （5 – 7 ）

DC017 I felt lonely. .

1. Rarely or none of the time (< 1 day) （< 1 ）
2. Some or a little of the time (1 – 2 days) （1 – 2 ）
3. Occasionally or a moderate amount of the time (3 – 4 days) （3 – 4 ）
4. Most or all of the time (5 – 7 days) （5 – 7 ）

DC018 I could not get going. .

1. Rarely or none of the time (< 1 day) （< 1 ）
2. Some or a little of the time (1 – 2 days) （1 – 2 ）
3. Occasionally or a moderate amount of the time (3 – 4 days) （3 – 4 ）
4. Most or all of the time (5 – 7 days) （5 – 7 ）

[IWER: Try to persuade R to answer if R refuses at first. Record the exact number R says .]

DC019 Lets try some subtraction of numbers this time. What does 100 minus 7 equal? .
100 7 ______

DC020 And 7 from that? （DC019） 7 ______

DC021 And 7 from that? （DC020） 7 ______

DC022 And 7 from that? （DC021） 7 ______

DC023 And 7 from that? （DC022） 7 ______

DC024 [IWER: Please indicate whether the respondent used paper and pencil or any other aid when completing the number subtraction]
1. Used aid
2. Did not use aid

DC025 Do you see this picture? Please draw that picture on this paper. . [IWER: Show the picture of two pentagons overlapped]
1. Drew the picture
2. Failed to draw the picture

DC026 [CAPI automatically record the current time: hour and minute. __(**DC026_1**) ___ (**DC026_2**) （24 ）]

DC027 A little while ago, I read you a list of words and you repeated the ones you could remember. Please tell me any of the words that you remember now. . . [IWER: Answers are displayed only for interviewer. Please do not show the screen to R .]

[IWER: Please look at the computer with interviewees and do these tests following the instructions. Interviewees could use pen and paper, but can not use computers or other devices for help .]

[Now we will show you several series of numbers on the computer screen. In each series there will be one number that is missing. The missing number will be indicated by a question mark ?. Please look at the pattern of the numbers. Based on this pattern, tell me what the number that is missing

.]

**PROCEDURE** : DC031_W3_1, DC031_W3_2 and DC031_W3_3 are for all respondents. DC031_W3_1, DC031_W3_2DC031_W3_3

DC031_W3_1 7 8_____ 10

DC031_W3_2 8_____ 12 14

DC031_W3_3 18 10 6_____ 3

**PROCEDURE** : DC032_W3_1, DC032_W3_2 and DC032_W3_3 are for respondents who got 0 (zero) question correct in the first 3 questions (DC031_W3_1-DC031_W3_3) （DC031_W3_1-DC031_W3_3） 0DC032_W3_1, DC032_W3_2 DC032_W3_3

DC032_W3_1 1 2 3______

DC032_W3_2 6 5 4______

DC032_W3_3 12_____ 16 18

**PROCEDURE** : DC033_W3_1, DC033_W3_2 and DC033_W3_3 are for respondents who got 1 (one) question correct in the first 3 questions (DC031_W3_1-DC031_W3_3) （DC031_W3_1-DC031_W3_3） 1DC033_W3_1, DC033_W3_2 DC033_W3_3

DC033_W3_1 5_____ 3 2

DC033_W3_2 4 7 10_____

DC033_W3_3 _____ 4 6 8

**PROCEDURE** : DC034_W3_1, DC034_W3_2 and DC034_W3_3 are for respondents who got 2 (two) question correct in the first 3 questions (DC031_W3_1-DC031_W3_3) （DC031_W3_1-DC031_W3_3） 2DC034_W3_1, DC034_W3_2 DC034_W3_3

DC034_W3_1 1 3 3 5 7 7______

DC034_W3_2 3_____ 8 12 17

DC034_W3_3 17_____,12 8

PROCEDURE :

DC035_W3_1, DC035_W3_2 and DC035_W3_3 are for respondents who got 3 (three) question correct in the first 3 questions (DC031_W3_1-DC031_W3_3) （DC031_W3_1-DC031_W3_3） 3DC035_W3_1, DC035_W3_2 DC035_W3_3

DC035_W3_1 10_____3 1

DC035_W3_2 18 17 15_____8

DC035_W3_3 3 3 4 6 6 7__________

DC042_W3 How satisfied are you with your health?

1. Completely satisfied
2. Very satisfied
3. Somewhat satisfied
4. Not very satisfied
5. Not at all satisfied

PROCEDURE :

If BE001 = 1 or 2, ask DC043_W3

DC043_W3 How satisfied are you with your marriage (relationship with spouse)?

1. Completely satisfied
2. Very satisfied
3. Somewhat satisfied
4. Not very satisfied
5. Not at all satisfied
6. No spouse now

DC044_W3 How satisfied are you with your relationship with children? []

1. Completely satisfied
2. Very satisfied
3. Somewhat satisfied
4. Not very satisfied
5. Not at all satisfied
6. No child now

DC028 Please think about your life-as-a-whole. How satisfied are you with it? Are you completely satisfied, very satisfied, somewhat satisfied, not very satisfied, or not at all satisfied?

1. Completely satisfied
2. Very satisfied
3. Somewhat satisfied
4. Not very satisfied
5. Not at all satisfied

This page intentionally left blank

E HEALTH CARE AND INSURANCE

CARD21 Health facilities

1. General hospital ( )
2. Specialized hospital ( )
3. Chinese medicine hospital
4. Community healthcare center
5. Township hospital
6. Health care post
7. Village clinic/ private clinic /
8. Other

CARD20 : Health insurance

1. Urban employee medical insurance (yi-bao) ( ) 2. Urban resident medical insurance
3. New cooperative medical insurance (he-zuo-yi-liao) ( )
4. Urban and rural resident medical insurance ( )
5. Government medical insurance (gong-fei)
6. Medical aid
7. Private medical insurance: purchased by work unit : 8. Private medical insurance: purchased by individual : 9. Urban non-employed personss health insurance 10. Other medical insurance (specify) ( )
11. No insurance

CARD23 : Health facilities for inpatient care

1. General Hospital ( )
2. Specialized hospital ( )
3. Chinese Medicine Hospital
4. Community Healthcare Center
5. Township Hospital
6. Health care post
7. Other

PART I MEDICAL INSURANCE

Now we would like to know about health insurance or benefits that you might have. .
[Show Card 14]

EA001 Are you the policyholder/primary beneficiary of any of the types of health insurance listed below? (circle all that apply) ( )

1. Urban employee medical insurance (yi-bao) ( ) 2. Urban resident medical insurance

3. New cooperative medical insurance (he-zuo-yi-liao) ( )

4. Urban and rural resident medical insurance ( )

5. Government medical insurance (gong-fei)

6. Medical aid

7. Private medical insurance: purchased by work unit : 8. Private medical insurance: purchased by individual : 9. Urban non-employed personss health insurance 10. Other medical insurance (specify) ____ (**EA001_1**)

11. No insurance Skip to EA009 EA009

[Soft check: If pick 11, cannot pick any other, you chose no insurance and a specific type of insurance, this is not possible 11]

F1: (1) .

(2) 2007 7 79 .

() .

(3) . . (4) .

(5) .

(6) . .

(7) .

(8) . .

(9) .

(10) . , , .

(11) .

. .
（12）, 16 60 16 50 , . 700 600 100 .

For each circled type of insurance (1-10), ask the following questions EA002 - EA008. 1-10 EA002 - EA008.

**PROCEDURE** IF EA001 = 7 or 8, skip EA002

EA002 Do you have supplemental insurance to this plan? （ ）

1. Yes
2. No

F1 . .

EA003 Where did you set up your insurance account/policy? ? 1. This county /

2. (if it is not in this county) the place of your hukou （/）
3. Other _______(**EA003_1**)province _________(**EA003_2**)county /

EA005 Through which agency did you purchase your primary plan?

1. Community committee/ village committee /
2. Work unit (incl. village collective)
3. Agency of Social insurance
4. Private insurance company
5. Other

EA006 Whats your out-of-pocket yearly premium? ( )

1. 1 _____Yuan (**EA006_1**) [soft check upper limit: 15,000 for choice 1, 3,000 choice 2, 1,000 choice 3, 1,500 choice 4, 4,000 choice 7, 2,500 choice 9 115,000, 23,000, 31,000, 41,500, 74,000, 92,500]
2. 2

EA007 Who pays the premium for you? (choose all that apply) () 1. Myself

2. Children
3. Relatives
4. Government
5. Work unit (incl. village collective) ( )
6. Loan
7. Donate
8. Others Specify (**EA007_1**)

EA008 When did this benefit begin? [IWER: Mark the year using four digits. Take down the month as its actual number. For example, write January as 1 not 01, December as 12. If do not remember month, fill 0. : 4 . 1 10112 12. 0.]

1900...2015Year (**EA008_1**)____ 0...12Month (**EA008_2**)_____

EA009 What is your main reason for not having health insurance?

1. I do not need it
2. Cannot afford it
3. Do not know where or from whom to get it
4. Do not trust the institutions that offer health insurance 5. Do not have suitable programs for me to buy
6. Do not know/never thought of it
7. Others (**EA009_1**)

**PROCEDURE** Skip to EC001 for new R EC001

**PROCEDURE** For XRType = REIW who had health insurance in last wave

EA001_W3_1 Our records from your last interview show that you have had [preload health insurance], is this right? [] []

1. Yes
2. No

EA001_W3_2 Are you still the policyholder/primary beneficiary of any of the types of health insurance [preload health insurance]? [[]

1. Yes
2. No

EA001_W3_3 Between the last interview and today, were you the policy holder/primary beneficiary of any of the types of health insurance [preload health insurance]? [[]

1. Yes
2. No

F1 (1) .
(2) 2007 7 79 .
() .
(3) . . (4) .
(5) .
(6) . .
(7) .
(8) . .
(9) .
(10) . , , .
(11) . . .
(12) , 16 60 16 50 , . 700 600 100 .

**PROCEDURE** For each circled type of insurance (1-10), ask EA008_W2_1 and EB003 1-10 EA008_W2_1 and EB003

EA008_W2_1 When did you start participating in this insurance? [IWER: Mark the year using four digits. Take down the month as its actual number. For example, write January as 1 not 01, December as 12. If do not remember month, fill 0. : 4 . 1 1 01,12 12. 0.]

1900: : :2013 Year (**EA008_W2_1_1**)____0: : :12 Month (**EA008_W2_1_2**)_____

EB003 When did you stop participating in this insurance?

[IWER: Mark the year using four digits. Take down the month as its actual number.

For example, write January as 1 not 01, December as 12. If do not remember month, fill 0. : 4 . 1 1 01,12 12. 0.]

1900: : :2013 Year (**EB003_1**)____0: : :12Month (**EB003_2**)_____

EB004 Why did you stop participating in this insurance? 1. Employer no longer exists.

2. Insurance not provided anymore locally.

3. I resigned/was fired. /

4. Other (please specify_____ ____(**EB004_1**)

PART II HEALTH CARE COSTS AND UTILIZATION

[IWER: Please do not allow proxy to answer Part II.]

EC001 **PROCEDURE** For XRType = NEWIW

When did you take the last physical examination? **PROCEDURE** For XRType = REIW

When did you take the last physical examination since Rs LAST IW MONTH, YEAR/in the last two years?

[IWER: Mark the year using four digits. Take down the month as its actual number. For example, write January as 1 not 01, December as 12. If do not remember month, fill 0. : 4 . 1 1 01,12 12. 0.]

1. 1900…2013Year (**EC001_1**)____0…12 Month (**EC001_2**)____2. Didnt ever take physical examination yet

3. Didnt take physical examination last two years

EC001_W3_1 Which item do you take in this physical examination?

1. Physical examination
2. Routine blood test
3. Routine urine test
4. Liver function test
5. Kidney function test
6. Lipids profile test
7. Blood glucose test
8. Surgical

9. Internal medicine
10. Five sense organ test
11. Electrocardiogram
12. B-type ultrasonic B
13. Chest fluoroscopy
14. Male or female specialist
15. Other ____, please specify ____(**EC001_W3_2**)

EC002 Who paid for the physical examination?

1. Myself
2. Children
3. Relatives
4. Government
5. Work unit (incl. village collective) ( )
6. Insurance
7. Loan
8. Donation
9. Other ____, please specify ____(**EC002_1**)

EC003_W3 Did you get public health service voucher last year?

1. Yes ____Yuan (**EC004_W3**)RMB [softcheck upper limit: 100000 100000]
2. No

The next questions pertain to medical facilities or medical providers you may have visited for outpatient care during the past 1 month (excluding hospitalization). ( ) .

ED001 In the last month have you visited a public hospital, private hospital, public health center, clinic, or health workers or doctors practice, or been visited by a health worker or doctor for outpatient care? ()

1. Yes Skip to ED004ED004
2. No

ED002 Have you been ill in the last month?

1. Yes
2. No

PROCEDURE

If ED001 = 2 and ED002 = 2 skip to EE001 ED001 = 2 ED002 = 2 EE001.

If ED001 = 1 skip to ED004 ED001 = 1ED004.

If ED002 = 1 and ED001 = 2 go to ED003. ED002 = 1 ED001 = 2 ED003.

ED003 Whats the main reason for not seeking medical treatment?

1. Already under treatment
2. Illness is not serious, dont need treatment 3. No money
4. No time
5. Inconvenient traffic
6. Poor service in hospital
7. Treatment is not useful
8. Other

[Show Card 15]

ED004 Which types of medical facilities have you visited in the last 4 weeks for outpatient treatment?（circle all that apply） ()

1. General hospital ()
2. Specialized hospital ()
3. Chinese medicine hospital
4. Community healthcare center
5. Township hospital
6. Health care post
7. Village clinic/ Private clinic /
8. Other

**PROCEDURE** For each item 1-7 checked in ED004, ask ED005ED004 1-7 ED005

ED005 How many times did you visit/been visited by [...] during the last month? ____ Times

**PROCEDURE** If sum(ED005)> 1, then ask ED006; otherwise, skip ED006. ED005 > 1, ED006, ED006.

ED006 How much did all the visits to [ED004 answer] cost during the last month? [ED004 answer] ? [IWER: If possible, please check the list of cost.]

1. Total cost ____(ED006_ 1) Yuan ; [soft check upper bound: 30,000 30,000. Brackets50/100/200/500/1000]
2. Didnt pay anything

**PROCEDURE** IF ED006 = 1, ASK ED007

ED007 1. Self-paid part ____ (ED007_ 1) Yuan [Brackets50/100/200/500/1000] 2. Didnt pay anything.

Now Id like to ask you some questions about your most recent visit to a health care provider in the last month. .

ED008 Which health care provider did you visit most recently during the past month?
[CAPIPreload the health care providers in ED004. ED004.]

ED009 Is this facility public or private?

1. Public
2. Private

**PROCEDURE** If ED008 = 1 – 3, ask ED010 ED008 = 1 – 3 ED010

ED010 Whats the level of this facility?

1. County/district //
2. Regional/city /
3. Provincial/affiliated to a ministry /
4. Military
5. Others ____ Specify ____(**ED011**)
6. Not applicable

ED012 Did the provider visit you at home?
1. Yes skip ED013 to ED016 and ED022 ED013 ED016 ED022 2. No

ED014 What is the travel time (one-way) to that facility? Minutes ____ (**ED014_1**)
How to go to the facility? (**ED014_2**)

1. Walk
2. Bus
3. Car
4. Ambulance
5. Bicycle or other manual vehicles
6. Electric bicycle/electric tricycle /
7. Motorcycle
8. Tractor
9. Train
10. Animal or animal-pulled cart

**PROCEDURE** If ED014 = 1, skip ED015 ED014 = 1 ED015

ED015 What was the total transportation cost to the facility (including fuel cost, one way trip)? ( ) ____ RMB [softcheck upper limit: 600 600]

ED017 What was the purpose of your visit? (circle all that apply) ()

1. Immunization
2. Consultation
3. Medical check-up
4. Treatment of illness
5. Other

**PROCEDURE** IfED017 = 4, then ask ED018 —ED021, ELSE ASK ED023. ED017 4ED018 — ED021 ED023.

ED018 Could you tell me the disease name? ____

ED019 Was the visit a first visit or a follow-up visit for the symptom?

1. First
2. Follow-up

ED020 Was the visit for ordinary outpatient service or an emergency?

1. Ordinary
2. Emergency

[Show Card 16]

ED021 What kind of treatment did you receive? (circle all that apply) ()

1. Injection
2. Laboratorium test
3. Surgery
4. X-ray, CT, B ultrasonic, MRI X- CTB
5. Medication or retrieve medicine
6. IV (Drip Infusion)
7. Traditional treatment, e.g. massage, accupuncture 8. Other

ED023 What was the total cost of this visit, including both treatment and medication cost (includes prescriptions you received)? ( )

1. _____ RMB (**ED023_1**) [softcheck upper limit: 30,000]
   [Brackets 25/50/120/400/1200]
2. There was no cost

**PROCEDURE** IF ED023 = 1, ASK ED024

ED024 How much did you pay out of pocket, after reimbursement from insurance?

1. ____RMB (**ED024_1**) [soft check upper limit: 30,000, also ED025 <= ED023_1, else Pay out of pocket cannot be more than total cost 30,000ED025 <= ED023_1]
2. Did not pay anything Go to ED026 ED026

[Brackets 15/10/100/300/1000]

ED025 Who contribute most for paying the out-of-pocket cost? 1. Myself

2. Children
3. Relatives
4. Government
5. Work unit (incl. village collective) ( )
6. Loan
7. Donation
8. Other ____(**ED025_1**)

ED026 What was the total medication cost for this visit, including prescriptions you received? ()

1. ____ RMB (**ED026_1**) [soft check upper limit: 5,000, and must be no more than ED023_1. 5,000ED023_1] [Brackets 10/30/80/250/600]
2. Doctor did not write a prescription skip ED027 ED027 3. Didnt fill prescription skip ED027 ED027

ED027 How much will you eventually pay out of pocket for the medications from this visit, including prescriptions you received? ( )

1. ____RMB (**ED027_1**) [softcheck upper limit: 5,000 and ED027_1 must be no more than ED026_1 and no more than ED023_1. 5,000 ED027_1 ED026_1ED023_1] [Brackets 10/20/70/200/500]
2. Didnt pay anything

[Show Card 14]

ED028 What insurance did you use or will you use? (circle all that apply) ( ) ( )

1. Urban employee medical insurance (yi-bao) ( ) 2. Urban resident medical insurance
3. New cooperative medical insurance (he-zuo-yi-liao) ( )

4. Urban and rural resident medical insurance （ ）

5. Government medical insurance (gong-fei)

6. Medical aid

7. Private medical insurance: purchased by work unit : 8. Private medical insurance: purchased by individual : 9. Urban non-employed personss health insurance 10. Other medical insurance (specify) , ____(**ED028_1**)

11. Reimbursed by Work unit (incl. village collective)

12. No insurance

13. Not applicable

F1（1）.

（2） 2007 7 79 .

（）.

（3） . . （4） .

（5） .

（6）. .

（7） .

（8）. .

（9）.

（10） . , , .

（11） . . .

（12） .

（13）, 16 60 16 50 , . 700 600 100 .

ED029 Did you give any red envelopesto the doctors for this visit?

1. Yes
2. No

The following questions pertain to hospitalization (inpatient care) that you have had during the past year. .

EE001 In the past year, did a doctor suggest that you needed inpatient care but you did not get hospitalized?

1. Yes
2. No Skip to EE003 EE003

EE002 Whats the main reason for not seeking inpatient care? 1. Not enough money

2. Not willing to go to the hospital
3. Felt that hospital was unlikely to cure problem—hospital quality poor
4. Felt that care was unlikely to cure the problem—problem too serious
5. No ward available
6. Other

EE003 Have you received inpatient care in the past year? 1. Yes

2. No Skip to EF001 EF001

EE004 How many times have you received inpatient care during the past year? ____Times

**PROCEDURE** If EE004 = 1, skip to EE007. EE004 = 1EE007.

EE005 What was the medical cost for all the inpatient care you received during the past year? (Only include fees paid to the hospital, including ward fees but excluding wages paid to a hired nurse, transportation costs, and accommodation costs for yourself or family members.) ? (.)

1. Total cost ____(**EE005_1**)Yuan ; [Brackets 1500/3000/7000/15000/30000]

2. Didnt pay anything. . [softcheck upper limit : 300,000]

**PROCEDURE** IF EE005 = 1 ASK EE006.

EE006

1. Self-paid part ____(**EE006_1**)Yuan
2. Didnt pay anything.

[softcheck upper limit: 300,000 and EE005 B.1<=EE005A.1. 300,000EE005 B.1<=EE005 A.1.] [Brackets 600/1500/4000/8000/18000]

We want details about the last hospitalization you had in the past year. .

**PROCEDURE** If ED001 = 1 ask EE007. ED001 = 1 EE007.

EE007 Is this the same facility as mentioned in ED008 for outpatient care? [ED008]

1. Yes Skip to EE016 EE016
2. No

**PROCEDURE** If EE007 = 2 or ED001 = 2 ask EE008 -EE015. EE007 = 2 ED001 = 2 EE008-EE015.

[Show Card 17]

EE008 What is the type of health service facility which you visited for last inpatient care (hospital admissions)/for your most recent hospitalization in the past year?

1. General Hospital ( )
2. Specialized hospital ( )
3. Chinese Medicine Hospital
4. Community Healthcare Center
5. Township Hospital
6. Health care post
7. Other

EE009 Is this facility public or private?

1. Public
2. Private

**PROCEDURE** If EE008 = 1 − 3, ask EE010, EE008 = 1 − 3EE010.

EE010 Whats the level of this facility?

1. County/district //
2. Regional/city /
3. Provincial/affiliated to a ministry /
4. Military
5. Others

EE012 What is the location of this facility?

1. province (**EE012_ 1**)
   A. This province
   B. Other province, (preload province) ( ) (**EE012_1_1**) [IWER: Choose from the list of provincessee appendix 2]
2. county/city / (**EE012_2_1**)
   A. This county/city /
   B. Other county/city, specify // (**EE012_2_2**)
3. township/district / (**EE012_3_1**)
   A. This township/district /
   B. Other township/district, specify / (**EE012_3_2**) /
4. village/ street / (**EE012__1_1**)
   A. This village/street /
   B. Other village/street, specify / (**EE012_4_2**)

EE013 How many kilometers is it from the medical facility to your residence? ____ Km
[softcheck upper limit : 3000]

EE014 What is the travel time (one-way) to that facility?

Unit (**EE014_2**)
1. _____Minute [check range : 1-59]
2. ____Hour [softcheck upper limit : 20]

How to go to the facility? (**EE014_3**)
1. Walk
2. Bus
3. Car
4. Ambulance
5. Bicycle or other manual vehicles
6. Electric bicycle/ electric tricycle /
7. Motorcycle
8. Tractor
9. Train
10. Animal or animal pulled cart

**PROCEDURE** IF EE014_2 = 2 − 10, ask EE015

EE015 What was the total transportation cost to the facility (including fuel cost, one way trip)? ( ) ____RMB [soft check upper limit : 600]

EE016 How many nights were you hospitalized there? ____Nights
[softcheck upper limit : 40]

EE017 What was the starting date of your hospital stay?

____1900...2015 (**EE017_1**)Year ____0...12 (**EE017_2**)Month ____0...31 (**EE017_3**)Day [IWER: Mark the year using four digits. Take down the month as its actual number. For example, write January as 1 not 01, December as 12. If do not remember month and day, fill 0. : 4 . 1 10112 12. 0.]

EE018 What was your date of exit?

1. ____1900...2015 (**EE018_1**) Year ____0...12 (**EE018_2**) Month ____0...31 (**EE018_3**) Day
2. Still there

[soft check: date of exit should be not before starting date, exit date is before starting date, please ask R again, also exit date should be within 1 year of today .]

EE019 Why were you hospitalized? (Choose one choice) ()

1. Sickness
2. Accident
3. Violence
4. Other

EE020 Could you tell me the name of the disease? ____

[Show Card 18]

EE021 During hospitalization, what kind of treatment did you receive? (circle all that apply) () [IWER: Read one by one. .]

1. Medical check-up/consultation
2. Injection
3. Laboratory test
4. Surgery
5. X-ray, CT, B ultrasonic, MRI X- CTB
6. Medication or medicine pick-up
7. IV (drip infusion)
8. Traditional treatment, e.g., massage, acupuncture
9. Delivery
10. Other

PROCEDURE IF EE018 = 2, please skip EE022. EE018 = 2EE022.

EE022 Under what conditions did you leave the hospital?

1. Fully recovered from illness, received doctors approval Skip EE023 EE023
2. Hadnt recovered from illness, but doctor suggested to leave Skip EE023 EE023
3. Hadnt recovered from illness, requested to leave without doctors suggestion
4. Other reasons skip EE023 EE023

EE023 Why did you want to leave the hospital before you recovered?
1. Cant recover from illness
2. Financial reason
3. No space in the hospital
4. Limited hospital conditions
5. Bad work attitute of medical personnel
6. Other reasons

EE024 What was the total medical cost of hospitalization? (Only include the fees paid to the hospital, excluding the wage of hired nurse, the fare or rent, but including the ward fees.) （ . ）
1. ____RMB (**EE024_1**)[softcheck upper limit : 100,000]
   [Brackets 700/1500/3500/8000/15000]
2. There was no cost

EE025 What was the total cost for hired nurse? ? 1. ____RMB (**EE025_1**)
2. There was no cost

EE026 What was the total cost for transportation, food and accommodation of patient and relatives? ?
1. ____RMB (**EE026_1**)
2. There was no cost

EE027 How much did you or will you eventually pay out of pocket for the total costs of hospitalization? （）
1. ____RMB (**EE027_1**) [softcheck upper limit: 100,000 and EE027_1 must be no more than EE024_1 100,000EE027_1 EE024_1] 2. Didnt pay anything. Skip EE028 EE028

EE028 Who contributes most for paying the out-of-pocket cost? 1. Myself
2. Children

3. Relatives
4. Government
5. Work unit (incl. village collective) ( )
6. Loan
7. Donate
8. Others Specify (**EE028_1**)

EE029 What was the total medication cost during this visit? 1. ____RMB (**EE029_1**)
[softcheck upper limit: 60,000 and EE029_1 should be
no more than EE024_1 60,000EE029_1 EE024_1.] 2. Didnt receive Skip EE030
EE030
[Brackets 200/500/1800/4000/8000]

EE030 How much did you pay out of pocket for medication costs during this visit?
1. ____RMB (**EE030_1**) [softcheck upper limit: 60,000 and EE030_1 should
be no more than EE029_1 and EE024_1 60,000EE030_1 EE029_1 EE024_1]
[Brackets 100/300/1000/2500/5000]
2. Didnt pay anything.

[Show Card 14]

EE031 (Preload from EA001 or EB002) What insurance did you use or will you use? (circle all that apply) ( )

1. Urban employee medical insurance (yi-bao) ( ) 2. Urban resident medical insurance
3. New cooperative medical insurance (he-zuo-yi-liao) ( )
4. Urban and rural resident medical insurance ( )
5. Government medical insurance (gong-fei)
6. Medical aid
7. Private medical insurance: purchased by work unit : 8. Private medical insurance: purchased by individual : 9. Urban non-employed personss health insurance 10. Other medical insurance (specify) , ____
11. Reimbursed by work unit (incl. village collective)
12. No insurance
13. Not applicable

F1 (1) .
(2) 2007 7 79 .

（）.
（3） . . （4） .
（5） .
（6）. .
（7） .
（8）. .
（9）.
（10） . , , .
（11） . . .
（12） .
（13）, 16 60 16 50 , . 700 600 100 .

EE032 Did you pay any Red Envelopes to the doctors for this visit?
1. Yes
2. No

Now wed like to know whether you have treated yourself during the past month. .
[Show Card 19]

EF001 Did you use any of the following self-treatment methods during the past month?
(circle all that apply) （ ）（） [check, if choose 7 cannot choose other options 7 .]
1. Purchased over-the-counter modern medicines

2. Purchased prescription medicine

3. Traditional herbs or traditional medicine 4. Vitamins/health supplements //

5. Health care equipment

6. Other

7. None Skip to EH001 EH001

**PROCEDURE** For each circled self-treatment method (1-6), ask questions EF002, EF003, EF005. 1-6 EF002EF003EF005.

EF002 What is the approximate total cost for [preload EF001] during the last month? [preload EF001]

1. _____RMB (**EF002_1**) [softcheck upper limits: 2,000 2,000] [Brackets 10/30/100/200/300]

2. There was no cost. .

**PROCEDURE** IF EF002 = 1 ASK EF003. EF002 = 1EF003.

EF003 How much did you pay out-of-pocket?

1. ____RMB (**EF003_1**) [softchecks upper limits: 2,000 and EF003_1 should be no more than EF002_1. 2,000EF003_1 EF002_1] [Brackets 10/30/100/200/300]

2. Didnt pay anything. Go to EF005 EF005.

EF004 Who contributed most for paying the out-of-pocket cost? 1. Myself

2. Children

3. Relatives

4. Government

5. Work unit (incl. village collective) ( )

6. Loan

7. Donation

8. Other Specify (**EF004_1**)

[Show Card 14]

EF005 What insurance did you use? (circle all that apply) ( )

1. Urban employee medical insurance (yi-bao) ( ) 2. Urban resident medical insurance

3. New cooperative medical insurance (he-zuo-yi-liao) ( )

4. Urban and rural resident medical insurance ( )

5. Government medical insurance (gong-fei)

6. Medical aid

7. Private medical insurance: purchased by work unit : 8. Private medical insurance: purchased by individual : 9. Urban non-employed personss health insurance 10. Other medical insurance (please specify) ____

11. Reimbursed by work unit

12. No insurance

13. Not revelent to me

F1 (1) .

(2) 2007 7 79 .

() .

(3) . . (4) .

(5) .

(6) . .

(7) .

(8) . .

(9) .

(10) . , , .

(11) . . .

(12) .

(13) , 16 60 16 50 , . 700 600 100 .

The following questions pertain to dental care that you have had during the past year. .

EH001 In the past year, have you seen a dentist for dental care, including dentures?
1. Yes
2. No skip this part

EH002 How many times have you received dental care during the past year? ____Times

EH003 What was the medical cost for all the dental care in the past year? ?
1. Total cost ____Yuan ;
2. Didnt pay anything. . [softcheck upper limit : 30,000]

**PROCEDURE** IF EH003 = 1 ASK EH004

EH004
1. Self-paid part _______(**EH004_1**)Yuan
2. Didnt pay anything

**PROCEDURE** IF EH004 = Didnt pay anything, skip EH005

EH005 Who contribute most for paying the out-of-pocket cost? 1. Myself
2. Children
3. Relatives
4. Government
5. Work unit (incl. village collective) ( )
6. Loan
7. Donate
8. Others Specify (**EH005_1**)

[Show Card 14]

EH006 What insurance did you use? (circle all that apply) ( )
1. Urban employee medical insurance (yi-bao) ( ) 2. Urban resident medical insurance
3. New cooperative medical insurance (he-zuo-yi-liao) ( )
4. Urban and rural resident medical insurance ( )
5. Government medical insurance (gong-fei)
6. Medical aid

7. Private medical insurance: purchased by work unit : 8. Private medical insurance: purchased by individual : 9. Urban non-employed personss health insurance 10. Other medical insurance (specify) , ____(**EH006_1**)

11. Reimbursed by work unit

12. No insurance

13. Not revelent to me.

EH007_W3 Are you satisfied with the quality, cost, and convenience of local health care services?

1. Very satisfied
2. Somewhat satisfied
3. Neutral
4. Somewhat dissatisfied
5. Very dissatisfied

F1（1）.

（2） 2007 7 79 .

（）.

（3） .. （4） .

（5） .

（6）. .

（7） .

（8）. .

（9）.

（10） . , , .

（11） . ..

（12） .

（13）， 16 60 16 50 ，. 700 600 100 .

EF006 How often did the respondent receive assistance in answering section D-Health care and insurance? [IWER: If it is answered by a proxy, the respondents reaction. .]

1. Never
2. A few times
3. Most or all of the time
4. The section was completed by a proxy respondent（the respondent is absent）
   → Skip to EF007 EF007

EF007 What is your relationship to R? [IWER: What is the proxys relationship to R? If unknown, please ask the proxy. . .]

1. Spouse
2. Mother
3. Father
4. Mother-in-law /
5. Father-in-law /
6. Sibling
7. Brother-in-law, sister-in-law /
8. Child
9. Spouse of child
10. Grandchild
11. Other relative
12. Helper or other non-relative

EF008 [IWER: Please record the reason for proxy] What is the main reason for proxy（the respondent is absent）

1. The respondent has serious physical handicaps 2. The respondent has serious mental handicaps,
3. The respondent has rejected this interview
4. Other ____(**EF008_1**)

F WORK, RETIREMENT AND PENSION

Type of Interview R	
XRType = REIW	This is a reinterview R
XRType = NEWIW	This is a new interview R

Rs LAST IW Time (ZIWTime) (ZIWTime)

PRELOAD VARIABLE ACCORDING TO LAST WAVE INTERVIEW: 一

ZF1= 1 Old R was only doing agricultural work in the last IW

ZF1= 2 Old R was only doing non-agricultural work in the last IW

ZF1= 3 Old R was doing both agricultural and non-agricultural work in the last IW

ZF1= 4 Old R was not working in the last IW

ZF5= 1 Old R was doing employed work in the last IW

ZF6= 1 Old R was doing self-employed work in the last IW

ZF7= 1 Old R was doing unpaid family business in the last IW

ZF8 The name of old Rs employer in the last IW

ZF9 The name of old Rs company or business in the last IW

ZF10 The name of company or workplace old R worked without wage in the last IW

ZF13= 1 Old R was working in the last IW

ZF14= 1 Old R had not worked prior to the last IW

ZF14= 2 Old R had worked prior to the last IW

ZF15= 1 Old R had completed retirement procedure (including early retirement) or internal retirement in the last IW. （）

ZF16= 1 Old R had completed receding position procedure in the last IW

ZF18 Old Rs position in employed work in the last IW

ZF22_1= 1 Old Rs last job is not missing, FL001, FL004 and FL005 are not missing
（FL001 FL004 FL005 ）

ZF22_1= 2 Old Rs last job is missing, FL001-FL022_bracket are all missing
（FL001 FL004 FL005 ）

ZF23= 1 Old R had completed internal retirement but not yet regular retirement prior to the last IW.

ZF24= 1 Old R had not completed retirment procedure or receding position procedure prior to the last IW.

ZF25_1= 1 Old R had completed retirment or receding position procedure in the last IW, but the work unit is missing.
/

ZF25_2= 1 Old R had been receding position procedure in the last IW, but the time is missing.

ZF25_3= 1 Old R had been receding position procedure in the last IW, but the wage is missing.

ZF25_4= 1 Old R had completed retirement or early retirement.
[/]

ZF25_5= 1 Old R had completed retirement or early retirement, but the time is missing.
[/]

ZF25_6= 1 Old R had completed retirement or early retirement, but the wage is missing.
[/]

ZF25_8= 1 Old R had completed internal retirement.

ZF25_9= 1 Old R had completed internal retirement, but the time is missing.

ZF25_10= 1 Old R had completed internal retirement, but the wage prior internal retirement is missing.

ZF25_11= 1 Old R had completed internal retirement, but the wage at internal retirement is missing.

ZF26= 1 Old R had competed regular retirment in the last IW

ZF27= 1 Old R had not worked after processing retirement procedure or receding position procedure by the last IW. /

XF1= 1 Old R is working in the current IW

XF1= 2 Old R is not working in the current IW

XF2= 1 R (including new R and old R) did not do faming last year but did non-agricultural

work last week. ( )

FA JOB STATUS

FA001 Did you engage in agricultural work (including farming, forestry, fishing, and husbandry for your own family or others) for more than 10 days in the past year? 10 ( )

1. Yes → NEWIW skips to FC001; REIW skips to FA006_w2_1 BRANCHPOINT FC001, FA006_w2_1 BRANCHPOINT
2. No

FA002 Did you work for at least one hour last week? We consider any of the following activities to be work: earn a wage, run your own business and unpaid family business work, et. al. Work does not include doing your own housework or doing activities without pay, such as voluntary work. .

1. Yes → NEWIW skips to FC019; REIW skips to FA006_w2_1 BRANCHPOINT FC019; FA006_w2_1 BRANCHPOINT
2. No

FA003 Do you have a job but are temporarily laid-off, or on sick or other leave, or in-job training?

1. Yes
2. No → NEWIW skips to FA007; REIW skips to FA006_w2_1 BRANCHPOINT FA007; FA006_w2_1 BRANCHPOINT

FA004 In what month and year did you leave or attend training?

_____ 1900...2015 (**FA004_1**) year _____ 0...12 (**FA004_2**) month

[IWERMark the year using four digits. Take down the month as its actual number. For example, write January as 1 not 01, December as 12. If do not remember month, fill 0 : 4 . 1 1 01,12 12. 0]

FA005 Do you expect to go back to this job at a definite time in the future or within 6 months? 6

1. Yes → NEWIW skips to FC019; REIW skips to FA006_w2_1 BRANCHPOINT FC019; FA006_w2_1 BRANCHPOINT

2. No

FA006 Do you still receive any salary from this job? ？

1. Yes → NEWIW skips to FC019; REIW skips to FA006_w2_1 BRANCHPOINT FC019; FA006_w2_1 BRANCHPOINT
2. No → NEWIW skips to FA007; REIW skips to FA006_w2_1 BRANCHPOINT FA007; FA006_w2_1 BRANCHPOINT

FA006_w2_1 BRANCHPOINT:

For REIW doing agricultuarl work (FA001= 1): If he/she was only doing agricultural work in the last IW (ZF1= 1), SKIP TO FA006_w3_1; If he/she was only doing non-agricultural work (ZF1= 2) or not doing any work in the last IW (ZF1= 4), SKIP TO FA006_w2_2; If he/she was doing both agricultural and non-agricultural work in the last IW(ZF1= 3), skip to FA006_w2_2a Branchpoint.

(FA001= 1) (ZF1= 1)FA006_w3_1, (ZF1= 2) (ZF1= 4) FA006_w2_2, (ZF1= 3) FA006_w2_2a Branchpoint

For REIW not doing agricultural work but doing non-agricultural work (FA001= 2 & FA002= 1): If he/she was only doing agricultural work in the last IW (ZF1= 1) or doing both agricultural and non-agricultural work (ZF1 = 3) in the last IW, skip to FA006_w2_1; If he/she was only doing non-agricultural work in the last IW (ZF1= 2), skip to FA006_w2_2a Branchpoint; If he/she was not doing any work in the last IW (ZF1= 4), SKIP TO FA006_w3_1;

(FA001= 2 & FA002= 1) (ZF1= 1) (ZF1 = 3) FA006_w2_1, (ZF1= 2) FA006_w2_2a Branch- point, (ZF1= 4) FA006_w3_1

For REIW not doing any work(XF1= 2): If he/she was only doing agricultural work in the last IW (ZF1= 1), or doing both agricultural and non-agricultural work in the last IW (ZF1= 3), skip to FA006_w2_1; If he/she was only doing non-agricultural work in the last IW (ZF1= 2), skip to FA006_w2_2a Branchpoint; If he/she was not doing any work in the last IW (ZF1= 4), skip to FA006_w3_1.

(XF1= 2) (ZF1= 1) (ZF1 = 3) FA006_w2_1, (ZF1= 2) FA006_w2_2a Branchpoint, (ZF1= 4) FA006_w3_1

FA006_w2_1 You told us that you were doing agricultural work (ZF1= 1 or ZF1= 3) in the last IW, when did you stop doing it? [ZIWTime] （ ）（ZF1= 1 ZF1= 3）

1. _____ (**FA006_w2_1_1**) year _____ (**FA006_w2_1_2**) month

2. I was not doing agricultural work in the last IW → Skip to FA006_w2_1a Branchpoint FA006_w2_1a Branchpoint

FA006_w2_1a What was the reason for you to stop doing this job?

1. Business closed /
2. Quit
3. I was laid off
4. I was fired
5. I went to school
6. I went abroad
7. I stopped working for health reasons
8. I stopped working for family reasons
9. I was transferred to another job
10. I started working off-farm locally
11. I went to work away from home
12. Better job in local area
13. Better job in another location
14. I retired
15. Other

FA006_w2_1a BRANCHPOINT:

IF XRType = REIW AND HE/SHE was only doing agricultural work (ZF1= 1) IN THE LAST WAVE, THEN SKIP TO FA006_w3_3 BRANCHPOINT.

(ZF1= 1)FA006_w3_3 BRANCHPOINT IF XRType = REIW AND HE/SHE was doing both agricultural and non-agricultural work (ZF1= 3) IN THE LAST WAVE, THEN SKIP TO FA006_w2_2a Branchpoint.

(ZF1= 3) FA006_w2_2a Branchpoint

FA006_w2_2 You told us that you were not doing agricultural work in the last IW (ZF1= 2 or ZF1= 4), when did you starting doing agricultural work? [ZIWTime] （ZF1= 2 ZF1= 4）

1. _____ (**FA006_w2_2_1**) year _____ (**FA006_w2_2_2**) month
2. I was doing agriclutural work in the last IW Skip to FA006_w3_3 FA006_w3_3

PROCEDURE :

If XRType = REIW and he/she was only doing non-agricultural work in the last IW (ZF1= 2), skip to FA006_w2_2a Branchpoint;

(ZF1= 2) FA006_w2_2a Branchpoint If XRType = REIW and he/she was not doing any work in the last IW (ZF1= 4), skip to FA006_w3_1;

(ZF1= 4) FA006_w3_1

FA006_w2_2a BRANCHPOINT:

For old R who was doing employed work in the last IW (ZF5= 1): If the name of the employer is not missing in the last IW (ZF8≠ :), skip to FA006_w2_2a; If the name of the employer is missing in the last IW (ZF8= :), skip to FA006_w2_2a_2.

(ZF5= 1) (ZF8 ≠ :)

FA006_w2_2a, (ZF8= :) FA006_w2_2a_2

For old R who was doing self-employed work in the last IW (ZF6= 1): If the name of the company/business is not missing in the last IW (ZF9≠ :), skip to FA006_w2_2b; If the name of the company/business is missing in the last IW (ZF9= :), skip to FA006_w2_2b_2.

(ZF6= 1)/ (ZF9≠ :) FA006_w2_2b, / (ZF9= :) FA006_w2_2b_2 For old R who was working for unpaid family business (ZF7= 1): If the name of the family business is not missing in the last IW (ZF10≠ :), skip to FA006_w2_2c. If the name of the family business is missing in the last IW (ZF10= :),skip to FA006_w2_2c_2. (ZF7= 1) (ZF10≠ :) FA006_w2_2c, (ZF10= :) FA006_w2_2c_2

FA006_w2_2a You told us that you were working for [Preload the name of the employer from the last IW(ZF8)] in the last IW, is the name correct? [ZIWTime] [(ZF8)]

1. Yes → Skip to FA006_w2_2a_3 FA006_w2_2a_3
2. No

[( ) (2)]

FA006_w2_2a_1 What is the correct name?

[IWER: Write the name of the household head if R works for a family]

1. Fill in the employers name ______ (**FA006_w2_2a_1_1**) (1 , 2) Name of the department ______ (1, 2) → Skip to FA006_w2_2a_3 FA006_w2_2a_3
2. Last IW, you ran your own business, fill in the firms name // ______ (**FA006_w2_2a_1_2**) (1 , 2) → Skip to FA006_w3_1 FA006_w3_1
3. Last IW, you were not employed and did work for family business without being paid, fill in the firms name / ______ (**FA006_w2_2a_1_3**) (1 , 2) → Skip to FA006_w3_1 FA006_w3_1
4. Last IW, you were not employed and did farm work → Skip to FA006_w3_1 FA006_w3_1

5. Last IW, you did not work → Skip to FA006_w3_1 FA006_w3_1

FA006_w2_2a_2 You told us that you were working for someone else in the last IW, but you did not tell us the name of the employer in the last IW, what is the name of the employer you were working for in the last IW? [ZIWTime]

[IWER: Write the name of the household head if R works for a family]

1. Fill in the employers name ______ (**FA006_w2_2a_2_1**) （ 1 ， 2 ） Name of the department ______ （ 1， 2 ）
2. Last IW, you ran your own business, fill in the firms name / / ______ (**FA006_w2_2a_2_2**) （ 1 ， 2 ） → Skip to FA006_w3_1
3. Last IW, you were not employed and did work for family business without being paid, fill in the firms name / ______ (**FA006_w2_2a_2_3**) （ 1 ， 2 ） → Skip to FA006_w3_1 FA006_w3_1
4. Last IW, you were not employed and did farm work → Skip to FA006_w3_1 FA006_w3_1
5. Last IW, you did not work → Skip to FA006_w3_1 FA006_w3_1

FA006_w2_2a_3 Are you still working for this employer?

1. Yes → Skip to FA006_w3_1 FA006_w3_1
2. No → Skip to FA006_w2_3 FA006_w2_3

FA006_w2_2b You told us that you were running ______ [Preload the name of the company/workplace from the last IW (ZF9), is the name correct? [ZIWTime] / [/（ZF9）] /

1. Yes → Skip to FA006_w2_2b_3 FA006_w2_2b_3
2. No

[(/) (2)]

FA006_w2_2b_1 What is the correct name?

1. Fill in the firms name / ______ (**FA006_w2_2b_1_1**) （ 1， 2） → Skip to FA006_w2_2b_3 FA006_w2_2b_3
2. Last IW, you were employed, fill in the firms name （ ） / ______ (**FA006_w2_2b_1_2**) （ 1 ， 2 ） Name of the department ______ （ 1， 2） → Skip to FA006_w3_1 FA006_w3_1

3. Last IW, you were not self-employed and did work for family business without being paid, fill in the firms name / _____ (**FA006_w2_2b_1_3**) （ 1 ， 2 ） → Skip to FA006_w3_1 FA006_w3_1
4. Last IW, you were not self-employed and did farm work → Skip to FA006_w3_1 FA006_w3_1
5. Last IW, you did not work → Skip to FA006_w3_1 FA006_w3_1

FA006_w2_2b_2 You told us that you were running a company/business in the last IW, but you did not tell us the name of the company/business in the last IW, what is the name of the company/business you were running in the last IW? [ZIWTime] / / /

1. Fill in the firms name / _____ (**FA006_w2_2b_2_1**) （ 1， 2 ）
2. Last IW, you were employed, fill in the firms name （ ） / _____ (**FA006_w2_2b_2_2**) （ 1 ， 2 ） Name of the department _____ （ 1， 2） → Skip to FA006_w3_1 FA006_w3_1
3. Last IW, you were not self-employed and did work for family business without being paid, fill in the firms name / _____ (**FA006_w2_2b_2_3**) （ 1 ， 2 ） → Skip to FA006_w3_1 FA006_w3_1
4. Last IW, you were not self-employed and did farm work → Skip to FA006_w3_1 FA006_w3_1
5. Last IW, you did not work → Skip to FA006_w3_1 FA006_w3_1

FA006_w2_2b_3 Are you still running this company/ business? /

1. Yes → Skip to FA006_w3_1 FA006_w3_1
2. No → Skip to FA006_w2_3 FA006_w2_3

FA006_w2_2c You told us that you were working for an unpaid family business [Preload the name of the unpaid family business from the last IW (ZF10)], is the name correct [ZIWTime] [（ZF10）]

1. Yes → Skip to FA006_w2_2c_3 FA006_w2_2c_3
2. No

[(2)]

FA006_w2_2c_1 What is the correct name?

1. Fill in the firms name / ______ (**FA006_w2_2c_1_1**) (1, 2) → Skip to FA006_w2_2c_3 FA006_w2_2c_3
2. Last IW, you were employed, fill in the firms name ( ) / ______ (**FA006_w2_2c_1_2**) (1 , 2) Name of the department ______ (1, 2) → Skip to FA006_w3_1 FA006_w3_1
3. Last IW, you ran your own business, fill in the firms name / / ______ (**FA006_w2_2c_1_3**) (1 , 2) → Skip to FA006_w3_1 FA006_w3_1
4. → Skip to FA006_w3_1 FA006_w3_1
5. → Skip to FA006_w3_1 FA006_w3_1

FA006_w2_2c_2 You told us that you were working for an family business without a wage in the last IW, but you did not tell us the name of the name of the family business, what is the name of this family business you were working for without a wage in the last IW? [ZIWTime]

1. Fill in the firms name / ______ (**FA006_w2_2c_2_1**) (1 , 2)
2. Last IW, you were employed, fill in the firms name ( ) / ______ (**FA006_w2_2c_2_2**) (1 , 2) Name of the department ______ (1, 2) → Skip to FA006_w3_1 FA006_w3_1
3. Last IW, you ran your own business, fill in the firms name / / ______ (**FA006_w2_2c_2_3**) (1 , 2) → Skip to FA006_w3_1 FA006_w3_1
4. → Skip to FA006_w3_1 FA006_w3_1
5. → Skip to FA006_w3_1 FA006_w3_1

FA006_w2_2c_3 Are you still working for this family business?
1. Yes → Skip to FA006_w3_1 FA006_w3_1
2. No → Skip to FA006_w2_3 FA006_w2_3

FA006_w2_3 When did you stop doing this job? ______ (**FA006_w2_3_1**) year ______ (**FA006_w2_3_2**) month

FA006_w2_4 What was the reason for you to stop doing this job?

1. Business closed /
2. Quit
3. I was laid off
4. I was fired
5. I went to school
6. I went abroad
7. I stopped working for health reasons
8. I stopped working for family reasons
9. I was transferred to another job
10. I started working off-farm locally
11. I went to work away from home
12. Better job in local area
13. Better job in another location
14. I retired
15. Other

FA006_w3_1 Did you engage in agricultural work (including farming, forestry, fishing, and husbandry for your own family or others) in the last IW? [ZIWTime] ( )

1. Yes
2. No → Skip to FA006_w3_3 FA006_w3_3

FA006_w3_2 What agricultural work did you engage in the last IW? ()

1. Household agricultural work
2. Farm employed /

**FA006_w3_3 BRANCHPOINT**:
(**ZF1**= 2 or **ZF1**= 3) FA006_w3_3 - FA006_w3_7
(**ZF1**= 1) FA006_w3_3
(**ZF1**= 4) FA006_w3_3

FA006_w3_3 Did you work in the last IW? We consider any of the following activities to be work: earn a wage, run your own business and unpaid family business work, et. al. Work does not include doing your own housework or doing activities without pay, such as voluntary work. [ZIWTime] ( ) ?

1. Yes

2. No → Skip FA006_w3_4— FA006_w3_7 FA006_w3_4— FA006_w3_7

FA006_w3_4 What non-agricultural work did you do in the last IW?

1. Employed
2. Self-employed → Skip FA006_w3_6 FA006_w3_6
3. Unpaid family business → Skip FA006_w3_7FA006_w3_7

FA006_w3_5 The detail information of employed work in the last IW.

1. What is the name of your workplace/employer? Please state specifically the name of your company or institution. ( )
   _____ **(FA006_w3_5_1)** (1, 2)
2. _____ **(FA006_w3_5_2)** (1 , 2)
3. What kind of business or industry do you work in—that is, what does your workplace primarily make or do? _____ **(FA006_w3_5_3)** (1, 2)
4. What sort of work do you do? _____ **(FA006_w3_5_4)** 1 , 2)

[IWER: Type of business .]

PROCEDURE :
Skip FA006_w3_6 — FA006_w3_7 FA006_w3_6 — FA006_w3_7

FA006_w3_6 The detail information of self-employed work in the last IW.

1. What is the name of your company or workplace? ( ) _____ **(FA006_w3_6_1)** (1, 2)
2. What kind of business or industry do you work in—that is, what does your company do or make? _____ **(FA006_w3_6_2)** (1 , 2)

[IWER: If there is more than one company, ask about the main one. Mark 0 if there is no name . 0]

PROCEDURE :
Skip FA006_w3_7 FA006_w3_7

FA006_w3_7 The detail information of unpaid family business in the last IW.

1. What is the name of your company or workplace that you work in without wage? ( ) _____ (**FA006_w3_7_1**) (1, 2)
2. What kind of business or industry did they work in—that is, what did their company do or make? _____ (**FA006_w3_7_2**) (1, 2)
3. What sort of work do you do? _____ (**FA006_w3_7_3**) 1 , 2)

**FA007 BRANCHPOINT**:<br>If REWIW is working now, or was working in the last IW, or have ever worked (ZF13=1 or ZF14=2 or XF1=1), then skip to FC001 BRANCHPOINT FC001 BRANCHPOINT<br>If REWIW had not worked prior to the last IW (ZF14= 1), and is not working now(XF1= 2), then skip to FA007 (ZF14= 1) (XF1= 2)FA007<br>If NEWIW is not working now(XF1= 2), then skip to FA007 (XF1= 2)FA007

FA007 Have you worked for at least three months during your lifetime (work includes agricultural work,earning wage work, self-employed activities, and unpaid family business work, et. al.)? ( )

1. Yes → Skip to FK Section BRANCHPOINT FK Section BRANCHPOINT 2. No

FA008 Work includes all kinds of labour excluding doing your own housework, whether you earn wages or not. Are you sure that you didn t work at least three months during your lifetime? .

1. Yes, never worked before
2. No, ever worked. → Skip to FK Section BRANCHPOINT FK Section BRANCHPOINT

FA009 What is the main reason for you not to work in your lifetime?

1. Disabled (physical or psychological) ( ) 2. Homemaker
3. My family is too rich that I don t need to work 4. Taking care of siblings
5. Other, ______ (**FA009_1**) _____ (**FA009_1**)

PROCEDURE :
Skip to FK Section BRANCHPOINT FK Section BRANCHPOINT

FC AGRICULTURE WORK

FC001 BRANCHPOINT]:
IfR (including new R and old R) did farming last year (FA001= 1), ask FC001 ( ) (FA001= 1) FC001

if R did not do farming last year, but did non-agricultural work last week (XF2= 1), ask FC019 (XF2= 1) FC019

FARM EMPLOYED 工

FC001 Did you work for other famers in wage for at least ten days in the past year (Agricultural work in wages) 10 ( )
1. Yes
2. No

[]

HOUSEHOLD AGRICULTURAL WORK

FC008 Did you work for your own household for at least ten days in the past year 10
1. Yes
2. No → skip to FC014 FC014

FC009 How many months did you work on [cropping (forestry), livestock, and fishing] for your own householdin the past year? [( )] [( )] [( )]____ 0...12 Months

FC010 How many days did you work for your own household per week on average during a normal work month in the past year? [( )] [()] [( )] ______ 0...7 Days

FC011 How many hours did you usually work for your own household per day during a normal work day in the past year? [( )] [()] [( )] ______ 0...24 Hours

[A check similar to the one for FC006. FC011 > 16 is unreasonable]

FC012 Where is your workplace for most time? [preload sam- pling community ID]

1. The same as permanent address
2. Another village/neighborhood in permanent addresss county/city/district //// _____ (**FC012_1**) village/neighborhood / 3. Other _______ (**FC012_2**) province_city_county/city/district ///_______ (**FC012_3**) village/neighborhood ////
4. Abroad

[]

FC012_w3 How satisfied are you with your job?

1. Completely satisfied
2. Very satisfied
3. Some what satisfied
4. Not very satisfied
5. Not at all satisfied

[]

FC013 How many days of work did you miss last year due to health problems? _____ 0...366 Days

[IWER: Mark 0 if you didnt miss any work days 0]

FC014 Besides agricultural work, did you work for at least one hour last week in wage or self-employed work or umpaid family business? ()

1. Yes → skip to FC019 FC019
2. No

FC015 Do you have wage or self-employed work but are temporarily laid-off or are on sick, seasonal, or other leave or in-job training? ( )

1. Yes
2. No → skip to FC018 FC018

FC016 In what month and year did leave or attend training? // _____ 1900...2015 (**FC016_1**) Year _____ 0...12 (**FC016_2**) Month

[IWER: Mark the year using four digits. Take down the month as its actual number. For example, write January as 1 not 01, December as 12. If do not remember month, fill 0 : 4 . 1 1 01,12 12. 0]

[Same check as the one for FA004. (Interview Year − FC016_1 + (Interview month − FC016_2)/12 > 1]

FC017 Do you expect to go back to this job at a definite time in the future or within 6 months? 6

1. Yes → skip to FC019 FC019
2. No

FC018 At what age do you plan to stop working? Stopping work in this context shall refer to having stopped all income-related activities, unpaid family business and having no intention of engaging in anything more serious than small pastime work. ( ) ? ____

1… 120 Years old

[IWER: Please tell me the approximate age. Mark 0 if you plan to keep working until you are physically able . 0]

[]

PROCEDURE :

Skip to FB011 BRANCHPOINTif IWR only have household agricultural work (FC001= 2 and FC008= 1 and FC014= 2) FB011 BRANCHPOINT

Skip to FD001 if IWR only have farm employed work (FC001= 1 and FC008= 2 and FC014= 2) or have both farm employed work and household farm work (FC001= 1 and FC008= 1 and FC014= 2) FD001

FC019 Besides agricultural work, do you currently hold more than one non-agricultural job? ( )

[IWER: Non-agricultural job includes paid jobs, self-employed activities, unpaid family business work, et. al. Activities without pay, such as voluntary work, are not included , 2]

1. Yes → skip to FC020 FC020
2. No → skip to FC021 FC021

FC020 Among all your jobs, which one is your main job? [Main job is defined as the job at which you work the longest hours] Do you earn a wage or do you run your own business or work for unpaid family business? [ ]

1. Employed → skip to FD001 FD001
2. Self-employed → skip to FH001 FH001
3. unpaid family business → skip to FH001 FH001

FC021 How do you describe your non-agricultural job? Do you earn a wage or do you run your own business or work for unpaid family business?

1. Employed → skip to FD001 FD001
2. Self-employed → skip to FH001 FH001
3. unpaid family business → skip to FH001 FH001

[IWER: （） 1]

FD EMPLOYED

FD001 Do you receive wages from your current workplace or receive them from a dispatch/contract company?

1. Place of work
2. Labor dispatch company
3. Individual

[. .]

[CAPI: For dispatched/contract workers (FD001= 2), mention the following for questions FD002 -FD016 (FD001= 2) FD002-FD016] The next few questions pertain to the situation at your current workplace, and not to the company that has dispatched/contracted you out. .]

FD002 Do you work for a government organization, institution, firm, NGO, individual farmer, or resident household? （ ）

1. Government
2. Institutions
3. NGO （）
4. Firm
5. Individual firm
6. Farmer
7. Individual household
8. Other

FD003 BRANCHPOINT:

For REWIW: If REWIW is working for the same employer (FA006_w2_2a_3= 1), skip FD003. （FA006_w2_2a_3= 1）FD003

[INTRO: We will ask your work history later.In order to distinguish the work units, we need the full name and address of your employer]

FD003 What is the name of your workplace/employer? Please state specifically the name of your company or institution. （） ______ **(FD003_1)** （ 1, 2 ）

______ **(FD003_2)** （ 1， 2 ）

[IWER: Write the name of the household head if R works for a family]

FD004 Where is your workplace located? (preload sampling community ID)

1. The same as permanent address
2. Another village/neighborhood in permanent addresss county/city/district
   // ______ (**FD004_1**) //______ (**FD004_2**) village/neighborhood /
3. Other ______ (**FD004_3**) province_city_county/city/district _ _ // _ (**FD004_4**) // ____ (**FD004_5**) village/neighborhood /
4. Abroad

[]

**PROCEDURE**<br>IfR is working for individual farmer or resident household (FD002= 6; 7),skip to FD011<br>（FD002= 6; 7）FD011

FD005 What kind of business or industry do you work in—that is, what does your workplace primarily make or do? ______ （ 1， 2 ）

[IWER: Type of business .]

**PROCEDURE**<br>If government employee (FD002= 1), ask FD006 to FD007, then skip to FD011<br>FD006 to FD007 FD011

FD006 Are you a civil servant?

1. Yes
2. No

FD007 Are you a formal employee of an establishment? 1. Yes

2. No

**PROCEDURE**<br>If institution (FD002 = 2), ask FD009, then skip to FD011. FD009 FD011

FD009 Are you a formal employee of an establishment? 1. Yes
2. No

PROCEDURE
If firm (FD002= 4), ask from FD010, FD010

FD010 What is the ownership type of the business?
1. 100% State owned firm
2. State-controlled firm
3. 100% Collective-owned firm
4. Collective-controlled firm
5. 100% Private firm /
6. Private-controlled firm
7. 100% foreign-owned
8. Joint venture
9. Other joint-ownership
10. Other

FD011 When did you start working for this employer? ____ 1900...2015 (**FD011_1**) Year _____ 0...12 (**FD011_2**) Month

[IWER: Mark the year using four digits. Take down the month as its actual number. For example, write January as 1 not 01, December as 12. If do not remember month, fill 0 : 4 . 1 1 01,12 12. 0]

[Soft Check: Consistency of employment start date. Make Sure that the individual was at least a minimum age when he/she started working for this employer (e.g., prompt to check if under 16). Specifically (FD011_1+FD011_2/12)-(CV009_a+CV009_b/12)<16 prompts a soft check.]

[Replace 16 in the above soft check with FB001. (FD011_1 + FD011_2/12)-(CV009_a + CV009_b/12) < FB001_1 or FD011_1 < FB001_2]

PROCEDURE :
（ZF1 = 4）FD011 FD011_1FD011_2 [ZIWTime]FD011_w2_1FD011_w2_2

FD011_w2_1 You told us that you were not working for someone else in the last IW, but you said you have worked since [(FD011_1) Year(FD011_2) Month]. Is our record wrong? [ZIWTime] [(FD011_1) (FD011_2)]
1. Yes, I worked in last IW → skip to FD012 FD012

2. No

FD011_w2_2 When did you begin working in this unit? _____ 1900...2015 (**FD011_w2_2_1**) Year _____ 0...12 (**FD011_w2_2_2**) Month

[FD011_w2_2（ZIWTime）]

FD012 What sort of work do you do? _____ (**FD012_1**) 1 , 2) _____ (**FD012_2**) (1 , 2) →

[IWER: Ask about the specific work that R does .]

FD013 What is your current position?

1. Clerk/worker
2. Team Leader ( )
3. Section Chief
4. Director of a division
5. Director-General of a bureau and above
6. Village Leader
7. Township Leader
8. Division manager
9. Overall/General manager
10. Other, ______ (**FD013_1**) _____ (**FD013_1**)

FD014 What is your current professional/technical level? / 1. Technician

2. Primary level
3. Intermediate level
4. Advanced level
5. No professional/technical level

[/]

FD015 Are you in a position to supervise others?

1. Yes
2. No → Skip to FD017. FD017

FD016 How many people are there under your supervision? 1. 1~5 people 1~5

2. 6~10 people 6~10
3. 11~15 people 11~15
4. 16~30 people 16~30
5. 31~99 people 31~99

6. More than 100 people 100

[CAPI: For dispatched worker(FD001 = 2), prompt for FD017-FD031 (FD001= 2) FD017-FD030

The next few questions are about your dispatch work unit. .]

PROCEDURE :

Do not ask FD017 if FD013= 3 - 5 (government officials) FD013= 3 - 5 ( ) FD017

FD017 What is your employment type at your current workplace? 1. Regular worker

2. Contract worker

3. Casual/Part-time worker /

FD020 Did you receive a labor contract (or employment contract) in written form from your current workplace(or labor dispatch company)? /

1. Yes

2. No → Skip to FD024. FD024

FD021 What is the agreed period of employment (labor contract period)?

1. Defined period _____ 0...100 (**FD021_1**) Years _____ 0...11 (**FD021_2**) Months

[IWER: If do not remember months, please fill 0 0]

2. Not defined → Skip to FD024. FD024

3. Same as the term of the project

FD022 Has the current employment contract ever been renewed?

1. Yes

2. No → Skip to FD024. FD024

FD023 How many times has the contract been renewed? _____ 1...50 times

FD024 How long do you expect to work at your current workplace?

1. Less than one year 1

2. One to two years 1-2

3. Two to three years 2-3 → Skip FD025. FD025
4. More than three years 3 → Skip FD025. FD025

FD025 Why do you expect so?

1. Because the predefined contract period will expire
2. Because typically the contract expires (although there s no written contract) ( )
3. Because I was hired under the condition that I would resign upon the request of my employer
4. Because the current job/project will be completed /
5. Because the person I am substituting/replacing will return to work
6. Because I can only work during certain seasons
7. Because I plan to find another job that better suits my job aptitude, abilities, and preferences
8. Because I will reach retirement age as set by regulations/practice
9. Because of family care responsibilities, poor health, etc.
10. Other

FD029 Except for national/public holidays, how many days of paid vacation do you have this year at your current workplace? ______ 0...366 Days

[IWER: Mark 0 if there is no paid vacation 0] [Soft Check for reasonable range: If FD029> 30, prompt for verification]

FD030 How many days of work did you miss at this current job in the past year due to health problems? ______ 0...366 Days

[IWER: Mark 0 if you didnt miss work 0]

**PROCEDURE** :<br>If FD030= 0, skip FD031. FD030= 0FD031

FD031 In these days, how many did not deduct wage or bonus? ______ 0...366 Days
[Soft Check for reasonable range: If FD031>FD030, prompt for verification]

FD032_w3 Do employer buy the personal accident insurance for you ?

1. Yes
2. No

PROCEDURE :
If FD032_w3= 2, skip FD033_w3. FD032_w3= 2 FD033_w3

FD033_w3 How much money can you receive if you encounter the catastrophe accident (e.g. death) ? _____ ? _____

FE QUESTIONS ABOUT LABOR SUPPLY

[CAPI: For dispatched worker(FD001= 2), prompt for FE001-FE003 (FD001= 2) FE001-FE003] The next few questions about labor supply are about the situation of your work place, not dispatch work unit. .

FE001 Counting paid vacations and sick leave not deducting wage as work, how many months did you work in the past year? ( ) _____ 0...12 Months

FE002 How many days a week did you work on average in the past year? _____ 0...7 Days

FE003 How many hours did you work per day on average in the past year, excluding meal breaks but including any paid or unpaid overtime? ( ) _____ 0...24 Hours

[Soft Check: Verify if number of hours per day is unreasonable, e.g., FE003 >16]

FF QUESTIONS ABOUT WAGES

[CAPI: For dispatched worker(FD001= 2), prompt for FF001-FG002 (FD001= 2) FF001-FG002] The following questions about salary and benefits refer to what you receive from the dispatch company. .

FF001 How is your wage paid mainly? Is it regularly paid, contract-based, performance-based, or other? If it is regularly paid, please tell me how often you receive your wages. Do you have a yearly contract, monthly, weekly, daily, or hourly? Please select one.

1. Yearly salary
2. Monthly salary → Skip to FF004. FF004
3. Weekly salary → Skip to FF006. FF006
4. Daily salary → Skip to FF008. FF008
5. Hourly salary → Skip to FF010. FF010

6. Contract-based → Skip to FF012. FF012
7. Performance-based → Skip to FF012. FF012
8. Other → Skip to FF012. FF012

FF002 What is the after-tax salary including bonus in the last year? () _____ (**FF002_1**) Yuan _____ (**FF002_2**) Yuan _____ (**FF002_3**)%

[Soft Check: Prompt for clarification if under a low threshold, e.g. under 1200 RMB annual. Specifically, prompt to clarify if FF002< 1200 RMB.]

FF003 [IWER: If R is unwilling to answer or does not remember, ask unfolding bracket questions here] () 10,000 /30,000 /50,000 /100,000 /200,000 yuan

FF003_w2 [IWER: If R is unwilling to answer or does not remember, ask unfolding bracket questions here] 10,000 /30,000 /50,000 /100,000 /200,000 yuan

**PROCEDURE** :
Skip to FF014. FF014

FF004 What is the after-tax salary including bonus in the last month? () _____ (**FF004_1**) Yuan _____ (**FF004_2**) Yuan _____ (**FF004_3**)%

[Soft Check: Prompt for clarification if under a low threshold, e.g. under 100 RMB per month. Specifically, prompt to clarify if FF004 < 100 RMB.]

FF005 [IWER: If R is unwilling to answer or does not remember, ask unfolding bracket questions here] () 500 /1,000 /2,500 /5,000 /10,000 yuan

FF005_w2 [IWER: If R is unwilling to answer or does not remember, ask unfolding bracket questions here] 500 /1,000 /2,500 /5,000 /10,000 yuan

**PROCEDURE** :
Skip to FF014. FF014

FF006 What is the wage including bonus last week? () _____ Yuan

[Soft Check: Prompt for clarification if under a low threshold, e.g. under 25 RMB per week. Specifically, prompt to clarify if FF006 < 25 RMB.]

PROCEDURE :
Skip to FF012. FF012

FF008 What is the usual daily wage? ______ Yuan

[Soft Check: Prompt for clarification if under a low threshold, e.g. under 5 RMB per day. Specifically, prompt to clarify if FF008 < 5 RMB.]

FF009 [IWER: If R is unwilling to answer or does not remember, ask unfolding bracket questions here] 20 /50 /100 /200 /500 yuan

PROCEDURE :
Skip to FF012. FF012

FF010 What is your hourly wage? ______ Yuan

[Soft Check: Prompt for clarification if under a low threshold, e.g. under 1 RMB per hour. Specifically, prompt to clarify if FF010 < 1 RMB.]

FF011 [IWER: If R is unwilling to answer or does not remember, ask unfolding bracket questions here] 10 /30 /50 /100 /200 yuan

PROCEDURE :
Skip to FF012. FF012

FF012 How much on average do you receive last month after taxes (including bonus)? , __ (**FF012_1**) Yuan _____ (**FF012_2**) Yuan _____ (**FF012_3**)%

[Soft Check: Prompt for clarification if under a low threshold, e.g. under 100 RMB per month. Specifically, prompt to clarify if FF012 < 100 RMB.]

FF013 [IWER: If R is unwilling to answer or does not remember, ask unfolding bracket questions here] , 1,000 /3,000 /5,000 /10,000 /20,000 yuan

FF013_w2 [IWER: If R is unwilling to answer or does not remember, ask unfolding bracket questions here] 1,000 /3,000 /5,000 /10,000 /20,000 yuan

FF014 What is the value of all other bonuses (not paid at same time as regular wage) received in the past year? ( ) ( ) _____ Yuan

[Soft Check: Prompt on bonus and monthly earnings if bonus is more than five times monthly net income]

FF015 [IWER: IfR is unwilling to answer or does not remember, ask unfolding bracket questions here] 1,000 /3,000 /5,000 /10,000 /20,000 yuan

FG Fringe Benefits

[Show Card 21]

FG001 The following are fringe benefits which maybe provided by a company. Please answer if the following are provided by your current workplace and whether you benefit from the following. (Check all that apply) . ()

1. Free lunch
2. Free breakfast
3. Free dinner
4. Meal cash subsidy
5. Transportation cash subsidizations
6. Free housing
7. Subsidization of housing
8. Company car
9. Company bus
10. Other subsidies, ______ (**FG001_ 1**) ( ) _____ (**FG001_ 1**) 11. None

**PROCEDURE** :
For each choice of FG001, ask FG002. FG002

FG002 How much is the value of the subsidy per month? ( ) Yuan

**PROCEDURE** :
Skip to FJ001. FJ001

FH NON-FARM SELF-EMPLOYED AND UNPAID FAMILY BUSINESS

FH001 How many months did you work in the past year? ______ 0…12 Months

FH002 How many days did you work per week on average in the past year? ______ 0...7 Days

FH003 How many hours did you work per day on average in the past year, excluding meal breaks but including any paid or unpaid overtime on a normal work month? __ 0…24 hours

[Soft Check: Verify if number of hours per day is unreasonable, e.g., FH003 >16]

FH004 How many days of work did you miss in the past year due to health problems? __
0…366 Days
[IWER: Mark 0 if you didnt miss any work days 0]

[Next are some questions about the main self- employed work]

**PROCEDURE** :<br>IF FC020= 2 or FC021= 2[Self-employed], ask FC020= 2 FC021= 2 :<br>If old R is running the same company or workplace (FA006_w2_2b_3= 1), skip FH005 / FH005

FH005 What is the name of your company or workplace? ( ) _____ (1, 2) [IWER: If there is more than one company, ask about the main one. Mark 0 if there is no name . 0]

FH006 Where is your company or workplace located? /

1. The same as permanent address
2. Another village/neighborhood in permanent addresss county/city/district // _____ (**FH006_1**) //_____ (**FH006_2**) village/neighborhood /
3. Other _____ (**FH006_3**) province_city_county/city/district _ _ // _ (**FH006_4**) // ____ (**FH006_5**) village/neighborhood /
4. Abroad

[IWER:]

FH007 What kind of business or industry do you work in—that is, what does your company do or make? _____ (1 , 2)
[IWER: Type of business .]

**PROCEDURE** :<br>If old R is running the same company or business(FA006_w2_2b_3= 1), skip FH008 / FH008

FH008 When did you start working at the current company or workplace? / _____
1900…2015 (**FH008_1**) Year _____ 0…12 (**FH008_2**) Month
[IWER: Mark the year using four digits. Take down the month as its actual number. For example, write January as 1 not 01, December as 12. If do not remember

month, fill 0 : 4 . 1 1 01, 12 12. 0]

[A check similar to the one for FD011. (FD011_ 1 + FD011_2 /12)- (CV009_a + CV009_b/12) < 16 or (FD011_ 1 + FD011_2 /12)- (CV009_a + CV009_b/12) < FB001_ 1 or FH008_ 1 < FB001_2]

FH009 Do any other household members work in the same self-employed activity?

1. Yes
2. No

[IWER: Income from self-employment ifR is the single operator（FH009= 2）. If other family member involved, income is asked in household section ,]

FH010 Not including spending on fixed capital, what is your best estimate of net income earned from this activity in the last year? Remember to consider the following types of costs: energy, housing or equipment rental, raw materials, transportation, marketing, wages, taxes, and other fees. / . ______ Yuan

[Soft Check: Prompt for verification if net income is less than 1200 RMB / year. Or, prompt if FH010 <1200 RMB]

FH011 [IWER: IfR is unwilling to answer or does not remember, ask unfolding bracket questions here] 5,000 /10,000 /50,000 /100,000 /200,000 yuan

**PROCEDURE** :<br>Skip to FJ001. FJ001

[Next are some questions about your unpaid family business]

**PROCEDURE** :<br>If old R is working for the same company or workplace without wage (FA006_w2_2c_3= 1), skipFH012 /FH012

FH012 What is the name of company or workplace that you work in without wage? _____ （ 1, 2 ）

[IWER:Mark 0 if there is no name 0]

FH013 Where is this company or workplace loacted? /
1. The same as permanent address
2. Another village/neighborhood in permanent addresss county/city/district
// _____ (**FH013_ 1**) //_____ (**FH013_2**) village/neighborhood /
3. Other _____ (**FH013_3**) province_city_county/city/district _ _ // _ (**FH013_4**) // ____ (**FH013_5**) village/neighborhood /
4. Abroad

[]

FH014 What kind of business or industry do you work in—that is, what does this company do or make? _____ （ 1 ， 2 ）

[IWER: Type of business .]

FH015 What sort of work did you do? _____ （ 1, 2 ）

[IWER: Ask the specific work .]

FH016 How many family members, relatives or friends who work without payment are there including yourself? / （ ） _____ People

FH017 When did this company or workplace start operation/ / _____ 1900…2015 (**FH017_ 1**) Year _____ 0…12 (**FH017_2**) Month

FH018 When did you start working at the current company or workplace? / _____ 1900…2015 (**FH018_ 1**) Year _____ 0…12 (**FH018_2**) Month

FH019 Do any other household members work in the same company or workplace? /
1. Yes
2. No

[IWER: Income from self-employment if no other household members work in（FH019= 2）. If other family member involved, income is asked in household section / ,]

FH020 Not including spending on fixed capital, what is your best estimate of net income earned from this activity in the last year? Remember to consider the following types of costs: energy, housing or equipment rental, raw materials, transportation, marketing,

wages, taxes, and other fees. / . ______ Yuan

[Soft Check: Prompt for verification if net income is less than 1200 RMB / year. Or, prompt if FH020 < 1200 RMB]

FH020_bracket [IWER: If R is unwilling to answer or does not remember, ask unfolding bracket questions here]
5,000 /10,000 /50,000 /100,000 /200,000 yuan

FJ SIDE JOB (EMPLOYED OR SELF-EMPLOYED)

If FC019 = 1(more than one job), proceed with the following section. FC019 = 1( )

FJ001 How many jobs do you currently hold, excluding your main job? ______ 1...20

FJ002 How many hours a week do you work on average at your side job(s), not considering your main job? ______ 0...168 Hours per week /

FJ003 What is the average monthly income or wage that you get from side job(s) other than your main job? ______ Yuan per month /

FJ004 [IWER: IfR is unwilling to answer or does not remember, ask unfolding bracket questions here] 500 /1,000 /2,500 /5,000 /10,000 yuan

FK UNEMPLOYMENT AND JOB SEARCH ACTIVITIES

**FK000_w3 BRANCHPOINT**:   FC019 missing FK000_w3

FK000_w3_1 How satisfied are you with your job? 1. Completely satisfied
2. Very satisfied

3. Some what satisfied
4. Not very satisfied
5. Not at all satisfied

FK000_w3_2 At what age do you plan to stop working? Stopping work in this context shall refer to having stopped all income-related activities, unpaid family business and having no intention of engaging in anything more serious than small pastime work. ?___
1… 120 Years old

[IWER: Please tell me the approximate age. Mark 0 if you plan to keep working until you are physically able . 0]

[]

**FK Section BRANCHPOINT**: • If new R is not working but has worked before (FA007= 1 or FA008= 2), or if new R never worked (FA007= 2 and FA008= 1) before , answer FK. FK • If old R is not working (XF1= 2) but worked before (ZF13= 1 or ZF14= 2) or if old R never worked (FA007= 2 and FA008= 1) before, answer FK FK

**FK001 BRANCHPOINT** : Please skip FK001 if R never worked(FA007 = 2 and FA008 = 1). (FA007 = 2 and FA008 = 1)FK001

FK001 Next are some questions about circumstances about your non-employment and job search activities. In what month and year did you last work? . _____ 1900…2015 (**FK001_1**) Year _____ 0…12 (**FK001_2**) Month

[IWER: Mark the year using four digits. Take down the month as its actual number. For example, write January as 1 not 01, December as 12. If do not remember month, fill 0 : 4 . 1 1 01,12 12. 0]

FK002 Did you search for a new job during the last month? 1. Yes
2. No → Skip to **FL Section FL Section**

FK003 At what age do you plan to stop working? Stopping work in this context shall refer to having stopped all income-related activities, unpaid family business and having no intention of engaging in anything more serious than small pastime work.

?_____ 1… 120 Years old

[IWER: Please tell me the approximate age. Mark 0 if you plan to keep working until you are physically able . 0]

[]

FL LAST JOB

FL001 BRANCHPOINT :

For Old R: If old Rs last job is not missing (ZF22_1 = 1), skip FL. FL If old Rs last job is missing (ZF22_1 = 2), please skip to procedure before FL001. FL001

For new R: Please skip to procedure before FL001. FL001

PROCEDURE :

Ask ifFA007 = 1 or FA008 = 2

(FA007 = 2 FA008 = 1) FN002_w3 If FA007 = 2 and FA008 = 1, skip to FN002_w3

[IWERThe next questions are about the last main job you had, which could be farming, earning a wage, running your own business or working for unpaid family business. It does not include doing your own housework or doing activities without pay, such as voluntary work. If you have more than one job, we are interested in the job at which you worked the longest hours. Were interested in your situation near the termination of this job .]

FL001 Did you work for someone else(including work for unpaid family business), were you self-employed, did you farm, or were you otherwise employed? ( )

1. Employed ( )
2. Self-employed ( )
3. Unpaid family business
4. Farming (,)

FL002 In which year and month did you start working at that job? _____ 1900...2015 (**FL002_1**) Year _____ 0...12 (**FL002_2**) Month

[IWER: Mark the year using four digits. Take down the month as its actual number. For example, write January as 1 not 01, December as 12. If do not remember month, fill 0 : 4 . 1 1 01,12 12. 0] [Soft Check: Prompt for Veri- fication/Clarification if the Respondent was Less than 16 at time of starting this job, e.g. Prompt if (FL002_ 1+FL002_2 /12)-(CV009_a+CV009_b/12)<16] [Replace 16 in the above check with FB001 . FL002_ 1 < FB001_2 or FL002_ 1 < (CV009_a + FB001_ 1)]

FL003 In which year and month did you stop working at that job? _____ 1900...2015 (**FL003_ 1**) Year _____ 0...12 (**FL003_2**) Month

[IWER: Mark the year using four digits. Take down the month as its actual number. For example, write January as 1 not 01, December as 12. If do not remember month, fill 0 : 4 .1 101,12 12. 0] [Soft Check: Prompt for Verification/clarification if End Date is Before the Start Date, e.g. FL003_ 1 < FL002_ 1]

FL004 BRANCHPOINT :

For old R, if FL003_1<Last IW Year （ZIWYear） or FL003_1=Last IW Year （ZIWYear） and FL003_2<Last IW Month （ZIWMonth）, then skip to Section FM FL003_1< （ZIWYear） FL003_1= （ZIWYear） FL003_2< （ZIWMonth） FM

FL004 Where was the job located?

1. The same as permanent address
2. Another village/neighborhood in permanent addresss county/city/district // _____ (**FL004_ 1**) //_____ (**FL004_2**) village/neighborhood /
3. Other _____ (**FL004_3**) province_city_county/city/district _ _ // _____ (**FL004_4**) // _____ (**FL004_5**) village/neighborhood / 4. Abroad

[IWER:]

PROCEDURE :

If FL001 = 4 Skip to FL020 FL001 = 4FL020

If non-agricultural work (FL001= 1/2/3), ask FL005 to FL008 . （FL001= 1/2/3）FL005 to FL008

FL005 What was the name of your workplace/employer? Please state specifically the name of your company or business. () _____ (**FL005_1**) （ 1, 2 ） , _____ (**FL005_2**) （ 1, 2）

FL006 What kind of business or industry was it—that is, what did they make or do at the place where you worked? ______ （1，2）
[IWER: Type of business .]

FL007 Is this employer still in existence?
1. Yes
2. No

FL008 How many hours a week did you usually work [for this employer/in this business]?
[______ 0...168 Hours per week /
[Soft Check: Prompt for Verification if FL008 > 80]

**PROCEDURE** :<br>Ask only if employed. （FL001= 1）

FL009 What were the monthly wages, bonuses, and subsidies from this jobbefore you stopped working at this job? （ ） ______ Yuan
[IWER: Mark 0 if there is no net income, and mark 999997 if running a deficit 0 999997] [Soft Check: Soft Check: Prompt for Verification if FL009 < 100]

FL010 [IWER: IfR is unwilling to answer or does not remember, ask unfolding bracket questions here] 500 /1,000 /2,500 /5,000 /10,000 yuan

FL011 What was the value of other bonuses not paid with regular wages each year? （ ） ______ Yuan

FL011_bracket [IWER: If R is unwilling to answer or does not remember, ask unfolding bracket questions here]
500 /1,000 /2,500 /5,000 /10,000 yuan

**PROCEDURE** :<br>Ask FL012 if self-employed (FL001 = 2). （FL001 = 2） FL012 Otherwise skip to FL013. FL013

FL012 Do you have employees?
1. Self-employed with employees
2. Self-employed without employees
3. Family business worker without pay

PROCEDURE :

Ask FL013－FL017 only if employed (FL001 = 1). FL013－FL017 （FL001 = 1）

Otherwise skip to FL020. FL020

FL013 Were you a regular worker, a temporary worker, or a casual worker?

1. Regular wage worker
2. Contract worker
3. Temporary wage worker
4. Casual wage worker

FL014 Did you work for the government, institution, firm, NGO, individual farmer or a resident household? （ ）

1. Government
2. Institutions → Skip to FL017 FL017
3. NGO （） → Skip to FL017 FL017 4. Firm Skip to → FL016 FL016
5. Individual firm → Skip to FL017 FL017
6. Individual farmer → Skip to FL017 FL017
7. Individual household → Skip to FL017 FL017
8. Other, ______ (**FL014_1**) _____ (**FL014_1**) → Skip to FL017 FL017

PROCEDURE :

If government employee (FL014= 1), ask FL015. FL015

FL015 Were you a civil servant?

1. Yes
2. No

PROCEDURE :

If firm (FL014= 4), ask FL016. (FL014= 4)FL016

Otherwise skip to FL017. FL017

FL016 What was the ownership type of the business? 1. 100% State owned firm

2. State-controlled firm
3. 100% Collective-owned firm
4. Collective-controlled firm
5. 100% Private firm /
6. Private-controlled firm

7. 100% foreign-owned
8. Joint venture
9. Other joint-ownership
10. Other, ______ (**FL016_1**) _____ (**FL016_1**)

FL017 What sort of work did you do? _____ (**FL017_1**) (1 , 2) _____ (**FL017_2**) (1 , 2)
[IWER: Ask the specific work .]

FL020 Why did you leave that employer?
[IWER: Do not probe but check all that apply]
1. Business closed / (**FL020s1**)
2. Quit (**FL020s2**)
3. I was laid off (**FL020s3**)
4. I was fired (**FL020s4**)
5. I went to school (**FL020s5**)
6. I went abroad (**FL020s6**)
7. I stopped working for health reasons (**FL020s7**) 8. I stopped working for family reasons (**FL020s8**) 9. I was transferred to another job (**FL020s9**)
10. I was sent down to the countryside to do manual labor (**FL020s10**) 11. I started working off-farm locally (**FL020s11**)
12. I went to work away from home (**FL020s12**)
13. Better job in local area (**FL020s13**)
14. Better job in another location (**FL020s14**)
15. I retired (**FL020s15**)
16. Other, ______ (**FL020_1**) _____ (**FL020_1**) (**FL020s16**)

FL021 Did you receive any payments other than the legal retirement allowance upon leaving your last job? (For example, condolence paymentworkers compensation, etc.) ( )
1. Yes
2. No
[ ]

**PROCEDURE** :<br>If FL021 = 1, ask FL022. FL021= 1 FL022

FL022 How much was the compensation and for how many years of work? ______ Yuan ______ years ? ______ (**FL022_1**) , ______ 0...120 (**FL022_2**)

FL022_bracket [IWER: If R is unwilling to answer or does not remember, ask unfolding bracket questions here]

1000 /2000 /5000 /10,000 /20,000 yuan

FM RETIREMENT

**FB011 BRANCHPOINT**:<br>If old R had completed retirement procedure (including early retirement) or internal retirement (ZF15= 1), skip FB011 and FB012 （ ） （ZF15= 1）FB011 FB012<br>NEWIW: Ask FB011

FB011 Have you completed retirement procedures （including early retirement） or internal retirement（Note: Retirement from government departments, enterprises and institutions, not including retirement in the sense of getting agricultural insurance）? （ ） （ ）

1. Yes
2. No

FB012 Have you completed receding position procedures 1. Yes

2. No

[.]

**PROCEDURE** :<br>FB011 = 2 and FB012 = 2, skip to FN002_w3 FN002_w3

**PROCEDURE** :<br>If R has processed retirement [FB011 = 1 or FB012 = 1 or ZF15 = 1 or ZF16 = 1], ask FM001 －FM059 . [FB011 = 1 ZF15 = 1] [FB012 = 1 ZF16 = 1] / /FM001 －FM059

FM001_1 BRANCHPOINT :

（ ZF16= 1 ） ZF25_1 ZF25_2 ZF25_3 ZF25_1= 1FM004ZF25_2= 1FM005ZF25_3= 1 FM007. FM001_2 BRANCHPOINT

[/] （ ZF25_4= 1 ） ZF25_1 ZF25_5 ZF25_6ZF25_1= 1FM004ZF25_5= 1FM014 ZF25_6= 1FM016-FM017. FM001_2 BRANCHPOINT

（ZF25_8= 1）ZF25_1ZF25_9ZF25_10 ZF25_11ZF25_1= 1FM004ZF25_9= 1FM025ZF25_10= 1 FM027ZF25_11= 1FM028-FM029. FM001_2 BRANCH- POINT

FM001_2 BRANCHPOINT :

If old R had completed receding position procedure prior to the last IW (ZF16= 1), or had completed regular retirement in the last IW (ZF16= 1), skip to FM052 BRANCHPOINT. FM052 BRANCHPOINT

If old R had completed internal retirement but not yet regular retirement prior to the last IW （ZF23= 1）, skip to FM037_w2 BRANCHPOINT. FM037_w2 BRANCHPOINT

If old R had not completed retirment procedure or receding position procedure prior to the last IW (ZF24= 1), continue with FM001 . FM001

All new R continues with FM001 FM001

FM001 Which of the following is the work unit that processed your [preload: retirement /receding position] [/]

[IWER: IfR has no work unit (no answer to FD003 or FL027), please choose (3) None of the above（3）]

1. Current work unit [for new R: preload FD003; for old R: ZF8 if FA006_w2_2a= 1 & FA006_w2_2a_3= 1, or FA006_w2_2a_ 1= 1 if FA006_w2_2a= 2 & FA006_w2_2a_3= 1, or FM003 if FA006_w2_2a_3= 2] → Skip to procedure before FM005 FM005
2. Last work unit [preload FL005 or ZF25] FL005 [FL005 or ZF25] → Skip to procedure before FM005 FM005
3. None of the above

PROCEDURE :

If answer to FM001is (3) None of the above, then ask FM002-FM004 : FM001 (3) FM002-FM004

FM002 What is the name of the employer that processed your [preload: retirement /receding position]? [/] ______

FM003 What was the type of your work unit at [preload: retirement /receding position]?
[/]

1. Government
2. Institutions
3. NGO （）
4. Firm
5. Individual firm
6. Farmer
7. Individual household
8. Other

FM004 Where is this work unit located?

1. The same as permanent address
2. Another village/neighborhood in permanent addresss county/city/district // __ (**FM004_1**) ////
3. Other ______ (**FM004_2**) province_city_county/city/district _ _ // ______ (**FM004_3**) ////
4. Abroad

[]

**PROCEDURE** :
If FB012= 1 (Receding), ask FM005-FM009 FB012= 1 （） FM005- FM009

FM005 In what month and year did you recede from your position? ______ (**FM005_1**) 1900…2015 year ______ (**FM005_2**) 0…12 month

[IWERMark the year using four digits. Take down the month as its actual number. For example, write January as 1 not 01, December as 12. If do not remember month, fill 0 : 4 . 1 1 01,12 12. 0]

FM006 What was the main reason you receded from your position? 1. Due to poor health, I couldnt continue my work any more, at the same time I wasnt eligible for retirement

2. Years of eligible work are less than threeand time of stopping work due to diseases or injures not related to work are more than one year
3. Im recruit worker within 6 months, but I had serious chronic disease once and cant tstick to work any more
4. I receded from my position voluntary
5. Reach retirement age, but not eligible working age. 6. Other

FM007 Your pre-receding total salary was _____ Yuan a month (including basic wage, bonus, et. al). _____ /, .

FM007_bracket [IWER: If R is unwilling to answer or does not remember, ask unfolding bracket questions here]
500 /1,000 /2,000 /3,500 /5,000 yuan

FM008 Did you receive any payments for leaving your job 1. Yes
2. No

**PROCEDURE** :
If FM008 = 1, ask FM009. FM008 = 1 FM009

FM009 How much was the compensation? ________ Yuan ? _____ ,

FM009_bracket [IWER: If R is unwilling to answer or does not remember, ask unfolding bracket questions here]
500 /1,000 /2,000 /3,500 /5,000 yuan

**PROCEDURE** :
If FB011 = 1 (Retirement), askFM011-FM041. FB011= 1 ( )FM011- FM041
Otherwise skip to FM042, FM042

FM011 Was your retirement normal retirement; early retirement; or internal retirement initially, followed by regular retirement? ,

1. Normal retirement
2. Early retirement
3. Internal retirement first, then regular retirement
4. Internal retirement, but not yet regular retirement

FM012 Did you retire as a worker or as a cadre? 1. Worker
2. Cadre

**PROCEDURE** :
If FM011 = 1 or FM011 = 2 , ask FM014-FM017. FM011 = 1/2 FM014-FM017

FM014 In what month and year did you take [preload: normal/early] retirement? [/] __ 1900…2015 (**FM014_1**) year _____ 0…12 (**FM014_2**) month
[IWERMark the year using four digits. Take down the month as its actual number.

For example, write January as 1 not 01, December as 12. If do not remember month, fill 0 : 4 . 1 1 01,12 12. 0]

[Soft Check: Prompt for Verification/Correction if Age of Early Retirement is Young, e.g. Prompt if ((FM014_ 1 +FM014_2 /12)-(CV009_a+CV009_b/12) < 45 & CV004==2) | ((FM014_ 1 +FM014_ 1 /12)-(CV009_a+CV009_b/12) <50 & CV004== 1)]

PROCEDURE :
Ask FM015 if FM011 = 2 (early retirement). FM015

FM015 What was the main reason you processed early retirement?

1. I have 30 years job experience, which is enough for early retirement. 30 .
2. My work unit belonged to the category of high-risk and hard manual labor and thus was eligible for offering early retirement
3. My work unit was restructuring/bankrupt, so it offered early retirement
4. Due to poor health
5. Due to family reason
6. Other

FM016 Your pre-retirement salary was ______ Yuan a month (including bonus and subsidy, et. al). _____ /.

FM017 [IWER: If R is unwilling to answer or does not remember, ask unfolding bracket questions here] 500 /1,000 /2,500 /5,000 /10,000 yuan

PROCEDURE :
Ask FM025-FM029 if FM011 = 3or FM011 = 4 (internal retirement) FM011=3/4 FM025-FM029
Otherwise skip to FM042, FM042

FM025 In what month and year did you take internal retirement? _____ 1900...2015 (**FM025_ 1**) year _____ 0...12 (**FM025_2**) month

[IWERMark the year using four digits. Take down the month as its actual number. For example, write January as 1 not 01, December as 12. If do not remember month, fill 0 : 4 . 1 1 01,12 12. 0]

[Soft Check: Prompt for Verification/Correction if Age of Retirement is Young, e.g.

Prompt if (FM030_ 1 +FM030_2 /12)-(CV009_a+CV009_b/12) <45 & CV004==2) | ((FM030_ 1 + FM030_2 /12)-(CV009_a+CV009_b/12) <50 & CV004== 1)]

FM026 What was the main reason you processed internal retirement?

1. 5 years less than the legal retirement age 5 .
2. My work unit was restructuring/bankrupt
3. Due to poor health
4. Due to family reason
5. Other

FM027 Your pre-internal retirement salary was ______ Yuan a month everything included. ? ______ /.

FM027_bracket [IWER: If R is unwilling to answer or does not remember, ask unfolding bracket questions here]

500 /1,000 /2,000 /3,500 /5,000 yuan

FM028 How much was the internal retirement wage (everything included) when you processed internal retirement? ___ /.

FM029 [IWER: If R is unwilling to answer or does not remember, ask unfolding bracket questions here] 500 /1,000 /2,000 /3,500 /5,000 yuan

**PROCEDURE** :
Ask FM030 - FM036 if FM011 = 3 FM011= 3FM030 - FM036

FM030 In what month and year did you process formal retirement? ______ 1900...2015 (**FM030_1**) year ______ 0...12 (**FM030_2**) month

[IWERMark the year using four digits. Take down the month as its actual number. For example, write January as 1 not 01, December as 12. If do not remember month, fill 0 : 4 . 1 1 01,12 12. 0]

[Soft Check: Prompt for Verification/Correction if Age of Retirement is Young, e.g. Prompt if ((FM030_1 +FM030_2 /12)-(CV009_a+CV009_b/12) <45 & CV004==2) | ((FM030_1 +FM030_2 /12)-(CV009_a+CV009_b/12) <50 & CV004==1)]

FM036 How many years of eligible work did you have at the time of formal retirement? / __ 0.00...100.00 Years

**PROCEDURE** :
Skip to FM042 FM042

FM037_w2 BRANCHPOINT :

If R had completed internal retirement but not yet regular retirement prio to the last IW (ZF23= 1), then ask FM037_w2- FM040_w2. FM037_w2FM040_w2

FM037_w2 Did you proceed formal retirement? 1. Yes

2. No → Skip to FM037 FM037

FM038_w2 In what month and year did you process formal retirement? ______ 1900…2015 (**FM038_w2_1**) year ______ 0…12 (**FM038_w2_2**) month

[IWERMark the year using four digits. Take down the month as its actual number. For example, write January as 1 not 01, December as 12. If do not remember month, fill 0 : 4 . 1 1 01,12 12. 0]

[Soft Check: Prompt for Verification/Correction if Age of Retirement is Young, e.g. Prompt if ((FM030_1 +FM030_2 /12)-(CV009_a+CV009_b/12) <45 & CV004==2) | ((FM030_1 +FM030_2 /12)-(CV009_a+CV009_b/12) <50 & CV004== 1)]

FM040_w2 How many years of eligible work did you have at the time of formal retirement? / 0.00…100.00 Years

PROCEDURE :

Ask FM037 – FM040 if FM011 = 4 or FM037_w2 = 2. FM011 = 4 FM037_w2 = 2FM037 – FM040

Otherwise Skip to FM042. FM042

FM037 In what month and year are you going to process formal retirement? ______ 1900…2015 (**FM037_1**) year ______ 0…12 (**FM030_2**) month

[IWERMark the year using four digits. Take down the month as its actual number. For example, write January as 1 not 01, December as 12. If do not remember month, fill 0 : 4 . 1 1 01,12 12. 0]

[Soft Check: Verify if Age of Respondent will be outside the legal retirement range, e.g., Prompt for verification if ((FM037_1 + FM037_2/12) - (CV009_a + CV009_b/12) < 50 | (FM037_1 + FM037_2/12) - (CV009_a + CV009_b/12) > 55) & CV004 == 2) | (FM037_1 + FM037_2 /12) - (CV009_a + CV009_b/12) < 55 | (FM037_1 + FM037_2/12) - (CV009_a + CV009_b/12) > 60) & CV004 == 1)]

FM040 How many years of eligible work will you have at the time of retirement? / ______ 0.00…100.00 Years

FM041 How many years of eligible work do you currently have? / ______ 0.00…100.00 Years
[FM040 > FM041]

PROCEDURE :
Skip (FM042 – FM051) if FM037_w2 = 2. FM037_w2 = 2FM042 – FM051

FM042 Did you have a spouse when you processed [preload: normal retirement / early retirement / internal retirement / receding position]?
[/// (FM011. FM037_w2 = 1 .)]

1. Yes
2. No

FM043 How was your health at the time of your [preload: normal retirement / early retirement / internal retirement / receding position], excellent, very good, good, fair or poor? [///] ?

1. Excellent
2. Very good
3. Good
4. Fair
5. Poor

PROCEDURE :
Skip FM044 – FM046 if FM042 = 2. FM042 = 2 FM044 – FM046

FM044 Had your spouse already processed retirement when you processed [Preload: normal retirement / early retirement / internal retirement / receding position]? [///]

1. Yes
2. No

FM045 What kind of economic activities was your spouse engaged in at the time of your [preload: retirement / receding position]? [/]

1. Employed by another person or company and received a wage
2. Ran own business
3. Non-employed and looking for a job
4. Non-employed and not looking for a job or only doing household work ,
5. Farming

FM046 How was your spouses health at the time of your [preload: normal retirement / early retirement / internal retirement / receding position], excellent, very good, good, fair or poor? [///] ?

1. Excellent
2. Very good
3. Good
4. Fair
5. Poor

FM047 Was your father alive at the time of your [preload: normal retirement / early retirement / internal retirement / receding position]? [///]

1. Yes
2. No → Skip to FM049 FM049

FM048 How about the health of your father at the time of your [preload: normal retirement / early retirement / internal retirement / receding position], excellent, very good, good, fair or poor? [///] ?

1. Excellent
2. Very good
3. Good
4. Fair
5. Poor

FM049 Did your mother alive when your [preload: normal retirement / early retirement / internal retirement / receding position]? [///]

1. Yes
2. No → Skip to FM051 FM051

FM050 How about the health of your mother at the time of your [preload: normal retirement / early retirement / internal retirement / receding position], excellent, very good, good, fair or poor? [///] ?

1. Excellent
2. Very good
3. Good
4. Fair
5. Poor

FM051 How many grandchildren below age 6 did you have at the time of your [preload: normal retirement / early retirement / internal retirement / receding position]? [///]
0…50 persons
[IWER: If none, fill 0 0]

FM052 BRANCHPOINT:

For old R who had processed receding position procedure in the last IW (ZF16 = 1) or had processed internal but not regular retirement in the last IW (ZF23 = 1), or had processed regular retirement in the last IW (ZF26 = 1): if old R had not worked after processing retirement/receding position procedure until the last IW (ZF26 = 1) and is currently not working (XF1 = 2), ask FM052_w2; if old R had not worked after processing retirement/receding position procedure until the last IW (ZF27 = 1) and is currently working (XF1 = 1), ask FM053 / FM052_w2, / FM053. FM052_w2

New R If R is currently not working (FA001 = 2 & FA002 = 2 & FA003 = 2), ask FM052. （FA001 = 2 & FA002 = 2 & FA003 = 2）FM052 If FA052 = 1 or If R is currently working, ask FM053. （FA052 = 1 or FA001 = 1 or FA002 = 1 or FA003 = 1）FM053

FM052 Did you work after you processed [preload: normal retirement / early retirement / internal retirement / receding position]? We consider any of the following activities to be work: agricultural work, earn a wage, run your own business and unpaid family business work, et. al. Work does not include doing your own housework or doing activities without pay, such as voluntary work. [///] .

1. Yes → Skip to FM053 FM053
2. No → Skip to FM054 FM054

FM052_w2 Did you work in the last two years? We consider any of the following activities to be work: agricultural work, earn a wage, run your own business and unpaid family business work, et.al. Work does not include doing your own householdwork or doing activities without pay, such as voluntary work.

1. Yes
2. No → Skip to FM054 FM054

FM053 After you processed [preload: normal retirement / early retirement / internal retirement / receding position] How long did you start to work again? [///] ______
0.00...100.00 Years (allow for decimal points)

PROCEDURE :

Skip to FN002_w3 FN002_w3

FM054 Are you currently engaged in paid small pastime work?

1. Yes
2. No → Skip to FN002_w3 FN002_w3

FM055 What kind of pastime job are you engaged in? ? _______ [IWER: Ask the specific work .]

FM056 When did you start this job? ______ 1900...2015 (**FM056_ 1**) year _____ 0...12 (**FM056_2**) month

[IWERMark the year using four digits. Take down the month as its actual number. For example, write January as 1 not 01, December as 12. If do not remember month, fill 0: 4 . 1 1 0112 12. 0]

FM057 How many days per week do you usually work for your pastime job? An average of _ days per week _____ 0...7

FM058 How many hours per week do you usually work at your pastime job? An average of _______ hours per week _____ 0.00...168.00

FM059 What is your monthly income from the pastime work? _____ YUAN

[IWER: Mark 0 if there is no net income, and mark 999997 if running a deficit 0 999997]

FM059_bracket [IWER: If R is unwilling to answer or does not remember, ask unfolding bracket questions here]

500 /1,000 /2,000 /3,500 /5,000 yuan

FN PENSION INSURANCE

[Intro: Next we ll ask you some questions about your pension insurance. It s important to assess existing pension policy and revise it in the future :
] [F1 (1)
.
.
. (2)
. (3)
. (4)
. (6)

. (7) . (8) ( ) . (9) ( ) 60 , .]

Part 1

FN002_w3

1. Yes, I receive it
2. Yes, but I dont receive it → Skip to FN006_w2 FN006_w2 3. No → Skip to FN030_w2 FN030_w2

FN002_w2 Are you currently receiving pension as follows (Check all that apply)? / ()

1. ( )
2.
3.

**PROCEDURE** :<br>FN002_w2FN003_w2-FN005_w2_bracket

FN003_w2 In what month and year did you start to receive you pension benefits? () _____ 1900…2015 (**FN003_w2_1**) year _____ 0…12 (**FN003_w2_2**) month

[IWERMark the year using four digits. Take down the month as its actual number. For example, write January as 1 not 01, December as 12. If do not remember month, fill 0 : 4 . 1 1 0112 12. 0]

FN004_w2 How much were the benefits (including subsidy) when you retired? / () _____ Yuan per month /.

FN004_w2_bracket [IWER: IfR is unwilling to answer or does not remember, ask unfolding bracket questions here]

1000 /2,000 /3,000 /5,000 /10,000 yuan

FN005_w2 What is your monthly pension (including subsidy)? / ()_____ Yuan

[Soft Check: Verify if monthly benefits are low or high, e.g., prompt if FN005_w2 <200 yuan/month]

FN005_w2_bracket [IWER: IfR is unwilling to answer or does not remember, ask unfolding bracket questions here]

1000 /2,000 /3,000 /5,000 /10,000 yuan

**PROCEDURE** :<br>Skip to FN030_w2 FN030_w2

FN006_w2 Are you currently enrolled in pension program of the government and institutions or basic pension of the firms?

1. Yes, pension program of the government and institutions
2. Yes, basic pension insurance of the firms 3. Yes, both of them. /.
4. No → Skip to FN022_w3 FN022_w3

**PROCEDURE** :<br>FN006_w2 = 3FN007_w2－FN014_w2_bracket

FN007_w2 From which of the following work units did you get the pension insurance you just told us?

[Preloadpension program of government and institutions if FN006_w2 = 1; or preload Basic pension insurance of the firms if FN006_w2 = 2] [IWER: Choose (4) None of the aboveif the respondent has no work unit （4）]

1. Current work unit [for new R: preload FD003; for old R: ZF8 if FA006_w2_2a = 1 & FA006_w2_2a_3= 1, or FA006_w2_2a_1 if FA006_w2_2a= 2 & FA006_w2_3= 1, or FD003 if FA006_w2_2a_3= 2] → Skip to FN011_w2 FN011_w2
2. Last work unit [preload FL005 or ZF25] FL005 [FL005 or ZF25] → Skip to FN011_w2 FN011_w2
3. The work unit that processed retirement for the respondent [preload FM002] FM002 [FM002] → Skip to FN011_w2 FN011_w2
4. None of the above

**PROCEDURE** :<br>If answer to FN007_w2 = 4 (None of the above), then ask FN008_w2 －FN010_w2.<br>FN007_w2 = 4 () FN008_w2 －FN010_w2

FN008_w2 What is the name of the unit that provides you this pension insurance? / ____

FN009_w2 What was the type of the unit that provides you the pension insurance?

1. Government
2. Institutions
3. NGO ( )
4. Firm
5. Individual
6. Other, ______ (**FN009_w2_1**) ______ (**FN009_w2_1**)

FN010_w2 Where is this work unit located?

1. The same as permanent address
2. Another village/neighborhood in permanent addresss county/city/district // _____ (**FN010_w2_1**) //______ (**FN010_w2_2**) village/ neighborhood /
3. Other ______ (**FN010_w2_3**) province_city_county/ city/ district _ _ // __ (**FN010_w2_4**) // ______ (**FN010_w2_5**) village / neighborhood /
4. Abroad

[IWER:]

FN011_w2 In what month and year did you start to participate in the basic pension insurance of the firms through this work unit? ______ 1900...2015 (**FN011_w2_1**) year __ 0...12 (**FN011_w2_2**) month

[IWER: Mark the year using four digits. Take down the month as its actual number. For example, write January as 1 not 01, December as 12. If do not remember month, fill 0 : 4 . 1 1 0112 12. 0]

FN012_w2 Do you need to pay the premium by yourself or by work unit?

1. Yes
2. No → Skip to FN017_w2 FN017_w2

[/ 1 2]

FN013_w2 How much is the premium you paid? ______ (**FN013_w2_1**) Yuan per month / Or ______ 0.00...100.00 (**FN013_w2_2**) percent

FN014_w2 How much the premium your unit paid? ______ (**FN014_w2_1**) Yuan per month / Or ______ 0.00...100.00 (**FN014_w2_2**)

FN014_w2_bracket [IWER: IfR is unwilling to answer or does not remember, ask unfolding bracket questions here] 500/1,000 /2,000 /3,500 /5,000 yuan

FN017_w2 For how many years altogether have you been included in this program at local province? [Include years with other employers if the same plan.] / () / [/.]_____
0.00...100.00 Years

FN017_w3 For how many years altogether have you been included in this program in other province? [Include years with other employers if the same plan.] / () / [/ .]___
0.00...100.00 Years

FN018_w2 For how many years altogether will you have been included in this program when you retire? [Include years with other employers if the same plan.] () / [/ .]__
0.00...100.00 Years

FN019_w2 Will this be enough years to receive pension? /

1. Yes → Skip to FN021_w2 FN021_w2
2. No

FN020_w2 What do you plan to do?

1. I will pay the remaining premiums all in one payment at retirement to qualify for pension .
2. I will receive a one-time payment at retirement and not get pension . Skip to FN030_w2 FN030_w2
3. I will not receive pension . Skip to FN030_w2 FN030_w2

PROCEDURE :
If R answered yes to pension [FN019_w2 = 1 or FN020_w2 = 1], ask FN021_w2 - FN021_w2_bracket, then skip to FN030_w2. [FN019_w2= 1 or FN020_w2= 1]FN021_w2 - FN021_w2_bracket FN030_w2

FN021_w2 About how much do you expect your benefits to be? (as a percentage of your pay at retirement, or as an amount per month or year?) . _____ (**FN021_w2_1**) Yuan per month / Or _____ 0.00..100.00 (**FN021_w2_2**) % of final pay

FN021_w2_bracket [IWER: IfR is unwilling to answer or does not remember, ask unfolding bracket questions here] 500/1,000 /2,000 /3,500 /5,000 yuan

FN022_w3 Are you ever enrolled in pension program of the government and institutions?

1. Yes, pension program of the government and institutions
2. No Skip to FN030_w2 FN030_w2

FN023_w2 From which of the following work units did you get the pension insurance you just told us[preload pension program of government and institutions

[Preload pension program of government and institutions if FN022_w2 = 1; or preload Basic pension insurance of the firmsif FN022_w2 = 2; FN022_w2 = 1, , FN022_w2 = 2,]

[IWER: Choose (4) None of the above if the respondent has no work unit , (4)]

1. Current work unit [for new R: preload FD003; for old R: ZF8 if FA006_w2_2a = 1 & FA006_w2_3 = 1, or FA006_w2_2a_1 if FA006_w2_2a = 2 & FA006_w2_3 = 1, or FD003 if FA006_w2_2a_3 = 2] → Skip to FN027_w2 FN027_w2
2. Last work unit [preload FL005 or ZF25] FL005 [FL005 or ZF25] → Skip to FN027_w2 FN027_w2
3. The work unit that processed retirement for the respondent [preload FM002] FM002 [FM002] → Skip to FN027_w2FN027_w2
4. None of the above

**PROCEDURE** :<br>If answer to FN023_w2 = 4 (None of the above), then ask FN024_w2 – FN026_w2.<br>FN023_w2 = 4 () FN024_w2 – FN026_w2

FN024_w2 What is the name of the unit that provides you this pension insurance? ? ____

FN025_w2 What was the type of the unit that provides you the pension insurance?

1. Government
2. Institutions
3. NGO ( )
4. Firm
5. Individual
6. Other, ______ (**FN025_w2_1**) _____ (**FN025_w2_1**)

FN026_w2 Where is this work unit located?

1. The same as permanent address
2. Another village/neighborhood in permanent addresss county/city/district // __ (**FN026_w2_1**) // _____ (**FN026_w2_2**) vil- lage/neighborhood /
3. Other _____ (**FN026_w2_3**) province_city_county/city/district _ _ // __ (**FN026_w2_4**) //_____ (**FN026_w2_5**) village/neighborhood /

4. Abroad

[IWER:]

FN027_w2 In what month and year did you start to participate in the basic pension insurance of the firms through this work unit? _ 1900...2015 (**FN027_w2_1**) year _____ 0...12 (**FN027_w2_2**) month

[IWER: Mark the year using four digits. Take down the month as its actual number. For example, write January as 1 not 01, December as 12. If do not remember month, fill 0 : 4 . 1 1 0112 12. 0]

FN028_w2 In what month and year did you start to participate in the basic pension insurance of the firms through this work unit? _____ 1900...2015 (**FN028_w2_1**) year ___ 0...12 (**FN028_w2_2**) month

[IWER: Mark the year using four digits. Take down the month as its actual number. For example, write January as 1 not 01, December as 12. If do not remember month, fill 0 : 4 . 1 1 0112 12. 0]

FN029_w2 What was the reason for you to stop participating in this pension? (Multiple choice)

1. Business closed
2. I changed job/I was fired /
3. I moved to other city, pension can not be transferred 4. I thought it was not worth and exited

Part 2

FN030_w2 Did you participate in supplemental pension insurance of the government(institutions) firm or receive the pension?

1. Yes, but I dont receive it
2. Yes, I receive it
3. No → Skip to FN069_w3 FN069_w3

FN031_w2 Where did you participate in the insurance? _____ (**FN031_w2_1**) Province // _____ (**FN031_w2_2**) city _____ (**FN031_w2_3**) county

FN032_w2 What type of retirement pension plan is/was your employer s supplement pension?

1. Defined Benefit (DB) Retirement Pension
2. Defined Contribution (DC) Retirement Pension

DB Retirement Pension Plan: A workers retirement pension is determined in advance and the amount paid by the user shall change based on how well the savings are managed.

.

DC Retirement Pension Plan: The amount paid by the user is determined in advance and the retirement pension paid to a worker shall change based on how well the savings are managed. .

PROCEDURE :
Skip FN033_w2 - FN040_w2_bracket if FN030_w2 = 2 FN030_w2 = 2, FN033_w2 - FN040_w2_bracket

FN033_w2 For how many years altogether have you been included in this plan? [Include years with other employers if the same plan.] [.] _____ 0.00...100.00 Years

FN034_w2 At what age do you expect to start receiving benefits from this plan?

1. At age _____ 0...120 (**FN034_w2_1**) or in _____ 0...120 (**FN034_w2_2**) years
2. I do not expect receiving these benefits because I have received cash settlements → Skip to FN040_w2 FN040_w2
3. I do not expect receiving these benefits because I have lost benefits . → Skip to FN069_w3 FN069_w3
4. Other → Skip to FN069_w3 FN069_w3

FN035_w2 What is the combined monthly contribution from you and your employer? Of which, how much do you pay? _____ (**FN035_w2_1**) Yuan , _____ 0.00...100.00 (**FN035_w2_2**) %

FN035_w2_bracket [IWER: IfR is unwilling to answer or does not remember, ask unfolding bracket questions here] 500/1,000 /2,000 /3,500 /5,000 yuan

PROCEDURE :
If FN032_w2 = 1 ask: FN032_w2 = 1

FN036_w2 For a DB plan, do you know how much you are entitled to at (age in FN034_w2)? [preloadFN034_w2] _____ (**FN036_w2_1**) Yuan per month / Or _____ 0.00...100.00 (**FN036_w2_2**) Or _____ (**FN036_w2_3**) Yuan (Lump sum amount)

FN036_w2_bracket [IWER: IfR is unwilling to answer or does not remember, ask unfolding bracket questions here] 500/1,000 /2,000 /3,500 /5,000 yuan

**PROCEDURE** :<br>Skip to FN069_w3 FN069_w3

**PROCEDURE** :<br>If FN032_w2 = 2 ask: FN032_w2 = 2

FN037_w2 For a DC plan, have you ever checked your account balance?

1. Yes _____ (**FN037_w2_1**) Yuan in ______ (1900...2015 (**FN037_w2_2**) Year _____ (0...12 (**FN037_w2_3**) Month

   [IWER: Mark the year using four digits. Take down the month as its actual number. For example, write January as 1 not 01, December as 12 . If do not remember month, fill 0 : 4 . 1 10112 12. 0]
2. No

FN038_w2 What is the earliest age at which you could leave this employer and start to receive pension benefits? ____ 45...120 Years old

FN039_w2 By how much would your pension be reduced from full benefits if you left this job at (AGE IN FN038_w2)? [preload FN038_w2] By _____ 0.00...100.00 (**FN039_w2_1**) % or _ (**FN039_w2_2**) Yuan

FN039_w2_bracket [IWER: IfR is unwilling to answer or does not remember, ask unfolding bracket questions here] 500/1,000 /2,000 /3,500 /5,000 yuan

**PROCEDURE** :<br>Skip to FN069_w3 FN069_w3

FN040_w2 How much cash settlements did you receive? _____ Yuan

FN040_w2_bracket [IWER: IfR is unwilling to answer or does not remember, ask unfolding bracket questions here]

1000 /5,000 /10,000 /50,000 /100,000 yuan

**PROCEDURE** :<br>Skip to FN069_w3 FN069_w3

FN041_w2 In what month and year did you start to receive pension benefits from supplement pension insurance of the firms? _____ 1900...2015 (**FN041_w2_1**) year _____ 0...12 (**FN041_w2_2**) month

[ ] [IWER: Mark the year using four digits. Take down the month as its actual number. For example, write January as 1 not 01, December as 12. If do not remember month, fill 0 : 4 . 1 10112 12. 0]

FN042_w2 What are your monthly benefits? _____ Yuan per month /

FN042_w2_bracket [IWER: IfR is unwilling to answer or does not remember, ask unfolding bracket questions here] 500/1,000 /2,000 /3,500 /5,000 yuan

Part 3

FN069_w3 ( ) / .

1. .
2. _____ (**FN069_w3_1**)
3. .
4. /.
5. _____ (**FN069_w3_2**).

FN057_w3 What kind of pension did you participate in or receive? (multiple choice) /

1.
2.
3.
4.

PROCEDURE :

If FN057_w3=4 and (FN069_w3=1 or FN069_w3=2) , ask FN057_w3_0.

FN057_w3=4 (FN069_w3=1 FN069_w3=2) FN057_w3_0

If FN057_w3=4 and (FN069_w3=3 or FN069_w3=4 or FN069_w3=5) , skip to FN079_w2_1. FN057_w3=4 (FN069_w3=3 FN069_w3=4 FN069_w3=5) FN079_w2_1

If FN057_w3 is multiple choiced (FN057_w3=1 or FN057_w3=2 or FN057_w3=3), ask FN058_w2-FN075_w3_5 for every answer FN075_w3_5 (=1 =2 =3) FN058_w2-FN075_w3_5

FN057_w3_0 1.
2.
3.

PROCEDURE :
If FN057_w3_0 is multiple choiced , ask FN057_w3_1-FN057_w3_6 for every answer
FN057_w3_0 FN057_w3_1-FN057_w3_6

FN057_w3_ 1 [FN057_w3_0]
[ ]
1. Skip to FN057_w3_2.
FN057_w3_2
2. Skip to FN057_w3_3. FN057_w3_3
3. Skip to FN057_w3_4. FN057_w3_4

FN057_w3_2 / [FN057_w3_0]
1.
2.

PROCEDURE :
Skip to FN057_w3_5. FN057_w3_5

FN057_w3_3 / [FN057_w3_0]
1. / 2.
3.
4.
5.
6.
7. _____ (**FN057_w3_3_1**)

PROCEDURE :
Skip to FN057_w3_5. FN057_w3_5

FN057_w3_4 [FN057_w3_0] 1.
2. skip to FN057_w3_6. FN057_w3_6

FN057_w3_5 [FN057_w3_0]

1.
2.
3.
4.
5.
6. _____ (**FN057_w3_5_1**)

**PROCEDURE** :<br>Skip to FN079_w2_1. FN079_w2_1

FN057_w3_6 [FN057_w3_0]

1.
2. .
3.
4.
5.
6.
7.
8. _____ (**FN057_w3_6_1**)

**PROCEDURE** :<br>Skip to FN079_w2_1. FN079_w2_1

FN058_w2 For every answers of FN057_w3 , do you participate in or receive the pension?

[FN057_w3] ( )

1. Participate in /
2. Receive the pension

FN059_w2 Where did you participate in the pension? [FN057_w3]

1. The same as permanent address
2. Another village/neighborhood in permanent addresss county/city/district // __ (**FN059_w2_1**) // _____ (**FN059_w2_2**) vil- lage/neighborhood /
3. Other _____ (**FN059_w2_3**) province_city_county/city/district _ _ // __ (**FN059_w2_4**) //_____ (**FN059_w2_5**) village/neighborhood /
4. Abroad

[IWER:]

FN061_w3 [FN057_w3] _____ 1900…2015 (**FN061_w3_1**) year _____ 0…12 (**FN061_w3_2**) month

PROCEDURE :
Skip FN060_w2-FN066_w2_bracket if FN058_w2 = 2. FN058_w2 = 2, FN060_w2-FN066_w2_bracket

FN060_w2 Have you ever contributed to your [preload FN057_w3]? [FN057_w3]
[CAPI: (1) (2) IfFN057_w3 = 1 or FN057_w3 = 2, prompt: If your residents pension was transferred from other pension programs, your contribution to these other programs also counts. FN057_w3 = 1 or FN057_w3 = 2]

1. Yes
2. No → FN063_w2

FN072_w3 [FN057_w3]

1.
2. Skip to FN072_w3_4 FN072_w3_4

[IWER: If R is unwilling to answer or does not remember, ask unfolding bracket questions here ( ) 60 15 , ,]

FN072_w3_1

1. ______ **(FN072_w3_1_1**)
2. ______ **(FN072_w3_1_1**)

FN072_w3_2

1. ______ **(FN072_w3_2_1**)
2. ______ **(FN072_w3_2_2**)

FN074_w3 ()

1
2-26 []
27
28 ______ **(FN074_w3_1)**

FN072_w3_4 [FN057_w3]

1. ______ **(FN072_w3_4_1**) ______ **(FN072_w3_4_2**) ______ **(FN072_w3_4_3**) ______ **(FN072_w3_4_4**) .
2. Skip to FN063_w2 FN063_w2

FN072_w3_5 ( )

1
2-26 []
27
28 _____ (**FN072_w3_5_1**)

> **PROCEDURE** :
> Ask FN063_w2 and FN064_w2 if the respondent is enrolled in residents pension FN057_w3 = 2 or FN057_w3 = 3 . If FN057_w3 = 1, skip to FN065_w2 FN057_w3 = 1, FN065_w2. FN057_w3 = 2 FN057_w3 = 3FN063_w2FN064_w2

FN063_w2 1. Yes
2. No → Skip FN064_w2 FN064_w2
[（1）. 1992 .（2） .]

FN064_w2
1. _____ (**FN064_w2_1**)
2.

FN065_w2 When do you expect to receive pension At age ____ 45…120 (**FN065_w2_1**) years old or in _____ 0.00…100.00 (**FN065_w2_2**) years
[Soft Check: Prompt or Verify if FN065_w2_1 <50 or FN065_w2_2 >60]

FN066_w2 About how much do you expect your benefits to be? (as an amount per month or year or a lump sum?) _____ (**FN066_w2_1**) Yuan per month / Or _____ (**FN066_w2_2**) Yuan （Lump sum amount ）
[Soft Check: Prompt or Verify if These Benefits are Low or High, e.g., FN066_w2_1 <100 | FN066_w2_1 >5000 per month.]

FN066_w2_bracket [IWER: IfR is unwilling to answer or does not remember, ask unfolding bracket questions here] 500/1,000 /2,000 /3,500 /5,000 yuan

> **PROCEDURE** :
> If FN058_w2 = 2 ask FN067_w2-FN075_w3_5
> Otherwise skip to FN079_w2_1. FN079_w2_1

FN067_w2 In what month and year did you start to receive your [preload FN057_w3]? [FN057_w3]_____ 1900...2015 (**FN067_w2_1**) year _____ 0...12 (**FN067_w2_2**) month

[IWER: Mark the year using four digits. Take down the month as its actual number. For example, write January as 1 not 01, December as 12. If do not remember month, fill 0 : 4 . 1 1 01,12 12. 0]

FN068_w2 How much do you receive now? (as an amount per month?) Yuan per month /

FN068_w2_bracket [IWER: IfR is unwilling to answer or does not remember, ask unfolding bracket questions here] 500/1,000 /2,000 /3,500 /5,000 yuan

FN068_w3 / FN057_w3 FN057_w3 [ ]

1.
2. FN075_w3 FN075_w3

FN068_w3_1 / FN057_w3 /

1.
2. Skip to FN075_w3 FN075_w3

FN068_w3_2 /FN057_w3

1-25 []

26

FN075_w3 FN057_w3

1.
2. Skip to FN075_w3_4 FN075_w3_4

[IWER: If R is unwilling to answer or does not remember, ask unfolding bracket questions here ( ) 60 15 , ,]

FN075_w3_1

1. _____ (**FN075_w3_1_1**)
2. _____ (**FN075_w3_1_2**)

FN075_w3_2

1. _____ (**FN075_w3_2_1**)

2. _____ (**FN075_w3_2_2**)

FN076_w3 ()

1
2-26 []
27
28 _____ (**FN076_w3_1**)

FN075_w3_4 FN057_w3

1. _____ (**FN075_w3_4_1**) _____ (**FN075_w3_4_2**) .
2. Skip to FN079_w2_1. FN079_w2_1.

FN075_w3_5 ( )

1
2-26 []
27
28 _____ (**FN075_w3_5_1**)

Part 4

FN079_w2_1 Whether your land was acquired?

1. Yes
2. No → Skip to FN080_w2 FN080_w2

FN079_w2_2 When was your land acquired? _____ 1900...2015 Year

FN079_w2_3 Did you participate inland expropriation pension insurance, or did you receive the pension? /

1. Yes, but I dont receive it
2. Yes, I receive it → Skip to FN079_w2_10FN079_w2_10 3. No → Skip to FN080_w2 FN080_w2

FN079_w2_4 Do you need to pay the premium by yourself?

1. Yes
2. No → Skip to FN079_w2_7 FN079_w2_7

FN079_w2_5 Who paid for this pension insurance? 1. Myself

2. My children
3. Other family member or relative

4. Others

FN079_w2_6 How much did you need to pay? _____ (**FN079_w2_6_1**) Yuan per month / Or _____ (**FN079_w2_6_2**) Yuan per year /

FN079_w2_7 How much did government and community subsidize? Government paid ____ (**FN079_w2_7_1**) Yuan per month / Or _____ (**FN079_w2_7_2**) Yuan per year / Community paid _____ (**FN079_w2_7_3**) Yuan per month / Or _____ (**FN079_w2_7_4**) Yuan per year /

FN079_w2_8 When do you expect to receive this pension At age _____ 45...120 (**FN079_w2_8_1**) years old or in _____ 0.00...100.00 (**FN079_w2_8_2**) years

FN079_w2_9 About how much do you expect your benefits to be? (as an amount per month or year or a lump sum?) _____ (**FN079_w2_9_1**) Yuan per month / Or _____ (**FN079_w2_9_2**) Yuan （Lump sum amount ）

**PROCEDURE** :
If FN079_w2_3 = 2 ask FN079_w2_10-FFN079_w2_11_bracket<br>Otherwise skip to FN080_w2. FN080_w2

FN079_w2_10 In what month and year did you start to receive this pension? _____ 1990...2015 (**FN079_w2_10_1**) year _____ 0...12 (**FN079_w2_10_2**) month

[IWER: Mark the year using four digits. Take down the month as its actual number. For example, write January as 1 not 01, December as 12. If do not remember month, fill 0 : 4 . 1 1 0112 12. 0]

FN079_w2_11 How much do you receive now? (as an amount per month?) _____ Yuan per month /

FN079_w2_11_bracket [IWER: If R is unwilling to answer or does not remember, ask unfolding bracket questions here] 10 /50 /100 /500 /1,000 yuan /

Part 5

FN080_w2 Did you receive old age pension allowance? 1. Yes

2. No → Skip to FN056_w2_1 FN056_w2_1

FN081_w2 In what month and year did you start to receive the pension subsidy for the oldest old? _____ 1990...2015 (**FN081_w2_1**) year _____ 0...12 (**FN081_w2_1**) month

[( ) 60 ,] [IWER: Mark the year using four digits. Take down the month as its actual number. For example, write January as 1 not 01, December as 12. If do not remember month, fill 0 : 4 . 1 1 0112 12. 0]

FN082_w2 How much do you receive now? _____ Yuan per month /

FN082_w2_bracket [IWER: IfR is unwilling to answer or does not remember, ask unfolding bracket questions here] 500/1,000 /2,000 /3,500 /5,000 yuan

Part 6

FN056_w2_1 Did you participate in life insurance? 1. Yes
2. No → Skip to FN043_w2 FN043_w2

FN056_w2_2 When did you start to participate in life insurance? _ 1900...2015 (**FN056_w2_2_1**) year _____ 0...12 (**FN056_w2_2_2**) month

FN056_w2_3 What kind of life insurance did you participate in? 1. Term life insurance
2. Whole life insurance
3. Survivorship insurance
4. Endowment insurance
5. Other, please specify (______) (**FN056_w2_3_1**)

FN056_w2_4 Who paid for your life insurance?
1. Myself
2. My children
3. Other family member or relative
4. Others

FN056_w2_5 How to pay the insurance premium?
1. Monthly
2. Quaterly
3. Half a year

4. Annually

FN056_w2_6 How much is the payment amount?

1. If pay it monthly ______ Yuan/Month / (**FN056_w2_6_1**)
2. If pay it quarterly ______ Yuan/Quarter / (**FN056_w2_6_2**)
3. If pay it every six months _____ Yuan/Half a year / (**FN056_w2_6_3**) 4. If pay it annually Yuan/Year / (**FN056_w2_6_4**)

FN056_w2_7 Did you receive reimbursement from your life insurance?

1. Yes
2. No → Skip to FN056_w2_9 FN056_w2_9

FN056_w2_8 How much did you get? ______ Yuan

FN056_w2_9 How much do you expect to receive? _____ (**FN056_w2_9_1**) Yuan/Month / Or _____ (**FN056_w2_9_2**) Yuan （Lump sum amount ）

Part 7 老 (人)

FN043_w2 Did you participate in commercial pension insurance, did someone buy commercial pension insurance for you, or did you get commercial pension insurance? /

1. Yes, but I dont receive it /
2. Yes, I receive it
3. No → Skip to FN083_w2 FN083_w2

FN044_w2 Where did you participate in this insurance? 1. The same as permanent address

2. Another village/neighborhood in permanent addresss county/city/district // __ (**FN044_w2_1**) // _____ (**FN044_w2_2**) vil- lage/neighborhood /
3. Other _____ (**FN044_w2_3**) province_city_county/city/district _ _ // __ (**FN044_w2_4**) //_____ (**FN044_w2_5**) village/neighborhood /

4. Abroad []

FN045_w2 Who paid for the commercial pension insurance? 1. Myself

2. My employer
3. My family or relative
4. Others

[ ]

PROCEDURE :
Skip FN046_w2-FN054_w2_bracket if FN043_w2 = 2 FN043_w2 = 2 ,
FN046_w2-FN054_w2_bracket

FN046_w2 When did you start paying for the commercial pension? ______ 1900…2015 (**FN046_w2_1**) year ______ 0…12 (**FN046_w2_2**) month

[ ] [IWER: Mark the year using four digits. Take down the month as its actual number. For example, write January as 1 not 01, December as 12. If do not remember month, fill 0 : 4 . 1 10112 12. 0]

FN047_w2 How do you contribute to the commercial pension? ? 1. Annual payment

2. Lump sum amount → Skip to FN050_w2 FN050_w2

FN048_w2 You contribute ______ yuan/ year to the commercial insurance ______ Yuan/ year /

FN049_w2 How many years do you need to pay? ? ______ Years

[Soft Check: Prompt for verification if greater than a legal maximum]

PROCEDURE :
Skip FN050_w2-FN050_w2_bracket if FN047_w2 = 1 FN047_w2 = 1 ,
FN050_w2-FN050_w2_bracket

FN050_w2 How much premium do you need to pay in total? __ Yuan

FN050_w2_bracket [IWER: IfR is unwilling to answer or does not remember, ask unfolding bracket questions here]

1,000 /2,000 /5,000 /10,000 yuan

FN051_w2 How do you receive the pension?

1. Lump sum amount → Skip FN052_w2-FN053_w2_bracketFN052_w2-FN053_w2_bracket
2. Yearly
3. Monthly

PROCEDURE :
If FN051_w2 = 2 ask FN052_w2 and FN052_w2_bracket

FN052_w2 How much do you expect to receive ______ yuan/ year after your retirement?
Yuan/year /

[Soft Check: Prompt for verification if low or high, e.g. FN052_w2 < 1200 per year or FN052_w2 > 60000 per year]

FN052_w2_bracket [IWER: IfR is unwilling to answer or does not remember, ask unfolding bracket questions here] 500/1,000 /2,000 /3,500 /5,000 yuan

PROCEDURE :
If FN051_w2 = 3 ask FN053_w2 and FN053_w2_bracket

FN053_w2 How much do you expect to receive ______ yuan/month in the future? ______
Yuan/ month /

[Soft Check: Prompt for verification if low or high, e.g. FN053_w2 < 100 per month or FN053_w2 >5000 per year]

FN053_w2_bracket [IWER: IfR is unwilling to answer or does not remember, ask unfolding bracket questions here] 500/1,000 /2,000 /3,500 /5,000 yuan

FN054_w2 How much do you expect to receive ________ yuan in total? ______ Yuan

FN054_w2_bracket [IWER: IfR is unwilling to answer or does not remember, ask unfolding bracket questions here]
1,000 /5,000 /10,000 /50,000 /100,000 yuan

PROCEDURE :
Skip to FN083_w2. FN083_w2

FN055_w2 In what month and year did you start to receive commercial pension benefits?
1900…2015 (**FN055_w2_1**) year ______ 0…12 (**FN055_w2_2**) month

[IWER: Mark the year using four digits. Take down the month as its actual number. For example, write January as 1 not 01, December as 12. If do not remember month, fill 0 : 4 . 1 1 0112 12. 0]

FN056_w2 What is your monthly benefit? ? ______ Yuan per month /

FN056_w2_bracket [IWER: IfR is unwilling to answer or does not remember, ask unfolding bracket questions here] 500/1,000 /2,000 /3,500 /5,000 yuan

Part 8 老

FN083_w2 You just told us you are receiving benefits from other pension program, what is the name of this pension program? /

1. Yes /
2. Yes
3. No → Skip to FN097_w2 FN097_w2

FN084_w2 What is the name of the program? ______
[/]

FN085_w2 where did you participate in the pension program? 1. The same as permanent address

2. Another village/neighborhood in permanent addresss county/city/district // __ (**FN085_w2_1**) // _____ (**FN085_w2_2**) vil- lage/neighborhood /
3. Other _____ (**FN085_w2_3**) province_city_county/city/district _ _ // __ (**FN085_w2_4**) //_____ (**FN085_w2_5**) village/neighborhood /
4. Abroad

[]

PROCEDURE :
Skip FN086_w2-FN094_w2_2_bracket if FN083_w2 = 2. FN083_w2 = 2, FN086_w2-FN094_w2_2_bracket

FN086_w2 When did you start to participate in this pension? _____
1990...2015(**FN086_w2_1**)year _____ 0...12 (**FN086_w2_2**)month

[IWER: Mark the year using four digits. Take down the month as its actual number. For example, write January as 1 not 01, December as 12. If do not remember month, fill 0: 4 . 1 1 01,12 12. 0]

FN087_w2 Did you need to pay the premium? 1. Yes

2. No → Skip to FN093_w2 FN093_w2

FN088_w2 Who paid for the this pension insurance? 1. Myself

2. My employer
3. My family or relative

4. Others

FN089_w2 How do you contribute to the commercial pension? ? 1. Annual/Monthly payment /
2. Lump sum amount → Skip to FN092_w2 FN092_w2

FN090_w2 You contribute ______ Yuan/ year to the commercial insurance ______ Yuan/ year /

FN091_w2 How many years do you need to pay? ? _____ Years
[Soft Check: Prompt for verification if greater than a legal maximum]

PROCEDURE :
Skip FN092_w2-FN092_w2_bracket if FN089_w2 = 1. FN089_w2 = 1, FN092_w2-FN092_w2_bracket

FN092_w2 How much premium do you need to pay in total? __ Yuan

FN092_w2_bracket [IWER: IfR is unwilling to answer or does not remember, ask unfolding bracket questions here]
2,000 /5,000 /10,000 /50,000/100,000 yuan

FN093_w2 When do you expect to receive pension At age _____ (**FN093_w2_1**) 45..120 or in (**FN093_w2_2**) years .

FN094_w2 About how much do you expect your benefits to be? (as an amount per month or year or a lump sum?) _____ (**FN094_w2_1**) Yuan per month / Or _____ (**FN094_w2_1**) Yuan （Lump sum amount ）

PROCEDURE :
If FN083_w2 = 2 ask FN095_w2 - FN096_w2_bracket . FN083_w2 = 2 FN095_w2 - FN096_w2_bracket
Otherwise skip to FN097_w2. FN097_w2

FN095_w2 In what month and year did you start to receive this pension benefits? （） __ 1990…2015 (**FN095_w2_1**) year _____ 0…12 (**FN095_w2_2**) month

[IWER: Mark the year using four digits. Take down the month as its actual number. For example, write January as 1 not 01, December as 12. If do not remember month, fill 0 : 4 . 1 1 0112 12. 0]

FN096_w2 How much do you receive now? _____ Yuan per month /

FN096_w2_bracket [IWER: IfR is unwilling to answer or does not remember, ask unfolding bracket questions here] 500/1,000 /2,000 /3,500 /5,000 yuan

[Ask all R]

FN097_w2 Who do you think you can rely on financially for old-age support

1. Children → Skip to FN098_w2 FN098_w2
2. Savings
3. Pension or retirement salary
4. Commercial pension insurance
5. Other

[ ]

FN098_w2 Which child(ren)? / (choose all that apply) [ ]

This page intentionally left blank

G&H INCOME, EXPENDITURES AND ASSETS

G2 HOUSEHOLD INCOME AND EXPENDITURES

[IWER: Part 1_1 is asked of the main respondent and spouse respectively. Other parts in this section is asked of the family respondent. Do not allow a proxy respondent to answer the entire section. Part 1_1 . .]

PART 1 Household Wage Income and Individual-based transfers
入人入

Part 1_1: Main Respondent and Spouses Wage Income and Individual-based Transfers
工入人入

[IWER: Please conduct Part 1_1 when the main respondent and spouse are at home. Dont allow a proxy to complete the part. Part 1_1 ..]

GA001 Did you receive any wage and bonus income in the past year? （）

1. Yes
2. No → Skip to GA003 GA003

GA002 How much did you receive last year? _____ Yuan

Wage income: yuan [soft check >240,000]

GA002_bracket [IWER: If R is unwilling to answer or does not remember, ask unfolding bracket questions here.]

10,000 /30,000 /50,000 /100,000 /200,000 Yuan

GA002_W2_1 Does the above mentioned wage exclude any insurance, income tax, public housing funds and other fees?

1. Yes
2. No

GA002_W2_2 What is the total amount ofyour insurance, income tax, public housing funds and other fees? /

1. ______ (**GA002_w2_2a**) Yuan/Year /

2. _____ (**GA002_w2_2b**) Yuan/Month /
3. About ___ (**GA002_w2_2c**) % of wage
4. No

[IWER: If R is unwilling to answer or does not remember, ask unfolding bracket questions here. :] / 300 /500 /1,000 /2,000 /3,000 Yuan/Month /.

Among it, :

1. Income tax
   1. ________ **GA002_w2_2_1a**)Yuan/Year /
   2. _____ (**GA002_w2_2_1b**)Yuan/Month /
   3. About ___ (**GA002_w2_2_1c**) % of wage
   4. No
2. Various insurance (pension insurance, health insurance, unemployment insurance, workers injury insurance, maternity insurance) ( )
   1. _______ (**GA002_w2_2_2a**)Yuan/Year /
   2. _____ (**GA002_w2_2_2b**)Yuan/Month /
   3. About ___ (**GA002_w2_2_2c**) % of wage
   4. No
3. Public housing fund
   1. _______ (**GA002_w2_2_3a**))Yuan/Year /
   2. _____ (**GA002_w2_2_3b**)Yuan/Month /
   3. About ___ (**GA002_w2_2_3c**) % of wage
   4. No

GA003 Did you receive any of the following types of individual income in the past year? (check all that apply) ()

2 Unemployment compensation
3 Pension subsidy for the oldest old/pension voucher /
4 Workers compensation from Industrial Accident Compensation Insurance includes wage-replacement benefits, disability benefits, and survivors benefits
5 Elderly family planning subsidies
6 Medical aid
7 Other government subsidies
8 Social assistance
9 Other income sources
10 None of the above → Skip GA004 GA004

GA004 How much did you receive last year? _____ Yuan

PROCEDURE :

If GA003 = 3, ask GA004_W3 and GA004_W3_1. / , GA004_W3GA004_W3_1.

GA004_W3 What type of the pension subsidy? / ( )

1. Cash → Skip to Part 1_2 Part 1_2
2. Card
3. Voucher

GA004_W3_1 How do you use the card/voucher? / () 1. Life care

2. Domestic service
3. Rehabilitation service
4. Buy food
5. Buy medicine
6. Buy life items
7. Other, please specify ____(**GA004_W3_1_1**)

Part 1_2: Other Household Members Wage Income and Individual-based transfers

[IWER reminder: make sure others are not present. .]

[Intro: We d like to ask you some questions about the income and assets of OTHER members of your household. . .]

PROCEDURE :

The names of other household members are preloaded from the cover screen information. For each member (excluding main respondent and spouse)ask GA005 to GA008_bracket. . ( ) GA005GA008_bracket.

GA005 Did [preload household member name] receive any wage and bonus income in the past year? [preload household member name] ( )

1. Yes
2. No → Skip to GA007 GA007

GA006 After tax and employee social insurance , how much did he/she receive in the past year year? / ______ (**GA006_1**) Yuan or _____ (**GA006_2**) Yuan/Month

Wage income: yuan [soft check >200,000] or yuan/month [soft check>15,000]

GA006_bracket [IWER: If R is unwilling to answer or does not remember, ask unfolding bracket questions here.]

10,000 /30,000 /50,000 /100,000 /200,000 Yuan

GA006_w2 How much did he/she pay the tax and employee social insurance in the past year year? [preload household member name] __ (**GA006_w2_1**) Yuan or _____ (**GA006_w2_2**) Yuan/Month /.

GA007 Did [preload household member name] receive any of the following types of individual income in the past year? (check all that apply) [preload household member name] ( )

1. Pensions (including wages from government institutions and firms, supplemental pension of the firms,and income from such programs as rural pension insurance, Urban residents pension and commercial pension insurance, new rural social pension insurance and pension subsidy for the oldest old) ( )
2. unemployment compensation
3. pension subsidy
4. Workers compensation from Industrial Accident Compensation Insurance includes wage-replacement benefits, disability benefits, and survivors benefits
5. elderly family planning subsidies
6. medical aid
7. other government subsidies
8. social assistance
9. other income sources
10. None of the above → Skip GA008 GA008

[F1 . , , .]

GA008 How much did he/she receive last year? ____ (**GA008_b**) Yuan [soft check >10000] or ____ (**GA008_c**) Yuan/Month / [soft check >3000]

PROCEDURE :

If GA007 = 1 and GA008 = DK, ask GA008_bracket. GA008_bracket

GA008_bracket [IWER: If R is unwilling to answer or does not remember, ask unfolding bracket questions here. :]
10,000 /30,000 /50,000 /100,000 /200,000 Yuan

**PROCEDURE** :
Skip to next person. .

PART 2 HOUSEHOLD AGRICULTURAL INCOME AND EXPENDITURE

[Intro: Next we will ask some questions about your household agricultural income and expenditure. .]

GB001 Did your household engage in agricultural work (including cropping, forestry, livestock, and fish) last year?

1. Yes
2. No → Skip to GC001 GC001

GB002 [IWER: The names of other household members not including respondent and spouse are preloaded from the cover screen information:] [preload other household member name] Who engaged in agricultural work in the past year? ()

Crops and forestry products

GB003 Did your household engage in cropping or forestry last year? ()

1. Yes
2. No → Skip to GB007 GB007

GB004 When was the most recent harvest? ____2009...2015 (**GB004_1**) Year ____0...12 (**GB004_2**) Month

[IWER: Mark the year using four digits. Take down the month as its actual number. For example, write January as 1 not 01, December as 12. If do not remember month, fill 0. : 4 . 1 1 0112 12. 0.]

GB005 What is the total value of all crops and forestry products produced in the past year? (**GB005_1**) Yuan [soft check: 75000 yuan] Among it, what is the value of the crops and forestry products that is home consumed? ____ (**GB005_2**)Yuan __ (**GB005_3**) %

GB005_bracket [IWER: If R is unwilling to answer or does not remember, ask unfolding bracket questions here. :]
1,000 /3,000 /5,000 /7,000 /10,000 Yuan

GB005_w2_bracket [IWER: IfR is unwilling to answer or does not remember, ask unfolding bracket questions here. :]
1,000 /3,000 /5,000 /7,000 /10,000 Yuan

GB006 What was the total cost of producing crops (including vegetables and Chinese herbs) and forestry products in the past year? (including Seeds (including home-used seeds), Fertilizer, Organic fertilizer, Pesticide, Plastic sheets, Hiring labor (including with machine or animals), Land rents, Rents (excluding land rents), Irrigation, Fuel, Transportation, Processing, Marketing (including packaging, management fee)) ( ) ( ) [( ) ( ) ( ) ( )] ____ Yuan [soft check: 50,000 yuan]

GB006_bracket [IWER: If R is unwilling to answer or does not remember, ask unfolding bracket questions here. :]
300 /600 /1,000 /2000 /5,000 Yuan

Livestock and fisheries 水

GB007 Did your household grow any livestock or aquatic life last year? ( )
1. Yes
2. No → Skip to GC001 GC001

GB008 What is the current value of all livestock（including chicken, duck, cattle, pig, sheep, etc.）and aquatic life? ( ) ____ Yuan [soft check: 100,000 yuan]

GB008_bracket [IWER: If R is unwilling to answer or does not remember, ask unfolding bracket questions here. :]
500 /1,500 /3,000 /4,500 /9,000 Yuan

GB009 What was the value of all livestock and aquatic life at this time last year? ____ Yuan [soft check: 100,000 yuan]

GB009_bracket [IWER: If R is unwilling to answer or does not remember, ask unfolding bracket questions here. :]
500 /1,500 /2,500 /4,000 /8,000 Yuan

GB010 How much did you spend purchasing new livestock and aquatic life in the past year? Yuan [soft check: 50,000]

GB011 What was the value of all livestock and aquatic life that were sold or consumed in the past year? ?____ (**GB011_1**) Yuan [soft check: 100,000 yuan] Among it, what is the amount or percent consumed at your home? ____ (**GB011_2**) Yuan ___ (**GB011_3**) %

GB011_bracket [IWER: If R is unwilling to answer or does not remember, ask unfolding bracket questions here. :]
200 /900 /1,500 /2,500 /5,000 Yuan

GB011_w2_bracket [IWER: IfR is unwilling to answer or does not remember, ask unfolding bracket questions here. :]
200 /900 /1,500 /2,500 /5,000 Yuan

GB012 What was the value of all livestock products produced (including the self consumption value) in the past year, including milk, wool (including cashmere, sheep or goat skin), and eggs? ( ) ( ) ____ (**GB012_1**) Yuan [soft check: 50,000 yuan] Among it, what is the amount or percent consumed at your home? ____ (**GB012_2**) Yuan ___ (**GB012_3**) % [soft check: 50,000 yuan]

GB012_bracket [IWER: If R is unwilling to answer or does not remember, ask unfolding bracket questions here. :]
( ) 100 /200 /300 /500 /1,000 Yuan

GB012_w2_bracket [IWER: IfR is unwilling to answer or does not remember, ask unfolding bracket questions here. :]
100 /200 /300 /500 /1,000 Yuan

GB013 What was the cost of producing livestock and aquatic life in the past year, including the value of all feed, medicine, pasture fees, animal pens, wages, etc. ____ Yuan
[soft check: 50,000 yuan] [soft check: reported raising livestock but no evidence of such activityif GB008 = 0 and GB009 = 0 GB008 = 0 GB009 = 0]

PART 3 Self-employed Activities

GC001 Did your household members engage in any self-employed activities last year?

1. Yes
2. No → Skip to GD001 GD001

GC002 How many types of activities did your household members participate in the past year? ____ Activities.

**PROCEDURE**: For each self-employed activity in GC002, ask GC003 - GC005. GC003 - GC005

GC003 Who engaged in this self-employment businesse in the past year?

[IWER: All the names of household members are preloaded from the cover screen information ( )] ( )

GC004 Which types of activities?

1. Services (cooking, sewing, private clinic etc.) ( )
2. Transportation
3. Construction
4. Mining
5. Processing production
6. Business
7. Others, please specify ____(**GC004_1**)

GC005 Not including fixed capital costs, what is your best estimate of the net income earned from this activity by your household members last year? [If the activity was conducted jointly with non-household members, report only the net income earned by household members. Remember to consider the following types of costs: energy, housing or equipment rental, raw materials, transportation, marketing, wages, taxes or fees.]

. . ____ Yuan

[soft check: 500,000 yuan]

GC005_bracket [IWER: If R is unwilling to answer or does not remember, ask unfolding bracket questions here. :]

5,000 /10,000 /50,000 /100,000 /200,000 Yuan

PART 4 HOUSEHOLD PUBLIC TRANSFER INCOME

[We ask the public transfers received by the households (with household as the unit). Public transfers have characteristic of welfare, such as Wubaohu Subsidy and Tekunhu Subsidy gived by government. . . ..]

GD001 How much Dibao assistance did your household receive last year? (if not applicable, fill in 0 yuan). （0） ____ Yuan

GD002 Did your household receive any of the following government subsidies in the past year? (check all that apply) （）

[soft checks for each category: 20,000 yuan 20,000 .]

1. Reforestation : how much? ____ (**GD002_1**) Yuan
2. Agricultural subsidies : how much? ____ (**GD002_2**) Yuan
3. Wubaohu (targets low-income, blind, disabled, aged persons, and young persons that have no means to support themselves. （ : how much? ____ (**GD002_3**) Yuan
4. Tekunhu : how much? ____ (**GD002_4**) Yuan
5. Work injury subsidies to the immediate family members how much? ____ (**GD002_5**) Yuan
6. Emergency or disaster relief (jiujikuan, jiuzaikuan) last year? （） （） : how much? ____ (**GD002_6**) Yuan
7. Other, please specify ____(**GD002_other**): how much? ___ (**GD002_7**) Yuan
8. None → Skip to GD003 GD003

GD003 Did your household receive any income from the following sources in the past year? (check all that apply) （）

1. Donations from the society (including cash, and items like food, clothing, etc.) （ ） : how much? ____ (**GD003_1**) Yuan [soft check: 20,000 yuan]
2. Compensation for land seizure last? : how much? ____ (**GD003_2**) Yuan [soft check: 100,000 yuan]
3. Compensation to pulling down your house or apartment last year? : how much? ____(**GD003_3**) Yuan [soft check: 100,000 yuan] 4. None

PART 5 HOUSEHOLD LIVING EXPENDITURE

[, （

) . .]

GE001 We wish to know your family food expenditure for the last week. Are you the primary person who purchases food for the household?

1. Yes → skip to GE004 GE004
2. No

GE002 Who is the primary person purchasing food for the household?

[CAPI: Preload all the HHmember list]

1. HHmember list
2. Other children ( ) 3. Nanny
4. Neighbor
5. Other, please specify

[IWER: If possible, the primary person who purchases food for the household should answer the questions about expenditures FE003-FE009] [ ]

GE004 In the past week, how many people usually ate meals together in your household (not including guests)? ( ) __ Persons

[soft check: 10]

GE005 Last week how many meals did you provide to guests? ( ) ____ Meals [soft check: 100]

[Intro: The next questions are about your household living expenditure, including your household members (preloaded names of household members) living expenditure. If one attends school/work outside and comes home almost every week, GE006 - GE008 includes his/her expenditureon food and meals outside. If one attends school/work outside but not come home every week, GE006 - GE008 excludes his/her expenditureon food and meals outside.] [. . / /, .]

GE006 In the past week, how much did your household spend on food (excluding eating out expenditure, alcohol, cigarettes, cigars and tobacco expenditure)? ____ Yuan [soft check: 6000 yuan]

GE006_W2 Does your household produce agricultural products yourself (including plants, meat, eggs, aquatic lives, oil, vegetables and fruits, cigaretts and wine, drinks and milk products, produced food, seasonings, etc.)? ( )

1. Yes
2. No → skip to GE007 GE007

GE006_W2_1 In the past week, what was the market value of the food that members of the household consumed that you grew yourselves? ____ Yuan

GE007 Among it, how much did your household spend on eating out? ____ Yuan [soft check: 3000 yuan]

GE008 Among it, how much did your household spend on alcohol, Cigarettes, cigars and tobacco? ____ Yuan [soft check: 3000 yuan]

GE009 Please tell me the expenditure last month for your household for the following items. .

[soft check for each category: 5000 yuan] [IWER: fill in 0 if no corresponding expenditure; fill in -9999 if the respondent cannot recall the expenditure. : 0 -9999]

1. Communication fees (including post, internet usage, telephone and cell phone usage) ( ) ____ **(GE009_1)** Yuan
2. Utilities: Water and electricity ____ **(GE009_2)** Yuan
3. Fuels (including gas, coal, etc.) ( ) ____ **(GE009_3)** Yuan
4. Fees for Matron, housekeepers and servants __**(GE009_4)** Yuan
5. Local Transportation ____ **(GE009_5)** Yuan
6. Household items and personal toiletries that are used daily plus beauty treatments (e.g., detergent, soap, toothpaste, toothbrush, cosmetics, beauty salon, etc.) ( ) ____ **(GE009_6)** Yuan
7. Entertainment (including fees to buy books, newspapers, VCCs, DVDs, going to cinema and bars) ( ) ____ **(GE009_7)** Yuan

GE010 In the last year how much did your household spend on the following items? .

[IWER: fill in 0 if no corresponding expenditure; fill in -9999 if the respondent cannot recall the expenditure. : 0 -9999 .] [soft check: >=100,000 yuan]

1. Clothing and bedding ____ (**GE010_1**) Yuan
2. Long distance traveling expenses ____ (**GE010_2**) Yuan
3. Heating(centrally heated) ()____ (**GE010_3**) Yuan
4. Furniture, consumption of durable goods and electronics, includes refrigerator, washing machine, TV, computers and expensive instruments like pianio. () _ (**GE010_4**) Yuan
5. Education and training(including tuition, training fees, etc.) ( ) ____ (**GE010_5**) Yuan
6. Medical expenditure (. .) ____ (**GE010_6**) Yuan
7. Fitness expenditures () ____ (**GE010_7**) Yuan
8. Beauty (including make-ups, facials, massages, etc.) ( ) ____ (**GE010_8**) Yuan
9. Automobiles ____ (**GE010_9**) Yuan
10. Purchase, Maintenance and repair (of transportation vehicles, appliances, communication products, etc.) ( ) () ____ (**GE010_10**) Yuan
11. Property management fees (including parking fee) () ____ (**GE010_11**) Yuan
12. Taxes and fees turned over to the government ( ) ____ (**GE010_12**) Yuan
13. Donations to the society (including cash, and items like food, clothing, etc.) ( ) ____ (**GE010_13**) Yuan

GE011 How often did the respondent receive assistance in answering section Household income and expenditure?

[IWER: If it is answered by a proxy, please record the respondents reaction. .]

1. Never
2. A few times
3. Most or all of the time

HA HOUSEHOLD ASSETS

[IWER: This section is asked of the family respondent. Do not allow a proxy respondent to answer the entire section. .]

PART 1 Current Residence

The following questions pertain to your current residence. .

HA000_W2_0

1.
2. / → skip to HA026_W2 Branchpoint HA026_W2 Branchpoint
3. → skip to HA026_W2 Branchpoint HA026_W2 Branchpoint
4. → skip to HA026_W2 Branchpoint HA026_W2 Branchpoint 5. → skip to HA026_W2 Branchpoint HA026_W2 Branchpoint

HA000_W2_1 BRANCHPOINT :
IF THIS IS A NEW INTERVIEWED HOUSEHOLD, GO TO HA001 HA001.
IF THIS IS A REINTERVIEWED HOUSEHOLD, GO TO HA000_W2_1 HA000_W2_1.

HA000_W2_1 [ZIWTime] [ ]

1. Yes → skip to HA001_W2 HA001_W2
2. No

HA001 When did your household start to live at your current residence? ____1900...2015 Year
[IWER: Mark the year using four digits. : 4 .]

HA001_W2 What is the construction area of the house? ____ m^2 [soft check<10 or >500]

HA002 Do you pay rent for your current residence? 1. Yes
2. No → skip to HA005 HA005

HA003 How much rent do you pay each month? _____ Yuan/Month / [soft check: <100, >10,000 yuan]

HA006 How much of the rent was paid by a housing subsidy from the employer of a household member? which household member? _____ (**HA006_1**)yuan [soft check >10,000 yuan] ____ (**HA006_2**) [preloaded list]

HA004 Did you pay less than the market rental value? 1. Yes
2. No → skip to HA007 HA007

HA005 If you rented the same housing unit from the market, what is the rent per month you would have to pay? ____ Yuan/Month / [soft check: <100, >10,000 yuan]

HA007 Who owns your current residence?

1.
2.
3. → skip to HA010 HA010

HA007_W2_1 [CAPI: Ask this question only when the earlier records show that ONE of the respondent (main respond OR the spouse passed away in the last two years] Has the owndership status changes following the death of [preload the name of the deceased main respondent or spouse] []

1. Yes
2. No → skip to HA008 HA008

HA007_W2_2 [] __ %.

HA007_W2_3 [] ?

[. 60 5050 .]

[Hard check: 100]

1. ___ (**HA007_w2_3_1[1]**)%
2. ___ (**HA007_w2_3_1[2]**)%
3. ___ (**HA007_w2_3_1[3]**)%
4. ___ (**HA007_w2_3_1[4]**)%
5. / ___ (**HA007_w2_3_1[5]**)%
6. ___ (**HA007_w2_3_1[6]**)%
7. ___ (**HA007_w2_3_1[7]**)%
8. ___ (**HA007_w2_3_1[8]**)%

PROCEDURE :
（HA007_W2_3 = 2） HA007_W2_4 HA007_W2_5

HA007_W2_4 [Load proxy].

HA007_W2_5 [HA007_W2_4] [ ] ___ %?

PROCEDURE :
（HA007_W2_3 = 5） HA007_W2_6 HA007_W2_7

HA007_W2_6 [Load proxy].

HA007_W2_7 [HA007_W2_6] [ ] __ %

HA008 Which household member(s) own the house? (preloaded names of household members) / ____ []

HA009 What share of the house is owned by [preloaded names of household members]? (HA008) ____ 0.00...100.00 % [hard check: range 0-100]

**PROCEDURE** :<br>Skip to HA011. HA011

HA010 Which non-household members own all or part of your current residence? (circle all that apply) ()

1. Working unit of household member, which household member? ____ (**HA010_1**) [preloaded list] [] 2. Government indemnificatory housing
3. Child(non- household member) of main respondent or spouse ( ) , which child? (**HA010_2**) [preloaded list] [ ]
4. Parent(non- household member) of main respondent or spouse ( ) ____ (**HA010_3**) [preload list] []
5. Nonresident other relatives
6. Friends
7. Other

**PROCEDURE** :<br>If HA007 = 3, skip HA011 and HA012. HA011HA012.

HA011 What is the present market value of your house? Or, what is the present market value of a similar housing unit within its neighborhood?

Total price ____ (**HA011_1**) 10000 Yuan [soft check < 10, > 500] Or unit price ____ (**HA011_2**) 1000 Yuan/m^2 /
[IWER: Skip to HA013 if R answered HA011. If not, ask unfolding brackets. HA011 HA013.]

HA012 [IWER: If R is unwilling to answer or does not remember, ask unfolding bracket questions here.]
20,000/50,000/100,000/200,000/500,000 Yuan

**HA026_W2 BRANCHPOINT**:<br>HA027.<br>HA000_W2_1 = 1 PART 2.

[. (HA026_W2_ 1[25]-HA026_W2_ 14_bracket[25]) (HA026_W2_1-HA026_W2_ 14_bracket)]

HA026_W2_1 []

1. →
2. Empty →
3. Sold
4. Rented out →
5. Being torn down → skip to HA026_W2_6 HA026_W2_6
6. My child(ren) stay in it → skip to HA026_W2_9 HA026_W2_9
7. My parent(s) stay in it → skip to HA026_W2_9 HA026_W2_9
8. Others (relatives, friends) stay in it () → skip to HA026_W2_9 HA026_W2_9
9. Give to others as gift → skip to HA026_W2_12 HA026_W2_12
10. Last residence is rented, no longer rent it →
11. Other, please specify ____ (**HA026_W2_1a**) →
12. Last record is wrong, the mentioned residence is not ours →

HA026_W2_2 When did you sell that house? _____ Year __ Month ____ (**HA026_W2_2_1**) __ (**HA026_W2_2_2**)

HA026_W2_3 What is the net income you got from selling the house, excluding relative fees? Total price ____ 10,000 Yuan [soft check < 0:1, > 500]

HA026_W2_4 [IWER: IfR is unwilling to answer or does not remember, ask unfolding bracket questions here.]

20,000/50,000/100,000/200,000/500,000 yuan

HA026_W2_5 How did you distribute the income from selling this house? (Can be multiple choice, ask the respondent to fill in corresponding percentages.) []

1. Deposits in bank account ___ (**HA026_W2_5a**) % of total income
2. Everyday living expenses ___ (**HA026_W2_5b**) % of total income
3. Buying new houses ___ (**HA026_W2_5c**) % of total income
4. Medical expenditure ___ (**HA026_W2_5d**) % of total income
5. Other important expenses ___ (**HA026_W2_5e**) % of total income
6. Trasfer to Children ___ (**HA026_W2_5f**) % of total income

7. Transfer to parents ___ (**HA026_W2_5g**) % of total income
8. Transfer to relatives ___ (**HA026_W2_5h**) % of total income
9. Other, please specify ___ (**HA026_W2_5i**) ___ (**HA026_W2_5j**) % of total income

[CAPI:]

HA026_W2_6 Did you receive any compensation for tearing down the house?
1. Yes
2. No →

HA026_W2_7 When did you receive the compensation for tearing down the house?
____Year (**HA026_W2_7_1**) __ Month (**HA026_W2_7_2**)

HA026_W2_8 What is the total amount of the compensation? ____ 10,000 Yuan

[CAPI:]

HA026_W2_9 Have those who live in your house pay you any cash, or provide you in-kind payment?
1. Yes
2. No →

HA026_W2_10 How did those who live in your house pay you the cash or the in-kind payment?
1. One lump-sum payment
2. Monthly payment
3. Irregular payment over the year

HA026_W2_11 What is the total amount did those who live in your house pay you in the past year? ____ (**HA026_W2_11a**) Yuan Among it, cash is ____ (**HA026_W2_11b**) Yuan in-kind payment is ____ (**HA026_W2_11c**) Yuan

[CAPI:]

HA026_W2_12 Have those who got your house as gifts pay you any cash, or provide you in-kind payment?
1. Yes
2. No →

HA026_W2_13 How did those who got your house as gifts pay you the cash or the in-kind payment?

1. One lump-sum payment
2. Monthly payment
3. Irregular payment over the year

HA026_W2_14 What is the total amount did those who got your house as a gift pay you in the past year? ____ (**HA026_W2_14a**) Yuan Among it, cash is ____ (**HA026_W2_14b**) Yuan in-kind payment is ____ (**HA026_W2_14c**) Yuan

[CAPI:]

**PROCEDURE** :<br>HA027 - HA028.

HA027 Excluding the house in which you live, do you or members of your household own any other residential properties? / (/)

1. Yes
2. No → skip to HA052 HA052

HA028 How many other housing units do you or members of your household currently own? / __ 0...10

PART 2 Other Residences

[For every other housing unit owned by main respondents and his/her spouse, ask the following questions,]

**HA029_W2 BRANCHPOINT**:<br>IF THE HOUSEHOLD IS A NEW INTERVIEW HOUSEHOLD, THEN SKIP TO HA029.<br>HA029<br>IF THE HOUSEHOLD IS A RE-INTERVIEW HOUSEHOLD, SKIP TO HA029_W2.<br>HA029_W2

HA029_W2 In the past two years since [preload the year of last wave] Year [preload the month of last wave] month, have your household obtained (purchased/built/inherited/received as gift/got new house due to tearing down) any new houses? [ZIWTime] (////) ( )

1. Yes, obtained __ 0...10 (**HA029_W2_1**) houses
2. No → skip to HA051_W2_1 Branchpoint HA051_W2_1 Branchpoint

[CAPIHA029-HA051]

HA030 Who owns this residence?

1. Owned completely by you and your spouse.
2. Owned partly by you and your spouse.

HA030_W2_1 [CAPI: Ask this question only when the earlier records show that one of the respondent (main respond or the spouse passed away in the last two years .] Has the owndership status changes following the death of [preload the name of the deceased main respondent or spouse]? []

1. Yes
2. No → skip to HA031 HA031

HA030_W2_2 [] __ %.

HA030_W2_3 [] ? [. 60% 50% 50% . 50%, 50%.]

[Hard check: 100]

1. ___ **(HA030_w2_3_1[1])**%
2. ___ **(HA030_w2_3_1[2])**%
3. ___ **(HA030_w2_3_1[3])**%
4. ___ **(HA030_w2_3_1[4])**%
5. / ___ **(HA030_w2_3_1[5])**%
6. ___ **(HA030_w2_3_1[6])**%
7. ___ **(HA030_w2_3_1[7])**%
8. ___ **(HA030_w2_3_1[8])**%

PROCEDURE :
（HA030_W2_3 =2） HA030_W2_4 HA030_W2_5

HA030_W2_4 [Load proxy]

HA030_W2_5 [HA030_W2_4] [ ] __ % ?

PROCEDURE :
（HA030_W2_3 =5） HA030_W2_6 HA030_W2_7

HA030_W2_6 [Load proxy].

HA030_W2_7 [HA030_W2_6] [ ] __ % ?

HA031 Who owns the house? [preloaded names of main respondents and his/her spouse] []

HA032 What share of the house is owned by [preloaded names of main respondents and his/her spouse]? [HA031] _____ %0.00...100.00% [hard check: range 0-100]

HA034 What is the present market value of your house? Or, what is the present market value of a similar housing unit within its neighborhood? Total price ____ (**HA034_1**) 10,000 Yuan [soft check <10, >500] or unit price ____ (**HA034_2**) 1,000Yuan/m^2 / [soft check<1, >25]

[CAPI: Skip to HA036 if R answered HA034. If not, ask unfolding brackets. HA034 HA036]

HA035 [IWER: If R is unwilling to answer or does not remember, ask unfolding bracket questions here.]

20,000/50,000/100,000/200,000/500,000 Yuan

HA039 How was this housing unit obtained?

1. Purchased from market
2. Purchased from working unit of respondent or spouse ____ (**HA039_1**)[preloaded list] []
3. Purchased by child of main respondent or spouse, which one? ( ) ____ (**HA039_2**) [preload list] []
4. Purchased by parents of main respondent or spouse, of who (main respondent or spouse) ? () () ____ (**HA039_3**)
5. Purchased from Other relatives
6. Self-built → Skip to HA041 HA041
7. Inherited, bequeathed, or given () → Skip to HA041 HA041
8. Received home as compensation for demolition of old home, → Skip to HA041 HA041
9. Other → Skip to HA045 HA045

HA040 Was it purchased at market price, subsidized by working unit, or as purchased as economical housing?

1. Market price
2. Subsidized by working unit
3. Economic housing
4. Other

HA041 When did you purchase/build/inherit it? //// ____ 1900...2015 Year
[IWER: Mark the year using four digits. : 4 .]

HA042 Can you sell the house freely if you want? 1. Yes
2. No, restricted by work unit

**PROCEDURE** :<br>If HA039 = 7 , then skip to HA045. HA039 = 7 HA045.

HA043 How much of your own money did you spend on the house (including loan-financed)?
// ____ 10,000Yuan [soft check<10, >500]
[IWER: Skip to HA045 if R answered HA043. If not, ask unfolding brackets. HA043 HA045.]

HA044 [IWER: If R is unwilling to answer or does not remember, ask unfolding bracket questions here.]
10,000/20,000/50,000/100,000/200,000 Yuan

**PROCEDURE** :<br>If HA040 = 1, then skip to HA051. HA040 = 1 HA051. If HA039 = 6, then skip to HA051. HA039 = 6 HA051.

HA045 What would you have to pay if you had paid a market-set price for the same housing? Total price ____ (**HA045_1**) 10,000 Yuan [soft check >500] or unit price ____ (**HA045_2**) 1,000 Yuan/m^2 / [soft check >30]

HA051 What is the construction area of the house? ____m^2 [soft check<10 or >500]

[Skip to next house until the last house]

**HA051_W2_1 BRANCHPOINT**:<br>If the household is a new interview household, skip to HA052 HA052

[CAPI HA026_W2_1-HA026_W2_14_bracket (1). HA052.]
[.]

HA026_W2_1 [] 1. →

2. Empty →
3. Sold
4. Rented out →
5. Being torn down → skip to HA026_W2_6 HA026_W2_6
6. My child(ren) stay in it → skip to HA026_W2_9 HA026_W2_9
7. My parent(s) stay in it → skip to HA026_W2_9 HA026_W2_9
8. Others (relatives, friends) stay in it () → skip to HA026_W2_9 HA026_W2_9
9. Give to others as gift → skip to HA026_W2_12 HA026_W2_12
10. Last residence is rented, no longer rent it →
11. Other, please specify ____ (**HA026_W2_1a**) →
12. Last record is wrong, the mentioned residence is not ours →
13. The house talked just now →

HA052 What is the monthly rental income for all room or houses owned by main respondent or spouse, that you are currently leasing? ()

1. ____ (**HA052_1**) Yuan/Month / [soft check >20,000]
2. Not applicable

HA054_W3 Do you or other household members take out a bank loan to finance the purchase of your and your spouses houses now? ()

1. Yes
2. No skip to HA054 HA054

HA055_W3 What is the outstanding amount of the loans? ____10,000Yuan

HA056_W3 How much is the unpaid interest? ____10,000 Yuan

HA057_W3 What is the monthly mortgage payment? ____10,000 Yuan [soft check >20,000]

PART 3 Land

[The following questions pertain to your land.]

HA054 Does your and your spouse have any collective distributing or rent cultivated land, forest land, pasture and/or pond? (Choose all that apply) ()

1. Cultivated land
2. Forest land
3. Pasture
4. Pond
5. None → Skip to HA064 HA064

PROCEDURE :
According to all options choosed in HA054 , ask HA055 -HA063 in loop. HA054 HA055 -HA063 .

HA055 How many mu of [preload answer fromHA054] do you and your spouse have? [HA054] [ ] ____ Mu [soft check > 50]

HA056 How many mu of them are irrigable? ____Mu [hard check cannot be > HA055]

HA057 What is the rent per mu per year you would get if you rent out all your [preload answer from HA054]? [HA054] ____ Yuan per mu per year [soft check < 10; > 4000]

HA058 Did you rent out any of your [preload answer from HA054] in the past year? [HA054]
1. Yes
2. No → Skip to HA061 HA061

HA059 How much [preload answer from HA054] did you rent out the past year? [HA054]____ Mu [hard check, cannot be > HA055]

HA060 How much rental income did you earn in the past year? [HA054] ____ Yuan

HA061 Did you rent in any [preload answer from HA054] from others (including the collective) in the past year? ( ) [HA054]
1. Yes
2. No → Skip toHA064 HA064

HA062 How much did you rent in the past year? [HA054]____ Mu [soft check > 100]

HA063 How much rent did you pay in the past year? [HA054] ____ Yuan [soft check >20,000]

HA064 How much rental income did you earn for any other household assets other than housing or land? (trees, use of fixed capital, durables, or livestock)? ()

1. ____ (**HA064_1**) Yuan
2. Not applicable

PART 4 Equipments, Consumption durables, and Valuables.

HA065 Do you and your spouse own the following assets? (Choose all that apply) ()

For all categories, add [soft check <100 or >30,000] unless other check is written

[For each asset owned by main respondent and his/her spouse] what is the assets current value? (Yuan) ()

1. Automobile [soft check <3000, >500,000] _____ (**HA065_1[1]**)
2. Electric Bicycle ____ (**HA065_1[2]**)
3. Motorcycle ____ (**HA065_1[3]**)
4. Refrigerator ____ (**HA065_1[4]**)
5. Washing machine ____ (**HA065_1[5]**)
6. TV ____ (**HA065_1[6]**)
7. Computer ____ (**HA065_1[7]**)
8. Stereo system ____ (**HA065_1[8]**)
9. Video camera ____ (**HA065_1[9]**)
10. Camera ____ (**HA065_1[10]**)
11. Air conditioner ____ (**HA065_1[11]**)
12. Mobile phone ____ (**HA065_1[12]**)
13. Furniture ____ (**HA065_1[13]**)
14. Music instrument ____ (**HA065_1[14]**)
15. Valuable decorations, ornaments ____ (**HA065_1[15]**)
16. Treasures and precious metal (such as gold) () __________ (**HA065_1[16]**)
17. Antiques, valuable paintings and calligraphic work, and other artistic work ____ (**HA065_1[17]**)
18. None

HA066 Do you and your spouse own the following fixed capital assets? How much are the assets worth? (check all that apply)) ( )

1. Tractor, current value_____ Yuan ____ (**HA066_1_1**) [soft check < 1000; > 30; 000]

2. Thresher, current value_____ Yuan () ____ (**HA066_1_2**) [soft check < 100 or > 10; 000]
3. Tractor tools, current value_____ Yuan ____ (**HA066_1_3**) [soft check < 100 or > 10; 000]
4. Water pump, current value_____ Yuan () ____ (**HA066_1_4**) [soft check < 100 or > 10; 000]
5. Processing equipment, current value_____ Yuan ____ (**HA066_1_5**) [soft check < 100 or > 10; 000]
6. None

HA067 What is the current value of other fixed capital assets used in household production or self-employed activities?) ____ Yuan

[IWER: Be sure to ask about fixed capital assets used in all self-employment activities, do not count assets already reported above. .]

HA068 Do you and your spouse have any other durable or fixed assets worth 500 yuan or more?) 500

1. Yes How much are the assets worth? ____ (**HA068_1**) Yuan [hard check > 500] [soft check > 50; 000]
2. No

HA076 How often did the respondent receive assistance in answering section Household assets?

[IWER: If it is answered by a proxy, please record the respondents reaction. .]

1. Never
2. A few times
3. Most or all of the time

HB INDIVIDUAL ASSETS

[IWER: Please conduct sections HB and HC when the main respondent and his/her spouse are at home. Dont allow a proxy to complete the entire sections. HB HC . .]

PART 1 Financial Assets

[The following questions pertain to your financial asset. .]

[IWER reminder: make sure others are not present, IWER read following instructions: the following questions pertain to your financial asset, the answers to these questions will be

kept strictly confidential and will be used for research purposes only. .. .]

HC001 How much cash is held by you and your spouse at home? () _____ Yuan [soft check > 50; 000 or < 100]

[IWER: Skip to HC005 if R answered HC001 and HC001 ≠ 0. If not, ask unfolding brackets. HC001 0 HC005]

HC002 [IWER: If R is unwilling to answer or does not remember, ask unfolding bracket questions here.] 500 /1,000 /2,000 /5,000 /10,000 Yuan

[CAPI: prompt for HC004 - HC019: IWER: for deposit, bondsstocks, and funds, only include assets legally in his/her name. HC004 - HC019]

HC005 What is the total amount of deposits you are currently holding in financial institutions (eg: bank) ? () ______ Yuan [soft check > 500; 000 or < 100]

PROCEDURE :

Skip to HC007 if R answered HC005. If not, ask unfolding brackets. HC005 HC007.]

HC006 [IWER: If R is unwilling to answer or does not remember, ask unfolding bracket questions here.]

2,000/10,000/50,000/100,000/500,000 Yuan

HC007 Do you have any government bonds (e.g. Treasury bills) in your name? ( ) ()

1. Yes
2. No → Skip to HC010 HC010

HC008 What is the total face value of government bonds that you are currently holding? () ?_____ Yuan [soft check > 50; 000]

PROCEDURE :

Skip to HC0010 if R answered HC008. If not, ask unfolding brackets HC009. HC008 HC0010HC009.

HC009 [IWER: If R is unwilling to answer or does not remember, ask unfolding bracket questions here.] 10,000 /50,000 /100,000 /200,000 /500,000 Yuan

HC010 Have you held any stocks in the past year in your name, excluding the equity or stock of your work unit? ( )

1. Yes
2. No → Skip to HC015 HC015

HC013 What is the present market value of all the stocks you are currently holding? __ Yuan [soft check > 200; 000]

**PROCEDURE** :<br>Skip to HC015 if R answered HC013. If not, ask unfolding brackets. HC013 HC015.

HC014 [IWER: If R is unwilling to answer or does not remember, ask unfolding bracket questions here.] 10,000 /50,000 /100,000 /200,000 /500,000 Yuan

HC015 Have you held any funds in your name in the past year? ( )

1. Yes
2. No → Go to PROGRAM before HC020 HC020

HC018 What is the present market value of all the mutual funds you are currently holding? Yuan [soft check > 200; 000]
[IWER: Skip to HC020 if R answered HC018. If not, ask unfolding brackets.
HC018HC020 .]

HC019 [IWER: If R is unwilling to answer or does not remember, ask unfolding bracket questions here.] 10,000 /50,000 /100,000 /200,000 /500,000 Yuan

**PROCEDURE** :<br>if HC005 = 0 and HC007 = 2 and HC010 = 2 and HC015 = 2, then skip HC020.<br>HC005 = 0HC007HC010HC015 2 HC020.

HC020 What percentage of the deposits, bonds, stocks, and funds held in your name is fully controlled by you and not your spouse? (%) ____ 0...100 % [hard check ≥ 0, ≤ 100]

HC021 Do you have any other deposits, bonds, stocks, or funds that belong to you but which are held in a persons name other than you or your spouse?

1. Yes
2. No → Skip to HC027 HC027

HC022 What is the value of such assets? ______ yuan [soft check > 200; 000]

HC027 Do you have public housing funding?

1. Yes
2. No → Skip to HC030 HC030

HC028 What is the total amount of money in your public housing fund? ______ Yuan [soft check > 100; 000]

**PROCEDURE** :<br>Skip to HC030 if R answered HC028. If not, ask unfolding brackets HC029. HC028 HC030HC029.

HC029 [IWER: If R is unwilling to answer or does not remember, ask unfolding bracket questions here.]

5,000/10,000/50,000/100,000/200,000 Yuan

HC030 Do you have Jizikuan that your work unit or other work units have collected from you and are still holding (Jizikuan is fund individuals provided to the work unit for the purpose of investment, building apartments, etc.)?

1. Yes
2. No → Skip to HC033 HC033

HC031 What is the amount of your jizikuan? ______Y uan [soft check > 200; 000]

**PROCEDURE** :<br>Skip to HC033 if R answered HC031. If not, ask unfolding brackets HC032. HC031 HC033 HC032.

HC032 [IWER: If R is unwilling to answer or does not remember, ask unfolding bracket questions here.]

5,000/10,000/50,000/100,000/200,000 Yuan

HC033 Do you have any unpaid salary that your work unit still owes you?

1. Yes
2. No → Skip to HC036 HC036

HC034 What is the amount of your unpaid salary? ______ Yuan [soft check > 100; 000]

PROCEDURE:
Skip to HC036 if R answered HC034. If not, ask unfolding brackets HC035. HC034 HC036 HC035.

HC035 [IWER: IfR is unwilling to answer or does not remember, ask unfolding bracket questions here.] 5,000/10,000 /50,000 /100,000 /200,000 Yuan

HC036 Have you participated in any private lending during the past year? ()
1. Yes
2. No → Skip to HC039_W3 HC039_W3

HC036_W3 What is the total amount of funds that you are still obligated to private lending during the past year ______ Yuan [soft check > 100; 000]

PROCEDURE:
Skip to HC037 ifR answered HC036_W3. If not, ask unfolding brackets HC036_W3_1. HC036_W3 HC037 HC036_W3_1.

HC036_W3_1 [IWER: IfR is unwilling to answer or does not remember, ask unfolding bracket questions here.] 5,000/10,000 /50,000 /100,000 /200,000 Yuan

HC037 What is the total amount of funds that you are still obligated to pay to private lending ______ Yuan

PROCEDURE:
Skip to HC039_W3 ifR answered HC037. If not, ask unfolding brackets HC038_W3. HC037 HC039_W3 HC038_W3.

HC038_W3 [IWER: If R is unwilling to answer or does not remember, ask unfolding bracket questions here.]
5,000/10,000/50,000/100,000/200,000 Yuan

HC039_W3 Have you lent to other families or individuals and not been repaid by them?
1. Yes
2. No → Skip HC040_W3 and HC041_W3 HC040_W3 HC041_W3

HC040_W3 What is the total amount of the loans? ______ Yuan [soft check > 500; 000]

**PROCEDURE** :<br>Skip HC041_W3 if R answered HC040_W3. If not, ask unfolding brackets HC041_W3.<br>HC040_W3 HC041_W3 HC041_W3.

HC041_W3 [IWER: If R is unwilling to answer or does not remember, ask unfolding bracket questions here.]
5,000/10,000/50,000/100,000/200,000 Yuan

PART 2 DEBTS

[The following questions pertain to your debt. .]

HD001 What is the total amount of loan that you havent repaid yet (not including loans for house)? ( ) ______ Yuan [soft check > 500; 000]

**PROCEDURE** :<br>Skip to HD003 if R answered HD001. If not, ask unfolding brackets HD002. HD001 HD003 HD002.

HD002 [IWER: If R is unwilling to answer or does not remember, ask unfolding bracket questions here.]
5,000/10,000/50,000/100,000/500,000 Yuan

HD003 What is the amount of your credit card balance? _____ Yuan [soft check > 50; 000] (0)

**PROCEDURE** :<br>Skip to HD004_W3 if R answered HD003 . If not, ask unfolding brackets HD004.<br>HD003 HD004_W3 HD004.

HD004 [IWER: If R is unwilling to answer or does not remember, ask unfolding bracket questions here.]
500/1,000/5,000/10,000/50,000 Yuan

HD004_W3 ( ) ___ Yuan [soft check > 50; 000] (0)

**PROCEDURE** :<br>Skip HD004_W3_1, if R answered HD004_W3. If not, ask unfolding brackets HD004_W3_1. HD004_W3HD004_W3_1 HD004_W3_1.

HD004_W3_1 [IWER: If R is unwilling to answer or does not remember, ask unfolding bracket questions here.]

500/1,000/5,000/10,000/50,000 Yuan

HD005_W3

1. Yes
2. No → Skip HD005_W3_1 HD005_W3_1

HD005_W3_1 ______ Yuan

HD012 How often did the respondent receive assistance in answering section H ASSETS?

[IWER: If it is answered by a proxy, please record the respondents reaction. .]

1. Never
2. A few times
3. Most or all of the time

This page intentionally left blank

I HOUSING CHARACTERISTICS

PROCEDURE :
Only main respondent answer I002 - I026. I002 - I026

I002 What is the total housing land area? ? ( ) _____ m^2 [soft check < 10, > 1000]
[0]

I003 Is your residence used for business as well? 1. Yes
2. No

I004 What type of structure is this building?
1. Concrete and steel/ Bricks and wood
2. Adobe /
3. Wood/ Thatched /
4. Cave dwelling
5. Mongolian yurt/Woolen felt/Tent //
6. Stone
7. Other, please specify _____ (**I004_1**)

I005 When was this house built? _____ Year (**I005_1**)
If R is unclear about year, please choose among following items.
1. 0 – 5 years 0 – 5
2. 5 – 10 years 5 – 10
3. 10 – 20 years 10 – 20
4. 20 – 30 years 20 – 30
5. 30 – 40 years 30 – 40
6. More than 40 years 40

I006 Is the building one story or multi-level building? 1. One-story building → skip to I007 I007
2. Common multi-story building → skip to I008 I008
3. Self-contained multi-story building → skip to I009 I009

I007 Is the story independent or compound?
1. Independent story
2. Compound

I008 Which story is this building on? ______ Storey

**PROCEDURE** :<br>If I008 > 1, ask I009. I008 > 1I009

I009 Does it has elevator

1. Yes
2. No

I010 Are there any handicapped facilities (e.g., non-stair ramp)? ( )

1. Yes
2. No

**PROCEDURE** :<br>If I010 = 2, ask I011. I010 = 2I011

I011 How many steps had to be climbed to get to the main entrance of the households flat? [IWER: Do not count steps if an elevator is available]

1. 0 0
2. 1 to 5 1 – 5
3. 6 to 15 6 – 15
4. 16 to 25 16 – 25
5. More than 25 25

I012 How many bedrooms, living rooms, bathrooms, and kitchens are there in your residence? ______ (**I012_1**) bedrooms ______ (**I012_2**) living rooms ____ (**I012_3**) toilets ( ) ______ (**I012_4**) kitchens ______ (**I012_5**) balcony [soft check: > 20]

**PROCEDURE** :<br>Ask I013 if no toilets in the answer to I012. I012I013

I013 How far is the nearest toilet to your house? _____ (**I013**) Meters [soft check: > 500]

I014 What is the type of toilet?

1. Toilet without a seat
2. Toilet with a seat → skip to I016 I016

I015 Is the toilet flushable?

1. Yes
2. No

I016 Does your residence have electricity?

1. Yes
2. No

I017 Does your residence have running water?

1. Yes
2. No

I018 Is there in-house shower or bath facility? What type? 1. Hot water provided

2. Water heater installed by the household
3. No

I019 Does your residence have coal gas or natural gas supply?

1. Yes
2. No

I020 Does your residence have heating? ( )

1. Yes
2. No → skip to I022 I022

I021 What is the main heating energy source? 1. Solar

2. Coal
3. Natural gas
4. Liquefied Petroleum Gas
5. Electric
6. Crop residue/Wood burning
7. Other, please specify _____ (**I021_1**)

I022 What is the main source of cooking fuel?

1. Coal
2. Natural gas
3. Marsh gas
4. Liquefied Petroleum Gas
5. Electric
6. Crop residue/Wood burning
7. Other, please specify _____ (**I022_1**)

I023 Does your residence have a telephone connection?

1. Yes
2. No

I024 Does your residence have broad-band internet connection?

1. Yes
2. No

I027_W3 Does your residence have an air cleaner in your home?

1. Yes
2. No

I025 [Interviewer records it] How clear and tidy is in this household

1. Excellent
2. Very clear
3. Clear
4. Fair
5. Poor
6. Not applicable

I026 [Interviewer records it] How is the temperature in this household

1. Very hot
2. Hot
3. Bearable
4. Cold
5. Very cold
6. Not applicable
