# Supplementary material for: Association of total sleep duration variability with risk of new stroke in the middle-aged and elderly Chinese population
Source: BMC Neurol. 2024 Jun 25;24:217. doi: 10.1186/s12883-024-03727-8 (PMC11197293; doi:10.1186/s12883-024-03727-8)
Supplement: Supplementary file 4 — Supplementary Material 4 [file 12883_2024_3727_MOESM4_ESM.pdf]

CHINA HEALTH AND RETIREMENT LONGITUDINAL STUDY
WAVE 4 (2018) QUESTIONNAIRE

2018

VERSIONID: 20200914

NATIONAL SCHOOL OF DEVELOPMENT
INSTITUTE OF SOCIAL SCIENCE SURVEY
PEKING UNIVERSITY

This page intentionally left blank

Contents

CV Cover Screen 1

B Demographic Backgrounds 5

C Family 19

C1 Parent, Children and Sibling Information . 20

CA Parent Information 20

CB Children Information 30

CC Sibling Information 41

A Household Member Information 47

C2 Time Transfer and Transfers 49

CD Time Transfer 49

CE Transfers 53

CF Time Spent Providing Care 59

D Health Status and Functioning 63

DA Health Status 64

PART I General Health Status and Disease History 64

PART II Lifestyle and Health Behaviors 81

DB Functional Limitations and Helpers 89

DC Cognition and Depression 101

SECTION INTRO Introducing Respondent Interview 101

SECTION MMSE Mini Mental State Exam 101

SECTION HT HRS Telephone Interview for Cognitive Status (TICS) 107

SECTION WR Word Recall 108

SECTION RF Retrieval Fluency 111

SECTION CSI-D Community Screening Instrument for Dementia Interviewee Part - 114

SECTION CESD Depression 114

SECTION SAT Satisfaction 117

SECTION DR Delayed Recall 118

SECTION NS Number Series 119
SECTION WRE Wordlist Recognition 125
END_DC Interview Observation DC 129

DD Informants Information 131
SECTION INTRO IF Introducing Informant Interview 133
SECTION DEMOGRAPHICS_IF Demographics of Informant 134
SECTION JORM IQCODE The Jorm Informant Quesionnaire on Cognitive Decline in the Elderly 135
SECTION BLESSED Blessed Dementia Scale Part II Blessed 143
SECTION CSI-D Community Screening Instrument for Dementia Informant Part 145
END_DD Interview Observation DD 146

E Health Care and Insurance 149
PART I Medical Insurance 149
PART II Health Care Costs and Utilization 152

F Work and Retirement 165
FA Work Status 167
Update Missing Work Status in the Last Visit . 173
Job Switch 175
FC Self-Employed Agricultural Work 182
FD Employed 183
FE Questions About Labor Supply 190
FF Questions About Wages 191
FG Fringe Benefits 194
FH Non-Farm Self-Employed and Unpaid Work for Family Business 195
FJ Side Job (Employed or Self-employed) ( ) 199
FK Unemployment and Job Search Activities 200
FL Last Job 201
FM Retirement 206

FN Pension 217

PART 1 Pension for Public Servants, Public Institution Employees, and Basic Pension for Enterprise Employees （ ） ... 217
PART 2 Supplementary Pension Insurance (Annuity) （ ） 222
PART 3 Urban and Rural Resident Pension, New Rural Resident Pension and Urban Resident Pension ... 226
PART 4 Pension for Land-Expropriated Farmers （ /） ... 231
PART 5 Life Insurance ... 233
PART 6 Commercial Pension Insurance（Exclude Life Insurance） （） ... 235
PART 7 Other Pension ... 238

G&H Income, Expenditures and Assets 245

G2 Household Income and Expenditures ... 245
PART 1 Household Wage Income and Individual-based Transfers ... 245
PART 2 Household Agricultural Income and Expenditure ... 253
PART 3 Self-Employed Activities ... 257
PART 4 Household Public Transfer Income ... 258
PART 5 Household Living Expenditure ... 260
HA Household Assets ... 263
PART 1 Land ... 263
PART 2 Equipments, Consumption Durables, and Valuables ... 266
HB Individual Assets ... 268
PART 1 Financial Assets ... 268
PART 2 Debts ... 272

HA&I House Property and Housing Characteristics 275

HA House Property ... 275
I Housing Characteristics ... 291

This page intentionally left blank

CV Cover Screen

CV_ HType CAPI preloads type of respondent household CAPI

1. Two-person household previously interviewed → CAPI generates XHType = 1 CAPI XHType = 1
2. One-person household previously interviewed → CAPI generates XHType = 1 CAPI XHType = 1
3. New household → CAPI generates XHType = 2 CAPI XHType = 2

[IWER: Respondent for cover screen must be [Name of Respondent 1] or [Name of Respondent 2]. In which case both respondents had died or can not answer questions due to deaf, mutism, mental illness, or dementia, another family member can answer [1] [2]]

CV001 Of people on the following list, who is answering the cover screen?

1. [Name of Respondent 1][1]
2. [Name of Respondent 2][2]
3. Informant name ______ (**CV001_1**)relationship with [Name of respon- dent 1] [1] __ (**CV001_2**)

CV002 Is [Name of Respondent 1] still alive? [1]

[IWER: If [Name of Respondent 1] is who answers the cover screen, then do not ask, choose alive directly [1]]

1. Alive
2. Died Date of death was ______ (**CV002_1**) Year ___ (**CV002_2**) Month ___ (**CV002_3**) Day

**PROCEDURE** :<br>If CV_HType ≠ 1, Skip CV003 CV_HType ≠ 1 CV003

CV003 Is [Name of Respondent 2] still alive? [2]

[IWER: If [Name of Respondent 2] is who answers the cover screen, then do not ask, choose alive directly [2]]

1. Alive
2. Died Date of death was ______ (**CV003_1**) Year ___ (**CV003_2**) Month ___ (**CV003_3**) Date

PROCEDURE :

If CV_HType = 1, and CV002 = 1, and CV003 = 1, ask CV004 to CAPI Split Household CV_HType = 1 CV002 = 1 CV003 = 1 CV004 CAPI

If CV_HType = 1, and CV002 = 1, and CV003 = 2, Save [Name of Respondent 1] as [Main Respondent], Save XRType of [Main Respondent] as 1.REIW, Generates Exit and Death Cause Questionair for [Name of Respondent 2] and skip to CV006 then CV_HType = 1 CV002 = 1 CV003 = 2 [1] [][ ] XRType 1.REIW [2] CV006 If CV_HType = 1, and CV002 = 2, and CV003 = 1, Save [Name of Respondent 2] as [Main Respondent], Save XRType of [Main Respondent] as 1. REIW, Generate Exit and Death Cause Questionaire for [Name of Respondent 1] and skip to CV006 then CV_HType = 1 CV002 = 2 CV003 = 1 [2] [][ ] XRType 1. REIW [1] CV006 If CV_HType = 1, and CV002 = 2, and CV003 = 2, Generate Exit and Death Cause Question- air for [Name of Respondent 1] and [Name of Respondent 2], and end Cover Screen then CV_HType = 1 CV002 = 2 CV003 = 2 [1] [2]

If CV_HType = 2, and CV002 = 1, Save [Name of Respondent 2] as [Main Respondent], SaveXRType of [Main Respondent] as 1. REIW, and skip to CV006 CV_HType = 2 CV002 = 1 [1] [][] XRTyp 1. REIW CV006

If CV_HType = 2, and CV002 = 2, Generate Exit and Death Cause Questionaire for [Name of Respondent 1], and end Cover Screen then CV_HType = 2 CV002 = 2 [1]

If CV_HType = 3, and CV002 = 1, Save [Name of Respondent 2] as [Main Respondent], and skip to HH_MRBirth1 then CV_HType = 3 CV002 = 1 [1] [ ] HH_MRBirth1

If CV_HType = 3, and CV002 = 2, end the interview CV_HType = 3 CV002 = 2

CV004 Are [Name of Respondent 1] and [Name of Respondent 2] still in a marital relationship or living together as married? [1] [2]

1. [Name of Respondent 1] and [Name of Respondent 2] are still in marital relationship or living together as married [1] [2] → CAPI save [Name of Respondent 1] as [Main Respondent], XRType of [Main Respondent] as 1. REIW, Save [Name of Respondent 2] as [MRs spouse],XRType of [Main Respondent] as 1. REIW, skip to CAPI generates personal questionaire CAPI [1] [][] XRType 1. REIW [2] [][] XRType 1. REIWCAPI
2. [Name of Respondent 1] and [Name of Respondent 2] have divorced [1]

[2]

3. [Name of Respondent 1] and [Name of Respondent 2] is living apart for a while, would not live as a couple in a foreseeable future [1] [2]

PROCEDURE：
If CV001 = 3, ask CV005 CV001 = 3 CV005

CV005 You are more familiar with [Name of Respondent 1] or [Name of Respondent 2]? [1] [2]

1. [Name of Respondent 1] [1]
2. [Name of Respondent 2] [2]

CAPI Split Household CAPI Split the divorced household into two households

Respondent of the Cover Screen（if CV001 ≠ 3）or CV005（if CV001 = 3）take the original household ID, save the name of the respondent into [Main Respondent name], save the XRType of [Main Respondent name] as 1. REIW. Save the name of the other respondent as [Name of Divorced Spouse], and skip to CV006 （CV001 ≠ 3） CV005 （CV001= 3） ID [][ ] XRType 1. REIW [] CV006

Generate a new household as a one-person household previously interviewed for this divorced respondent

HH_MRBirth1 According to the information of previously interviewed, the birthdate of the [Main Respondent] is [pre-loaded Birthdate of Respondent] [] []

1. Right → Skip HH_MRBirth2 HH_MRBirth2
2. Not Right

HH_MRBirth2 The birthdate of [Main Respondent name] is [] ______ (**HH_MRBirth2_1**) Year ___ (**HH_MRBirth2_2**) Month ___ (**HH_MRBirth2_3**) Date

CAPI Determine the respondent is age-eligible or not CAPI

CAPI Based on [pre-loaded Birthdate of Respondent] and the refreshed HH_MRBirth2, determine the respondent is age-eligible or not. Standard is whether the birthday is early or equal to 1972/7/1 CAPI [] HH_MRBirth2 [] 1972 7 1

If eligible, Save the Xrtype of [Main Respondent name] as 2. NEWIW, continue on ask CV006 [] XRType 2. NEWIW CV006

If not eligible, end the interview

CV006 What is [preload MR name]s marital status? []

1. Married with spouse present → Skip to CV008 CV008
2. Married but not living with spouse temporarily for reasons such as work → Skip to CV008 CV008 3. Separated
4. Divorced
5. Widowed
6. Never married

CV007 For [preload MR name] who is single, or separate in marital status, does [preload MR] have any partner living together as a spouse? [] []

1. Yes Name ______ (**CV007_1**) → Skip to CAPI Generates Spouse CAPI
2. No → Skip to CAPI Generates Spouse CAPI

CV008 What is the current spouses name of [preload MR name]? [] _______

**CAPI Generates Spouse CAPI**
IfCV006 = 1, 2, SaveCV008as [MRs spouse name], Save the XRType of [MRs Spouse name] as 2.NEWIW CV006 = 1, 2 CV008 [][] XRType 2. NEWIW<br>If CV006 = 3, 4, 5, 6 and CV007 = 1, Save CV007_1 as [MRs spouse name], Save XRType of [MRs spouse name] as 2. NEWIW CV006 = 3, 4, 5, 6 CV007 = 1 CV007_1 [ ][ ] XRType 2. NEWIW<br>Otherwise, Save [MRs spouse] as Null [ ]

**CAPI Generates Personal Questionaire CAPI**
Generate Personal Questionaire for [preload MRs name], Generate MainR=1 for [preload MRs name] [] [] **MainR** = 1 If [MRs spouses name] is not Null, generate Personal Questionaire for [MRs spouses name] [] []

B Demographic Backgrounds

CAPI: Ask [Name of Main Respondent] and [Name of Main Respondents spouse] questions in this module. Write [Name of Respondent] in this questionnaire, unless ambiguity occurs [ ] [ ] []

PROCEDURE :

If this is a reinterview, XRType = 1 and [ZFRgender] = 1, ask BA000_W2_3, then renew XRGender = BA000_W2_3 XRType = 1 [ZFRgender] = 1 BA000_W2_3 XRGender BA000_W2_3

If this is a new interview, XRType = 2, ask BA000_W2_3, then renew XRGender = BA000_W2_3 XRType = 2 BA000_W2_3 XRGender BA000_W2_3

If not, XRGender=[ZRGender], then skip to the procedure before BA001 XRGen- der [ZRGender] BA001

BA000_W2_3 Interviewer record the Respondents gender 1. Male
2. Female

PROCEDURE :

If this is a reinterview, XRType = 1 and ZFRgender = 1, ask BA001 XRType = 1 [ZFRzodiac] = 1 BA001

If this is a new interview, XRType = 2, ask BA001 XRType = 2 BA001

If not, skip to the procedure before BA004_W3 BA004_W3

BA001 What is your Chinese Zodiac sign?

1. Rat
2. Ox
3. Tiger
4. Rabbit
5. Dragon
6. Snake
7. Horse
8. Goat
9. Monkey
10. Rooster
11. Dog
12. Pig

PROCEDURE :

If this is a reinterview, XRType = 1 and ZFRgender = = 1, ask BA004_W3 to BA003 XRType = 1 [ZFRbirth] = 1 BA004_W3 BA003

If this is a new interview, XRType = 2, ask BA004_W3 to BA003 XRType = 2 BA004_W3 BA003

If not, skip to the procedure before BB000_W3. BB000_W3

BA004_W3 Whats your date ofbirth on ID card or Household register?
1. _______ (**BA004_W3_1**) Year ___ (**BA004_W3_2**) Month ___ (**BA004_W3_3**) Day 2. Does not have Hukou or ID card → Skip to BA002BA002 3. Has Hukou or ID card, but refuses to answer the date ofbirth on ID card or Household register → Skip to BA002 BA002

[IWER: If the respondent doesnt remember the date of birth on ID card or Household register, remind him/her to find ID card or Household register to confirm the date. The year must be a number in the range [1900-2018]. Mark the year using four digits. Take down the month as its actual number. For example, write January as 1 not 01, December as 12 [1900-2018] 4 1 10112 12]

BA005_W4 Is your actual date ofbirth the same as on ID card or Household register?
1. Yes → Skip BA002 BA002
2. No

BA002 Whats your actual date ofbirth? ______ (**BA002_1**) Year ______ (**BA002_2**) Month ______ (**BA002_3**) Day

[IWER: The year must be a number in the range [1900-2018]. Mark the year using four digits. Take down the month as its actual number. For example, write January as 1 not 01, December as 12. If does not remember month and day, fill 0 [1900-2018] 4 1 101 12 120]

PROCEDURE :

CAPI generates [actual date ofbirth] CAPI []

If BA005_W4 = 1, then [actual date of birth] = BA004_W3_1 year BA004_W3_2 month BA004_W3_3 date BA005_W4 = 1 [] = BA004_W3_1 BA004_W3_2 BA004_W3_3

If BA005_W4 = 2 or BA004_W3 = 2, 3, then [actual date ofbirth]= BA002_1 year BA002_2 month BA002_3 date BA005_W4 = 2 BA004_W3 = 2, 3 [] = BA002_1 BA002_2 BA002_3

BA003 Is your previous answer based on the solar or the lunar calendar? [] ( ) ( )
1. Solar calendar ( )
2. Lunar calendar ( )

PROCEDURE :

If this is a reinterview, XRType = 1 and [ZRpaddress] is missing, ask BB000_W3, then generate an updated address for the last wave [XRLpaddress] = BB000_W3 XRType = 1 [ZRpaddress] BB000_W3 [XRLpaddress] = BB000_W3

If this is a reinterview, XRType = 1, and [ZRpaddress] is not missing, generate an updated address for the last wave [XRLpaddress] = BB000_W3, then skip to BB001_W3 XRType = 1 [ZRpaddress] [XRLpaddress] = BB000_W3 BB001_W3

If this is a new interview, XRType = 2, skip to BB001_W3 XRType = 2 BB001_W3

BB000_W3 Whats your address in [ZIWTime]? [ZIWTime]

[IWER: county/city includes conty-level administrative units/county-level city/disrtict ///]

1. Domestic

______ (**BB000_W3_a_1**) province/city/county //

______ (**BB000_W3_a_2**) township/village/neighborhood //// 2. Abroad

BB000_W3_1 What was the type of your address BB000_W3 in [ZIWTime]? [ZIWTime] BB000_W3

1. Family housing
2. Nursing home
3. Hospital
4. Other please specify ______ （**BB000_W3_1_1**）

BB000_W3_2 Was your address, BB000_W3, in the village or city/town? [ZIWTime] BB000_W3

1. The center of city/town
2. Combination zone between urban and rural areas 3. Village
4. Special area

BB001_W3 Whats your address now?

[IWER: county/city includes conty-level administrative units/county-level city/disrtict ///]

1. preload address in the last wave [XRLpaddress] [XRLpad- dress]
2. Other

______ (**BB001_W3_a_1**) province/city/county //

______ (**BB001_W3_a_2**) township/village/neighborhood //// 3. Abroad

BB001_W3_1 What was the type of your address, BB001_W3? BB001_W3

1. Family housing
2. Nursing home
3. Hospital
4. Other please specify ______ （**BB001_W3_1_1**）

BB001_W3_2 Was your addressBB001_W3in the village or city/town? BB001_W3

1. The center of city/town
2. Combination zone between urban and rural areas
3. Village
4. Special area

PROCEDURE :

If this is a new interview, XRType = 2, ask BB001 to BB005 XRType = 2 BB001 BB005
If not, skip to BB005_W3_1 BB005_W3_1

BB001 Where were you born?
[IWER: county/city includes conty-level administrative units/county-level city/disrtict ///]

1. preload current address BB001_W3 BB001_W3
2. Another village/neighborhood in current addresss (BB001_W3) county/city/district BB001_W3///
   ______ (**BB001_1**) township //
   ______ (**BB001_2**) village/neighborhood /
3. Other
   ______ (**BB001_3**) province/city/county/district //
   ______ (**BB001_4**) township/village/neighborhood ////
4. Abroad

BB003 When did you first move to this county/city/district where your current address located(BB001_W3) BB001_W3//______ [1900..2018] Year

BB004 When you first moved to current addressBB001_W3, did you live in the same village/community as you currently do? BB001_W3// /

1. Yes → skip to BB005_W3_1 BB005_W3_1
2. No

BB005 In what year did you first live in current address BB001_W3s village/community BB001_W3 / ______ [1900..2018] Year

BB005_W3_1 Would you change your address in next two years? 2 , ? 1. Yes
2. No → Skip to the procedure before BC001_W3_1 BC001_W3_1

BB005_W3_2 Where would your new address?
1. I am not sure
2. Another house in the same village/community /, 3. Other
______ (**BB005_W3_2_1**) province/city/county //
______ (**BB005_W3_2_2**) township/village/neighborhood ////

PROCEDURE :
If this is a reinterview, XRType = 1, ask BC001_W3_1 to BC002_W3_6 XRType = 1 BC001_W3_1 BC002_W3_6
If this is a new interview, XRType = 2, ask BC002_W3_1 and BC002_W3_4, then skip to the procedure before BD001_W2_4 XRType = 2 BC002_W3_1 BC002_W3_4 BD001_W2_4

BC001_W3_1 We record your Hukou type was [ZBC004] in [ZIWTime], right? [ZIW- Time] [ZBC004]
1. Yes → Skip to BC001_W3_3 BC001_W3_3
2. No

BC001_W3_2 What was your Hukou type in [ZIWTime]? [ZIWTime] 1. Agricultural Hukou
2. Non-agricultural Hukou
3. Unified Residence Hukou
4. Do not have Hukou

BC001_W3_3 Where was your Hukou in [ZIWTime]? [ZIWTime] 1. ______ (**BC001_W3_3_1**) province/city/county //
______ (**BC001_W3_3_2**) township/village/neighborhood //// 2. Did not have Hukou

BC002_W3 Since [ZIWTime], have you Hukou type and Hukou location changed? [ZIW- Time]
1. Only Hukou type has changed
2. Only Hukou registration place has changed
3. Both Hukou type and registration place have changed 4. Neither of Hukou type nor place has changed

PROCEDURE :
If BC002_W3 = 1, 3, ask BC002_W3_1 to BC002_W3_3 BC002_W3 = 1, 3 BC002_W3_1 BC002_W3_3

BC002_W3_1 What is your current Hukou type?

1. Agricultural Hukou
2. Non-agricultural Hukou
3. Unified Residence Hukou
4. Do not have Hukou

BC002_W3_2 Why did your Hukou type change?

1. Go to school
2. Marriage
3. Employment
4. Land is acquired by the government
5. Migration of the whole village
6. Other ______ (**BC002_W3_2_1**)

BC002_W3_3 When did your type of Hukou change? ____ (**BC002_W3_3_1**) Year ___ (**BC002_W3_3_2**) Month

**PROCEDURE** :
If BC002_W3 = 2, 3, ask BC002_W3_4 to BC002_W3_6 BC002_W3 = 2, 3 BC002_W3_4 BC002_W3_6

BC002_W3_4 Whats your current location of Hukou? 1. Preload current address BB001_W3 BB001_W3 2. Other ______ (**BC002_W3_4_1**) province/city/county // ______ (**BC002_W3_4_2**) township/village/neighborhood //// 3. Does not have Hukou

BC002_W3_5 Why did your location of Hukou change?

1. Education
2. Marriage
3. Employment
4. Land is acquired by the government
5. Migration of the whole village
6. Other ______ (**BC002_W3_5_1**)

BC002_W3_6 When did your location of Hukou change? _____ (**BC002_W3_6_1**) Year ______ (**BC002_W3_6_2**) Month

PROCEDURE :
If this is a reinterview, XRType = 1 and [ZFRedu] = 1, ask BD001_W2_4 to BD010_W4, then skip to the procedure before BD012 XRType = 1 [ZFRedu] = 1 BD001_W2_4 BD010_W4 BD012
If this is a reinterview, XRType = 1 and [ZFRedu] = 2, ask BD007_W4_1 to BD010_W4, then skip to the procedure before BD012 XRType = 1 [ZFRedu] = 2 BD007_W4_1 BD010_W4 BD012
If this is a new interview, XRType = 2, ask BD001_W2_4 to BD006, then skip to BD007_W2_1 XRType = 2 BD001_W2_4 BD006 BD007_W2_1

BD001_W2_4 Whats the highest level of education your have now (not including adult education)? ( )

1. No formal education (illiterate) ( )
2. Did not finish primary school
3. Sishu/home school
4. Elementary school
5. Middle school
6. High school
7. Vocational school ( )
8. Two-/Three-Year College/Associate degree
9. Four-Year College/Bachelors degree
10. Masters degree
11. Doctoral degree/Ph.D.

PROCEDURE :
If BD001_W2_4 = 1, 2, 3, ask BD001_W3_1 BD001_W2_4 = 1, 2, 3 BD001_W3_1

BD001_W3_1 Are you literate?

1. Yes
2. No

PROCEDURE :
If BD001_W2_4 ≠ 1, 2, 3, ask BD002_W3 BD001_W2_4 ≠ 1, 2, 3 BD002_W3

BD002_W3 How many years have you spent in school after your highest level of education, BD001_W2_4? (If havent attended school after highest level of education, take down 0) BD001_W2_4 (0) ______ Year

BD006 How old were you when you finished your highest level of education? ___ [0...120] years old
[IWER: If havent attended school, take down 0 0]

BD007_W4_1 Since [ZIWTime], have you attended school for adult education? (Choose all that apply) [ZIWTime] （ ）？（）

1. None → Skip to the procedure before BD012 BD012 2. TV University
3. Night School
4. Zikao (examinations for self-taught students)
5. Hanshou/Correspondence course/Distance learning
6. Literacy course
7. Accelerated education course
8. Other please specify ______ (**BD007_W4_1_1**)

BD008_W4_1 How many years have you spent in adult education since ZIWTime? [ZIW-Time] ______ year

BD009_W4_1 Have you gotten a diploma or degree from the adult education program you have attended since ZIWTime? [ZIWTime]

1. Yes
2. No → Skip to the procedure before BD012 BD012

BD011_W4 What is the highest adult schooling degree or diploma you have since [ZIWTime]? [ZIWTime]

1. Vocational school
2. Two/Three Year College / Associate degree
3. Four Year College / Bachelors degree
4. Others please specify ______ (**BD011_W4_1**)

BD010_W4 When did you attain the degree or diploma? _ Year

BD007_W2_1 Have you attended school for adult education? (Choose all that apply) （）？（ ）

1. None → Skip to the procedure before BD012 BD012 2. TV University
3. Night School
4. Zikao (examinations for self-taught students)
5. Hanshou/Correspondence course/Distance learning
6. Literacy course
7. Accelerated education course
8. Other Please specify ______ (**BD007_W2_1_1**)

BD008_W2_1 How many years did you spend in adult education? ___ year

BD009_W2_1 Did you get a diploma or degree from the adult education program you attended?

1. Yes
2. No → Skip to the procedure before BD012 BD012

BD011 What is the highest adult schooling degree or diploma you have?

1. Vocational school
2. Two/Three Year College / Associate degree
3. Four Year College / Bachelors degree
4. Others Please specify ______ (**BD011_1**)

BD010 When did you attain the degree or diploma? ______ [1900...2018] year

PROCEDURE :

If this is a reinterview, XRType = 1, ask BD012 to BD016_W4, then skip to BE001 XRType = 1 BD012 BD016_W4 BE001

If this is a new interview, XRType = 2, skip to BD012_W4 XRType = 2 BD012_W4

BD012 Since [ZIWTime], have you participated in vocational and technical training? [ZIWTime]

1. Yes
2. No → Skip to BE001 BE001

BD013 Since [ZIWTime], how many times have you participated in vocational and technical training? [ZIWTime] ______ Times

BD014 Since [ZIWTime], how many months have you spent participating in vocational and technical training? [ZIWTime] ______ Months

[IWER: If respondent has participated in training more than once, please add up the traing time]

BD015_W4 Since [ZIWTime], have you attained vocational skill certificate by participating in vocational and technical training? [ZIWTime]

1. Yes
2. No → Skip to BE001 BE001

BD016_W4 Since [ZIWTime], what vocational skill certificates you have attained by participating in vocational and technical training? [ZIWTime] ______

BD012_W4 Have you participated in vocational and technical training?

1. Yes
2. No → Skip to BE001 BE001

BD013_W4 How many times have you participated in vocational and technical training? ___ Times

BD014_W4 How many months have you spent participating in vocational and technical training? ______ Months

[IWER: If respondent has participated in training more than once, please add up the traing time]

BD017_W4

1. Yes
2. No → Skip to BE001 BE001

BD018_W4 Have you attained vocational skill certificate by participating in vocational and technical training? _______

BE001 What is your marital status?

[IWER: common-law marriage is considered as married]

1. Married and live with spouse → Skip BE002 BE002
2. Married but dont living with spouse temporarily for reasons such as work → Skip BE002 BE002
3. Separated, dont live together as a couple anymore ( ) 4. Divorced
5. Widowed
6. Never married

BE002 Do you have a mate living with you as a couple (cohabit)? ( )

1.
2. → Skip BE003_W4 BE003_W4

BE003_W4 How long have you been living with your spouse in the last year? / ______ [0...12] Month

PROCEDURE :

If this is a reinterview, XRType = 1, and the respondent had separated from his/her spouse for a long time, CV004 = 3, ask BF005_W4 and BF005_W4_3, then skip to BF008 XRType= 1 CV004 = 3 BF005_W4 BF005_W4_3 BF008

If this is a reinterview, XRType = 1. And the respondent had divorced, CV004 = 2, skip to BF006_W4 XRType = 1 CV004 = 2 BF006_W4

If not, skip to BG001_W4 BG001_W4

BF005_W4 When did you separate from your [name of ex-spouse]? [ ] ______ 1900...2015 (**BF005_W4_1**) Year ___ 1...12 (**BF005_W4_2**) Month

BF005_W4_3 Why did you separate from your [name of ex-spouse]? []

1. Emotional feud
2. Other please specify ______ (**BF005_W4_3_1**)

BF006_W4 When did you divorce [name of ex-spouse]? [] _ [2011...2018] (**BF006_W4_1**) Year 1...12 (**BF006_W4_2**) Month

BF006_W4_3 Whats the main reason why you divorced your [name of ex-spouse]? [ ]

1. Emotional feud
2. Live in different places
3. In order to facilitate the property purchase transactions 4. Other please specify ____ (**BF006_W4_3_1**)

BF006_W4_4 When you divorced [name of ex-spouse], regarding divided assets, how much is yours including real estate, car, etc calculated as currency [] ( ) ______ (**BF006_W4_4_1**) Yuan How much is [name of ex-spouse] [] ______ (**BF006_W4_4_2**) Yuan

BF006_W4_5 When you divorced [name of ex-spouse], did you have any infancy children (under 18)? [] (18)

1. Yes there is/are ______ (**BF006_W4_5_1**) child/children
2. No → Skip to BG001_W4 BG001_W4

BF006_W4_6 When you divorced [name of ex-spouse], who brought up infancy child/children? []

Ibrought up ______ (**BF006_W4_6_1**) child/children [name of ex-spouse] brought up [] _____ (**BF006_W4_6_2**) child/children

[IWER: Take down 0 if one didnt bring up children. The total number of children here should match the number of children when they divorced 0]

PROCEDURE :
If BF006_W4_6_1 ≥ 1ask BF006_W4_7 BF006_W4_6_1 ≥ 1 BF006_W4_7

BF006_W4_7 How much does [name of ex-spouse] pay for you every month as child support payment? [] ______ Yuan

PROCEDURE :
If BF006_W4_6_2 ≥ 1ask BF006_W4_8 BF006_W4_6_2 ≥ 1 BF006_W4_8

BF006_W4_8 How much do you pay for [name of ex-spouse] every month as child support payment? [] ______ Yuan

BG001_W4 Are you Han or Ethnic Minorities?

1. Han
2. Zhuang
3. Man
4. Hui
5. Miao
6. Weiwuer
7. Tujia
8. Yi
9. Mongol
10. Zang
11. Other please specify ______ (**BG001_W4_1**)

BG002_W4 Do you have any religious belief? Such as Buddhism, Taosim, Christianity, etc.

1. Yes
2. No → Skip BG003_W4 BG003_W4

BG003_W4 Which religious belief do you believe in?

1. Buddhism
2. Taosim
3. Islam
4. Catholicism
5. Christianity
6. Other please specify ______ (**BG003_W4_1**)

BG004_W4 Are you the Communist party member?

1. Yes When did you join the Cmmunist Party ______ (**BG004_W4_1**) Year
2. No

BG005_W4 Did you participate in the Up to Mountains and Down to the Countryside Movement as an educated youth?

1. Yes When did you participate in the Up to Mountains and Down to the Country- side Movement as an educated youth ______ (**BG005_W4_1**) Year
2. No → Skip BG006_W4 BG006_W4

BG006_W4 Were you back to the city after the Up to Mountains and Down to the Countryside Movement as an educated youth?

1. Yes When were you back to the city ______ (**BG006_W4_1**) Year 2. No

BF008 Interviewer records respondent behavior throughout the questionnaire: how often did [name of respondent] receive assistance in answering this module, Demographic backgrounds? []

1. Never
2. A few times
3. Most of time
4. Proxy by others

C Family

PROCEDURE :

If the value of [MRs spouse name] is not null, ask CV009, and save the name as [Name of the family respondent] and save name of the other respondent as [Name of spouse of the family respondent] [ ] CV009 [] []

If the value of [MRs spouse name] is null, save [Main Respondents name] as [Name of the family respondent] and save [Name of spouse of the family respondent] as null, and skip directly to PARENT, CHILDREN AND SIBLING INFORMATION [ ] [] [][]

CV009 We would like to know the information about your family members, including children, parents and siblings of you, and your husband/wife, etc. Who has a better idea about such information, [Main Respondents name] or [MRs spouse name]? [] []

1. [Main Respondents name][]
2. [MRs spouse name][]

C1 Parent, Children and Sibling Information

CA Parent Information

CAPI:

CA section is regarding parent information of [Name of the family respondent] and [Name of spouse of the family respondent]. Respondent for this part should be [Name of the family respondent], while it is allowed for [Name of spouse of the family respondent] to answer questions about his/her own parents during on-site interview. Answers to questions in this part will be saved under [Name of the family respondent]s ID CA [] [] CA [] [] CA [] ID

Codes for parents of [Name of the family respondent] are: 1 Biological father, 2 Biological mother, 3 Adoptive father, 4 Adoptive mother; and codes for parents of [Name of spouse of the family respondent] are: 5 Biological father, 6 Biological mother, 7 Adoptive father, 8 Adoptive mother [] 1 2 3 4 [] 5 6 7 8

The following intermediate variables (series) will be generated, with the codes for parents/parents-in-law as subscript: （）

- XParExist denotes whether such parent exists or not
- XParBirth denotes the date ofbirth of the parents
- XParAlive denotes whether such parent still alives or not

[INTRO: Firstly, here are a couple of questions about parents of [Name of the family respondent] []]

CA000_W4_0 Does [Name of the family respondent] and [Name of spouse of the family respondent] know information about biological father of [Name of the family respondent]? [ ] [] []

[IWER: The vast majority of people know information about their biological father, unless under certain circumstances where they were adopted by strangers upon birth, they may have no idea about their biological father. During on-site interview, there is no need to ask respondent this question and select Yes, I know directly. In the subsequent parts of the interview, if youve got to know that the respondent was given away to someone else for adoption or if he/she was abandoned upon birth, you should return to this question and select No, I dont after a telephone call for approval code and skip questions about biological father of [Name of the family respondent]]

1. Yes, I know → CAPI updates the value of XParExist[1] to 1 CAPI XParEx- ist[1] 1
2. No, I dont. Please specify the reason why the respondent doesnt know information about his/her biological father ______ (**CA000_W4_0_1**) input the approval code here ______ (**CA000_W4_0_2**) → CAPI updates the value of XParExist[1] to 0 CAPI XParEx- ist[1] 0

PROCEDURE :

Ask the family respondent and his/her spouse (if applicable) questions from CA000_W4_1 to CA007_W3_5 . Automatically preload [Name of the family respondent] or [Name of spouse of the family respondent] in corresponding questions, choices, prefaces, and logics of procedures, with all variables postfixed and i ∈ {1, 2} as loop index ( )

CA000_W4_1 CA007_W3_5 [] [] i i ∈ {1, 2}

PROCEDURE :

If XParExist[1] = 1 go on with the next question, otherwise goto CA001_W4_0 XParEx- ist[1] = 1 CA001_W4_0

If the value of [ZParBirth[1]] is not null, question CA000_W4_1 should be asked [ZParBirth[1]] CA000_W4_1

CA000_W4_1 Was the biological father of [Name of the family respondent] born in [ZParBirth[1]]? [] [ZParBirth[1]]

1. Yes → CAPI updates XParBirth[1] to ZParBirth[1], and go directly to CAPI XParBirth[1] ZParBirth[1] CA000_W3_2
2. No

CA000_W3_1 When was the biological father of [Name of the family respondent] born? [ ] _

Year [1800..1970]

[IWER: Mark the year using four digits. If the respondent does not remember the birth year of his/her biological father clearly, you could calculate it from information about his/her biological fathers age now, his/her biological fathers death year and the age of death, or age of his/her biological father when he/she was born 4
]

[CAPI: update XParBirth[1] to the value of CA000_W3_1 XParBirth[1] CA000_W3_1]

CA000_W3_2 Is [Name of the family respondent]s biological father still alive? [ ]

1. Yes → CAPI updates XParAlive[1] to 1, and go to procedures before CA001_W4_1 CAPI XParAlive[1] 1 CA001_W4_1
2. No → CAPI updates XParAlive[1] to 0, and go on with the next question CAPI XParAlive[1] 0

PROCEDURE :
If [ZParDYear[1]] is null, go on with question CA000_W3_3 [ZParDYear[1]] CA000_W3_3

CA000_W3_3 When did [Name of the family respondent]s biological father pass away? [ ] ? _ (**CA000_W3_3_1**) Year or how old was he when he died? ______ (**CA000_W3_3_2**) Age

PROCEDURE :
If [ZParDReason[1]] is null, go on with question CA000_W3_4 [ZParDReason[1]] CA000_W3_4

CA000_W3_4 Whats the leading cause of death for [Name of the family respondent]s biological father? [] _______

[IWER: If the cause of death is illness, please specify the kind of illness. For example, if it is cancer, the specific type of cancer should be recorded (gastric cancer, lung cancer, etc.); if it is infectious disease, please specify the disease (tuberculosis, dysentery, etc.); or if it is an accident, please give information on the accident type (car accident, fire, intoxication, etc.) ( ) ( )]

CA001_W4_0 Does [Name of the family respondent] and [Name of spouse of the family respondent] know information about biological mother of [Name of the family respondent]? [ ] [] []

[IWER: The vast majority of people know information about their biological mother, unless under certain circumstances where they were adopted by strangers upon birth, they may have no idea about their biological mother. During on-site interview, there is no need to ask respondent this question and select Yes, I know directly. In the subsequent parts of the interview, if youve got to know that the respondent was given away to someone else for adoption or if he/she was abandoned upon birth, you should return to this question and select No, I dont after a telephone call for approval code and skip questions about biological mother of [Name of the family respondent]]

1. Yes, I know → CAPI updates the value of XParExist[2] to 1 CAPI XParEx- ist[2] 1
2. No, I dont Please specify the reason why the respondent doesnt know information about his/her biological mother

   ______ (**CA001_W4_0_1**) input the approval code here ______ (**CA001_W4_0_2**) → CAPI updates the value of XParExist[2] to 0 CAPI XParExist[2] 0

**PROCEDURE** :<br>If XParExist[2] = 1 go on with the next question, otherwise skip to CA006_W3_1 XParExist[2] = 1CA006_W3_1<br>If the value of [ZParBirth[2]] is not null, question CA001_W4_1 should be asked [ZPar- Birth[2]] CA001_W4_1

CA001_W4_1 Was the biological mother of [Name of the family respondent] born in [ZPar- Birth[2]]? [] [ZParBirth[2]]

1. Yes → CAPI updates XParBirth[2] to ZParBirth[2], and go directly to CAPI XParBirth[2] ZParBirth[2] CA001_W3_2
2. No

CA001_W3_1 When was the biological mother of [Name of the family respondent] born? [ ]

Year [1800..1970]

[IWER: Mark the year using four digits. If the respondent does not remember the birth year of his/her biological mother clearly, you could calculate it from information about his/her biological mothers age now, his/her biological mothers death year and the age of death, or age of his/her biological mother when he/she was born 4

]

[CAPI: Update XParBirth[2] to the value of CA001_W3_1 XParBirth[2] CA001_W3_1]

CA001_W3_2 Is [Name of the family respondent]s biological mother still alive? [ ]

1. Yes → CAPI updates XParAlive[2] to 1, and go to procedures before CA002_W4 CAPI XParAlive[2] 1 CA002_W4
2. No → CAPI updates XParAlive[2] to 0, and go on with the next question CAPI XParAlive[2] 0

**PROCEDURE** :<br>If [ZParDYear[2]] is null, go on with question CA001_W3_3 [ZParDYear[2]] CA001_W3_3

CA001_W3_3 When did [Name of the family respondent]s biological mother pass away? [ ] ? _ (**CA001_W3_3_1**) Year or how old was she when she died? ______ (**CA001_W3_3_2**) Age

PROCEDURE :

If [ZParDReason[2]] is null, go on with question CA001_W3_4 [ZParDReason[2]] CA001_W3_4

CA001_W3_4 Whats the leading cause of death for [Name of the family respondent]s biological mother? [] ______

[IWER: If the cause of death is illness, please specify the kind of illness. For example, if it is cancer, the specific type of cancer should be recorded (gastric cancer, lung cancer, etc.); if it is infectious disease, please specify the disease (tuberculosis, dysentery, etc.); or if it is an accident, please give information on the accident type (car accident, fire, intoxication, etc.) ( ) ( )]

PROCEDURE :

If ZParAlive[1] = 1 and ZParAlive[2] = 1, go on with question CA002_W4 ZParAlive[1] = 1 ZParAlive[2] = 1 CA002_W4

CA002_W4 Are [Name of the family respondent]s biological father and biological mother still spouse of each other?[]

[IWER: Long-term separation due to lack of affection constitutes divorce in this context; and a couple remarrying each other after a divorce are not deemed as divorced]

1. Yes
2. No

CA006_W3_1 Does [Name of the family respondent] have an adoptive father? [ ]

1. Yes → CAPI updates XParExist[3] to 1, and go on with the next question CAPI XParExist[3] 1
2. No → CAPI updates XParExist[3] to 0, and go to CAPI XParExist[3] 0 CA007_W3_1

CA006_W3_2 Does [Name of the family respondent]s adoptive father still alive? [ ]

[IWER: If more than one adoptive fathers exist, please ask about information on the last one that the respondent stay with []]

1. Yes → CAPI updates XParAlive[3] to 1, and go on with the next question CAPI XParAlive[3] 1
2. No → CAPI updates XParAlive[3] to 0, and go to CAPI XParAlive[3] 0 CA007_W3_1

PROCEDURE :
If [ZParBirth[3]] is not null, go on with question CA006_W4_3 [ZParBirth[3]] CA006_W4_3

CA006_W4_3 Was the adoptive father of [Name of the family respondent] born in [ZParBirth[3]]? [] [ZParBirth[3]]

1. Yes → CAPI updates XParBirth[3] to ZParBirth[3], and go directly to CAPI XParBirth[3] ZParBirth[3] CA007_W3_1
2. No

CA006_W3_3 When was the adoptive father of [Name of the family respondent] born? [ ] _ Year [1800..1970]

[IWER: Mark the year using four digits. If the respondent does not remember the birth year of his/her adoptive father clearly, you could calculate it from information about his/her adoptive father age now, his/her adoptive fathers death year and the age of death, or age of his/her adoptive father when he/she was born 4]

[CAPI: Update XParBirth[3] to the value of CA006_W3_3] XParBirth[3] CA006_W3_3]

CA007_W3_1 Does [Name of the family respondent] have an adoptive mother? [ ]

1. Yes → CAPI updates XParExist[4] to 1, and go on with the next question CAPI XParExist[4] 1
2. No → CAPIupdates XParExist[4] to 0, and goto procedures before CA007_W3_5 directly CAPI XParExist[4] 0 CA007_W3_5

CA007_W3_2 Does [Name of the family respondent]s adoptive mother still alive? [ ]

[IWER: If more than one adoptive mothers exist, please ask about information on the last one that the respondent stay with []]

1. Yes → CAPI updates XParAlive[4] to 1, and go on with the next question CAPI XParAlive[4] 1
2. No → CAPI updates XParAlive[4] to 0, and go to procedures before CA007_W3_5 directly CAPI XParAlive[4] 0 CA007_W3_5

PROCEDURE :
If [ZParBirth[4]] is not null, go on with question CA007_W4_3 [ZParBirth[4]] CA007_W4_3

CA007_W4_3 Was the adoptive mother of [Name of the family respondent] born in [ZParBirth[4]]? [] [ZParBirth[4]]

1. Yes → CAPI updates XParBirth[4] to ZParBirth[4], and go to procedures before CA007_W3_5 CAPI XParBirth[4] ZParBirth[4] CA007_W3_5
2. No

CA007_W3_3 When was the adoptive mother of [Name of the family respondent] born? [ ] _ Year [1800..1970]

[IWER: Mark the year using four digits. If the respondent does not remember the birth year of his/her adoptive mother clearly, you could calculate it from information about his/her adoptive father age now, his/her adoptive mothers death year and the age of death, or age of his/her adoptive mother when he/she was born 4]

[CAPI: Update XParBirth[4] to the value of CA007_W3_3 XParBirth[4] CA007_W3_3]

PROCEDURE :

If XParAlive[3] = 1 and XParAlive[4] = 1, go on with question CA007_W3_5 XParAlive[3] = 1 XParAlive[4] = 1, CA007_W3_5

CA007_W3_5 Are [Name of the family respondent]s adoptive father and adoptive mother still spouse of each other? []

1. Yes
2. No

CAPI: Ask the respondent questions from CA009_W4_i to CA026_W3_i on his/herbiological father, biological mother, adoptive father and adoptive mother sequentially with i as the loop index and following the rules below [] CA009_W4_i CA026_W3_i i

Define such a series as [biological father, biological mother, adoptive father and adoptive mother,biological father, biological mother, adoptive father and adoptive mother] ParType []

Questions will be asked only if XParExist[i] = 1 XParExist[i] = 1

PROCEDURE :

- For biological parents, i.e. i = 1, 2, questions CA009_W4_i to CA023_W3_i should be asked no matter the parent is alive or not; questions CA016_W3_i to CA026_W3_i are only applicable to biological parents who is still alive (i.e. XParAlive[i] = 1) i = 1, 2 CA009_W4_i CA023_W3_i （XParAlive[i] = 1） CA016_W3_i CA026_W3_i
- For other type of parents, i.e. i = 3, 4, questions CA009_W4_i to CA026_W3_i should only be asked for alive ones (i.e. XParAlive[i] = 1) i = 3, 4XParAlive[i] = 1 CA009_W4_i CA026_W3_i

PROCEDURE :

If [ZParEdu[i]] is not null, go on with question CA009_W4_i [ZParEdu[i]] CA009_W4_i

CA009_W4_i Is [Name of the family respondent]s [ParType[i]]s highest achieved education [ZParEdu[i]]? [] [ParType[i]] [ZParEdu[i]]

1. Yes → Skip to procedures before CA014_W4_i directly CA014_W4_i
2. No

CA009_i Whats [Name of the family respondent]s [ParType[i]]s highest achieved education? [] [ParType[i]]

1. No formal education (illiterate)
2. Did not finish elementary school
3. Sishu/home school
4. Elementary school
5. Middle school
6. High school
7. Vocational school ( )
8. Two-/Three-Year College / Associate degree
9. Four-Year College / Bachelors degree
10. Post-graduate, Masters degree
11. Post-graduate, Doctoral degree/Ph.D

PROCEDURE :

If CA009_i = 1, 2, 3, go on with question CA009_W3_iCA009_i = 1, 2, 3CA009_W3_i

CA009_W3_i Is [Name of the family respondent]s [ParType[i]] literate? [] [ParType[i]]

1. Yes
2. No

PROCEDURE :

If [ZParOcc[i]] is not null, go on with question CA014_W4_i [ZParOcc[i]] CA014_W4_i

CA014_W4_i Is [ZParOcc[i]] [Name of the family respondent]s [ParType[i]]s highest achieved position in his/her career? [] [ParType[i]] [ZParOcc[i]]

1. Yes → Skip to CA023_W3_i
2. No

CA014_i Whats [Name of the family respondent]s [ParType[i]]s highest achieved position in his/her career? [] [ParType[i]]

1. Managers in an enterprise or the persons in charge of general affairs in government agency/ organization of political party/ public service organization
2. Professionals and technicians
3. Clerks
4. Workers in business and services
5. Workers in Agriculture, forestry, animal husbandry and fishery
6. Operating personnel of production/ transportation equipment
7. Others, please specify ______ (**CA014_1_i**)

CA023_W3_i Is [Name of the family respondent]s [ParType[i]] a member of the Communist Party? [] [ParType[i]]

1. Yes
2. No

CA016_W3_i Where was [Name of the family respondent]s [ParType[i]] born? [ ] [ParType[i]]

______ (**CA016_W3_1_i**) Province-city-county --

______ (**CA016_W3_2_i**) District-county/district-village/community --

[CAPI: Drop-down lists are provided for all levels of address, of which the option of other is available for choice and corresponding manual inputs are allowed for address level of village]

CA016_i Where does [Name of the family respondent]s [ParType[i]] live? [] [ParType[i]]

1. Living with [Name of the family respondent] []
2. Living in houses in the same/neighboring courtyard(s) or flats in the same/neighboring building(s) as/of [Name of the family respondent]s [] ( ) ( )
3. The place where he/she was born in [] [ParType[i]]
4. Other ______ (**CA016_1_i**) Province-city-county --______ (**CA016_2_i**) District- county/district -village/community --
5. Living abroad

[CAPI: Drop-down lists are provided for all levels of address, of which the option of other is available for choice and corresponding manual inputs are allowed for address level of village]

PROCEDURE :

If CA016_i = 4, 5, go on with question CA017_i CA016_i = 4, 5, CA017_i

CA017_i The place where [Name of the family respondent]s [ParType[i]] lives in is [ ]
[ParType[i]]
1. Urban areas
2. Suburban areas
3. Rural areas
4. Other special areas

CA026_W3_i Does [Name of the family respondent]s [ParType[i]] have the ability to take care of him/herself? [] [ParType[i]]
1. Yes
2. No

PROCEDURE : []

If the value of [Name of spouse of the family respondent] is not null, questions CA009_W4_i to CA026_W3_i should then be asked to gather information on [Name of spouse of the family respondent]s biological father, biological mother, adoptive father and adoptive mother, with i ∈ {5, 6, 7, 8} being the loop index [ ] CA009_W4_i CA026_W3_i ii ∈ {5, 6, 7, 8}

CAPI: CAPI: Generate a new series XParName = [Name of the family respondent]s biological father, [Name of the family respondent] biological mother, [Name of the family respondent]s adoptive father, [Name of the family respondent]s adoptive mother, [Name of spouse of the family respondent]s biological father, [Name of spouse of the family respondent] biological mother, [Name of spouse of the family respondent]s adoptive father, [Name of spouse of the family respondent]s adoptive mother. The newly defined series XParName, together with the previously defined XParExist and XParAlive will be used in subsequent sections to preload the names of the repondents parents in relevant questions XParName = [[ ] [] [] [] [] [ ] [ ] []] XParExist XParAlive

CB Children Information

CAPI:

CB section is regarding childrens information of [Name of the family respondent]. Respondent for this part should be [Name of the family respondent], while it is allowed for [Name of spouse of the family respondent] to answer questions in this section during on-site interview. Answers to questions in this part will be saved under [Name of the family respondent]s ID. All relationships are defined from the perspective of [Name of the family respondent] CB [] CB [] [] CB [] ID []

Codes for children of [Name of the family respondent] will follow rules as they are preloaded in CAPI. Any newly mentioned children will be coded after the preloaded ones. If the respondent is added to the survey for the first time, the codes for children will start from 1 [] 1

The following intermediate variables (series) will be generated, with the codes for children as subscript ( )

- XChildName a string variable, saving names of children
- XChildGender drop-down list provided, with 1 denoting male and 2 denoting female, saving genders of children 1 2
- XChildBirth an integer of 4 digits, saving the birth year of children
- XChildType drop-down list provided, with 1 denoting biological child of [Name of the family respondent] and [Name of spouse of the family respondent], 2 denoting adoptive child of [Name of the family respondent] and [Name of spouse of the family respondent], 3 denoting biological child of [Name of the family respondent], 4 denoting biological child of [Name of spouse of the family respondent], 5 denoting not my children ; if the value of [Name of spouse of the family respondent] is null,

  CAPI will not show options with [Name of spouse of the family respondent] 1 [] [] 2 [] [ ] 3 [] 4 [] 5 []
- XChildEdu saving education of children; definition for this can be found in CB052_W3_i CB052_W3_i

[INTRO: In the following section, we would like to ask some questions about your children]

PROCEDURE :

If this household has been already included in our previous survey (XHType = 1), and ZChildNum ≠ 0, skip to CB050_W4_1 XHType = 1 ZChildNum ≠ 0 CB050_W4_1

If this household has been already included in our previous survey (XHType = 1), and ZChildNum = 0, skip to CB051_W3_1 XHType = 1 ZChildNum = 0 CB051_W3_1

If this household is added into the survey for the first time (XHType = 2), questions CB050_W3 and CB050_W4 should be asked and then skip to procedures before CB039_i XHType = 2 CB050_W3 CB050_W4 CB039_i CAPI

CB050_W3 Including biological children, stepchildren, and adoptive children. How manychildren who are still alive does [Name of the family respondent] have? [] ______

PROCEDURE :

If CB050_W3 ≥ 1, go on with question CB050_W4, otherwise skip all questions about children CB050_W3 ≥ 1 CB050_W4

CB050_W4 Please ask the respondent the following questions regarding his/her children in the order from the oldest to the youngest

[CAPI: CB050_W3 is the number of rows/children; the option 5 not my children should not be available for choice for this question CB050_W3 5]

Serial number of children	(XChildName)	(XChildGender)	(XChildBirth)	(XChildType)
1				
2				
...				
CB050_W3				

CB050_W4_1 Please confirm with the respondent the following information regarding his/her children in the order from the oldest to the youngest; If true, leave it as it is; if false, correct it accordingly in the following table. Please be noted that childerns education cannot be adjusted in this table

[IWER: Pleasedont skip to questions about another child before you finish asking/confirming all information about one particular child. Typos or other kinds of errors in childrens names may occur due to misunderstanding of answers given in dialect; in such cases, please check with the respondent carefully before you conclude that this child does not exist]

[CAPI: In the following table, all information except for children education can be modified directly according to respondents answers]

Serial number of children	(ZChildName)	(ZChildGender)	(ZChildBirth)	(ZChildAlive)	(ZChildType)	(ZChildEdu)
1						
2						
...						
ZChildNum						

[CAPI: Update the values of relevant Xchild series according to confirmation/ modification; dont generate variables for children classified as 5 not my children in ZChildType XChild ZChildType XChild]

CB051_W3_1 In addition to [XChildName], does [Name of the family respondent] have any other children who are still alive, including biological children, stepchildren and adoptive children? [XChildName][] ______ Number

PROCEDURE :
If CB051_W3_1 ≥ 1, go on with question CB051_W4 CB051_W3_1 ≥ 1 CB051_W4

CB051_W4 Please give information about this/ these child(ren) mentioned in CB051_W3_1 in the order from the oldest to the youngest [ ] CB051_W3_1

[CAPI: The number of rows in the below table is CB051_W3_1; and the serial number for children starts from ZChildNum+1 CB051_W3_1 ZChildNum+1]

	(XChildName)	(XChildGender)	(XChildBirth)	(XChildType)
ZChildNum+1				
ZChildNum+2				
...				
ZChildNum+ CB051_W3_1				

CAPI: For each child who has been dead, i.e. XChildAlive= 0, questions CB039_i to CB040_W4_i should be asked iteratively, with i being the loop index XChildAlive = 0 CB039_i CB040_W4_i i

PROCEDURE :
If ZChildDYear[i] is null, go on with question CB039_i ZChildDYear[i] CB039_i

CB039_i When did [XChildName[i]] pass away? [XChildName[i]] ? ______ (**CB039_1_i**) Year or his/her age when he/she died ______ (**CB039_2_i**) Age

PROCEDURE :
If ZChildDReason[i] is null, go on with question CB040_W4_i ZChildDReason[i] CB040_W4_i

CB040_W4_i Whats the leading cause of death for [XChildName[i]]? [XChildName[i]] ______

[IWER: If the cause of death is illness, please specify the kind of illness. For example, if it is cancer, the specific type of cancer should be recorded (gastric cancer, lung cancer, etc.); if it is infectious disease, please specify the disease (tuberculosis, dysentery, etc.); or if it is an accident, please give information on the accident type (car accident, fire, intoxication, etc.) ( ) ( )]

CAPI: For each child who is still alive, i.e. XChildAlive = = 1, questions CB052_W4_i to CB063_W3_6_i should be asked iteratively, with i being the loop index （XChildAlive = 1） CB052_W4_i CB063_W3_6_i i

PROCEDURE :
If ZChildEdu[i] is not null, go on with question CB052_W4_i ZChildEdu[i] CB052_W4_i

CB052_W4_i Without taking continuing education into account, is [ZChildEdu[i]] [XChildName[i]s highest achieved education? [XChildName[i]] [ZChildEdu[i]]

1. Yes → Skip to CB053_i
2. No

CB052_W3_i Without taking continuing education into account, whats [XChildName[i]s highest achieved education? [XChildName[i]]

1. No formal education (illiterate)
2. Did not finish elementary school
3. Sishu/home school
4. Elementary school
5. Middle school
6. High school
7. Vocational school ( )
8. Two-/Three-Year College/Associate degree
9. Four-Year College/Bachelors degree
10. Masters degree

11. Doctoral degree/Ph.D.
997. Have no idea
999. Refuse to answer this question

PROCEDURE :
If CB052_W3_i = 1, 2, 3, go on with question CB052_W3_1_i CB052_W3_i = 1, 2, 3, CB052_W3_1_i

CB052_W3_1_i Is [XChildName[i]] literate? [XChildName[i]]
1. Yes
2. No

CB053_i Where does [XChildName[i]] live? [XChildName[i]]
1. Living with [Name of the family respondent] and not financially independent [ ]
2. Living with [Name of the family respondent] and financially independent [ ]
3. Living in houses in the same/neighboring courtyard(s) or flats in the same/neighboring building(s) as/of [Name of the family respondent]s [] ( ) ( )
4. Other ______ (**CB053_1_i**) Province-city-county/district --______ (**CB053_2_i**) county/district -village/community --
5. Living abroad
997. Have no idea
999. Refuse to answer this question

[CAPI: Drop-down lists are provided for all levels of address, of which the option of other is available for choice and corresponding manual inputs are allowed for address level of village]

PROCEDURE :
If CB053_i = 4, 5, go on with question CB054_i CB053_i = 4, 5 CB054_i

CB054_i The place where [XChildName[i]] lives in is [XChildName[i]] 1. Urban areas
2. Suburban areas
3. Rural areas
4. Other special areas
997. Have no idea
999. Refuse to answer this question

CB055_i What is [XChildName[i]]s hukou type? [XChildName[i]]
[IWER: Unified Residency Hukou is a hukou type in some areas where Hukou system has

been reformed and it no longer differentiates between agricultural and non-agricultural residents. Instead, a single hukou type is used]

1. Agriculture Hukou
2. Non-Agriculture Hukou
3. Unified Residency Hukou
4. Do not have Hukou → Skip to CB063_W3_2_i CB063_W3_2_i 997. Have no idea → Skip to CB057_i CB057_i

999. Refuse to answer this question → Skip to CB057_i CB057_i

PROCEDURE:
If CB055_i = 3, go on with questions CB055_W2_1_i and CB055_W2_2_i CB055_i = 3 CB055_W2_1_i CB055_W2_2_i

CB055_W2_1_i What was [XChildName[i]]shukou type before his/her Unified Residency Hukou? [XChildName[i]]

1. Agriculture Hukou
2. Non-Agriculture Hukou
3. Do not have Hukou

997. Have no idea
999. Refuse to answer this question

CB055_W2_2_i When did [XChildName[i]] get the Unified Residency Hukou? [XChildName[i]]
Year
[CAPI: The answer could be dont know here]

CB057_i Whats the registered location in [XChildName[i]] s hukou? [XChildName[i]]

1. The place where he/she was born in /
2. The place where he/she lives in /
3. Other ______ (**CB057_1_i**) Province-city-county --______ (**CB057_2_i**) county / district-village / community --

997. Have no idea
999. Refuse to answer this question

[CAPI: Drop-down lists are provided for all levels of address, of which the option of other is available for choice and corresponding manual inputs are allowed for address level of village]

CB063_W3_2_i Is [XChildName[i]] a member of the Communist Party? [XChildName[i]]

1. Yes
2. No

997. Have no idea
999. Refuse to answer this question

CB063_W3_5_i Does [XChildName[i]] have any religious belief? [XChildName[i]]

1. Yes
2. No → Skip CB063_W4_6_i CB063_W4_6_i

997. Have no idea → Skip CB063_W4_6_i CB063_W4_6_i

999. Refuse to answer this question → Skip CB063_W4_6_i CB063_W4_6_i

CB063_W4_6_i Whats XChildName[i]]s religious belief? [XChildName[i]]

1. Buddhism
2. Taoism
3. Islam
4. Catholicism
5. Christian
6. Other, Please specify ______ (**CB063_W4_6_1_i**)

CB070_W4_i Is [XChildName[i]] a student or does [XChildName[i]] work? [XChildName[i]]

[IWER: By definition here, work means being a farmer, doing work for salary, self-employed or assisting in family business without getting paid]

1. Working
2. Student
3. A student working part-timely or full-time worker while studying part-timely
4. Neither a student nor does he work please specify what he has been doing ______ (**CB070_W4_1_i**)

997.
999.

PROCEDURE :

If CB070_W4_i = 1, go on with question CB71_i, and then skip to procedures before CB063_i CB070_W4_i = 1 CB71_i CB063_i

If CB070_W4_i = 2, skip to CB059_W4_i CB070_W4_i = 2 CB059_W4_i

If CB070_W4_i = 3, go on with question CB71_i and CB059_W4_i CB070_W4_i = 3

If CB070_W4_i = 4, 997, 999, skip to procedures before CB063_i CB070_W4_i = 4, 997, 999 CB063_i

CB071_i What does [XChildName[i]] do? [XChildName[i]]

1. Managers in an enterprise or the persons in charge of general affairs in government agency/ organization of political party/ public service organization
2. Professionals and technicians
3. Clerks
4. Workers in business and services
5. Workers in Agriculture, forestry, animal husbandry and fishery
6. Operating personnel of production/ transportation equipment
7. Others, please specify ______ (**CB071_1**_i)

CB059_W4_i What kind of education is [XChildName[i]] receiving now? [XChildName[i]]

1. Preschool education
2. Primary school
3. Middle school
4. High school
5. College/ University
6. Graduate school (Masters degree)
7. Graduate school (Doctoral degree)
8. Others, please specify ______ (**CB059_W4_1**_i)

**PROCEDURE**: If [XChildName[i]] >16, i.e. XChildBirth[i] ≤ 2002, go on with questions CB063_i to CB072_W3_i, otherwise skip to CB063_W3_1_i [XChildName[i]] 16 XChild- Birth[i] ≤ 2002 CB063_i CB072_W3_i CB063_W3_1_i

CB063_i Whats [XChildName[i]]s current marital status? [XChildName[i]]

1. Married with spouse present → Skip CB063_W4_iCB063_W4_i
2. Married but not living with spouse temporarily for reasons such as work → Skip CB063_W4_i CB063_W4_i 3. Separated ()
4. Divorced
5. Widowed
6. Never married

997. Have no idea → Skip to CB065_i CB065_i
999. Refuse to answer this question → Skip to CB065_i CB065_i

CB063_W4_i Is there anyone who is living together with [XChildName[i]] as his/her partner? [XChildName[i]]

1. Yes
2. No

997. Have no idea
999. Refuse to answer this question

PROCEDURE :
If [XChildName[i]] has a partner currently, i.e. CB063_i = 1, 2 or CB063_W4_i = 1, go on with questions CB090_W4_i to CB093_W4_i, otherwise skip to CB065_i [XChildName[i]] CB063_i = 1, 2 CB063_W4_i = 1 CB090_W4_i CB093_W4_i CB065_i

CB090_W4_i Where does [XChildName[i]]s partner live? [XChildName[i]]

1. Living togher with [XChildName[i]] generally [XChildName[i]]
2. Other ______ (**CB090_W4_1_i**) Province ______ (**CB090_W4_2_i**) City ______ (**CB090_W4_3_i**) County ______ (**CB090_W4_4_i**) District/Town //, ______ (**CB090_W4_5_i**) Village/Community /

997. Have no idea
999. Refuse to answer this question

CB091_W4_i Without taking continuing education into account, whats [XChildName[i]] spartners highest achieved education? [XChildName[i]]

1. No formal education (illiterate)
2. Did not finish primary school
3. Sishu/home school
4. Elementary school
5. Middle school
6. High school
7. Vocational school ( )
8. Two-/Three-Year College/Associate degree
9. Four-Year College/Bachelors degree
10. Masters degree
11. Doctoral degree/Ph.D.

997. Have no idea
999. Refuse to answer this question

CB092_W4_i Does [XChildName[i]]s partner work? [XChildName[i]]

[IWER: By definition here, work means being a farmer, doing work for salary, self-employed or assisting in family business without getting paid]

1. Yes
2. No please specify what he/she has been doing /______ (**CB092_W4_1_i**) → Skip CB093_W4_i CB093_W4_i

997. Have no idea → Skip CB093_W4_i CB093_W4_i
999. Refuse to answer this question → Skip CB093_W4_i CB093_W4_i

CB093_W4_i What does [XChildName[i]]s partner do? [XChildName[i]]

1. Managers in an enterprise or the persons in charge of general affairs in government agency/ organization of political party/ public service organization
2. Professionals and technicians
3. Clerks
4. Workers in business and services
5. Workers in Agriculture, forestry, animal husbandry and fishery
6. Operating personnel of production/ transportation equipment
7. Others, please specify ______ (**CB093_W4_1_i**)

CB065_i How many childern does [XChildName[i]] have? [XChildName[i]] ______ Number

[CAPI: The answer could be dont know here]

**PROCEDURE** :
If CB065_i = 0, go directly to CB069_i CB065_i = 0 CB069_i

CB066_i Of those children, how many are under age of 16? [XChildName[i]] 16 ______ Number

[CAPI: The answer could be dont know here]

CB067_i How many grandchildren does [XChildName[i]] have? [XChildName[i]] ______

[CAPI: The answer could be dont know here]

**PROCEDURE** :
If CB067_i ≥ 1, go on with question CB068_i CB067_i ≥ 1, CB068_i

CB068_i Of those grandchildren, how many are under age of 16? [XChildName[i]] 16 ______

[CAPI: The answer could be dont know here]

CB069_i To which of the following buckets, does [XChildName[i]] and his/her partners aggregate annual income for last year belong? [XChildName[i]] /

1. 0
2. less than 2000 yuan 2
3. 2000-5000 yuan 2 5
4. 5000-10000 yuan 5 1

5. 10000-20000 yuan 1 2
6. 20000-30000 yuan 2 3
7. 30000-50000 yuan 3 5
8. 50000-100000 yuan 5 10
9. 100000-150000 yuan 10 15
10. 150000-200000 yuan 15 20
11. 200000-300000 yuan 20 30
12. More than 300000 yuan 30

997. Have no idea
999. Refuse to answer this question

CB071_W3_i Does [XChildName[i]] have any real estate property under his/her name? [XChildName[i]]

1. Yes
2. No → Skip CB072_W3_i CB072_W3_i

997. Have no idea → Skip CB072_W3_i CB072_W3_i
999. Refuse to answer this question → Skip CB072_W3_i CB072_W3_i

CB072_W3_i How much is this real eatate property worth now? [XChildName[i]] ______ (in 10000 yuan)

[CAPI: The answer could be dont know here]

CB063_W3_1_i What would you say about [XChildName[i]] s health status? [XChildName[i]]

1. Very good
2. Good
3. Fair
4. Poor
5. Very poor

997. Have no idea
999. Refuse to answer this question

PROCEDURE :

If [XChildName[i]] >=60, i.e. XChildBirth[i] ≤ 1958, go on with question CB063_W3_6_i

[XChildName[i]] 60 XChildBirth[i] ≤ 1958 CB063_W3_6_i

CB063_W3_6_i Does [XChildName[i]] have the ability to take care of him/herself? [XChildName[i]]

1. Yes
2. No

997. Have no idea
999. Refuse to answer this question

CC Sibling Information

CAPI:

CC section is regarding sibling information of [Name of the family respondent] and [Name of spouse of the family respondent] (If still alive). The family respondent and his/her spouse should answer questions regarding their own siblings. Answers to questions in this part will be saved under [Name of the family respondent]s ID CC [] [] ( ) [ ] [] CC [] ID

Codes for sibling of [Name of the family respondent] will follow rules as they are preloaded in CAPI. Any newly mentioned siblings will be coded after the preloaded ones. If the respondent is added to the survey for the first time, the codes for siblings will start from 1 [] 1

The following intermediate variables (series) will be generated, with the codes for siblings as subscript ( )

- XSibName a string variable, saving names of siblings
- XSibGender drop-down list provided, with 1 denoting male and 2 denoting female, saving genders of siblings 1 2
- XSibBirth an integer of 4 digits, saving the birth year of siblings
- XSibType drop-down list provided, with 1 denoting same-parents siblings of [Name of the family respondent], 2 denoting same-father half-blood siblings of [Name of the family respondent], 3 denoting same-mother half-blood siblings of [Name of the family respondent], 4 denoting siblings of [Name of the family respondent] with different father and different mother, 5 denoting not siblings of [Name of the family respondent] 1 [] 2 []
  3 [] 4 [] 5 []

[INTRO: In the following section, we would like to ask some questions about [Name of the family respondent]s siblings []]

PROCEDURE :

If this household has been already included in our previous survey (XRType = 1), and ZSibNum ≠ 0, skip to CC001_W4; XRType = 1 ZSibNum ≠ 0 CC001_W4

If this household has been already included in our previous survey (XRType = 1), and ZSibNum = 0, skip to CC002_W4_1 XRType = 1 ZSibNum = 0 CC002_W4_1

If this household is added into the survey for the first time (XRType = 2), questions CC000_W4_1 and CC000_W4 should be asked and then skip to procedures before CC003_W4_1 XRType = 2 CC000_W4_1 CC000_W4 CC003_W4_1 CAPI

CC000_W4_1 Including biological children and adoptive children of your parents/step-parent, How many siblings does [Name of the family respondent] have? [] ______

PROCEDURE :

If CC000_W4_1 ≥ 1, go on with question CC000_W4, otherwise skip all questions about [Name of the family respondent]s siblings CC000_W4_1 ≥ 1 CC000_W4 []

CC000_W4 Please ask the respondent the following questions regarding his/her siblings in the order from the oldest to the youngest

[CAPI: CC000_W4_1 is the number of rows/siblings; the option 5 not siblings of [Name of the family respondent] should not be available for choice for this question CC000_W4_1 5 []]

Serial number of siblings	(XSibName)	(XSibGender)	(XSibBirth)	(XSibAlive)	(XSibType)
1					
2					
...					
CC000_W4_1					

CC001_W4 Please confirm with the respondent the following information regarding his/her siblings in the order from the oldest to the youngest; If true, leave it as it is; if false, correct it accordingly in the following table

[IWER: Pleasedont skip to questions about another sibling before you finish asking/confirming all information of one particular sibling. Typos or other kinds of errors in sibling names may occur due to misunderstanding of answers given in dialect; in such cases, please check with the respondent carefully before you conclude that this sibling does not exist]

	(ZSibName)	(ZSibGender)	(ZSibBirth)	(ZSibAlive)	(ZSibType)
1					
2					
...					
ZSibNum					

[CAPI: Update the values of relevant Xsib series according to confirmation/ modification; dont generate variables for siblings classified as 5 not siblings of [Name of the family respondent] in ZsibType XSib ZSib- Type 5 [] XSib]

CC002_W4_1 In addition to [XSibName], does [Name of the family respondent] have any other siblings, including biological children and adoptive children of his/her parents/ step-parent? [XSibName][] ______ Number

**PROCEDURE** :<br>IfCC002_W4_1 ≥ 1, go on with question CC002_W4CC002_W4_1≥ 1CC002_W4

CC002_W4 Please give information about this/ these sibling(s) mentioned in CC002_W4_1 in the order from the oldest to the youngest [ ] CC002_W4_1
[CAPI: The number of rows in the below table is CC002_W4_1; and the serial number for siblings starts from ZSibNum+1 CC002_W4_1 ZSibNum+1]

	(XSibName)	(XSibGender)	(XSibBirth)	(XSibAlive)	(XSibType)
ZSibNum+1					
ZSibNum+2					
...					
ZSib+CC002_W4_1					

**CAPI**: For each sibling who has been dead, i.e. XSibAlive = 0, questions CC003_W4_1_i to CC003_W4__2__i should be asked iteratively, with i being the loop index XSibAlive = 0 CC003_W4_1_i CC003_W4_2_i i

**PROCEDURE** :<br>If ZSibDYear[i] is null, go on with question CC003_W4_2_i ZSibDYear[i] CC003_W4_2_i

CC003_W4_1_*i* When did [ZSibName[*i*]] pass away? [] [ZSibName[*i*]] ?

______ (**CC003_W4_1_1_*i***) Year or his/her age when he/she died ______ (**CC003_W4_1_2_*i***) Age

PROCEDURE :

If ZSibDReason[*i*] is null, go on with question CC003_W4_2_*i* ZSibDReason[*i*] CC003_W4_2_*i*

CC003_W4_2_*i* Whats the leading cause of death for [ZSibName[*i*]]? [] [ZSibName[*i*]] ______

[IWER: If the cause of death is illness, please specify the kind of illness. For example, if it is cancer, the specific type of cancer should be recorded (gastric cancer, lung cancer, etc.); if it is infectious disease, please specify the disease (tuberculosis, dysentery, etc.); or if it is an accident, please give information on the accident type (car accident, fire, intoxication, etc.) ( ) ( )]

CAPI: For each sibling (both the alive and the dead), questions CC003_W4_5_*i* to CC011_W4_*i* should be asked iteratively, with i being the loop index ( ) CC003_W4_5_*i* CC011_W4_*i* *i*

PROCEDURE :

If ZSibEdu[*i*] is not null, go on with question CC003_W4_5_*i* ZSibEdu[*i*] CC003_W4_5_*i*

CC003_W4_5_*i* Without taking continuing education into account, is [ZSibEdu[*i*]] [XSibName[*i*]s highest achieved education? [] [ZSibName[*i*]] [ZSibEdu[*i*]]

1. Yes → Skip to procedures before CC015_W3_*i* CC015_W3_*i* 2. No

CC003_W3_*i* Without taking continuing education into account, whats [ZSibName[*i*]]s highest achieved education? [] [ZSibName[*i*]]

1. No formal education (illiterate)
2. Did not finish elementary school
3. Sishu/home school
4. Elementary school
5. Middle school
6. High school
7. Vocational school ( )

8. Two-/Three-Year College/Associate degree
9. Four-Year College/Bachelors degree
10. Masters degree
11. Doctoral degree/Ph.D.
997. Have no idea
999. Refuse to answer this question

PROCEDURE :
If CC003_W3_i = 1, 2, 3, go on with question CC003_W3_0_i CC003_W3_i = 1, 2, 3, CC003_W3_0_i

CC003_W3_0_i Is [ZSibName[i]] literate? [] [ZSibName[i]] 1. Yes
2. No

CC015_W3_i Whats [ZSibName[i]]s highest achieved position in his/her career? [ ] [ZSibName[i]]
1. Managers in an enterprise or the persons in charge of general affairs in government agency/ organization of political party/ public service organization
2. Professionals and technicians
3. Clerks
4. Workers in business and services
5. Workers in Agriculture, forestry, animal husbandry and fishery
6. Operating personnel of production/transportation equipment
7. Others, please specify ______ (**CC015_W3_1_i**)

CC004_W3_i Is [ZSibName[i]] a member of the Communist Party? [] [ZSibName[i]]
1. Yes, Specify the year when he/she became a member of the Communist Party _____ (**CC004_W3_1_i**) Year
2. No
997. Have no idea
999. Refuse to answer this question
[CAPI: An answer of have no idea is allowed for the year]

CC016_W3_i Did [ZSibName[i]] have the experience to go down to the coutryside as an educated urban youth? [] [ZSibName[i]]

1. Yes, specify the year ______ (**CC016_W3_1_i**) Year
2. No → Skip CC016_W4_i CC016_W4_i

997. Have no idea → Skip CC016_W4_i CC016_W4_i
999. Refuse to answer this question → Skip CC016_W4_i CC016_W4_i

CC016_W4_i Did [ZSibName[i]] return to his/her home city/other urban areas after that movement? [] [ZSibName[i]] 1. Yes, specify the year ______ (**CC016_W4_1_i**) Year
2. No
997. Have no idea
999. Refuse to answer this question

CC011_W4_i How old was [ZSibName[i]] when he/she first married? [] [ZSibName[i]]
1. ______ (**CC011_W4_a_i**) At the age of
2. Never married
997. Have no idea
999. Refuse to answer this question

**CAPI**: For each sibling who is still alive, i.e. XSibAlive = 1, questions CC011_W3_i to CC006_W3__2__i should be asked iteratively, with i being the loop index （XSibAlive = 1） CC011_W3_i CC006_W3_2_i i

CC011_W3_i Whats [ZSibName[i]]s current marital status? [] [ZSibName[i]]
1. Married with spouse present → Skip CC011_W4_1_iCC011_W4_1_i 2. Married but not living with spouse temporarily for reasons such as work
→ Skip CC011_W4_1_i CC011_W4_1_i 3. Separated （）
4. Divorced
5. Widowed
6. Never married
997. Have no idea
999. Refuse to answer this question

CC011_W4_1_i Is there anyone who is living together with [ZSibName[i]] as his/her partner? [] [ZSibName[i]]
1. Yes
2. No
997. Have no idea
999. Refuse to answer this question

CC012_W3_i What would you say about [ZSibName[i]]s health status? [] [ZSibName[i]]
1. Very good

2. Good
3. Fair
4. Poor
5. Very poor
997. Have no idea
999. Refuse to answer this question

CC006_W3_2_*i* Does [ZSibName[*i*]] have the ability to take care of him/herself? [ ]
[ZSibName[*i*]]
1. Yes
2. No
997. Have no idea
999. Refuse to answer this question

CAPI:
If [Name of spouse of the family respondent] is still alive, we would also like to gather information on [Name of spouse of the family respondent]s siblings. The questions used are almost the same as those of [Name of the family respondent]s siblings, except that we would replace [Name of the family respondent] with [Name of spouse of the family respondent] in corresponding questions, choices, prefaces, and logics of procedures, and all generated variables in this part are further subscripted by an _s [ ] CC000_W4_1 CC006_W3_2_*i* [ ] [] [] s

A Household Member Information

[INTRO: We would like to know some information on your household members. By definition here, a household member is someone who lives together with you, and with whom you would share family income and expenses]

A001_W4 Of all people listed here, which are your household members? ( )
[IWER: By definition here, a household member is someone who lives together with you, and with whom you would share family income and expenses. During on-site interview, a particular difficulty is to determine whether a child is a household member of the family respondent or not. A trick that could be used during on-site interview is to inquire the respondent whether the child has been financially indenpendent from him/her]

1-8. CAPI will automatically load all parents of [Name of the family respondent] and [Name of spouse of the family respondent] who are still alive CAPI

9-33. CAPI will automatically load all children of [Name of the family respondent] and [Name of spouse of the family respondent] who are still alive CAPI

34-48. CAPI will automatically load all siblings of [Name of the family respondent] and [Name of spouse of the family respondent] who are still alive CAPI

99. None of people in above list

[CAPI: Updates the list of household members HHMember, in an order which is in accordance with the respondents answers to A001_W4 HHMember]

A002_W4_1 In addition to [HHMember], is there anyone else who is your household member? If any, please specify [HHMember]

1. ______
2. ______

... ______

[CAPI: Updates the list of household members HHMember once more in accordance with the respondents answers, adds any new ones sequentially HHMember]

PROCEDURE :
For each household member added from A002_W4_1, questions A005_W3_*i* to A008_W4_*i* should be asked iteratively, with *i* being the loop index HHMember A002_W4_1 A005_W3_*i* A008_W4_*i* *i*

A005_W3_*i* [HHMember[i]] is a [HHMember[*i*]]

1. Male
2. Female

A005_W4_*i* How old is [HHMember[*i*]] ? [HHMember[*i*]] ______ Age

A006_*i* [HHMember[*i*]] is [Name of the family respondent]s [HHMember[*i*]] [ ]

1. Daughter-in-law/ son-in-law
2. Grandchild
3. Brother-in-law
4. Father
5. Mother
6. Mother-in-law /
7. Father-in-law /

8. Child
9. Sibling
10. Carer/driver of this household
11. Other kind of relative, please specify ______ (**A006_1_i**)

PROCEDURE:
If A006_i = 1, go on with question A007_W4 A006_i = 1 A007_W4

A007_W4_i Which child of yours has [HHMember[i]] been married with? [HHMember[i]]

1-25. CAPI will automatically load names of all children, both the alive and dead ones CAPI ( )

99. Other, please specify the name ______ (**A007_W4_1_i**)

PROCEDURE:
If A006_i = 2, go on with question A008_W4 A006_i = 2 A008_W4

A008_W4_i Who is [HHMember[i]]s parent? [HHMember[i]]

1-25. CAPI will automatically load names of all children, both the alive and dead ones CAPI ( )

99. Other, please specify the name ______ (**A008_W4_1_i**)

C2 Time Transfer and Transfers

[INTRO: We would then like to ask some questions about interaction within your family, including contact between, financial support and care-giving provided to and received from your/your spouses parents, children and siblings]

CD Time Transfer

Contact with Parents

PROCEDURE:
All relationships are defined from the perspective of [Name of the family respondent] and [Name of spouse of the family respondent]. Questions CD001_W4 to CD002_W4 should only be asked for parents who are still alive and not household members. For parents who are a couple and at least one of which is household member, the couple is deemed as household members and questions CD001_W4 to CD002_W4 should be skipped [] [] / CD003_W4

LOOP:

Loop: Questions CD001_W4 to CD002_W4 should be asked iteratively for parents who are still alive and not household members. For each couple: If father and mother are still spouse of each other, the two should be treated as a whole and CAPI preloads both their names in each question; If father and mother are both still alive but no longer spouse of each other, the following questions should be asked separately for each of them; If only one of the couple is alive, the following questions should be asked just for the alive one [] [] / [XConParName] XParName [XConParName] XParName [XConParName] XParName .

CD001_W4 With whom is [XConParName] living together? [XConParName] ()

1. Living by him/herself
2. Living with his/her spouse/ partner /
3. Living together with [Name of the family respondent] []
4. Living with [Name of the family respondent]s siblings []
5. Living with [Name of spouse of the family respondent]s siblings [ ]
6. Living with [Name of the family respondent]s children []
7. Living with children of [Name of the family respondent]s siblings []
8. Living with children of [Name of spouse of the family respondent]s siblings [ ]
9. Living with other relatives
10. Livinng in nursing home
11. Living with someone who is not a relative, please specify ______ (**CD001_W4_1**)

PROCEDURE :

If CD001_W4 = 4, go on with question CD001_W3_1 CD001_W4 = 4[ ] CD001_W3_1

CD001_W3_1 Of [Name of the family respondent]s siblings, with whom is [XConParName] living together? (CAPIpreloads names of [Name of the family respondent]s siblings) [XConParName] [] ([])

PROCEDURE :

If CD001_W4 = 5, go on with question CD001_W3_2 CD001_W4 = 5[ ] CD001_W3_2

CD001_W3_2 Of [Name of spouse of the family respondent]s siblings, with whom is [XConParName] living together? (CAPI preloads names of [Name of spouse of the family respondent]s siblings) [XConParName] [] （ []）

PROCEDURE :

If CD001_W4 = 6, go on with question CD001_W3_3 CD001_W4 = 6[ ] CD001_W3_3

CD001_W3_3 Of [Name of the family respondent]s children, with whom is [XConParName] living together? (CAPI preloads names of [Name of the family respondent]s children) [XConParName] [] （ [] ）

PROCEDURE :

If CD001_W4 = 7, go on with question CD001_W3_4 CD001_W4 = 7[ ] CD001_W3_4

CD001_W3_4 Of [Name of the family respondent]s siblings, with whose children, is [XConParName] living together? (CAPI preloads names of [Name of the family respondent]s siblings) [XConParName] [] （ []）

PROCEDURE :

If CD001_W4 = 8, go on with question CD001_W3_5 CD001_W4 = 8[ ] CD001_W3_5

CD001_W3_5 Of [Name of spouse of the family respondent]s siblings, with whose children, is [XConParName] living together? (CAPIpreloads names of [Name of spouse of the family respondent]s siblings) [XConParName] [] （ []）

CD002_W4 When [XConParName] is not living with you, how often do you and/or your spouse go visit him/her? [XConParName]

1. Almost every day
2. 2-3 times a week 2-3
3. Once a week
4. Every two weeks
5. Once a month
6. Once every three months
7. Once every six months
8. Once a year
9. Almost never
10. Other

Contact with Children

**PROCEDURE** :
For each of [Name of the family respondent]s children who are still alive, questions CD003_W4 to CD004 should be asked iteratively, with i being loop index [ ] XChildAlive = 1 CD003_W4 CD004 i

CD003_W4_i During last year, how long had [XChildName[i]] lived with you and your spouse? (in month) [XChildName[i]] ______ Month

[IWER: A short visit does not constitute living together; input 0 here if not living together at all and 12 if always living together 0 12]

**PROCEDURE** :
If CD003_W4_i = 12, skip to the next child CD003_W4_i = 12

CD003_i When [XChildName[i]] is not living with you, how often do you contact with him/her on phone/by message/ on wechat/ by mail/ by email? [XChildName[i]] [XChildName[i]] ?

1. Almost every day
2. 2-3 times a week 2-3
3. Once a week
4. Every two weeks
5. Once a month
6. Once every three months
7. Once every six months
8. Once a year
9. Almost never
10. Other

**PROCEDURE** :
If CD003_i = 1, 2, 3, skip to the next child CD003_i = 1, 2, 3

CD004_i When [XChildName[i]] is not living with you, how often do you contact with him/her on phone/by message/ on wechat/ by mail/ by email? [XChildName[i]] [XChildName[i]] ?

1. Almost every day
2. 2-3 times a week 2-3
3. Once a week
4. Every two weeks
5. Once a month
6. Once every three months
7. Once every six months

8. Once a year
9. Almost never
10. Other

CE Transfers

[INTRO: From time to time, family members will provide help or support each other in various forms, and each form of support matters. And in the following section, here are some questions about the financial support you and your spouse received and provided]

Financial Support Received from and Provided to Parents

**PROCEDURE** :<br>Questions CE002 to CE023 should be asked iteratively for parents in the list of [XConParName] [XConParName] CE002 CE023

CE002 During last year, whats the amount of financial support received from [XConParName] when he/she/they was/were not living with you? [XConParName] [XConParName]

1. Money received in total ______ (**CE002_1**) Yuan of which regular payment was ______ (**CE002_2**) Yuan (Regular payment includes providing living expenses, paying for water, electricity or telephone bill, paying for mortgage/ rent or other forms of regular expenses) (/)
2. In-kind payment received worth in total ______ (**CE002_3**) Yuan of which regular payment was ______ **CE002_4**) Yuan (For example, buying food, clothes or other stuff regularly for you) ( )

[IWER: Regular payments are payments occurring monthly, quarterly, semi-annually, annually, in cash or in-kind, at some fixed points of time; If nothing received, input 0 here; if an answer of have no idea is given or no answer is provided, input -1 here 0-1]

CE003 If the answer for CE002_1, CE002_2, CE002_3 or CE002_4 is have no idea or the respondent refused to answer those questions, please further ask about these questions by giving choices of number buckets (100, 200, 400, 800, 1600 yuan) CE002_1, CE002_2, CE002_3 CE002_4 (100, 200, 400, 800, 1600)

CE022 During last year, whats the amount of financial support provided to [XConParName] when he/she/they was/were not living with you? [XConParName] [XConParName]

1. Money received in total ______ (**CE022_1**) Yuan of which regular payment was ______ (**CE022_2**) Yuan (Regular payment includes providing living expenses, paying for water, electricity or telephone bill, paying for mortgage/ rent or other forms of regular expenses) （ /）
2. In-kind payment provided worth in total ______ (**CE022_3**) Yuan of which regular payment was ______ (**CE022_4**) Yuan (For example, buying food, clothes or other stuff regularly for you)（ ）

[IWER: Regular payments are payments occurring monthly, quarterly, semi-annually, annually, in cash or in-kind, at some fixed points of time; If nothing received, input 0 here; if an answer of have no idea is given or no answer is provided, input -1 here 0-1]

CE023 If the answer for CE022_1, CE022_2, CE022_3 or CE022_4 is have no idea or the respondent refused to answer those questions, please further ask about these questions by giving choices of number buckets (100, 200, 400, 800, 1600 yuan) CE022_1, CE022_2, CE022_3 CE022_4 （100, 200, 400, 800, 1600 ）

Financial Support Received from and Provided to Children

PROCEDURE :

Questions CE009 to CE030 should be asked iteratively for children who are still alive and not household member of [Name of the family respondent] (i.e. XChildAlive = 1 and CD003_W4 < 12) [] 12 XChildAlive = 1 CD003_W4 < 12 CE009 CE030

CE009 During last year, whats the amount of financial support received from [XChildName] when he/she was not living with you? [XChildName] [XChildName]

[IWER: Financial support received from [XChildName]s children should be included here [XChildName]]

1. Money received in total ______ (**CE009_1**) Yuan of which regular payment was ______ (**CE009_2**) Yuan (Regular payment includes providing living expenses, paying for water, electricity or telephone bill, paying for mortgate/ rent or other forms of regular expenses)（ /）
2. In-kind payment received worth in total ______ (**CE009_3**) Yuan of which regular payment was ______ (**CE009_4**) Yuan (For example, buying food, clothes or other stuff regularly for you)（ ）

[IWER: Regular payments are payments occuring monthly, quarterly, semi-annully, annually, in cash or in kind, at some fixed points of time; If nothing received, input 0 here; if an answer of have no idea is given or no answer is provided, input -1 here 0-1]

CE010 If the answer for CE009_1, CE009_2, CE009_3 or CE009_4 is have no idea or the respondent refused to answer those questions, please further ask about these questions by giving choices of number buckets (100, 200, 400, 800, 1600 yuan) CE009_1, CE009_2, CE009_3 CE009_4 （100, 200, 400, 800, 1600 ）

CE029 During last year, whats the amount of financial support provided to [XChildName] when he/she was not living with you? [XChildName] [XChildName]

1. Money provided in total ______ (**CE029_1**) Yuan of which regular pay- ment was __ (**CE029_2**) Yuan (Regular payment includes pro- viding living expenses, paying for water, electricity or telephone bill, paying for mortgage/ rent or other forms of regular expenses)（ /）
2. In-kind payment provided worth in total ______ (**CE029_3**) Yuan of which regular payment was ______ (**CE029_4**) Yuan (For example, buying food, clothes or other stuff regularly for you)（ ）

[IWER: Regular payments are payments occurring monthly, quarterly, semi-annually, annually, in cash or in-kind, at some fixed points of time; If nothing received, input 0 here; if an answer of have no idea is given or no answer is provided, input -1 here] [0-1]

CE030 If the answer for CE029_1, CE029_2, CE029_3 or CE029_4 is have no idea or the respondent refused to answer those questions, please further ask about these questions by giving choices of number buckets (100, 200, 400, 800, 1600 yuan) CE029_1, CE029_2, CE029_3 CE029_4 （100, 200, 400, 800, 1600 ）

Financial Support Received from and Provided to Siblings

CE072_W3 During last year, did you/your spouse ever receive any financial support from or provide financial support to any of your/ your spouses siblings?

1. CAPI preloads names of [Name of the family respondent]s and [Name of spouse of the family respondent]s siblings who are still alive [] [ ]

99. None of the above → Skip to CE016_W4 CE016_W4

PROCEDURE :
Questions CE072_W2 to CE075_W2 should be asked iteratively for each sibling chosen in CE072_W3 CE072_W3 CE072_W2 CE075_W2

CE072_W2 During last year, whats the amount of financial support received from [name of sibling_i]?

1. Money received in total ______ (**CE072_W2_1**) Yuan (Money received is cash gifts for marriage, funeral, migration to new home, child birth or children schooling, or financial support for illness/ difficulty, but any kind of borrowing should be excluded) ( ) of which regular payment was ______ (**CE072_W2_2**) Yuan (Regular payment includes providing living expenses, paying for water, electricity or telephone bill, paying for mortgage/ rent or other forms of regular expenses) (/)
2. In-kind payment received worth in total ______ (**CE072_W2_3**) Yuan of which regular payment was ______ (**CE072_W2_4**) Yuan (For ex- ample, buying food, clothes or other stuff regularly for you) ( )

[IWER: Regular payments are payments occurring monthly, quarterly, semi-annually, annually, in cash or in-kind, at some fixed points of time; If nothing received, input 0 here; if an answer of have no idea is given or no answer is provided, input -1 here 0-1]

CE073_W2 If the answer for CE072_W2_1, CE072_W2_2, CE072_W2_3 or CE072_W2_4 is have no idea or the respondent refused to answer those questions, please further ask about these questions by giving choices of number buckets (100, 200, 400, 800, 1600 yuan) CE072_W2_1CE072_W2_2CE072_W2_3 CE072_W2_4 (100, 200, 400, 800, 1600)

CE074_W2 During last year, whats the amount of financial support provided to [name of sibling_i]?

1. Money provided in total ______ (**CE074_W2_1**) Yuan (Money received is cash gifts for marriage, funeral, migration to a new home, childbirth or children schooling, or financial support for illness/ difficulty, but any kind of borrowing should be excluded) ( ) of which regular payment was ______ (**CE074_W2_2**) Yuan (Regular payment includes providing living expenses, paying for water, electricity or telephone bill, paying for mortgage/ rent or other forms of regular expenses) (/)

2. In-kind payment provided worth in total ______ (**CE074_W2_3**) Yuan of which regular payment was ______ (**CE074_W2_4**) Yuan (For example, buying food, clothes or other stuff regularly for you) ( )

[IWER: Regular payments are payments occurring monthly, quarterly, semi-annually, annually, in cash or in-kind, at some fixed points of time; If nothing received, input 0 here; if an answer of have no idea is given or no answer is provided, input -1 here 0-1]

CE075_W2 If the answer for CE074_W2_1, CE074_W2_2, CE074_W2_3 or CE074_W2_4 is have no idea or the respondent refused to answer those questions, please further ask about these questions by giving choices of number buckets (100, 200, 400, 800, 1600 yuan) CE074_W2_1CE074_W2_2CE074_W2_3 CE074_W2_4 (100, 200, 400, 800, 1600)

Financial Support Received from and Provided to Other Relatives and Friends

CE016_W4 During last year, how much did you/ your spouse spend in inviting others to dinner for marriage, funeral, migration to new home, childbirth or children schooling? _____ Yuan

[IWER: if no such expense, input 0 here; if the answer is dont know or the respondent refused to answer this question, input -1 here 0 -1]

CE017_W4 If the answer for CE016_W4 is have no idea or the respondent refused to answer this question,please further ask about this question by giving choices of number buckets (500, 1000, 2000, 5000, 10000 yuan) CE016_W4 (500, 1000, 2000, 5000, 10000)

CE016_W3 During last year, did you/your spouse ever receive cash gifts from any other relatives or friends except for parents/ children/ siblings? Or any in-kind giving? ______ Yuan (gifts in cash or in-kind are gifts for marriage, funeral, migration to new home, childbirth or children schooling) ( )

[IWER: if no such receiving, input 0 here; if the answer is dont know or the respondent refused to answer this question, input -1 here 0 -1]

CE017_W3 If the answer for CE016_W3 is have no idea or the respondent refused to answer this question,please further ask about this question by giving choices of number buckets (500, 1000, 2000, 5000, 10000 yuan) CE016_W3 （500, 1000, 2000, 5000, 10000 ）

CE036_W3 During last year, did you/your spouse ever give cash gifts to any other relatives or friends except for parents/ children/ siblings? Or any in-kind giving? ___ Yuan (gifts in cash or in-kind are gifts for marriage, funeral, migration to new home, childbirth or children schooling)（ ）

[IWER: if no such receiving, input 0 here; if the answer is dont know or the respondent refused to answer this question, input -1 here 0 -1]

CE037_W3 If the answer for CE036_W3 is have no idea or the respondent refused to answer this question,please further ask about this question by giving choices of number buckets (100, 200, 400, 800, 1600 yuan) CE036_W3 （100, 200, 400, 800, 1600 ）

CE016 During last year, in addition to gifts received mentioned above, did you/your spouse ever receive other financial support from any other relatives or friends except for parents/ children/ siblings? Or any in-kind receiving? ______ Yuan (financial support for illness/ difficulty, but any kind of borrowing should be excluded)（ ）

[IWER: if no such financial support, input 0 here; if an answer of have no idea is given or no answer is provided, input -1 here 0 -1]

CE017 If the answer for CE016 is have no idea or the respondent refused to answer this question, please further ask this question by giving choices of number buckets (100, 200, 400, 800, 1600 yuan) CE016 （100, 200, 400, 800, 1600 ）

CE036 During last year, in addition to gifts given mentioned above, did you/your spouse ever give other financial support to any other relatives or friends except for parents/ children/ siblings? Or any in-kind giving? ______ Yuan (financial support for illness/ difficulty, but any kind of lending should be excluded)（ ）

[IWER: if no such financial support, input 0 here; if an answer of have no idea is given

or no answer is provided, input -1 here 0 -1]

CE037 If the answer for CE036 is have no idea or the respondent refused to answer this question, please further ask this question by giving choices of number buckets (100, 200, 400, 800, 1600 yuan) CE036 （100, 200, 400, 800, 1600 ）

Marriage Gifts

**PROCEDURE**:
Questions CE066_W2 to CE070_W2_1 should be asked iteratively for each children who are still alive and ever been married (i.e. XChildAlive = 1 and CB063 = 1, 2, 3, 4, 5) XChildAlive = 1 CB063 = 1, 2, 3, 4, 5 CE066_W2 - CE070_W2_1

CE066_W2 When did [XChildName] first get married? [XChildName] ___ [1900...2018] (**CE066_W2_1**) Year ___ [0...12] (**CE066_W2_2**) Month

CE067_W2_1 Did you give any marriage gifts when [XChildName] first get married? [XChild-Name]

1. Yes
2. No → Skip to CE069_W2_1 CE069_W2_1

CE068_W2_1 What were these gifts worth at the price level at that time? ______ Yuan

CE069_W2_1 Did you buy [XChildName] a home when he/she first get married? [XChildName]

1. Yes
2. No → Skip to next child or skip to CF001 for the last child CF001

CE070_W2_1 How much did the house/flat cost you at that time? ______ (In 10000 yuan)
[IWER: input the amount in 10000]

CF Time Spent Providing Care

CF001 During last year, did you/ your spouse spend time in taking care of your grandchildren?

1. Yes
2. No → Skip to procedures before CF004_W4 CF004_W4

3. Have no grandchild → Skip to procedures before CF004_W4 CF004_W4

CF002 During last year, whose children did you take care of?

1. CAPIpreloads names of all children of the respondent, both the alive and dead ones

99. None of the above → Skip to procedures before CF004_W4 CF004_W4

> **PROCEDURE** :
> For each children name chosen, question CF003 should be asked iteratively CF002 CF003

CF003 During last year, how many weeks did you spend in taking care of [XChildName]s children? And on average, how many hours per week? [XChildName]

I ______ (**CF003_1**) Weeks ______ (**CF003_2**) Hours per week

My spouse ______ (**CF003_3**) Weeks ______ (**CF003_4**) Hours per week

[IWER: input 0 here if no time spending in taking care of grandchildren; and 1 if less than 1 week last year and 1 for less than 1 hour per week 0 11]

[Hardcheck: For answers of greater than 52 weeks or 140 hours per week, please check for any error 52 140]

> **PROCEDURE** :
> For each parent in the list of [XConParName], questions CF004_W4 to CF006_W4 should be asked iteratively [XConParName] CF004_W4 CF006_W4

CF004_W4 During last year, did you/ your spouse ever help [XConParName] with dealing with everyday activities, such as doing household work, cooking, doing laundry, shopping or financial management? [] [] ( ) [XConParName] ( )

1. Yes
2. No → Skip to the next parent

CF005_W4 During last year, how many weeks did you ([Name of the Family Respondent]) spend in taking care of [XConParName]? And on average, how many hours per week? [] [XConParName]

______ (**CF005_W4_1**) Weeks ______ (**CF005_W4_2**) Hours per week

[IWER: input 0 here if no time spending in taking care of parent; and 1 if less than 1 week

last year and 1 for less than 1 hour per week 0 11]

[Hardcheck: For answers of greater than 52 weeks or 140 hours per week, please check for any error 52 140]

CF006_W4 During last year, how many weeks did your spouse ([Name of spouse of the Family Respondent]) spend in taking care of [XConParName]? And on average, how many hours per week? [] [XConPar- Name]

_______ (**CF006_W4_1**) Weeks _______ (**CF006_W4_2**) Hours per week

[IWER: input 0 here if no time spending in taking care of parent; and 1 if less than 1 week last year and 1 for less than 1 hour per week 0 11]

[Hardcheck: For answers of greater than 52 weeks or 140 hours per week, please check for any error 52 140]

CF007_W2 Has the respondent ever turned to others for help when answering the questions in this part?

[IWER: If the family respondent was assisted by others, please record the respondents response to such answers; if the family respondent was away from home or couldnt answer questions for other reasons, select 4 here if questions were answered by family respondents spouse 4]

1. Never
2. A few times
3. Most or all of the time
4. Proxy by the spouse

D Health Status and Functioning

Health Conditions Reported in Last Wave Interview

**Zdisability[i]**	If Zdisability[i] =1 Yes, R had kind of disabilities listed in DA005 at ZIWTime
**ZDA006[i]**	If ZDA006[i] =1 Yes, R reported disabled time at ZIWTime
**Zdiagnosed[i]**	If Zdiagnosed[i] =1 Yes, R had kind of doctor diagnosed disease at ZIWTime
**Zdisease[i]**	If Zdisease[i] =1 Yes, R had kind of disease at ZIWTime
**ZDA008[i]**	If ZDA008[i] =1 Yes, R had known R had kind of disease at ZIWTime
**ZDA009[i]**	If ZDA009[i] =1 Yes, R had answered When was the condition first dignosed or known by yourself? at ZIWTime
**Zmenopause**	If Zmenopause =1 Yes, R had started menopause at Rs last interview
**ZDA030**	If ZDA030 =1 Yes, R had answered When was the condition first diagnosed at ZIWTime
**Zcataract**	If Zcataract =1 Yes, R had cataract surgery at ZIWTime
**ZcataractNUM**	If ZcataractNUM = 1, R had cataract surgery for one eye at ZIWTime
**Zglaucoma**	If Zglaucoma =1 Yes, R had Glaucoma at ZIWTime
**Ztooth**	If Ztooth =1 Yes, R had lost all teeth at ZIWTime
**Zsmoke**	If Zsmoke =1 Yes, R had ever smoked at Rs last interview
**ZDA065**	If ZDA065 =1 Yes, R had answered At what age did you start to smoke on a regular basis? at ZIWTime
**ZDA069**	Did you ever drink alcoholic beverages in the past in last IW?
**ZDA070**	If ZDA070 =1 Yes, R had answered When did you quit or reduce drinking? at ZIWTime
**ZDA071**	If ZDA071 =1 Yes, R had answered When did you start drinking? at ZIWTime

DA Health Status

PART I General Health Status and Disease History

DA002 Next,I have some questions about your health. Would you say your health is very good, good, fair, poor or very poor?

[IWERInterviewer should read all the following options]

1. Very good
2. Good
3. Fair
4. Poor
5. Very poor

997 Dont know

PROCEDURE :

If XRType = REIW, ask DA002_W2_1 DA002_W2_1

DA002_W2_1 Compared with your health when we talked with you in Rs LAST IW MONTH, YEAR, would you say that your health is better now, about the same, or worse? []

1. Better
2. About the same
3. Worse

997 Dont know

PROCEDURE :

If XRType = REIW who reported disability and the year became disabled in last iw time (Zdisability[i] = 1) and (ZDA006[i] ≠ null),skip to DA007 DA007

If XRType = REIW who had disability but did not report the year became disabled in last iw time (Zdisability[i] = 1) and (ZDA006[i] = null),skip to DA006_W4 DA006_W4

If this is a new interview R or this is a reinterview R who did not report yes in last wave (Zdisability[i] ≠ 1), go to DA005 DA005

DA005 Do you have one of the following disabilities?

DA005[1] Do you have Physical disabilities

1. Yes
2. No

DA005[2] Do you have Brain damage/intellectual disability /

1. Yes
2. No

DA005[3] Do you have Vision problem

1. Yes
2. No

DA005[4] Do you have Hearing problem

1. Yes
2. No

DA005[5] Do you have Speech impediment

1. Yes
2. No

PROCEDURE :

If DA005[i] = 1, ask DA006[i] DA006[i]

DA006[i] In what year did you become disabled? DA005[i]______ Year

[IWER: Mark the year using four digits 4]

→ Skip to procedure before DA007 DA007

DA006_W4 Our records from your last interview show that you have had [Zdisability[i] disability name], In what year did you become disabled? [Zdisability[i]] [Zdisability[i]] 1. ______ (**DA006_W4_1**) Year

99. Have never had the disability

[IWER: Mark the year using four digits. 4]

PROCEDURE :

If XRType = NEIW or XRType = REIW and REIW R not diagnosed with chronic disease, ask DA007 Zdiagnosed[i] = . DA007

DA007 Have you been diagnosed with [conditions listed below, read one by one] by a doctor?

DA007_1_ Have you been diagnosed with Hypertension by a doctor?

1. Yes
2. No

DA007_2_ Have you been diagnosed with Dyslipidemia (elevation of low density lipoprotein, triglycerides (TGs),and total cholesterol, or a low high density lipoprotein level) by a doctor? （）

1. Yes
2. No

DA007_3_ Have you been diagnosed with Diabetes or high blood sugar by a doctor? ( )

1. Yes
2. No

DA007_4_ Have you been diagnosed with Cancer or malignant tumor (excluding minor skin cancers) by a doctor? ( )

1. Yes
2. No

DA007_5_ Have you been diagnosed with Chronic lung diseases, such as chronic bronchitis , emphysema (excluding tumors, or cancer) by a doctor? ( )

1. Yes
2. No

DA007_6_ Have you been diagnosed with Liver disease (except fatty liver, tumors, and cancer) by a doctor? ( )

1. Yes
2. No

DA007_7_ Have you been diagnosed with Heart attack, coronary heart disease, angina, congestive heart failure, or other heart problems by a doctor? ( )

1. Yes
2. No

DA007_8_ Have you been diagnosed with Stroke by a doctor? ( )

1. Yes
2. No

DA007_9_ Have you been diagnosed with Kidney disease (except for tumor or cancer) by a doctor? ( )

1. Yes
2. No

DA007_10_ Have you been diagnosed with Stomach or other digestive diseases (except for tumor or cancer) by a doctor? ( )

1. Yes
2. No

DA007_11_ Have you been diagnosed with Emotional, nervous, or psychiatric problems by a doctor?

1. Yes
2. No

DA007_12_ Have you been diagnosed with Memory-related disease (such as dementia, brain atrophy, and Parkinsons disease) by a doctor?

( )

1. Yes
2. No

DA007_13_ Have you been diagnosed with Arthritis or rheumatism by a doctor?

1. Yes
2. No

DA007_14_ Have you been diagnosed with Asthma by a doctor? 1. Yes

2. No

PROCEDURE :

If DA007[1,5,11] ≠ 1 and ZDA008 ≠ 1, ask DA008 DA007 1511 ZDA008 ≠ 1 DA008

DA008 Do you know if you have [preload the current choice in DA007 [1,5,11]]? [DA007 1511]

1. Yes, I know I have this disease
2. No, I dont I have this disease
3. I dont know if I have this disease

PROCEDURE :

If DA007[i] = 1 or DA008[i] = 1, ask DA008_W2_1 DA008_W2_1

DA008_W2_1 How did you know that you had had [preload disease], through routine or charls physical examination, or any other? [] [] charls

1. Physical examination after had [preload disease] attack [] 2. Physical examination after had ill
3. Physical examination organized by work unit
4. Physical examination organized by community
5. CHARLS physical examination charls
6. Other, please specify ______ (**DA008_W2_1_1**)

DA009 BRANCHPOINT:

If DA007[i] = 1 or DA008[i] = 1 or (Zdisease[i] = 1 and ZDA009[i] = null), ask DA009 (DA007[i] = 1 DA008[i] = 1 (Zdisease[i] = 1 ZDA009[i] = null)) DA009

DA009 When was the condition first diagnosed or known by yourself? []

1. ______ (**DA009_1**) Year ______ (**DA009_2**) Age

99. Have never had the disease
[IWER: Mark the year using four digits : 4]

PROCEDURE :
If (DA007[2,5,6,7,9,10,12,13] = 1 or DA008[5] = 1 or Zdisease[2,5,6,7,9,10,12,13] = 1)ask DA010_W4 DA007 25679101213 (DA007[2,5,6,7,9,10,12,13] = 1 DA008[5] = 1 Zdisease[2,5,6,7,9,10,12,13] = 1) DA010_W4

DA010_W4 Are you now taking any of the following treatments to treat [preload health condition] or its complications (Check all that apply)? Taking Chinese traditional medicine, taking Western modern medicine, other treatments? [] () ()
[IWER: Read one by one]
1. Taking Chinese traditional medicine
2. Taking Western modern medicine
3. Other treatments, please specify ______ (**DA010_W4_1**) 4. None of the above
99. Have never had the disease

PROCEDURE :
Ask DA010_W2_1if you have hypertension or diabetes （DA007[1] = 1 DA008[1] = 1 Zdisease[1] = 1）（DA007[3] = 1 Zdisease[3] = 1) DA010_W2_1

DA010_W2_1 Is your [Blood pressure/ sugar] generally under control? [/]

1. Yes
2. No
3. Dont know
99. Have never had the disease

PROCEDURE :
If XRType = REIW that had chronic diesease in last wave (Zdisease[i] = 1), ask DA010_W2_2 （Zdisease[i] = 1） DA010_W2_2

DA010_W2_2 Compared to when we interviewed you in [ZIWTime], is your [preload heatlh condition] better, about the same as it was then or worse? ( ) []
1. Better
2. Worse

3. About the same
99. Have never had the disease

**PROCEDURE**:
If respondents have hypertension, then ask DA011_W4 - DA013 （DA007[1] = 1 DA008[1] = 1 Zdisease[1] = 1） DA011_W4 - DA013 If DA010_W2_1[1] = 99 or DA010_W2_2[1] = 99, skip DA011_W4 - DA013 DA010_W2_1[1] DA010_W2_2[1] 99DA011_W4 - DA013

DA011_W4 Are you now taking any of the following treatments to treat or control your hypertension?(Check all that apply) Taking Chinese traditional medicine, taking Western modern medicine? （）

[IWER: Read one by one and let R answer]

1. Taking Chinese traditional medicine
2. Taking Western modern medicine
3. Other treatments, please specify ______(**DA011_W4_a**) 4. None of the above

[Soft check: If DA011_W4 = 4 None of the above and DA010_W2_1[1] = 1, remind IWER You just said your hypertension is under control. Please confirm whether you are taking any treatments. Or revise the response to DA010_W2_1 DA011_W4 4 DA010_W2_1[1] = 1 DA010_W2_1]

**PROCEDURE**:
If XRType = NEWIW, ask DA011_W2_1 DA011_W2_1 If XRType = REIW, ask DA011_W4_1 DA011_W4_1

DA011_W2_1 Have you ever had your blood pressure checked by a doctor or nurse?

1. Yes
2. No → Skip to DA013 DA013

DA011_W4_1 Since [ZIWTime], have you had your blood pressure checked by a doctor or nurse? []

1. Yes
2. No → Skip to DA013 DA013

DA011_W2_2 When did you last have it checked?

______ (**DA011_w2_2_1**) Year ______ (**DA011_w2_2_2**) Month

DA012 During last year (last 12 months), how many times have you had blood pressure examination? （12 ） _____ [0 ... 999] Times

DA012_W4 During the last year (last 12 months), have you had blood pressure examination by community/village doctors regularly? /

1. Yes
2. No → Skip to DA013 DA013

DA012_W3_1 How often did you have blood pressure examination by community/village doctors? /

1. Once a week
2. Once half a month
3. Once a month
4. Once every two months
5. Once every three months
6. Once half a year
7. Once a year

DA012_W3_2 Do you have to pay for the blood pressure examination by community/village doctors? /

1. Yes
2. No

DA013 Have your care providers ever given you health education/advice on the following (check all that apply)? Weight control, exercise, diet and/or smoking control? （）

[IWER: Read one by one and let R answer]

1. Weight control
2. Exercise
3. Diet
4. Smoking control
5. None of the above

PROCEDURE

If respondents have diabetes, then ask DA014_W4 - DA016 （DA007[3] = 1 Zdisease[3] = 1） DA014_W4 - DA016

if DA010_W2_1[2] = 99 or DA010_W2_2[2] = 99, skip DA014_W4 - DA016 DA010_W2_1[2] DA010_W2_2[2] 99DA014_W4 - DA016

DA014_W4 Are you now taking any of the following treatments to treat or control your diabetes? (check all that apply) （）

[IWER: Read one by one]

1. Taking Chinese traditional medicine
2. Taking Western modern medicine
3. Taking insulin injections
4. Other treatments, please specify ______ (**DA014_W4_1**) 5. None of the above

[Soft check: If DA014_W4 = 5 None of the above and DA010_W2_1[3] = 1, remind IWER You just said your diabetes is under control. Please confirm whether you are taking any treatments. Or revise the response to DA010_W2_1 DA014_W45 DA010_W2_1[3] = 1DA010_W2_1]

DA015 During last year (last 12 months), how many times have you had the following? （12 ）

1. Blood glucose test ______ (**DA015_1**) 0...999 Times
2. Urine glucose test ______ (**DA015_2**) 0...999 Times
3. Fundus examination ______ (**DA015_3**) 0...999 Times
4. Micro-albuminuria test ______ (**DA015_4**) 0...999 Times 5. None of the above → Skip to DA016 DA016

DA016_W4 During the last year (last 12 months), have you had diabetes examination by community/village doctors regularly? /

1. Yes
2. No → Skip DA016_W3_1 and DA016_W3_2 DA016_W3_1 DA016_W3_2

DA016_W3_1 How often did you have diabetes examination by community/village doctors? /

1. Once a week
2. Once half a month
3. Once a month
4. Once every two months
5. Once every three months
6. Once half a year
7. Once a year

DA016_W3_2 Do you have to pay for diabetes examination done by community/village doctors? /

1. Yes
2. No

DA016 Have your care providers ever given you health education/advice on the following? (check all that apply) ( )

[IWER: Read one by one and let R answer]

1. Weight control
2. Exercise
3. Diet
4. Smoking control
5. Foot self-care
6. None of the above

PROCEDURE

If reinterview respondents have heart attack (Zdisease[7] = 1), then ask DA007_W2_5 （Zdisease[7]=1）DA007_W2_5

If DA010_W2_2[7] = 99, skip DA007_W2_5 - DA007_W2_6 DA010_W2_2[7] 99 DA007_W2_5 - DA007_W2_6

DA007_W2_5 Since [ZIWTime], have you had a heart attack? []

1. Yes
2. No → Skip DA007_W2_6 DA007_W2_6

DA007_W2_6 When was the (mostrecent) heart attack? _____ (**DA007_W2_6_1**) Year ______ (**DA007_W2_6_2**) Age

PROCEDURE

If respondents have cancer or maligant tumor (excluding minor skin cancers) (DA007[4] = 1 or Zdisease[4] = 1), ask DA017 and DA018_W4 （ ）（DA007[4] = 1 Zdisease[4] = 1） DA017 DA018_W4

If DA010_W2_2[4] = 99, skip DA017 and DA018_W4 DA010_W2_2[4] 99 DA017 DA018_W4

DA017 In which organ or part of your body do you have cancer? Including the origins and metastasis of tumor.（check all that apply） （）

[IWER: Read one by one. We should still ask R even if he/she has already been cured]

1. Brain
2. Oral cavity
3. Larynx
4. Other pharynx
5. Thyroid
6. Lung
7. Breast
8. Oesophagus
9. Stomach
10. Liver

11. Pancreas
12. Kidney
13. Prostate
14. Testicle
15. Ovary
16. Cervix
17. Endometrium
18. Colon or rectum
19. Bladder
20. Skin
21. Non-Hodgkin lymphoma （）
22. Leukemia
23. Other organ ______ (**DA017_1**)

DA018_W4 Have you taken any of the following treatments to treat your cancer or relieve its/their symptoms (e.g., pain, nausea, etc.) in the past two years? (Check all that apply) Taking Chinese traditional medicine, taking Western modern medicine, chemotherapy, surgery, radiation therapy? （）

[IWER: Read one by one and let R answer]

1. Taking Chinese traditional medicine
2. Taking Western morden medicine
3. Chemotherapy
4. Surgery
5. Radiation therapy
6. Other treatments, please spcify ______ (**DA018_W4_1**) 7. None of the above

[F1 (1) （）

（2） （3）]

**PROCEDURE**<br>If respondents have stroke (DA007[8] = 1 or Zdisease[8] = 1), then ask DA019_W4 （ DA007[8] = 1 Zdisease[8] = 1） DA019_W4<br>If DA010_W2_2[8] = 99, skip DA019_W4 - DA019_W2_2 DA010_W2_2[8] 99 DA019_W4 - DA019_W2_2

DA019_W4 Are you now taking any of the following treatments because of your stroke?(Check all that apply) Taking Chinese traditional medicine, taking Western modern medicine, physical therapy, acupuncture and moxibustion, occupational therapy?

( )

[IWER: Read one by one and let R answer]

1. Taking Chinese traditional medicine
2. Taking Western morden medicine
3. Physical therapy
4. Acupuncture and moxibustion
5. Occupational therapy
6. Other treatments, please spcify ______(**DA019_W4_1**) 7. None of the above

[F1 (1) ( )

(2)

(3) / /]

PROCEDURE
If reinterview respondents have stroke, then ask DA019_W2_1 and DA019_W2_2 (Zdisease[8] = 1) DA019_W2_1 DA019_W2_2

DA019_W2_1 Since Rs LAST IW MONTH, YEAR, has a doctor told you that you had another stroke? []

1. Yes
2. No → Skip DA019_W2_2 DA019_W2_2

DA019_W2_2 When was your most recent stroke? . (**DA019_W2_2_1**) Year _____ (**DA019_W2_2_2**) Age

PROCEDURE
If respondents have emotional, nervous, or psychiatric problems (DA007[11] = 1 or DA008[11] = 1 or Zdisease[11] = 1), then ask DA020_W4 (DA007[11] = 1 DA008[11] = 1 Zdisease[11] = 1) DA020_W4<br>If DA010_W2_2[11] = 99 never have this disease, skip DA020_W4 DA010_W2_2[11] 99 DA020_W4

DA020_W4 Are you now taking any of the following treatments for your emotional, nervous, or psychiatric problems?(Check all that apply) Receiving psychiatric or psychological treatment, taking anti depressants, taking tranquilizers or sleeping pills?

( )

[IWER: Read one by one and let R answer]

1. Receiving psychiatric or psychological treatment 2. Taking anti depressants
3. Taking tranquilizers or sleeping pills
4. Other treatments, please specify ______ (**DA020_W4_1**) 5. None of the above

**PROCEDURE**
If XRType = NEWIW, ask DA021 DA021
If XRType = REIW, ask DA021_W4 DA021_W4

DA021 Have you ever been in a traffic accident or any other kind of major accidental injury and received medical treatment?

1. Yes
2. No → Skip to procedure before DA023 DA023

DA021_W4 Have you ever been in a traffic accident or any other kind of major accidental injury and received medical treatment [since Rs LAST IW MONTH, YEAR/ in the last two years]? (),

1. Yes
2. No → Skip to procedure before DA023 DA023

DA022 Does your injury caused by the accident limit your daily activities?

1. Yes
2. No

**PROCEDURE**
If XRType = NEWIW, ask DA023 DA023
If XRType = REIW, ask DA023_W4 DA023_W4

DA023 Have you fallen down?

1. Yes
2. No → Skip to procedure before DA025 DA025

DA023_W4 Have you fallen down since [ZIWTime]? []

1. Yes
2. No → Skip to procedure before DA025 DA025

DA024 How many times have you fallen down seriously enough to need medical treatment?

? ______ times

**PROCEDURE**
If XRType = NEWIW, ask DA025 DA025
If XRType = REIW, ask DA025_W4 DA025_W4

DA025 Have you ever fractured your hip?

1. Yes
2. No

[F1 ,]

DA025_W4 Have you fractured your hip since we talked in [ZIWTime]? [ ]

1. Yes
2. No

[F1 ,]

**PROCEDURE**
IF R is male, skip to procedure before DA029 DA029

**DA026 BRANCHPOINT**:
If XRType = REIW reported in previous wave that has not started menopause, skip to DA027 Zmenopause ≠ 1 DA027
If XRType = REIW reported in previous wave that has started menopause, skip to DA032 Zmenopause = 1 DA032
If XRType = NEWIW, ask DA026 - DA028 DA026 - DA028

DA026 When did you begin the menarche?

1. ______ 1900...2018 (**DA026_1**) Year Or Age ______ 1...120 (**DA026_2**) Years 99 Never have menstrual periods

[IWER: Mark the year using four digits : 4]

DA027 Have you started menopause?

1. Yes
2. No → Skip to DA032 DA032
3. Dont Know → Skip to DA032 DA032

DA028 When did you begin the menopause?

______ (**DA028_1**) Year Or Age ______ (**DA028_2**) Years [IWER: Mark the year using four digits : 4]

PROCEDURE

If XRType = NEWIW and R is male, ask DA029 DA029

If XRType = REIW and R is male, ask DA029_W4 DA029_W4

DA029 Have you ever been diagnosed with a prostate illness, such as prostate hyperplasia (excluding prostatic cancer) ? （ ）

1. Yes → Skip to procedure before DA030 DA030
2. No

DA029_W4 Have you ever been diagnosed with a prostate illness, such as prostate hyperplasia（excluding prostatic cancer）since [ZIWTime]? （） （）

1. Yes → Skip to procedure before DA030 DA030
2. No

[F1]

DA029_W2_1 Do you know if you had a prostate illness, such as prostate hyperplasia (excluding prostatic cancer) ? （）

1. Yes
2. No → Skip to DA032 DA032
3. Dont know → Skip to DA032 DA032

PROCEDURE

If ZDA030 is not missing, skip DA030 ZDA030 DA030

DA030 When was the condition first diagnosed?

______ (**DA030_1**) Year Or Age ______ (**DA030_2**) Years

[IWER: Mark the year using four digits : 4]

DA031 Are you now taking medication or other treatment for your prostate illness?

1. Yes
2. No

DA032 Now I have some questions about your eyesight. Do you usually wear glasses or corrective lenses? （）

1. Yes
2. Legally blind → Skip to DA038_W4 DA038_W4
3. No

4. Sometimes

DA033 How good is your eyesight for seeing things at a distance, like recognizing a friend from across the street (with glasses or corrective lenses if you wear them)? Would you say your eyesight for seeing things at a distance is excellent, very good, good, fair, or poor? （） （）

1. Excellent
2. Very good
3. Good
4. Fair
5. Poor

997 Dont know

DA034 How good is your eyesight for seeing things up close, like reading ordinary newspaper print (with glasses or corrective lenses if you wear them)? Would you say your eyesight for seeing things up close is excellent, very good, good, fair, or poor? （ ）

1. Excellent
2. Very good
3. Good
4. Fair
5. Poor

997 Dont know

PROCEDURE

If XRType = NEWIW, ask DA035 DA035

If XRType = REIW and R reported in last IW that had cataract surgery on one eye (ZcataractNUM = 1), ask DA035_W2_1 （ZcataractNUM = 1） DA035_W2_1

If XRType = REIWandR did not report in last IW that had cataract surgery (Zcataract ≠ 1), ask DA035_W2_2 DA035_W2_2

DA035 Have you ever had cataract surgery?

1. Yes → Skip to DA036 DA036
2. No → Skip to procedure before DA037 DA037

DA035_W2_1 Have you had another cataract surgery since [ZIWTime] other than what you told us about then? （）

1. Yes → Skip to procedure before DA037 DA037
2. No → Skip to procedure before DA037 DA037

DA035_W2_2 Have you had cataract surgery since [ZIWTime]? （）

1. Yes
2. No → Skip to procedure before DA037 DA037

DA036 Have you had cataract surgery on both eyes or just one?

1. One eye only
2. Both eyes

PROCEDURE

If XRType = NEWIW or XRType = REIW and R did not report in last IW that had glaucoma (Zglaucoma ≠ 1), ask DA037 （Zglaucoma ≠ 1） DA037

If XRType = REIW and R reported in last IW that had glaucoma (Zglaucoma = 1), ask DA037_W2（Zglaucoma = 1） DA037_W2

DA037 Has a doctor/nurse/paramedical/doctor of traditional Chinese medicine doctor ever treated you for glaucoma?

1. Yes → Skip to DA038_W4 DA038_W4
2. No → Skip to DA038_W4 DA038_W4

DA037_W2 You told us you had glaucoma, has a doctor/nurse/paramedical/ doctor of traditional Chinese medicine doctor ever treated you for glaucoma relapses since [ZIWTime]? []

1. Yes
2. No
3. Never had glaucoma

DA038_W4 Now I have some questions about your hearing. Do you ever wear a hearing aid?

1. Yes
2. No

DA039 Is your hearing very good, good, fair, poor, or very poor (with a hearing aid if you normally use it and without if you normally dont)? Would you say your hearing is excellent, very good, good, fair, or poor? （ ）

1. Excellent
2. Very good
3. Good
4. Fair
5. Poor

997 Dont know

PROCEDURE
If XRType = NEWIW or XRType = REIW that did not lost all teeth in last IW (Ztooth ≠ 1), ask DA040（Ztooth≠ 1）DA040

DA040 Have you lost all of your teeth?
1. Yes
2. No

DA040_W4_1 Do you wear dentures?
1. Yes
2. No

DA040_W4_2 Have you experienced difficulty chewing solid foods (for example, apples)(IW: ask the ability to chew with dentures for denture wearers) （）（）
1. Yes
2. No
3. Not Applicable

DA041_W4 Are you often troubled with any body pains?
[IWER: The pain in all parts of Rs body]
1. None → Skip to DA045 DA045
2. A little
3. Somewhat
4. Quite a bit
5. Very

DA042 On what part of your body do you feel pain? Please list all parts of body you are currently feeling pain.
1. Head (Headache)
2. Shoulder
3. Arm
4. Wrist
5. Fingers
6. Chest
7. Stomach (Stomachache)
8. Back
9. Waist
10. Buttocks
11. Leg

12. Knees
13. Ankle
14. Toes
15. Neck
16. Other, please specify ______ (**DA042_1**)

DA042_W2_1 Are you taking measures to reduce the pain? (Check all that apply) ( )

1. Taking Chinese traditional medicine
2. Taking Western modern medicine
3. Acupuncture treatment
4. Professional massage therapy
5. Other, please specify ______ (**DA042_W2_1_1**)
6. None

DA045 Are there any other medical diseases or conditions that are important to your health now that we have not talked about?

1. Yes
2. No → Skip DA046 DA046

DA046 What illness is that? _______

DA048 How would you evaluate your health during childhood, up to and including age 15? Excellent, very good, good,fair, poor? 15 (15)

1. Excellent
2. Very Good
3. Good
4. Fair
5. Poor

997 Dont know

PART II Lifestyle and Health Behaviors

DA049 During the past month, how many hours of actual sleep did you get at night (average hours for one night)? (This maybe shorter than the number of hours you spend in bed.)
( ) _ 0...24 hours

DA050 During the past month, how long did you take a nap after lunch? ______ Minutes
[IWERIfR didnt take a nap, please record for 0

0]

[INTRO: We would like to knowhow much time you usually spend on physical activities every week]

CAPI

Ask DA051 for each type of physical activity, including: DA051

1Vigorous-intensity activity (Vigorous activities can cause shortness of breath. Examples of vigorous-intensity activities include carrying heavy stuff, digging, hoeing, aerobic workout, bicycling at a fast speed, riding a cargo bike/ motorcycle, etc.) （ ）

2Moderate activity (Moderate activities can make you breathe faster than usual. Examples of moderate activities include carrying light stuff, bicycling at a normal speed, mopping, Tai-Chi, and speed walking. （ ）

3Mild activities such as walking (walking from one place to another place at a workplace or home, and taking a walk for leisure, sports, exercise or entertainment) （ ）

If DA051[i] = 1, ask DA052 to DA051_1 for each type of physical activity. DA051[i] = 1DA052 DA051_1

DA051 Please recall the [preload the type of physical activity] that you have taken part in for at least 10 minutes every time in a week. Do you usually take this type of activity for at least 10 minutes every week? []

1. Yes
2. No → Start the next type of physical activity or skip to DA056 DA056

DA052 How many days a week do you take part in [preload the type of physical activity] for at least 10 minutes? [] ______ 1...7 days

DA053 During the days you take part in [preload the type of physical activity], how long do you do [preload the type of physical activity] every day? [] []

1. < 2 hours
2. ≥ 2 hours → Skip to DA055 DA055

DA054 During the days you take part in [preload the type of physical activity], how long do you do [preload the type of physical activity] every day? [] []

1. < 30 minutes → Skip to DA051_1 DA051_1
2. ≥ 30 minutes → Skip to DA051_1 DA051_1

DA055 During the days you take part in [preload the type of physical activity], how long do you do [preload the type of physical activity] every day? [] []

1. < 4 hours
2. ≥ 4 hours

DA051_1 Whats the purpose for doing these physical activities, for entertainment, job demand or exercise in doing these physical activities?

1. Job demands
2. Entertainments
3. Exercise
4. Other

DA056 Have you done any of these activities in the last month? (Check all that apply) ( )

1. Interacted with friends
2. Played Ma-jong,played chess, played cards, or went to community club
3. Provided help to family, friends, or neighbors who do not live with you
4. Went to a sport, social, or other kind of club 5. Took part in a community-related organization 6. Done voluntary or charity work
7. Cared for a sick or disabled adult who does not live with you
8. Attended an educational or training course
9. Stock investment ( )
10. Used the Internet
11. Other
12. None of these

**PROCEDURE**
If DA056 = 10, ask DA056_W3 DA056 = 10 DA056_W3

DA056_W3 Which types of devices do you use to access the Internet? (check all that apply) ? ( )

1. Desktop computer
2. Laptop computer
3. Tablet computer (such as IPAD) (IPAD)

4. Cellphone
5. Other devices, please specify ______ （**DA056_W3_1**）

**PROCEDURE**
Ask the frequency of each social activity selected in DA056 in the last month in DA057 DA057 DA056

DA057 How often in the last month [did/have][you] do [preload DA056]? Almost daily, almost every week, or not regularly? [DA056]

1. Almost daily
2. Almost every week
3. Not regularly

**PROCEDURE**
If DA056 = 10, ask DA056_W4_1 - DA056_W4_4 DA056 = 10 DA056_W4_1 - DA056_W4_4

DA056_W4_1 What do you usually do on the Internet? (check all that apply) ()

1. Chat
2. Watch news
3. Watch videos
4. Play games
5. Financial management
6. Others, please specify ______ （**DA056_W4_1_1**）

DA056_W4_2 Do you use mobile payments, such as Alipay and WeChat pay?

1. Yes
2. No

DA056_W4_3 Do you use WeChat? ?

1. Yes
2. No → Skip to branchpoint before DA059 DA059

DA056_W4_4 Do you post WeChat moments? ?

1. Yes
2. No

[INTRO: Next,I would like to ask whether you have had the habit of smoking cigarettes/smoking a pipe/chewing tobacco, now or in the past. By smoking we mean smoking more than 100 cigarettes in your life （ 100 ）]

DA059 BRANCHPOINT:

If XRType = REIW and R reported ever smoked (Zsmoke = 1), skip to DA061_W4 DA061_W4

If XRType = REIW and R did not report ever smoked (Zsmoke ≠ 1), or XRType = NEWIW, ask DA059 DA059

DA059 Have you ever chewed tobacco, smoked a pipe, smoked self-rolled cigarettes, or smoked cigarettes/cigars? ( )

1. Yes
2. No → Skip to DA067 DA067

DA061 Do you still have the habit or have you totally quit?

1. Still have → Skip to DA060 DA060
2. Quit → Skip to DA060 DA060

DA061_W4 Our records from your last interview show that you have ever smoked, Do you still have the habit or have you totally quit?

1. Still have
2. Quit
3. Never smoked → Skip to DA067 DA067

DA060 Which products did/do you normally use?

1. Smoking a pipe ( )
2. Smoking self-rolled cigarettes
3. Filtered cigarette
4. Unfiltered cigarette
5. Cigar
6. Water cigarettes

PROCEDURE

If R stopped smoking (DA061 = 2 or DA061_W4 = 2), ask DA062 DA061 = 2 DA061_W4 = 2 DA062

DA062 At what age did you totally quit smoking? __ 1...120 (**DA062_1**) Age or ______ 1900...2018 (**DA062_2**) Year

[IWER: Mark the year using four digits : 4]

PROCEDURE

If DA060 = 3, 4, ask DA063 DA060 = 3, 4 DA063

DA063 In one day about how many cigarettes do/did you consume [preload: now/before totally quitting]? [/] _____ Cigarettes

PROCEDURE

If XRType = REIW that has been asked when started smoking, skip DA065 ZDA065 ≠ . DA065

DA065 At what age did you start to smoke on a regular basis? _____ 1...120 (**DA065_1**) Age
Or ______ 1900...2018 (**DA065_2**) Year
[IWER: Mark the year using four digits : 4]

DA067 Did you drink any alcoholic beverages, such as beer, wine, or liquor in the past year? How often?

1. Drink more than once a month
2. Drink but less than once a month → Skip to procedure before DA069 DA069
3. None of these → Skip to procedure before DA069 DA069

DA068 What type of alcoholic beverages did you drink? Liquor, wine, or beer? (check all that apply) （）

1. Liquor, including white liquor, whisky, and others → Skip to procedure before DA071 DA071
2. Beer → Skip to procedure before DA071 DA071
3. Wine or rice wine → Skip to procedure before DA071 DA071

PROCEDURE

If XRType = REIW that has never been asked whether drinking alcoholic beverages in the past, ask DA069 ZDA069 = . DA069 If XRType = REIW that only never had a drink and used to drink a little (ZDA069 = 1 or ZDA069 = 2),skip to DA081. ZDA069 = 1 ZDA069 = 2 DA081

If XRType = REIWthat used to drink (ZDA069 = 3), skip to the procedure before DA070 ZDA069 = 3 DA070

DA069 Did you ever drink alcoholic beverages in the past? How often?

1. I never had a drink. → Skip to DA081 DA081
2. I used to drink less than once a month. → Skip to DA081 DA081
3. I used to drink more than once a month.

PROCEDURE

If XRType = REIW that has been asked when quitting alcoholic beverages in the past, skip DA070 ZDA069 = 3 ZDA070 ≠ . DA070

DA070 When did you quit or reduce drinking?

______ 1900...2018 (**DA070_1**) Year or Age : ______1...120 (**DA070_2**) Years

[IWER: Record year in 4 digits 4]

**PROCEDURE**
If XRType = REIW that has been asked when started drinking, skip DA0 7 1 ZDA071 ≠ . DA071

DA071 When did you start drinking?

______ 1900...2018 (**DA071_1**) Year or Age ______ 1...120 (**DA071_2**) Years

[IWER: Record year in 4 digits. 4]

[INTRO: Now, I am going to ask you how often and how much you drank during the past year. Please tell me how often you drank per month, and how much you drank at a time on average. I will repeat the questions for different types of alcoholic beverages]

**PROCEDURE**
If DA068 = 1, ask DA072 DA068 = 1 DA072

DA072 How often did you drink liquor, including white liquor, whisky, and others per month in the last year?

1. Once a month
2. 2-3 times a month 2-3
3. Once a week
4. 2-3 times a week 2-3
5. 4-6 times a week 4-6
6. Once a day
7. Twice a day
8. More than twice a day

DA073 The last time you drank liquor last year, how many liang of liquor did you drink? (1 liang = 50 ml) (1 = 50) _____ Liang

**PROCEDURE**
If DA068 = 2, ask DA074 DA068 = 2 DA074

DA074 How many times per month did you drink beer in the last year?

1. Once a month
2. 2-3 times a month 2-3
3. Once a week
4. 2-3 times a week 2-3

5. 4-6 times a week 4-6
6. Once a day
7. Twice a day
8. More than twice a day

DA075 The last time you drank beer last year, how many bottles of beer did you drink? (1 bottle = 2.5 mugs, 1 mug = 220 ml) ?（1 =2.5 1 = 220）
______ (**DA075_1**) 0...120 Bottles or ______ (**DA075_2**) 0...300 Mugs

PROCEDURE
If DA068 = 3, ask DA076 DA068 = 3 DA076

DA0 7 6 How often did you drink wine or rice wine per month in the last year?
1. Once a month
2. 2-3 times a month 2-3
3. Once a week
4. 2-3 times a week 2-3
5. 4-6 times a week 4-6
6. Once a day
7. Twice a day
8. More than twice a day

DA077 The last time you drank it last year, how many liang of wine did you drink? (1 liang = 50 ml) （1 = 50 ） _____ 0.00...100.00 Liang

**INTERVIEWER CHECK AGE OF RESPONDENT?**	**1.** < 65 **YEAR → COLUMN A A** **2.** 65 – 69 **YEAR → COLUMN B B** **3.** 70 – 74 **YEAR → COLUMN C C** **4.** 75 – 79 **YEAR → COLUMN D D** **5.** 80 – 84 **YEAR → COLUMN E E**				**6.** 85 – 89 **YEAR → COLUMN F F** **7.** 90 – 94 **YEAR → COLUMN G G** **8.** 95 – 99 **YEAR → COLUMN H H** **9.** ≥ 100 **YEAR → COLUMN I I**				
	A	B	C	D	E	F	G	H	I
AGE	75 years	80 years	85 years	90 years	95 years	100 years	105 years	110 years	115 years
**DA081** Suppose there are 5 steps, where the lowest step represents the smallest chance and the highest step represents the highest chance, on what step do you think is your chance in reaching the age of [...]? 1 Almost impossible 2 Not very likely 3 Maybe 4 Very likely 5 Almost certain 997 Dont know	1<br>2<br>3<br>4<br>5	1<br>2<br>3<br>4<br>5	1<br>2<br>3<br>4<br>5	1<br>2<br>3<br>4<br>5	1<br>2<br>3<br>4<br>5	1<br>2<br>3<br>4<br>5	1<br>2<br>3<br>4<br>5	1<br>2<br>3<br>4<br>5	1<br>2<br>3<br>4<br>5

DB Functional Limitations and Helpers

PROCEDURE

IfR is younger than 50 (year of birth is after 1968) and if DA002 = 1, 2 andDA005[i] = 2 and Zdisability[i] = 2 and DA007[i] = 2 and DA008[i] = 2 and Zdisease[i] = 2 and DA041_W4 = 1, skip DB001 - DB015 50 （ 1968 ） DB001 - DB015

[We need to understand difficulties people may have with various activities because of a health or physical problem. Please tell me whether you have difficulty performing any of the following tasks on a regular basis. Exclude any difficulties that you expect to last less than three months （）]

DB001 Do you have any difficulty with running or jogging about 1 Km? 1

1. No, I dont have any difficulty → Skip to DB004 DB004
2. I have difficulty but can still do it
3. Yes, I have difficulty and need help
4. I can not do it

DB002 Do you have difficulty with walking 1 km? 1 1. No, I dont have any difficulty → Skip DB003 DB003

2. I have difficulty but can still do it
3. Yes, I have difficulty and need help
4. I can not do it

DB003 Do you have difficulty with walking 100 metres? 100 1. No, I dont have any difficulty

2. I have difficulty but can still do it
3. Yes, I have difficulty and need help
4. I can not do it

DB004 Do you have difficulty with getting up from a chair after sitting for a long period?

1. No, I dont have any difficulty
2. I have difficulty but can still do it
3. Yes, I have difficulty and need help
4. I can not do it

DB005 Do you have difficulty with climbing several flights of stairs without resting?

1. No, I dont have any difficulty
2. I have difficulty but can still do it
3. Yes, I have difficulty and need help
4. I can not do it

DB006 Do you have difficulty with stooping, kneeling, or crouching?

1. No, I dont have any difficulty
2. I have difficulty but can still do it
3. Yes, I have difficulty and need help
4. I can not do it

DB007 Do you have difficulty with reaching or extending your arms above shoulder level? (he/she is regarded as not having difficulty only if he/she can extend both of his/her arms, otherwise he/she is regarded as having difficulty.) （ ）

1. No, I dont have any difficulty
2. I have difficulty but can still do it
3. Yes, I have difficulty and need help
4. I can not do it

DB008 Do you have difficulty with lifting or carrying weights over 10 jin, like a heavy bag of groceries? 10 （）

1. No, I dont have any difficulty
2. I have difficulty but can still do it
3. Yes, I have difficulty and need help
4. I can not do it

DB009 Do you have difficulty with picking up a small coin from a table?

1. No, I dont have any difficulty
2. I have difficulty but can still do it
3. Yes, I have difficulty and need help
4. I can not do it

**PROCEDURE**
If (DB001 = 1 & DB004 = 1 ... DB009 = 1), then skip to DB016 DB001 ~ DB009 DB016

[INTRO: Here are a few more everyday activities. Please tell me if you have any difficulties with these because of a physical, mental, emotional or memory problem. Again, exclude any that you expect to last less than three months]

DB010 Because of health and memory problems, do you have any difficulty with dressing? Dressing includes taking clothes out from a closet, putting them on, buttoning up, and fastening a belt.

1. No, I dont have any difficulty → Skip to DB011 DB011
2. I have difficulty but can still do it
3. Yes, I have difficulty and need help
4. I can not do it

DB010_W2 Does anyone ever help you dress? 1. Yes

2. No

DB011 Because of health and memory problems, do you have any difficulty with bathing or showering?

1. No, I dont have any difficulty → Skip to DB012 DB012
2. I have difficulty but can still do it
3. Yes, I have difficulty and need help
4. I can not do it

DB011_W2 Does anyone ever help you bathe?

1. Yes
2. No

DB012 Because of health and memory problems, do you have any difficulty with eating, such as cutting up your food? (Definition: By eating, we mean eating food by oneself when it is ready) ( )

1. No, I dont have any difficulty → Skip to DB013 DB013
2. I have difficulty but can still do it
3. Yes, I have difficulty and need help
4. I can not do it

DB012_W2 Does anyone ever help you eat?

1. Yes
2. No

DB013 Do you have any difficulty with getting into or out of bed? 1. No, I dont have any difficulty → Skip to DB014 DB014

2. I have difficulty but can still do it
3. Yes, I have difficulty and need help
4. I can not do it

DB013_W2 Does anyone ever help you get in or out of bed? 1. Yes
2. No

DB014 Because of health and memory problems, do you have any difficulties with using the toilet, including getting up and down?

1. No, I dont have any difficulty → Skip to DB015 DB015
2. I have difficulty but can still do it
3. Yes, I have difficulty and need help
4. I can not do it

DB014_W2 Does anyone ever help you use the toilet?

1. Yes
2. No

DB015 Because of health and memory problems, do you have any difficulties with controlling urination and defecation? If you use a catheter (conduit) or a pouch by yourself, then you are not considered to have difficulties. （）

1. No, I dont have any difficulty
2. I have difficulty but can still do it
3. Yes, I have difficulty and need help
4. I can not do it

DB016 Because of health and memory problems, do you have any difficulties with doing household chores? (Definition: By doing household chores, we mean house cleaning, doing dishes, making the bed, and arranging the house) （）

[IWER: If R cannot mop the floor, but can scrub, or R cannot fold heavy bedding, but is able to do light ones, then mark (3) （3）]

1. No, I dont have any difficulty → skip to DB017 DB017 2. I have difficulty but can still do it
3. Yes, I have difficulty and need help
4. I can not do it

DB016_W2 Does anyone help you do household chores? 1. Yes
2. No

DB017 Because of health and memory problems, do you have any difficulties with preparing hot meals? (Definition: By preparing hot meals, we mean preparing ingredients, cooking,

and serving food) （ ）

[IWER: If another person prepares ingredients or if R can cook rice, but is not able to prepare side dishes, then mark (3) （3）]

1. No, I dont have any difficulty → skip to DB018 DB018
2. I have difficulty but can still do it
3. Yes, I have difficulty and need help
4. I can not do it

DB017_W2 Does anyone help you prepare hot meals? 1. Yes
2. No

DB018 Because of health and memory problems, do you have any difficulties with shopping for groceries? By shopping, we mean deciding what to buy and paying for it.

1. No, I dont have any difficulty → skip to DB035 DB035
2. I have difficulty but can still do it
3. Yes, I have difficulty and need help
4. I can not do it

DB018_W2 Does anyone help you shop for groceries?

1. Yes
2. No

DB035 Because of health and memory problems, do you have any difficulties with making phone calls?

1. No, I dont have any difficulty → skip to DB020 DB020
2. I have difficulty but can still do it
3. Yes, I have difficulty and need help
4. I can not do it
5. Not relevant to me (no phone) → skip to DB020 DB020

DB035_W2 Does anyone help you make telephone calls? 1. Yes
2. No

DB020 Because of health and memory problems, do you have any difficulties with taking medications? By taking medications, we mean taking the right portion of medication right on time.

1. No, I dont have any difficulty → skip to DB019 DB019 2. I have difficulty but can still do it
3. Yes, I have difficulty and need help
4. I can not do it

DB020_W2 Does anyone help you take medications?
1. Yes
2. No

DB019 Because of health and memory problems, do you have any difficulties with managing your money, such as paying your bills, keeping track of expenses, or managing assets?
1. No, I dont have any difficulty → skip DB019_W2 DB019_W2 2. I have difficulty but can still do it
3. Yes, I have difficulty and need help
4. I can not do it

DB019_W2 Does anyone help you manage your money? 1. Yes
2. No

PROCEDURE
If DB010_W2 = 1 or DB011_W2 = 1 or DB012_W2 = 1 or DB013_W2 = 1 or DB014_W2 = 1 or DB016_W2 = 1 or DB017_W2 = 1 or DB018_W2 = 1 or DB035_W2 = 1 or DB020_W2 = 1 or DB019_W2 = 1, ask DB022_W3_1; otherwise, skip to DB029

DB022_W3_1 Who most often helps you with [make sure we ask this only once for all these activities; do not ask for each problem separately] (dressing, bathing, eating, getting out of bed, using the toilet, controlling urination and defecation, doing chores, preparing hot meals, shopping, managing money, making phone calls, taking medications) (check all that apply)? ( ) ()
1. Spouse
2. Father, Mother, Father-in-law, Mother-in-law
3. Children, Childrens spouses, Grandson, Granddaughter //
4. Sibling, Brother-in-law, Sister-in-law, Sibling of spouse, Children of sibling, Brother-in-law of spouse, Sister-in-law of spouse, Children of brother-in-law, Children of sister-in-law
5. Other relative
6. Paid helper (such as nanny) () ______ (**DB022_W3_1_1**)
7. Volunteer
8. Employee(s) of facility

9. Community
10. Other, please specify ______ (**DB022_W4_1**)

[IWER: employee(s) of facility appears on list only for an R currently living in a nursing home or who was living in a nursing home or hospice when he/she died]

**PROCEDURE**
If DB022_W3_1 = 2, ask DB023_W3_1 DB022_W3_1 = 2, ask DB023_W3_1

DB023_W3_1 Among father, mother, father-in-law, mother-in-law, who help you? (check all that apply) ( )

1. Father
2. Mother
3. Father-in-law /
4. Mother-in-law /

**PROCEDURE**
If DB022_W3_1 = 3, ask DB023_W3_2

DB023_W3_2 For the children, children-in-law, grandson, granddaughter who helped you, which children s family are they from? (check all that apply) / / ( )

1-25 [Preload childrens name] []

26 None of the above

**PROCEDURE**
For each helper from every childs family, repeat the question DB023_W3_3 DB023_W3_3

DB023_W3_3 For the family members of [Preload childs name], who help you in person? (check all that apply) [] ( )

1 [Preload childs name] himself/herself []

2 [Preload childs name] his/her spouse []

3 [Preload childs name] his/her children. How many helped you in person? [ ] ( ) [] ______ (**DB023_W3_3_1**)

**PROCEDURE**
If DB022_W3_1 = 4, ask DB023_W3_4 DB022_W3_1 = 4, DB023_W3_4

DB023_W3_4 For the siblings, spouse and children of siblings, siblings of your spouse, spouse and children of siblings of your spouse who helped you, which siblings family are they from? ( )

1-15 [Preload siblings name] []
16-30 [Preload spouses siblings name] []
99 None of the above

PROCEDURE
For each helper from every sibling, siblings of spouse, repeat the question DB023_W3_5 DB023_W3_5

DB023_W3_5 For the family members of [Preload name of siblings, siblings of spouse], who help you in person? (check all that apply) [/] ()

1 [Preload siblings (of spouse) name] himself/herself [()]
2 [Preload siblings (of spouse) name] his/her spouse [()]
3 For the children from [Preload siblings (of spouse) name], how many children from [Preload siblings (of spouse) name] help you in person? [()] [()] ______ (**DB023_W3_5_1**)

PROCEDURE
If DB022_W3_1 = 5, ask DB023_W3_6 DB022_W3_1 = 5, DB023_W3_6

DB023_W3_6 The number of the relatives who help you in person ______ Whats their relationship with you ______ (**DB023_W3_6_1**)

PROCEDURE
If DB022_W3_1 = 10, ask DB023_W3_7 DB022_W3_1 = 10, DB023_W3_7

DB023_W3_7 The number of the others who help you in person __ Whats that persons relationship with you ______ (**DB023_W3_7_1**)

PROCEDURE
If the number of helpers larger than 7, ask DB023_W3_9 7 （ 7DB022_W3_1 = 1DB022_W3_1 = 5DB022_W3_1 = 6 DB022_W3_1 = 7DB022_W3_1 = 8DB022_W3_1 = 9DB022_W3_1 = 10DB023_W3_1 = 1 DB023_W3_1 = 2DB023_W3_1 = 3DB023_W3_1 = 4DB023_W3_3[i] = 1DB023_W3_3[i] = 2DB023_W3_3[i] = 3DB023_W3_5[i] = 1DB023_W3_5[i] = 2DB023_W3_5[i] = 3） DB023_W3_9

DB023_W3_9 From all the helpers list below, please select the most important 7 helpers for you 7

Name of all helpers from DB022_W3_1 - DB023_W3_5 DB022_W3_1 - DB023_W3_5

**PROCEDURE**
For each helper chosen in DB022_W3_1 -DB023_W3_5, ask DB023 - DB025 DB022_W3_1 - DB023_W3_5 DB023 - DB025

DB023 During the last month, on about how many days did [helper] help you? [] ______
1...31 Days

DB024 On the days [helper] helps you, about how many hours per day is that? [] / ______
1...24 Hours
[IWER: less than an hour, mark 1 1]

DB025 Is he/she living in your home? / 1. Yes
2. No

DB029 Do you use the following auxiliary? (Check all that apply) ( )
1. Walking stick
2. Travel device
3. Manual wheelchair
4. Electric Wheelchair
5. Catheter, urine collection bag
6. Toilet Series
7. None of the above

DB030 Suppose that in the future, you needed help with basic daily activities like eating or dressing. Do you have relatives or friends (besides your spouse/partner) who would be willing and able to help you over a long period of time?
1. Yes
2. No → Skip to DB036_W4 DB036_W4

DB031 What is the relationship to you of that person or those persons? (Check all that apply) / ()
1. Spouse
2. Father, Mother, Father-in-law, Mother-in-law
3. Children, Childrens spouses, Grandson, Granddaughter //
4. Sibling, Brother-in-law, Sister-in-law, Sibling of spouse, Children of sibling, Brother-in-law of spouse, Sister-in-law of spouse, Children of brother-in-law, Children of sister-in-law
5. Other relative
6. Paid helper (such as nanny) ()

7. Volunteer
8. Employee(s) of facility
9. Community
10. Other, please specify ______ (**DB031_1**)

**PROCEDURE**
If DB031 = 2, ask DB031_W3_1

DB031_W3_1 Father, mother, father-in-law, mother-in-law, who will help you in future? （ ）

1. Father
2. Mother
3. Father-in-law /
4. Mother-in-law /

**PROCEDURE**
If DB031 = 3, ask DB031_W3_2 DB031 = 3 DB031_W3_2

DB031_W3_2 For the children, children-in-law, grandchildren who will help you in future, which childrens family are they from? // （）

1-25 [Preload childrens name] []

26 None of the above

**PROCEDURE**
If DB031 = 4, ask DB031_W3_4 DB031 = 4, DB031_W3_4

DB031_W3_4 For the siblings, spouse and children of siblings, spouses siblings, spouse and children of spouses siblings who will help you in future, which childrens family are they from? （）

1-15 [Preload siblings name] []

16-30 [Preload siblings name of spouse] []

99 None of the above

**PROCEDURE**
If DB031 = 5, ask DB031_W3_6 DB031 = 5, DB031_W3_6

DB031_W3_6 The number of other relatives who will help you in person in future ______

**PROCEDURE**
If DB031 = 10, ask DB031_W3_7 DB031 = 10, DB031_W3_7

DB031_W3_7 The number of others who will help you in person in future ______

[INTROWe would like to know whether your health problems limit your ability to work]

DB036_W4 Do you think the following description fits you: I cannot work because of my disability or health problems

1. I am unable to work → Skip to DB032 DB032 2. I cannot work long hours
3. I dont have any problem

DB037_W4 Do you think the following description fits you: I cannot do housework because of my disability or health problems.

1. I am unable to do housework
2. I cannot do housework for an extended period of time 3. I dont have any problem

DB032 How often did the respondent receive assistance in answering this section

[IWER: If it is answered by a proxy, please record the respondents reaction]

1. Never
2. A few times
3. Most or all of the time
4. The section was completed by a proxy respondent（the respondent is absent） → Skip to DB033 DB033

DB033 What is your relationship to R?

[IWER: What is the proxys relationship to R? If unknown, please ask the proxy]

1. Spouse
2. Mother
3. Father
4. Mother-in-law /
5. Father-in-law
6. Sibling
7. Brother-in-law, sister-in-law /
8. Child
9. Spouse of child

10. Grandchild
11. Other relative
12. Helper or other non-relative

DB034 What is the main reason for proxy (the respondent is absent)

1. The respondent has serious physical handicaps 2. The respondent has serious mental handicaps

3. The respondent has declined this interview.
4. Other ______ (**DB034_1**)

DC Cognition and Depression

SECTION INTRO Introducing Respondent Interview

[INTRO: Next I will be asking you to complete a series of memory, thinking, and other tasks. When we are done, I will ask one of your informant to also complete a short questionnaire. If we should come to any question that you dont want to answer, just let me know and I will go on to the next question]

PROCEDURE

Respondent aged 60 years and older should done all tests in DC 60 （Age>=60） DC

Respondent aged less than 60 years should only done the tests listed below 60 （Age <60）

DC001_W4DC002_W4DC003_W4DC005_W4DC006_W4DC004DC014_W4 DC014_W4_1DC014_W4_2DC014_W4_3DC014_W4_4DC014_W4_5DC024 DC024_W4 in SECTION MMSE

All items in SECTION WR

All items in SECTION CESD

All items in SECTION SAT

All items in SECTION DR

All items in SECTION NS

SECTION MMSE Mini Mental State Exam

[INTRO: First I would like to ask you some questions to check your memory and concentration. Some of them may be easy and some may be hard]

DC001_W4 What is the year?

1 = Correct ; 5 = Error ; 97 = Not assessed

DC002_W4 What is the season of the year?

1 = Correct ; 5 = Error ; 97 = Not assessed

DC003_W4 What is the date?

[IWER: Lunar date is correct,not allowed to check the calendar]

1 = Correct ; 5 = Error ; 97 = Not assessed

DC005_W4 What is the day of the week?

1 = Correct ; 5 = Error ; 97 = Not assessed

DC006_W4 What is the month?

[IWER: Lunar month is correct,not allowed to check the calendar]

1 = Correct ; 5 = Error ; 97 = Not assessed

DC007_W4 What state are we in?

1 = Correct ; 5 = Error ; 97 = Not assessed

DC008_W4 What county are we in? （） 1 = Correct ; 5 = Error ; 97 = Not assessed

DC009_W4 What city or town are we in? （/）

1 = Correct ; 5 = Error ; 97 = Not assessed

DC010_W4 What floor of the building are we on?

1 = Correct ; 5 = Error ; 97 = Not assessed

DC012_W4 What is this address? (If institutionalized, what is the name of the institution?)

[IWER: Ask the name of the hospital if interviewed in hospital, ask the name of the building or street if interviewed in community]

1 = Correct ; 5 = Error ; 97 = Not assessed

DC004 What is the month? 1 = Excellent ; 2 = Very Good ; 3 = Good ; 4 =Fair ; 5 = Poor

[INTRO: I am going to name three objects. After I have said them, I want you to repeat them. Remember what they are because I am going to ask you to name them again in a few minutes. Ball, flag, tree. Please repeat the names for me]

DC013_W4_1 Interviewer please record words which are correctly recalled

1. Ball
2. Flag
3. Tree
4. None

97 Not assessed

PROCEDURE

If all 3 are recalled correctly (DC013_W4_1s1 = 1 and DC013_W4_1s2 = 1 and DC013_W4_1s3 = 1),skip to DC014_W4, otherwise, go on to DC013_W4_2 （DC013_W4_1s1 = 1 DC013_W4_1s2 = 1 DC013_W4_1s3 = 1） DC014_W4 DC013_W4_2

[INTRO: I am going to read the three objects again. After I have said them, I want you to repeat them. Ball, Flag, Tree. Please repeat the names for me]

DC013_W4_2 Interviewer please record words which are correctly recalled

1. Ball
2. Flag
3. Tree
4. None
97. Not assessed

PROCEDURE

If all 3 are recalled correctly (DC013_W4_2s1 = 1 and DC013_W4_2s2 = 1 and DC013_W4_2s3 = 1),skip to DC014_W4, otherwise, go on to DC013_W4_3 （DC013_W4_2s1 = 1 DC013_W4_2s2 = 1 DC013_W4_2s3 = 1） DC014_W4 DC013_W4_3

[INTRO: I am going to read the three objects again. After I have said them, I want you to repeat them. Ball, Flag, Tree. Please repeat the names for me]

DC013_W4_3 Interviewer please record words which are correctly recalled

1. Ball
2. Flag
3. Tree
4. None
97. Not assessed

PROCEDURE

If all 3 are recalled correctly (DC013_W4_3s1 = 1 and DC013_W4_3s2 = 1 and DC013_W4_3s3 = 1),skip to DC014_W4, otherwise, go on to DC013_W4_4 （DC013_W4_3s1 = 1 DC013_W4_3s2 = 1 DC013_W4_3s3 = 1） DC014_W4 DC013_W4_4

[INTRO: I am going to read the three objects again. After I have said them, I want you to repeat them. Ball, Flag, Tree. Please repeat the names for me]

DC013_W4_4 Interviewer please record words which are correctly recalled

1. Ball
2. Flag
3. Tree
4. None
97. Not assessed

PROCEDURE

If all 3 are recalled correctly (DC013_W4_4s1 = 1 and DC013_W4_4s2 = 1 and DC013_W4_4s3 = 1),skip to DC014_W4, otherwise, go on to DC013_W4_5 （DC013_W4_4s1 = 1 DC013_W4_4s2 = 1 DC013_W4_4s3 = 1） DC014_W4 DC013_W4_5

[INTRO: I am going to read the three objects again. After I have said them, I want you to repeat them. Ball, Flag, Tree. Please repeat the names for me]

DC013_W4_5 Interviewer please record words which are correctly recalled

1. Ball
2. Flag
3. Tree
4. None
97. Not assessed

[INTRO: please calculate 100 minus 7, and keep minus 7 continuously, tell me each answer you get from minus 7, until I say stop 100 7 7 7]

[IWER: After read the introduction, do not give any others instructions during the test, and do not remind the subject what should be done. No extra instructions except saying continue]

DC014_W4 100 minus 7, and keep minus 7 continuously for five times 100 7, 5

DC014_W4_1 Record answer from respondent ______ (**DC014_W4_1_1**) 97. DK → Skip to DC015_W4 DC015_W4

98. RF → Skip to DC015_W4 DC015_W4

DC014_W4_2 Record answer from respondent ______ (**DC014_W4_2_1**) 97. DK → Skip to DC015_W4 DC015_W4

98. RF → Skip to DC015_W4 DC015_W4

DC014_W4_3 Record answer from respondent ______ (**DC014_W4_3_1**) 97. DK → Skip to DC015_W4 DC015_W4
98. RF → Skip to DC015_W4 DC015_W4

DC014_W4_4 Record answer from respondent ______ (**DC014_W4_4_1**) 97. DK → Skip to DC015_W4 DC015_W4
98. RF → Skip to DC015_W4 DC015_W4

DC014_W4_5 Record answer from respondent ______ (**DC014_W4_5_1**) 97. DK
98. RF

DC024 [IWER: Please Indicate whether the respondent used paper and pencil or any other aid during Used paper, pen or other aid when completing the number subtraction]
1. Used aid
2. Did not use aid

PROCEDURE
If (DC013_W4_1s97 = 1, and DC013_W4_2s97 = 1, and DC013_W4_3s97 = 1, and DC013_W4_4s97 = 1, and DC013_W4_5s97 = 1), or DC013_W4_5s4 = 1, then skip to DC016_W4 (DC013_W4_1s97 = 1 DC013_W4_2s97 = 1 DC013_W4_3s97 = 1 DC013_W4_4s97 = 1 DC013_W4_5s97 = 1) DC013_W4_5s4 = 1 DC016_W4

DC015_W4 What were the three objects I asked you to remember? , ?
1. Ball
2. Flag
3. Tree
4. None
97. Not assessed

[PROGRAMMER: Display a picture of a watch on this page]

DC016_W4 [Interviwer, point to the watch picture on screen to respondent and ask] What is this called?
1 = Correct 5 = Error 97 = Not assessed

[PROGRAMMER: Display a picture of a pencil on this page]

DC017_W4 [Interviwer, point to the pencil picture on screen to respondent and ask] What is this called?
1 = Correct 5 = Error 97 = Not assessed

DC018_W4 I would like you to repeat a phrase after me. The phrase is: No ifs ands or buts

[IWER: Allow only one attempt to repeat the phrase

IWER: You can repeat the phrase up to five times if the respondent is struggling to hear the phrase

IWER: You cannot repeat it if respondent already attempted the phrase]

1 = Correct 5 = Error 97 = Not assessed

[PROGRAMMER: The following sentence will be displayed on a single page, occupy the whole screen]

DC019_W4 Interviwer show the page to respondent, which says Close your eyes, ask respondent to Read the words on this page, then do what it says.

1. Participant closed eyes; correct
2. Correct, Examiner read the phrase aloud
5. Error/Omission (e.g. participant did not close eyes) / （ ）
97. Not assessed

[INTRO: I am going to give you a piece of paper. When I do, take the paper in your right hand, fold the paper in half with both hands, and put the paper down on your left lap]

[IWER: You can read this instruction only once. Do not repeat instructions unless respindent didnt hear the instruction and do not coach]

DC020_W4 Hand

1. Respondent takes the paper with her/his right hand, correct
5. Respondent does not take the paper with the right hand score, error
97. Not assessed

DC021_W4 Fold

1. Respondent folds the paper with both hands, correct
5. Respondent does not fold the paper with both hands, error
97. Not assessed

DC022_W4 Leg

1. Respondent places the paper in her/his left lab, correct
5. The participate does not place the paper in her/ his left lap, error
97. Not assessed

DC023_W4 Write any complete sentence on that piece of paper for me

[IWER: Make sure to take photo of the paper, upload the photo onto system, check and score correctness of sentence （ ）]

1 = Correct ; 5 = Error ; 97 = Not assessed

[PROGRAMMER: Here add camera function for interviewers to take photo]

DC024_W4 Here is a drawing. Please copy the drawing on this paper

[IWER: Score correct if A) There are two five-sided figures which intersect to form a four-sided figure and B) All angles in the five sided figure much be preserved A) B)]

1 = Correct ; 5 = Error ; 97 = Not assessed

[PROGRAMMER: Here add camera function for interviewers to take photo]

SECTION HT HRS Telephone Interview for Cognitive Status (TICS)

[INTRO: Now Im going to ask you for the names of some people and things]

DC025_W4 What do people usually use to cut paper? 1. Scissors or shears only /

5. Not correct
8. DK
97. RF

DC026_W4 What do you call the kind of prickly plant that grows in the desert?

1. Cactus or name of kind of cactus /
5. Not correct

8. DK
97. RF
99. Other please specify ______ （**DC026_W4_1**）

DC027_W4 Who is the President of the United States right now? 1. Last name (XI) correct /
5. Not correct
8. DK
97. RF

SECTION WR Word Recall

WR101_INTRO I am going to show you ten printed words. Read each word out loud as I show it to you. Later I will ask you to recall all ten words. Is this clear?

[IWER: Probe as needed for understanding of task]

1. Yes → skip DC028_W4_1 DC028_W4_1
2. No

PROCEDURE
If respondent aged 60 years and above who refused this task, skip to RF101_Intro 60 RF101_Intro<br>If respondent aged less than 60 years who refused this task, skip to SECTION CESD 60 SECTION CESD

DC028_W4_1 Please record reasons for refusal? 1. Refused or unwilling to to
2. Cannot speak in whole life
3. Cannot speak when getting old
4. Deaf or poor hearing
5. Other please specify ______ (**DC028_W4_1_1**）

PROCEDURE
After DC028_W4_1, skip to RF101_Intro DC028_W4_1 RF101_Intro

[IWER: Show wordlist at a slow, steady rate, approximately one word every two seconds]

[IWER: IfR indicates he/she cannot read: /:

TELL THE R: I will read the words for you and you repeat them after me

Read the words out loud as you show the wordlist in a slow, steady rate, approximately one word every seconds

R should repeat the word after you read it our loud, before moving to the next word
]

[PROGRAMMER:

Word sequence: Butter, Arm, Shore, Letter, Queen, Cabin, Pole, Ticket, Grass, Engine

Display one word at a time in landscape manner, the word should occupy the whole screen]

DC028_W4 Now please tell me the words you can recall.

[IWER: PERMIT as much time as R wishes – up to about 2 minutes

Please select words that is being correctly recalled by respondent (Select all that apply) ( )]

1. Butter
2. Arm
3. Shore
4. Letter
5. Queen
6. Cabin
7. Pole
8. Ticket
9. Grass
10. Engine
11. None
12. RF → Skip to DC031_W4 DC031_W4

[INTRO: I am going to show you the same list of words in a different order. Read each word out loud as I show it to you. Later I will ask you to recall all ten words. Are you ready?]

[IWER: Show wordlist at a slow, steady rate, approximately one word every two seconds 1 – 2]

[IWER: IfR indicates he/she cannot read: /:

TELL THE R: I will read the words for you and you repeat them after me

Read the words out loud as you show the wordlist in a slow, steady rate, approximately one word every seconds

R should repeat the word after you read it our loud, before moving to the next word]

[PROGRAMMER:

Word sequence: Pole, Letter, Butter, Queen, Arm, Shore, Grass, Cabin, Ticket, Engine

Display one word at a time in landscape manner, the word should occupy the whole screen]

DC029_W4 Now please tell me the words you can recall

[IWER: PERMIT as much time as R wishes – up to about 2 minutes

Please select words that is being correctly recalled by respondent (Select all that apply) ( )]

1. Pole
2. Letter
3. Butter
4. Queen
5. Arm
6. Shore
7. Grass
8. Cabin
9. Ticket
10. Engine
11. None
12. RF → Skip to DC031_W4 DC031_W4

[INTRO: I am going to show you the same list of words in a different order. Read each word out loud as I show it to you. Later I will ask you to recall all ten words. Are you ready?]

[IWER: Show wordlist at a slow, steady rate, approximately one word every two seconds 1 – 3]

[IWER: IfR indicates he/she cannot read: /:

TELL THE R: I will read the words for you and you repeat them after me

Read the words out loud as you show the wordlist in a slow, steady rate, approximately one word every seconds

R should repeat the word after you read it our loud, before moving to the next word]

[PROGRAMMER:

Word sequence: Shore, Letter, Arm, Cabin, Pole, Ticket, Engine, Grass, Butter, Queen

Display one word at a time in landscape manner, the word should occupy the whole screen]

DC030_W4 Now please tell me the words you can recall.

[IWER: PERMIT as much time as R wishes – up to about 2 minutes

Please select words that is being correctly recalled by respondent(Select all that apply) （）]

1. Shore
2. Letter
3. Arm
4. Cabin
5. Pole
6. Ticket
7. Engine
8. Grass
9. Butter
10. Queen
11. None
12. RF

DC031_W4 [IWER: Indicate whether any of the following apply to the administation of the word lists (select all that apply) （）]

1. Administered verbally
2. An interrution occured during administration of list
3. Respondent had difficulty reading the words
4. No issues occured

SECTION RF Retrieval Fluency

RF INTRODUCTION ANIMAL NAMING RF

[This test can not only assess the degree of impairment of oral expression, but also measure semantic memory and language skills. Tell R I will tell you a certain category, please say names belonging to this category as many as you can, the sooner the better. For example: when I say types of clothing, you can say shirts, ties or hats and so on. Can you think of other names in the category of clothing?

Wait for the respondent to say two names. If the respondent succed, then announce the answer is correct and start the test. If the respondent says the wrong name or responds incorrectly, please correct him/her and repeat the instruction. If the respondent cannot answer, repeat the instruction. If the interviewee apparently cannot understand the guideline, stop the task and ask why

After making sure that the respondent understand the test and say two names correctly, please say Now I want to see how many different animals you can name. You will have 60 seconds. When I say, Begin, say the animal names as fast as you can. 60]

[IWER: Get ready to time 60 seconds. Repeat instructions if necessary. If the respondent stopped before the time was up, they can be encouraged to try to figure out more animal names. If the respondent stop for more than 15 secons, repeat the instrucion I want you to name as many animal names as you can. Even though the instruction is repeated during the test, the time should mot be prolonged 60 15 ( )]

[IWER: The scores are number of animals the respondet name every 15 seconds. As long as they do not belong to minerals and plants can be regarded as animals. Only real animals (including the dragon) are considered the correct answers, non-real animals (such as phoenix, unicorn, etc.) cannot be counted. Please exclude the names of duplicates, the nick-names of owners for animals, the names of animals of the same species of different colors, sex or cubs. Specificly, count categories of animals (E.G., dog), as well as specific types (E.G., collie, terrier) as correct 15 ( ) ( ) ( ) ( )]

[IWER: Please record the interview process, to facilitate the recording of all animal names after the test.After the test is finshed, please make a positive and negative judgement on the names of the animals listed, and enter the correct number on to tablet]

[IWER: Start timing: press [START TIMING] as soon as you tell the R to begin []]

RF101_Intro Are you ready? (PAUSE.) Begin （） !

1. → skip DC039_W4_1 DC039_W4_1
2.

DC039_W4_1 Please record reasons for not complete this test

1. Refuse or unwilling to do
2. Cannot speak in whole life
3. Cannot speak after getting old
4. Deaf or poor hearing
5. Other pleasespeify ______ (**DC039_W4_1_1**)

**PROCEDURE**
After DC039_W4_1 skip to SECTION CSI-D DC039_W4_1 SECTION CSI-D

[PROGRAMMER: DONT KNOW/DK and REFUSE/RF are not recorded for retrieval fluency test]

[IWER: Every time when respondent speak out a name, interviewer should press ENTER. If the name is correct, interviewer make a mark on paper. After the 60 second end, record all answers on system 60]

DC032_W4 System record the total number of animal names during 0-15 seconds 0-15 _____

DC033_W4 The amount of correct names during 0-15 seconds0-15 _______

DC034_W4 System record the total number of animal names during 16-30 seconds 16-30 ___

DC035_W4 The amount of correct names during 16-30 seconds16-30 _______

DC036_W4 System record the total number of animal names during 31-45 seconds 31-45 ___

DC037_W4 The amount of correct names during 31-45 seconds31-45 _______

DC038_W4 System record the total number of animal names during 46-60 seconds 46-60 ___

DC039_W4 The amount of correct names during 46-60 seconds46-60 _______

SECTION CSI-D Community Screening Instrument for Dementia Interviewee Part -

DC042_W4 [IWER: Point to your elbow and say] What do we call this?

1. Correct
5. Error
97. Cannot answer

DC043_W4 What do you do with a hammer?

[IWER: Acceptable responses are: pound, to drive a nail into something, to pound something , to hit something with]

1. Correct
5. Error
97. Cannot answer

DC044_W4 Where is the local market/ local store? /

[IWER: Can you be more specific if the Respondent uses vague answer]

1. Clearly indicate the route local market/ local store /
2. Address of local market/ local store /
3. Name of local market/ local store /
4. A vague answer
5. DK
97. Cannot answer

DC045_W4 Please point first to the window and then to the door 1. R point to the window and then the door

2. R just point to window – there is no door nearby – 3. R just point to door – there is no window nearby – 4. Not applied

5. Incorrect action
97. Cannot answer

SECTION CESD Depression

[INTRO: The 10 items below refer to how you have felt and behaved during the last week. Every item has the same selective answers including rarely or none of the time, some, occasionally, and most or all of the time. Choose the appropriate response 10]

DC009 I was bothered by things that dont usually bother me

1. Rarely or none of the time （<1 day）
2. Some or a little of the time （1-2 days）
3. Occasionally or a moderate amount of the time （3-4 days）
4. Most or all of the time （5-7 days）
8. DK
9. RF

DC010 I had trouble keeping my mind on what I was doing

1. Rarely or none of the time （<1 day）
2. Some or a little of the time （1-2 days）
3. Occasionally or a moderate amount of the time （3-4 days）
4. Most or all of the time （5-7 days）
8. DK
9. RF

DC011 I felt depressed

1. Rarely or none of the time （<1 day）
2. Some or a little of the time （1-2 days）
3. Occasionally or a moderate amount of the time （3-4 days）
4. Most or all of the time （5-7 days）
8. DK
9. RF

DC012 I felt everything I did was an effort

1. Rarely or none of the time （<1 day）
2. Some or a little of the time （1-2 days）
3. Occasionally or a moderate amount of the time （3-4 days）
4. Most or all of the time （5-7 days）
8. DK
9. RF

DC013 I felt hopeful about the future

1. Rarely or none of the time （<1 day）
2. Some or a little of the time （1-2 days）
3. Occasionally or a moderate amount of the time （3-4 days）
4. Most or all of the time （5-7 days）

8. DK
9. RF

DC014 I felt fearful

1. Rarely or none of the time （<1 day ）
2. Some or a little of the time （1-2 days ）
3. Occasionally or a moderate amount of the time （3-4 days）
4. Most or all of the time （5-7 days ）
8. DK
9. RF

DC015 My sleep was restless

1. Rarely or none of the time （<1 day ）
2. Some or a little of the time （1-2 days ）
3. Occasionally or a moderate amount of the time （3-4 days）
4. Most or all of the time （5-7 days ）
8. DK
9. RF

DC016 I was happy

1. Rarely or none of the time （<1 day ）
2. Some or a little of the time （1-2 days ）
3. Occasionally or a moderate amount of the time （3-4 days）
4. Most or all of the time （5-7 days ）
8. DK
9. RF

DC017 I felt lonely

1. Rarely or none of the time （<1 day ）
2. Some or a little of the time （1-2 days ）
3. Occasionally or a moderate amount of the time （3-4 days）
4. Most or all of the time （5-7 days ）
8. DK
9. RF

DC018 I could not get going

1. Rarely or none of the time （<1 day ）
2. Some or a little of the time （1-2 days ）

3. Occasionally or a moderate amount of the time （3-4 days）
4. Most or all of the time （5-7 days）
8. DK
9. RF

SECTION SAT Satisfaction

DC028 Please think about your life-as-a-whole. How satisfied are you with it? Are you completely satisfied, very satisfied, somewhat satisfied, not very satisfied, or not at all satisfied?

1. Completely satisfied
2. Very satisfied
3. Somewhat satisfied
4. Not very satisfied
5. Not at all satisfied

DC042_W3 How satisfied are you with your health?

1. Completely satisfied
2. Very satisfied
3. Somewhat satisfied
4. Not very satisfied
5. Not at all satisfied

PROCEDURE
If BE001 = 1, 2, ask DC043_W3

DC043_W3 How satisfied are you with your marriage (relationship with spouse)?

1. Completely satisfied
2. Very satisfied
3. Somewhat satisfied
4. Not very satisfied
5. Not at all satisfied
6. No spouse now

DC044_W3 How satisfied are you with your relationship with children?
[IWE: Only for respndents who have living offspring]

1. Completely satisfied
2. Very satisfied
3. Somewhat satisfied
4. Not very satisfied
5. Not at all satisfied
6. No child now

DC046_W4 How satisfied are you with the air quality this year? Are you completely satisfied, very satisfied, somewhat satisfied, not very satisfied, or not at all satisfied?

1. Completely satisfied
2. Very satisfied
3. Somewhat satisfied
4. Not very satisfied
5. Not at all satisfied

SECTION DR Delayed Recall

PROCEDURE

If respondent refuse to do WR101_INTRO, or DC028_W4s12 = 1, skip to SECTION NS

WR101_INTROL DC028_W4s12 = 1, SECTION NS

[INTRO: A few minutes ago I asked you to learn a list often words which you read one at a time from cards. Now I want you to try to recall as many of those 10 words as you can. OK, now tell me as many of those ten words as you can remember]

[IWER: PERMIT as much time as R wishes – up to about 2 minutes]

DC047_W4 Please select words that is being correctly recalled by respondent

1. Shore
2. Letter
3. Arm
4. Cabin
5. Pole
6. Ticket
7. Engine
8. Grass
9. Butter
10. Queen

11. None word being recalled
12. Refuse to recall

SECTION NS Number Series

[IWER: The following procedure requires the interviewer and the respondent to look at screen together, and following instructions. Respondent is allowed to use pencil and paper to calculate, but other aids such as a calculator will not be allowed to use]

Number Series First Example

INTRO: Next Im going to show you several numbers on the screen. There will be a blank number in the series with a ? mark. Please look at the numbers from left to right and try to find their pattern. Based on this pattern, tell me what number goes in the blank. Sometimes the blank will be at the end of the series, and sometimes the blank will be in the beginning or in the middle. Now can we start with an example to illustrate

Please look at this group of numbers, what number should go into the blank?

3	4	5	?

DC029_W4_1 Record answer from respondent: 345______ (**DC029_W3_1**) 6. R doesnt understand instructions

7. R cant write / no paper / pen available → Skip to END OF SECTION NS
8. DK
9. RF

[IWER :

IfR does not give the Correct response (6) then say: the answer we were looking for is 6. Then ask R do you understand the direction for this test?, in order to check that the R understand the task 6

If the respondent says he or she does not konw the answer, record DK.Do no record a DKresponse as R doesntunderstand instructions]

Number Series Second Example

Next, show R the second example on the card and say: Lets try another one. What number goes in the blank based on the pattern of numbers?

7	6	?	4

DC030_W4_1 Record answer from respondent: 76______4 (**DC030_W3_1**) 6. R doesnt understand instructions

8. DK
9. RF

[IWER :

IfR does not give the Correct response (5) then say: the answer we were looking for is 5 5

If the respondent sys he or she does not konw the answer, record DK.Do no record a DKresponse as R doesntunderstand instructions

]

DC031_W4_0 [IWER: Now, ask respondent do you understand the directions for this task? To see if respondent actually konw the rule in this task γ]

1. Continue
5. R seems confused or does not understand task
8. DK → Skip to END OF SECTION NS
9. RF → Skip to END OF SECTION NS

[INTRO: I am now going to show you six more questions like the one you just did. Sometimes the blank will be at the end of the series, and sometimes it may be at the beginning or in the middle. You maybe asked a question with more than one blank in the sequence. The numbers might increase, like 1, 2, 3, or decrease, like 3, 2, 1. Some of the problems maybe easy but others may be hard. Just do the best you can 123 321

It is more important to answer the item correctly than to answer quickly, so take a little time to think before answering. It is okay if you do not know the answer because some of the items are intended to be very difficult. You can go on to the next item at any time. Are you ready to begin?]

[IWER: Permit as much time as R wishes for each question. If the respondent says he or she does not know the answer, record DK. Do not record A Dont Know response as R doesnt

understand instructions. If the R has not given an answer after about a minute, ask: would you just like to go on to the next quesion?]

[IWER: Do not give any hit and do not tell R whether his/her answer is correct or not]

**PROCEDURE**<br>All respondents answer DC031_W3_1, DC031_W3_2, DC031_W3_3<br>DC031_W3_1DC031_W3_2DC031_W3_3

DC031_W3_1 Number Series

7	8	?	10

[IWER: Correct response 9]

DC031_W4_1 Record answer from respondent: 78______ (**DC031_W3_1**)10

[IWER: If the respondent says not know the answer, record DK., do not record a dont know response as unable to do]

7. R unable to do → Skip to END OF SECTION NS
8. DK
9. RF

DC031_W3_2 Number Series

8	?	12	14

[IWER: Correct response 10]

DC031_W4_2 Record answer from respondent: 8______ (**DC031_W3_2**)1214

[IWER: If the respondent says not know the answer, record DK]

8. DK
9. RF

DC031_W3_3 Number Series

18	10	6	?	3

[IWER: Correct response 4]

DC031_W4_3 Record answer from respondent: 18106______ (**DC031_W3_3**)3

[IWER: If the respondent says not know the answer, record DK]

8. DK
9. RF

PROCEDURE

If respondent answered none of the STARTING BLOCK correctly, continue on to DC032_W3_1, DC032_W3_2, DC032_W3_3 STARTING BLOCK 0 DC032_W3_1DC032_W3_2DC032_W3_3

DC032_W3_1 Number Series

1	2	3	?

[IWER: Correct response 4]

DC032_W4_1 Record answer from respondent: 123______ (**DC032_W3_1**)

[IWER: If the respondent says not know the answer, record DK]

8. DK
9. RF

DC032_W3_2 Number Series

6	5	4	?

[IWER: Correct response 3]

DC032_W4_2 Record answer from respondent: 654______ (**DC032_W3_2**)

[IWER: If the respondent says not know the answer, record DK]

8. DK
9. RF

DC032_W3_3 Number Series

12	?	16	18

[IWER: Correct response 14]

DC032_W4_3 Record answer from respondent: 12______ (**DC032_W3_3**)1618

[IWER: If the respondent says not know the answer, record DK]

8. DK
9. RF

PROCEDURE

After finishing DC032_W3_1, DC032_W3_2, DC032_W3_3, Skip to END OF SECTION NS DC032_W3_1DC032_W3_2DC032_W3_3

If respondent got one answer correctly in the STARTING BLOCK, continue on to DC033_W3_1, DC033_W3_2, DC033_W3_3 STARTING BLOCK 1 DC033_W3_1DC033_W3_2DC033_W3_3

DC033_W3_1 Number Series

5	?	3	2

[IWER: Correct response 4]

DC033_W4_1 Record answer from respondent: 5______ (**DC033_W3_1**)32

[IWER: If the respondent says not know the answer, record DK]

8. DK
9. RF

DC033_W3_2 Number Series

4	7	10	?

[IWER: Correct response 13]

DC033_W4_2 Record answer from respondent: 4710______ (**DC033_W3_2**)

[IWER: If the respondent says not know the answer, record DK]

8. DK
9. RF

DC033_W3_3 Number Series

?	4	6	8

[IWER: Correct response 2]

DC033_W4_3 Record answer from respondent: ______ (**DC033_W3_3**)468

[IWER: If the respondent says not know the answer, record DK]

8. DK
9. RF

PROCEDURE

After finshing DC033_W3_1, DC033_W3_2, DC033_W3_3, Skip to END OF SECTION NS DC033_W3_1DC033_W3_2DC033_W3_3

If respondent got two answers correctly in the STARTING BLOCK, continue on to DC034_W3_1, DC034_W3_2, DC034_W3_3 STARTING BLOCK 2 DC034_W3_1DC034_W3_2DC034_W3_3

DC034_W3_1 Number Series

1	3	3	5	7	7	?

[IWER: Correct response 9]

DC034_W4_1 Record answer from respondent: 133577_____ (**DC034_W3_1**)

[IWER: If the respondent says not know the answer, record DK]

8. DK
9. RF

DC034_W3_2 Number Series

3	?	8	12	17

[IWER: Correct response 5]

DC034_W4_2 Record answer from respondent: 3______ (**DC034_W3_2**)81217

[IWER: If the respondent says not know the answer, record DK]

8. DK
9. RF

DC034_W3_3 Number Series

17	?	12	8

[IWER: Correct response 15]

DC034_W4_3 Record answer from respondent: 17______ (**DC034_W3_3**)128

[IWER: If the respondent says not know the answer, record DK]

8. DK
9. RF

PROCEDURE

After finishing DC034_W3_1, DC034_W3_2, DC034_W3_3, Skip to END OF SECTION NS DC034_W3_1DC034_W3_2DC034_W3_3

If respondent got all three answers correctly in the STARTING BLOCK, continue on to DC035_W3_1, DC035_W3_2, DC035_W3_3 STARTING BLOCK 3 DC035_W3_1DC035_W3_2DC035_W3_3

DC035_W3_1 Number Series

10	?	3	1

[IWER: Correct response 6]

DC035_W4_1 Record answer from respondent: 10______ (**DC035_W3_1**)31

[IWER:If the respondent says not know the answer, record DK]

8. DK
9. RF

DC035_W3_2 Number Series

18	17	15	?	8

[IWER: Correct response 12]

DC035_W4_2 Record answer from respondent: 181715______ (**DC035_W3_2**)8

[IWER:If the respondent says not know the answer, record DK]

8. DK
9. RF

DC035_W3_3 Number Series

3	3	4	6	6	7	?	?

[IWER: Correct response 99]

DC035_W4_3 Record answer from respondent: 334667_____ (**DC035_W3_3_1**) ____ (**DC035_W3_3_2**)

[IWER:If the respondent says not know the answer, record DK]

8. DK
9. RF

[END OF SECTION NS]

SECTION WRE Wordlist Recognition

PROCEDURE

If respondent refuse to do WR101_INTRO, or DC028_W4s12 = 1, skip to DD

WR101_INTROL DC028_W4s12 = 1DD

WRE_Intro [INTRO: Now I am going to show you a set of words printed on cards. Some of the words are from the list you saw earlier and some are words I havent shown you before. I want you to tell me which words are from the list you saw earlier. Do you have any questions? Are you ready?]

1. Yes
2. No → Skip to DC – Interview Observation DC

[PROGRAMMER: Display one word at a time in landscape manner, the word should occupy the whole screen]

[IWER: Show next word. If R is unable to read, say the word Church out loud while still showing the card]

DC048_W4 Is this one of the words you saw earlier?

1 = Yes ; 5 = No ; 8 = DK

[IWER: Show next word. IfR is unable to read, say the word Coffee out loud while still showing the card]

DC049_W4 Is this one of the words you saw earlier?

1 = Yes ; 5 = No ; 8 = DK

[IWER: Show next word. IfR is unable to read, say the word Butter out loud while still showing the card]

DC050_W4 Is this one of the words you saw earlier?

1 = Yes ; 5 = No ; 8 = DK

[IWER: Show next word. IfR is unable to read, say the word Dollar out loud while still showing the card]

DC051_W4 Is this one of the words you saw earlier?

1 = Yes ; 5 = No ; 8 = DK

[IWER: Show next word. IfR is unable to read, say the word Arm out loud while still showing the card]

DC052_W4 Is this one of the words you saw earlier?

1 = Yes ; 5 = No ; 8 = DK

[IWER: Show next word. IfR is unable to read, say the word Shore out loud while still showing the card]

DC053_W4 Is this one of the words you saw earlier?
1 = Yes ; 5 = No ; 8 = DK

[IWER: Show next word. IfR is unable to read, say the word Five out loud while still showing the card]

DC054_W4 Is this one of the words you saw earlier?
1 = Yes ; 5 = No ; 8 = DK

[IWER: Show next word. IfR is unable to read, say the word Letter out loud while still showing the card]

DC055_W4 Is this one of the words you saw earlier?
1 = Yes ; 5 = No ; 8 = DK

[IWER: Show next word. IfR is unable to read, say the word Hotel out loud while still showing the card]

DC056_W4 Is this one of the words you saw earlier?
1 = Yes ; 5 = No ; 8 = DK

[IWER: Show next word. If R is unable to read, say the word Mountain out loud while still showing the card]

DC057_W4 Is this one of the words you saw earlier?
1 = Yes ; 5 = No ; 8 = DK

[IWER: Show next word. IfR is unable to read, say the word Queen out loud while still showing the card]

DC058_W4 Is this one of the words you saw earlier?
1 = Yes ; 5 = No ; 8 = DK

[IWER: Show next word. IfR is unable to read, say the word Cabin out loud while still showing the card]

DC059_W4 Is this one of the words you saw earlier?
1 = Yes ; 5 = No ; 8 = DK

[IWER: Show next word. If R is unable to read, say the word Slipper out loud while still showing the card]

DC060_W4 Is this one of the words you saw earlier?

1 = Yes ; 5 = No ; 8 = DK

[IWER: Show next word. IfR is unable to read, say the word Pole out loud while still showing the card]

DC061_W4 Is this one of the words you saw earlier?

1 = Yes ; 5 = No ; 8 = DK

[IWER: Show next word. IfR is unable to read, say the word Village out loud while still showing the card]

DC062_W4 Is this one of the words you saw earlier?

1 = Yes ; 5 = No ; 8 = DK

[IWER: Show next word. IfR is unable to read, say the word String out loud while still showing the card]

DC063_W4 Is this one of the words you saw earlier?

1 = Yes ; 5 = No ; 8 = DK

[IWER: Show next word. IfR is unable to read, say the word Ticket out loud while still showing the card]

DC064_W4 Is this one of the words you saw earlier?

1 = Yes ; 5 = No ; 8 = DK

[IWER: Show next word. If R is unable to read, say the word Troops out loud while still showing the card]

DC065_W4 Is this one of the words you saw earlier?

1 = Yes ; 5 = No ; 8 = DK

[IWER: Show next word. IfR is unable to read, say the word Grass out loud while still showing the card]

DC066_W4 Is this one of the words you saw earlier?

1 = Yes ; 5 = No ; 8 = DK

[IWER: Show next word. If R is unable to read, say the word Engine out loud while still showing the card]

DC067_W4 Is this one of the words you saw earlier?

1 = Yes ; 5 = No ; 8 = DK

END_DC Interview Observation DC

DC068_W4 Does any of the following happen during the interview?

1. Poor eyesight
2. Poor hearing withour hearing-aid
3. Wearing hearing-aid
4. Shaking hands
5. The interview is interrupted by some stuff or noises
6. The quality of the interview is doubted due to some emotional problems of R
7. Others, please specify ______ （**Other_ Interviewee2**） 8. None

DC069_W4 What kind of language is used during the interview? 1. Mandarin

2. Local dialect
3. Other, please specify ______ （**LANGUAGE_OTHER**）

DC070_W4 Whether hired local translators during interview because of language barrier?

1. Yes
2. No

This page intentionally left blank

DD Informants Information

PROCEDURE
If respondent aged less than 60, skip this section 60

INTRO_INFSELECT [INTRO: We would also like you to nominate a person, relative or friend, who can complete a short interview of about twenty minutes about you. This person should be someone who knows you well and interacts with you frequently. This person will receive $25 as a token of appreciation for his/her interview / /

We would like you to nominate up to three people in order of preference that you would like us to contact. We will contact the second or third person only if we are unable to complete the interview with your first nominee]

1. Continue
2. Respondent doesnt have any informant to give [END INTERVIEW] → END

INF1_Name What is the first persons name? _______

INF1_RTR What is this persons relationship to you?

1. Spouse/Partner /
2. Child
3. Grandchild /
4. Sibling
5. Parent
6. Friend
7. Guardian
8. Neighbor
9. Other please specify ______ (**INF1_RTR_OTHER**)

NOTICE1 [IWER: Does the respondent have a second informant nominee to give?]

1. Yes → Skip to INF2_Name INF2_Name
2. No → Skip to INFORMANT SELECTION INFORMANT SELECTION

INF2_Name What is the second persons name? _______

INF2_RTR What is this persons relationship to you? 1. Spouse/Partner /

2. Child

3. Grandchild /
4. Sibling
5. Parent
6. Friend
7. Guardian
8. Neighbor
9. Other please specify ______ (**INF2_RTR_OTHER**)

NOTICE2 [IWER:Does the respondent have a third informant nominee to give?]
1. Yes → Skip to INF3_Name INF3_Name
2. No → Skip to INFORMANT SELECTION INFORMANT SELECTION

INF3_Name What is the third persons name? ________

INF3_RTR What is this persons relationship to you? 1. Spouse/Partner /
2. Child
3. Grandchild /
4. Sibling
5. Parent
6. Friend
7. Guardian
8. Neighbor
9. Other please specify ______ (**INF3_RTR_OTHER**)

[IWER: Suspend blaise and contact informant nominee to schedule the interview]

INFORMANT_SELECTION – [IWER: Who is your selected informant]
1. INF1 [INF1_Name] → Skip to VOL_STMT_IF VOL_STMT_IF
2. INF2 [INF2_Name] → Skip to INF1_NOIWREASON INF1_NOIWREASON
3. INF3 [INF3_Name] → Skip to INF1_NOIWREASON INF1_NOIWREASON

97. Other informant → Skip to INFOTHER_ REASONINFOTHER_ REASON

INF1_NOIWREASON [IWER: Why was INFORMANT 1 not selected for the interviwe? 1 [I NF1_Name]]
1. Refused 1
2. No contact
3. Health reasons 1
4. Not kowledgeable about R

5. Other please specify ______ (**INF1_NOIWREASON_OTHER**)

**PROCEDURE**
If INFORMANT_SELECTION = 3, then skip to INF2_NOIWEASON INFORMANT_SELECTION = 3 INF2_NOIWREASON

INF2_NOIWREASON IWER: Why was INFORMANT 2 not selected for the interviwe? 2

1. Refused 2
2. No contact
3. Health reasons 2
4. Not kowledgeable about R
5. Other, specify ______ (**INF2_NOIWREASON_OTHER**)

**PROCEDURE**
If INFORMANT_SELECTION = 97, then skip to INFOTHER_REASON. INFORMANT_SELECTION = 97 INFOTHER_REASON

INFOTHER_REASON IWER: Why was another informant selected for the interviwe?

1. Other please specify ______ (**INFOTHER_REASON_Other**)
2. None of the original informant nominees worked out

INFOTHER_Name The name of the informant is _______

SECTION INTRO IF Introducing Informant Interview

VOL_STMT_IF [INTRO: Before we begin, I want you to know that this interview is completely voluntary. If we should come to any question that you dont want to answer, just let me know and I will go on to the next question. Your identity as a participant and any personally identifying information you provide will be kept confidential]

[INTRO: Thankyou for agreeing to participate in this study. For this interview I will be asking you a series of thing about [preload R name], including his/her ability to complete memory, thinking, and other tasks [ ] /]

SECTION DEMOGRAPHICS_IF Demographics of Informant

DD001_W4 What is your age? ______ 18...110

DD002_W4 [IWER: indicate informant gender]

1. Male
2. Female

DD003_W4 What is the highest grade of school or year of college you completed?

1. No formal education （）
2. Did not finish primary school
3. Sishu/home school
4. Elementary school
5. Middle schoo
6. High school
7. Vocational school （）
8. Two-/Three-Year College/Associate degree
9. Four-Year College/Bachelors degree
10. Masters degree
11. Doctoral degree/Ph.D.

DD004_W4 What is your relationship with [RESPONDENT NAME]? []

1. Spouse/Partner → Skip to DD005_W4 DD005_W4
2. Child → Skip to DD006_W4 DD006_W4
3. Grandchild → Skip to DD005_W4 DD005_W4
4. Sibling → Skip to DD006_W4 DD006_W4
5. Parent → Skip to DD006_W4 DD006_W4
6. Friend → Skip to DD005_W4 DD005_W4
7. Guardian → Skip to DD005_W4 DD005_W4
8. Neighbor → Skip to DD005_W4 DD005_W4
9. Other please specify ______ (**DD004_W4_1**) → Skip to DD005_W4 DD005_W4

PROCEDURE

Assign DD005_W4 = 10 ifDD004_W4 = 2 (Child), 4 (Sibling), 5 (Parent) DD004_W4 = 2 4 5 DD005_W4 10

DD005_W4 How many years have you known [RESPONDENT NAME]? [] ______ 1...100 （）

DD006_W4 On average in the past year how often did you see [RESPONDENT NAME]? [ ]

1. Lives with respondent
2. Daily
3. Several times/week /
4. Once a week
5. One-three times a month1-3 /
6. LEess than once a month<1 /
7. Never
8. Other please specify ______ (**DD006_W4_1**)

DD007_W4 Are you a caregiver for [Respondent]? []

1 Yes
2 No

DD008_W4 Has [Respondent] been diagnosed with stroke? [] 1. Yes

2. No
9. DK

DD009_W4 Has [Respondent] been diagnosed with Parkinsons disease? []

1. Yes
2. No
9. DK

DD010_W4 Has [Respondent] been diagnosed with Alzheimers disease? []

1. Yes
2. No
9. DK

DD011_W4 Has [Respondent] been diagnosed with memory problems? []

1. Yes
2. No
9. DK

SECTION JORM IQCODE The Jorm Informant Quesionnaire on Cognitive Decline in the Elderly

[INTRO: We want you to remember what [R NAME] was like [DD005_W4/ 10 ifDD005_W4 > 10] years ago and to compare it with what (he/she) is like now. DD005_W4 / Ten years ago was in

[current year – DD005_W4/ 10 if DD005_W4 > 10] [] [DD005_W4DD005_W4> 10 10] /[DD005_W4 10] [– DD005_W4 DD005_W4 > 10 10]

First, we want to confirm [R NAME]s memory and intelligence with you, regarding his/her memory on Daily rountines and past situations. Please note the importance of comparing (his/her) present performance with [DD005_W4 / 10 if DD005_W4 > 10] years ago. So if [DD005_W4/ 10 if DD005_W4 > 10] years ago [R NAME] always forgot where (he/she) had left things, and (he/she) still does, then this would be considered Not much changed [] / [] / [DD005_W4 DD005_W4 > 10 10] / [DD005_W4 10 DD005_W4 > 10] For DD005_W4 = DK or RF, display 10 years. DD005_W4 = 10]

DD012_W4 Compared with [DD005_W4 / 10 if DD005_W4 > 10] years ago, how is [R NAME] at recognizing the face of family members and friends? [DD005_W4 10 DD005_W4 > 10] [] ?

1. Much better
2. Improved
3. Not much changed
4. Gotten worse
5. Much worse
6. Does not apply; R doesnt do activity
8. DK
9. RF

DD013_W4 Compared with [DD005_W4 / 10 if DD005_W4 > 10] yearsago, how is [R NAME] at recognizing the name of family members and friends? [DD005_W4 10 DD005_W4 > 10] [] ?

1. Much better
2. Improved
3. Not much changed
4. Gotten worse
5. Much worse
6. Does not apply; R doesnt do activity
8. DK
9. RF

DD014_W4 Compared with [DD005_W4 / 10 if DD005_W4 > 10] years ago, how is [R NAME] at remembering things about family and friends, such as occupations, birthdays, and addresses? [DD005_W4 10 DD005_W4 > 10] [] ?

1. Much better
2. Improved
3. Not much changed
4. Gotten worse
5. Much worse
6. Does not apply; R doesnt do activity
8. DK
9. RF

DD015_W4 Compared with [DD005_W4 / 10 if DD005_W4 > 10] years ago how is [R NAME] at Remembering things that have happened recently? [DD005_W4 10 DD005_W4 > 10] [] ?

1. Much better
2. Improved
3. Not much changed
4. Gotten worse
5. Much worse
6. Does not apply; R doesnt do activity
8. DK
9. RF

DD016_W4 Compared with [DD005_W4 / 10 if DD005_W4 > 10] years ago how is [R NAME] at recalling conversations a few days later? [DD005_W4 10 DD005_W4 > 10] [] ?

1. Much better
2. Improved
3. Not much changed
4. Gotten worse
5. Much worse
6. Does not apply; R doesnt do activity
8. DK
9. RF

DD017_W4 Compared with [DD005_W4 / 10 if DD005_W4 > 10] years ago how is [R NAME] at forgetting what was about to say in the middle of conversations? [DD005_W4 10 DD005_W4 > 10] [] ?

1. Much better
2. Improved
3. Not much changed
4. Gotten worse
5. Much worse
6. Does not apply; R doesnt do activity

8. DK
9. RF

DD018_W4 Compared with [DD005_W4 / 10 if DD005_W4 > 10] years ago how is [R NAME] at remembering [his/her] address and telephone number? [DD005_W4 10 DD005_W4 > 10] [] ?

1. Much better
2. Improved
3. Not much changed
4. Gotten worse
5. Much worse
6. Does not apply; R doesnt do activity
8. DK
9. RF

DD019_W4 Compared with [DD005_W4 / 10 if DD005_W4 > 10] years ago how is [R NAME] at remembering what day and month it is? [DD005_W4 10 DD005_W4 > 10] [] ?

1. Much better
2. Improved
3. Not much changed
4. Gotten worse
5. Much worse
6. Does not apply; R doesnt do activity
8. DK
9. RF

DD020_W4 Compared with [DD005_W4 / 10 if DD005_W4 > 10] years ago how is [R NAME] at remembering where things are usually kept? [DD005_W4 10 DD005_W4 > 10] [] ?

1. Much better
2. Improved
3. Not much changed
4. Gotten worse
5. Much worse
6. Does not apply; R doesnt do activity
8. DK
9. RF

DD021_W4 Compared with [DD005_W4 / 10 if DD005_W4 > 10] years ago how is [R NAME] at remembering where to find things which have been put in a different place from usual? [DD005_W4 10 DD005_W4 > 10] [] ?

1. Much better
2. Improved
3. Not much changed
4. Gotten worse
5. Much worse
6. Does not apply; R doesnt do activity
8. DK
9. RF

DD022_W4 Compared with [DD005_W4 / 10 if DD005_W4 > 10] years ago how is [R NAME] at adapting changes in Daily life? [DD005_W4 10 DD005_W4 > 10] [ ] ?

1. Much better
2. Improved
3. Not much changed
4. Gotten worse
5. Much worse
6. Does not apply; R doesnt do activity
8. DK
9. RF

DD023_W4 Compared with [DD005_W4 / 10 if DD005_W4 > 10] years ago how is [R NAME] at knowing how to work familiar machines around the house? [DD005_W4 10 DD005_W4 > 10] [] ( ) ?

1. Much better
2. Improved
3. Not much changed
4. Gotten worse
5. Much worse
6. Does not apply; R doesnt do activity
8. DK
9. RF

DD024_W4 Compared with [DD005_W4 / 10 if DD005_W4 > 10] years ago how is [R NAME] at learning to use a new gadget or machine around house? [DD005_W4 10 DD005_W4 > 10] [] ?

1. Much better
2. Improved
3. Not much changed
4. Gotten worse
5. Much worse
6. Does not apply; R doesnt do activity

8. DK
9. RF

DD025_W4 Compared with [DD005_W4 / 10 if DD005_W4 > 10] years ago how is [R NAME] at learning new things in general? [DD005_W4 10 DD005_W4 > 10] [] ?

1. Much better
2. Improved
3. Not much changed
4. Gotten worse
5. Much worse
6. Does not apply; R doesnt do activity
8. DK
9. RF

DD026_W4 Compared with [DD005_W4/ 10 ifDD005_W4> 10] years ago how is [R NAME] at remembering childhood or adolescent memories? [DD005_W4 10 DD005_W4 > 10] [] ?

1. Much better
2. Improved
3. Not much changed
4. Gotten worse
5. Much worse
6. Does not apply; R doesnt do activity
8. DK
9. RF

DD027_W4 Compared with [DD005_W4 / 10 if DD005_W4 > 10] years ago how is [R NAME] at remembering things learned when he/she was young? [DD005_W4 10 DD005_W4 > 10] [] ?

1. Much better
2. Improved
3. Not much changed
4. Gotten worse
5. Much worse
6. Does not apply; R doesnt do activity
8. DK
9. RF

DD028_W4 Compared with [DD005_W4 / 10 if DD005_W4 > 10] years ago how is [R NAME] at recognizing words which were not commonly used? [DD005_W4 10 DD005_W4 > 10] [] ?

1. Much better
2. Improved
3. Not much changed
4. Gotten worse
5. Much worse
6. Does not apply; R doesnt do activity
8. DK
9. RF

DD029_W4 Compared with [DD005_W4 / 10 if DD005_W4 > 10] years ago how is [R NAME] at following articles in magazines? [DD005_W4 10 DD005_W4 > 10] [] ?

1. Much better
2. Improved
3. Not much changed
4. Gotten worse
5. Much worse
6. Does not apply; R doesnt do activity
8. DK
9. RF

DD030_W4 Compared with [DD005_W4 / 10 if DD005_W4 > 10] years ago how is [R NAME] at following a story in a book or on TV? [DD005_W4 10 DD005_W4 > 10] [] ?

1. Much better
2. Improved
3. Not much changed
4. Gotten worse
5. Much worse
6. Does not apply; R doesnt do activity
8. DK
9. RF

DD031_W4 Compared with [DD005_W4 / 10 if DD005_W4 > 10] years ago how is [R NAME] at writting letters? [DD005_W4 10 DD005_W4 > 10] [] ?

1. Much better
2. Improved
3. Not much changed
4. Gotten worse
5. Much worse
6. Does not apply; R doesnt do activity

8. DK
9. RF

DD032_W4 Compared with [DD005_W4 / 10 if DD005_W4 > 10] years ago how is [R NAME] at knowing some important historical events? [DD005_W4 10 DD005_W4 > 10] [] ?

1. Much better
2. Improved
3. Not much changed
4. Gotten worse
5. Much worse
6. Does not apply; R doesnt do activity
8. DK
9. RF

DD033_W4 Compared with [DD005_W4 / 10 if DD005_W4 > 10] years ago how is [R NAME] at making decisions on everyday matters? [DD005_W4 10 DD005_W4 > 10] [] ?

1. Much better
2. Improved
3. Not much changed
4. Gotten worse
5. Much worse
6. Does not apply; R doesnt do activity
8. DK
9. RF

DD034_W4 Compared with [DD005_W4 / 10 if DD005_W4 > 10] years ago how is [R NAME] at handling money for shopping? [DD005_W4 10 DD005_W4 > 10] [ ] ?

1. Much better
2. Improved
3. Not much changed
4. Gotten worse
5. Much worse
6. Does not apply; R doesnt do activity
8. DK
9. RF

DD035_W4 Compared with [DD005_W4 / 10 if DD005_W4 > 10] years ago how is [R NAME] at handling financial matters; for example, the pension, or dealing with the bank? [DD005_W4 10 DD005_W4 > 10] [] （ ） ?

1. Much better
2. Improved
3. Not much changed
4. Gotten worse
5. Much worse
6. Does not apply; R doesnt do activity
8. DK
9. RF

DD036_W4 Compared with [DD005_W4 / 10 if DD005_W4 > 10] years ago how is [R NAME] at handling other everyday arithmetic problems; for example, knowing how much food to buy, knowing how long between visits from family or friends? [DD005_W4 10 DD005_W4 > 10] [] ?

1. Much better
2. Improved
3. Not much changed
4. Gotten worse
5. Much worse
6. Does not apply; R doesnt do activity
8. DK
9. RF

DD037_W4 Compared with [DD005_W4 / 10 if DD005_W4 > 10] years ago how is [R NAME] at using his/her intelligence to understand whats going on and to reason things through? [DD005_W4 10 DD005_W4 > 10] [] ?

1. Much better
2. Improved
3. Not much changed
4. Gotten worse
5. Much worse
6. Does not apply; R doesnt do activity
8. DK
9. RF

SECTION BLESSED Blessed Dementia Scale Part II Blessed

[INTRO: Next I am going to ask you how well [R NAME] does with different activities []]

[INTRO: Please tell me the number you see on the screen that represents the correct answer]

DD039_W4 Regarding eating, would you say [R NAME] feeds (himself/herself) without assistance, with minor assistance, with much assistance, or has to be fed? [] /

1. Feeds self without assistance
2. Feeds self with monor assistance
3. Feeds self with much assistance
4. Has to be fed
8. DK
9. RF

[INTRO: Please tell me the number you see on the screen that represents the correct answer]

DD040_W4 Regarding using the toilet, would you say [R NAME] can clean and care for (himself/herself) at a toilet, has occasional incontinence or needs to be reminded, has frequent incontinence or needs much assistance, or has little or no control? []

1. Clean, cares for self at toilet

2. Occasional incontinence, or needs to be reminded 3. Frequent incontinence, or needs much assistance 4. Little or no control

8. DK
9. RF

[INTRO: Please tell me the number you see on the screen that represents the correct answer]

DD041_W4 Regarding dressing, would you say [R NAME] is able to get dressed Unaided, occasionally misplaces buttons, etc., and requires minor help, gets dressed with the wrong sequences, forgets items and requires much assistance, or is Unable to dress? []

1. Unaided
2. Occasionally misplaces buttons, etc., requires minor help
3. Wrong sequences, forgets items, requeres much assistance

4. Unable to dress
8. DK
9. RF

SECTION CSI-D Community Screening Instrument for Dementia Informant Part

DD042_W4 Comparing with his/her condition a few years ago, would you say [R NAME]s ability to speak is significantly worse off recently? []

1. No
2. Yes
8. DK
9. RF

DD043_W4 Comparing with his/her condition a few years ago, would you say [R NAME]s ability to think and understand is significantly worse off recently? [ ]

1. No
2. Yes
8. DK
9. RF

DD044_W4 Comparing with his/her condition a few years ago, would you say [R NAME] always forgot where he/she put things recently? []

1. No
2. Yes
8. DK
9. RF

DD045_W4 Comparing with his/her condition a few years ago, would you say [R NAME] always forgot what happened the day before yesterday recently? [ ]

1. No
2. Yes
8. DK
9. RF

DD046_W4 Comparing with his/her condition a few years ago, would you say [R NAME] sometimes could not recognize the current location? []

1. No
2. Yes
8. DK
9. RF

DD047_W4 Comparing with his/her condition a few years ago, would you say [R NAME] had difficulty dressing (for example, wrong button, could not dress or dress in the wrong sequence)? [] （ ）

[INTRO: If the difficulty is caused by physical disability, then choose No]

1. No
2. Yes
8. DK
9. RF

END_DD Interview Observation DD

[INTRO: Thankyou for completing the main interview！]

DD048_W4 Whether one of the following situations happened during the interview?

1. The informant hesitated on the questions regarding cognition and daily routines, because the respondent was on the scene
2. The quality of informant interview was questionable due to his/her cognition problems
3. The quality of informant interview was low due to his/her emotional problem
4. The interview was interrupted by noises or other stuff
5. Other please specify ______ (**DD048_W4_1**)
6. None of the above happed

DD049_W4 Language used during interview?

1. Mandarin
2. Local dialect
3. Other, please specify ______ (**DD049_W4_1**)

DD050_W4 Whether hired local translators during interview because of language barrier?

1. Yes

2. No

DD051_W4 Telephone interview or not? 1. Yes
2. No

This page intentionally left blank

E Health Care and Insurance

PART I Medical Insurance

[INTRO: Now we would like to know about health insurance or benefits that you might have]

EA001_W4 Are you the policyholder/primary beneficiary of any of the types of health insurance listed below? (circle all that apply) （） 1. Urban employee medical insurance (yi-bao) （）

2. Urban and rural resident medical insurance (integrated urban resident medical insurance and new rural cooperative medical insurance) （ ）
3. Urban resident medical insurance
4. New rural cooperative medical insurance (he-zuo-yi-liao) （ ）
5. Government medical insurance
6. Medical aid

7. Private medical insurance: purchased by work unit : 8. Private medical insurance: purchased by individual : 9. Urban non-employed personss health insurance 10. Long-term care insurance

11. Other medical insurance specify ______ (**EA001_1**) 12. No insurance → Skip to EA009 EA009

[Soft Check: If pick 12, cannot pick any other, you chose no insurance and a specific type of insurance, this is not possible 12

Cannot pick 2, 3, 4 at the same time, cannot pick 1 and 5 at the same time, cannot pick (2 or 3 or 4) and (1 or 5) at the same time, You choose contradictory insurances, please check 2 3 4 1 5 3 1]

[F1:（1） （2）

（3） 2007 7 79 （）

（4）

（5）

（6）

（7）

（8） （9）

（10）

（11），16 60 16 50， 700 600 100

（12）]

PROCEDURE

If EA001_W4 = 1 – 11, skip to Procedure beforeEA002 1-11 EA002

If EA001_W4 = 7, 8, skip EA002 EA002

EA002 Do you have supplemental insurance to this plan? （e.g., critical illness insurance, etc.)

( )

1. Yes
2. No

[F1]

PROCEDURE

For each circled type of insurance (1-11), ask the following questions EA003_W4 - EA008 1-11 EA003_W4 - EA008

EA003_W4 Where did you set up your insurance account/policy? ?

1. This county [Load the name of current living county] [ ]
2. (If it is not in this county) the place of your hukou （/） 3. Other (Province/City/County) // ______ (**EA003_W4_1**)

EA008 When did this benefit begin?

______ 1900...2018 (**EA008_1**) Year ______ 0...12 (**EA008_2**) Month

[IWER: Please estimate the year if respondents cannot recall the time clearly :

Mark the year using four digits. Take down the month as its actual number. For example, write January as 1 not 01, December as 12. If do not remember month, fill 0 : 4 1 10112 120]

**PROCEDURE**<br>If EA001_W4≠ 12, skip to PROCEDURE beforeEC001EC001

EA009 What is your main reason for not having health insurance? (circle all that apply) ()

1. I do not need it
2. Cannot afford it
3. Do not know where or from whom to get it
4. Do not trust the institutions that offer health insurance 5. Do not have suitable programs for me to buy
6. Never thought of it
7. Others ______ (**EA009_1**)

EB001_W4 Are you the policyholder/primary beneficiary of any of the types of health insurance listed below BEFORE? (circle all that apply) （ ）

1. Urban employee medical insurance (yi-bao) （ ）
2. Urban and rural resident medical insurance (integrated urban resident medical insurance and new rural cooperative medical insurance) （ ）
3. Urban resident medical insurance
4. New rural cooperative medical insurance (he-zuo-yi-liao) （ ）
5. Government medical insurance
6. Medical aid
7. Private medical Insurance: Purchased by employer : 8. Private medical Insurance: Purchased by Individual : 9. Urban non-employed personss health insurance 10. Long-term care insurance
11. Other medical insurance please specify _______ (**EB001_W4_1**) 12. None of the above

PROCEDURE

If EB001_W4 = 1 – 11, ask EB003 and EB004_W4 EB001_W4 1-11 EB003 EB004_W4

EB003 When did you quit this insurance?

______ 1900...2013 (**EB003_1**) Year ______ 0...12 (**EB003_2**) Month

[IWER: Mark the year using four digits. Take down the month as its actual number. For example, write January as 1 not 01, December as 12. If do not remember month, fill 0 : 4 1 101 ,12 120]

EB004_W4 Why did you quit this insurance? [ ]

1. Employer no longer exists
2. Insurance no longer provided locally
3. I resigned/ was fired from the employer /
4. I didnt want to participate in
5. My family did not want me to participate in
6. Premium was too expensive
7. Other please specify ______ (**EB004_W4_1**)

PART II Health Care Costs and Utilization

[IWER: Please do not allow proxy to answer Part II]

PROCEDURE

For XRType = NEWIW, answer EC001 EC001

For XRType = REIW, answer EC001_W4 EC001_W4

EC001 When did you take the last physical examination? (Not including CHARLS physical examination) （: CHARLS ）

1. ______ 1900...2018 (**EC001_1**) Year ______ 0...12 (**EC001_2**) Month 2. Have never take physical examination yet

[IWER: Mark the year using four digits. Take down the month as its actual number. For example, write January as 1 not 01, December as 12. If do not remember month, fill 0 : 4 1 101 ,12 120]

EC001_W4 When did you take the last physical examination since [ZIWTIME]? (Not including CHARLS physical examination) [] : CHARLS

1. ______ 1900...2018 (**EC001_W4_1**) Year ______ 0...12 (**EC001_W4_2**) Month

2. Have never take any other physical examination since last survey

[IWER: Mark the year using four digits. Take down the month as its actual number. For example, write January as 1 not 01, December as 12. If do not remember month, fill 0 : 4 1 101 ,12 120]

EC001_W3_1 Which item do you take in this physical examination?

1. Physical examination
2. Routine blood test
3. Routine urine test
4. Liver function test
5. Kidney function test
6. Lipids profile test
7. Blood glucose test
8. Surgical
9. Internal medicine
10. Five sense organ test
11. Electrocardiogram
12. B-type ultrasonic B
13. Chest fluoroscopy
14. Male or female specialist
15. Other , please specify ______ (**EC001_W3_1_1**)

[INTRO: The next questions pertain to medical facilities or medical providers you may have visited for outpatient care during the past 1 month (excluding hospitalization)]

ED001 In the last month have you visited a public hospital, private hospital, public health center, clinic, or health workers or doctors practice, or been visited by a health worker or doctor for outpatient care? (Not including physical examination) ()

1. Yes
2. No → Skip to EE003 EE003

ED004_W4 Which types of medical facilities have you visited in the last 4 weeks for outpatient treatment? (circle all that apply) ()

1. General hospital (Not including traditional chinese medicine hospital) ( )
2. Specialized hospital (Not including traditional chinese medicine hospital) ()

3. Chinese medicine hospital
4. Community healthcare center
5. Township hospital
6. Health care post
7. Village clinic/Private clinic /
8. Nursing home
9. Other

PROCEDURE
For each item 1-8 checked in ED004_W4, ask ED005 ED004_W4 1-8 ED005

ED005 How many times did you visit/been visited by [preload ED004_W4 answer] during the last month? [ED004_W4] ______ Times

PROCEDURE
If sum(ED005) > 1, then ask ED005_W4 and ED006_W4; otherwise, skip to ED008_W4
ED005 > 1, ED005_W4 ED006_W4 ED008_W4

ED005_W4 Did you visit the [preload ED004_W4 answer] medical facitiliy for the same disease during the last month? [ED004_W4]

1. Yes
2. No

ED006_W4 How much did all the visits to [preload ED004_W4 answer] spent during the last month? (Include self-paid part and reimbursement part) [ED004_W4] ? ( )

[IWER: If possible, please check the list of hospital chargescost]

1. Total cost ______ (**ED006_W4_1**) Yuan
   [Soft Check: upper bound: 30,000 30,000]
2. Dont know
   [Brackets: 50/100/200/500/1000]

ED007 Out-of-pocket part

1. Out-of-pocket part ? ______ (**ED007_1**) Yuan
   [Soft Check: ED007_1 ≤ ED006_W4_1, else remind Self-paid part cannot be more than total cost]
2. Didnt pay anything
3. Dont know
   [Brackets: 50/100/200/500/1000]

[INTRO: Now Id like to ask you some questions about your most recent visit to a health care provider in the last month]

ED008_W4 Which health care provider did you visit most recently during the past month?

1. Preload the health care providers in ED004_W4 ED004_W4

ED009 Is this facility public or private?

1. Public
2. Private

**PROCEDURE**
If ED008_W4 = 1 – 3, ask ED010 ED008_W4 = 1 – 3 ED010

ED010 Whats the level of this facility?

1. County/district //
2. Regional/city /
3. Provincial/ affiliated to a ministry /
4. Military
5. Others please specify ______ (**ED010_1**)
6. Not applicable

ED012 Did the provider visit you at home?

1. Yes → kip to ED023_W4 ED023_W4
2. No

ED013 How many kilometers is it from the medical facility to your residence? ______ Km

[Soft Check: upper limit : 3000]

ED017 What was the purpose of your visit? (circle all that apply) ()

1. Immunization
2. Consultation
3. Medical check-up
4. Treatment of illness
5. Other

**PROCEDURE**
If ED017 = 4, then ask ED018 —ED020, else ask ED023_W4 ED017 4 ED018 —ED020 ED023_W4

ED018 Could you tell me the disease name? _______

ED019 Was the visit a first visit or a follow-up visit for the symptom?

1. First
2. Follow-up

ED020 Was the visit for ordinary outpatient service or an emergency?

1. Ordinary
2. Emergency

ED023_W4 What was the total cost of this visit (including both treatment and medication cost, which includes the purchase of prescription drugs from this medical facility or other pharmacies)? (Includes out-of-pocket part and reimbursement part) ( ) ( )

1. ______ (**ED023_W4_1**) Yuan
   [Soft Check: upper limit : 30,000]
2. Dont know
   [Brackets: 25/50/120/400/1200]

ED024 How much did you pay out of pocket, after reimbursement from insurance?

1. ______ (**ED024_1**) Yuan
   [Soft Check: upper limit: 30,000 30,000
   ED024_1 must be no more than ED023_W4_1 else Pay out of pocket cannot be more than total cost]
2. Did not pay anything
3. Dont know
   [Brackets: 15/30/100/300/1000]

ED028_W4 What insurance did you use or will you use? (circle all that apply) ( ) ( )

1-11 Load the medical insurance type filled in EA001_W4 EA001_W4 12 Reimbursed by Rs union

13 No insurance

14 Not revelent to R

ED029 Did you give any red envelopes to the doctors for this visit?

1. Yes
2. No

PROCEDURE

If ED008_W4 = 8 (nursing home), ask ED030_W4 ED008_W4 =8 ED030_W4

ED030_W4 Can you be reimbursed for the nursing and rehabilitation cost in the nursing home?

1. Nursing expenses can be reimbursed
2. Rehabilitation expenses can be reimbursed
3. Both nursing and rehabilitation expenses can be reimbursed
4. None can be reimbursed

[INTRO: The following questions pertain to hospitalization (inpatient care) that you have had during the past year]

EE003 Have you received inpatient care in the past year? 1. Yes

2. No → Skip to EF001_W4 EF001_W4

EE004 How many times have you received inpatient care during the past year? ______ Times

**PROCEDURE**
If EE004 = 1, skip to PROCEDURE beforeEE007 EE004 = 1 EE007

EE004_W4 Did you receive the several inpatient care for the same reason?

1. Yes
2. No

EE005_W4 What was the total medical cost for all the inpatient care you received during the past year? (Include out-of-pocket part and reimbursement part. Onlyinclude fees paid to the hospital, including ward fees but excluding wages paid to a hired nurse, transportation costs, and accommodation costs for yourself or family members) ?

1. Total cost ______ (**EE005_W4_1**) Yuan
   [Soft Check: upper limit : 300,000]
2. Dont know
   [Brackets: 1500/3000/7000/15000/30000]

EE006 Out-of-pocket part

1. Out-of-pock part ______ Yuan
   [Soft Check: EE006_1 ≤ EE005_W4_1else Out-of-pocket part cannot be more than total cost]
2. Didnt pay anything
3. Dont know
   [Brackets: 600/1500/4000/8000/18000]

[INTRO: We want details about the last hospitalization you had in the past year]

PROCEDURE
If ED001 = 1 ask EE007 ED001 = 1 EE007

EE007 Is this the same facility as mentioned inED008_W4 for outpatient care?
[ED008_W4]

1. Yes → Skip to EE016 EE016
2. No

PROCEDURE
If EE007 = 2 or ED001 = 2 ask EE008_W4 - EE013 EE007 = 2 ED001 = 2 EE008_W4 - EE013

EE008_W4 What is the type of health service facility which you visited for last inpatient care (hospital admissions) for your most recent hospitalization in the past year?

1. General hospital (Not including traditional chinese medicine hospital) （ ）
2. Specialized hospital (Not including traditional chinese medicine hospital) （）
3. Chinese Medicine Hospital
4. Community Healthcare Center
5. Township Hospital
6. Health care post
7. Nursing home
8. Other

EE009 Is this facility public or private?

1. Public
2. Private

PROCEDURE
If EE008_W4 = 1 – 3, ask EE010 EE008_W4 = 1 – 3 EE010

EE010 Whats the administrative level of this facility?

1. County/district //
2. Regional/city /
3. Provincial/ affiliated to a ministry /
4. Military
5. Others

EE012_W4 What is the location of this facility?

1. This county []
2. Other // ______ (**EE012_W4_1**)

EE013 How many kilometers is it from the medical facility to your residence? ______ Km
[Soft Check: upper limit : 3000]

EE016 How many nights were you hospitalized there? ______ Nights
[Soft Check: upper limit : 40]

EE017 What was the starting date of your hospital stay?
______ 1900........... 2018 (**EE017_1**) Year ______ 0...12 (**EE017_2**) Month ______ 0...31 (**EE017_3**) Day

[IWER: Mark the year using four digits. Take down the month as its actual number. For example, write January as 1 not 01, December as 12. If do not remember month and day, fill 0 : 4 1 1 0112 120]

EE018 What was your date of exit?

1. ______ 1900...2018 (**EE018_1**) Year ______ 0...12 (**EE018_2**) Month ______ 0...31 (**EE018_3**) Day
2. Still there

[Soft Check: Date of exit should be not before starting date, exit date is before starting date, please ask R again, also exit date should be within 1 year of today, else exit date is one year ago, please verify the revision]

EE019 Why were you hospitalized?

1. Sickness
2. Accident → Skip to PROCEDURE before EE024_W4 EE024_W4 3. Violence → Skip to PROCEDURE before EE024_W4 EE024_W4
4. Other → Skip to PROCEDURE before EE024_W4 EE024_W4

EE020 Could you tell me the name of the disease? _______

EE024_W4 What was the total medical cost of hospitalization? (Only include the fees paid to the hospital, excluding the wage of hired nurse, the fare or rent, but including the ward fees) （ ）

1. ______ (**EE024_W4_1**) Yuan
   [Soft Check: upper limit : 100,000]
2. Dont know
   [Brackets: 700/1500/3500/8000/15000]

EE027 How much did you or will you eventually pay out of pocket for the total costs of hospitalization? ( )

1. ______ (**EE027_1**) Yuan
   [Soft Check: upper limit: 100,000 100,000
   EE027_1 must be no more than EE024_W4_1 else Pay out of pocket cannot be more than total cost]
2. Didnt pay anything.
3. Dont know
   [Brackets: 400/800/2000/5000/10000]

EE031_W4 What kind of medical insurance did you use or will you use to reimburse your costs? (circle all that apply) ( )

1-11 Load the medical insurance type filled in EA001_W4 EA001_W4 12 Reimbursed by Employer

13 No insurance

14 Not revelent to R

EE032 Did you pay any Red Envelopes to the doctors for this visit?

1. Yes
2. No

**PROCEDURE**
If EE008_W4 =7, ask EE033_W4 EE008_W4 =7 EE033_W4]

EE033_W4 Can you be reimbursed for the cost of nursing and rehabilitation at the nursing home?

1. Nursing expenses can be reimbursed
2. Rehabilitation expenses can be reimbursed
3. Both nursing and rehabilitation expenses can be reimbursed
4. None can be reimbursed

[INTRO: Now wed like to know whether you have treated yourself during the past month]

EF001_W4 Did you take any purchased medicine during the past month? (Not including prescription medications) ( )

[IWER: Taking any medicine delivered by others or stored by oneself is also counted]

1. Yes
2. No → Skip to EH001_W4 EH001_W4

EF002_W4 What is the approximate total cost for purchased medicine during the last month? (Include out-of-pocket part and reimbursement part) ( )

1. ______ (**EF002_W4_1**) Yuan
   [Soft Check: upper limits: 2,000 2,000]
2. Dont know
   [Brackets: 10/30/100/200/300]

**PROCEDURE**
If EF002_W4 = 0, ask EH001_W4 EF002_W4 = 0 EH001_W4

EF003 How much did you pay out-of-pocket? 1. ______ (**EF003_1**) Yuan
[Soft Check: upper limits: 2,000 2,000
EF003_1 should be no more than EF002_W4_1EF003_1 EF002_W4_1]

2. Didnt pay anything.
3. Dont know
   [Brackets: 10/30/100/200/300]

EF005_W4 What insurance did you use or will you use to reimburse your costs ? (circle all that apply) ( )

1-11 Load the medical insurance type filled in EA001_W4 EA001_W4 12 Reimbursed by Rs union

13 No insurance

14 Not revelent to R

EH001_W4 Have you ever received the benefits of long-term care insurance?

1. Yes
2. No → Skip to EH005_W4 EH005_W4

EH002_W4 What services did long-term care insurance pay or partially pay for? (circle all that apply) ()

1. Basic life care, such as bathing and turning over
2. Common clinical care, such as nasal feeding and catheterization
3. Risk prevention guidance, such as fall prevention guidance
4. Functional maintenance or rehabilitation training, such as eating training, passive joint activity
5. Other please specify ______ (**EH002_W4_1**)

EH003_W4 In the past month, your total expenditure on long-term care insurance services (including leasing and purchasing corresponding equipment) was ( ) ______ (**EH003_W4_1**) Yuan of which long-term care insurance paid ______ (**EH003_W4_2**) Yuan

EH004_W4 How much does long-term care insurance help you pay for long-term care services?

1. Extremely helpful
2. Very helpful
3. Somewhat helpful
4. Somewhat unhelpful
5. Not helpful at all

PROCEDURE

If R aged 60 and above, ask EH005_W4, otherwise skip to EH007_W3 60 EH005_W4 EH007_W3

EH005_W4 Have you ever receive the following home and community care services? (circle all that apply) ( )

1. Day care centers, nursing homes, senior dining tables, etc
2. Regular physical examination
3. Onsite visits
4. Family beds
5. Community nursing
6. Health management
7. Entertainment
8. Other please specify ______ (**EH005_W4_1**)
9. None of the above → Skip to EH007_W3 EH007_W3

EH006_W4 Did you have the subsidies of home and community care services?

1. Yes
2. No
3. Dont know

EH007_W3 Are you satisfied with the quality, cost and convenience of local medical services? Choose from vary satisfied, somewhat satisfied, neutral, somewhat dissatisfied and very dissatisfied.

1. Very satisfied
2. Somewhat satisfied
3. Neutral

4. Somewhat dissatisfied
5. Very dissatisfied

EH008_W4 Have you ever receive the paid family doctor services?

1. Yes
2. No

EF006 How often did the respondent receive assistance in answering section E-Health care and insurance

[IWER: If it is answered by a proxy, please record the reaction of respondent]

1. Never → End of healthcare module
2. A few times → End of healthcare module
3. Most or all of the time → End of healthcare module
4. The section was completed by a proxy respondent（the respondent is absent）

EF007 What is your relationship to R?

[IWER: What is the proxys relationship to R? If unknown, please ask the proxy]

1. Spouse
2. Mother
3. Father
4. Mother-in-law /
5. Father-in-law /
6. Sibling
7. Brother-in-law, sister-in-law /
8. Child
9. Spouse of child
10. Grandchild
11. Other relative
12. Helper or other non-relative

EF008 What is the main reason for proxy（the respondent is absent）

1. The respondent has serious physical handicaps 2. The respondent has serious mental handicaps,

3. The respondent has rejected this interview
4. Other please specify ______ (**EF008_1**)

This page intentionally left blank

F Work and Retirement

Preloaded Variables from the Last Waves Interview:

**ZF1** = 1	Not working in the last IW
**ZF1** = 2	Never worked in the last IW
**ZF1** = 3	Working in the last IW
**ZF4** = 1	Self-employed agricultural work in the last IW
**ZF5** = 1	Only at self-employed agricultural work in the last IW
**ZF5** = 2	Had jobs other than self-employed agricultural in the last IW
**ZF7** = 1	Nonfarm employed in the last IW
**ZF7** = 2	Nonfarm self-employed in the last IW
**ZF7** = 3	Unpaid help for family business in the last IW
**ZF7** = 4	Agricultural employed in the last IW
**ZF11** = 1	Employed in the last IW
**ZF11** = 2	Self-employed nonfarm in the last IW
**ZF13** = 1	Missing work status in the last IW
**ZF14**	Name of the employer in the last IW
**ZF15**	Name of the self-employment business in the last IW
**ZF16**	Name of the family business without getting paid in the last IW
**ZF17** = 1	Retirement processed in the last IW
**ZF18** = 1	Internal retirement processed in the last IW
**ZF19** = 1	Receding processed in the last IW
**ZF20** = 1	Internal retirement processed, but not yet retirement in the last IW

ZF21 = 1 No (internal) retirement or receding processed in the last IW
//

ZF25_1 = 1 Old R had completed retirment or receding procedures in the last IW, but the processing employer/office is missing.
/

ZF25_2 = 1 Old R had completed receding procedures in the last IW, but the time is missing.

ZF25_3 = 1 Old R had completed receding procedures in the last IW, but the wage is missing.

ZF25_4 = 1 Old R had completed retirement or early retirement.
[/]

ZF25_5 = 1 Old R had completed retirement or early retirement, but the time is missing.
[/]

ZF25_6 = 1 Old R had completed retirement or early retirement, but the wage is missing.
[/]

ZF25_8 = 1 Old R had completed internal retirement.

ZF25_9 = 1 Old R had completed internal retirement, but the time is missing.

ZF25_10 = 1 Old R had completed internal retirement, but the wage prior to internal retirement is missing.

ZF25_11 = 1 Old R had completed internal retirement, but the wage at the time of internal retirement is missing.

FA Work Status

[INTRO: Now we will ask you some questions about your work and retirement. (IWER: Work is defined as activities for earning a livelihood. If the products or services created in an activity contribute to Rs livelihood, even if no income is earned, the activity should be defined as work. For example, agricultural and individual business are both considered as work) : ( )
]

FC008 Did you engage in agricultural work for at least 10 days in the past year for your own household? Agricultural work includes farming, forestry, fishing, animal production and selling agricultral products produced by your own household. 10

1. Yes
2. No

FC001 Did you work for other farmers/employers and get paid for at least ten days in the past year? 10

1. Yes
2. No

FA002_W4 Not including agricultural work, did you work for at least one hour last week in paid work, individual business or family business without getting paid?

1. Yes → Skip to FC019_W4 BRANCHPOINT FC019_W4 BRANCHPOINT 2. No

[IWER: If R has a business that involves both agricultural and non-agricultural work, please classify it as agricultural self-employed work.]

FA003 Are you currently engaged in any non-agricultural work but are on vacation, on sick or other leave, or in job training? ( )

1. Yes
2. No → Skip to FA007_W4 BRANCHPOINT FA007_W4 BRANCHPOINT

FA004 In which month and year did you start your leave or training?

______ 1900...2018 (**FA004_1**) year ______ 0...12 (**FA004_2**) month

[IWERMark a year using four digits. Record a month using the calendar number. For

example, January as 1 instead of 01 and December as 12. If R does not recall the month, fill in with 0. : 4 1 1 0112 120]

FA005 Do you expect to get back to this job at a definite time in the future or within 6 months? 6

1. Yes → Skip to FC019_W4 BRANCHPOINT FC019_W4 BRANCHPOINT 2. No

FA006 Do you still receive any salaries or incomes from this work? ?

1. Yes → Skip to FC019_W4 BRANCHPOINT FC019_W4 BRANCHPOINT 2. No

FA007_W4 BRANCHPOINT :

- If (FC001 = 1), skip to FC019_W4 BRANCHPOINT. (FC001 = 1) FC019_W4 BRANCHPOINT
- If (FC001 = 2) & (FC008 = 1 | ZF1 = 1 | ZF1 = 3),Skip to FA010_W4_1 BRANCHPOINT. (FC001 = 2) & (FC008 = 1 | ZF1 = 1 | ZF1 = 3) FA010_W4_1 BRANCHPOINT
- Otherwise, Skip to FA007. FA007

FA007 Have you ever worked for at least three months during your lifetime? Work includes agricultural work, paid work, self-employed business, and unpaid help for family business. ( )

1. Yes → Skip to FA010_W4_1 BRANCHPOINT FA010_W4_1 BRANCHPOINT 2. No

FA008 Work includes all kinds of labor activities other than housework, whether you earn a wage or not. Are you sure that you never worked for at least three months during your lifetime?

1. Yes, never worked before
2. No, ever worked. → Skip to FA010_W4_1 BRANCHPOINT FA010_W4_1 BRANCHPOINT

FA009 What is the main reason for you not to work in your lifetime?

1. Disability (physical or psychological) 2. Housekeeping
3. No need to work because of family affluence

4. Taking care of siblings
5. Other, please specify ______ (**FA009_1**)

PROCEDURE :
Skip to FA010_W4_1 BRANCHPOINT FA010_W4_1 BRANCHPOINT

FC019_W4 BRANCHPOINT:

- Engaged in agricultural employed, agricultural self-employed, and non-agricultural work (FC008 = 1 & FC001 = 1 & (FA002_W4 = 1 | FA005 = 1 | FA006 = 1)),Skip to FC020_W4_a (FC008 = 1 & FC001 = 1 & (FA002_W4 = 1 | FA005 = 1 | FA006 = 1)) FC020_W4_a
- Engaged in agricultural employed, agricultural self-employed, but not non-agricultural work (FC008 = 1 & FC001 = 1 & !(FA002_W4 = 1 | FA005 = 1 | FA006 = 1)), Skip to FC019_W4_b (FC008 = 1 & FC001 = 1 & !(FA002_W4 = 1 | FA005 = 1 | FA006 = 1)) FC019_W4_b
- Engaged in agricultural self-employed, non-agricultural work, but not agricultural employed (FC008 = 1 & FC001 = 2 & (FA002_W4 = 1 | FA005 = 1 | FA006 = 1)), Skip to FC019_W4_c (FC008 = 1 & FC001 = 2 & (FA002_W4 = 1 | FA005 = 1 | FA006 = 1)) FC019_W4_c
- Engaged in agricultural employed, non-agricultural work, but not agricultural self-employed (FC008 = 2 & FC001 = 1 & (FA002_W4 = 1 | FA005 = 1 | FA006 = 1)), Skip to FC020_W4_d (FC008 = 2 & FC001 = 1 & (FA002_W4 = 1 | FA005 = 1 | FA006 = 1)) FC020_W4_d
- Engaged in agricultural employed, but not agricultural self-employed and non-agricultural work (FC008 = 2 & FC001 = 1 & !(FA002_W4 = 1 | FA005 = 1 | FA006 = 1)), Skip to FC019_W4_e (FC008 = 2 & FC001 = 1 & !(FA002_W4 = 1 | FA005 = 1 | FA006 = 1)) FC019_W4_e
- Engaged in non-agricultural work, but not agricultural self-employed and agricultural employed (FC008 = 2 & FC001 = 2 & (FA002_W4 = 1 | FA005 = 1 | FA006 = 1)), Skip to FC019_W4_f (FC008 = 2 & FC001 = 2 & (FA002_W4 = 1 | FA005 = 1 | FA006 = 1)) FC019_W4_f

[INTRO: We will now ask about your main job. Your main job is the one you currently spend most of the time at work. Short-term jobs of the same type are considered as one job :]

FC020_W4_a Except agricultural work for your household, what is your main job? A job can be paid agricultural work or non-agricultural work. Is this job paid agricultural work, paid non-agricultral work, individual business, or unpaid help for family business?

[IWER: Short-term jobs are temporary jobs for one employer or a variety of employers, for example, working for different agricultural households. The name of employers can be coded as not the same household or not the same firm. The workplace can be coded as the location of most employers]

1. Non- agricultural employed
2. Non-agricultural self-employed
3. Unpaid help for family business
4. Agricultural employed

PROCEDURE :
Skip to FA010_W4_1 BRANCHPOINT FA010_W4_1 BRANCHPOINT

[INTRO: We will only ask about your main job. The main job refers to the one you currently spend most of the time at work. Short-term jobs of the same type are considered as one job :
]

FC019_W4_b Except agricultural work for your household, are you currently engeged in more than one paid agricultral job?

1. Yes, at least two jobs
2. No, only one job

PROCEDURE :
Skip to FA010_W4_1 BRANCHPOINT FA010_W4_1 BRANCHPOINT

FC019_W4_c Short-term jobs of the same type are considered as one job. Except agricultural work for your household, are you currently engeged in more than one non-agricultural job?

1. Yes, at least two jobs → Skip to FC020_W4_c FC020_W4_c 2. No, only one job → Skip to FC021_W4_c FC021_W4_c

[IWER: Short-term jobs are temporary jobs for one employer or a variety of employers, for example, working for different agricultural households. The name of employers can be coded as not the same household or not the same enterprise. The workplace can be coded as the general location of Rs employers]

[INTRO: We will only ask about your main job. The main job refers to the one you currently spend most of the time at work. Short-term jobs of the same type are considered as one job :
]

FC020_W4_c Except agricultural work for your household, what is your main job? Is this job paid work, individual business, or unpaid help for family business?

1. Non-agricultural employed
2. Non-agricultural self-employed
3. Unpaid help for family business

FC021_W4_c How do you describe your current job? Do you earn a wage or do you run your own business or do unpaid work for family business?

1. Non-agricultural employed
2. Non-agricultural self-employed
3. Unpaid help for family business

PROCEDURE :
Skip to FA010_W4_1 BRANCHPOINT FA010_W4_1 BRANCHPOINT

[INTRO: We will only ask about your main job. The main job refers to the one you currently spend most of the time at work. Short-term jobs of the same type are considered as one job :
]

FC020_W4_d What is your current main job? A job can be paid agricultural work or non-agricultural work. Is this job paid agricultural work, paid non-agricultral work, individual business, or unpaid help for family business?

[IWER: Short-term jobs are temporary jobs for one employer or a variety of employers, for example, working for different agricultural households. The name of employers can be coded as not the same household or not the same enterprise. The workplace can be coded as the general location of Rs employers]

1. Non-agricultural employed
2. Non-agricultural self-employed
3. Unpaid help for family business
4. Agricultural employed

PROCEDURE :
Skip to FA010_W4_1 BRANCHPOINT FA010_W4_1 BRANCHPOINT

[INTRO: We will only ask about your main job. The main job refers to the one you currently spend most of the time at work. Short-term jobs of the same type are considered as one job :
]

FC019_W4_e Are you currently engeged in more than one paid agricultural job?

1. Yes, at least two jobs
2. No, only one job

PROCEDURE :
Skip to FA010_W4_1 BRANCHPOINT FA010_W4_1 BRANCHPOINT

FC019_W4_f Short-term jobs of the same type are considered as one job. Are you currently engeged in more than one non-agricultural job?

1. Yes, at least two jobs → Skip to FC020_W4_f FC020_W4_f 2. No, only one job → Skip to FC021_W4_f FC021_W4_f

[IWER: Short-term jobs are temporary jobs for one employer or a variety of employers, for example, working for different agricultural households. The name of employers can be coded as not the same household or not the same enterprise. The workplace can be coded as the general location of Rs employers]

[INTRO: We will only ask about your main job. The main job refers to the one you currently spend most of the time at work. Short-term jobs of the same type are considered as one job :
]

FC020_W4_f What is your main non-agricultural job? Is this job paid work, individual business or unpaid help for family business?

1. Non- agricultural employed
2. Non-agricultural self-employed
3. Unpaid help for family business

FC021_W4_f How do you describe your current job? Do you earn a wage or do you run your own business or do unpaid work for family business?

1. Non-agricultural employed
2. Non-agricultural self-employed
3. Unpaid help for family business

FA010_W4_1 BRANCHPOINT:
Generate variables of work status

- XF1 = 1 if currently not working (FC008 = 2 & FC001 = 2 & (FA003 = 2 | FA006 = 2) & !(FA008 = 1))
- XF1 = 2 if never works (FA008 = 1)
- XF1 = 3 if working (FC008 = 1 | FC001 = 1 | FA002_W4 = 1 | FA005 = 1 | FA006 = 1)
- XF4 = 1 if self-employed agricultural (FC008 = 1)
- XF5 = 1 if self-employed agricultural is the only job (FC008 = 1 & ! (FC001 = 1 | FA002_W4 = 1 | FA005 = 1 | FA006 = 1))
- XF5 = 2 if work other than self-employed agricultural (FC001 = 1 | FA002_W4 = 1 | FA005 = 1 | FA006 = 1)

Generate variables of main job types

- XF7 = 1 if nonfarm employed (FC020_W4_a = 1 | FC020_W4_c = 1 | FC020_W4_d = 1 | FC020_W4_f = 1 | FC021_W4_c = 1 | FC021_W4_f = 1)
- XF7 = 2 if nonfarm self-employed (FC020_W4_a = 2 | FC020_W4_c = 2 | FC020_W4_d = 2 | FC020_W4_f = 2 | FC021_W4_c = 2 | FC021_W4_f = 2)
- XF7 = 3 if unpaid help for family business (FC020_W4_a = 3 | FC020_W4_c = 3 | FC020_W4_d = 3 | FC020_W4_f = 3 | FC021_W4_c = 3 | FC021_W4_f = 3)
- XF7=4 if agricultural employed (FC020_W4_a = 4 | FC020_W4_d = 4 | (FC019_W4_b = 1 | FC019_W4_b = 2 | FC019_W4_e = 1 | FC019_W4_e = 2))
- XF11 = 1 if employed (XF7 = 1 | XF7 = 4)
- XF11 = 2 if self-employed nonfarm (XF7 = 2 | XF7 = 3)

BRANCHPOINT

- For old R missing work status in the last IW (XRTYPE = REIW & ZF13 = 1),Skip to FA006_W3_1 FA006_W3_1
- For new R (XRTYPE = NEWIW), Skip to FA_END_W4 BRANCHPOINT FA_END_W4 BRANCHPOINT
- Otherwise Skip toFA010_W4_2 BRANCHPOINT FA010_W4_2 BRANCHPOINT

Update Missing Work Status in the Last Visit

[INTRO: Due to incomplete answers on your work information in the last visit, we will ask you some questions about your job at that time :]

FA006_W3_1 Did you engage in agricultural work (including farming, forestry, fishing, and animal production for your own household or others) in the last IW? [ZIWTime]

1. Yes
2. No → Skip to FA006_W3_3 FA006_W3_3

FA006_W3_2 What agricultural work did you engage in the last IW? (Select all that apply) ( )

1. Household agricultural work
2. Agricultural employed /

FA006_W3_3 Did you engage in non-agricultural work in the last IW? We consider any of the following activities to be at work: earn a wage, run your own business and unpaid work for family business. Work does not include doing your own housework or doing activities without pay, such as volunteer work. [ZIWTime]

1. Yes
2. No → Skip FA006_W3_4 FA006_W3_4

FA006_W3_4 What non-agricultural work did you do in the last IW?

1. Employed
2. Self-employed
3. Unpaid help for family business

PROCEDURE : Update preloaded work variables from the last IW **XZF**

- XZF1 = 1 if then not working (FA006_W3_1 = 2 &FA006_W3_3 = 2)
- XZF1 = 2 if never works ()
- XZF1 = 3 if working (FA006_W3_1 = 1 | FA006_W3_3 = 1)
- XZF4 = 1 if self-employed agricultural (FA006_W3_2s1 = 1)
- XZF5 = 1 if self-employed agricultural is the only job (FA006_W3_2s1 = 1 & !(FA006_W3_2s2 = 1) & FA006_W3_3 = 2)
- XZF5 = 2 if work other than self-employed agricultural (FA006_W3_2s2 = 2 | FA006_W3_3 = 1)
- XZF7 = 1 if nonfarm employed (FA006_W3_4 = 1)
- XZF7 = 2 if nonfarm self-employed (FA006_W3_4 = 2)
- XZF7 = 3 if unpaid help for family business (FA006_W3_4 = 3)
- XZF7 = 4 if agricultural employed (FA006_W3_2s2 = 2)
- XZF11 = 1 if employed (FA006_W3_2s2 = 2 | FA006_W3_4 = 1)
- XZF11 = 2 if self-employed nonfarm (FA006_W3_4 = 2 | FA006_W3_4 = 3)
- XZF13 = 0 work status in the last IW updated

Job Switch

FA010_W4_2 BRANCHPOINT:

- Not working → Working → (XZF1 = 1 & XF1 = 3),Skip toFA010_W4_1 FA010_W4_1
- Working → Not working → (XZF1 = 3 & XF1 = 1),Skip toFA011_W4_1 FA011_W4_1
- Self-employed agricultural only → Other jobs → (XZF5 = 1 & XF5 = 2), Skip to FA012_W4_1 FA012_W4_1
- Other jobs → Self-employed agricultural only → (XZF5 = 2 & XF5 = 1),Skip to FA013_W4_1 FA013_W4_1
- Employed → Nonfarm self-employed → (XZF11 = 1 & XF11 = 2), Skip to FA014_W4_1 FA014_W4_1
- Nonfarm self-employed → Employed → (XZF11 = 2 & XF11 = 1), Skip to FA015_W4_1 FA015_W4_1
- Otherwise , Skip to FA_END_W4 BRANCHPOINT FA_END_W4 BRANCH-POINT

FA010_W4_1 In the last IW you told us you did not work, so between the last IW and now, when did you start working? [ZIWTime]

1. ______ (**FA010_W4_1_1**) year ______ (**FA010_W4_1_2**) month
2. I was working → Skip to FA006_W3_1 FA006_W3_1

[Hard Check: FA010_W4_1_1 ≥ ZIWTime
IWER: Job switch happened later than the last IW. If the respondent can not remember the time, please code as −1 −1]

FA010_W4_3 What was the reason for you to start to work? 1. Economic reasons, need more income
2. Bad health status in the past
3. Caring family members in the past
4. Personal development in the past, e.g. short leave, study, go abroad, traveling etc.

5. Others, please specifiy ______ (**FA010_W4_3_1**)

FA010_W4_4 Please describe your first job after you started to work recently. ( )
1. Non-agricultral employoed
2. Non-agricultral self-employed
3. Unpaid nonfarm family business
4. Agricultral employed
5. Agricultral self-employed

**PROCEDURE** :
Skip to FA_END_W4 BRANCHPOINT FA_END_W4 BRANCHPOINT

FA011_W4_1 In the last IW you told us you were working. Work refers to agricultural work, paid work, individual business, unpaid help for family business. When did you end that work? [ZIWTime]
1. ______ (**FA011_W4_1_1**) year ______ (**FA011_W4_1_2**) month → Skip to FA011_W4_2 2. I did not work at that time → Update variable XZF1 = 1
[Hard Check: FA011_W4_1_1 ≥ ZIWTime
IWER: Job switch happened later than the last IW. If the respondent can not remember the time, please code as −1. −1]

FA011_W4_1_1 When did you end your last job? /
______ (**FA011_W4_1_1_1**) year ______ (**FA011_W4_1_1_2**) month
[IWER: If the respondent can not remember the time, please code as −1 −1]

FA011_W4_2 Please describe your last job. ( )

1. Non-agricultral employoed
2. Non-agricultral self-employed
3. Unpaid nonfarm family business
4. Agricultral employed
5. Agricultral self-employed

FA011_W4_3 What was the reason for you to stop doing this job? 1. Forced to leave, e.g. being fired

2. Health reason
3. Caring for family members
4. Retirement
5. Others, please specifiy ______ (**FA011_W4_3_1**)

FA011_W4_4 Upon leaving your last job, did you receive any severance package other than retirement allowance? (For example,lump-sum payment, condolence payment, etc.)

1. Yes
2. No → Skip to FA_END_W4 BRANCHPOINT FA_END_W4 BRANCHPOINT

[IWER: Thelump-sum payment refers to the onetime payment that state-owned companies offered to its workers upon terminating contract with them in the 1980s and 1990s, when the companies were going through privatization and laying off redundant employees. The specific amount of the payment would be bargained by both sides based on the previous position, wage level, etc. of the employee, as well as the situation of the company]

FA011_W4_5 What is the severance pay? ? _______ (**FA011_W4_5_1**) Yuan ; The pay is based on what? / ______ (**FA011_W4_5_2**) For lump-sum pay- ment, what was the length of your service used for its calculation? ______ 0...120 (**FA011_W4_5_3**) Year

FA011_W4_bracket [IWER: If R is unwilling to answer or does not remember, ask unfolding bracket questions here 0] 1000 /2000 /5000 /10,000 /20,000 Yuan

PROCEDURE :

Skip to FA_END_W4 BRANCHPOINT FA_END_W4 BRANCHPOINT

FA012_W4_1 In the last IW you told us your work is household agricultural work. Between that time and now, when did you start your other new job(s)? [ZIWTime]

1. ______ (**FA012_W4_1_1**) year ______ (**FA012_W4_1_2**) month
2. I did not do household agricultural work at that time. → Skip to FA006_W3_1 FA006_W3_1
   [Hard Check: FA012_W4_1_1 ≥ ZIWTime
   IWER: Job switch happened later than the last IW. If the respondent can not remember the time, please code as −1 −1]

FA012_W4_2 Not including household agricultural work, when did you start your current main job? ( )

______ (**FA012_W4_2_1**) year ______ (**FA012_W4_2_2**) month

[Hard Check: FA012_W4_2_1 ≥ FA012_W4_1_1

IWER: The start time of your current/most recent job should not be earlier than the job switch time. If the respondent can not remember the time, please code as −1 −1]

FA012_W4_3 What is the reason for switching from household agricultural work only to other work?

1. Forced to switch, e.g. having lost farmland 2. Switch job for higher income
3. Health reason
4. Caring for family members
5. Moving, e.g. leaving rural villages
6. Others, please specifiy ______ (**FA012_W4_3_1**)

PROCEDURE :
Skip to FA_END_W4 BRANCHPOINT FA_END_W4 BRANCHPOINT

FA013_W4_1 In the last IW you told us you were working, besides doing household agricultural work. Between that time and now, when did you end other work? [ZIWTime]

1. ______ (**FA013_W4_1_1**) year ______ (**FA013_W4_1_2**) month
2. I had no other job then. Skip toFA006_W3_1FA006_W3_1 [Hard Check: FA013_W4_1_1 ≥ ZIWTime
   IWER: Job switch happened later than the last IW. If the respondent can not remember the time, please code as −1 −1]

FA013_W4_2 When did you start doing household agricultural work only?

______ (**FA013_W4_2_1**) year ______ (**FA013_W4_2_2**) month

[Hard Check: FA013_W4_2_1 ≥ FA013_W4_1_1

IWER: The starting time of doing household agricultural work only should not be earlier than the job switching time. If the respondent can not remember the time, please code as −1 −1]

FA013_W4_3 What is your reason for switching from having other jobs to household agricultural work only?

1. Forced to leave the last job, e.g. being fired
2. Switch job for higher income
3. Health reason
4. Caring for family members
5. Moving, e.g. leaving rural villages
6. Retirment
7. Others, please specifiy ______ (**FA013_W4_3_1**)

PROCEDURE :
Skip to FA_END_W4 BRANCHPOINT FA_END_W4 BRANCHPOINT

FA014_W4_1 In the last IW you told us you worked largely for some other employers. Between that time and now, when did you end this kind of employed work? [ZIWTime]

1. ______ (**FA014_W4_1_1**) year ______ (**FA014_W4_1_2**) month
2. I didnt work for some other employers at that time. → Skip to FA006_W3_1 FA006_W3_1
   [Hard Check: FA014_W4_1_1 ≥ ZIWTime
   IWER: Job switch happened later than the last IW. If the respondent can not remember the time, please code as −1 −1]

FA014_W4_2 When did you start working largely with your current business, or for family business without pay? /

______ (**FA014_W4_2_1**) year ______ (**FA014_W4_2_2**) month

[Hard Check: FA014_W4_2_1 ≥ FA014_W4_1_1

IWER: Individual/helper work should not start earlier than the job switching time. If the respondent can not remember the time, please code as −1 / −1]

FA014_W4_3 What is your reason for switching from wage earning jobs to individual business or family business unpaid work? （） //

1. Inherit business or get business from relatives ( )
2. Forced to leave, e.g. being fired
3. Higher income in self-employed business, or due to family business reasons
4. Health reasons
5. Caring for family members
6. Retirement
7. Others, please specify ______ (**FA014_W4_3_1**)

PROCEDURE:

If FA014_W4_3 ≠ 1, Skip to FA_END_W4 BRANCHPOINT FA014_W4_3 ≠ 1 FA_END_W4 BRANCHPOINT

FA014_W4_4 From which relative did you get the individual business? /

1. Parents
2. Parents in law
3. Spouse
4. Siblings
5. Children (preload names of children) ([]) (**FA014_W4_4_1**)
6. Relatives, please specify the relationship ______ (**FA014_W4_4_2**)

PROCEDURE:

Skip to FA_END_W4 BRANCHPOINT FA_END_W4 BRANCHPOINT

FA015_W4_1 In the last IW you told us you worked largely for individual business or family business unpaid work. Between that time and now, when did you end that job? [ZIW Time] //

1. ______ (**FA015_W4_1_1**) year ______ (**FA015_W4_1_2**) month
2. I did not have a non-farm self-employed job then. Skip to FA006_W3_1 FA006_W3_1

   [Hard Check: FA015_W4_1_1 ≥ ZIWTime

   IWER: Job switch happened later than the last IW. If the respondent can not remember the time, please code as −1 −1]

FA015_W4_2 When did you start your current paid work?

______ (**FA015_W4_2_1**) year ______ (**FA015_W4_2_2**) month

[Hard Check: FA015_W4_2_1 ≥ FA015_W4_1_1

IWER: The start time of this employed job should not be earlier than the job switching time. If the respondent can not remember the time, please code as −1 −1]

FA015_W4_3 What is the reason for switching from individual business or unpaid work for family business to this wage earning employed job? / /

1. Inherited by children or transfered to relatives （ ）
2. Forced to leave, e.g. being fired 3. Higher income
4. Health reasons
5. Caring for family members
6. Family or economic conflicts
7. Others, please specify ______ (**FA015_W4_3_1**)

**PROCEDURE** : If FA015_W4_3 ≠ 1, Skip to FA_END_W4 BRANCHPOINT FA015_W4_3 ≠ 1 FA_END_W4 BRANCHPOINT

FA015_W4_4 Which relative did you transfer the business to? /

1. Parents
2. Parents in law
3. Spouse
4. Siblings
5. Children (preload names of children) （ [] ）__ (**FA015_W4_4_1**)
6. Spouse of children (preload names of children) （ [ ] ） ______ (**FA015_W4_4_2**)
7. Grandchildren (preload names of children who are the transferees parents) （ [] ） ______ (**FA015_W4_4_3**)
8. Relatives, please specify the relationship ______ (**FA015_W4_4_4**)

FA_END_W4 BRANCHPOINT :

- Never worked (XF1 = 1 | XF1 = 2),Skip to FK002 FK002
- Agricultral self-employed (XF4 = 1),Skip to FC022_W4 FC022_W4
- Employed but not agricultral self-employed (XF11 = 1 & ! XF4 = 1), Skip to FD001 FD001
- Non-farm self-employed but not agricultral self-employed (otherwise) Skip to FH001 FH001

FC Self-Employed Agricultural Work

[INTRO: Now we will ask you some questions about your work on household agricultural work in the past year]

FC022_W4 Which type of agricultural work are you engaged in? e.g. growing crops, growing fruits and raising animals. _______

FC023_W4 What kind of work do you mainly do?

1. Manual labor
2. Management
3. Machine operation
4. Others, pleas specify ______ (**FC023_W4_1**)

FC009 How many months did you work on [farming, forestry, animal production, and fishing] for your own household in the past year? [ ] [] [ ]______ 0...12 months

FC010 How many days did you work for your own household per week on average during a normal month in the past year? [ ] [] [] ______ 0...7 days

FC011 How many hours did you usually work for your own household during a normal work day in the past year? [ ] [] [] ______ 0...24 Hours

[Soft Check: FC011 > 16 is unreasonable]

FC012 Where is your workplace most of the time? [preload sampling community ID]

1. Same as the permanent address / [BB001_W3]
2. Other village/community in the permanent addresss county/city/district BB001_W3 ///
   ______ (**FC012_1**) township //
   ______ (**FC012_2**) village/community /
3. Other
   ______ (**FC012_3**) province/city/county/district //
   ______ (**FC012_4**) township/village/community /////

[IWER: Fill in with others if unknown to R]

FC012_W3 How satisfied are you with your job? 1. Completely satisfied

2. Very satisfied
3. Some what satisfied
4. Not very satisfied
5. Not at all satisfied

FC013 How many days of work did you miss in the past year due to health problems? _____ 0...366 days

[IWER: Mark 0 ifR didnt miss any work days 0]

FC024_W4 Do you employ other people in your household agricultural work? How many people are employed? ______ 0...9999

PROCEDURE :

- If no other job (XF5 = 1), Skip to FM_BEGINNING_W4 BRANCHPOINT FM_BEGINNING_W4 BRANCHPOINT
- If employed (XF11 = 1),Skip to FD001 FD001
- If nonfarm self-employed (XF11 = 2),Skip to FH001 FH001

FD Employed

[INTRO: Now we will ask you some information about that wage earning job. We need to confirm whether you are a dispatched worker by a company or some individual]

FD001 Do you receive wages from your current workplace, or from a dispatch/contract company, or some individual? ( )

1. Place of work
2. Labor dispatch company
3. Individual

[IWER: Workplace is where labor activities take place. Dispatch company is where workers establish work contracts. Individual refers to a boss of small business or a farm household ( )]

[CAPI: For dispatched/contract workers (FD001 = 2), note: the next few questions pertain to the situation at your current workplace, but not to the company that dispatched/contracted you out; for those hired by individuals (FD001 = 3), note: the next few questions pertain to the situation at your current workplace, but not to the individual that paid you wages

(FD001 = 2) ( ) (FD001 = 3) ( ) ( )]

FD002 What is the type of your workplace/employer? / ( )

1. Government
2. Public institution
3. NGO
4. Firm
5. Individual firm
6. Farmer
7. Individual household
8. Other ______ (**FD002_1**)

FD003 What is the name of your workplace/employer? Please state specifically the name of your company or institution. / ( )

Name of the workplace ______ (**FD003_1**) (1 2)

Name of the department ______ (**FD003_2**) (1 2)

[IWER: Write down the name of the household head ifR works for a family]

**PROCEDURE** :
• If REIW Rs employer in the last IW not missing (XZF14 ≠ null), Skip to FD003_W4_1 FD003_W4_1<br>• If REIW R was employed and the employer in the last IW is missing (XZF11 = 1 & XZF14 = null),Skip to FD003_W4_2 FD003_W4_2<br>• Otherwise NEWIW REIW Skip toFD004 FD004

FD003_W4_1 For the interviewer, check whether the names of the workplace at the last wave and this wave are the same. **[FD003_1] [XZF14]**

1. Yes → Skip to FD004 FD004
2. No

FD003_W4_2 [If XZF14 ≠ null: in the last interview [ZIWTime] you told us the name of your workplace is XZF14, is your current workplace same as the one in the last interview [ZIW-Time]? XZF14≠ null → [ZIWTime] [XZF14]] [ZIWTime]

1. Yes → The interview determines whether the name of Rs employer should be modified in FD003_1 FD003_1then Skip to FD004 FD004
2. No

FD003_W4_3 What is the reason for your change to the current workplace/employer? /

1. Involuntary switch: business failure (including decrease in working hours), lay-off, bankruptcy, and etc. （ ）
2. For higher income
3. Health reasons
4. Voluntary switch: personal interest, relaxation, better relationship with current colleagues.
5. Family reasons: moving, childrens education
6. Retirement
7. Others, please specify ______ (**FD003_W4_3_1**)

FD004 Where is your workplace located? Where is your workplace located? / (preload sampling community ID)

1. Same as the permanent address / [BB001_W3]
2. Other village/community in the permanent addresss county/city/district BB001_W3 ///
   ______ (**FD004_1**) township //
   ______ (**FD004_2**) village/community /
3. Other
   ______ (**FD004_3**) province/city/county/district //
   ______ (**FD004_4**) township/village/community /////
4. Abroad

[IWER: Fill in with others if unknown to R]

PROCEDURE

If R is working for an individual farmer or resident household (FD002 = 6, 7), Skip to FD011（FD002 = 6, 7）FD011

FD005 What industry does you workplace belong to—that is, what does your workplace make or do? / / ___ （ 1 2）

[IWER: Type of business]

PROCEDURE

- If employed by government （FD002 = 1）FD006
- If employed by a public institution （FD002 = 2）FD009
- If employed by a firm （FD002 = 4）FD010
- Otherwise （） (FD002 = 3, 5, 8） FD011

FD006 Are you a civil servant?

1. Yes
2. No

FD007 Are you a formal employee of an establishment? 1. Yes

2. No

PROCEDURE :

Skip to FD011 FD011

FD009 Are you a formal employee of an establishment? 1. Yes

2. No

PROCEDURE :

Skip to FD011 FD011

FD010 What is the ownership type of your workplace/employer? / 1. 100% State owned firm

2. State-controlled firm
3. 100% Collective-owned firm
4. Collective-controlled firm
5. 100% Private firm /
6. Private-controlled firm
7. 100% foreign-owned
8. Joint venture
9. Other joint-ownership
10. Other ______ (**FD010_1**)

FD011 When did you start working for this employer? / / ______ 1900...2018 (**FD011_1**) Year _ 0...12 (**FD011_2**) Month

[IWERMark a year using four digits. Record a month using the calendar number. For example, January as 1 instead of 01 and December as 12. If R does not recall the month, fill in with 0 : 4 1 1 01, 12 120]

[Soft Check: Consistency of employment start date. Make Sure that the individual was at least a minimum age when he/she started working for this employer (e.g., prompt to check if under 16). Specifically (FD011_1+FD011_2/12)-(CV009_a+CV009_b/12)<16 prompts a soft check]

FD012 What sort of work do you do? ______ (**FD012_1**) （1 2） Do you have any professional certificate? ______ (**FD012_2**)（1 2）

[IWER: Ask about the specific work that R does]

FD013 What is your current position?

1. Clerk/worker （）
2. Team Leader
3. Section Chief
4. Director of a division
5. Director- General of a bureau and above
6. Village Leader
7. Township Leader
8. Division manager
9. Overall/ General manager
10. Other, ______ (**FD013_1**)

FD014 What is your current professional/technical level? / /

1. Technician
2. Primary level
3. Intermediate level
4. Advanced level
5. No professional/ technical level

[IWER: A professional/technical level is referred to an occupation rank evaluated professionally /]

FD015 Are you in a position to supervise others?

1. Yes
2. No → Skip to FD020 FD020

FD016 How many people are there under your supervision?

1. 1~5 people 1~5
2. 6~10 people 6~10
3. 11~15 people 11~15
4. 16~30 people 16~30

5. 31~99 people 31~99
6. More than 100 people 100

[CAPI: For dispatched worker (FD001 = 2), note: the questions on labor contract and social insurances are related to your dispatch company. For those hired by individuals (FD001 = 3), note: the questions on labor contract and social insurances are related to the person who paid you wages. (FD001 = 2) (FD001 = 3) ( )]

FD020 Did you receive a labor contract (or employment contract) in written form from your current workplace (or labor dispatch company)? /
1. Yes
2. No → Skip to FD024 FD024

FD021 What is the agreed period of employment (labor contract period)?
1. Defined period ______ 0...100 (**FD021_1**) Years ______ 0...11 (**FD021_2**) months
   [IWER: If R does not recall the months, please fill in with 0 0]
2. Not defined → Skip to FD024 FD024
3. Same as the term of the project

FD022 Has the current employment contract ever been renewed?
1. Yes
2. No → Skip to FD024 FD024

FD023 How many times has the contract been renewed? ______ 1...50 times

FD024 How long do you expect to work at your current workplace? ( )
1. Less than one year 1
2. One to two years 1-2
3. Two to three years 2-3 → Skip FD025 FD025
4. More than three years 3 → Skip FD025 FD025

FD025 Why do you expect so?
1. Because the pre-defined contract period will soon expire
2. Because the verbally agreed contract period will soon expire (although theres no written contract)

3. Because I expect no contract renewal will be agreed upon 4. Because the current job/project will be completed /
5. Because the person I am substituting/ replacing will return
6. Because I can only work during certain seasons
7. Because I want to look for a job that better suits my job aptitude, abilities, and preferences
8. Because I will reach the retirement age as set by regulations/practice
9. Because of family care responsibilities, poor health, etc. 10. Other

FD029 Except for national/public holidays, how many days of paid vacation do you have this year at your current workplace? _ 0…366 days

[IWER: Mark 0 if there is no paid vacation 0] [Soft Check for a reasonable range: If FD029 > 30, prompt for verification]

FD030 How many days of work did you miss at this current job in the past year due to health problems? / ______ 0…366 days

[IWER: Mark 0 if the R didntmiss work days 0]

**PROCEDURE** :
If FD030 = 0, skip FD031 FD030 = 0 FD031

FD031 In these days, how many are fully paid? _______

0…366 days

[Soft Check for reasonable range: If FD031 > FD030, prompt for verification]

FG003_W4 Does your employer provide pension insurance, health insurance, unemployment insurance, worker sinjury insurance, maternity insurance, and housing provident fund (Select all that apply) ( )

1. Pension insurance
2. Health insurance
3. Unemployment insurance
4. Workers injury insurance
5. Maternity insurance
6. Housing provident fund
7. None → Skip to FD034_W4 FD034_W4

FG010_W4 On what income base is the contribution to the above-mentioned social insurance determined? ( ) ( )

1. ______ Yuan/month / (**FG010_W4_1**)
2. 60% of local average income 60% ( ) 997. Dont know

FD034_W4 Does your employer purchase supplementary health insurance for you?

1. Yes
2. No

[IWER: Supplementary health insurance is for out-patient use, which is different from the accident insurance : **]**

FD032_W3 Does your employer purchase workplace accident insurance for you?

1. Yes
2. No → Skip to FE001 FE001

FD033_W3 How much does the insurance pay at the most if you encounter a severe accident at your workplace (e.g. death) ? ? ______ Yuan

FE Questions About Labor Supply

[CAPI: For dispatched/contract workers (FD001 = 2), note for FE001: the next few questions on labor supply pertain to the situation at your current workplace, but not to the company that dispatched/contracted you out; for those hired by individuals (FD001 = 3), note for FE001: the next few questions on labor supply pertain to the situation at your current workplace, but not to the individual that paid you wages (FD001 = 2) FE001 (FD001 = 3)FE001 ( ) ( )]

FE001 Including paid vacations and sick leave fully paid, how many months did you work in the past year? ______ 1...12 months

[IWER: Working month is at least 1. Each month needs to be considered if R ever worked]

FE002 How many days a week did you work on average in the past year? ______ 0...7 days

[IWER: If Rs working days are not regular, working days in a week can be imputed by total working days in a year divided by working months and then dividied by four 4]

FE003 How many hours did you work per day on average in the past year, excluding meal breaks but including any paid or unpaid overtime? ____ 0...24 Hours

[Soft Check: Verify if number of hours per day is unreasonable, e.g., FE003 > 16]

FF Questions About Wages

[CAPI: For dispatched/contract workers (FD001 = 2), note for FF001 - FG002: the next few questions on wages are related to your dispatch company; for those hired by individuals (FD001 = 3), note for FF001 - FG002: the next few questions on wages are related to the person who paid you wages. Benefits from the workplace should not be included (FD001 = 2) FF001 - FG002 (FD001 = 3)FF001 - FG002 ( )]

FF001 How is your wage paid mainly? Is it regularly paid, contract-based, performance-based, or other? If it is regularly paid, please tell me how often you receive your wages. Do you have a yearly contract, monthly, weekly, daily, or hourly? Please select one.

1. Yearly wages
2. Monthly wages → Skip to FF004_W4 FF004_W4
3. Weekly wages → Skip to FF006_W4 FF006_W4
4. Daily wages → Skip to FF008 FF008
5. Hourly wages → Skip to FF010 FF010
6. Contract-based → Skip to FF012_W4 FF012_W4
7. Performance-based → Skip to FF012_W4 FF012_W4
8. Other ______ (**FF001_W4_1**) → Skip to FF012_W4 FF012_W4

FF002_W4 What is your salary including bonuses in the past year? / _____ (**FF002_W4_a**) Yuan Bonuses ______ (**FF002_W4_b**) Yuan

[Soft Check: Prompt for clarification if under a low threshold, e.g. under 1200 Yuan annual. Specifically, prompt to clarify if FF002_W4_a < 1200.]

[Hard Check: FF002_W4_a ≥ FF002_W4_b]

FF002_W4_bracket [IWER: If R is unwilling to answer or does not remember, ask unfolding bracket questions here 0] What is your salary from that workplace in the past year, including all bonuses? 10,000 /30,000 /50,000 /100,000 /200,000 Yuan

FF002_W4_1 Are personal income tax, social insurance contributions, housing fund contributions and other fees already deducted from this salary?

1. Yes
2. No

997. Dont know

FF002_W4_2 What is the total amount of these personal income tax, social insurance contributions, housing fund contributions and other fees? ______ (**FF002_W4_2_1**) Yuan

**PROCEDURE** :<br>Skip to FF014 FF014

FF004_W4 What is your salary including bonuses last month? / ______ (**FF004_W4_a**) Yuan Bonuses _____ (**FF004_W4_b**) Yuan

[Soft Check: Prompt for clarification if under a low threshold, e.g. under 100 Yuan per month. Specifically, prompt to clarify if FF004_W4_a < 100]

[Hard Check: FF004_W4_a ≥ FF004_W4_b]

FF004_W4_bracket [IWER: If R is unwilling to answer or does not remember, ask unfolding bracket questions here 0] What is your monthly salary from that workplace, including all bonuses? 500 /1,000 /2,500 /5,000 /10,000 Yuan

FF004_W4_1 Are personal income tax, social insurance contributions, housing fund contributions and other fees already deducted from this salary?

1. Yes

2. No
997. Dont know

FF004_W4_2 What is the total amount of these personal income tax, social insurance contributions, housing fund contributions and other fees? ______(**FF004_W4_2_1**) Yuan

PROCEDURE :
Skip to FF014 FF014

FF006_W4 What is your salary including bonuses last week? / _____ Yuan

[Soft Check: Prompt for clarification if under a low threshold, e.g. under 25 Yuan per week. Specifically, prompt to clarify if FF006_W4 < 25]

FF006_W4_bracket [IWER: If R is unwilling to answer or does not remember, ask unfolding bracket questions here 0] 20 /50 /100 /200 /500 Yuan

PROCEDURE :
Skip to FF012_W4 FF012_W4

FF008 In general, what is your daily wage? ______ Yuan

[Soft Check: Prompt for clarification if under a low threshold, e.g. under 5 Yuan per day. Specifically, prompt to clarify if FF008 < 5]

FF009 [IWER: If R is unwilling to answer or does not remember, ask unfolding bracket questions here 0] 20 /50 /100 /200 /500 Yuan

PROCEDURE :
Skip to FF012_W4 FF012_W4

FF010 What is your hourly wage? ______ Yuan

[Soft Check: Prompt for clarification if under a low threshold, e.g. under 1 Yuan per hour. Specifically, prompt to clarify if FF010 < 1]

FF010_bracket [IWER: IfR is unwilling to answer or does not remember, ask unfolding bracket questions here 0]
10 /30 /50 /100 /200 Yuan

FF012_W4 How much were you paid including bonuses last month from this job? , ______ (**FF012_W4_a**) Yuan Bonuses _____ (**FF012_W4_b**) Yuan

[Soft Check: Prompt for clarification if under a low threshold, e.g. under 100 Yuan per month. Specifically, prompt to clarify if FF012_W4 < 100]

[Hard Check: FF012_W4_a ≥ FF012_W4_b]

FF012_W4_bracket [IWER: If R is unwilling to answer or does not remember, ask unfolding bracket questions here 0] 500 /1,000 /2,500 /5,000 /10,000 Yuan

FF012_W4_1 Are personal income tax, social insurance contributions, housing fund contributions and other fees already deducted from this salary?

1. Yes
2. No

997. Dont know

FF012_W4_2 What is the total amount of these personal income tax, social insurance contributions, housing fund contributions and other fees? ______ (**FF002_W4_2_1**) Yuan

FF014 What is the amount of all your other bonuses (not paid regularly as your wages) received in the past year? / ___ Yuan

[Soft Check: Prompt on bonuses and monthly earnings if bonuses is more than five times monthly net income]

FG Fringe Benefits

FG001 Please answer yes if any of the following benefits are provided by your current workplace/employer. (Select all that apply) / ()

1. Free lunch
2. Free breakfast
3. Free dinner
4. Meal allowance
5. Transportation allowance

6. Free housing
7. Housing allowance
8. Company car
9. Company shuttle bus
10. Other allowance, please specify ______ (**FG001_1**) 11. None

PROCEDURE :
For each checked answer of FG001, ask FG002 FG002

FG002 What is the monetary value of each allowance you receive per month? ______ Yuan

PROCEDURE :
Skip to FJ_BEGINNING_W4 BRANCHPOINT FJ_BEGINNING_W4 BRANCHPOINT

FH Non-Farm Self-Employed and Unpaid Work for Family Business

[INTRO: Now we will ask you some questions about your individual business or helper job //]

FH001 How many months did you work in the past year? ______ 0...12 months

FH002 How many days did you work per week on average in the past year? ______ 0...7 days

FH003 How many hours did you work per day on average in the past year, excluding meal breaks but including any paid or unpaid overtime on a normal work month? _ 0...24 hours

[Soft Check: Verify if number of hours per day is unreasonable, e.g., FH003 > 16]

FH004 How many days of work did you miss in the past year due to health problems? _____ 0...366 days

[IWER: Mark 0 if you didnt miss any work days 0]

PROCEDURE :
If R is a family business helper (XF7 = 3), Skip toFH012 (XF7 = 3) FH012

FH005 What is the name of your company or workplace? ( ) ______ (1 2)

[IWER: If there is more than one company, ask about the main one. Mark 0 if there is no name 0]

PROCEDURE :

- If REIW Rs business in the last IW not missing, / (XZF15 ≠ null),Skip to FH005_W4_1 FH005_W4_1
- If REIW R was self-employed and the business in the last IW is missing / (XZF7=2 & XZF15 = null),Skip to FH005_W4_2 FH005_W4_2
- Otherwise NEWIW REIW / (else),Skip to FH006 FH006

FH005_W4_1 For the interviewer, check whether the names of the businesses at the last wave and this wave are the same. **[FH005] [XZF15]**

1. Yes → Skip to FH006 FH006
2. No

FH005_W4_2 [If XZF15 ≠ null: in the last interview [ZIWTime] you told us the name of your business is XZF15, is your current business same as the one in the last interview [ZIW-Time]? XZF15≠ null → [ZIWTime] [XZF15]] [ZIWTime]

1. Yes → The interview determines whether the name of Rs business should be modified in FH005FH005then Skip to FH006 FH006
2. No

FH005_W4_3 What is the reason for the business change? ( )

1. For higher income
2. Unanticipated business failure
3. External problems, e.g. government restrictions
4. Health reasons
5. Personal interest
6. Housing or location considerations
7. Others, please specify ______ (**FH005_W4_3_1**)

FH006 Where is your company or workplace located? /

1. Same as the permanent address / [BB001_W3]
2. Other village/community in the permanent addresss county/city/district BB001_W3 ///
   ______ (**FH006_1**) township //
   ______ (**FH006_2**) village/community /
3. Other
   ______ (**FH006_3**) province/city/county/district //
   ______ (**FH006_4**) township/village/community /////
4. Abroad

[IWER: Fill in with others if unknown to R]

FH007 What industry does your business beong to? that is, what does your business do or make? ______ （1 2）

[IWER: Type of business]

FH008 When did you start working at the current company or workplace? / ______ 1900...2018 (**FH008_1**) Year ______ 0...12 (**FH008_2**) Month

[IWER: Mark the year using four digits. Take down the month as its actual number. For example, write January as 1 not 01, December as 12. If do not remember month, fill in with 0 : 4 1 1 01, 12 120]

[A check similar to the one for FD011. (FD011_1 + FD011_2 /12)- (CV009_a + CV009_b/12) < 16 or (FD011_1 + FD011_2 /12)- (CV009_a + CV009_b/12) < FB001_1 or FH008_1 < FB001_2]

FH009_W4 How many people are employed in your company/ workplace? / ______ 0...9999

**PROCEDURE** :<br>Skip to FJ_BEGINNING_W4 BRANCHPOINT FJ_BEGINNING_W4 BRANCHPOINT

FH012 What is the name of company or workplace that you help without getting paid? _____ （1 2）

[IWER:Mark 0 if there is no name 0]

PROCEDURE :

- If REIW Rs helping business in the last IW not missing (XZF16 ≠ null),Skip to FH012_W4_1 FH012_W4_1
- If REIW R help a family business without getting paid and the helping business in the last IW is missing (XZF7=3 & XZF16 = null), Skip to FH012_W4_2 FH012_W4_2
- Otherwise NEWIW REIW , Skip toFH013 FH013

FH012_W4_1 For the interviewer, check whether the names of the businesses at the last wave and this wave are the same. **[FH012]** **[XZF16]**

1. Yes → Skip to FH013 FH013
2. No

FH012_W4_2 [If XZF16 ≠ null: in the last interview [ZIWTime] you told us the name of your helping business is XZF16, is your current helping business same as the one in the last interview [ZIWTime] XZF16 ≠ null → [ZIWTime] [XZF16]] [ZIWTime]

1. Yes → The interview determines whether the name of Rs employed should be modified in FH012FH012then Skip to FH013 FH013
2. No

FH012_W4_3 ( )

1. For higher income
2. Unanticipated business failure
3. External problems, e.g. government restrictions
4. Health reasons
5. Personal interest
6. Housing or location considerations
7. Others, please specify ______ (**FH012_W4_3_1**)

FH013 Where is this company or workplace loacted? /

1. Same as the permanent address / [BB001_W3]
2. Other village/community in the permanent addresss county/city/district BB001_W3 ///
   ______ (**FH013_1**) township //
   ______ (**FH013_2**) village/community /

3. Other
______ (**FH013_3**) province/city/county/district //
______ (**FH013_4**) township/village/community ///// 4. Abroad
[IWER: Fill in with others if unknown to R]

FH014 What industry does you helping business belong to–—that is, what does this company do or make? ______ （1 2）
[IWER: Type of business]

FH015 What sort of work did you do? ______ （1 2 ）
[IWER: Ask the specific work]

FH018 When did you start working at the current company or workplace? / ______
1900...2018 (**FH018_1**) Year ______ 0...12 (**FH018_2**) Month

FJ Side Job (Employed or Self-employed) （ ）

FJ_BEGINNING_W4 BRANCHPOINT:
IfR doesnt have a side job (FC019_W4_b = 2 | FC019_W4_c = 2 | FC019_W4_e = 2 | FC019_W4_f = 2)Skip to FM_BEGINNING_W4 BRANCHPOINT FM_BEGINNING_W4 BRANCHPOINT

FJ001_W4 How many jobs do you currently engage in, excluding your main job and household agricultral work? （） ______ 1...20
[IWER: We know R has a few jobs from the beginning of this section. These jobs may include agricultrual employed and non-agricultrual employed. If R asserts having no more side job at this point, check and correct FC008, FC001, FA002_W4, FC019_W4 FC008FC001 FA002_W4FC019_W4 （）]

FJ002_W4 How many hours a week do you work on average at your side job(s),not considering your main job? （） ______ 0...168 Hours per week /

FJ003_W4 What is the average monthly income or wage that you get from side job(s) other than your main job? （） ______ Yuan per month /

FJ003_bracket [IWER: IfR is unwilling to answer or does not remember, ask unfolding bracket questions here] 500 /1,000 /2,500 /5,000 /10,000 Yuan

**PROCEDURE** :<br>Skip to FM_BEGINNING_W4 BRANCHPOINT FM_BEGINNING_W4 BRANCHPOINT

FK Unemployment and Job Search Activities

FK002 Did you search for a new job during the past month? 1. Yes
2. No

FM054 Are you currently engaged in any pastime activitity and still get some incomes from doing it?
1. Yes
2. No → Skip to FK_END_W4 BRANCHPOINT FK_END_W4 BRANCHPOINT

[IWER: Pastime work refers to an activity not for the purpose of earnings, and also not for helping family business either]

FM055 What kind of pastime work are you engaged in? ? ______
[IWER: Ask the specific work]

FM056 When did you start this work? ______ 1900...2018 (**FM056_1**) year ______ 0...12 (**FM056_2**) month

[IWERMark the year using four digits. Take down the month as its actual number. For example, write January as 1 not 01, December as 12. If do not remember month, fill 0: 4 1 101 12 120]

FM057 How many days per week do you usually work for your pastime job? An average of 0...7 days per week

FM058 How many hours per week do you usually work at your pastime job? An average of 0.00...168.00 hours per week

FM059 What is your monthly income from the pastime work? ______ Yuan

[IWER: Mark 0 if there is no net income, and mark 999997 if running a deficit 0 999997]

FM059_bracket [IWER: IfR is unwilling to answer or does not remember, ask unfolding bracket questions here 0]

500 /1,000 /2,000 /3,500 /5,000 Yuan

FK_END_W4 BRANCHPOINT:

- If new R is working (XRTYPE = NEWIW & XF1 = 1)Skip to FL001 FL001
- If old R ever worked (XRTYPE = REIW & XF1 = 1)Skip to FM_BEGINNING_W4 BRANCHPOINT FM_BEGINNING_W4 BRANCHPOINT
- IfR never works (XF1 = 2)Skip to FN002_W4 FN002_W4

FL Last Job

[INTROThe next questions are about the last main job you had, which could be agricultural, earning a wage, running your own business or working for unpaid help for family business. It does not include doing your own housework or doing activities without pay, such as voluntary work. If you have more than one job, we are interested in the job at which you spent most of the time. Were also more interested in your situation near the termination of this job]

FL001 Were you engaged in paid work for someone else (including unpaid work for family business), self-employed business, or household agricultural work?

1. Employed
2. Self-employed 3. Unpaid help for family business
4. Self-employed agricultural

FL002 In which year and month did you start this job? ______ 1900...2018 (**FL002_1**) Year ______ 0...12 (**FL002_2**) Month

[IWER: Mark the year using four digits. Take down the month as its actual number. For example, write January as 1 not 01, December as 12. If do not remember month, fill in with 0 : 4 1 1

01, 12 120]

[Soft Check: Prompt for Verification/Clarification if the Respondent was Less than 16 at time of starting this job, e.g. Prompt if (FL002_1 + FL002_2 /12) - (CV009_a + CV009_b/12) < 16, Replace 16 in the above check with FB001. FL002_1 < FB001_2 or FL002_1 < (CV009_a + FB001_1)]

FL003 In which year and month did you stop this job? ______ 1900...2018 (**FL003_1**) Year ____ 0...12 (**FL003_2**) Month

[IWER: Mark the year using four digits. Take down the month as its actual number. For example, write January as 1 not 01, December as 12. If do not remember month, fill in with 0 : 4 1 1 01, 12 120]

[Soft Check: Prompt for Verification/clarification if End Date is Before the Start Date, e.g. FL003_1 < FL002_1]

FL004 Where was the job located?

1. Same as the permanent address / [BB001_W3]
2. Other village/community in the permanent addresss county/city/district BB001_W3 ///
   ______ (**FL004_1**) township //
   ______ (**FL004_2**) village/community /
3. Other
   ______ (**FL004_3**) province/city/county/district //
   ______ (**FL004_4**) township/village/community /////
4. Abroad

[IWER: Fill in with others if unknown to R]

PROCEDURE :

For agricultral work (FL001 = 4),Skip to FL004_W4_1 FL001 = 4 FL004_W4_1 For non-agricultural work (FL001 = 1, 2, 3), ask FL005 to FL008 （FL001 = 1/2/3）FL005 FL008

FL004_W4_1 Which type of agricultural work are you engaged in? e.g. growing crops, growing fruits and raising animals. ______

FL004_W4_2 What kind of work do you mainly do?

1. Manual labor
2. Management
3. Machine operation
4. Others, pleas specify ______ (**FL004_W4_2_1**)

PROCEDURE :
If FL001 = 4, Skip to FL020_W4 FL001 = 4 FL020_W4

FL005 What was the name of your workplace/employer? Please state specifically the name of your company or business. () Name of the workplace ______ **(FL005_1)** （ 1 2 ） , Name of the department ______ **(FL005_2)** （ 1 2）

FL006 What industry did the workplace belong to, that is, what did the workplace make or do? （ 1 2）
[IWER: Type of business]

FL007 Is this workplace still in business?
1. Yes
2. No

FL008 In general, how many hours per week did you work? ______ 0...168 Hours per week /
[Soft Check: Prompt for Verification if FL008 > 80]

PROCEDURE :
- If FL001 = 1 Skip to FL009 (FL001 = 1) FL009
- If FL001 = 2 Skip toFL012_W4 / (FL001 = 2) FL012_W4
- If FL001 = 3 Skip toFL020_W4 (FL001 = 3) FL020_W4

FL012_W4 In general, how many people did the business employ? / ______ 0...9999

PROCEDURE :
Skip to FL020_W4 FL020_W4

FL009 What were your monthly wages, bonuses, and allowance from this job, before you stopped this job? ______ Yuan
[IWER: Mark 0 if there is no net income, and mark 999997 if running a deficit 0 999997]
[Soft Check: Prompt for Verification if FL009 < 100]

FL009_bracket [IWER: IfR is unwilling to answer or does not remember, ask unfolding bracket questions here 0]
500 /1,000 /2,500 /5,000 /10,000 Yuan

FL011 What was theyearly amount of other bonuses not paid regularly as wages? ______ Yuan

FL011_bracket [IWER: IfR is unwilling to answer or does not remember, ask unfolding bracket questions here 0]
500 /1,000 /2,500 /5,000 /10,000 Yuan

FL013 Were you a formal/contract/temporary employee or a hourly paid worker?
1. Formal employee
2. Contract employee
3. Temporary employee
4. Hourly paid worker

FL014 Did you work for the government, public institution, firm, NGO, individual farmer or a resident household? / ( )
1. Government
2. Public institution → Skip to FL017 FL017
3. NGO → Skip to FL017 FL017 4. Firm Skip to → FL016 FL016
5. Individual firm → Skip to FL017 FL017
6. Individual farmer → Skip to FL017 FL017
7. Individual household → Skip to FL017 FL017
8. Other ______ (**FL014_1**) → Skip to FL017 FL017

FL015 Were you a civil servant?
1. Yes
2. No

**PROCEDURE** : Skip to FL017 FL017

FL016 What was the ownership type of the business?
1. 100% State owned firm
2. State-controlled firm
3. 100% Collective-owned firm
4. Collective-controlled firm
5. 100% Private firm /
6. Private-controlled firm
7. 100% foreign-owned
8. Joint venture
9. Other joint-ownership

10. Other, please specify ______ (**FL016_1**)

FL017 What sort of work did you do? ______ (**FL017_1**) (1 2) Do you have any professional certificate? ______ (**FL017_2**) (1 2)
[IWER: Ask about the specific work]

FL020_W4 Why did you stop working?
1. Forced to leave 2. Health reasons
3. Caring for family members
4. Retirement
5. Others, please specify ______ (**FL020_W4_1**)

FL021 Upon leaving your last job, did you receive any severance package other than retirement allowance? (For example,lump-sum payment, condolence payment, etc.)
1. Yes
2. No → FM_BEGINNING_W4 BRANCHPOINT
[IWER: Thelump-sum payment refers to the onetime payment that state-owned companies offered to its workers upon terminating contract with them in the 1980s and 1990s, when the companies were going through privatization and laying off redundant employees. The specific amount of the payment would be bargained by both sides based on the previous position, wage level, etc. of the employee, as well as the situation of the company]

FL022_W4 What is the severance pay? ? ______ (**FL022_W4_1**) Yuan The pay is based on what? / ______ (**FL022_W4_2**) If you received a lump-sum payment, what was the length of your service used for its calculation? ______ 0...120 (**FL022_W4_3**) Year

FL022_bracket [IWER: IfR is unwilling to answer or does not remember, ask unfolding bracket questions here 0]
1000 /2000 /5000 /10,000 /20,000 Yuan

FM Retirement

FM_BEGINNING_W4 BRANCHPOINT:

If (XF5 = 2)Skip to FK000_W3_1 (XF5 = 2) FK000_W3_1 If ((XF1 = 1 &FK002 = 1) | XF5 = 1),Skip toFK000_W3_2 ((XF1 = 1 & FK002 = 1) | XF5 = 1) FK000_W3_2

If (XF1 = 1 & FK002 = 2),Skip toFM000_W4 BRANCHPOINT (XF1 = 1 &FK002 = 2) FM000_W4 BRANCHPOINT

FK000_W3_1 How satisfied are you with your job? ( ) 1. Completely satisfied

2. Very satisfied
3. Some what satisfied
4. Not very satisfied
5. Not at all satisfied

FK000_W3_2 At what age do you plan to stop working,i.e. stop earning incomes or working for family business without pay or engaging in any other work more serious than pasttime work? ?______ 1...120 Years old

[IWER: pastime refers to any work that is not mainly for earning incomes or working for family business, please ask for an approximation. 0 if plan to keep working as long as being physically capable 0]

FM000_W4 BRANCHPOINT :

If (XRTYPE = NEWIW | XZF21 = 1),Skip to FB011_W4 + // (XRTYPE = NEWIW | XZF21 = 1) FB011_W4

FM000_W4 Last time you told us that [preload ZIWTime] you had already processed [preload one of the following types: normal retirement/ internal retirement/ receding]. Is this record correct? (Retirement refers to the pension-guaranteed retirement from government, public institutions, and enterprises, and also eligible by individuals from the informal sector who have contributed to the elemental social pension insurance. Having started to receive pensions from the Urban Residence Insurance, the New Agricultural Insurance, and the Insurance for Urban and Rural Residents is not considered as retirement) [ZIWTime] [XZF17 = 1][XZF18 = 1][XZF19 = 1] ( )

1. Yes

2. No → [If having retired XZF17 = 1, update variables XZF17 = 0XZF21 = 1][If having processed internal retirement XZF18 = 1, update variables XZF18 = 0XZF20 = 0XZF21 = 1][If having processed receding XZF19 = 1, update variables XZF19 = 0XZF21 = 1]

FM001_W4_1 BRANCHPOINT:

1. Update missing information of old Rs retirement
   - If in the last wave XZF17 = 1, check XZF25_1 XZF25_5 XZF25_6; if XZF25_1 = 1, ask FM004, if XZF25_5 = 1, ask FM014, if XZF25_6 = 1, ask FM016. INTRO: You informed us in the last wave that you have completed the retirement procedures, and we would like to confirm some answers. [/] （XZF17 = 1）XZF25_1XZF25_5XZF25_6 XZF25_1 = 1 FM004 XZF25_5 = 1 FM014 XZF25_6 = 1 FM016
   - If in the last wave XZF18 = 1, check XZF25_1, XZF25_9, XZF25_10, XZF25_11; if XZF25_1 = 1, ask FM004, if XZF25_9 = 1, ask FM025, if XZF25_10 = 1, ask FM027, if XZF25_11 = 1, ask FM028. INTRO: You informed us in the last wave that you have completed the internal retirement procedures, and we would like to confirm some answers. （XZF18 = 1）XZF25_1 XZF25_9XZF25_10XZF25_11 XZF25_1 = 1 FM004 XZF25_9 = 1 FM025 XZF25_10 = 1 FM027 XZF25_11 = 1 FM028
   - If in the last wave XZF19 = 1, check XZF25_1, XZF25_2, XZF25_3; if XZF25_1 = 1, ask FM004, if XZF25_2 = 1, ask FM005, if XZF25_3 = 1, ask FM007. INTRO: You informed us in the last wave that you have completed the receding process, and we would like to confirm some answers （XZF19 = 1） XZF25_1XZF25_2XZF25_3 XZF25_1 = 1 FM004 XZF25_2 = 1 FM005 XZF25_3 = 1 FM007
2. PROCEDURE
   - If (XZF20 = 1),Skip toFM037_W2 (XZF20 = 1) FM037_W2
   - If (XZF21 = 1), Skip to FB011_W4 // (XZF21 = 1) FB011_W4
   - Others Skip to FN002_W4 FN002_W4

FB011_W4 Have you completed retirement procedures（including early retirement）or internal retirement（Note: Retirement refers to the pension-guaranteed retirement from government, public institutions, and enterprises, and also eligible by individuals from the informal sector who have contributed to the elemental social pension insurance. Having started to receive pensions from the Urban Residence Insurance, the New Agricultural Insurance, and the Insurance for Urban and Rural Residents is not considered as retirement.）?

1. Yes → Skip to FM001 FM001
2. No

FB012 Have you completed the receding procedures 1. Yes

2. No → Skip to FN002_W4 FN002_W4

[IWER:receding refers to the case where the employee is not eligible to retire in terms of age or length of work years, but has lost the ability to work due to illness or disabilities and hence needs to withdraw from his/her previous position. According to the law the employee shall receive certain amount of compensation in this scenario]

FM001 Which of the following is the employer/office that processed your [preload: retirement /receding] [/]

[IWER: If R has no employer (no answer to FD003 or FL005),please choose (3) None of the above (FD003 &FL005)（3）]

1. Current employer [FD003] → Skip to FM005 FM005
2. Last employer [FL005
   XZF14] → Skip to FM005 FM005
3. None of the above

FM002 What is the name of the employer that processed your [preload: retirement /receding]? [/] ______

FM003 What was the type of your employer [preload: retirement /receding]? [/]

1. Government
2. Public institution
3. NGO
4. Firm
5. Individual firm
6. Farmer
7. Individual household
8. Other

FM004 Where is this employer located? [/]

1. Same as the permanent address / [BB001_W3]

2. Other village/community in the permanent addresss county/city/district BB001_W3 ///
   ______ (**FM004_1**) township //
   ______ (**FM004_2**) village/community /
3. Other
   ______ (**FM004_3**) province/city/county/district //
   ______ (**FM004_4**) township/village/community /////
4. Abroad

[IWER: Fill in with others if unknown to R]

**PROCEDURE** :
If (FB011_W4 = 1),Skip toFM011 (FB011_W4 = 1) FM011

FM005 In what month and year did you recede from your position? ______ (**FM005_1**) 1900...2018 year ______ (**FM005_2**) 0...12 month

[IWERMark a year using four digits. Record a month using the calendar number. For example, January as 1 instead of 01 and December as 12. If R does not recall the month, fill in with 0 : 4 1 1 01, 12 120]

FM006 What was the main reason you receded from your position?

1. Unable to work anymore due to health condition but not eligible to retire
2. With length of eligible work years less than three, paused working more than a year due to illness or injuries unrelated to work
3. Was diagnosed with a severe chronic disease which rendered work impossible within six months of employment
4. Receded from position voluntarily
5. Reached retirement age, but length of eligible work years not long enough
6. Other

FM007 Before your receding, what was your monthly salary? _____ Yuan/month / (including basic wages, bonuses, and etc. ,)

FM007_bracket [IWER: IfR is unwilling to answer or does not remember, ask unfolding bracket questions here 0]
500 /1,000 /2,000 /3,500 /5,000 Yuan

FM008 Upon receding, did you receive any compensation? 1. Yes
2. No → Skip to FM042 FM042

FM009 How much was the compensation? ? ________ Yuan

FM009_bracket [IWER: IfR is unwilling to answer or does not remember, ask unfolding bracket questions here 0]
500 /1,000 /2,000 /3,500 /5,000 Yuan

PROCEDURE :
Skip to FM042 FM042

FM011 Was your retirement normal retirement, early retirement, or internal retirement initially, followed by normal retirement? ,

1. Normal retirement
2. Early retirement
3. Internal retirement first, then normal retirement
4. Internal retirement, but not yet normal retirement

FM012 Did you retire as a worker or as a cadre? [/] 1. Worker
2. Cadre

PROCEDURE :
If FM011 = 3 or FM011 = 4 , Skip to FM025 FM011 = 3, 4 FM025

FM014 In what month and year did you take [preload: normal/early] retirement? [/] ______ 1900...2018 (**FM014_1**) year ______ 0...12 (**FM014_2**) month

[IWERMark a year using four digits. Record a month using the calendar number. For example, January as 1 instead of 01 and December as 12. If R does not recall the month, fill in with 0 : 4 1 1 01, 12 120]

[Soft Check: Prompt for Verification/Correction if Age of Early Retirement is Young, e.g. Prompt if ((FM014_1 + FM014_2 /12) - (CV009_a + CV009_b/12) < 45 & CV004 = 2) | ((FM014_1 + FM014_1/12) - (CV009_a + CV009_b/12) < 50 & CV004 = 1)]

PROCEDURE :
Skip to FM016 if FM011 = 1 (normal retirement). FM011 = 1 FM016

FM015 What was the main reason you processed early retirement?

1. I have work experience of 30 years, which is enough for early retirement. 30
2. My work had high risk and high intensity, for which I was eligible for early retirement.
3. My employer was restructured/bankrupt, and hence I was offered early retirement
4. Due to poor health
5. Due to family reason
6. Other

FM016 Before your retirement, including bonuses and subsidies, et. al, what was your total monthly salary? ______ Yuan/month /

FM016_bracket [IWER: IfR is unwilling to answer or does not remember, ask unfolding bracket questions here 0]
500 /1,000 /2,500 /5,000 /10,000 Yuan

**PROCEDURE** :<br>Skip to FM036 FM036

FM025 In what month and year did you take internal retirement? 1900...2018 (**FM025_1**) year 0...12 (**FM025_2**) month

[IWERMark a year using four digits. Record a month using the calendar number. For example, January as 1 instead of 01 and December as 12. If R does not recall the month, fill in with 0 : 4 1 1 01, 12 120]

[Soft Check: Prompt for Verification/Correction if Age of Retirement is Young, e.g. Prompt if (FM030_1 + FM030_2/12) - (CV009_a + CV009_b/12) < 45 & CV004 = 2) | ((FM030_1 + FM030_2/12) - (CV009_a + CV009_b/12) < 50 & CV004 = 1)]

FM026 What was the main reason you processed internal retirement?

1. 5 years less than the legal retirement age 5
2. My employer was restructured/bankrupt
3. Due to poor health
4. Due to family reason
5. Other

FM027 Before your retirement, what was your total monthly salary, including basic wage, bonus, etc.? ? ______ Yuan/month /

FM027_bracket [IWER: IfR is unwilling to answer or does not remember, ask unfolding bracket questions here 0]
500 /1,000 /2,000 /3,500 /5,000 Yuan

FM028 How much was the internal retirement wage (everything included) when you processed internal retirement? /

FM028_bracket [IWER: IfR is unwilling to answer or does not remember, ask unfolding bracket questions here 0]
500 /1,000 /2,000 /3,500 /5,000 Yuan

**PROCEDURE** :
If (FM011 = 4),Skip toFM037 (FM011 = 4) FM037

FM030 In what year and month did you complete the normal retirement procedures ______
1900...2018 (**FM030_1**) year ______ 0...12 (**FM030_2**) month

[IWERMark a year using four digits. Record a month using the calendar number. For example, January as 1 instead of 01 and December as 12. If R does not recall the month, fill in with 0 : 4 1 1 01, 12 120]

[Soft Check: Prompt for Verification/Correction if Age of Retirement is Young, e.g. Prompt if ((FM030_1 + FM030_2 /12) - (CV009_a+CV009_b/12) < 45 & CV004 = 2) | ((FM030_1 + FM030_2/12) - (CV009_a + CV009_b/12) < 50 & CV004 = 1)]

FM036 How many years of eligible service or social insurance contributions did you have at the time of normal retirement? / ______ 0.00...100.00 Years

**PROCEDURE** :
Skip to FM042 FM042

FM037_W2 Have you completed normal retirement procedures? 1. Yes → Skip to FM030 FM030
2. No → Skip to FN002_W4 FN002_W4

FM037 In what month and year are you going to process normal retirement? ______
2018...2050 (**FM037_1**) year ______ 0...12 (**FM037_2**) month

[IWERMark a year using four digits. Record a month using the calendar number. For example, January as 1 instead of 01 and December as 12. If R does not recall the month, fill in with 0 : 4 1 1 01, 12 120]

[Soft Check: Verify if Age of Respondent will be outside the legal retirement range, e.g., Prompt for verification if ((FM037_1 + FM037_2/12) - (CV009_a + CV009_b/12) < 50 | (FM037_1 + FM037_2/12) - (CV009_a + CV009_b/12) > 55) & CV004 = 2) | (FM037_1 + FM037_2 /12) - (CV009_a + CV009_b/12) < 55 | (FM037_1 + FM037_2/12) - (CV009_a + CV009_b/12) > 60) & CV004 = 1)]

FM040 How many years of eligible service or social insurance contributions will you have at the time of retirement? / ______ 0.00...100.00 Years

FM041 How many years of eligible service or social insurance contributions do you currently have? / ______ 0.00...100.00 Years

[Hard Check: FM040 > FM041]

FM042 Did you have a spouse when you processed [preload: normal retirement / early retirement / internal retirement / receding]? [/// （FM011 FM037_W2 = 1）]

1. Yes
2. No

FM043 How was your health at the time of your [preload: normal retirement / early retirement / internal retirement / receding]? Was it excellent, very good, good, fair or poor? [///] ?

1. Excellent
2. Very good
3. Good
4. Fair
5. Poor

**PROCEDURE** :
Skip to FM047 if FM042 = 2 FM042 = 2 FM047

FM044 Had your spouse already processed retirement when you processed [Preload: normal retirement / early retirement / internal retirement / receding]? [/ //]

1. Yes
2. No

FM045 What kind of economic activities was your spouse engaged in at the time of your [preload: retirement / receding]? [/]

1. Employed
2. Ran own business

3. Not working, but looking for a job
4. Not working and not looking for a jobor only doing housework ,
5. Farming

FM046 How was your spouses health at the time of your [preload: normal retirement / early retirement / internal retirement / receding]? Was it excellent, very good, good, fair or poor? [///] ?

1. Excellent
2. Very good
3. Good
4. Fair
5. Poor

FM047 Was your father alive at the time of your [preload: normal retirement / early retirement / internal retirement / receding]? [///]

1. Yes
2. No → Skip to FM049 FM049

FM048 How about the health of your father at the time of your [preload: normal retirement / early retirement / internal retirement / receding]? Was it excellent, very good, good, fair or poor? [///] ?

1. Excellent
2. Very good
3. Good
4. Fair
5. Poor

FM049 Was your mother alive at the time of your [preload: normal retirement / early retirement / internal retirement / receding]? [///]

1. Yes
2. No → Skip to FM051 FM051

FM050 How about the health of your mother at the time of your [preload: normal retirement / early retirement / internal retirement / receding]? Was it excellent, very good, good, fair or poor? [///] ?

1. Excellent
2. Very good
3. Good

4. Fair
5. Poor

FM051 How many grandchildren below age 6 did you have at the time of your [preload: normal retirement / early retirement / internal retirement / receding]? [/ //] ______ 0...50 persons
[IWER: If none, fill in with 0 0]

PROCEDURE :
For a new R with a job (XRTYPE = NEIW & XF1 = 3),Skip toFM053 (XRTYPE = NEIW & XF1 = 3) FM053

FM052 Did you work after you processed [preload: regular retirement / early retirement / internal retirement / receding position], including agricultural, paid work, running your own business or working for family business, but excluding housework and volunteering. [///]

1. Yes
2. No → Skip to FN002_W4 FN002_W4

FM053 After you processed [preload: normal retirement / early retirement / internal retirement / receding], how long had you waited before you started to work again? [///] _____ 0.00...100.00 Years

This page intentionally left blank

FN Pension

[:]

[F1 (1) (2) (3) () (4) (5) (6) () (7) (8) (9) ()]

PART 1 Pension for Public Servants, Public Institution Employees, and Basic Pension for Enterprise Employees ()

FN002_W4 Do you currently receive, expect to receive, or contribute to the pension for public servants, or pension for public institution employees, or basic pension for enterprise employees?

1. Yes I currently participate in or receive benefits
2. No I do not participate in or receive benefit → Skip to FN030_W4 FN030_W4

FN002_W4_a Which pension(s) do you currently receive, expect to receive benefits from, or contribute to? (Check all that apply) ()

1. Pension for public servants
2. Pension for public institution employees
3. Basic pension for enterprise employees
4. Unknown

PROCEDURE :

For each choice chosen in FN002_W4_a ask FN002_W4_b - FN014_W4_c in a loop FN002_W4_a FN002_W4_b - FN014_W4_c

To generate [Name of Pension] in a loop, if FN002_W4_a = 1, 2, 3, then [Name of Pension] is the corresponding choice [] FN002_W4_a = 1, 2, 3 [ ]

if FN002_W4_a = 4, then [Name of Pension] is pension for public servants, or public institution employees, or basic pension for enterprise employees FN002_W4_a = 4 []

FN002_W4_b Do you currently receive benefits from [Name of Pension]? [ ]

1. Yes I do
2. No I havent started to receive any benefits → Skip to FN007_W4 FN007_W4

FN003_W2 In what month and year did you start to receive benefits from [Name of Pension]?
[]______ 1900…2018 (**FN003_W2_1**) Year ______ 0…12 (**FN003_W2_2**) Month

[IWERYear is in four digits. Month is in its actual value. For example, January is 1 not 01, December is 12. IfR does not remember the month, fill 0 [1900-2018] 4 1 101 12 120]

FN005_W2 How much pension benefits do you receive from [Name of Pension] each month (including subsidies?) [] / ()______ Yuan

[IWER: Use -1 for unknown, ask unfolding bracket questions for -1 or 0 Yuan 11 0]

[Soft Check: Verify if monthly benefits are low or high, e.g., prompt if FN005_W2 < 200 yuan/month]

FN005_W2_bracket [IWER: IfR is unwilling to answer, does not remember, or the answer is 0 Yuan, ask unfolding bracket questions 0] 1500 /2,000 /2,500 /3,000 /4,000 yuan

FN006_W4 Where do you currently receive benefits from [Name of Pension]? [ ]

1. The same as current residence BB001_W3 BB001_W3
2. County/city/district of current residence BB001_W3 BB001_W3 // ______ (**FN006_W4_1**) other townships/subdistricts // ______ (**FN006_W4_2**) villages/neighborhoods /

3. Other ______ (**FN006_W4_3**) province_city_county/city/district _ _// ____ (**FN006_W4_4**) township/subdistrict/village/neighborhood ////

997. Unknown

PROCEDURE :
Skip to FN014_W4_a FN014_W4_a

FN007_W4 Where do you currently contribute to [Name of Pension]? [ ]

1. The same as current residence BB001_W3 BB001_W3
2. County/city/district of current residence BB001_W3 BB001_W3 // ______ (**FN007_W4_1**) other townships/subdistricts // ______ (**FN007_W4_2**) villages/neighborhoods /
3. Other ______ (**FN007_W4_3**) province_city_county/city/district _ _// ______ (**FN007_W4_4**) township/subdistrict/village/neighborhood //// 997. Unknown

FN008_W4 Do you participate this pension plan by yourself or through your company? []

1. By Myself
2. Through my employer → Skip to FN012_W2 FN012_W2

FN104_W4 Do you receive 4050 subsidies? 4050

[IWER: The 4050 group refers to unemployed females over 40 years old, unemployed males over 50, and the severely disabled of the same age. Because of no work, they need government support. Thus, the government implements a series of social protection policies, including social insurance subsidies that target the 4050 group 4050 40 50 4050]

1. Yes
2. No → Skip to FN012_W2 FN012_W2

997. Unknown → Skip to FN012_W2 FN012_W2

FN105_W4 In what month and year did you start to receive 4050 subsidies? 4050 ______ 1900...2018 (**FN105_W4_1**) Year ______ 0...12 (**FN105_W4_2**) Month

[IWERYear is in four digits. Month is in its actual value. For example, January is 1 not 01, December is 12. If R does not remember the month, fill 0 : 4 1 10112 12 0]

FN106_W4 What is the share or the amount of the pension subsidy? ______ 0.00...100.00 (**FN106_W4_1**) percent Or ______ (**FN106_W4_2**) Yuan per month /
[CAPIAllow unknown casesdenote as -1 -1]

FN107_W4 What is the share or the amount of the medical insurance subsidy? ______ 0.00...100.00 (**FN107_W4_1**) percent Or ______ (**FN107_W4_2**) Yuan per month /
[CAPIAllow unknown cases, denote as -1 -1]

**PROCEDURE** :
FN104_W4 - FN107_W4 appear once in the loop FN104_W4 - FN107_W4

FN012_W2 Do you or your employer need to contribute to [Name of Pension] from which you expect to receive benefits or you currently contribute to? []

1. Yes
2. No → Skip to FN017_W2 FN017_W2

**PROCEDURE** :
If FN008_W4 = 1 and FN012_W2 = 1, then ask FN013_W2 FN008_W4 = 1 FN012_W2 = 1 FN013_W2
If FN008_W4 = 2 and FN012_W2 = 1, then ask FN013_W2- FN014_W2_bracket FN008_W4 = 2 FN012_W2 = 1 FN013_W2 - FN014_W2_bracket

FN013_W2 For [Name of Pension], how much do you currently contribute by yourself? [] _ (**FN013_W2_1**) Yuan per month / Or ______ 0.00...100.00 (**FN013_W2_2**) percent of wage
[IWER: Use -1 for unknown, ask unfolding bracket questions for -1 or 0 Yuan 11 0]

FN013_W2_bracket [IWER: If R is unwilling to answer or does not remember, ask unfolding bracket questions here]50 /200 /300 /500 /750 yuan

FN014_W2 For [Name of Pension], how much does your employer currently contribute? [] _ (**FN014_W2_1**) Yuan per month / Or ______ 0.00...100.00 (**FN014_W2_2**) percent of wage
[IWER: Use -1 for unknown, ask unfolding bracket questions for -1 or 0 Yuan 11 0]

FN014_W2_bracket [IWER: If R is unwilling to answer or does not remember, ask unfolding bracket questions here]50 /100 /200 /400 /700 yuan

FN017_W2 For [Name of Pension], how many years of contribution history have you established so far in this province/city? [Whether or not working for the same employer, countable working history/social insurance contribution years or equivalent contribution years should be counted.] []/ / [/] ______ 0.00...100.00 Years

FN017_W3 For [Name of Pension], how many years of contribution history have you established so far in other provinces/cities? [Whether or not working for the same employer, countable working history/social insurance contribution years or equivalent contribution years should be counted] []/ / [/] ______ 0.00...100.00 Years

FN019_W4 Can you receive pension benefits from [Name of Pension] in the future? []

1. Yes
2. No → Skip to FN014_W4_a FN014_W4_a

997. Unknown → Skip to FN014_W4_a FN014_W4_a

FN020_W4 At what age do you expect to receive benefits from [Name of Pension]? []______ 45...120 Years old

FN021_W2 How much do you expect to receive from [Name of Pension]? It can be in the percentage of your wage before retirement or the amount of monthly benefit [] （**FN021_W2_1**）Yuan per month / Or ______ 0.00..100.00（**FN021_W2_2**）percent of wage before retirement of final pay %

[IWER: Use -1 for unknown, ask unfolding bracket questions for -1 or 0 Yuan 11 0]

FN021_W2_bracket [IWER: IfR is unwilling to answer, does not remember, or the answer is 0 Yuan, ask unfolding bracket questions 0] 1,000 /1,500 /2,000 /3,000 /4,500 Yuan

FN014_W4_a When you enrolled in [Name of Pension], do you need to make up the contribution? []

[IWERThe make-up contribution to pension programs for public institutions and enterprises refers to the lump sum or multiple payments that R wants to pay in the following cases: R predicts insufficient contribution history at retirement and needs to make up the previous contributions, or R hopes to receive more benefits in the future]

1. Yes
2. No → Skip to FN030_W4 FN030_W4

FN014_W4_b For [Name of Pension], how much do you need to make up the contribution overall? []

1. ______ (**FN014_W4_b_1**) Yuan
2. ______ (**FN014_W4_b_2**) 10,000 Yuan

[IWER: Use -1 for unknown, ask unfolding bracket questions for -1 or 0 Yuan 11 0]

FN014_W4_b_bracket [IWER: IfR is unwilling to answer, does not remember, or the answer is 0 Yuan, ask unfolding bracket questions 0] 1,000/3,000 /5,000 /8,000 /10,000 Yuan

FN014_W4_c For [Name of Pension], when did you make up the contribution? [] ______ 1900...2018 (**FN014_W4_c_1**) Year ______ 0...12 (**FN014_W4_c_2**) Month

[IWERYear is in four digits. Month is in its actual value. For example, January is 1 not 01, December is 12. IfR does not remember month, fill 0 : 4 1 10112 12 0]

PART 2 Supplementary Pension Insurance (Annuity) ()

FN030_W4 Do you currently receive, or expect to receive or contribute to supplementary pension insurance of government and public institutions (Annuity), or Enterprises (Enterprise annuity)? , () ()

1. Yes I currently participate in or receive pension benefits
2. No I currently do not participate in or receive pension benefits → Skip to FN058_W4 FN058_W4

FN030_W4_a Which supplementary pension do you currently receive, or expect to receive or contribute to? ,

1. Supplementary Pension for public servants (occupational annuity for public servants) ()
2. Supplementary Pension for public institution employees (occupational annuity for public institution employees) ()
3. supplementary pension for enterprise employees (enterprise annuity) ()
4. Unknown

PROCEDURE :

For each choice chosen in FN030_W4_aask FN031_W4 - FN037_W2 FN030_W4_a FN031_W4 - FN037_W2

To generate [Name of Pension]: If FN030_W4_a = 1, 2, 3, then [Name of Pension] is the corresponding choice, if FN030_W4_a = 4, then [Name of Pension] is Supplementary Pension for public servants, public institution employees, or for enterprise employees [] FN030_W4_a = 1, 2, 3 [] FN030_W4_a= 4 []

FN031_W4 Do you currently receive benefits from [Name of Pension]? [ ]

1. Yes I do
2. No I have not started to receive any benefits

FN032_W4 Which type does your [Name of Pension] belong to? []

[IWER: DB Retirement Pension Plan: The pension benefit is pre-determined and the contributionis determined by the benefit accordingly DC Retirement Pension Plan: The contribution is pre-determined, and the pension benefit depends on the management of the pension funds]

1. Defined Benefit (DB) Retirement Pension
2. Defined Contribution (DC) Retirement Pension

997. Unknown type

PROCEDURE :

If FN031_W4 = 1, Skip to FN041_W2 FN031_W4 = 1, FN041_W2

FN034_W4 For [Name of Pension], where do you currently contribute? []

1. The same as current residence BB001_W3 BB001_W3
2. County/city/district of current residence BB001_W3 BB001_W3 // ______ (**FN034_W4_1**) other townships/subdistricts // ______ (**FN034_W4_2**) villages/neighborhoods /
3. Other ______ (**FN034_W4_3**) province_city_county/city/district _ _ // ____ (**FN034_W4_4**) township/subdistrict/village/neighborhood ////

997. Unknown

FN033_W2 For how many years have you enrolled in [Name of Pension]? [Including years working for different employers but enrolling in the same pension program] [ ] [] ____ 0.00...100.00 Years

FN035_W4_a Does [Name of Pension] that you expect to receive benefit from or that you contribute to require contributions from you or your employer? []

1. Yes
2. No → Skip to FN038_W2 FN038_W2

FN035_W4_b For [Name of Pension], how much does your employer currently contribute?
[] ______ (**FN035_W4_b_1**) Yuan per month / Or ______ 0.00...100.00 (**FN035_W4_b_2**) percent of wage
[IWER: Use -1 for unknown, ask unfolding bracket questions for -1 or 0 Yuan 11 0]

FN035_W4_b_bracket [IWER: IfR is unwilling to answer, does not remember, or the answer is 0 Yuan, ask unfolding bracket questions 0] 500 /1,000 /2,000 /3,500 /5,000 Yuan

FN035_W4_c For [Name of Pension], how much do you currently pay by yourself? [] ______ (**FN035_W4_c_1**) Yuan per month / Or ______ 0.00...100.00 (**FN035_W4_c_2**) percent of wage
[IWER: If R is unwilling to answer, does not remember, or the answer is 0 Yuan, ask unfolding bracket questions -1 -1 0]

FN035_W4_c_bracket [IWER: IfR is unwilling to answer or does not remember, ask unfolding bracket questions here 0] 500 /1,000 /2,000 /3,500 /5,000 yuan

FN038_W2 What is the earliest age at which you could leave this employer and start to receive benefits from [Name of Pension]? [ ]______ 45...120 Years old

FN039_W4 How much do you expect to receive from [Name of Pension]? It can be in the percentage of your wage before retirement or it can be the amount of monthly benefit [] _（**FN039_W4_1**）Yuan per month / Or ______ 0.00..100.00（**FN039_W4_2**）of wage before retirement %
[IWER: Use -1 for unknown, ask unfolding bracket questions for -1 or 0 Yuan 11 0]

FN039_W4_bracket [IWER: IfR is unwilling to answer, does not remember, or the answer is 0 Yuan, ask unfolding bracket questions 0] 500 /1,000 /2,000 /3,500 /5,000 Yuan

PROCEDURE :
If FN032_W4 = 2, 997, Skip to FN037_W2 FN032_W4 = 2, 997 FN037_W2 If FN032_W4 = 1, Skip to FN058_W4 FN032_W4 = 1 FN058_W4

FN041_W2 In what month and year did you start to receive pension benefits from [Name of Pension]? [] ______ 1900...2018 (**FN041_W2_1**) Year ______ 0...12 (**FN041_W2_2**) Month

[IWERYear is in four digits. Month is in its actual value. For example, January is 1 not 01, December is 12. If R does not remember the month, fill 0 : 4 1 10112 12 0]

FN042_W2 How much is your benefit from [Name of Pension] each month? [ ] ______ Yuan per month /

[IWER: Use -1 for unknown, ask unfolding bracket questions for 1 or 0 Yuan 11 0]

FN042_W2_bracket [IWER: IfR is unwilling to answer, does not remember, or the answer is 0 Yuan, ask unfolding bracket questions 0] 500 /1,000 /2,000 /3,500 /5,000 Yuan

FN042_W4 Where do you receive benefits from [Name of Pension]? [ ]

1. The same as current residence BB001_W3 BB001_W3
2. County/city/district of current residence BB001_W3 BB001_W3 // ______ (**FN042_W4_1**) other townships/subdistricts // ______ (**FN042_W4_2**)villages/neighborhoods /
3. Other ______ (**FN042_W4_3**) province_city_county/city/district _ _ // ____ (**FN042_W4_4**) township/subdistrict/village/neighborhood ////

997. Unknown

**PROCEDURE** :
If FN032_W4 = 2, 997, ask FN037_W2 FN032_W4 = 2, 997 FN037_W2 If FN032_W4 = 1, Skip to FN058_W4 FN032_W4 = 1 FN058_W4

FN037_W2 Have you ever checked your account balance of [Name of Pension]? []

1. Yes ______ (**FN037_W2_1**) Yuan in ______ 1900...2018 (**FN037_W2_2**) Year ___0...12 (**FN037_W2_3**) Month

   [IWERYear is in four digits. Month is in its actual value. For example, January is 1 not 01, December is 12. IfR does not remember the month, fill 0 : 4 1 10112 120]
2. No

PART 3 Urban and Rural Resident Pension, New Rural Resident Pension and Urban Resident Pension

FN058_W4 Do you currently receive, or expect to receive or contribute to Urban and Rural Resident Pension, New Rural Resident Pension, and Urban Resident Pension?

1. Yes I do → Skip to FN058_W4_a FN058_W4_a 2. No I do not participate or receive any benefit

FN069_W4 Are you eligible for Urban and Rural Resident Pension, New Rural Resident Pension, and Urban Resident Pension? In other words, your hukou-registered village or neighborhood implemented the three pension programs above, and you can participate if you are willing to.

1. Yes → Skip to FN057_W4_4 FN057_W4_4
2. No

997. I do not know → Skip to FN057_W4_4 FN057_W4_4

FN069_W4_a The reason for ineligibility

1. Not available in my Hukou registration area
2. I have participated in government and public institution pension or basic pension for enterprise workers 3. Other please specify ______ (**FN069_W4_a_1**)

**PROCEDURE** :
Skip to FN103_W4 FN103_W4

FN057_W4_4 According to the age on yourID, have you reached the age requirement to receive the following pensions for residents? Pensions for residents include Urban and Rural Resident Pension, New Rural Resident Pension, and Urban Resident Pension

1. Yes
2. No → Skip to FN057_W3_6 FN057_W3_6

FN057_W4_5 What is the reason that you do not receive any benefits?

1. did not pay the insurance premium
2. I am not in my hukou place
3. Do not know where to receive

4. Poor health, cannot go and receive
5. Other please specify ______ (**FN057_W4_5_1**)

PROCEDURE :
Skip to FN103_W4 FN103_W4

FN057_W3_6 What is the reason that you do not pay the premium? 1. No money
2. I do not satisfy with the benefit, it is not worthwhile to participate
3. Low benefit level, does not make any differences to my life
4. It is not convenient to apply and to pay
5. The design of the payments is not reasonable
6. I do not have the local hukou
7. I have already enrolled in other pension plans, and cannot double enroll
8. Others please specify ______ (**FN057_W3_6_1**)

PROCEDURE :
Skip to FN103_W4 FN103_W4

FN058_W4_a Which pension program do you currently receive benefit from, expect to receive benefit from, or contribute to? ?
1. Urban and Rural Resident Pension
2. New Rural Resident Pension
3. Urban Resident Pension
4. I do not know the type

PROCEDURE :
For each choice chosen in FN058_W4_a, ask FN058_W4_b - FN072_W4_b FN058_W4_a FN058_W4_b - FN072_W4_b
To generate [Name of Pension], if FN058_W4_a = 1, 2, 3, then [Name of Pension] is the corresponding choice [] FN058_W4_a = 1, 2, 3 []
If FN058_W4_a = 4, then [Name of Pension] is Urban and Rural Resident Pension, New Rural Resident Pension, and Urban Resident Pension FN058_W4_a = 4 [ ]

FN058_W4_b Do you currently receive pension benefits from [Name of Pension]? []
1. Yes I do → Skip to FN067_W2 FN067_W2

2. No I have not started to recieve any benefits

FN061_W4 For how many years have you contributed to or participated in other ways in [Name of Pension]? [] ______ 0.00...100.00 Year

FN059_W2 Where did you enroll in [Name of Pension]? [] 1. The same as current residence BB001_W3 BB001_W3

2. County/city/district of current residence BB001_W3 BB001_W3 // ______ (**FN059_W4_1**) other townships/subdistricts // ______ (**FN059_W4_2**) villages/neighborhoods /
3. Other ______ (**FN059_W4_3**) province_city_county/city/district _ _ // ____ (**FN059_W4_4**) township/subdistrict/village/neighborhood ////

997. Unknown

FN062_W4 Does [Name of Pension] that you expect to receive benefit from or you currently contribute to require contributions? Contributions include individual contributions and government subsidies []

1. Yes
2. No → Skip to FN065_W2 FN065_W2

FN062_W4_a How much does the government subsidize your [Name of Pension]? [] ______ (**FN062_W4_a_1**) Yuan per month / Or ______ (**FN062_W4_a_2**) Yuan per year / [IWER: Use -1 for unknown, ask unfolding bracket questions for -1 or 0 Yuan 11 0]

FN062_W4_a_bracket [IWER: IfR is unwilling to answer or does not remember, ask unfolding bracket questions here] 5 /10 /15 /20 /30 Yuan

FN062_W4_b For your [Name of Pension], how much is your current individual contribution? [] ______ (**FN062_W4_b_1**) Yuan per month / Or ______ (**FN062_W4_b_2**) Yuan per year / [IWER: Use -1 for unknown, ask unfolding bracket questions for -1 or 0 Yuan 11 0]

FN062_W4_b_bracket [IWER: IfR is unwilling to answer, does not remember, or the answer is 0 Yuan, ask unfolding bracket questions 0] 10 /50 /100 /200 /500 Yuan

FN065_W2 When do you expect to receive pension benefits from [Name of Pension] ? []At age ______ 45...120 (**FN065_W2_1**) years old or in _____ 0.00...100.00 (**FN065_W2_2**) years

[Soft Check: Prompt or Verify if FN065_w2_1 < 50 or FN065_w2_2 > 60]

FN066_W2 How much do you expect to receive from [Name of Pension]? [ ] ______ (**FN066_W2_1**) Yuan per month / Or ______ (**FN066_W2_2**) Yuan （Lump sum amount ）

[IWER: Use -1 for unknown, ask unfolding bracket questions for -1 or 0 Yuan 11 0]

[Soft Check: Prompt or Verify if These Benefits are Low or High, e.g., FN066_w2_1 < 100 | FN066_w2_1 > 5000 per month.]

FN066_W2_bracket [IWER: IfR is unwilling to answer, does not remember, or the answer is 0 Yuan, ask unfolding bracket questions 0] 55 /60 /75 /100 /400 Yuan

FN063_W4 For those who currently contribute to or participate in other ways in pension programs for residents, have you ever checked your account balance of [Name of Pension]? []

1. Yes ______ （**FN063_W4_1**） Yuan in ______ 1900...2018 （**FN063_W4_2**） Year ____ 0...12（**FN063_W4_3**） Month

   [IWERYear is in four digits. Month is in its actual value. For example, January is 1 not 01, December is 12. IfR does not remember the month, fill 0 : 4 1 10112 120]
2. No

**PROCEDURE** :<br>Skip to FN072_W3 FN072_W3

FN067_W2 In what month and year did you start to receive benefits from [Name of Pension]? []______ 1900...2018 (**FN067_W2_1**) Year ______ 0...12 (**FN067_W2_2**) Month

[IWERYear is in four digits. Month is in its actual value. For example, January is 1 not 01, December is 12. If R does not remember the month, fill 0 : 4 1 10112 12 0]

FN068_W2 How much do you currently receive from [Name of Pension] each month? [] __ Yuan per month /

[IWER: Use -1 for unknown, ask unfolding bracket questions for -1 or 0 Yuan -1 -1 0]

FN068_W2_bracket [IWER: If R is unwilling to answer or does not remember, ask unfolding bracket questions here 0] 55 /60 /75 /85 /100 Yuan

FN070_W4 Where do you receive the pension from [Name of Pension]? [ ]

1. The same as current residence BB001_W3 BB001_W3
2. County/city/district of current residence BB001_W3 BB001_W3 // ______ (**FN070_W4_1**) other townships/subdistricts // ______ (**FN070_W4_2**) villages/neighborhoods /
3. Other ______ (**FN070_W4_3**) province_city_county/city/district _ _ // ____ (**FN070_W4_4**) township/subdistrict/village/neighborhood ////

997. Unknown

FN072_W3 Do you need to pay make-up contributions when you enroll in [Name of Pension]? []

[IWERThe make-up contribution for (new pension for rural residents) refers to the lump-sum payment or multiple payments that the R wants to pay in the following cases: R predicts a less-than-15 year contribution history by the age of 60, or has never participated and needs to make up previous contributions, or R hopes to receive more pensions in the future （） 60 15]

1. Yes
2. No → Skip to FN103_W4 FN103_W4

997. Unknown → Skip to FN103_W4 FN103_W4

999. Refuse to answer → Skip to FN103_W4 FN103_W4

FN072_W3_1 For [Name of Pension], how much do you make up for? []

1. ______ (**FN072_W3_1_1**) Yuan
2. ______ (**FN072_W3_1_2**) 10,000Yuan

[IWER: Use -1 for unknown, ask unfolding bracket questions for -1 or 0 Yuan 11 0]

FN072_W3_1_bracket [IWER: IfR is unwilling to answer, does not remember, or the answer is 0 Yuan, ask unfolding bracket questions 0] 1,000/3,000 /5,000 /8,000 /10,000 Yuan

FN072_W4_b For [Name of Pension], when did you make up the contribution? [] ______ 1900...2018 (**FN072_W4_b_1**) Year ___ 0...12 (**FN072_W4_b_2**) Month

[IWERYear is in four digits. Month is in its actual value. For example, January is 1 not

01, December is 12. If R does not remember the month, fill 0 : 4 1 10112 12 0]

FN103_W4 Have you ever participated in the Rural Social Pension Insurance (old rural pension) ( )

[IWER: Rural pension insurance refers to the Rural Social Pension Insurance. At the end of 1992, the Ministry of Civil Affairs promulgated Basic scheme of county-level rural social pension insurance confirmed that the rural social pension was implemented at the county level 1992]

1. Yes
2. No

PART 4 Pension for Land-Expropriated Farmers (/)

FN079_W2_3 Do you currently receive or expect to receive or contribute to the pension for land-expropriated farmers?

1. Currently enroll have not received any benefits
2. Currently receive benefits → Skip to FN079_W2_10
   FN079_W2_10
3. No → Skip to FN073_W4 FN073_W4

FN079_W4 Where did you participate in the pension?

1. The same as current residence BB001_W3 BB001_W3
2. County/city/district of current residence BB001_W3 BB001_W3 // ______ (**FN079_W4_1**) other townships/subdistricts // ______ (**FN079_W4_2**) villages/neighborhoods /
3. Other ______ (**FN079_W4_3**) province_city_county/city/district _ _ // __ (**FN079_W4_4**) township/subdistrict/village/neighborhood ////

997. Unknown

FN079_W2_4 Does your pension for land-expropriated farmers require individual contributions?

1. Yes
2. No → Skip to FN079_W2_8 FN079_W2_8

FN079_W2_6 How much do you need to pay? ______ (**FN079_W2_6_1**) Yuan per month / or ____ (**FN079_W2_6_2**) Yuan per year /

[IWER: Use -1 for unknown, ask unfolding bracket questions for -1 or 0 Yuan 11 0]

FN079_W2_6_bracket [IWER: IfR is unwilling to answer, does not remember, or the answer is 0 Yuan, ask unfolding bracket questions 0] 100 /500 /800 /1,000 /1,500 Yuan/month /

FN079_W2_8 When do you expect to receive this pension At age ______ 45...120 (**FN079_W2_8_1**) years old or in ______ 0.00...100.00 (**FN079_W2_8_2**) years

FN079_W2_9 How much do you expect to receive? ______ (**FN079_W2_9_1**) Yuan per month / Or ___ (**FN079_W2_9_2**) Yuan （Lump sum amount ）

[IWER: Use -1 for unknown, ask unfolding bracket questions for -1 or 0 Yuan 11 0]

FN079_W2_9_bracket [IWER: IfR is unwilling to answer, does not remember, or the answer is 0 Yuan, ask unfolding bracket questions 0] 100 /500 /1,000 /1,500 /2,000 Yuan/month /

PROCEDURE :
Skip to FN073_W4 FN073_W4

FN079_W2_10 In what month and year did you start to receive this pension? ______ 1900...2018 (**FN079_W2_10_1**) Year ______ 0...12 (**FN079_W2_10_2**) Month

[IWERYear is in four digits. Month is in its actual value. For example, January is 1 not 01, December is 12. If R does not remember the month, fill 0 : 4 1 10112 12 0]

FN079_W2_11 How much do you currently receive each month? ______ Yuan per month /
[IWER: Use -1 for unknown, ask unfolding bracket questions for -1 or 0 Yuan 11 0]

FN079_W2_11_bracket [IWER: IfR is unwilling to answer, does not remember, or the answer is 0 Yuan, ask unfolding bracket questions 0] 10 /50 /100 /500 /1,000 Yuan/month /

FN080_W4 Where do you currently receive the benefits?
1. The same as current residence BB001_W3 BB001_W3
2. County/city/district of current residence BB001_W3 BB001_W3 // ______ (**FN080_W4_1**) other townships/subdistricts // ______ (**FN080_W4_2**) villages/neighborhoods /
3. Other ______ (**FN080_W4_3**) province_city_county/city/district _ _ // ____ (**FN080_W4_4**) township/subdistrict/village/neighborhood ////

997. Unknown

PART 5 Life Insurance

FN073_W4 Do you currently receive or expect to receive or contribute to any life insurance?

1. Yes ,
2. No → Skip to FN043_W4 FN043_W4

FN056_W2_3 What kind of life insurance did you buy?

[IWER: If R has multiple life insurances policies, record the most important one]

1. Term life insurance
2. Whole life insurance
3. Pure endowment insurance
4. Endowment insurance
5. Other please specify ______ (**FN056_W2_3_1**)

FN056_W2_7 have you ever received a life insurance payout?

1. Yes → Skip to FN077_W4 FN077_W4
2. No

FN074_W4 Where did you participate in this insurance?

1. The same as current residence BB001_W3 BB001_W3
2. County/city/district of current residence BB001_W3 BB001_W3 // ______ (**FN074_W4_1**) other townships/subdistricts // ______ (**FN074_W4_2**) villages/neighborhoods /
3. Other ______ (**FN074_W4_3**) province_city_county/city/district _ _ // ____ (**FN074_W4_4**) township/subdistrict/village/neighborhood ////

997. Unknown

FN075_W4 For how many years have you contributed to or participated in other ways in this pension? ______ 0.00..100.00 Year(s)

FN056_W2_5 How to pay the insurance premium?

1. Monthly
2. Quaterly
3. Biannually
4. Annually

FN056_W2_6 How much is the payment amount?

1. If pay it monthly ______ (**FN056_W2_6_1**) Yuan/Month / 2. If pay it quarterly ______ (**FN056_W2_6_2**) Yuan/Quarter /

3. If pay it biannually ______ (**FN056_W2_6_3**) Yuan/Half a year / 4. If pay it annually (**FN056_W2_6_4**) Yuan/Year /

FN076_W4 In the future, can you receive pension benefits for yourself from the life insurance mentioned above

1. Yes
2. No → Skip to FN043_W4 FN043_W4

FN076_W4_a When do you expect to claim life insurance benefits? At age ______ 45...120 (**FN076_W4_a_1**) years old or in ______ 0.00...100.00 (**FN076_W4_a_2**) years

FN056_W2_9 How much do you expect to receive? __ (**FN056_W2_9_1**) Yuan/Month / Or __ (**FN056_W2_9_2**) Yuan （Lump sum amount ）

[IWER: Use -1 for unknown, ask unfolding bracket questions for -1 or 0 Yuan 11 0]

FN056_W2_9_bracket [IWER: IfR is unwilling to answer, does not remember, or the answer is 0 Yuan, ask unfolding bracket questions 0] 1,000/2,000 /3,000 /6,000 /10,000 Yuan

PROCEDURE :

Skip to FN043_W4 FN043_W4

FN077_W4 In what month and year did you start to receive benefits from life insurance? _ 1900...2018 (**FN077_W4_1**) Year ______ 0...12 (**FN077_W4_2**) Month

[IWERYear is in four digits. Month is in its actual value. For example, January is 1 not 01, December is 12. If R does not remember the month, fill 0 : 4 1 10112 12 0]

FN056_W2_8 How much did you get? ______ Yuan

[IWER: Use -1 for unknown, ask unfolding bracket questions for -1 or 0 Yuan 11 0]

FN056_W2_8_bracket [IWER: IfR is unwilling to answer, does not remember, or the answer is 0 Yuan, ask unfolding bracket questions 0] 1,000/3,000 /5,000 /8,000 /10,000 Yuan

FN078_W4 From where do you receive these life insurance benefits?

1. The same as current residence BB001_W3 BB001_W3

2. County/city/district of current residence BB001_W3 BB001_W3 // ______ (**FN078_W4_1**) other townships/subdistricts // ______ (**FN078_W4_2**) villages/neighborhoods /
3. Other ______ (**FN078_W4_3**) province_city_county/city/district _ _ // ____ (**FN078_W4_4**) township/subdistrict/village/neighborhood ////

997. Unknown

PART 6 Commercial Pension Insurance（Exclude Life Insurance）（）

FN043_W4 Do you currently receive or expect to receive or contribute to any commercial pension insurance?

1. Yes, I purchased
2. Yes, Im receivng the benefits → Skip to FN055_W2 FN055_W2 3. Neither → Skip to FN083_W2 FN083_W2

FN044_W2 Where did you participate in this insurance?

1. The same as current residence BB001_W3 BB001_W3
2. County/city/district of current residence BB001_W3 BB001_W3 // ______ (**FN044_W4_1**) other townships/subdistricts // ______ (**FN044_W4_2**) villages/neighborhoods /
3. Other ______ (**FN044_W4_3**) province_city_county/city/district _ _ // ____ (**FN044_W4_4**) township/subdistrict/village/neighborhood ////

997. Unknown

FN047_W2 How do you contribute to the commercial pension? ? 1. By annually

2. By lump sum payment → Skip to FN046_W4 FN046_W4

FN045_W4 For how many years have you contributed to or participated in other ways in this pension? ______ 0.00..100.00 Year(s)

FN048_W2 How much premium do you pay every year ______ Yuan/year /

[IWER: Use -1 for unknown, ask unfolding bracket questions for -1 or 0 Yuan 11 0]

FN048_W2_bracket [IWER: IfR is unwilling to answer, does not remember, or the answer is 0 Yuan, ask unfolding bracket questions 0] 3,000 /6,000 /10,000 /20,000 /30,000 Yuan per year /

FN049_W2 How many years do you need to pay? ? _______ 0.00..100.00 Years

[Soft Check: Prompt for verification if greater than a legal maximum]

**PROCEDURE** :<br>Skip to FN051_W4 FN051_W4

FN046_W4 When did you start paying for the commercial pension? ______ 1900...2018 (**FN046_W4_1**) Year ______ 0...12 (**FN046_W4_2**) Month

[IWERYear is in four digits. Month is in its actual value. For example, January is 1 not 01, December is 12. If R does not remember the month, fill 0 : 4 1 10112 12 0]

FN050_W2 How much premium do you need to pay in total? ______ Yuan

[IWER: Use -1 for unknown, ask unfolding bracket questions for -1 or 0 Yuan 11 0]

FN050_W2_bracket [IWER: IfR is unwilling to answer, does not remember, or the answer is 0 Yuan, ask unfolding bracket questions 0] 1,000 /2,000 /5,000 /10,000 /20,000 Yuan

FN051_W4 When do you expect to claim this insurance benefit? At age ______ 45...120 (**FN051_W4_1**) years old or in ______ 0.00...100.00 (**FN051_W4_2**) years

FN051_W2 How do you receive the pension?

1. 1.A lump-sum payout → Skip to FN054_W4 FN054_W4 2. Annually
3. Monthly → Skip to FN053_W2 FN053_W2

FN052_W4 How much benefit do you expect to receive? ______ Yuan/year /

[IWER: Use -1 for unknown, ask unfolding bracket questions for -1 or 0 Yuan 11 0]

[Soft Check: Prompt for verification if low or high, e.g. FN052_w4 < 1200 per year or FN052_w4 > 60000 per year]

FN052_W4_bracket [IWER: IfR is unwilling to answer, does not remember, or the answer is 0 Yuan, ask unfolding bracket questions 0] 1,200 /6,000 /12,000 /24,000 /36,000 Yuan

**PROCEDURE** :<br>Skip to FN083_W2 FN083_W2

FN053_W2 How much do you expect to receive each month in the future? ______ Yuan/month /
[IWER: Use -1 for unknown, ask unfolding bracket questions for -1 or 0 Yuan 11 0]
[Soft Check: Prompt for verification if low or high, e.g. FN053_w2 < 100 per month or FN053_w2 >5000 per year]

FN053_W2_bracket [IWER: IfR is unwilling to answer, does not remember, or the answer is 0 Yuan, ask unfolding bracket questions 0] 500 /1,000 /2,000 /3,500 /5,000 Yuan

**PROCEDURE** :<br>Skip to FN083_W2 FN083_W2

FN054_W4 How much lump sum benefit do you expect to receive? ______ Yuan
[IWER: Use -1 for unknown, ask unfolding bracket questions for -1 or 0 Yuan 11 0]

FN054_W4_bracket [IWER: IfR is unwilling to answer, does not remember, or the answer is 0 Yuan, ask unfolding bracket questions 0] 1,000 /5,000 /10,000 /50,000 /100,000 Yuan

**PROCEDURE** :<br>Skip to FN083_W2 FN083_W2

FN055_W2 In what month and year did you start to receive commercial pension benefits? _
1900…2018 (**FN055_W2_1**) Year ______ 0…12 (**FN055_W2_2**) Month
[IWERYear is in four digits. Month is in its actual value. For example, January is 1 not 01, December is 12. If R does not remember the month, fill 0 : 4 1 10112 12 0]

FN056_W2 What is your monthly benefit? ? ________ Yuan per month /
[IWER: Use -1 for unknown, ask unfolding bracket questions for -1 or 0 Yuan 11 0]

FN056_W2_bracket [IWER: IfR is unwilling to answer, does not remember, or the answer is 0 Yuan, ask unfolding bracket questions 0] 500 /1,000 /2,000 /3,500 /5,000 Yuan

FN056_W4 Where do you currently receive benefits from this commercial insurance?

1. The same as current residence BB001_W3 BB001_W3
2. County/city/district of current residence BB001_W3 BB001_W3 // ______ (**FN056_W4_1**) other townships/subdistricts // ______ (**FN056_W4_2**) villages/neighborhoods /
3. Other ______ (**FN056_W4_3**) province_city_county/city/district _ _ // ____ (**FN056_W4_4**) township/subdistrict/village/neighborhood ////

997. Unknown

PART 7 Other Pension

FN083_W2 In addition to the aforementioned pension programs, do you currently receive or expect to receive or contribute to any other pension?

1. Yes participate in /
2. Yes receive
3. No

PROCEDURE :

If FN083_W2 = 3 and (FN002_W4 = 1 or FN030_W4 = 1 or FN058_W4 = 1 or FN079_W2_3 = 1, 2 or FN073_W4 = 1 or FN043_W4 = 1, 2)Skip to FN097_W2 FN083_W2 = 3 (FN002_W4 = 1 FN030_W4 = 1 FN058_W4 = 1 FN079_W2_3 = 1, 2 FN073_W4 = 1 FN043_W4 = 1, 2) FN097_W2

If FN083_W2 = 3 and FN002_W4 = 2 and FN030_W4 = 2 and FN058_W4 = 2 and FN079_W2_3 = 3 and FN073_W4 = 2 and FN043_W4 = 3 Skip to FN099_W4 FN083_W2 = 3 FN002_W4 = 2 FN030_W4 = 2 FN058_W4 = 2 FN079_W2_3 = 3 FN073_W4 = 2 FN043_W4 = 3 FN099_W4

FN084_W2 What is the name of the program? ______

[IWER: If R participates/receives multiple other types of pensions, choose the most important one to answer /]

FN084_W4 Do you currently receive benefits from this pension program?

1. Yes → Skip to FN095_W2 FN095_W2
2. No

FN085_W2 Where did you participate in the pension program? 1. The same as current residence BB001_W3 BB001_W3

2. County/city/district of current residence BB001_W3 BB001_W3 // ______ (**FN085_W4_1**) other townships/subdistricts // ______ (**FN085_W4_2**) villages/neighborhoods /
3. Other ______ (**FN085_W4_3**) province_city_county/city/district _ _ // ____ (**FN085_W4_4**) township/subdistrict/village/neighborhood ////

997. Unknown

FN087_W2 Did you need to pay the premium? 1. Yes
2. No → Skip to FN093_W2 FN093_W2

FN089_W2 How do you contribute to the pension? ? 1. Annually or Monthly
2. Lump sum payment → Skip to FN086_W4 FN086_W4

FN090_W4 How many years have you participated in this pension ______ 0.00...100.00 Year(s)

FN091_W2 How many years do you need to pay? ? _______ 0.00...100.00 Years
[Soft Check: Prompt for verification if greater than a legal maximum]

FN090_W2 On an annual basis, how much do you pay every year? ______ Yuan/year /
[IWER: Use -1 for unknown, ask unfolding bracket questions for -1 or 0 Yuan 11 0]

FN090_W2_bracket [IWER: IfR is unwilling to answer, does not remember, or the answer is 0 Yuan, ask unfolding bracket questions 0] 500 /1,000 /2,000 /3,000 /5,000 Yuan

**PROCEDURE** :<br>Skip to FN093_W2 FN093_W2

FN086_W4 When did you start paying this pension? ______ 1900...2018 (**FN086_W4_1**) Year 0...12 (**FN086_W4_2**) Month

[IWERYear is in four digits. Month is in its actual value. For example, January is 1 not 01, December is 12. If R does not remember the month, fill 0 : 4 1 10112 12 0]

FN092_W4 How much premium do you need to pay in total? ______ Yuan
[IWER: Use -1 for unknown, ask unfolding bracket questions for -1 or 0 Yuan 11 0]

FN092_W4_bracket [IWER: IfR is unwilling to answer, does not remember, or the answer is 0 Yuan, ask unfolding bracket questions 0] 1,000 /2,000 /5,000 /10,000 /20,000 Yuan

FN093_W2 When do you expect to receive pension At age ______ (**FN093_W2_1**) 45…120 or in ___ (**FN093_W2_2**) years

FN094_W2 How much do you expect to receive? ______ (**FN094_W2_1**) Yuan per month / Or (**FN094_W2_2**) Yuan （Lump sum amount ）

**PROCEDURE**：<br>Skip to FN097_W2 FN097_W2

FN095_W2 In what month and year did you start to receive this pension benefits? ______ 1900…2018 (**FN095_W2_1**) year ______ 0…12 (**FN095_W2_2**) month

[IWERYear is in four digits. Month is in its actual value. For example, January is 1 not 01, December is 12. If R does not remember the month, fill 0： 4 1 10112 12 0]

FN096_W2 How much do you currently receive each month? ______ Yuan per month /
[IWER: Use -1 for unknown, ask unfolding bracket questions for -1 or 0 Yuan 11 0]

FN096_W2_bracket [IWER: IfR is unwilling to answer, does not remember, or the answer is 0 Yuan, ask unfolding bracket questions 0] 500 /1,000 /2,000 /3,500 /5,000 Yuan

FN096_W4 Where do you currently receive this pension? 1. The same as current residence BB001_W3 BB001_W3
2. County/city/district of current residence BB001_W3 BB001_W3 // ______ (**FN096_W4_1**) other townships/subdistricts // ______ (**FN096_W4_2**) villages/neighborhoods /
3. Other ______ (**FN096_W4_3**) province_city_county/city/district _ _// ______ (**FN096_W4_4**) township/subdistrict/village/neighborhood //// 997. Unknown

**PROCEDURE**：<br>Skip to FN097_W2 FN097_W2

[Ask R who do not enroll in any pension]

FN099_W4 Do you currently receive, or expect to receive or contribute to the following pensions? (Choose all that apply) ()

[Hard Check: If the response is that R receives and enrolls the following pensions, option 1-13, IWER should check and confirm. If it was missing previously, go back and fill in the information 1-13]

1. Pension for public servants
2. Pension for public institution employees
3. Basic pension for enterprise employees
4. Supplementary pension for public servants (Annuity) ()
5. Supplementary pension for public institution employees (Annuity) ()
6. Supplementary pension for enterprise employees (Enterprise Annuity) ()
7. Urban and Rural Resident Pension
8. New Rural Resident Pension
9. Urban Resident Pension
10. Pension for land-expropriated farmers
11. Life insurance
12. Commercial pension insurance (Exclude life insurance) ( )
13. Other pension programs
14. Never claimed or enrolled in any pension program 997. Unknown
999. Refuse to answer

PROCEDURE:

If FN099_W4 = 14, 997, 999, ask FN100_W4 - FN102_W4 FN099_W4 = 14, 997, 999 FN100_W4 - FN102_W4

FN099_W4 = 14, 997, 999 cannot be chosen together with other options in FN099_W4 FN099_W4 = 14, 997, 999 FN099_W4

FN100_W4 Have you ever had any of the following pensions? (choose all that apply) ()

1. Pension for public servants
2. Pension for public institution employees
3. Basic pension for enterprise employees
4. Supplementary pension for public servants (Annuity) ()
5. Supplementary pension for public institution employees (Annuity) ()

6. Supplementary pension for enterprise employees (Enterprise Annuity) ( )
7. Urban and Rural Resident Pension
8. New Rural Resident Pension
9. Urban Resident Pension
10. Pension for land-expropriated farmers
11. Life insurance
12. Commercial pension insurance (Exclude life insurance) ( )
13. Pension insurance for rural residents (Old rural pension) ( ) 14. Other pension programs, please specify , ______ (**FN100_W4_1**)
15. Never claimed or enrolled in any pension program
997. Unknown
999. Refuse to answer

PROCEDURE :

If FN100_W4 = 1, 2, 3, 4, 5, 6, 7, 8, 9, 10, 11, 12, 13, 14then ask FN101_W4 - FN102_W4 in loops FN100_W4 = 1, 2, 3, 4, 5, 6, 7, 8, 9, 10, 11, 12, 13, 14 FN101_W4 - FN102_W4 To generate [Name of Pension] in a loop: if FN100_W4 = 1, 2, 3, 4, 5, 6, 7, 8, 9, 10, 11, 12, 13, 14 then [Name of Pension] is the corresponding option [] FN100_W4 = 1, 2, 3, 4, 5, 6, 7, 8, 9, 10, 11, 12, 13, 14 []

If FN100_W4 = 15, 997, 999then Skip to FN097_W2 FN100_W4 = 15, 997, 999 FN097_W2

FN101_W4 When did you stop participating in [Name of Pension]? [] . 1900...2018 (**FN101_W4_1**) Year ______ 0...12 (**FN101_W4_2**) Month

[IWER: Year is in four digits. Month is in its actual value. For example, January is 1 not 01, December is 12. If R does not remember the month, fill 0 : 4 1 10112 12 0]

FN102_W4 Why did you stop participating in [Name of Pension]? []

______

[Ask all R]

FN097_W2 If you are too old to work, what would be your financial resource?

[IWERCommercial insurance refers to the life insurance that R directly purchases from the commercial insurance company, pays premium regularly, and starts to receive benefits continuously and regularly at the contracted age]

1. Children → Skip to FN098_W2 FN098_W2
2. Savings → End this section
3. Pension → End this section
4. Commercial pension insurance → End this section
5. Other please specify ______ (**FN097_W2_1**) → End this section

FN098_W2 Which child(ren)? (choose all that apply) / （） 1-N. Preload the list of children

This page intentionally left blank

G & H Income, Expenditures and Assets

G2 Household Income and Expenditures

[IWER: Part 1_1 is asked of the main respondent and spouse respectively. Other parts in this section is asked of the family financial respondent. Do not allow a proxy respondent to answer the entire section Part 1_1]

PART 1 Household Wage Income and Individual-based Transfers

Part 1_1: Main Respondent and Spouses Wage Income and Individual-based Transfers

[IWER: Please conduct Part 1_1 when the main respondent and spouse are at home. Dontallow a proxy to complete the part Part 1_1]

GA001 Did you receive any wage and bonus income in the past year?

1. Yes
2. No → Skip to GA003_W4 GA003_W4

GA002 How much did you receive last year? _______ Yuan

[Soft Check: > 240, 000]

[IWER: IfR is unwilling to answer or does not remember, ask unfolding bracket questions]

GA002_bracket [CAPI: If Respondent is unwilling to answer, does not remember, or the input value is 0, please ask unfolding bracket questions here 0]
5,000/10,000/30,000/50,000/100,000 Yuan

GA002_W2_1 Does the above mentioned wage exclude any insurance, income tax, public housing funds and other fees?

1. Yes
2. No

997. Do not know
999. Refuse to answer → Skip to GA003_W4 GA003_W4

GA002_W2_2 What is the total amount of your insurance, income tax, public housing funds and other fees? /

1. ______ (**GA002_W2_2a**) Yuan/Year /
2. ______ (**GA002_W2_2b**) Yuan/Month /
3. About ______ (**GA002_W2_2c**) % of wage
4. No

997. Do not know

999. Refuse to answer

[IWER: Ask unfolding bracket questions for option 1,2,3 if respondent forget the value 123]

PROCEDURE :

If GA002_W2_2 = 1, but GA002_W2_2a = 0 or missing, ask the unfolding bracket questions GA002_W2_2 = 1 GA002_W2_2a = 0

If GA002_W2_2 = 2, but GA002_W2_2b = 0 or missing, ask the unfolding bracket questions GA002_W2_2 = 2 GA002_W2_2b = 0

If GA002_W2_2 = 3, but GA002_W2_2c = 0 or missing, ask the unfolding bracket questions GA002_W2_2 = 3 GA002_W2_2c = 0

If GA002_W2_2 = 997, ask the unfolding questions GA002_W2_2 = 997

GA002_W2_2_bracket What is the total amount of personal income tax, insurance, public housing funds and other fees? / 300/500/1,000/2,000/3,000 Yuan/Month /

GA003_W4 Did you receive any of the following types of income transfers in the past year? (check all that apply) ()

[F1: Medical Aid: Medical Aid for Serious Diseases refers to additional cash assistance outside of Medical Insurance Scope for urban and rural residents who are living difficultly caused by a major illness. Medical Aid play as final support outside the basic medical insurance F1]

1. Pensions (including wages from governments, public institutions and firms, supplemental pension of the firms,and income from such programs as rural pension insurance, Urban residents pension and commercial pension insurance, new rural social pension insurance) , // ______ (**GA003_W4_1**) Yuan
2. Unemployment compensation ______ (**GA003_W4_2**) Yuan 3. Pension voucher / ______ (**GA003_W4_3**) Yuan

4. Pension subsidy for the oldest old ______ (**GA003_W4_4**) Yuan
5. Workers compensation from Industrial Accident Compensation Insurance includes wage-replacement benefits, disability benefits, and survivors benefits ______ (**GA003_W4_5**) Yuan
6. Elderly family planning subsidies ______ (**GA003_W4_6**) Yuan
7. Medical aid ______ (**GA003_W4_7**)Yuan
8. Other government subsidies please specify ______ (**GA003_W4_8_1**) ______ (**GA003_W4_8**) Yuan
9. Other social income transfer sources please specify ______ (**GA003_W4_9_1**) ______ (**GA003_W4_9**) Yuan
10. None of the above → Skip to GA009_W4 BRANCHPOINT GA009_W4 BRANCHPOINT

PROCEDURE:

If GA003_W4 = 3, ask GA004_W4_3_1 GA003_W4 = 3/ GA004_W4_3_1

GA004_W4_3_1 How do you use the voucher? (check all that apply) / （ ）

1. Life care
2. Domestic service
3. Rehabilitation service
4. Buy food
5. Buy medicine
6. Buy life items
7. Emergency
8. Other please specify ______ (**GA004_W4_3_2**)

PROCEDURE:

If GA003_W4 = 1 andGA003_W4_1 = 0 / missing, Respondent received pension but did not answer how much received, or input value is 0, ask GA004_W4__1_bracketGA003_W4 = 1 GA003_W4_1 = 0 0 GA004_W4_1_bracket

GA004_W4_1_bracket How much pension have you received last year? /

400 /600 /1,000 /3,000 /20,000 Yuan /

GA009_W4 BRANCHPOINT:

If XRType = REIW, Skip to GA009_W4_1, ask GA009_W4_1 to GA009_W4_5 XRType = REIW GA009_W4_1 GA009_W4_1 GA009_W4_5

If XRType = NEWIW, Skip to GA011_W4_1, ask GA011_W4_1 to GA011_W4_5 XRType = NEWIW GA011_W4_1 GA011_W4_1 GA011_W4_5

GA009_W4_1 Have you ever inherited anything since 2013? 2013 1. Yes ______ (**GA009_W4_1_1**) times

2. No → Skip to GA000_W4 GA000_W4

999. Refuse to answer → Skip to GA000_W4 GA000_W4

**PROCEDURE** :<br>If GA009_W4_1 = 1 and GA009_W4_1_1 = 1ask GA009_W4_2 and GA009_W4_3<br>GA009_W4_1 = 1 GA009_W4_1_1 = 1 GA009_W4_2 GA009_W4_3

GA009_W4_2 How much have you inherited? The original value when inherited was __ (**GA009_W4_2_1**) Yuan When did the inheritance occur? ______ (**GA009_W4_2_2**) Year

[Soft Check: GA009_W4_2_1 > 200, 000]

GA009_W4_3 From whom you inherited

1. Parents
2. Parents-in-law
3. Children
4. Relatives
5. Others

**PROCEDURE** :<br>If GA009_W4_1 = 1 andGA009_W4_1_1 > 1 or missingask GA009_W4_4 and GA009_W4_5<br>GA009_W4_1 = 1 GA009_W4_1_1 > 1 GA009_W4_4 GA009_W4_5

GA009_W4_4 How much in total have you inherited? The original value when inherited was ______ (**GA009_W4_4_1**) Yuan

The amount for largest inheritance was ______ (**GA009_W4_4_2**) Yuan

When did the largest inheritance occur? ______ (**GA009_W4_4_3**) Year

[Soft Check: GA009_W4_4_2 > 200, 000]

GA009_W4_5 The largest inheritance was inerited from whom?

1. Parents
2. Parents-in-law
3. Children
4. Relatives
5. Others

**PROCEDURE** :<br>If new R, ask GA011_W4_1 to GA011_W4_5 GA011_W4_1 GA011_W4_5

GA011_W4_1 Have you ever inherited anything

1. Yes ______ (**GA011_W4_1_1**) Times
2. No → Skip to GA000_W4 GA000_W4

999. Refuse to answer → Skip to GA000_W4 GA000_W4

**PROCEDURE** :
If GA011_W4_1 = 1 and GA011_W4_1_1 = 1ask GA011_W4_2 and GA011_W4_3 GA011_W4_1 = 1 GA011_W4_1_1 = 1 GA011_W4_2 GA011_W4_3

GA011_W4_2 How much have you inherited? The original value when inherited was __ (**GA011_W4_2_1**) Yuan When did the inheritance occur? ______ (**GA011_W4_2_2**) Year

[Soft Check: GA011_W4_2_1 > 200, 000]

GA011_W4_3 From whom you inherite?

1. Parents
2. Parents-in-law
3. Children
4. Relatives
5. Others

**PROCEDURE** :
If GA011_W4_1 = 1, and GA011_W4_1_1 > 1 or missing, ask GA011_W4_4 and GA011_W4_5 GA011_W4_1 = 1 GA011_W4_1_1 > 1 GA011_W4_4 GA011_W4_5

GA011_W4_4 How much in total have you inherited? The original value when inherited was ______ (**GA011_W4_4_1**) Yuan

The amount for largest inheritance was ______ (**GA011_W4_4_2**) Yuan

When did the largest inheritance occur? ______ (**GA011_W4_4_3**) Year

[Soft Check: GA011_W4_4_2 > 200, 000]

GA011_W4_5 The largest inheritance was inerited from whom?

1. Parents
2. Parents-in-law
3. Children
4. Relatives
5. Others

Part 1_2: Other Household Members Wage Income and Individual-based transfers

GA000_W4 Please pick one member from the [Preload all household family members] who would be most familiar with the financial condition of the household?
[]

[IWER: Make sure no others are at present]

[INTRO: Wed like to ask you some questions regarding the incomes of OTHER members in your household. Your answers will be kept strictly confidential and will only be used for academic research]

**GA005 BRANCHPOINT**
Preload the other household members from Family Module. Ask GA005 to GA012_W4_5 to all other household members (excluding Main Respondent and the spouse of Main Respondent). If there are no other household members, Skip to GB001_W4 （ ） GA005 GA012_W4_5 GB001_W4

GA005 Did [preload other household members name] receive any wage and bonus income in the past year? []

1. Yes
2. No → Skip to GA007_W4 GA007_W4

997. Do not know → Skip to GA007_W4 GA007_W4

999. Refuse to answer → Skip to GA007_W4 GA007_W4

GA006_W4 How much did [preload other household members name] receive last year? ___ Yuan

[Soft Check: > 240, 000]

[IWER: IfR is unwilling to answer or does not remember, ask unfolding bracket questions]

GA006_bracket [CAPI: If Respondent is unwilling to answer, does not remember, or the input value is 0, please ask unfolding bracket questions here 0] How much did [preload other household members name] receive last year?
5,000/10,000/30,000/50,000/100,000 Yuan

GA006_W4_1 Does the above mentioned wage that [preload other household members name] earned excludes any insurance, income tax, public housing funds and other fees? []

1. Yes
2. No

997. Do not know

999. Refuse to answer → Skip to GA007_W4 GA007_W4

GA006_W4_2 What is the total amount of [preload other household members name]s insurance, income tax, public housing funds and other fees? [] /

1. ______ (**GA006_W4_2a**) Yuan/Year /
2. ______ (**GA006_W4_2b**) Yuan/Month /
3. About ______ (**GA006_W4_2c**) % of wage
4. No

997. Do not know

999. Refuse to answer

PROCEDURE :

If GA006_W4_2 = 1 but GA006_W4_2a = 0 / missingask the unfolding bracket questions

GA006_W4_2 = 1 GA006_W4_2a = 0

If GA006_W4_2 = 2 but GA006_W4_2b = 0 / missingask the unfolding bracket questions

GA006_W4_2 = 2 GA006_W4_2b = 0

If GA006_W4_2 = 3 but GA006_W4_2c = 0 / missingask the unfolding bracket questions

GA006_W4_2 = 3 GA006_W4_2c = 0

If GA006_W4_2 = 997, ask the unfolding questions GA006_W4_2 = 997

GA006_W4_2_bracket What is the total amount of [preload other household members name]s insurance, income tax, public housing funds and other fees? /
300/500/1,000/2,000/3,000 Yuan/Month /

GA007_W4 Did [preload other household members name] receive any of the following types of individual transfer income in the past year? (check all that apply) [ ] ()

1. Pensions (including wages from governments, public institutions and firms, supplemental pension of the firms,and income from such programs as rural pension insurance, Urban residents pension and commercial pension insurance, new rural social pension insurance) , // ______ (**GA007_W4_1**) Yuan

   [IWER: If R is unwilling to answer or does not remember, ask unfolding bracket questions here]

2. Unemployment compensation ______ (**GA007_W4_2**) Yuan 3. Pension voucher / ______ (**GA007_W4_3**) Yuan
4. Pension subsidy for the oldest old ______ (**GA007_W4_4**) Yuan

5. Workers compensation from Industrial Accident Compensation Insurance includes wage-replacement benefits, disability benefits, and survivors benefits ______ （**GA007_W4_5**） Yuan
6. Elderly family planning subsidies ______ （**GA007_W4_6**） Yuan
7. Medical aid ______ （**GA007_W4_7**） Yuan

   [F1: Medical Aid: Medical Aid for Serious Diseases refers to additional cash assistance outside of Medical Insurance Scope for urban and rural residents who are living difficultly caused by a major illness. Medical Aid play as final support outside the basic medical insurance F1
   ]
8. Other government subsidies please specify ______ (**GA007_W4_8_1**) ______ （**GA007_W4_8**） Yuan
9. Other social income sources please specify _ （**GA007_W4_9_1**） ______ （**GA007_W4_9**） Yuan
10. None of the above → Skip to GA012_W4_1 GA012_W4_1

PROCEDURE :

If GA007_W4 = 1 and GA007_W4_1 = 0 / missing, which means respondent has received pension but did not answer how much received, or the input value is 0 Yuan, ask GA008_W4_1_bracket GA007_W4 = 1 GA007_W4_1 = 0 0 GA008_W4_1_bracket

GA008_W4_1_bracket How much pension have [preload other household members name] received last year? /
400/600/1,000/3,000/20,000 Yuan/Year /

GA012_W4_1 Have [preload other household members name] ever inherited anything? [ ]

1. Yes ______ (**GA012_W4_1_1**) times
2. No → Skip to next household member

997. Do not know → Skip to next household member

999. Refuse to answer → Skip to next household member

PROCEDURE :

If GA012_W4_1 = 1 and GA012_W4_1_1 = 1, ask GA012_W4_2 and GA012_W4_3 GA012_W4_1 = 1 GA012_W4_1_1 = 1 GA012_W4_2 GA012_W4_3

GA012_W4_2 How much in total have [preload other household members name] inherited? The original value when inherited was [] ______ (**GA012_W4_2_1**) Yuan When did the inheritance occur? ______ (**GA012_W4_2_2**) Year

[Soft Check: GA012_W4_2_1 > 200, 000]

GA012_W4_3 From whom [preload other household members name] inherited[ ]

1. Parents of [preload other household members name] []
2. Parents-in-law of [preload other household members name] [ ]
3. Children of [preload other household members name] []
4. Relatives of [preload other household members name] []
5. Others

997. Do not Know

PROCEDURE :

If GA012_W4_1 = 1 andGA012_W4_1_1 > 1 or missingask GA012_W4_4 and GA012_W4_5

GA012_W4_1 = 1 GA012_W4_1_1 > 1 GA012_W4_4 GA012_W4_5

GA012_W4_4 How much in total have [preload other household members name] inherited

The original value when inherited was [] ______ (**GA012_W4_4_1**) Yuan

The amount for largest inheritance was ______ (**GA012_W4_4_2**) Yuan

When did the largest inheritance occur? ______ (**GA012_W4_4_3**) Year

[Soft Check: GA012_W4_4_2 > 200, 000]

GA012_W4_5 The largest inheritance of [preload other household members name] was inerited from whom? []

1. Parents of [preload other household members name] []
2. Parents-in-law of [preload other household members name] [ ]
3. Children of [preload other household members name] []
4. Relatives of [preload other household members name] []
5. Others

997. Do not know

PART 2 Household Agricultural Income and Expenditure

[INTRO: Next we will ask some questions about agricultural income and expenditure in your household]

GB001_W4 In the past year, did your household members, including you, your spouse, and other household members, engage in agricultural activities (including cropping, forestry, livestock,and fish), or selling agricultural products you produced at market? , ,

1. Yes
2. No → Skip to GC001 GC001

GB002_W4 Who engaged in agricultural work in the past year? (check all that apply) () [preload all household member name, include the MainRand Spouse] [ ]

Crops and Forestry Products

GB003 Did your household engage in cropping or forestry last year?

1. Yes
2. No → Skip to GB007 GB007

GB004 When was the most recent harvest? ______ 2009...2018 (**GB004_1**) Year ______ 0...12 (**GB004_2**) Month

[IWER: Mark the year using four digits. Take down the month as its actual number. For example, write January as 1 not 01, December as 12. If do not remember month, fill 0 : 4 1 101 12 120]

GB005 What is the total value of all crops and forestry products produced in the past year? _ (**GB005_1**) Yuan Among it, what is the value of the crops and forestry products that is home consumed? ______ (**GB005_2**) Yuan or percentage ______ (**GB005_3**) %

[Soft Check: GB005_1 > 75000]

[IWER: IfR is unwilling to answer or does not remember, ask unfolding bracket questions]

GB005_bracket [CAPI: If Respondent is unwilling to answer/does not remember, or the input value is 0 for GB005_1, please ask unfolding bracket questions here GB005_1 0] What is the total value of all crops and forestry products?
1,000/3,000/5,000/7,000/10,000 Yuan

GB005_W2_bracket [CAPIIf Respondent is unwilling to answer/does not remember, or the input value is 0 for GB005_2 and GB005_3, please ask unfolding bracket questions here GB005_2 GB005_3, 0] What is the total value of all crops and forestry products for household consump- tion?

1,000/3,000/5,000/7,000/10,000 Yuan

GB006 What was the total cost of producing crops (including vegetables and Chinese herbs) and forestry products in the past year? (including Seeds (including home-used seeds), Fertilizer, Organic fertilizer, Pesticide, Plastic sheets, Hiring labor (including with machine or animals), Land rents, Rents (excluding land rents), Irrigation, Fuel, Transportation, Processing, Marketing (including packaging, management fee)) , () () ( ) ( ) ______ Yuan

[soft check: 50,000 yuan]

[IWER: ifR is unwilling to answer or does not remember, ask unfolding bracket questions]

GB006_bracket [CAPIIf Respondent is unwilling to answer, does not remember, or the input value is 0, please ask unfolding bracket questions here 0 :] 300/600/1,000/2000/5,000 Yuan

Livestock and Fisheries

GB007 Has your family raised any livestock or fish in the past year? (including chicken, duck, cattle, pig, sheep, etc.)

1. Yes
2. No → Skip to GC001 GC001

GB008 What is the current value of all livestock (including chicken, duck, cattle, pig, sheep, etc.) and aquatic life? ______ Yuan

[Soft Check: > 100, 000]

[IWER: IfR is unwilling to answer or does not remember, ask unfolding bracket questions]

GB008_bracket [CAPI: If Respondent is unwilling to answer, does not remember, or the input value is 0, please ask unfolding bracket questions here 0] 500/1,500/3,000 /4,500 /9,000 Yuan

GB009 What were the value of all livestock and aquatic life you have at this time last year? Yuan

[Soft Check: > 100, 000]

[IWER: IfR is unwilling to answer or does not remember, ask unfolding bracket questions]

GB009_bracket [CAPI: If Respondent is unwilling to answer, does not remember, or the input value is 0, please ask unfolding bracket questions here 0] 500 /1,500 /2,500 /4,000 /8,000 Yuan

GB010 How much did you spend on purchasing new livestock and aquatic life in the past year? Yuan

[Soft Check: > 50, 000]

GB011 What was the value of all livestock and aquatic life that were sold or consumed in the past year? ? ______ (**GB011_1**) Yuan Among it, what is the amount or percent consumed at your home? ______ (**GB011_2**) Yuan ______ (**GB011_3**) %

[Soft Check: > 100, 000]

[IWER: IfR is unwilling to answer or does not remember, ask unfolding bracket questions]

GB011_bracket [CAPI: For GB011_1, if Respondent is unwilling to answer, does not remember, or the input value is 0, please ask unfolding bracket questions here GB011_1 0] What was the value of all livestock and aquatic life?

200 /900 /1,500 /2,500 /5,000 Yuan

GB011_w2_bracket [CAPI: For GB011_2 and GB011_3, if Respondent is unwilling to answer, does not remember, or the input value is 0, please ask unfolding bracket questions here GB011_2 GB011_3 0] What is the total value of all livestock and aquatic life for household consumption

200 /900 /1,500 /2,500 /5,000 Yuan

GB012 What was the value of all livestock byproducts produced (including the self consumption value) in the past year, including milk, wool (including cashmere, sheep or goatskin), and eggs? ______ (**GB012_1**) Yuan Among it, what is the amount or percent consumed at your home? ______ (**GB012_2**) Yuan ______ (**GB012_3**) %

[Soft Check: > 50, 000]

[IWER: IfR is unwilling to answer or does not remember, ask unfolding bracket questions]

GB012_bracket [CAPI: For GB012_1, if Respondent is unwilling to answer, does not remember, or the input value is 0, please ask unfolding bracket questions here GB012_1 0] What was the value of all livestock byproducts produced (including the self consumption value) in the past year?

100 /200 /300 /500 /1,000 Yuan

GB012_w2_bracket [CAPI: For GB012_2 and GB012_3, If Respondent is unwilling to answer, does not remember, or the input value is 0, please ask unfolding bracket questions here GB012_2 GB012_3 0] What was the total value for household consumption

100 /200 /300 /500 /1,000 Yuan

GB013 In the past year, how much did you spend on raising these livestock and aquatic products, including feeding fees, medical expenses, grazing fees, barn fence fees, and labor costs, etc.? ______ Yuan

[Soft Check: $> 50,000$]

PART 3 Self-Employed Activities

GC001 Did your household members engage in any self-employed activities last year?

1. Yes
2. No → Skip to GD001_W4_1 GD001_W4_1

GC002 How many types of activities did your household members participate in the past year?

**PROCEDURE** :<br>Ask all these GC002 activities in GC006_W4 GC006_W4 GC002

GC006_W4 Please briefly describe the self employed activities that your household member engaged, such as location/business type, etc.

1. ______ (**GC006_W4_1**)
2. ______ (**GC006_W4_2**)
3. ______ (**GC006_W4_3**)

...

GC002 ______ (**GC006_W4_i**)

PROCEDURE :
Please ask GC003 - GC005 for all self employed activities mentioned above. The description loaded from GC006_W4 GC003 - GC005 GC006_W4

GC003 Who engaged in this self-employment businesse [Preload Self Employeed Activity i] in the past year? (check all that apply) [i] ()
[Preload the list of all household members]

GC004 [Preload Self Employeed Activity i] which types of activities? [i]
1. Services (cooking, sewing, private clinic etc.) ( ) 2. Transportation
3. Construction
4. Mining
5. Processing production
6. Business
7. Others please specify ______ (**GC004_1**)

GC005 Not including fixed capital costs, what is your best estimate of the net income earned from this activity [Preload Self Employeed Activity i] by your household members last year? If the activity was conducted jointly with non-household members, report only the net income earned by household members. Remember to consider the following types of costs: energy, housing or equipment rental, raw materials, transportation, marketing, wages, taxes or fees [i]

______ Yuan

[Soft Check: > 500, 000]

[IWER: IfR is unwilling to answer or does not remember, ask unfolding bracket questions]

GC005_bracket [CAPI: If Respondent is unwilling to answer, does not remember, or the input value is 0, please ask unfolding bracket questions here 0] 5,000 /10,000 /50,000 /100,000 /200,000 Yuan

PART 4 Household Public Transfer Income

[INTRO: We ask the public transfers received by the households (with household as the unit). Public transfers have characteristic of welfare, such as Wubaohu Subsidy and Tekunhu Subsidy gived by government.]

GD001_W4_1 Does your household receive Dibao assistance? 1. Yes
2. No → Skip to GD003_W4_1 GD003_W4_1
999. Refuse to answer → Skip to GD003_W4_1 GD003_W4_1

GD001_W4_2 When did your household become a Dibaohu? ______ Year

GD001_W4_3 How much did you receive in the past year? __ Yuan

GD003_W4_1 Is your household a registered Pinkunhu? 1. Yes
2. No → Skip to GD002_W4 GD002_W4
997. Do not know → Skip to GD002_W4 GD002_W4
999. Refuse to answer → Skip to GD002_W4 GD002_W4

GD003_W4_2 When did you become a registered Pinkunhu? ______ Year

GD002_W4 Did your household receive any of the following government subsidies in the past year? (check all that apply) （ ）

1. Reforestation : how much? ______ (**GD002_W4_1**) Yuan
2. Agricultural subsidies : how much? ______ (**GD002_W4_2**) Yuan
3. Wubaohu (targets low-income, blind, disabled, aged persons, and young persons that have no means to support themselves) : how much? ______ (**GD002_W4_3**) Yuan
4. Pinkunhu subsidy, including registered Pinkunhu and non-registered Pinkunhu : how much? ______ (**GD002_W4_4**) Yuan
5. Work injury subsidies to the immediate family members how much? ______ (**GD002_W4_5**) Yuan
6. Emergency or disaster relief (jiujikuan, jiuzaikuan) last year? : how much? ______ (**GD002_W4_6**) Yuan
7. Social donations and subsidies, including food, clothes, school bags, crowdfunding medical expenses, and physical assistance : how much? ______ (**GD002_W4_7**) Yuan
8. Compensation for land seizure last? : how much? ______ (**GD002_W4_8**) Yuan

9. Compensation to pulling down your house or apartment last year? :how much? _____ (**GD002_W4_9**) Yuan
10. Other please specify ______(**GD002_W4_other**): how much? _ (**GD002_W4_10**) Yuan
11. None → Skip to GD004_W4 GD004_W4

[Soft Check: > 20, 000 for each item]

GD004_W4 Does your household receive any claims from Productive insurance in past year? How much? (input 0 if not received). Productive Insurance include Agricultural insurance claims, etc. （ 0） ______ Yuan

PART 5 Household Living Expenditure

[IWER: Please read aloud This section is about your household living expenditure, including all your household members [preloaded names of household members] living expenditure []]

GE000_W4 Generally, how much does your family spend a month? Including rent, food, clothing, communication expenses, water and electricity costs, fuel costs, service expenditures, entertainment expenditures, daily necessities and medical expenses. ______ Yuan

GE001 We wish to know your family food expenditure for the last week. Are you the primary person who purchases food for the household?

1. Yes → Skip to GE004 GE004
2. No

GE002 Who is the primary person purchasing food for the household? 1-25. Other household member list

26-50. Other children [List all the children who are not in the household member list] []

51. Nanny
52. Neighbor
53. Other
97. Main Respondent
98. Spouse of the main respondent

[IWER: If possible, the primary person who purchases food for the household should answer the questions about expenditures GE004 - GE008 GE004 - GE008]

GE004 In the past week, how many people usually ate meals together in your household (not including guests)? , ______ Persons

[Soft Check: > 10]

GE005 Last week how many meals did you provide to guests? ______ Meals

[Soft Check: > 100]

[INTRO: The next questions are about your household living expenditure for all household members, including the expenditure happened outside, such as accommodation and meals fees]

GE006_W4 In the past week, how much did your household spend on food (excluding eating out expenditure, alcohol, cigarettes, cigars and tobacco expenditure)? ______ Yuan

[Soft Check: > 6, 000]

GE006_W2 Does your household produce agricultural products yourself (including plants, meat, eggs, aquatic lives,oil, vegetables and fruits, cigaretts and wine, drinks and milk products, produced food, seasonings, etc.)? ,

1. Yes
2. No → Skip to GE007_W4 GE007_W4

GE006_W2_1 In the past week, what was the market value of the food that members of the household consumed that you grew yourselves? ______ Yuan

GE007_W4 Among it, how much did your household spend on eating out? ______ Yuan

[Soft Check: > 3, 000]

GE008 Among it, how much did your household spend on alcohol, Cigarettes, cigars and tobacco? ______ Yuan

[Soft Check: > 3, 000]

GE009 Please tell me the expenditure on the following items last month for your household

[IWER: Fill in 0 if no corresponding expenditure; missing if the respondent cannot recall the expenditure : 0]

1. Communication fees, including post, internet usage, telephone and cellphone usage (**GE009_1**) Yuan

2. Utilities: Water and electricity ______ (**GE009_2**) Yuan
3. Fuels, including gas, coal, etc. ______ (**GE009_3**) Yuan
4. Expenses for babysitters, housekeepers and servants __ (**GE009_4**) Yuan
5. Local Transportation ______ (**GE009_5**) Yuan
6. Daily necessities, including toiletries, household items, kitchen supplies, decorative items, etc. ______ (**GE009_6**) Yuan
7. Entertainment,including expenses for books, newspapers,VCCs, DVDs, cinematickets andbars ______ (**GE009_7**) Yuan

[Soft Check: > 5, 000 for each category]

PROCEDURE :

If GE009 = 4, babysisters, housekeepers and servants, ask GE009_W4_4 GE009 = 4 //GE009_W4_4
Otherwise, Skip to GE010 GE010

GE009_W4_4 Will the babysitters/hourly worker/servant employed by your household take care of the following people? (check all that apply) // ()

1. The elders in the household
2. the children in the household
3. Others please specify ______ (**GE009_W4_4_1**)

GE010 In the last year how much did your household spend on the following items?

[IWER: Fill in 0 if no corresponding expenditure; fill in missing if the respondent cannot recall the expenditure : 0]

1. Clothing and bedding ______ (**GE010_1**) Yuan
2. Long distance traveling expenses ______ (**GE010_2**) Yuan
3. Heating(centrally heated) ______ (**GE010_3**) Yuan
4. Furniture, consumption of durable goods and electronics, includes refrigerator, washing machine, TV, computers and expensive instruments like pianio. ______ (**GE010_4**) Yuan
5. Education and training(including tuition, training fees, etc.) ______ (**GE010_5**) Yuan
6. Direct and Indirect Medical expenses. Note: Indirect medical expenses refer to transportation expenses, nutrition expenses, family expenses, etc. incurred due

to medical treatment. It does not include the part already paid by Medicare (**GE010_6**) Yuan

7. Fitness expenditures ______ (**GE010_7**) Yuan
8. Beauty (including make-ups, facials, massages, etc.) ______ (**GE010_8**) Yuan
9. Automobiles ______ (**GE010_9**) Yuan
10. Purchase, Maintenance and repair (of transportation vehicles, appliances, communication products, etc.) ( ) ______ (**GE010_10**) Yuan
11. Property management fees (including parking fee) ______ (**GE010_11**) Yuan
12. Taxes and fees turned over to the government ______ (**GE010_12**) Yuan
13. Donations to the society (including cash, and items like food, clothing, etc.) ______ (**GE010_13**) Yuan
14. The rent of the house or bed, including the accommodation expenses for household members, such as the campus dormitory fee, excluding the hotel fee for tourism , , , ______ (**GE010_W4_14**) Yuan
15. Expenses for setting and holding banquets ______ (**GE010_W4_15**) Yuan

[Soft Check: > 100, 000 for each item]

GE011 How often did the respondent receive assistance in answering section Household income and expenditure?

[IWER: If it is answered by a proxy, please record the respondents reaction]

1. Never
2. A few times
3. Most or all of the time

HA Household Assets

[IWER: This section is asked of the family financial respondent. Do not allow a proxy respondent to answer the entire section]

PART 1 Land

[INTRO: The following questions pertain to your land]

HA054 Does [preload main respondents name] and [preload main respondents spouses name] (if any) have any collective distributing or rent cultivated land, forest land, pasture and/or pond? (Choose all that apply) [] [] ( ) ()

1. Cultivated land
2. Forest land
3. Pasture
4. Pond
5. None → Skip to HA064 HA064

**PROCEDURE** :<br>According to all options choosed in HA054, ask HA070_W4 - HA063 in loop HA054 HA070_W4 - HA063

HA070_W4 Have the right of [Load the chosed option in HA054] been confirmed by government? [HA054]

1. Yes
2. No → Skip to HA055 HA055

997. Do not Know → Skip to HA055 HA055

999. Refuse to answer → Skip to HA055 HA055

HA071_W4 When the right of [Load the chosed option in HA054] been confirmed? [HA054] Year

HA055 How many mu of [preload answer from HA054] do you and your spouse have? [ ] [] () [HA054] ______ Mu

[IWER: Only those assigned to the Main Respondent and the spouse are recorded here, and those assigned to other household members should not be included]

[Soft Check: > 50]

HA056 How many mu of them are irrigable? ______ Mu

[Hard Check: > HA055]

HA057 What is the rent per mu per year you would get if you rent out all your [preload answer from HA054]? [HA054] ______ Yuan per mu per year

[Soft Check: < 10, > 4000]

[IWER: IfR is unwilling to answer or does not remember, ask unfolding bracket questions]

HA057_W4_bracket [CAPI: If Respondent is unwilling to answer or does not remember, please ask unfolding bracket questions here 0] 50/100/300/600/1,000 yuan

**PROCEDURE** :<br>If HA057 = 0, ask HA057_W4_1 HA057 = 0 HA057_W4_1<br>Otherwise, Skip to HA058 HA058

HA057_W4_1 Why the rent is 0? 0

1. The profit is low
2. No one rents
3. Other please specify ______ (**HA057_W4_2**)

HA058 Do [preload main respondents name] and [preload main respondents spouses name] (if any) rent out any of [preload answer from HA054] in the past year? [ ] [] ( ) [HA054]

1. Yes
2. No → Skip to HA061 HA061

HA059 How much mu [preload answer from HA054] did [preload main respondents name] and [preload main respondents spouses name] (if any) rent out the past year? [] [] ( ) [HA054]______ Mu

[Soft Check: > HA055]

HA060 How much rental income did [preload main respondents name] and [preload main respondents spouses name] (if any) earn from [preload answer from HA054] in the past year? [] [] ( ) [HA054] ______ Yuan

HA061 Did [preload main respondents name] and [preload main respondents spouses name] (if any) rent in any [preload answer from HA054] from others (including the collective) in the past year? [] [] ( ) [HA054]

1. Yes
2. No → Skip to HA064 HA064

HA062 How much mu [preload answer from HA054] did [preload main respondents name] and [preload main respondents spouses name] (if any) rent at the past year? [ ] [] ( ) [HA054]______ Mu

[Soft Check: > 100]

HA063 How much rent did [preload main respondents name] and [preload main respondents spouses name] (if any) pay for [preload answer from HA054]? [ ] [] ( ) [HA054] ____ Yuan

[Soft Check: > 20, 000]

HA064 How much rental income did [preload main respondents name] and [preload main respondents spouses name] (if any) earn for any other household assets other than housing or land? (trees, use of fixed capital, durables, or livestock)? [] [] ( )

1. Yes ______ (**HA064_1**) Yuan
2. Not applicable
997. Do not know
999. Refuse to answer

PART 2 Equipments, Consumption Durables, and Valuables

HA065 Do [preload main respondents name] and [preload main respondents spouses name] (if any) own the following assets? (Choose all that apply) Only consider the part that owned if the asset is shared with others [] [ ] ( ) [] [ ] ( ) ( )

For each asset owned by main respondent and his/her spouse] what is the assets current value?

1. Automobile ______ (**HA065_1**)
2. Electric Bicycle ______ (**HA065_2**)
3. Motorcycle ______ (**HA065_3**)
4. Refrigerator ______ (**HA065_4**)
5. Washing machine ______ (**HA065_5**)
6. TV ______ (**HA065_6**)
7. Computer and pad ______ (**HA065_7**)
8. Stereo system ______ (**HA065_8**)
9. Video camera ______ (**HA065_9**)
10. Camera ______ (**HA065_10**)
11. Air conditioner ______ (**HA065_11**)
12. Mobile phone ______ (**HA065_12**)
13. Furniture ______ (**HA065_13**)
14. Music instrument ______ (**HA065_14**)
15. Valuable decorations, ornaments ______ (**HA065_15**)
16. Treasures and precious metal (such as gold) ______ (**HA065_16**)

17. Antiques, valuable paintings and calligraphic work, and other artistic work ______ (**HA065_17**)
18. Air Purifier ______ (**HA065_18**)
19. None

[Soft Check: $< 3,000$, $> 500,000$ for automobile, and < 100, $> 30,000$ for all other items]

HA066_W4 Do [preload main respondents name] and [preload main respondents spouses name] (if any) own the following fixed capital assets? How much are the assets worth? Only consider the part that owned if the asset is shared with others (check all that apply)[] [] ( ) [] [] ( ) ( )

1. Tractor current value ______ (**HA066_W4_1**) Yuan
   [Soft Check: < 1000, $> 30,000$]
2. Thresher current value ______ (**HA066_W4_2**) Yuan
   [Soft Check: < 100, $> 10,000$]
3. Harvester current value ______ (**HA066_W4_3**) Yuan
   [Soft Check: < 100, $> 10,000$]
4. Water pump current value ______ (**HA066_W4_4**) Yuan
   [Soft Check: < 100, $> 10,000$]
5. Processing equipment current value ______ (**HA066_W4_5**) Yuan
   [Soft Check: < 100, $> 10,000$]
6. Seeder current value ______ (**HA066_W4_6**) Yuan
   [Soft Check: < 100, $> 10,000$]
7. Agricultural aircraft current value ______ (**HA066_W4_7**) 10,000 Yuan
   [Soft Check: < 1, $> 1,000$]
8. None

HA067_W4 What is the current value of other fixed capital assets for [preload main respondents name] and [preload main respondents spouses name] (if any) used in household production or self-employed activities? Including business property, such as shops, factories and warehouses [] [] ( ) ______ Yuan

[IWER: Be sure to ask about fixed capital assets used in all self-employment activities, do not count assets already reported above]

HA068 Do [preload main respondents name] and [preload main respondents spouses name] (if any) have any other durable or fixed assets worth 500 yuan or more? [] [] ( )
500

1. Yes How much are the assets worth? ______ (**HA068_1**) Yuan

   [Hard Check: > 500] [Soft Check: > 50, 000]

2. No

997. Do not know

999. Refuse to answer

HA076 How often did the respondent receive assistance in answering section Household assets?

[IWER: If it is answered by a proxy, please record the respondents reaction]

1. Never
2. A few times
3. Most or all of the time

HB Individual Assets

[IWER: Please conduct sections HB and HC when the main respondent and his/her spouse are at home. Dont allow a proxy to complete the entire sections HC HD]

PART 1 Financial Assets

[INTRO: The following questions pertain to your financial asset]

[IWER: make sure others are not present,IWER read following instructions: the following questions pertain to your financial asset, the answers to these questions will be kept strictly confidential and will be used for research purposes only]

HC001 How much cash is held by you and your spouse at home? ______ Yuan

[Soft Check: > 50, 000, < 100]

[IWER: IfR is unwilling to answer or does not remember, ask unfolding bracket questions]

HC002 [CAPI: If Respondent is unwilling to answer, does not remember, or the input value is 0, please ask unfolding bracket questions here 0] 500 /1,000 /2,000 /5,000 /10,000 Yuan

[CAPI: Prompt for HC003_W4 - HC017_W4, add IWER: for electronic money, deposit, bonds, stocks, and funds, onlyinclude assets legally in his/her name HC003_W4 - HC017_W4]

HC003_W4 How much electronic money do you have currently, including the money in the WeChat Wallet and Alipays balance, etc? Exclude Yuebao and other products that generate revenue ______ Yuan

[Soft Check: > 1, 000]

[IWER: IfR is unwilling to answer or does not remember, ask unfolding bracket questions]

HC004_W4 [CAPI: If Respondent is unwilling to answer, does not remember, or the input value is 0, please ask unfolding bracket questions here 0] 10/50/200/1,000/5,000 Yuan

HC005 What is the total amount of deposits you are currently holding in financial institutions (eg: bank, credit union)? ______ Yuan

[Soft Check: > 500, 000, < 100]

[IWER: IfR is unwilling to answer or does not remember, ask unfolding bracket questions]

HC006 [CAPI: If Respondent is unwilling to answer, does not remember, or the input value is 0, please ask unfolding bracket questions here 0] 2,000/10,000/50,000/100,000/500,000 Yuan

HC007 Do you have any government bonds (e.g. Treasury bills, corporate bonds) in your name? （）

1. Yes
2. No → Skip to HC010 HC010

999. Refuse to answer → Skip to HC010 HC010

HC008 What is the total face value of government bonds (e.g. Treasury bills, corporate bonds) that you are currently holding? ? _ Yuan

[Soft Check: > 50, 000]

[IWER: IfR is unwilling to answer or does not remember, ask unfolding bracket questions]

HC009 [CAPI: If Respondent is unwilling to answer, does not remember, or the input value is 0, please ask unfolding bracket questions here 0] 10,000 /50,000 /100,000 /200,000 /500,000 Yuan

HC010 Do you hold any stocks (in your name) currently? （） 1. Yes

2. No → Skip to HC015 HC015

999. Refuse to answer → Skip to HC015 HC015

HC013 What is the present market value of all the stocks you are currently holding? ______ Yuan

[Soft Check: > 200, 000]

[IWER: IfR is unwilling to answer or does not remember, ask unfolding bracket questions]

HC014 [CAPI: If Respondent is unwilling to answer, does not remember, or the input value is 0, please ask unfolding bracket questions here 0] 10,000 /50,000 /100,000 /200,000 /500,000 Yuan

HC015 Do you hold any funds in your name currently? ( ) 1. Yes

2. No → Skip to HC016_W4 HC016_W4

999. Refuse to answer → Skip to HC016_W4 HC016_W4

HC018 What is the present market value of all the mutual funds you are currently holding? Yuan

[Soft Check: > 200, 000]

[IWER: IfR is unwilling to answer or does not remember, ask unfolding bracket questions]

HC019 [CAPI: If Respondent is unwilling to answer, does not remember, or the input value is 0, please ask unfolding bracket questions here 0] 10,000 /50,000 /100,000 /200,000 /500,000 Yuan

HC016_W4 Do you hold any other (in your name) wealth management products currently in addition to deposits, bonds, stocks, and funds? Including Yuebao, P2P, income insurance, etc. ( ) P2P

1. Yes

2. No → Skip to PROCEDURE before HC020 HC020

999. Refuse to answer → Skip to PROCEDURE before HC020 HC020

HC017_W4 What is the present market value of all the wealth management products you are currently holding? ______ Yuan

[Soft Check: > 200, 000]

[IWER: IfR is unwilling to answer or does not remember, ask unfolding bracket questions]

HC017_W4_bracket [CAPI: If Respondent is unwilling to answer, does not remember, or the input value is 0, please ask unfolding bracket questions here 0] 10,000 /50,000 /100,000 /200,000 /500,000 Yuan

PROCEDURE :

if HC005 = 0 and HC003_W4 = 0 and HC007 = 2 and HC010 = 2 and HC015 = 2 and HC016_W4 = 2, then skip HC020 HC005 = 0 HC003_W4 = 0 HC007HC010 HC015HC016_W4 2HC020

HC020 What percentage of the deposits, bonds, stocks, funds, and other wealth management products held in your name is fully controlled by you and not your spouse? (%) ______ 0...100 %

[Hard Check: ≥ 0, ≤ 100]

HC021 Do you have any other deposits, bonds, stocks, or funds that belong to you but not in your or your spouses name?

1. Yes
2. No → Skip to HC027 HC027

999. Refuse to answer → Skip to HC027 HC027

HC022 What is the value of such assets? _______ yuan

[Soft Check: > 200, 000]

HC027 Do you have public housing fund?

1. Yes
2. No → Skip to HC030 HC030

999. Refuse to answer → Skip to HC030 HC030

HC028 What is the total amount of money in your public housing fund? ______ Yuan

[Soft Check: > 100, 000]

[IWER: IfR is unwilling to answer or does not remember, ask unfolding bracket questions]

HC029 [CAPI: If Respondent is unwilling to answer, does not remember, or the input value is 0, please ask unfolding bracket questions here 0] 5,000/10,000/50,000/100,000/200,000 Yuan

HC030 Have you contributed any Jizikuan to your employer or other emploers and have not got it back? (Jizikuan is a type of fund that employers collect from workers for investment or building apartments, etc.)?

1. Yes
2. No → Skip to HC033 HC033

999. Refuse to answer → Skip to HC033 HC033

HC031 What is the amount of your jizikuan? _______ Yuan

[Soft Check: > 200, 000]

[IWER: IfR is unwilling to answer or does not remember, ask unfolding bracket questions]

HC032 [CAPI: If Respondent is unwilling to answer, does not remember, or the input value is 0, please ask unfolding bracket questions here 0] 5,000/10,000/50,000/100,000/200,000 Yuan

HC033 Do you have any unpaid salary that your employer still owes you?

1. Yes
2. No → Skip to HC039_W3 HC039_W3

999. Refuse to answer → Skip to HC039_W3 HC039_W3

HC034 What is the amount of your unpaid salary? _______ Yuan

[Soft Check: > 100, 000]

[IWER: IfR is unwilling to answer or does not remember, ask unfolding bracket questions]

HC035 [CAPI: If Respondent is unwilling to answer, does not remember, or the input value is 0, please ask unfolding bracket questions here 0] 5,000/10,000 /50,000 /100,000 /200,000 Yuan

HC039_W3 Have you lent to other families or individuals and not been repaid by them?

1. Yes
2. No → Skip HC040_W3 and HC041_W3 HC040_W3 HC041_W3

999. Refuse to answer → Skip HC040_W3 and HC041_W3 HC040_W3 HC041_W3

HC040_W3 What is the total amount of the loans? _______ Yuan

[Soft Check: > 500, 000]

[IWER: IfR is unwilling to answer or does not remember, ask unfolding bracket questions]

HC041_W3 [CAPI: If Respondent is unwilling to answer, does not remember, or the input value is 0, please ask unfolding bracket questions here 0] 5,000/10,000/50,000/100,000/200,000 Yuan

PART 2 Debts

[INTRO: The following questions pertain to your debt]

HD001 What is the total amount of loans that you havent repaid yet (not including mortage and credit card balance)? Please fill in 0 if there is no loan

0______ Yuan

[Soft Check: > 50, 000]

[IWER: IfR is unwilling to answer or does not remember, ask unfolding bracket questions]

HD002 [CAPI: If Respondent is unwilling to answer or does not remember, please ask unfolding bracket questions here]
5,000/10,000/50,000/100,000/500,000 Yuan

HD003 Do you have any credit card? A credit card includes Jingdong Baitiao and Huabei. If so, what is the amount of your credit card balance? If not, please fill in 0 0_____ Yuan

[Soft Check: > 50, 000]

[IWER: IfR is unwilling to answer or does not remember, ask unfolding bracket questions]

HD004 [CAPI: If Respondent is unwilling to answer or does not remember, please ask unfolding bracket questions here]
500/1,000/5,000/10,000/50,000 Yuan

HD004_W3 What is the total amount of money you owe to other families, individuals, or employers? Exclude the loans and credit card debt mentioned above. Please fill in 0 if you owe no money 0______ Yuan

[Soft Check: > 50, 000]

[IWER: IfR is unwilling to answer or does not remember, ask unfolding bracket questions]

HD004_W3_1 [CAPI: If Respondent is unwilling to answer or does not remember, please ask unfolding bracket questions here]
500/1,000/5,000/10,000/50,000 Yuan

HD006_W4 Has someone tried to defraud you in the past year? There is no need to have lost property

1. Yes
2. No → Skip to HD005_W4 HD005_W4

999. Refuse to answer → Skip to HD005_W4 HD005_W4

HD007_W4 What is the type of the fraud?

1. Telecommunications fraud

2. Fundraising event fraud
3. Fraudulent pyramid scheme
4. Sales fraud
5. Other please specify ______ (**HD007_W4_1**)

HD008_W4 What is the amount of money you lost? If no loss, fill in 0 Yuan 0 ______ Yuan

HD005_W4 Have you experienced fraud in the past with property loss? Not including that you experienced in the past year

1. Yes in total ______ (**HD005_W4_0**) times
2. No → Skip to HD012 HD012

999. Refuse to answer → Skip to HD012 HD012

HD005_W4_1 If ever experienced, what is the amount of money you lost? Consider only that with the largest number of losses ______ Yuan

HD005_W4_2 Which year? Consider only that with the largest number of losses ______

HD012 How often did the respondent receive assistance in answering section HASSETS?

[IWER: If it is answered by a proxy, please record the respondents reaction.]

1. Never
2. A few times
3. Most or all of the time

HA&I House Property and Housing Characteristics

**Preloaded Variables from the Last Waves Interview:**	
**ZLocation_*i*_**	Location of ith house in last IW<br>*i*
**ZSize_*i*_**	Size of ith house in last IW<br>*i*
**ZTimeBuy_*i*_**	Time of purchase of ith house in last IW<br>*i*
**ZTimePrice_*i*_**	Purchase price of ith house in last IW<br>*i*
**ZValueLastTime_*i*_**	Market value of ith house in last IW<br>*i*
**ZHA000_W4_1_*i*_**	Whether i^{th} house have ownership information, 1 for yes, 0 for no<br>*i* 1 0
**ZHA000_W4_2_*i*_**	String expression for i^{th} houses ownership information in last IW<br>*i*
**Z_N**	Totol number of house properties preloaded for the household

HA House Property

[IWER: Ask financial respondent to answer questions in this section. Do not allow others to completely answer the entire section]

[INTRO: The following questions pertain to the house properties of [preload main respondents name] and [preload main respondents spouses name] (if any) [] []()]

HA000_W4_0 BRANCHPOINT :

Skip to HA000_W4_0 if this is a new household HA000_W4_0

Skip to HA000_W4_1 BRANCHPOINT if previously interviewed HA000_W4_1 BRANCHPOINT

HA000_W4_0 How many house properties are in [preload main respondents name] and [preload main respondents spouses name] (if any)? [] [ ]() ______ houses

[IWER: A property under somebodys name is that [load main respondents name] or [load main respondents spouses name] (if any) is on the property right certificate. Fill in 0 if they have no property [] []() 0]

HA000_W4_1 BRANCHPOINT :
Skip to HA024_W4 BRANCHPOINTif this is a new household HA024_W4 BRANCHPOINT

[CAPI: Preload information about all the house properties recorded in the last wave, i.e. the houses with property rights. For each of the house properties, ask loop questions from HA000_W4 to HA022_W4 Bracket. Skip to HA023_W4 when the loop ends HA000_W4 - HA022_W4 Bracket (1) HA023_W4]

[INTRO: Now we would like to know more about the house(s) you mentioned on the last interview]

HA000_W4 The interviewer confirms the following table to the respondent. If correct, it does not need to be modified, if not, modify it directly in the table, noting that the time of purchase, the purchase price and the market value in [ZIWTime] cannot be modified [ZIWTime]

[CAPI: The location and size of the house can be directly modified. But the time of purchase, the purchase price and the market value in [ZIWTTime] cannot be modified [ZIWTime]]

[IWER: Do not check the Do not know this house box just because the house doesnt exist this year (e.g. it was demolished), or because the house is not owned by main respondent and spouse this year (due to sale or other reasons) ( ) ( )]

				[ZIWTime]
(ZLocation_i_)	(ZSize_i_)	(ZTimeBuy_i_)	(ZPriceBuy_i_)	(ZValueLastTime_i_)

2. Do not know this house → Skip to the next house property (if any) ( )

PROCEDURE :
1. Preload house property information from the previous interview
Skip to HA000_W4_2 if house property information is blank, e.g. ZHA000_W4_1 = 0 ZHA000_W4_1 = 0 HA000_W4_2
If not (ZHA000_W4_1 = 1), Skip to HA000_W4_1 ZHA000_W4_1 = 1 HA000_W4_1

HA000_W4_1 In [ZIWTime], the ownership structure of [preload house information] is like this [preload house property information in the previous interview ZHA000_W4_2], correct? [ZIWTime] [] [ZHA000_W4_2]

1. Yes → Skip to HA001_W4_0 HA001_W4_0
2. No

999. Refuse to answer

HA000_W4_2 In [ZIWTime], what is the ownership structure of [preload house information] (check all that apply) [ZIWTime] [] （）

[IWER: Check Other, specify the reason, and fill in 100 (%) for the percentage if the house is not related to the main respondent and his/her spouse, or to other relatives or friends at that time (e.g. renting from unrelated people) （） 100（%）]

1. [Preload the name(s) of the main respondent and his/her spouse (if any)] [ ] ______ (**HA000_W4_2_1**) %
2. [Preload main repsondents name]s children, son-in-law, daughter-in-law [ ]
3. [Preload main repsondents name]s siblings [] __ (**HA000_W4_2_3**) %
4. [Preload main repsondents name]s parents, parents-in-law []
5. [Preload main repsondents name]s grandchildren []
6. [Preload main repsondents name]s other relatives [] ______ (**HA000_W4_2_6**) %
7. [Preload main repsondents name]s friend(s) [] ______ (**HA000_W4_2_7**) %
8. Other please specify ______ (**HA000_W4_2_9**) ______ (**HA000_W4_2_8**) %
9. No property right certificate. But [Preload the name(s) of the main respondent and his/her spouse (if any)] has(ve) the rights to use and inherit, but no right to sell [ ]

999. Refuse to answer

PROCEDURE :

Ask HA000_W4_3 and HA000_W4_4 if child(ren), son-in-law or daughter-in-law has property rights of the house (HA000_W4_2 = 2) (HA000_W4_2 = 2) HA000_W4_3 HA000_W4_4

HA000_W4_3 Which children or spouses of children? (check all that apply) （）

[Preload the list of children]

PROCEDURE :

For each child selected in HA000_W4_3, ask HA000_W4_4 HA000_W4_3 HA000_W4_4

HA000_W4_4 The share of [preload names of children in HA000_W4_3] or spouse in house property rights is? [HA000_W4_3] [] ______ %

PROCEDURE :
Ask HA000_W4_5 and HA000_W4_6 if parents or parents-in-law own property rights to the house (HA000_W4_2 = 4) (HA000_W4_2 = 4) HA000_W4_5 HA000_W4_6

HA000_W4_5 Which parents or parents-in-law? (check all that apply) / ()
[Preload list of parents/parents-in-law]

PROCEDURE :
For each parent selected in HA000_W4_5, ask HA000_W4_6 HA000_W4_5 HA000_W4_6

HA000_W4_6 The share of [preload name of parents / parents-in-law inHA000_W4_5] in house property is? [HA000_W4_5] [] _____ %

PROCEDURE :
Ask HA000_W4_7 and HA000_W4_8 if grandchildren have property rights of the house (HA000_W4_2 = 5) (HA000_W4_2 = 5) HA000_W4_7 HA000_W4_8

HA000_W4_7 These grandchildren are children of which of your children? (check all that apply) / ()
[Preload the list of children]

PROCEDURE :
For each child selected in HA000_W4_7, ask HA000_W4_8 HA000_W4_7 HA000_W4_8

HA000_W4_8 The share of [preload names of children in HA000_W4_7]s child in house property is? [HA000_W4_7] [] ______ %

HA001_W4_0 Has the ownership structure of [preload house information] changed now compared to when it was [ZIWTime]? [ZIWTime] []

1. Yes, changed
2. No, no change

997. Do not know
999. Refuse to answer

PROCEDURE：

If ZHA000_W4_1 = 0 and HA000_W4_2 ≠ 999 and HA001_W4_0 = 2, or ZHA000_W4_1 = 1 and HA001_W4_0 = 2, 999 and HA000_W4_2 ≠ 999 and HA001_W4_0 = 2, i.e. if the respondent answered the detailed ownership structure and there is no change until now, then Skip to HA004_W4 BRANCHPOINT ZHA000_W4_1 = 0 HA000_W4_2 ≠ 999 HA001_W4_0 = 2 ZHA000_W4_1 = 1 HA001_W4_0 = 2, 999 HA000_W4_2 ≠ 999 HA001_W4_0 = 2 HA004_W4 BRANCHPOINT

Otherwise, ask HA001_W4_1 HA001_W4_1

HA001_W4_1 What is the ownership structure of [preload house information] now (check all that apply)? [] （）

[IWER: Check Other, specify the reason, and fill in 100% if the house was demolished or sold to someone you Do not know 100%]

1. [Preload the name(s) of the main respondent and his/her spouse (if any)] [ ] ______ (**HA001_W4_1_1**) %
2. [Preload the name(s) of the main respondent and his/her spouse (if any)] [ ]
3. [Preload main repsondents name]s siblings [] __ (**HA001_W4_1_3**) %
4. [Preload main repsondents name]s parents, parents-in-law []
5. [Preload main repsondents name]s grandchildren []
6. [Preload main repsondents name]s other relatives [] ______ (**HA001_W4_1_6**) %
7. [Preload main repsondents name]s friend(s) [] ______ (**HA001_W4_1_7**) %
8. Other please specify ______ (**HA001_W4_1_9**) ______ (**HA001_W4_1_8**) %
9. No property right certificate. But [preload the name(s) of the main respondent and his/her spouse (if any)] has(ve) the rights to use and inherit, but no right to sell [ ]

997. Do not know

999. Refuse to answer

PROCEDURE：

Ask HA001_W4_2 and HA001_W4_3 if child(ren), or child(ren)-in-law has property right to the house (HA001_W4_1 = 2) (HA001_W4_1 = 2) HA001_W4_2 HA001_W4_3

HA001_W4_2 Which children or spouses of children? (check all that apply) ()
[Preload the list of children]

PROCEDURE :
For each child selected in HA001_W4_2, ask HA001_W4_3 HA001_W4_2 HA001_W4_3

HA001_W4_3 The share of [preload name of children in HA001_W4_2] or spouse in house property rights is? [HA001_W4_2]) [] ______ %

PROCEDURE :
Ask HA001_W4_4 and HA001_W4_5 if parents or parents-in-law have property rights to the house (HA001_W4_1 = 4) (HA001_W4_1 = 4) HA001_W4_4 HA001_W4_5

HA001_W4_4 Which parents / parents-in-law? (check all that apply) / ()
[Preload list of parents/parents-in-law]

PROCEDURE :
For each parent selected in HA001_W4_4, ask HA001_W4_5 HA001_W4_4 HA001_W4_5

HA001_W4_5 The share of [preload names of parents / parents-in-law in HA001_W4_4] in house property is? [HA001_W4_4] [] ______ %

PROCEDURE :
Ask HA001_W4_6 and HA001_W4_7 if grandchildren own property rights to the house (HA001_W4_1 = 5) (HA001_W4_1 = 5) HA001_W4_6 HA001_W4_7

HA001_W4_6 These grandchildren of children of which of your children? (check all that apply) / ()
[Preload the list of children]

PROCEDURE :
For each child selected in HA001_W4_6, ask HA001_W4_7 HA001_W4_6 HA001_W4_7

HA001_W4_7 The share of [preload childrens names in HA001_W4_6]s child in house property is? [HA001_W4_6] [] ______ %

HA004_W4 BRANCHPOINT :

Ask questions from HA004_W4 to HA007_W4 Bracket if HA001_W4_1 = 1, 9, or (HA000_W4_2 = 1, 9) and HA001_W4_0 = 2, i.e., the main respondent or his/her spouse owns property right to the house currently HA001_W4_1= 1, 9（HA000_W4_2 = 1, 9）HA001_W4_0 = 2 HA004_W4 HA007_W4 Bracket

Ask questions from HA010_W4 to HA022_W4 Bracket if ZHA000_W4_1 = 1 and HA000_W4_1 = 1 and HA001_W4_0 = 1 and (HA001_W4_1 ≠ 1, 9), or ZHA000_W4_1 = 1 and (HA000_W4_1 = 2, 999) and (HA000_W4_2 = 1, 9) and HA001_W4_0 = 1 and (HA001_W4_1 ≠ 1, 9), or ZHA000_W4_1 = 0 and (HA000_W4_2 = 1, 9) and HA001_W4_0 = 1 and (HA001_W4_1 ≠ 1, 9), i.e., the main respondent or his/her spouse previously owned property right to the house, but now does not ZHA000_W4_1 = 1 HA000_W4_1 = 1 HA001_W4_0 = 1 HA001_W4_1≠ 1, 9）ZHA000_W4_1 = 1（HA000_W4_1 = 2, 999）（HA000_W4_2 = 1, 9） HA001_W4_0 = 1 （HA001_W4_1 ≠ 1, 9）ZHA000_W4_1 = 0 （HA000_W4_2 = 1, 9） HA001_W4_0 = 1 （HA001_W4_1 ≠ 1, 9） HA010_W4 HA022_W4 Bracket

In other cases, end this loop and Skip to the next house property, if any

HA004_W4 What is the current market value of the house [preload house info]? That is, how much would the house sell for if it were sold now [] Total Price ______ (**HA004_W4_1**) 10,000 Yuan or Unit Price ______ (**HA004_W4_2**) 1,000 Yuan/square meter /

[IWER: IfR is unwilling to answer or does not remember, ask unfolding bracket questions]

HA004_W4 Bracket [CAPI: If Respondent is unwilling to answer, does not remember, or the input value is 0, please ask unfolding bracket questions here 0]
Unit Price 1,000/3,000/5,000/8,000/15,000 Yuan/Square Meter /

HA005_W4 Is the house [preload house info] for rent? [] 1. Yes
2. No → Skip to HA007_W4 HA007_W4
999. Refuse to answer → Skip to HA007_W4 HA007_W4

HA006_W4 What is the monthly rent of the house [preload house information]? [ ] ______ Yuan/Month /

[IWER: IfR is unwilling to answer or does not remember, ask unfolding bracket questions]

HA006_W4 Bracket [CAPI: If Respondent is unwilling to answer, does not remember, or the input value is 0, please ask unfolding bracket questions here 0]
Monthly rent 500/1,000/2,000/5,000/8,000 Yuan/Month /

HA007_W4 What is the market rent for the house [load house info]? [] ______ Yuan/month /
[IWER: IfR is unwilling to answer or does not remember, ask unfolding bracket questions]

HA007_W4 Bracket [CAPI: If Respondent is unwilling to answer, does not remember, or the input value is 0, please ask unfolding bracket questions here 0]
Market rent 500/1,000/2,000/5,000/8,000 Yuan/month /

[CAPI: End the loop and Skip to the next house property, if any]

HA010_W4 What is the reason why the house [load house info] does not belong to [preload name of main respondent] and [preload name of main respondents spouse] (if any)? [ ] [] []()

1. Sale
2. Demolished
3. Grants
4. Other

**PROCEDURE**：<br>Ask questions from HA011_W4 to HA012_W4 Bracket if the house is sold (HA010_W4 = 1)（HA010_W4 = 1） HA011_W4 HA012_W4 Bracket

HA011_W4 When was the house [preload house information] sold? [] ______ (**HA011_W4_1**) Year ______ (**HA011_W4_2**) Month

HA012_W4 What is the net income received from the sale of the house after deducting related expenses? ______ 10,000 Yuan
[IWER: IfR is unwilling to answer or does not remember, ask unfolding bracket questions]

HA012_W4 Bracket [CAPI: If Respondent is unwilling to answer, does not remember, or the input value is 0, please ask unfolding bracket questions here 0]
The net income 20,000/50,000/100,000/200,000/500,000 Yuan

[CAPI: End the loop and Skip to the next house property, if any ,]

PROCEDURE：
Ask questions from HA013_W4 to HA015_W4 Bracket if the house is demolished (HA010_W4 = 2)（HA010_W4 = 2）HA013_W4 HA015_W4 Bracket

HA013_W4 Did your family receive any compensation for the demolition of the house [preload house info]? []

1. Yes
2. No → End the loop and Skip to the next property, if any ,

999. Refuse to answer → End the loop and Skip to the next property, if any ,

HA014_W4 When did your family receive compensation for the demolition of the house?
(**HA014_W4_1**) Year ______ (**HA014_W4_2**) Month

HA015_W4 What was the total amount of money your family received for the demolition?
10,000 Yuan
[IWER: IfR is unwilling to answer or does not remember, ask unfolding bracket questions]

HA015_W4 Bracket [CAPI: If Respondent is unwilling to answer, does not remember, or the input value is 0, please ask unfolding bracket questions here 0]
Total amount of money 20,000/50,000/100,000/200,000/500,000 Yuan

[CAPI: End the loop and Skip to the next house property, if any ,]

PROCEDURE：
Ask questions from HA016_W4 to HA019_W4 Bracket if the house is gifted out (HA010_W4 = 3)（HA010_W4 = 3）HA016_W4 HA019_W4 Bracket

HA016_W4 To whom was the house [preload house information] gifted? (check all that apply) []（）

1. [Preload name of the main respondent]s children, son-in-law, daughter-in-law [ ]
2. [Preload name of the main respondent]s sibling(s) []
3. [Preload name of the main respondent]s parents, or parents-in-law [ ]

4. [Preload name of the main respondent]s grandchildren []
5. [Preload name of the main respondent]s other relatives []
6. [Preload name of the main respondent]s friends []
7. Other please specify ______ (**HA016_W4_1**)

PROCEDURE :
Ask HA016_W4_2 if the gift was made to a child, son-in-law or daughter-in-law (HA016_W4 = 1) （HA016_W4 = 1） HA016_W4_2

HA016_W4_2 Which child(ren) / child(ren)s spouse(s)? (check all that apply) / （）
[Preload the list of children]

PROCEDURE :
Ask HA016_W4_3 if the gift was made to parents or parents-in-law (HA016_W4 = 3) （HA016_W4 = 3） HA016_W4_3

HA016_W4_3 Which parents / parents-in-law? (check all that apply) / （ ）
[Preload list of parents/parents-in-law]

PROCEDURE :
Ask HA016_W4_4 if the gift was made to grandchildren (HA016_W4 = 4) （HA016_W4 = 4） HA016_W4_4

HA016_W4_4 These grandchildren are children of which of your children? (check all that apply) （）
[Preload the list of children]

HA017_W4 When was the house [preload house information] gifted to someone else? [ ] _ (**HA017_W4_1**) Year ______ (**HA017_W4_2**) Month

HA018_W4 Did the person who acquired the house property offer your family any compensation in cash or in kind?
1. Yes
2. No → End the loop, Skip to the next property, if any ,

999. Refuse to answer → End the loop, Skip to the next property, if any ,

HA019_W4 What is the total amount of compensation to your family? In-kind included ___ 1,000 Yuan

[IWER: IfR is unwilling to answer or does not remember, ask unfolding bracket questions]

HA019_W4 Bracket [CAPI: If Respondent is unwilling to answer or does not remember, please ask unfolding bracket questions here 0]
The total amount of compensation 5,000/10,000/50,000/100,000/200,000 Yuan

[CAPI: End the loop and Skip to the next house property, if any ,]

**PROCEDURE** :<br>Ask questions from HA020_W4 to HA022_W4 Bracket if the house is handled in other ways (HA010_W4 = 4) （HA010_W4 = 4） HA020_W4 HA022_W4 Bracket

HA020_W4 You said that the house [preload house info] is not owned by [preload main respondent name] and [preload his/her spouse] (if any), then how is it handled? [ ] [] [] （） ______

HA021_W4 When was it [Preload house information] disposed of? [] ______ (**HA021_W4_1**) Year ______ (**HA021_W4_2**) Month

HA022_W4 What is the net income from handling of the house [load house info], in-kind included? [] ______ 10,000 Yuan

[IWER: IfR is unwilling to answer or does not remember, ask unfolding bracket questions]

HA022_W4 Bracket [CAPI: If Respondent is unwilling to answer, does not remember, or the input value is 0, please ask unfolding bracket questions here 0]
20,000/50,000/100,000/200,000/500,000 Yuan

[CAPI: End the loop and Skip to the next house property, if any ,]

HA023_W4 Is there any other house property owned by [load name of main respondent] and [load name of main respondents spouse] (if any) in addition to the properties listed below? fill in 0 if not [Preload properties that the main respondent and his/her spouse own property rights] [] [] （） 0______

[]

HA024_W4 BRANCHPOINT :

If it is a new respondent and the number of house properties is not 0, i starts at 1 and ends at the number of properties recorded in HA000_W4_0. Skip to HA054_W3when the loop ends 0 i 1 HA000_W4_0 HA054_W3

If the respondent has previously been asked, i starts at 1 and ends at the number of properties recorded in HA023_W4. Skip to HA054_W3 when the loop ends 0 i 1 HA023_W4 HA054_W3

Skip to HA054_W3 if the number of newly acquired house properties is 0 0 HA054_W3

HA024_W4[i] The ith new house you mentioned is located in i ___ (**HA024_W4_1**[i]) province-city-county/district --/

______ (**HA024_W4_2**[i]) county/district-town/village/street-village/community /-// -/

______ (**HA024_W4_4**[i]) No./Building /

HA025_W4[i] Is the house [preload house property information] located in a rural or urban area? []

1. City or town central areas
2. Town or semi-rural areas
3. Rural areas
4. Special regions

HA026_W4[i] What type of house is the property [preload property information]? [ ]

1. Unit buildings
2. Detached house
3. Row housing
4. Courtyard house
5. Compound
6. Work shed
7. Irregular construction
8. Other please specify ______ (**HA026_W4_1**[i])

HA027_W4[i] Does the house [preload house property information] belong exclusively to [preload name of main respondent] and [preload name of his/her spouse] (if any)? []
[] [] ( )

1. Yes → Skip to HA035_W4 HA035_W4
2. No

HA028_W4[i] What is the ownership structure of the house now [preload house information] (check all that apply)? [] ( )

1. [Preload the name(s) of the main respondent and his/her spouse (if any)] [ ] ______ (**HA028_W4_1**[i]) %
2. [Preload main repsondents name]s children, son-in-law, daughter-in-law [ ]
3. [Preload main repsondents name]s siblings [] __ (**HA028_W4_3**[i]) %
4. [Preload main repsondents name]s parents, parents-in-law []
5. [Preload main repsondents name]s grandchildren []
6. [Preload main repsondents name]s other relatives [] ______ (**HA028_W4_6**[i]) %
7. [Preload main repsondents name]s friend(s) [] ______ (**HA028_W4_7**[i]) %
8. Other please specify ______ (**HA028_W4_9**[i]) ______ (**HA028_W4_8**[i]) %
9. No property right certificate. But [preload the name(s) of the main respondent and his/her spouse (if any)] have the rights to use and inherit, but no right to sell []

999. Refuse to answer

**PROCEDURE** :
Ask HA029_W4 and HA030_W4 if child(ren), son-in-law or daughter-in-law own property right to the house (HA028_W4 = 2) （HA028_W4 = 2） HA029_W4 HA030_W4

HA029_W4[i] Which children / spouses of children? (check all that apply) / （）

[Preload the list of children]

**PROCEDURE** :
For each child selected in HA029_W4[i], ask HA030_W4[i] HA029_W4[i] HA030_W4[i]

HA030_W4[i] The share of [preload names of children in HA029_W4[i]] or spouse in house property rights is? [HA029_W4[i]]/ [] _____ %

**PROCEDURE** :
Ask HA031_W4 and HA032_W4 if parents or parents-in-law own property rights to the house (HA028_W4 = 4) （HA028_W4= 4）HA031_W4 HA032_W4

HA031_W4[i] Which parents / parents-in-law? (check all that apply) / ()

[Preload list of parents/parents-in-law]

**PROCEDURE** :<br>For each parent selected in HA031_W4[i], ask HA032_W4[i] HA031_W4[i] HA032_W4[i]

HA032_W4[i] The share of [preload names of parents/parents-in-law inHA031_W4[i]] in house property is? [HA031_W4[i]] [] ______ %

**PROCEDURE** :<br>Ask HA033_W4 and HA034_W4 if grandchildren own property rights to the house (HA028_W4 = 5) （HA028_W4= 5）HA033_W4 HA034_W4

HA033_W4[i] These grandchildren are children of which of your children? (check all that apply) / ()

[Preload the list of children]

**PROCEDURE** :<br>For each child selected in HA033_W4[i], ask HA034_W4[i] HA033_W4[i] HA034_W4[i]

HA034_W4[i] The share of [preload childrens names in HA033_W4[i]]s child in house property is [HA033_W4[i]] [] ______ %

HA035_W4[i] [Preload house information] What is the floor area of the house? [] ______ Square meter(s)

HA036_W4[i] What is the market value of the house [preload house information] now? That is, how much would the house sell for if it were sold now [] Total price ______ (**HA036_W4_1**[i]) 10,000 Yuan or unit price ______ (**HA036_W4_2**[i]) 1,000 Yuan/Square metre /

[IWER: IfR is unwilling to answer or does not remember, ask unfolding bracket questions]

HA036_W4[i] **Bracket** [CAPI: If Respondent is unwilling to answer, does not remember, or the input value is 0, please ask unfolding bracket questions here 0]

unit price 1,000/3,000/5,000/8,000/15,000 Yuan/Square metre /

HA037_W4[i] What year was the house [preload house information] acquired or built? [ ] _ Year

HA038_W4[i] How much did it cost to acquire or build the house [preload house information]? [] ______ 10,000 Yuan

HA039_W4[i] Is the house [preload house information] for rent? []

1. Yes
2. No → Skip to HA041_W4 HA041_W4

999. Refuse to answer → Skip to HA041_W4 HA041_W4

HA040_W4[i] What is the monthly rent of the house [preload house information]? [ ] ______ Yuan/month /

[IWER: IfR is unwilling to answer or does not remember, ask unfolding bracket questions]

HA040_W4[i] **Bracket** [CAPI: If Respondent is unwilling to answer, does not remember, or the input value is 0, please ask unfolding bracket questions here 0]
The monthly rent 500/1,000/2,000/5,000/8,000 Yuan/month /

HA041_W4[i] What is the market rent of the house [preload house information]? [ ] ______ Yuan/Month /

[IWER: IfR is unwilling to answer or does not remember, ask unfolding bracket questions]

HA041_W4[i] **Bracket** [CAPI: If Respondent is unwilling to answer, does not remember, or the input value is 0, please ask unfolding bracket questions here 0]
The market rent 500/1,000/2,000/5,000/8,000 Yuan/Month /

[CAPI: End the loop and Skip to the next house property, if any]

HA054_W3 Do you or other household members take mortage to purchase your and your spouses houses now?

[IWER: Ask questions for all house properties owned by the respondent and his/her spouse, excluding houses with paid off mortgage]

1. Yes
2. No → End section HA Housing

999. Refuse to answer → End section HA Housing

HA055_W3 What is the outstanding amount of the loans? What is the outstanding amount of the loans including the principal and interest? ______ 10,000 Yuan

HA056_W3 How much is the unpaid interest? ______ 10,000 Yuan

HA057_W3 What is the monthly mortgage payment? ______ 10,000 Yuan

[Soft Check: $> 20,000$]

I Housing Characteristics

PROCEDURE：
Only main respondent answer I000_W4 - I026 I000_W4 - I026

I000_W4 Which of the following houses are you currently living in?

[CAPI: The serial number of house in this question is assigned by loaded house serial number and then assigned by new house seiral number]

1-25. [Preload list of house properties] [] → Skip to I002 I002 99. None of the above

I000_W4_1 The house you are living in is located at
______ (**I000_W4_1_1**) province-city-county/district --/
______ (**I000_W4_1_2**) town-village/street office-neighborhood association -/ -
______ (**I000_W4_1_4**) No./Building /

I000_W4_2 Is the house that you are living in in rural or urban area?

1. City or town central areas
2. Town or semi-rural areas
3. Rural areas
4. Special regions

I000_W4_3 What type of building does the house you are living in belong to?

1. Unit buildings
2. Detached house
3. Row housing
4. Courtyard house
5. Compound
6. Work shed
7. Irregular construction
8. Other please specify ______ (**I000_W4_3_1**)

I000_W4_4 What is the ownership of the house you are living in? (check all that apply) （）

[IWER: Check Other, specify the reason, and fill in 100 (%) in the percentages if the house is not related to the main respondent and his/her spouse, or to other relatives or friends at that time (e.g. renting from unrelated people) （） 100（%）]

1. [Preload the name(s) of the main respondent and his/her spouse (if any)] [()] _ (**I000_W4_4_1**) %
2. [Preload main repsondents name]s children, son-in-law, daughter-in-law [ ]
3. [Preload main repsondents name]s siblings [] __ (**I000_W4_4_3**) %
4. [Preload main repsondents name]s parents, parents-in-law []
5. [Preload main repsondents name]s grandchildren []
6. [Preload main repsondents name]s other relatives [] ______ (**I000_W4_4_6**) %
7. [Preload main repsondents name]s friend(s) [] ______ (**I000_W4_4_7**) %
8. Other please specify ______ (**I000_W4_4_9**) ______ (**I000_W4_4_8**) % 9. No property right certificate. But [preload the name(s) of the main respondent and his/her spouse (if any)] has(ve) the rights to use and inherit, but no right to sell [()]

999. Refuse to answer

PROCEDURE :
Ask I000_W4_5 and I000_W4_6 if child(ren), son-in-law or daughter-in-law owns property right to the house (I000_W4_4 = 2) (I000_W4_4 = 2) I000_W4_5 I000_W4_6

I000_W4_5 Which children/spouses of children? (check all that apply) / ()
[Preload the list of children]

PROCEDURE :
For each child selected in I000_W4_5, ask I000_W4_6 I000_W4_5 I000_W4_6

I000_W4_6 The share of [preload names of children in I000_W4_5] and spouse in house property rights is? [I000_W4_5] [] ______ %

PROCEDURE :
Ask I000_W4_7 and I000_W4_8 if parents or parents-in-law own property rights to the house (I000_W4_4 = 4) (I000_W4_4= 4) I000_W4_7 I000_W4_8

I000_W4_7 Which parents/parents-in-law? (check all that apply) / ( )
[Preload list of parents/parents-in-law]

PROCEDURE :
For each parent selected in 000_W4_7, ask I000_W4_8 000_W4_7 I000_W4_8

I000_W4_8 The share of [preload names of parents/parents-in-law in I000_W4_7] in house property is? [I000_W4_7] [] ______ %

PROCEDURE :
Ask I000_W4_9 and I000_W4_10 if grandchildren own property rights to the house (I000_W4_4 = 5) （I000_W4_4 = 5）I000_W4_9 I000_W4_10

I000_W4_9 Which of your sons or daughters are these grandchildren? (check all that apply) / ( )
[Preload the list of children]

PROCEDURE :
For each child selected in I000_W4_9, ask I000_W4_10 I000_W4_9 I000_W4_10

I000_W4_10 The share of [preload child(ren)s name(s) inI000_W4_9]s child in house property is? [I000_W4_9] [] ______ %

PROCEDURE :
Skip to the I000_W4_12 if the main respondent or his/her spouse owns property right to the house where they live in (I000_W4_4 = 1, 9) （I000_W4_4 = 1, 9）I000_W4_12
Ask I000_W4_11 then Skip to I000_W4_13 if the main respondent or his/her spouse owns no property right to the house where they live in (I000_W4_4 ≠ 1, 9) （I000_W4_4 ≠ 1, 9 ）I000_W4_11 I000_W4_13

I000_W4_11 What is the usable area of the house you are living in? ______ Square metres

I000_W4_12 What is the floor area of the house you are living in? ______ Square meters

I000_W4_15 What is the market value of the house now? That is, how much the house would sell for if it were sold now Total price ______ （**I000_W4_15_1**）10,000 Yuan or unit price ______ （**I000_W4_15_2**）1,000 Yuan/Square meter /
[IWER: IfR is unwilling to answer or does not remember, ask unfolding bracket questions]

I000_W4_15 Bracket [CAPI: If Respondent is unwilling to answer, does not remember, or the input value is 0, please ask unfolding bracket questions here 0]
unit price 1,000/3,000/5,000/8,000/15,000 1,000 Yuan/Square meter /

I000_W4_16 Which year was the house acquired or built? ______ Year

I000_W4_17 How much money was spent to acquire or build this house? ______ 10,000 Yuan

I000_W4_13 What is the monthly rent for the house? ______ Yuan/Month /
[IWER: If don t have to pay for the rent, fill 0. If R is unwilling to answer or does not remember, ask unfolding bracket questions 0]

I000_W4_13 Bracket [CAPI: If Respondent is unwilling to answer, does not remember, or the input value is 0, please ask unfolding bracket questions here 0]
The monthly rent 500/1,000/2,000/5,000/8,000 Yuan/Month /

I000_W4_14 What is the market rent for the house? ______ Yuan/Month /
[IWER: IfR is unwilling to answer or does not remember, ask unfolding bracket questions]

I000_W4_14 Bracket [CAPI: If Respondent is unwilling to answer, does not remember, or the input value is 0, please ask unfolding bracket questions here 0]
The market rent 500/1,000/2,000/5,000/8,000 Yuan/Month /

I002 What is the total area of the homestead of the house you are living in? (Homestead is the land allocated by rural collective economic organizations to farmers for building houses and small gardens to meet their living needs.) ? ( )
______ m^2
[Soft Check: < 10, > 1000]
[IWER: If no entry/not applicable, please fill in 0 0]

I003 Is your residence used for business as well? 1. Yes
2. No

I004 What type of structure is this building? 1. Concrete and steel/Bricks and wood

2. Adobe /
3. Wood/Thatched /
4. Cave dwelling
5. Mongolian yurt/Woolen felt/Tent //
6. Stone
7. Other please specify ______ (**I004_1**)

I005 When was this house built? ______ Year

**PROCEDURE** :<br>Ask I005_1 if I005 is missing, i.e. not sure when the house was built I005 I005_1<br>Otherwise, skip to I006 I006

I005_1 IfR is unclear about year, please choose among following items

1. 0 – 5 years 0 – 5
2. 5 – 10 years 5 – 10
3. 10 – 20 years 10 – 20
4. 20 – 30 years 20 – 30
5. 30 – 40 years 30 – 40
6. More than 40 years 40

I006 Is the building one story or multi-level building?

1. One-story building
2. Common multi-story building → Skip to I008 I008
3. Self-contained multi-story building → Skip to I009 I009

I007 Is the story independent or compound?

1. Independent story
2. Compound

**PROCEDURE** :<br>Skip to I010_W4 I010_W4

I008 Which story is this building on? ______ Storey

**PROCEDURE** :<br>If I008 > 1, ask I009 I008 > 1 I009<br>Otherwise, skip to I010_W4 I010_W4

I009 Does it have elevator

1. Yes
2. No

I010_W4 Are there any handicapped facilities (e.g., non-stair ramp)? （ ）

1. Yes
2. No
3. No steps on level ground, no need for handicapped facilities

PROCEDURE :
If I010_W4 = 2, ask I011 I010_W4 = 2 I011

I011 How many steps had to be climbed to get to the main entrance of the households flat?
[IWER: Do not count steps if an elevator is available]

1. 0 step 0
2. 1 to 5 steps 1 – 5
3. 6 to 15 steps 6 – 15
4. 16 to 25 steps 16 – 25
5. More than 25 steps 25

I012 How many bedrooms, living rooms, bathrooms, and kitchens are there in your residence?
(**I012_1**) bedrooms ______ (**I012_2**) living rooms ______ (**I012_3**) toilets （） ______ (**I012_4**) kitchens ______ (**I012_5**) balcony
[Soft Check: > 20]

PROCEDURE :
Ask I013 if no toilets in the answer to I012 I012 I013

I013 How far is the nearest toilet to your house? ______ Meters
[Soft Check: > 500]

I014 What is the type of toilet? Is it with or without a seat? If the respondent has both, select toilet with a seat 1. Toilet without a seat

2. Toilet with a seat → Skip to I016 I016

I015 Is the toilet flushable?

1. Yes
2. No

I016 Does your residence have electricity?

1. Yes
2. No

I017 Does your residence have running water?

1. Yes
2. No

I018 Is there in-house shower or bath facility? What type? 1. Concentration supply of hot warter

2. Water heater installed by the household
3. No

I019 Does your residence have coal gas or natural gas supply? 1. Yes

2. No

I020 Does your residence have heating? (does not include AC with heating or self-made heating) ( )

1. Yes
2. No → Skip to I022_W4 I022_W4

I021_W4 What is the main heating energy source? 1. Solar

2. Coal
3. Natural gas
4. Liquefied Petroleum Gas
5. Electric
6. Crop residue/Wood burning
7. Other please specify ______ (**I021_1**)
8. Concentration heating

I022_W4 What is the main source of cooking fuel? 1. Coal

2. Natural gas
3. Marsh gas
4. Liquefied Petroleum Gas
5. Electric
6. Crop residue/Wood burning
7. Other please specify ______ (**I022_1**)
8. Do not cook

I023 Does your residence have a telephone connection?

1. Yes
2. No

I024 Does your residence have broad-band internet connection?

1. Yes
2. No

I027_W3 Does your residence have an air cleaner in your home?

1. Yes
2. No

I025 [Interviewer records it] How clear and tidy is in this household

1. Excellent
2. Very clear
3. Clear
4. Fair
5. Poor
6. Not applicable

I026 [Interviewer records it] How is the temperature in this household

1. Very hot
2. Hot
3. Bearable
4. Cold
5. Very cold
6. Not applicable
